# Efficacy of Messenger RNA–1273 Against Severe Acute Respiratory Syndrome Coronavirus 2 Acquisition in Young Adults From March to December 2021

**DOI:** 10.1093/ofid/ofad511

**Published:** 2023-11-02

**Authors:** Kathryn E Stephenson, Jasmine R Marcelin, Audrey E Pettifor, Holly Janes, Elizabeth Brown, Moni Neradilek, Catherine Yen, Jessica Andriesen, Nicole Grunenberg, Nicole Espy, Meg Trahey, Rebecca S B Fischer, Christopher A DeSouza, Joanna L Shisler, Elizabeth Connick, Eric R Houpt, Helen Y Chu, Russel J McCulloh, Sylvia Becker-Dreps, Nadja A Vielot, Corey A Kalbaugh, Kartik Cherabuddi, Karen M Krueger, Molly Rosenberg, Richard N Greenberg, Arnel Joaquin, Lilly Cheng Immergluck, Lawrence Corey, James G Kublin

**Affiliations:** Center for Virology and Vaccine Research, Beth Israel Deaconess Medical Center, Boston, Massachusetts, USA; Ragon Institute of MGH, MIT and Harvard, Cambridge, Massachusetts, USA; Division of Infectious Diseases, Department of Medicine, University of Nebraska Medical Center, Omaha, Nebraska, USA; Department of Epidemiology, University of North Carolina at Chapel Hill, Chapel Hill, North Carolina, USA; Vaccine and Infectious Disease Division, Fred Hutchinson Cancer Center, Seattle, Washington, USA; Vaccine and Infectious Disease Division, Fred Hutchinson Cancer Center, Seattle, Washington, USA; Vaccine and Infectious Disease Division, Fred Hutchinson Cancer Center, Seattle, Washington, USA; Division of AIDS, National Institute of Allergy and Infectious Diseases, National Institutes of Health, Rockville, Maryland, USA; Vaccine and Infectious Disease Division, Fred Hutchinson Cancer Center, Seattle, Washington, USA; Vaccine and Infectious Disease Division, Fred Hutchinson Cancer Center, Seattle, Washington, USA; Vaccine and Infectious Disease Division, Fred Hutchinson Cancer Center, Seattle, Washington, USA; Vaccine and Infectious Disease Division, Fred Hutchinson Cancer Center, Seattle, Washington, USA; Department of Epidemiology and Biostatistics, School of Public Health, Texas A&M University, College Station, Texas, USA; Department of Integrative Physiology, University of Colorado, Boulder, Colorado, USA; Department of Microbiology, University of Illinois, Urbana, Illinois, USA; Department of Medicine, University of Arizona, Tucson, Arizona, USA; Division of Infectious Diseases and International Health, University of Virginia, Charlottesville, Virginia, USA; Department of Medicine, University of Washington, Seattle, Washington, USA; Children's Hospital and Medical Center, University of Nebraska Medical Center, Omaha, Nebraska, USA; Department of Epidemiology, University of North Carolina at Chapel Hill, Chapel Hill, North Carolina, USA; Department of Family Medicine, University of North Carolina at Chapel Hill, Chapel Hill, North Carolina, USA; Department of Family Medicine, University of North Carolina at Chapel Hill, Chapel Hill, North Carolina, USA; Department of Public Health Sciences, Clemson University, Clemson, South Carolina, USA; Department of Medicine, University of Florida, Gainesville, Florida, USA; Department of Medicine, Northwestern University Feinberg School of Medicine, Chicago, Illinois, USA; Center for Sexual Health Promotion, Indiana University School of Public Health–Bloomington, Bloomington, Indiana, USA; Department of Medicine, University of Kentucky, Lexington, Kentucky, USA; Department of Medicine, Charles R. Drew University of Medicine and Science, Los Angeles, California, USA; Department of Microbiology, Biochemistry and Immunology, Morehouse School of Medicine, Atlanta, Georgia, USA; Vaccine and Infectious Disease Division, Fred Hutchinson Cancer Center, Seattle, Washington, USA; Department of Medicine, University of Washington, Seattle, Washington, USA; Department of Laboratory Medicine, University of Washington,Seattle, Washington, USA; Vaccine and Infectious Disease Division, Fred Hutchinson Cancer Center, Seattle, Washington, USA

**Keywords:** COVID-19, SARS-CoV-2 infection, lifestyle circumstances, mRNA-1273 vaccine

## Abstract

**Background:**

The efficacy of messenger RNA (mRNA)–1273 against severe acute respiratory syndrome coronavirus 2 (SARS-CoV-2) infection is not well defined, particularly among young adults.

**Methods:**

Adults aged 18–29 years with no known history of SARS-CoV-2 infection or prior vaccination for coronavirus disease 2019 (COVID-19) were recruited from 44 US sites from 24 March to 13 September 2021 and randomized 1:1 to immediate vaccination (receipt of 2 doses of mRNA-1273 vaccine at months 0 and 1) or the standard of care (receipt of COVID-19 vaccine). Randomized participants were followed up for SARS-CoV-2 infection measured by nasal swab testing and symptomatic COVID-19 measured by nasal swab testing plus symptom assessment and assessed for the primary efficacy outcome. A vaccine-declined observational group was also recruited from 16 June to 8 November 2021 and followed up for SARS-CoV-2 infection as specified for the randomized participants.

**Results:**

The study enrolled 1149 in the randomized arms and 311 in the vaccine-declined group and collected >122 000 nasal swab samples. Based on randomized participants, the efficacy of 2 doses of mRNA-1273 vaccine against SARS-CoV-2 infection was 52.6% (95% confidence interval, −14.1% to 80.3%), with the majority of infections due to the Delta variant. Vaccine efficacy against symptomatic COVID-19 was 71.0% (95% confidence interval, −9.5% to 92.3%). Precision was limited owing to curtailed study enrollment and off-study vaccination censoring. The incidence of SARS-CoV-2 infection in the vaccine-declined group was 1.8 times higher than in the standard-of-care group.

**Conclusions:**

mRNA-1273 vaccination reduced the incidence of SARS-CoV-2 infection from March to September 2021, but vaccination was only one factor influencing risk.

**Clinical Trials Registration:**

NCT04811664.

## Teaser

Two doses of messenger RNA-1273 had 52.6% efficacy against severe acute respiratory syndrome coronavirus 2 (SARS-CoV-2) infection and 71.0% efficacy against symptomatic coronavirus disease 2019 (COVID-19) during the Delta wave. Vaccine-Declined participants reported different lifestyle circumstances than randomized participants.

The messenger RNA (mRNA)–1273 vaccine (ModernaTX) [1, 2] received Food and Drug Administration emergency use authorization (EUA) in the United States (US) for individuals aged ≥18 years on 20 December 2020 and full approval on 31 January 2022 [3]. The mRNA-1273 vaccine showed 94.1% efficacy for preventing symptomatic coronavirus disease 2019 (COVID-19) due to the ancestral variant over 2 months [4, 5] and 93.2% efficacy through 6 months. From December 2020 to December 2022 >342 million doses of the mRNA-1273 vaccine were administered in the United States [6]. The estimated efficacy against symptomatic COVID-19 was >90% after 2 doses during the initial phase of the pandemic [7–10], 80%–84% after 2 doses during the Delta variant wave in the United States [11–14], and 61% after 3 doses during the BA.1/BA.2 Omicron wave [15].

Data on mRNA-1273 vaccine efficacy against infection (including asymptomatic infection) are more limited, especially by variant and population characteristics. In observational studies, estimates of mRNA-1273 effectiveness against all infections varied from 53% to 84% against Delta and from 14% to 44% against Omicron [11, 16–18]. Young adults are particularly at risk of acquiring and transmitting severe acute respiratory syndrome coronavirus 2 (SARS-CoV-2) [19, 20], highlighting the importance of characterizing vaccine efficacy in this population, yet rigorous, prospective studies focusing on young adults remain scant.

To fill this gap, in March 2021, CoVPN 3006 (ClinicalTrials.gov NCT04811664) was launched, recruiting young adults in the United States, and using prospective, daily nasal swab SARS-CoV-2 reverse-transcription polymerase chain reaction (PCR) testing. Participants were randomized to immediate vaccination (receipt of 2 doses of mRNA-1273 vaccine at months 0 and 1) or the standard of care (SoC; receipt of COVID-19 vaccine per federal, state, and local guidelines or mRNA-1273 at months 4 and 5 if the vaccine was not previously received off study). Later, an observational group of individuals who declined COVID-19 vaccination was added. Here, we report efficacy of the mRNA-1273 vaccine against SARS-CoV-2 infection diagnosed by means of PCR (a primary end point) and against symptomatic COVID-19 (a secondary end point). We also describe lifestyle circumstances for this important population.

## METHODS

### Participants and Randomization

In the final version of the protocol, this randomized controlled, open-label trial enrolled young adults at 44 University and healthcare sites. Eligible participants were aged 18–29 years, with no known history of SARS-CoV-2 infection or prior vaccination for COVID-19. Protocols for inclusion and exclusion criteria are provided in the Supplementary Materials. Participants for the randomized arms were enrolled from March 24 through 13 September 2021 and assigned to immediate vaccination (immediate arm; eg, receipt of 2 doses of mRNA-1273 vaccine at months 0 and 1) or SoC (eg, receipt of COVID-19 vaccine per federal, state, and local guidelines or 2 doses of mRNA-1273 provided by the study team at month 4 and 28 days later at month 5, if vaccine was not received previously off study) in a 1:1 ratio using a centralized interactive response technology system. During this period Pfizer, Moderna, and Janssen vaccines were available [21]. Randomization was stratified and done in blocks to ensure balance among contemporaneously evaluated immediate and SoC arms by study site and type of residence (eg, dormitory, apartment).

Initially, adults aged 18–26 years were eligible if they were enrolled in a higher education institution, as the social living situation of university students made them more susceptible to SARS-CoV-2 infection and transmission than others in the age group, and participants randomized to the SoC arm were to be vaccinated at the end of the 4-month study. Recruitment for these participants primarily occurred through flyers, tabling, word- of- mouth, student email lists and other strategies that took advantage of local university communication networks. These outreach activities were informed by the CoVPN 3006 Youth Advisory Board comprising >40 students representing 20 participating universities.

In May 2021, federal vaccine recommendations expanded to include all adults aged ≥18 years, reducing the pool of eligible unvaccinated young adults [22]. Thus, in June 2021 the protocol was amended: the upper age limit at enrollment was raised from 26 to 29 years, and the requirement to recruit only students was removed. The amendment clarified that participants in the SoC arm could be vaccinated outside the study at any time. A vaccine-declined observational group was also added for participants who declined COVID-19 vaccination but otherwise satisfied the eligibility criteria and enrolled from June 16 through 8 November 2021. This group provided additional data on SARS-CoV-2 infection incidence but was not included in primary vaccine efficacy analyses. All participants in the vaccine-declined group were encouraged to receive COVID-19 vaccination and instructed to report external vaccinations to the study. Recruitment strategies shifted to reach these new populations, with sites reporting success with posting flyers at laundromats, nail and hair salons, apartment complex mailboxes, and other neighborhood settings, giving talks at faith-based organizations, and using mobile units to reach rural areas.

### Vaccine

Two mRNA-1273 vaccine doses were provided to the immediate and SoC arms, as described above, by ModernaTX through the US government COVID-19 vaccine response and administered as intramuscular injections given 28 days apart, in a volume of 0.5 mL containing 100 µg of mRNA-1273. Site vaccination providers were referred to EUA vaccine instructions at https://www.modernatx.com/COVID19vaccine-eua/.

### Adverse Event Reporting

Adverse events were reported to the Vaccine Adverse Event Reporting System (VAERS) (https://vaers.hhs.gov/reportevent.html) and/or the US Centers for Disease Control and Prevention (CDC) V-safe program (https://www.cdc.gov/vaccinesafety/ensuringsafety/monitoring/v-safe/index.html). VAERS is comanaged by the CDC and the Food and Drug Administration and serves as the reporting mechanism for adverse events occurring from licensed and EUA vaccines, including the mRNA-1273 vaccine used in this study. Study vaccination providers reported adverse events to VAERS after COVID-19 vaccination according to the CDC (https://www.cdc.gov/vaccines/COVID-19/vaccination-provider-support.html and https://vaers.hhs.gov/faq.html). Any safety issues or eligibility questions outside the scope of VAERS or V-safe reporting were referred to the protocol safety review team for discussion.

### Daily Nasal Swab Samples

Infection was assessed by detection of viral RNA in nasal swab samples. Participants were instructed to perform self-swabs on the anterior nares daily starting at vaccination or day 1 (depending on group) and continuing for 16 weeks and to return swab samples to sites 3 times per week. Testing with reverse-transcription PCR (SCV2-SPX-EP Molecular Test [Corteva Agriscienc] or LabGold ultra-high-throughput SARS-CoV-2 end-point PCR [Northwell Health Laboratories]) was performed on every-other-day specimens, and a positive PCR result triggered testing of swab samples from 3 days before to 14 days after the positive specimen or until viral RNA was no longer detected. Positive PCR results were reported back to the site and participant. The infecting variant was determined by Spike sequencing of the peak viral load swab sample using Illumina NextSeq 500 and Illumina NextSeq 2000 whole-genome sequencing.

### Infection Serology, Symptom Surveillance, and Lifestyle Circumstance Assessment

Participants were assessed for SARS-CoV-2–binding antibodies specific to the SARS-CoV-2 nucleocapsid protein (Roche Elecsys Anti-SARS-CoV-2 Immunoassay [University of Washington Retrovirology Laboratory, Seattle]) at weeks 0, 8, and 16. COVID-19 symptom surveillance was performed weekly by means of eDiary. In the event of SARS-CoV-2 infection, participants provided an additional serum sample for SARS-CoV-2 clinical serology and performed daily eDiary symptom tracking for 28 days. Lifestyle circumstance questionnaires pertinent to potential SARS-CoV-2 exposure were administered at baseline and weekly thereafter.

### Trial Oversight, Patient Consent Statement, and Interim Monitoring

The trial was conducted in accordance with the International Council for Harmonisation of Technical Requirements for Pharmaceuticals for Human Use, Good Clinical Practice guidelines, and applicable government regulations. A central institutional review board approved the protocol and consent forms, and local institutional review boards approved site-specific consent forms and documents as appropriate. Participants provided written informed consent before enrollment.

Interim data on baseline participant characteristics and capture of study end points were reviewed by an independent data monitoring committee. Study feasibility and operations were reviewed by an oversight group including the study sponsor (National Institute of Allergy and Infectious Diseases) and CoVPN leadership. On 12 November 2021, following a protracted enrollment period due largely to rapid rollout of COVID-19 vaccines in the community (all US adults were eligible to be vaccinated by May 2021 [23] and by November 2021 about 68% of the population had received ≥1 vaccine) [24], the study was terminated early. Participant follow-up was completed on 26 January 2022. Reported clinical data corresponds to the final clinical database; the laboratory data cutoff was 25 August 2022.

### Statistical Analysis

The trial was designed to reject the null hypothesis that vaccine efficacy against SARS-CoV-2 infection was ≤30%, with 90% power under 57% vaccine efficacy assuming 4% incidence without vaccination over a 4-month follow-up period. A total of 2565 person-years of follow-up was required, equally balanced between vaccinated and unvaccinated person-time. With early stopping and high rates of outside vaccination, the study accrued 214.1 person-years of follow-up for the primary efficacy analysis, including 80.4 unvaccinated person-years, 43.8 following a single mRNA-1273 dose, and 89.9 fully vaccinated (after 2 doses of mRNA-1273) person-years.

Follow-up for efficacy analyses started at the time of first nasal swab sample. Participants without SARS-CoV-2 infection were censored at the last negative swab sample result, no later than their outside vaccination date and no later than study vaccination for the SoC arm (henceforth “outside vaccination censoring”). Primary analyses were conducted in the “primary efficacy cohort,” defined as randomized participants in the full analysis set who collected nasal swab samples and who were SARS-CoV-2 negative at baseline based on PCR of the first nasal swab sample and serology of the first blood sample. Vaccine efficacy was estimated among randomized participants in the primary efficacy cohort using outside vaccination censoring and a site-stratified Cox proportional hazards model with calendar time scale (ie, time since study opened) and a time-dependent indicator for the number of mRNA-1273 doses received. The model included baseline covariates associated with outside vaccination or SARS-CoV-2 infection absent vaccination: sex assigned at birth, residence, team sport participation, mask wearing, and race/ethnicity (Supplementary Methods). Secondary analyses were conducted among randomized participants in the full analysis set with PCR data, including those who were SARS-CoV-2 positive at baseline, and post–outside vaccination follow-up using “intention-to-treat (ITT) censoring” whereby participants were censored at their last negative swab sample, no later than study vaccination for the SoC arm.

The secondary end point, symptomatic COVID-19, was defined by PCR-confirmed SARS-CoV-2 infection and concurrent symptoms captured by daily or weekly symptom reporting (symptoms in Supplementary Materials). Participants without concurrent symptom data were assumed to be not symptomatic and were censored at SARS-CoV-2 infection. Vaccine efficacy against symptomatic COVID-19 was estimated using the same time-dependent Cox regression model as for the primary end point (Supplementary Materials).

In addition to the analyses above, we compared SARS-CoV-2 incidence between the vaccine-declined group and SoC arm, using a site-stratified Cox regression model with calendar time scale and outside vaccination censoring. Analyses were performed using R software, version 4.0.4, and SAS software, version 9.4. All statistical tests were significant at the .05 level and 2 sided, and no adjustment for multiplicity was performed.

## RESULTS

### Study Population

**Figure 1.**
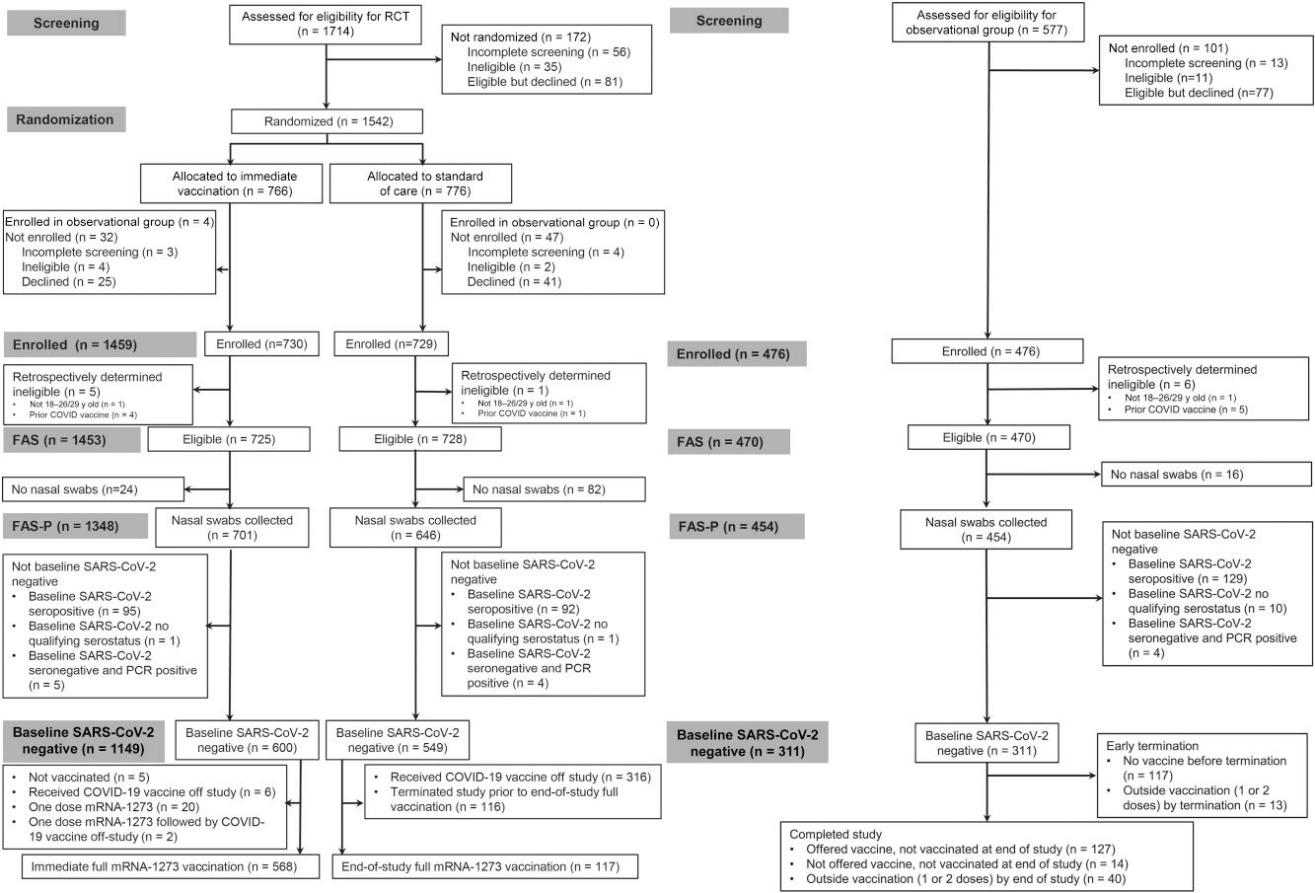
Consort diagram of randomized and observational groups. Abbreviations: COVID-19, coronavirus disease 2019; FAS, full analysis set; FAS-P, full analysis set with PCR data; mRNA, messenger RNA; PCR, polymerase chain reaction; SARS-CoV-2, severe acute respiratory syndrome coronavirus 2.

A total of 1542 participants underwent randomization; 83 randomized participants were subsequently not enrolled (Figure 1). Of the 1459 enrolled participants, 310 (130 in the immediate group, 180 in the SoC group) were excluded from primary efficacy analyses, either because the participants had no nasal swab samples collected (n = 106), were not SARS-CoV-2 negative at baseline (n = 198), or were retrospectively determined to be ineligible (n = 6). Approximately 15% of eligible participants with nasal swab sample data were SARS-CoV-2 positive at baseline (196 of 1348; 187 seropositive and 9 PCR positive only). Of the remaining participants, 600 in the immediate group and 549 in the SoC group were included in the primary efficacy analyses. More than 94% of participants (568 of 600) in the primary efficacy cohort of the immediate group received both mRNA-1273 doses. A total of 476 participants were enrolled in the observational vaccine-declined group. Approximately 29% of eligible participants (133 of 454) in the vaccine-declined group who collected nasal swab samples were positive for SARS-CoV-2 infection at baseline.

Among baseline SARS-CoV-2–negative participants, the median follow-up before SARS-CoV-2 infection or outside vaccination censoring was 105 days in the immediate and 44 days in the SoC arm; this differential was primarily due to uptake of outside vaccination, with 1.3% versus 58% receiving off-study vaccination, respectively. With continued encouragement by study staff to be vaccinated, 17% of the vaccine-declined group (53 of 311) received outside vaccination. Follow-up for primary efficacy analyses took place between March 26 and 31 December 2021 (Supplementary Table 1).

### Baseline Demographics

.Of all the participants, 56% were female sex assigned at birth (Table 1); 15.1% in the vaccine-declined group and 8.4% of randomized participants identified as Black or African American, and 22.8% in the vaccine-declined group and 17.8% of randomized participants identified as Hispanic or Latino/a. About 47% of participants in both the randomized arms and the vaccine-declined group lived in an apartment building or condominium, 39% of all participants lived with >2 people (40% in the immediate randomized arm and 37% in the vaccine-declined group). The randomized arms comprised 93% (immediate) and 92% (SoC) students, whereas the vaccine-declined group was 64% students.

**Table 1.** Baseline Demographic Characteristics of Participants in the Primary Efficacy Cohort

Characteristic	Participants, No. (%)^a^			
	Total	Randomized Arms		Vaccine-Declined Group (No Vaccine)
		Immediate	SoC	
Total no. SARS-CoV-2 negative at baseline^b^	1460	600	549	311
Sex assigned at birth				
Male	641 (43.9)	259 (43.2)	255 (46.4)	127 (40.8)
Female	819 (56.1)	341 (56.8)	294 (53.6)	184 (59.2)
Gender identity^c^				
Man	630 (43.2)	255 (42.5)	249 (45.4)	126 (40.5)
Woman	788 (54.0)	327 (54.5)	286 (52.1)	175 (56.3)
Transgender man	6 (0.4)	4 (0.7)	1 (0.2)	1 (0.3)
Transgender woman	3 (0.2)	1 (0.2)	2 (0.4)	0 (0.0)
Gender nonconforming or gender variant	25 (1.7)	8 (1.3)	13 (2.4)	4 (1.3)
Genderqueer	11 (0.8)	7 (1.2)	3 (0.5)	1 (0.3)
Additional identity	8 (0.5)	4 (0.7)	2 (0.4)	2 (0.6)
Prefer not to answer	9 (0.6)	5 (0.8)	0 (0.0)	4 (1.3)
Race^d^				
American Indian or Alaska Native	6 (0.4)	2 (0.3)	3 (0.5)	1 (0.3)
Asian	201 (13.8)	97 (16.2)	93 (16.9)	11 (3.5)
Black or African American	143 (9.8)	47 (7.8)	49 (8.9)	47 (15.1)
Multiple	121 (8.3)	52 (8.7)	43 (7.8)	26 (8.4)
Native Hawaiian or other Pacific Islander	4 (0.3)	2 (0.3)	1 (0.2)	1 (0.3)
Other	40 (2.7)	19 (3.2)	13 (2.4)	8 (2.6)
Prefer not to answer	50 (3.4)	23 (3.8)	13 (2.4)	14 (4.5)
White	895 (61.3)	358 (59.7)	334 (60.8)	203 (65.3)
Ethnicity				
Hispanic or Latino/a	276 (18.9)	106 (17.7)	99 (18.0)	71 (22.8)
Not Hispanic or Latino/a	1175 (80.5)	490 (81.7)	448 (81.6)	237 (76.2)
Age				
18–22 y	1008 (69.0)	449 (74.8)	402 (73.2)	157 (50.5)
23–26 y	334 (22.9)	122 (20.3)	128 (23.3)	84 (27.0)
27–29 y	118 (8.1)	29 (4.8)	19 (3.5)	70 (22.5)
Median (range), y	21 (18, 29)	21 (18, 29)	21 (18, 29)	22 (18, 29)
Residence				
Dormitory or campus housing	186 (12.7)	87 (14.5)	78 (14.2)	21 (6.8)
Fraternity or sorority house	19 (1.3)	9 (1.5)	10 (1.8)	0 (0.0)
Apartment building or condominium	689 (47.2)	285 (47.5)	258 (47.0)	146 (46.9)
Stand-alone house (not a fraternity or sorority)	496 (34.0)	190 (31.7)	178 (32.4)	128 (41.2)
Shelter	3 (0.2)	0 (0.0)	1 (0.2)	2 (0.6)
RV/trailer	10 (0.7)	4 (0.7)	3 (0.5)	3 (1.0)
Staying with friends/”couch surfing”	9 (0.6)	4 (0.7)	3 (0.5)	2 (0.6)
No residence	2 (0.1)	0 (0.0)	0 (0.0)	2 (0.6)
Other	45 (3.1)	21 (3.5)	18 (3.3)	6 (1.9)
No. of people in shared communal space				
0	166 (11.4)	66 (11.0)	63 (11.5)	37 (11.9)
1	412 (28.2)	172 (28.7)	145 (26.4)	95 (30.5)
2	304 (20.8)	118 (19.7)	124 (22.6)	62 (19.9)
>2	573 (39.2)	243 (40.5)	216 (39.3)	114 (36.7)
Student				
Yes	1262 (86)	560 (93)	504 (92)	198 (64)
No	193 (13.2)	39 (6.5)	44 (8.0)	110 (35)

Abbreviations: RV, recreational vehicle; SARS-CoV-2, severe acute respiratory syndrome coronavirus 2; SoC, standard of care.

^a^Data represent no. (%) of participants unless otherwise specified. Owing to missing data, numbers may not add up to the totals for each group and percentages may not sum to 100%.

^b^Polymerase chain reaction negative on the first swab sample and seronegative at the first blood sample collection.

^c^Participants may report >1 gender identity, so numbers may total to >100%.

^d^Participants may report >1 racial category; those who do so are categorized as “multiple.” The denominator for all percentages is the total baseline number SARS-CoV-2 negative.

### Nasal Swab Samples

More than 122 000 swab samples were collected, and approximately 72 500 were tested for SARS-CoV-2. Nasal swab sampling compliance before study completion or outside vaccination was highest in the early weeks of follow-up (week 1 mean rate of completed nasal swab samples, 85% in the immediate and 78% in the SoC arm) and declined over follow-up (week 8 mean rates, 55% and 46%, respectively). Mean weekly rates stayed above 26% (2 swab samples per week) throughout follow-up for all participants (Supplementary Table 2).

### Incidence of and Efficacy Against SARS-CoV-2 Infection

**Figure 2.**
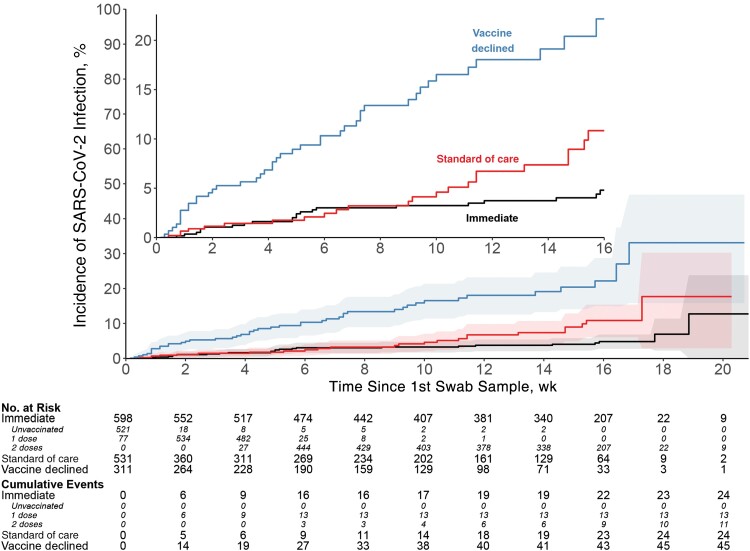
Vaccine efficacy against severe acute respiratory syndrome coronavirus 2 (SARS-CoV-2) infection, displayed as cumulative SARS-CoV-2 incidence by group, among baseline SARS-CoV-2–negative participants.

In the primary efficacy cohort, 48 SARS-CoV-2 infection cases were identified (Figure 2 and Table 2). Incident infection was confirmed in 24 participants in the immediate arm: 11 who received 2 mRNA-1273 doses (incidence, 12.2 per 100 person-years [95% confidence interval [CI], 6.1–21.9]), and 13 who received 1 dose (29.7 per 100 person-years [15.8–50.7]). Incident infection was confirmed in 24 participants in the SoC arm (incidence, 31.8 per 100 person-years [95% CI, 20.3–47.2]). The vaccine efficacy of 2 doses of mRNA-1273 against SARS-CoV-2 infection was 52.6% (95% CI, −14.1% to 80.3%) (Table 2). Cumulative incidence curves for the immediate and SoC arms began to diverge about 9 weeks after enrollment (Figure 2).

**Table 2.** Incidence of Severe Acute Respiratory Syndrome Coronavirus 2 Infection and Adjusted Estimates of Vaccine Efficacy by Group and Vaccine Receipt^a^

Group	Exposure Time Period	Participants Contributing Person-Time, No.	Incident Infections, No.	Person- Years	Incidence Rate Per 100 Person-Years (95% CI)	Adjusted Estimate of Vaccine Efficacy Against SARS-CoV-2 Infection (95% CI), %^b^
Immediate	Before vaccination	521	0	4.8	0.0 (.0–77.3)	NA
	After 1 dose	573	13	43.8	29.7 (15.8–50.7)	−6.8 (−138.3, to 52.2)
	After 2 doses	489	11	89.9	12.2 (6.1–21.9)	52.6 (−14.1 to 80.3)
SoC	Unvaccinated	531	24	75.6	31.8 (20.3–47.2)	NA
Vaccine declined	Unvaccinated	311	45	50.2	89.6 (65.4–119.9)	NA

Abbreviations: CI, confidence interval; NA, not applicable; SARS-CoV-2, severe acute respiratory syndrome coronavirus 2; SoC, standard of care.

^a^This analysis included the subset of participants without SARS-CoV-2 at baseline, from the full analysis set with polymerase chain reaction data; they were censored at outside vaccination.

^b^Cox proportional hazard models of incident SARS­CoV­2 infection event on the calendar time scale, stratified by site and adjusted for factors associated with outside vaccination or SARS-CoV-2 infection absent vaccination: sex, residence, team sport participation at baseline, mask wearing at baseline, and SARS­CoV­2 exposure risk score.

The estimated incidence of infection was lower in the SoC arm with ITT censoring compared to outside vaccination censoring (Supplementary Table 3). It was also lower in both randomized arms and the observational group, including baseline SARS-CoV-2–positive participants, and vaccine efficacy was further attenuated (Supplementary Table 4).

The highest incidence of SARS-CoV-2 infection was in the vaccine-declined group, with 45 incident infections. The incidence among baseline SARS-CoV-2–negative and unvaccinated vaccine-declined participants (89.6 per 100 person-years [95% CI, 65.4–119.9]) was higher than that among similarly characterized participants in the SoC arm (31.8 per 100 person-years [20.3–47.2]) (Figure 2 and Table 2), although much of this was due to effects of geography and calendar time. After adjustment for clinical site and calendar time of follow-up, the estimated risk ratio for incident SARS-CoV-2 infection was attenuated to 1.81 (95% CI, .96–3.41).

Most SARS-CoV-2 infections were attributed to the Delta variant, based on viral sequencing (Supplementary Table 5). There were also no definite cases of SARS-CoV-2 reinfection during the study (eg, no instances of PCR-positive nasal swab samples >90 days apart).

### Incidence of and Efficacy Against Symptomatic COVID-19

Of the 48 participants with SARS-CoV-2 infection in the randomized arms, 24 reported symptoms and were considered to have symptomatic COVID-19–4 after 2 mRNA-1273 doses in the immediate arm (incidence, 4.5 per 100 person-years [95% CI, 1.2–11.4]), 4 after 1 mRNA-1273 dose in the immediate arm (9.1 per 100 person-years [2.5–23.4]), and 14 in the SoC arm (18.5 per 100 person-years [10.1–31.1]) (Supplementary Table 6). The vaccine efficacy of 2 doses of mRNA-1273 against symptomatic COVID-19 was 71.0% (95% CI, −9.5% to 92.3%) (Supplementary Table 7). Efficacy estimates based on ITT censoring and including baseline SARS-CoV-2–positive participants were lower (Supplementary Table 7).

Of the 45 participants with incident infection in the vaccine-declined group, 27 were characterized as having symptomatic COVID-19 (incidence, 53.8 per 100 person-years [95% CI, 35.5–78.3]) (Supplementary Table 6). The proportion of SARS-CoV-2 infections classified as COVID-19 was 27 of 45 (60%) in the vaccine-declined group versus 14 of 24 (58%) in the SoC group (Table 2 and Supplementary Table 6).

In this young adult population, there were no emergency room visits or definite hospitalizations reported in association with any of the symptomatic COVID-19 cases. One hospitalization was reported in a participant who had otherwise not reported symptoms or had a positive PCR nasal swab sample for the previous 9 days.

### Lifestyle Circumstances

**Figure 3.**
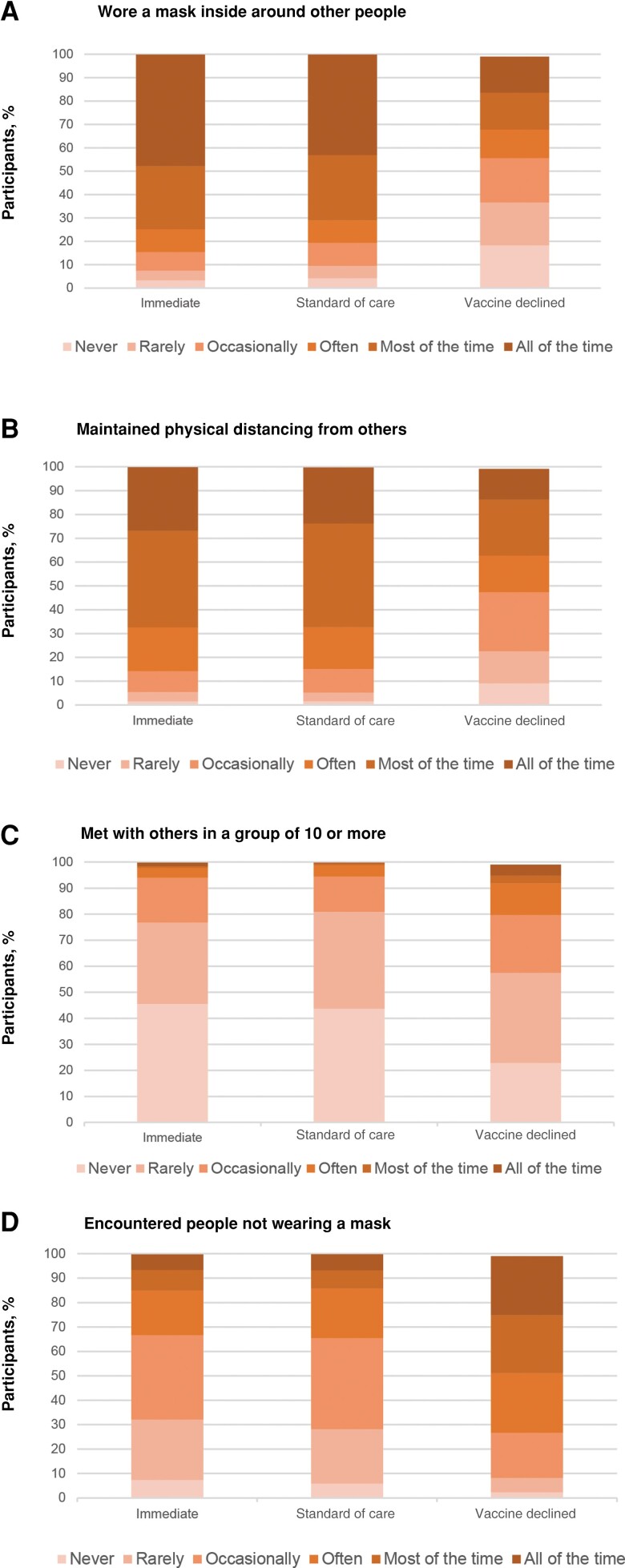
Baseline lifestyle circumstance, displayed as the proportions of participants in each group who reported masking (*A*) physical distancing (*B*) meeting with a group of 10 or more (*C*) or encountering others not wearing a mask (*D*) in the previous 2 weeks.

Lifestyle circumstances differed between the randomized arms and vaccine-declined group, despite the fact that these populations were recruited from the same sites and were thus subject to largely overlapping infection control mandates and policies (Figure 3). For example, mask-wearing and physical distancing behaviors were different: among the vaccine-declined group, 18.3% of participants reported that in the last 2 weeks they “never” wore a mask when inside around other people, compared with 3.8% of the randomized arms, and 24.1% of the vaccine-declined group reported that in the last 2 weeks they encountered people not wearing masks “all of the time,” compared with only 6.5% of the randomized arms. In addition, 4.2% of the vaccine-declined group reported that in the last 2 weeks they met with others “all of the time” in a group of 10 or more, versus 1.5% of those in the immediate arm and 0.2% in the SoC arm. Also, 24.1% of those in the vaccine-declined group reported encountering people not wearing a mask in the last 2 weeks “all of the time,” compared with 6.3% for the immediate arm and 6.6% for the SoC arm.

In addition, 59% of the randomized arms reported being a student but attending school remotely (“on campus class attendance 0 days/week”) versus 21.5% of the vaccine-declined group (Supplementary Table 8). Participants in the vaccine-declined group were almost twice as likely as randomized participants to report working in person or volunteering/studying on campus ≥5 days per week.

## DISCUSSION

Two doses of mRNA-1273 vaccine prevented 52.6% of SARS-CoV-2 infections and 71.0% of symptomatic COVID-19 in young adults who had no evidence of prior SARS-CoV-2 infection in March–December 2021. However, the 95% CIs around these estimates are wide, reflecting uncertainty due to curtailed study enrollment and censoring by off-study vaccination. These estimates of messenger RNA (mRNA)–1273 vaccine efficacy are lower than the 82.0% efficacy against SARS-CoV-2 infection and 93.2% efficacy against symptomatic COVID-19 from the Coronavirus Efficacy (COVE) trial, despite partially overlapping study periods [5]. One possible reason is enhanced detection of SARS-CoV-2 infection in our study through PCR testing of daily nasal swab samples. However, another study that used weekly nose and throat swab samples found that the ChAdOx1 nCoV-19 vaccine was 81.5% effective against symptomatic COVID-19 for non-B.1.1.7 lineages. A potential reason we found lower vaccine efficacy is the predominance of the SARS-CoV-2 Delta variant during our study, which had a larger number of mutations than earlier variants [25] leading to greater escape from existing immunity from vaccines or previous infection [26, 27] compared with the pre-Delta infections that accrued during the COVE and ChAdOx1 nCoV-19 vaccine trials.

The current study was unique for its intensive nasal swabbing protocol to capture all SARS-CoV-2 infections completely and accurately during the study period. Many participants did not report symptoms coinciding with SARS-CoV-2 infection: 64% in the immediate group after 2 doses (7 of 11 infections), 69% in the immediate group after 1 dose (9 of 13 infections), 42% in the SoC arm (10 of 24 infections), and 40% in the vaccine-declined group (18 of 45 infections). These proportions are similar to those reported in the COVE study, which enrolled a broader age range and had limited capture of SARS-CoV-2 infections [28]. The asymptomatic infection proportion among the unvaccinated is also similar to findings of meta-analyses and systematic reviews, although there was high heterogeneity across studies. Our study provides solid estimates for SARS-CoV-2 infection in a closed cohort of young adults with frequent surveillance for infection, including asymptomatic infection.

The incidence of SARS-CoV-2 infection among unvaccinated participants without SARS-CoV-2 infection at baseline was 1.8-fold higher in the vaccine-declined group compared with the SoC arm, even after controlling for differences in geography and calendar time. This increased incidence was likely due to differing lifestyle circumstances, such as the rates of encountering people wearing masks indoors, physical distancing, and avoidance of large gatherings. The higher incidence in the vaccine-declined group may also represent structural inequities across race/ethnicity and/or socioeconomic status leading to increased SARS-CoV-2 exposure. There was little observed difference in swab sample completion between the groups, making varying adherence to study procedures an unlikely explanation for divergent incidence rates (Supplementary Table 1). These findings support vaccination as one of many factors that influence SARS-CoV-2 incidence; additional studies should focus on understanding transmission dynamics in diverse settings.

The current study has several limitations. The trial included a short duration of follow-up and was open label. Adhering to daily swabbing was difficult in this young cohort, though all study groups maintained nasal swabbing at least twice per week on average throughout the study (Supplementary Table 1). The fast-changing pandemic was also a challenge [22, 29]. Participants were encouraged to follow local standards of care for COVID-19 prevention, and consequently rates of “off-study” vaccination in the SoC arm rose to 58% by the end of study follow-up, contributing to uncertainty in our efficacy estimates. Our study population was limited to healthy young adults by design, limiting generalizability to older individuals or those with serious illnesses. The data were limited to the mRNA-1273 vaccine and to variants circulating in March to September 2021 (eg, Alpha and Delta) and may not inform our understanding of other vaccines or other subsequent variants, such as Omicron. Thus, continued research is needed to understand the efficacy of other vaccines and vaccine efficacy against additional variants. Finally, differentiating between SARS-CoV-2 infection and symptomatic COVID-19 depended on defining and capturing symptoms, which we did through self-report. Missing symptom data may have led to an underestimated incidence of symptomatic COVID-19. Despite these limitations, we were able to assess the efficacy of the mRNA-1273 vaccine against infection and COVID-19 for 18–29-year-olds and estimate the fraction of SARS-CoV-2 infections that were asymptomatic.

Strengths of this study include its randomized, prospective controlled design with daily nasal swab testing, which contrasts with much of the literature on vaccine protection against SARS-CoV-2 infection, which is often observational, retrospective, and reliant on infrequent SARS-CoV-2 infection testing [11, 16–18]. Another strength was our ability to enroll 476 volunteers not interested in receiving a vaccination; adherence to study procedures in this vaccine-declined group was no different than in the SoC arm.

In conclusion, we provide confirmatory data that mRNA COVID-19 vaccination reduced the incidence of SARS-CoV-2 infection from March to September 2021. However, vaccination is only one of many factors that influence infection risk, and additional studies should focus on understanding transmission dynamics across a range of diverse settings and populations.

## Supplementary Data

Supplementary materials are available at *Open Forum Infectious Diseases* online. Consisting of data provided by the authors to benefit the reader, the posted materials are not copyedited and are the sole responsibility of the authors, so questions or comments should be addressed to the corresponding author.

## Supplementary Material

ofad511_Supplementary_Data
