# Supplementary material for: Efficacy of Messenger RNA–1273 Against Severe Acute Respiratory Syndrome Coronavirus 2 Acquisition in Young Adults From March to December 2021
Source: Open Forum Infect Dis. 2023 Nov 2;10(11):ofad511. doi: 10.1093/ofid/ofad511 (PMC10655942; doi:10.1093/ofid/ofad511)
Supplement: ofad511_Supplementary_Data [file ofad511_supplementary_data.zip › 3006 Protocols 1 through 4.pdf]

COVID-19
Prevention Network

PROTOCOL

CoVPN 3006

A randomized controlled study to assess SARS CoV-2 infection, viral shedding, and subsequent potential transmission in university students immunized with Moderna COVID-19 Vaccine

DOCUMENT ID 38822

Non-IND Study

CLINICAL TRIAL SPONSORED BY

National Institute of Allergy and Infectious Diseases (NIAID)
National Institutes of Health (NIH)
Department of Health and Human Services (DHHS)
Bethesda, Maryland, USA

February 17, 2021
Final
CoVPN 3006
Version 1.0

Contents

1 Overview 5
1.1 Protocol Team 8
2 Background 10
2.1 Rationale for trial concept 10
2.2 Measures of vaccine efficacy 11
2.3 Importance of understanding vaccine effects on infectiousness and transmissibility 12
2.4 Limitations of the current Phase 3 trials for addressing unanswered questions 15
2.5 Burden of SARS-CoV-2 infection and COVID-19 disease on university campuses 16
2.6 Age and Asymptomatic Infection 17
2.7 Evidence for viral load as a predictor of infectiousness 18
2.8 Evidence for viral load as a marker of COVID-19 disease severity and clinical course 19
2.9 Methods for capturing transmission 19
2.10 Moderna COVID-19 Vaccine 21
2.11 Trial design rationale 21
3 Objectives and endpoints 24
4 Ethical considerations 27
5 IRB/EC review considerations 29
5.1 Minimized risks to participants 29
5.2 Reasonable risk/benefit balance 29
5.3 Equitable participant selection 29
5.4 Appropriate informed consent 30
5.5 Adequate safety monitoring 30
5.6 Protect privacy/confidentiality 30
6 Statistical considerations 32
6.1 Accrual restrictions 33
6.2 Accrual and sample size calculations 34
6.3 Role of the Independent Data Monitoring Committee (IDMC) 38
6.4 Randomization 38
6.5 Statistical analyses 38
7 Selection and withdrawal of participants 47
7.1 Inclusion criteria for Main Cohort 47
7.2 Exclusion criteria for Main Cohort 48
7.3 Inclusion criteria for Prospective Close Contact (PCC) cohort 49
7.4 Inclusion criteria for Case-ascertained Close Contact (CACC) cohort 49
8 Study product 50
8.1 Vaccine regimen 50
8.2 Storage, handling, preparation, and administration 50
8.3 Acquisition of Moderna COVID-19 Vaccine 50

9 Clinical procedures 51
9.1 Informed consent 51
9.2 Pre-enrollment procedures for Main Cohort, Immediate and Delayed Vaccination Groups 52
9.3 Enrollment and vaccination visits for Main Cohort, Immediate Vaccination Group 53
9.4 Follow-up procedures for Main Cohort, Immediate Vaccination Group 53
9.5 Enrollment (visit 2) and visits 3-5 for Main Cohort, Delayed Vaccination Group 54
9.6 Vaccination visits for Main Cohort, Delayed Vaccination Group 54
9.7 Procedures for Main Cohort participant diagnosed with a SARS-CoV-2 infection during the study 54
9.8 Enrollment and follow-up procedures for Prospective Close Contact (PCC) cohort 55
9.9 Enrollment and Follow-up procedures for Case-Ascertained Close Contact (CACC) cohort 56
9.10 Visit windows and missed visits 57
9.11 Early termination visit 57
9.12 Pregnancy for Main Cohort 57
9.13 SARS-CoV-2 monitoring by universities for Main Cohort 57
9.14 COVID-19 symptom surveillance for Main Cohort 58
9.15 Use of electronic Diary (eDiary) for Clinical Outcomes Assessments 58
10 Laboratory 60
10.1 CRS laboratory procedures 60
10.2 Total blood volume 60
10.3 Endpoint assays 60
10.4 Lab assay portfolio 61
10.5 Exploratory studies 61
10.6 Specimen storage and other use of specimens 61
10.7 Biohazard containment 62
11 Safety Reporting 63
11.1 FDA/CDC Vaccine Adverse Event Reporting System (VAERS) 63
11.2 Reporting safety events to v-safe 64
12 Protocol conduct 65
12.1 Study termination 65
12.2 Emergency communication with study participants 66
13 Version history 67
14 Document references (other than literature citations) 68
15 Acronyms and abbreviations 70
16 Literature cited 72
Appendix A Sample informed consent form for Main cohort 77
Appendix B Sample consent form for Prospective Close Contact (PCC) cohort 93
Appendix C Sample consent form for Case-Ascertained Close Contact (CACC) cohort 103

Appendix D Procedures for Main Cohort, Immediate Vaccination Group 113
Appendix E Procedures for Main Cohort, Delayed Vaccination Group 115
Visit: 115
Appendix F Procedures for Prospective Close Contact (PCC) cohort 117
Appendix G Procedures for Case-Ascertained Close Contact Cohort............................ 118
Appendix H Visit windows... 119
Appendix I Protocol Signature Page 120

1 Overview

Title

A randomized controlled study to assess SARS CoV-2 infection, viral shedding, and subsequent potential transmission in university students immunized with Moderna COVID-19 Vaccine

Primary objective(s)

Primary objective 1:

To evaluate the efficacy of Moderna COVID-19 Vaccine against SARS-CoV-2 infection (ie, to evaluate vaccine efficacy against infection or VE_S)

Primary objective 2:

To evaluate the effect of Moderna COVID-19 Vaccine on peak nasal viral load as a measure of infection and a proxy of infectiousness

Study product and route of administration

Moderna COVID-19 Vaccine: a lipid nanoparticle (LNP) dispersion of a messenger ribonucleic acid (mRNA) encoding the prefusion stabilized S protein of SARS-CoV-2 formulated in LNPs composed of 4 lipids (1 proprietary and 3 commercially available). The Moderna COVID-19 Vaccine, also known as mRNA-1273, is a suspension for intramuscular injection administered as a series of two doses (0.5 mL each) 1 month apart.

This vaccine has been issued an Emergency Use Authorization (EUA) by the US Food and Drug Administration (FDA) for active immunization to prevent COVID-19 in individuals 18 years and older.

Table 1-1 Schema for the Main Cohort

		Injection schedule in months			
Group	N	0	1	4	5
Immediate Vaccination	6000	Moderna COVID-19 Vaccine	Moderna COVID-19 Vaccine	-	-
Delayed Vaccination	6000	-	-	Moderna COVID-19 Vaccine	Moderna COVID-19 Vaccine
Total	12,000				

Participants

Main Cohort (main study participants)

Approximately 12,000 healthy university student volunteers aged 18 through 26 years who will be followed for 5 months after enrollment; 6,000 randomized to immediate vaccination, 6,000 randomized to delayed vaccination

Prospective Close Contact (PCC) Cohort

Up to approximately 24,000 volunteers subject to university SARS-CoV-2 testing who are in frequent close physical proximity with main study participants

Case-Ascertained Close Contact (CACC) Cohort

Approximately 3 contacts per incident case. Actual number dependent on incidence rate. Up to approximately 1500 volunteers aged 18 years and older who have been in close contact with a SARS-CoV-2 positive case from the Main Cohort

Design

Multicenter, randomized, controlled, open-label trial

Duration per participant

Up to 5 months per participant for the Main Cohort and for the Prospective Close Contact Cohort. 1 month per participant in Case-Ascertained Close Contact Cohort.

Estimated total study duration

7 months (includes enrollment and follow-up).

Core operations

CoVPN Vaccine leadership Group/Core Operations Center, Fred Hutchinson Cancer Research Center (Fred Hutch) (Seattle, Washington, USA)

PPD, Inc. (Wilmington, North Carolina, USA)

Statistical and Data Management Center (SDMC)

Statistical Center for HIV/AIDS Research and Prevention (SCHARP), Fred Hutch (Seattle, Washington, USA)

Laboratory Center (LC)

Endpoint assay laboratories

- University of Washington Virology Laboratory (UW-VL) (Seattle, Washington, USA)
- University of Washington Virology Specialty Laboratory (UW-VSL) (Seattle, Washington, USA)
- Duke University Medical Center (Durham, North Carolina, USA)

Study sites

- University clinical research sites (CRSs) across the US that have large-scale asymptomatic SARS-CoV-2 testing programs to be specified in the Site Announcement Memo

Study monitoring

Independent Data Monitoring Committee (IDMC)

1.1 Protocol Team

Protocol leadership

Co-chairs	Kathryn Stephenson<br>Harvard University School of Medicine<br>Beth Israel Deaconess Medical Center<br>617-735-4556<br>kstephen@bidmc.harvard.edu<br><br>Audrey Pettifor<br>Department of Epidemiology<br>Gillings School of Global Public Health<br>University of North Carolina<br>919-966-7439<br>apettif@email.unc.edu	*Protocol Lead Advisors*	Larry Corey<br>CoVPN LOC, Fred Hutch<br>206-667-6770<br>lcorey@fredhutch.org<br><br>Myron Cohen<br>University of North Carolina<br>919-966-2537<br>myron_cohen@med.unc.edu<br><br>James Kublin<br>CoVPN LOC, Fred Hutch<br>206-667-1970<br>jkublin@fredhutch.org
Statisticians	Holly Janes<br>CoVPN SDMC, Fred Hutch<br>206-667-6353<br>hjanes@fredhutch.org<br><br>Elizabeth Brown<br>CoVPN SDMC, Fred Hutch<br>206-667-1731<br>erbrown@fredhutch.org	*Laboratory lead*	John Hural<br>CoVPN Laboratory Program<br>206-667-1683<br>jhural@fredhutch.org
Protocol Team leader	Nicole Grunenberg<br>CoVPN LOC, Fred Hutch<br>206-667-2043<br>ngrunenb@fredhutch.org	*DAIDS Medical Officer*	Catherine Yen<br>DAIDS, NIAID<br>240-292-4783<br>catherine.yen@nih.gov

Other contributors to the original protocol

Core medical monitor	Nicole Grunenberg<br>CoVPN LOC, Fred Hutch	*CoVPN Physician consultant*	Nzeera Ketter<br>CoVPN LOC, Fred Hutch
Laboratory consultants	Alexander Greninger<br>Robert Coombs<br>University of Washington Medical Center (UWMC)<br>Keith Jerome<br>Fred Hutch, UWMC	*Clinical safety specialists*	Megan Jones<br>Maija Andersen<br>CoVPN LOC, Fred Hutch
Laboratory center representatives	Nicole Espy<br>Lisa Sanders<br>Jen Hanke<br>CoVPN Laboratory Center, Fred Hutch	*Clinical trials manager*	Julie Hunt<br>CoVPN LOC, Fred Hutch
Regulatory affairs associate	Megan Brandon<br>CoVPN LOC, Fred Hutch	*Clinical data managers*	Melissa Cummings<br>Vicky Kim<br>CoVPN SDMC, Fred Hutch

Community Advisory Board (CAB) members	John Isaac<br>Boston University	*SDMC Associate Director of Operations*	Jessica Andriesen<br>CoVPN SDMC, Fred Hutch
Statistical research associate	Hua Zheng<br>CoVPN SDMC, Fred Hutch	*SDMC Associate director of lab science*	April Randhawa<br>CoVPN SDMC, Fred Hutch
Community engagement unit representative	Francisco E Rentas<br>CoVPN LOC, Fred Hutch	*Data Management Director*	Kristine Donaty<br>CoVPN SDMC, Fred Hutch
		Protocol development managers	Kajari Mondal<br>Meg Trahey<br>CoVPN LOC, Fred Hutch

2 Background

2.1 Rationale for trial concept

The United States has the most infections and deaths from SARS-CoV-2 (the virus that causes Coronavirus disease 2019 [COVID-19]) of any nation in the world (1). Fortunately, there is now hope that the large burden of COVID-19-related illness may lessen in 2021 due to the discovery that two candidate COVID-19 vaccines, including the Moderna COVID-19 vaccine, can prevent symptomatic COVID-19 and severe disease in adults (2, 3). Ongoing Phase 3 trials are not designed to estimate a vaccine's efficacy against all infections, with a critical gap being asymptomatic infections. The current trials are also not designed to evaluate vaccine effects on infectiousness as measured by viral shedding or on onward transmission of infection. For example, at an individual level, it is currently unknown if vaccinated individuals should continue to wear masks or social distance, or if they should be quarantined if exposed to COVID-19. At a policy level, it is unknown if vaccination should be required for certain settings, such as public transit including air travel, places of employment, or school. These questions are especially important given that SARS-CoV-2 is highly infectious compared to other respiratory illnesses such as the flu. Recent data also demonstrate that even individuals with asymptomatic COVID-19 can have high titers of nasal viral shedding and thus may be highly infectious. Furthermore, many individuals with asymptomatic infection develop low level and transient antibodies against SARS-CoV-2 (4), suggesting that trials relying on seroconversion as a marker of infection may miss infections.

While there are many populations at risk for SARS-CoV-2 infection, young people are particularly at risk of acquiring and transmitting SARS-CoV-2. Between August and September 2020, COVID-19 cases among young people aged 18-22 years increased 55% nationally (5) and during June-August 2020, young people aged 20-29 years had the highest incidence of disease in the US, accounting for >20% of all confirmed cases (6). Further, increases in SARS-CoV-2 infection among younger adults in the Southern US preceded increases among older adults by 4-15 days, which confirms the importance of younger adults spreading infection to older, more vulnerable populations (5). At the same time, individuals aged 18-29 account for a tiny percentage (0.5%) of all deaths due to COVID-19 and are very unlikely to develop severe disease (7). These reasons plus the congregate and social living situations of university students and that many universities are regularly testing their student body make them an excellent population to examine. The proposed study is designed to evaluate comprehensively the vaccine efficacy profile of the Moderna COVID-19 vaccine, including its effect on acquisition of infection including asymptomatic infection, on disease and severity of disease, on measures of infectiousness, and transmission of infection.

This study is a 2-arm randomized, open-label trial in university students aged 18 through 26 years that will compare those who receive the Moderna COVID-19 Vaccine upon enrollment (immediate vaccination arm) to those who receive the vaccine 4 months later (delayed vaccination arm). **The study will capture all incident SARS-CoV-2 infections among study participants in real time, by partnering with universities who have implemented intensive testing for SARS-CoV-2 among their students.** The university testing systems

will enable infections among study participants to be captured in real time. In addition, study participants will be asked to collect daily nasal swabs, to enable retrospective ascertainment of the timing of acquisition and to capture the full viral load trajectory for incident infections. To capture secondary transmission events, very close contacts of study participants will be enrolled and surveilled by the university for incident infection over the duration of the study ('prospective contacts'); in addition, diagnosis of infection among a study participant will trigger recruitment of additional participant contacts ('case-ascertained close contacts'). When a study participant tests positive based on university testing, both prospective contacts and case-ascertained close contacts will be asked to collect daily nasal swabs for 14 days to detect incident infection and also will have blood collected for serology. The study will take place over the course of the 2021 spring and summer school terms. At the end of the trial, all participants in the delayed arm will be offered vaccine. The goal of the study is to report results prior to fall 2021 to inform school policy prior to when the new year begins for most educational institutions.

2.2 Measures of vaccine efficacy

Ongoing Phase 3 COVID-19 vaccine trials are designed with the primary objective to evaluate vaccine efficacy against COVID-19 disease, written VE_D, and defined as the multiplicative reduction in risk of disease in the vaccine vs. placebo groups. COVID-19 disease is defined by a protocol-specified set of signs and symptoms of disease coincident with PCR-confirmed SARS-CoV-2 infection. While the reduction in risk of disease is arguably the most relevant vaccine effect to the individual participant, understanding the population effect of a vaccine requires considering the other effects of the vaccine (see Table 2-1) (8). The most fundamental effect of a vaccine is to reduce acquisition of infection, sometimes referred to as 'vaccine efficacy against susceptibility', written VE_S, and measured by the multiplicative reduction in PCR-confirmed infection in the vaccine vs. placebo group. A vaccine that reduces acquisition of infection will provide benefit to the individual (i.e. *direct* effects), as well as benefit to the population by reducing the number of infected and therefore potentially infectious individuals who may expose and infect unvaccinated individuals in the population (i.e. *indirect* effects). Modeling studies suggest that COVID-19 vaccines that reduce acquisition are predicted to have considerably greater population impact, not only reducing disease, hospitalizations, and mortality, but reducing transmission of infection and thereby magnifying their effects (9, 10).

An additional potential indirect effect of a vaccine is its effect on reducing secondary transmission of infection, written VE_I and defined as the multiplicative reduction in the rate of secondary transmission among vaccine vs. placebo individuals who become SARS-CoV-2 infected. A vaccine might reduce transmission through reducing the magnitude and/or duration of viral shedding, by reducing symptoms associated with transmission such as coughing, or by selectively blocking infection with less transmissible viral strains. Evaluating the effect of a COVID-19 vaccine on transmissibility is critical because a vaccine with high efficacy against COVID-19 disease but low efficacy against SARS-CoV-2 infection would necessarily yield a net increase in the number of asymptomatic infections. If individuals who become asymptomatically infected despite having received the vaccine are still infectious,

and given that asymptomatic individuals are less likely to be diagnosed and self-isolate, such a vaccine has the potential to increase transmission and prolong the pandemic (2, 3).

As we prepare this study, we recognize there are other approaches to measuring population level benefit (eg, vaccine effect on infection and transmissibility). These approaches include: observational studies, such as those being conducted in Israel to examine population benefit of the vaccine and those planned by the CDC to examine transmission from vaccinated individuals to household contacts in longitudinal cohorts; and ancillary studies with frequent swabbing on a subset of participants in Phase 3 studies. However, none of these studies will allow for the rigorous capture, through daily nasal swabbing, of the early dynamics of viral replication and the capture of transmission events during the acute period of infection in a randomized trial.

All of these vaccine salutatory effects may depend on a variety of individual characteristics such as age, prior infection with SARS-CoV-2; on virological characteristics; and in the case of VE_I which conditions on infection, may depend on clinical characteristics such as symptomatology.

As will be discussed below in detail, the ongoing Phase 3 trials evaluate VE_D and partially identify VE_S, but do not allow direct estimation of VE_I. In contrast, the proposed study will collect data to enable evaluation of all three vaccine efficacy parameters in the university student population. Importantly, the use of an open-label delayed vaccination arm as opposed to a blinded placebo arm implies that the various effects evaluated will capture not only biological vaccine effects, but also potential behavioral effects associated with vaccination, including potential behavioral disinhibition. Behavioral effects most plausibly influence exposure and therefore infection, but knowledge of vaccine receipt is viewed as less likely to influence disease, viral load, and secondary transmission.

Table 2-1 Population measures of vaccine efficacy that will be evaluated using the proposed study, where a delayed vaccination arm will be used instead of placebo

Efficacy measure	Definition
Vaccine efficacy against SARS-CoV-2 infection (VE_S)	The multiplicative reduction in SARS-CoV-2 infection as measured by PCR of nasal swabs, vaccine vs. placebo
Vaccine efficacy against COVID-19 disease (VE_D)	The multiplicative reduction in symptomatic COVID-19*, vaccine vs. placebo
Vaccine efficacy against viral replication (VE_{VL})	The reduction in SARS-CoV-2 viral burden as measured by PCR of nasal swabs, vaccine vs. placebo
Vaccine efficacy against transmission (VE_I)	The multiplicative reduction in rate of SARS-CoV-2 transmission from infected individuals on the vaccine arm relative to the placebo arm

*Defined by a protocol-specific set of signs and symptoms defined in Section 3.

2.3 Importance of understanding vaccine effects on infectiousness and transmissibility

The most effective way for a vaccine to prevent onward transmission of infection is to prevent acquisition of infection. Vaccine efficacy against infection, VE_s, is a measure of this effect (8). However, a vaccine that does not completely block acquisition of infection may

still reduce onward transmission among individuals who become infected despite having received the vaccine, as measured by VE_I (8, 11). As discussed above, understanding the effect of a COVID-19 vaccine on transmission is critical because a vaccine with high efficacy against COVID-19 disease but low efficacy against SARS-CoV-2 infection would necessarily yield a net increase in the number of asymptomatic infections. If individuals who become asymptomatically infected despite having received the vaccine are still infectious, and given that asymptomatic individuals are less likely to be diagnosed and self-isolate, such a vaccine has the potential to increase transmission and prolong the pandemic (2, 3).

If a COVID-19 vaccine did reduce infectiousness, mathematical modeling suggests that much greater population impact could be achieved. Deterministic compartmental models fit to data from King County, Washington and envisioning vaccine rollout starting December 1, 2020 to 18% of the adult population proportionally across age groups, suggest that while a vaccine that reduces COVID-19 disease by 90% and SARS-CoV-2 infection by 10% reduces total infections in 2021 by 18.1% and deaths by 27.5%, if the vaccine additionally reduces infectiousness by 50% the impact on infections would more than double (to 49.3% reduction) and deaths would be cut in half (to 50.9% reduction) (Swan and Dimitrov et al, in preparation; Figure 2-1).

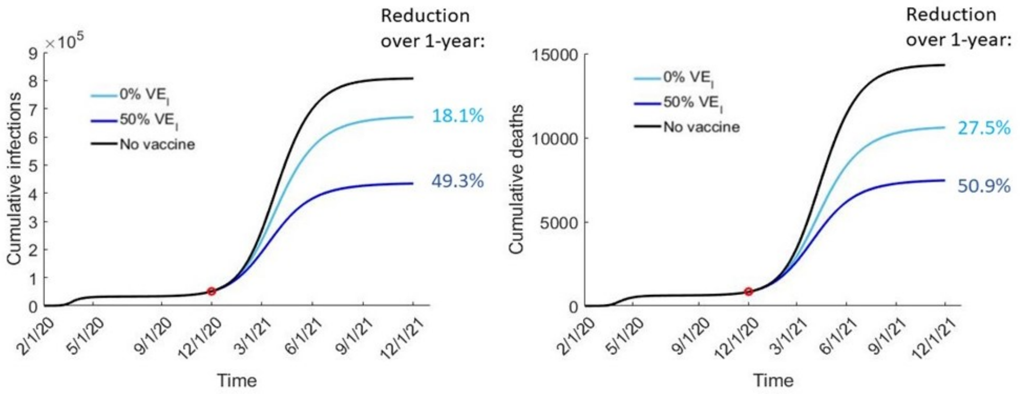

Figure 2-1 COVID-19 vaccine that reduces infectiousness by 50% has greater impact on infections and deaths. Deterministic compartmental model calibrated to King County, WA stratified by age, SARS-CoV-2 infection status, COVID-19 treatment status and vaccination. 800,000 doses vaccine (for 18% of population) starting Dec 1, 2020, allocated proportionally across age groups. Absent vaccination, 20% of infections are asymptomatic; asymptomatic infections are 28% less infectious than symptomatic infections, but more transmissible due to lower rates of diagnosis and quarantine. Both vaccines reduce COVID-19 disease by 90% and SARS-CoV-2 infection by 10%. While one vaccine (light blue) does not reduce infectiousness among individuals who become infected, the other vaccine (dark blue) reduces infectiousness by 50%. As a result, it achieves a much greater reduction in both infections and deaths. (Swan, Dimitrov et al, in preparation).

In the context of limited vaccine supply, mathematical modeling also suggests that optimal allocation of vaccine depends on how well a vaccine reduces infectiousness. Take, for instance, a deterministic compartmental model calibrated to Washington State that uses the following assumptions: that, absent vaccination, 40% of infections are asymptomatic, asymptomatic infections confer equal immunity and are equally infectious, and that there is sufficient vaccine supply for 30% of the population. For a vaccine that reduces COVID-19 disease by 90% but has no effect on infection or infectiousness, the limited supply of vaccine

should be allocated to older adults who have a high burden of disease in order to minimize deaths and peak hospitalizations (Figure 2-2). However, if the vaccine also reduces infectiousness by 60%, minimizing peak hospitalizations is achieved by allocating limited vaccine to the young, while minimizing deaths still requires vaccinating older adults. These results again highlight the critical importance of understanding vaccine effects on acquisition and infectiousness in order to make policy decisions.

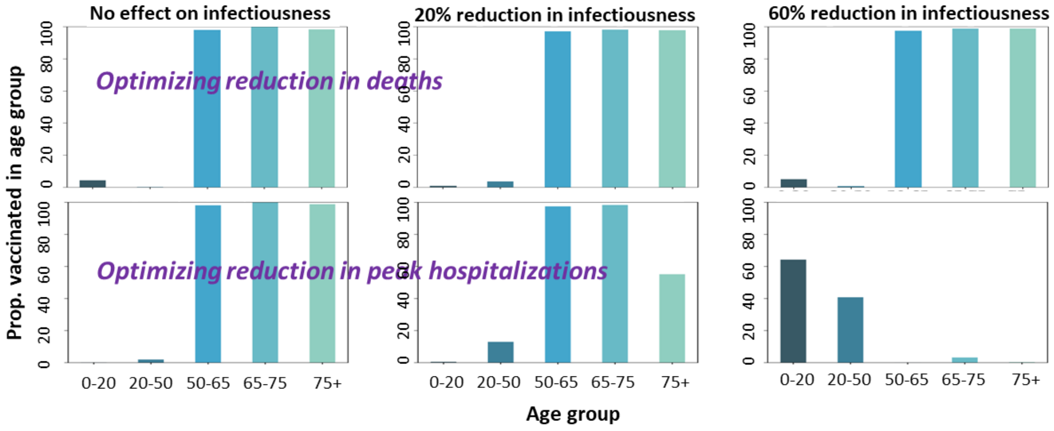

Figure 2-2 Deterministic compartmental model calibrated to Washington State (7.6M people), reflecting US demographics. Model assumes 4.56M doses vaccine (for 30% of population) assuming 10% of the population is previously infected and immune and there are 1000 current infections. Absent vaccination, 40% of infections are asymptomatic; asymptomatic infections confer equal immunity (for at least 6 mo. duration) and are equally infectious. (Matrajt et al, in preparation)

Non-human primate (NHP) data also suggests that elimination of viral shedding by a COVID-19 vaccine is not guaranteed. Table 2-2 shows the comparison of NHP data between candidate COVID-19 vaccines in current Phase 3 trials. While most of the vaccines were shown to prevent viremia within the lower respiratory tract (bronchoalveolar lavage fluid), and thus improve markers of disease severity, three out of the four platforms tested did not eradicate nasal viral shedding in all cases.

Table 2-2 Comparison of NHP data between Janssen Ad26, Moderna RNA, AstraZeneca ChimpAdOx1 and Novavax COVID-19 vaccines

	**Ad26 One dose 1 x10^{11}**	**mRNA (4 weeks post-dose 2)**	**AstraZeneca/Oxford 2.5x10^{10} 0, 4 weeks**	**Novavax 2 doses (0,21) 5 mcg, 50 mcg**
**Pseudovirus neutralization (GMT)**	408	1,862	5 – 40 boost 10 - 160	No
**Live virus assay**	113	3,481		16,026
**Spike protein**	15,000 (not shown)	36,186 (AUC)		335,017
**Lung BAL protection**	Complete	Complete at day 7	75% both prime and boost	Complete
**Nasal protection**	5/6 (83%)	7/8	0%	Complete (12/12)
**T-cell immune response**	Some CD8	No CD8+ T cells	Not reported	CD4 and CD8

Finally, not understanding relative infectivity is a huge gap in translating vaccine use to public health messaging. The demonstration of reduced infectivity may reduce vaccine hesitancy. Public health, medical personnel and organizations may embrace vaccination, as well as users, if the vaccine both provides individual as well as family/community benefit. For individuals, knowing that a vaccine reduces infectivity to household and personal contacts is often a prime motivator for vaccination. Data from this trial can facilitate this messaging as well as help decision-making among individuals about interactions with household members and attending work and school.

2.4 Limitations of the current Phase 3 trials for addressing unanswered questions

Currently, none of the current Phase 3 vaccine efficacy trials will enable robust estimation of vaccine effects on infectiousness. The primary objectives for all trials are to evaluate vaccine efficacy (VE) against COVID-19 disease. Secondary objectives include evaluating VE against seroconversion using infrequent serology (every 1-6 months over 1 year, with more frequent sampling over the first 3 months), but this will not fully capture all infections including those that are asymptomatic. Estimates based on acute infection cohorts (12) suggest that 12-50% of asymptomatic infections might be missed using this sampling, leading to an unknown magnitude and direction of bias in the estimate of VE_S. Data from Long et al. found that 40% of individuals with asymptomatic RT-PCR confirmed SARS-CoV-2 infections were IgG negative at 8 weeks after the acute phase of infection (Figure 2-3) (4). Further, it has been observed that asymptomatic cases have lower IgG titers across all antigens (including NP) (Figure 2-4) (13). In current trials, infectiousness is not captured robustly among those who become infected; viral load (VL) is only measured for those who are symptomatic and only after the onset of symptoms. Data from the Moderna trial where nasal swabs were collected at the time of vaccine administration on days 0 and 28 among all

participants suggest that the vaccine may reduce the level of nasal carriage of virus (14). However, one time point does not robustly capture the full viral dynamics of infection, in particular, asymptomatic infections. The data are suggestive that the vaccine may reduce viral carriage (15). The level of viral shedding prior to the onset of symptoms, when most transmission occurs (16), will not be captured for any infected participants. Importantly, no data in current trials are collected on onward transmission of infection and therefore VE_I cannot be evaluated directly.

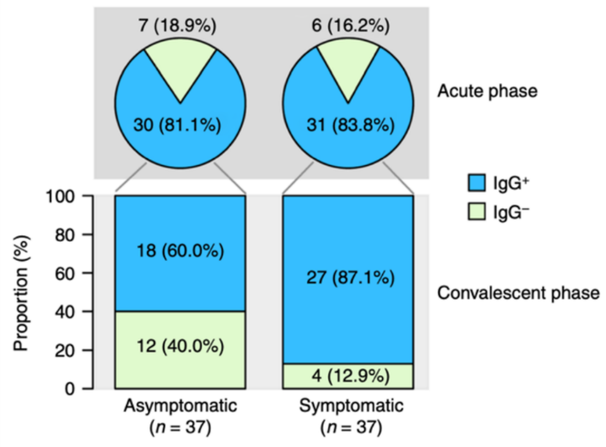

Figure 2-3 40% of asymptomatic individuals were IgG negative by 8 weeks post-acute infection (Long et al.(4).

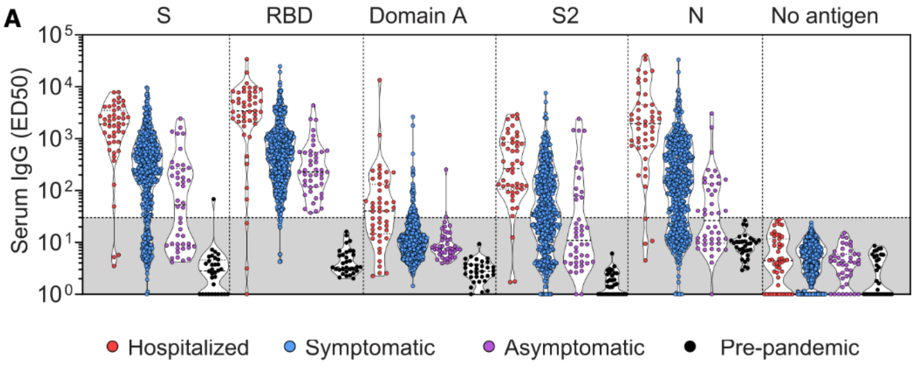

Figure 2-4 Asymptomatic Patients with COVID-19 have Lower IgG Titers Across Multiple Antigens Among Italian Medical Staff N>600 (13).

2.5 Burden of SARS-CoV-2 infection and COVID-19 disease on university campuses

The study will be conducted among students enrolled in universities because these are ideal locations for studying SARS-CoV-2 transmission clusters. Large numbers of cases of COVID-19 have been reported on numerous campuses throughout the US as colleges and universities re-opened in fall 2020. More than 397,000 cases were counted at 1,800+ colleges

and universities in fall 2020 (17, 18). Over the time period July-December 11th, 2020, more than 85 colleges and Universities had more than 1,000 cases. Among these 49 campuses the cumulative attack rate from July to October was on average 6.2%. These estimates are somewhat uncertain given the fact that the number of reported cases for some schools includes faculty, staff and graduate students; however, the number of such individuals is very small in comparison to the number of undergraduates.

Among 15 Association of American Universities (AAU) universities, and using data from the start of semester through September 18, 2020, there was an estimated 3.7% 1-month attack rate. The attack rate appeared to decline over time; it was 1.7% over September 19 to October 20, 2020. Attack rates may be underestimated because the denominator is assumed to be all undergraduates, whereas some schools had only a subset of students on campus. These statistics suggest that a conservative assumption of SARS-CoV-2 incidence among US university students for Fall semester 2020 is 6%. A conservative incidence assumption for Spring and Summer 2021semesters, anticipating potential improvements in testing, contact tracing, social distancing measures, as well as vaccine rollout to priority populations outside the universities, is therefore 3-6%.

The outbreaks of SARS-CoV-2 infection at college campuses have been reported to be associated with students residing in high density housing, socializing off campus, and not following social distancing guidelines (19). In the US, it is estimated that 45 percent of young adults aged 18-22 years were enrolled in colleges and universities in 2019 (16). As such, much of the increase in cases this summer and fall are attributed to college students. The college campuses are associated with a high rate of SARS-CoV-2 infection and resulting asymptomatic carriage due to huge impulse to socialize as well as no fear of severe disease among the young students.

2.6 Age and Asymptomatic Infection

For this study, participants will be enrolled who are 18 to 26 years of age. This age range is chosen because of the high incidence of asymptomatic infection in this population. A considerable body of data suggest that SARS-CoV-2 can be spread by individuals with and without symptoms (20-23). VL data among individuals with acute SARS-CoV-2 infection also indicate that peak VL usually occurs before disease onset (22, 24). This suggests that there is a substantial transmission potential before symptom onset. According to the latest Centers for Disease Control and Prevention (CDC) estimate 40% of SARS-CoV-2 infections are asymptomatic (25). Available data indicate that younger individuals appear more likely to remain asymptomatic following infection than older individuals (26, 27). A recent study done in the UK showed about 80% of children between 10-19 years of age remain asymptomatic (28). As infected young students are less likely to show clinical symptoms, the number of clinical cases reported among young populations would be lower, but the asymptomatic young students could still be capable of transmitting the virus to others (29).

2.7 Evidence for viral load as a predictor of infectiousness

Mathematical modelling in human immunodeficiency virus (HIV) and herpesviruses and now COVID-19 suggests a significant percentage of transmission occurs from high peak VL which in all these diseases is during the asymptomatic period. Models by Josh Schiffer and Ashish Goyal suggest that a person has a 50% chance of infection with exposure to 10^7 SARS-CoV-2 RNA and <5% chance of infection with exposure to 10^5 SARS-CoV-2 RNA (Figure 2-5) (21). The model by Goyal et al (21) suggests that vaccine reduction in peak VL may reduce infectiousness. A 0.6 log reduction corresponds to ~50% VE_I and a 2.5 log reduction to ~90% VE_I (Figure 2-6). This suggests collecting nasal swabs every other day would be required to measure the vaccine effect on peak VL and area under the VL curve. Asymptomatic and pre-symptomatic cases of SARS-CoV-2 appear to play an important role in the onward transmission of the virus.

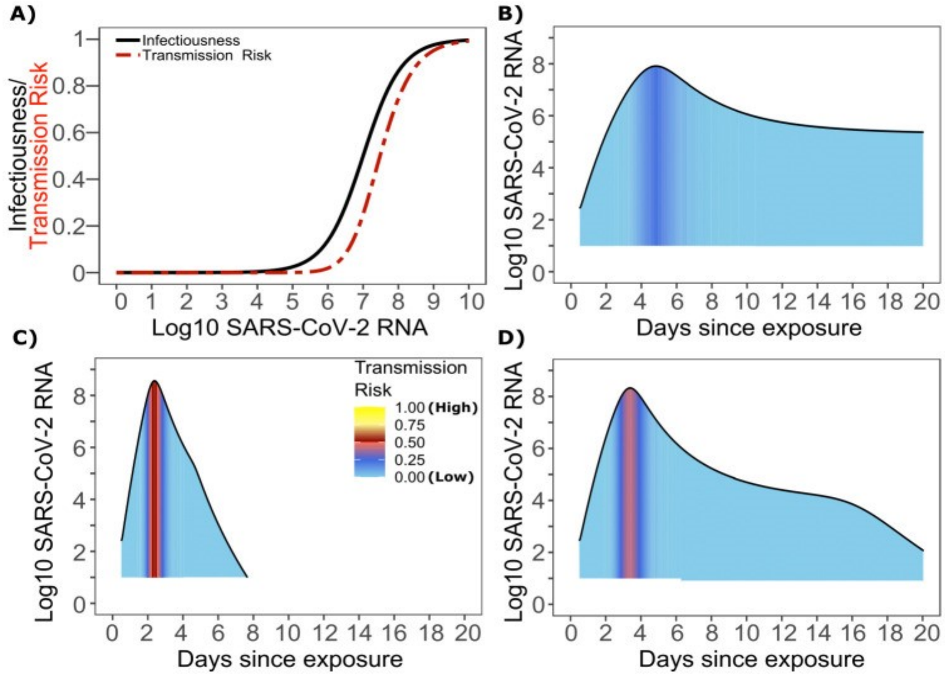

Figure 2-5 SARS-CoV-2 transmission probability as a function of shedding. A. Optimal infectious dose (ID) response curve (infection risk = Pt) and transmission dose (TD) response curve (transmission risk = Pt * Pt) curves for SARS-CoV-2. Transmission probability is a product of two probabilities, contagiousness and infectiousness. B-D. Three simulated viral shedding curves. Heat maps represent risk of transmission at each shedding timepoint given an exposed contact with an uninfected person at that time.

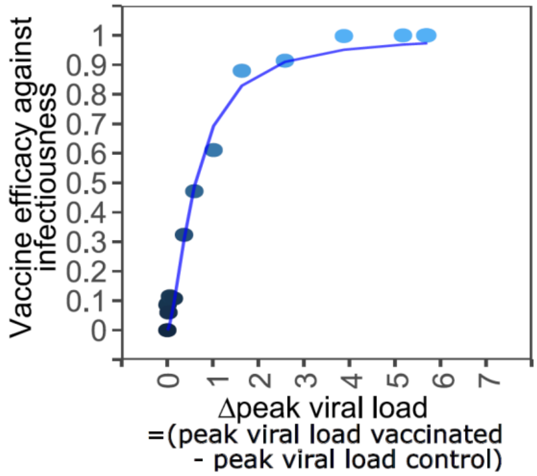

Figure 2-6 Vaccine efficacy against infectiousness versus the change in peak viral load. Model by Goyal et al (personal communication).

2.8 Evidence for viral load as a marker of COVID-19 disease severity and clinical course

Beyond infectiousness, there is a growing body of evidence supporting a linkage between VL and COVID-19 disease outcome. Two Phase 2/3 trials of monoclonal antibodies for COVID-19 treatment in outpatient settings found that VL at enrollment and early after intervention was associated with future clinical outcomes and disease course (30, 31). In the BLAZE-1 trial of intravenous infusion of the LY-CoV555 antibody, on day 7 post-infusion the frequency of hospitalization among patients with a high VL (Ct value of less than 27.5) was 12% (7 of 56 patients), as compared to 0.9% (3 of 340 patients) among those with a lower VL. Numerous observational studies have also identified associations between VL and clinical outcomes (32). The highest quality evidence comes from large cohort studies. In a large hospitalized cohort, Pujadas et al (33) estimated a highly significant 7% increase in mortality per log10 increase in VL at admission, after adjusting for other factors associated with clinical outcomes ($p = 0.0014$, multivariate Cox model). Chen et al (34) confirmed this finding in a larger cohort of patients admitted to three New York City hospitals. These data suggest that VL at and soon after the time of symptom onset may be a useful measure of disease severity.

2.9 Methods for capturing transmission

Reducing transmission is key to ending/controlling the COVID-19 pandemic. Due to vaccination, the number of asymptomatic individuals might increase as vaccine-induced immune responses may lead to VL reduction. However, it is still to be discovered whether those individuals will continue to shed and transmit virus to others. Hence, it is important to identify secondary transmissions from study participants to estimate secondary infections rates by arm and to validate VL as a surrogate for transmission risk. Onward transmission of

SARS-CoV-2 infection from study participants can be identified in combination with sequence-analysis and phylogenetics methods (35).

Estimating vaccine efficacy in reducing transmission (VE_I), requires measuring transmission of SARS-CoV-2 from vaccinated and unvaccinated individuals to others (36, 37). Measuring transmission of infected individuals to uninfected individuals is challenging, especially for transient infections where it may be more difficult to ascertain which infection occurred first. For example, because HIV is not transient and per-exposure transmission is low, resulting in a potentially long time period of exposure, transmission can be studied in sero-discordant sexual partnerships. In a transient infection, ideally both members of the transmission pair are monitored before either is infected so that timing and direction of transmission can be easily determined. Prospectively following a cohort of vaccinated and unvaccinated individuals and their close contacts to observe whether infection is transmitted from study participants to close contacts, or from contacts to study participants, based on frequent testing of both allows for better quantification of the temporality of events regarding infection transmission, if it occurs. To do this, Datta et al. propose augmenting a randomized, controlled vaccine trial with a cohort of close contacts of trial participants. Both trial participants and the contact cohort are followed prospectively for acquisition, allowing for estimation of VE_I. Another approach to capturing transmission events is a case ascertainment design (38, 39). Under this design, contacts of index cases are enrolled and tested as the cases emerge over time. A similar case ascertainment design was used for a recent household study for SARS-CoV-2 prevention. In the HCQ PEP trial, which was a household-randomized, double-blind, controlled trial of hydroxychloroquine postexposure prophylaxis to prevent acquisition of SARS-CoV-2 among close contacts recently exposed to persons diagnosed with SARS-CoV-2 infection, individuals were self-referred from infected individuals and enrolled online and all study procedures were done remotely including informed consent and daily self-nasal swabbing for 14 days (40). A case-ascertained approach is more resource-efficient than a prospective contact approach which requires enrollment of a large number of contacts, most of whom will not become infected nor have an infected index case in the main study. Because contacts are only identified and tested after index case diagnosis, the case-ascertained approach also enables assessment of secondary transmission in contacts not participating in frequent, prospective testing programs. However, the case-ascertained approach has the potential to miss or misclassify transmission events given the gap between diagnosing an index case and identifying, enrolling, and testing a contact. Index cases may also experience stigma when identifying contacts at the time of diagnosis, as opposed to when identifying contacts prospectively and before infection. The feasibility of recruiting and retaining university contacts, and of classifying transmission events among contacts, using the two designs has yet to be evaluated and compared.

This study will employ both the augmented and case ascertainment approaches to studying transmission through enrollment of two contact cohorts, a Prospective Close Contact (PCC) cohort and a Case-Ascertained Close Contact (CACC) cohort. The PCC Cohort will be identified at enrollment as close and sustained contacts of randomized participants (eg, roommates or co-workers). Participants in the PCC cohort will undergo routine screening for SARS-CoV-2 infection as part of their attendance or employment at their university, which will allow the participant and study team to receive the same real-time clinical diagnosis of

COVID-19 during the study as main study participants. Additionally, when a positive case is identified for a main study participant, the participant ('index case') will identify and refer their recent close contacts for enrollment into the CACC cohort. For both cohorts, infection diagnosis of the associated main study participant will trigger intensive short term sample collection (with a potential delay for the CACC cohort due to the need for informed consent and enrollment) including sera followed by daily nasal swabs for 14 days. Assessment of viral sequences, viral load trajectories, antibody detection and epidemiologic information from the infected participant and any positive close contacts in the PCC or CACC will aid in identifying transmission events between the participant and the contact. Details of the enrollment and procedures for both contact cohorts are described below.

2.10 Moderna COVID-19 Vaccine

This trial will utilize vaccine obtained under EUA by the US Government's COVID-19 Vaccine Response.

2.10.1 Clinical studies of Moderna COVID-19 Vaccine

Table 2-3 Summary of clinical studies

ClinicalTrials.gov Identifier	Study number	Phase	N	Dose groups	Route	Schedule	Reference
NCT04283461	P101	1	120	25, 50, 100, or 250 mcg	IM	M0, M1	(41, 42)
NCT04405076	P201	2	600	50 or 100 mcg	IM	M0, M1	(14, 15, 43)
NCT04470427	P301	3	30,351	100 mcg	IM	M0, M1	(14, 15, 43)

2.10.2 Potential risks of Moderna COVID-19 Vaccine

Risks associated with receipt of the Moderna COVID-19 Vaccine are described in the "Fact Sheet For Healthcare Providers Administering Vaccine (Vaccination Providers) Emergency Use Authorization (EUA) Of The Moderna COVID-19 Vaccine To Prevent Coronavirus Disease 2019 (COVID-19)", available at https://www.modernatx.com/covid19vaccine-eua/.

2.11 Trial design rationale

We are proposing a 2-arm randomized, controlled, open-label trial design. The design is augmented by two cohorts of contacts of study participants – the Prospective Close Contact

(PCC) cohort and the Case-Ascertained Close Contact (CACC) cohort – to allow capture of secondary transmission events.

Participants in the main study will self-collect nasal swabs daily in order to capture all incident SARS-CoV-2 infection events over 4 months of follow-up and to capture the full course of viral shedding – from onset of infection to viral clearance – among those with both asymptomatic and symptomatic infection.

To minimize participant burden and maximize study efficiency, as many study procedures as possible will be conducted remotely (not requiring an in-person visit) and electronically, such as initial screening and consent. Participants diagnosed with SARS-CoV-2 infection will complete daily e-diaries to capture symptoms.

Given that primary analyses will be conducted among baseline seronegative participants, the rate of baseline SARS-CoV-2 seropositivity will be monitored operationally with an operational target of no more than 10% for the main study cohort.

2.11.1 Dose and schedule

The latest information regarding Moderna COVID-19 Vaccine dose and administration instructions can be found at https://www.modernatx.com/covid19vaccine-eua/.

For this study, vaccine will be administered at D1 and D29 for the immediate vaccination arm, and at D113 and D141 for the delayed vaccination arm.

2.11.2 Choice of delayed vaccination

There is no licensed SARS-CoV-2 vaccine currently available to serve as a reference control. Currently, there are two SARS-CoV-2 vaccines available in the US under Emergency Use Authorization (EUA), including the Moderna COVID-19 Vaccine being used in this study. Accordingly, a delayed vaccination arm has been chosen as an internal control. No placebo will be used, and participants will not be blinded to randomization assignment. The open-label design has been chosen primarily to achieve operational efficiency and to minimize resource utilization.

With an open-label design, differences between the randomized arms in study endpoints including SARS-CoV-2 infection, viral load, and secondary transmission will capture not only biological effects of vaccination, but potentially behavioral effects influencing exposure, including adherence to study procedures. This is most relevant for the SARS-CoV-2 infection endpoint: if participants who are randomized to immediate vaccination engage in more risk-taking behavior and thus experience higher exposure to SARS-CoV-2 relative to participants randomized to delayed vaccination, ie, there is behavioral disinhibition, this will be captured in the estimate of vaccine efficacy against infection: the biological reduction in incidence of SARS-CoV-2 infection attributable to vaccination will be offset by an increase in incidence due to higher exposure. Therefore, the measure of vaccine efficacy against infection that is estimated in this study is in truth a measure of effectiveness. However, we retain the terminology “vaccine efficacy” for simplicity. Section 6.5.3.1 discusses this issue

in greater detail, including how the statistical analysis will attempt to control for potential imbalances in SARS-CoV-2 exposure between treatment arms when evaluating vaccine efficacy, and how both efficacy and effectiveness measures will be evaluated and compared. We note that endpoints measured after diagnosis of SARS-CoV-2 infection, including SARS-CoV-2 viral load and secondary transmission, are viewed as much less likely to be influenced by participant behavior and knowledge of vaccine receipt.

3 Objectives and endpoints

**Primary Objectives**	**Primary Endpoints**
1. To evaluate the efficacy of Moderna COVID-19 Vaccine against SARS-CoV-2 infection (ie, to evaluate vaccine efficacy against infection or VE_S).	SARS-CoV-2 infection diagnosed by PCR post visit 3 (month 1) among participants who were SARS-CoV-2 seronegative at enrollment and SARS-CoV-2 negative by PCR and serology through visit 3 (month 1)
2. To evaluate the effect of Moderna COVID-19 Vaccine on peak nasal viral load as a measure of infection and a proxy of infectiousness	Peak viral load in nasal samples from participants diagnosed with SARS-CoV-2 infection post visit 3 (month 1), among participants who were SARS-CoV-2 seronegative at enrollment and SARS-CoV-2 negative by PCR and serology through visit 3 (month 1)
**Secondary Objectives**	**Secondary Endpoints**
1. To evaluate the impact of Moderna COVID-19 Vaccine on secondary transmission of SARS-CoV-2 infection, using data for the Prospective Close Contact (PCC) cohort and Case-Ascertained Close Contact (CACC) cohort (ie, to evaluate vaccine efficacy against infectiousness or VE_I)	Number of adjudicated* secondary transmission events in PCC and CACC cohorts from main study participants who were SARS-CoV-2 seronegative at enrollment and SARS-CoV-2 negative by PCR and serology through visit 3 (month 1).<br>*based on questionnaire-based epidemiologic data, antibody and viral dynamics, and viral sequence data
2. To evaluate the efficacy of Moderna COVID-19 Vaccine to prevent serologically confirmed SARS-CoV-2 infection	SARS-CoV-2 antibodies to the nucleocapsid protein post visit 3 (month 1), among participants who were SARS-CoV-2 seronegative at enrollment and SARS-CoV-2 negative by PCR and serology through visit 3 (month 1)
3. To evaluate vaccine efficacy against COVID-19 disease, (ie, to evaluate VE_D) using weekly e-diaries and confirmatory PCR testing	SARS-CoV-2 infection confirmed by PCR and diagnosed post-visit 3 (month 1), among participants SARS-CoV-2 who were seronegative at enrollment and SARS-CoV-2 negative by PCR and serology through visit 3 (month 1), coincident with the following symptoms:<br>• at least TWO of the following systemic symptoms: Fever (≥ 38ºC), chills, myalgia, headache, sore throat,<br>OR<br>at least ONE of the following signs/symptoms: cough, shortness of breath or difficulty breathing, new olfactory or taste disorder, clinical or radiographical evidence of pneumonia, thromboembolic event, myocardial infarction, myocarditis, chilblains, or multi-inflammatory syndrome

4. To evaluate vaccine effect on additional measures of magnitude of viral load over time, e.g. area under the viral load curve, duration of shedding, and time to log10 viral load above 10^5 copies/mL	Summary measures of the viral load curve, all evaluated among participants diagnosed with SARS-CoV-2 infection post-visit 3 (month 1) who were SARS-CoV-2 seronegative at enrollment and SARS-CoV-2 negative by PCR and serology through visit 3 (month 1):<br>• Transmission potential endpoint, defined as 0 for an individual without SARS-CoV-2 infection, and peak log10 viral load, for an individual with SARS-CoV-2 infection<br>• Area under log10 viral load curve (AUC)<br>• Area under log10 viral load curve above 10^5 copies/mL<br>• Time from enrollment to viral load above 10^5 copies/mL
5. To evaluate vaccine efficacy against SARS-CoV-2 infection, the vaccine effect on viral load, and the vaccine effect on secondary transmission in all enrolled participants without regard to baseline SARS-CoV-2 serostatus	• SARS-CoV-2 infection diagnosed by PCR [Primary endpoint 1]<br>• Peak log10 viral load in nasal samples from participants who are diagnosed with SARS-CoV-2 infection [Primary endpoint 2]<br>• Number of secondary transmission events in the PCC and CACC [Secondary endpoint 1]
6. To evaluate the immunogenicity of the vaccine	Magnitude and response rate of immune responses to vaccination measured at Months 0, and 2 stratified by baseline SARS-CoV-2 serostatus as measured by binding antibody and neutralization assays in the case-cohort immunogenicity set
7. To evaluate immune responses 1 month post-second vaccination as correlates of risk of SARS-CoV-2 acquisition, viral load, secondary infection, and COVID-19 disease among vaccine recipients in the immediate vaccination group	Magnitude and response rate of immune responses to vaccination in the immediate vaccination group and measured at Months 0 and 2, stratified by baseline SARS-CoV-2 serostatus, as measured by binding antibody and neutralization assays in the case-cohort immunogenicity set
8. To evaluate vaccine efficacy against asymptomatic SARS-CoV-2 infection	SARS-CoV-2 infection by PCR or monthly serology post-visit 3 (month 1) among participants seronegative for SARS-CoV-2 at baseline, SARS-CoV-2 negative by PCR and serology through visit 3 (month 1), and without any prior reporting of symptoms that led to confirmation of a COVID-19 disease endpoint
9. To evaluate vaccine efficacy against SARS-CoV-2 infection and COVID-19 disease and the vaccine effect on viral load and secondary	Endpoints evaluated among participants who were SARS-CoV-2 seronegative at enrollment and SARS-CoV-2 negative by PCR and serology through visit 3

transmission among participants who received all planned immunizations at the designated immunization visits within specific visit windows	(month 1) and counting SARS-CoV-2 infection events post-visit 3 (month 1)<br>• SARS-CoV-2 infection diagnosed by PCR [Primary endpoint 1]<br>• Peak log10 viral load in nasal samples from participants who are diagnosed with SARS-CoV-2 infection [Primary endpoint 2]<br>• Number of secondary transmission events in the PCC and CACC [Secondary endpoint 1]<br>• SARS-CoV-2 infection coincident with signs and symptoms of COVID-19 listed under Secondary endpoint 3
10. To evaluate the association between measures of the magnitude of viral load over time and secondary transmission of SARS-CoV-2 infection, among individuals with incident SARS-CoV-2 infection in each treatment group, i.e. to conduct analyses aimed at evaluating viral load measures as surrogates of secondary transmission	• Peak log10 viral load [Primary endpoint 2] and other viral load summaries [Secondary endpoints 4]<br>• Number of secondary transmission events in the PCC cohort and CACC cohort [Secondary endpoint 1]
11. To evaluate vaccine effects on peak viral load and on secondary transmission of SARS-CoV-2 infection separately among individuals with asymptomatic vs. symptomatic SARS-CoV-2 infection	• Peak log10 viral load [Primary endpoint 2] and other viral load summaries [Secondary endpoints 4]<br>• Number of secondary transmission events in the PCC cohort and CACC cohort [Secondary endpoint 1]<br>• SARS-CoV-2 infection diagnosed using PCR, with and without signs and symptoms of COVID-19 listed under Secondary endpoint 3

Exploratory Objectives

1. To evaluate the associations between demographic and clinical factors and secondary transmission of SARS-CoV-2 infection and COVID-19 disease, among individuals with incident SARS-CoV-2 infection in the delayed vaccination arm and their contacts in the PCC and CACC
2. To assess if and how vaccine efficacy against SARS-CoV-2 infection, transmission and COVID-19 disease depends on baseline demographic, clinical, socio-economic indicators, and/or behavioral characteristics of study participants
3. To assess if and how vaccine efficacy against SARS-CoV-2 infection and COVID-19 disease depends on genotypic or neutralization phenotypic characteristics of SARS-CoV-2 (sieve analysis)
4. To further evaluate immunogenicity of the vaccine, additional immunogenicity assays may be performed such as antibody (Ab) Avidity and Fc Receptor function assays, based on the CoVPN Laboratory Center assay portfolio
5. To conduct analyses related to furthering the understanding of SARS-CoV-2, immunology, vaccines, and clinical trial conduct

4 Ethical considerations

It is critical that universally accepted ethical guidelines are followed at all sites involved in the conduct of clinical trials. The COVID-19 Prevention Network (CoVPN) have addressed ethical concerns in the following ways:

- CoVPN trials are designed and conducted to enhance the scientific knowledge base necessary to find a preventive vaccine, using methods that are scientifically rigorous and valid, and in accordance with International Council for Harmonisation of Technical Requirements for Pharmaceuticals for Human Use (ICH) and/or other Good Clinical Practice (GCP) guidelines.

- CoVPN scientists and operational staff incorporate the philosophies underlying major codes (44-46), declarations, and other guidance documents relevant to human subjects research into the design and conduct of SARS-CoV-2 vaccine clinical trials.

- CoVPN scientists and operational staff are committed to substantive community input—into the planning, conduct, and follow-up of its research—to help ensure that locally appropriate cultural and linguistic needs of study populations are met.

- The CoVPN provides training so that all participating sites similarly ensure fair participant selection, protect the privacy of research participants, and obtain meaningful informed consent. During the study, participants will have their wellbeing monitored, and to the fullest extent possible, their privacy protected. Participants may withdraw from the study at any time.

- Prior to implementation, CoVPN trials are rigorously reviewed by scientists who are not involved in the conduct of the trials under consideration.

- CoVPN trials are reviewed by local and national regulatory bodies and are conducted in compliance with all applicable national and local regulations.

- The CoVPN designs its research to minimize risk and maximize benefit to both study participants and their local communities. For example, CoVPN protocols provide enhancement of participants' knowledge of SARS-CoV-2 and SARS-CoV-2 prevention, as well as counseling, guidance, and assistance with any social impacts that may result from research participation. CoVPN protocols also include careful medical review of each research participant's health conditions and reactions to study products while in the study.

- CoVPN research aims to benefit local communities by directly addressing the health and SARS-CoV-2 prevention needs of those communities and by strengthening the capacity of the communities through training, support, shared knowledge, and equipment. Researchers involved in CoVPN trials are able to conduct other critical research in their local research settings.

- The CoVPN values the role of in-country Institutional Review Boards (IRBs), Ethics Committees (ECs), and other Regulatory Entities (REs) as custodians responsible for ensuring the ethical conduct of research in each setting.

5 IRB/EC review considerations

US Food and Drug Administration (FDA) and other US federal regulations require IRBs/ECs/REs to ensure that certain requirements are satisfied on initial and continuing review of research (Title 45, Code of Federal Regulations (CFR), Part 46.111(a) 1-7; 21 CFR 56.111(a) 1-7). The following section highlights how this protocol addresses each of these research requirements. Each CoVPN Investigator welcomes IRB/EC/RE questions or concerns regarding these research requirements.

5.1 Minimized risks to participants

45 CFR 46.111 (a) 1 and 21 CFR 56.111 (a) 1: Risks to subjects are minimized.

This protocol minimizes risks to participants by (a) correctly and promptly informing participants about risks so that they can join in partnership with the researcher in recognizing and reporting harms; (b) respecting local/national blood draw limits; (c) performing direct observation of participants postvaccination and reporting information regarding side effects to the FDA/CDC Vaccine Adverse Event Reporting System (VAERS) per EUA guidance; (d) having staff properly trained in administering study procedures that may cause physical harm or psychological distress, such as blood draws, vaccinations, COVID-19 testing and counseling; and (e) providing safety monitoring.

5.2 Reasonable risk/benefit balance

45 CFR 46.111(a) 2 and 21 CFR 56.111(a) 2: Risks to subjects are reasonable in relation to anticipated benefits, if any, to subjects, and the importance of the knowledge that may reasonably be expected to result.

In all public health research, the risk-benefit ratio may be difficult to assess because the benefits to participants in prevention protocols are not as apparent as they would be in treatment protocols, where a study participant may be ill and may have exhausted all conventional treatment options. However, this protocol is designed to minimize the risks to participants while maximizing the potential value of the knowledge it is designed to generate.

5.3 Equitable participant selection

45 CFR 46.111 (a) 3 and 21 CFR 56.111 (a) 3: Subject selection is equitable

This protocol has specific inclusion and exclusion criteria for investigators to follow in admitting participants into the protocol. Participants are selected because of these criteria and not because of positions of vulnerability or privilege. Investigators are required to maintain screening and enrollment logs to document volunteers who screened into and out of the protocol and for what reasons.

5.4 Appropriate informed consent

45 CFR 46.111 (a) 4 and 5 and 21 CFR 56.111 (a) 4 and 5: Informed consent is sought from each prospective subject or the subject's legally authorized representative as required by 45 CFR 46.116 and 21 CFR Part 50; informed consent is appropriately documented as required by 45 CFR 46.117 and 21 CFR 50.27

The protocol specifies that informed consent must be obtained before any study procedures are initiated and assessed throughout the trial (see Section 9.1). Each site is provided training in informed consent by the CoVPN and is required to have a Standard Operating Procedure (SOP) on the informed consent process. The CoVPN requires a signed consent document for documentation, in addition to chart notes or a consent checklist.

5.5 Adequate safety monitoring

45 CFR 46.111 (a) 6 and 21 CFR 56.111 (a) 6: There is adequate provision for monitoring the data collected to ensure the safety of subjects.

Vaccination providers are required to report to VAERS the certain adverse events after COVID-19 vaccination, under Emergency Use Authorization (EUA) and encouraged to report adverse events to ModernaTX |(https://www.cdc.gov/vaccines/covid-19/vaccination-provider-support.html, see Section 11). Additionally, the use of v-safe will be recommended to vaccine recipients (https://www.cdc.gov/coronavirus/2019-ncov/vaccines/safety/vsafe.html, see Section 11), and an Independent Data Monitoring Committee (IDMC) will periodically review study data.

5.6 Protect privacy/confidentiality

45 CFR 46.111 (a) 7 and 21 CFR 56.111 (a) 7: There are adequate provisions to protect the privacy of subjects and maintain the confidentiality of data.

Privacy refers to an individual's right to be free from unauthorized or unreasonable intrusion into his/her private life and the right to control access to individually identifiable information about him/her. The term "privacy" concerns research participants or potential research participants as individuals whereas the term "confidentiality" is used to refer to the treatment of information about those individuals. This protocol respects the privacy of participants by informing them about who will have access to their personal information and study data (see Appendix A, Appendix B and Appendix C). The privacy of participants is protected by assigning unique identifiers in place of the participant's name on study data and specimens. In the United States, research participants in CoVPN protocols are protected by a Certificate of Confidentiality from the US NIH, which can prevent disclosure of study participation even when that information is requested by subpoena. Participants are told of the use and limits of the certificate in the study consent form. In addition, each staff member at each study site in this protocol signs an Agreement on Confidentiality and Use of Data and Specimens with the

CoVPN. In some cases, a comparable confidentiality agreement process may be acceptable. Each study site participating in the protocol is required to have a standard operating procedure on how the staff members will protect the confidentiality of study participants.

6 Statistical considerations

We anticipate enrolling approximately 12,000 students. Participants will be randomized 1:1 to immediate or delayed vaccination, at Months 0 and 1 or Months 4 and 5, respectively. Randomization will be stratified by place of residence, for each university.

Primary analyses of vaccine efficacy against infection (VE_S) and of the vaccine effect on viral load (ΔVL) will be conducted among participants SARS-CoV-2 seronegative at enrollment in the "Day 29 Negative Set", i.e. the set of participants who are SARS-CoV-2 seronegative at Day 29 (the second vaccination visit, for the immediate vaccination arm) and without seroconversion or PCR-confirmed infection on or prior to Day 29; see Table 6-1 for a full definition of the various analysis sets for the study. Primary efficacy analyses will only count SARS-CoV-2 infection events diagnosed post-Day 29. Secondary analyses will be conducted among all participants who receive both immunizations without protocol violation, i.e. the Per-Protocol Set, and in the set of all enrolled participants (the "Full Analysis Set" or FAS), counting infection events diagnosed after enrollment (Day 0). Immunogenicity assessment will be done in the Case-Cohort Immunogenicity Set—a subsample of the participants randomized to immediate vaccination and sampled on the basis of SARS-CoV-2 infection status and baseline characteristics that will be fully described in the study SAP.

The FAS differs from a full Intention-to-Treat set by excluding randomized volunteers who do not enroll. The exclusion of these individuals is justified because the effects of the vaccine are unlikely to influence such exclusion. Because of the brief length of time between randomization and enrollment (ideally these will be on the same day), we expect almost all randomized volunteers to be in the FAS.

All primary and secondary analyses will be done according to the treatment randomly assigned ("as randomized").

While main study participants will be tested using both the real-time university testing system and using PCR testing of stored swabs, it is the latter that will be used to identify SARS-CoV-2 infection events for primary analysis.

The two contact cohorts – the PCC and the CACC-- will both be used for analyses of the vaccine effect on secondary transmission, as described below.

The study is "time-driven", i.e. the primary analysis will occur when all participants have completed the 5 months follow-up (with 4 months/16 weeks of primary follow-up and a final 5 month contact). The number of primary endpoint infection events that accrue in the study is random and will depend on the level of SARS-CoV-2 incidence in each arm.

Table 6-1 Study Analysis Sets and Definitions

Analysis Set	Definition	Analyses
Full Analysis Set (FAS)	Randomized participants who enroll in the study	Secondary efficacy analyses*
Day 29 Negative (D29-) Set	Participants in the FAS who were seronegative at Day 29 and without seroconversion or PCR-confirmed infection on or before Day 29	Primary efficacy analyses*
Per-Protocol (PP) Set	Participants in the D29- set who received all planned immunizations at the designated immunization visits within specific visit windows	Secondary efficacy analyses*
Day 29 Negative Transmission Set (D29-TS)	Participants in the D29- set and their contacts in the Prospective and Case Ascertained Contact Cohorts	Primary analyses of secondary transmission*
Case-Cohort Immunogenicity Set (ccIS)	Subset of the FAS that is randomly sampled for measurement of immunological markers and fully described in study SAP	Immunology marker analyses

*These analysis sets include participants without regard to baseline SARS-CoV-2 status. However, primary efficacy analyses will condition on baseline SARS-CoV-2 seronegative status.

Table 6-2 Assumptions underlying study power/sample size calculations, unless otherwise specified. Parameters and their assumed values pertain to participants in the main study cohort.

Parameter	Assumption
Enrollment	Uniform enrollment over 4 weeks
Baseline seroprevalence in main study and PCC and CACC	10% of enrolled participants are baseline SARS-CoV-2 seropositive. This parameter is monitored and controlled operationally (see Section 6.3)
Per-protocol rate	98% of enrolled participants are in the per-protocol set
Loss to follow-up rate in the main study, the PCC and the CACC	5% of participants are lost to follow-up over 16 weeks at a constant rate
SARS-CoV-2 incidence in the delayed vaccination group and incidence un-linked to main study participants in the PCC	Exponential (constant rate) over 16 weeks; primary assumption is 4.0% over 16 weeks
Secondary transmission rate among susceptible contacts, in the delayed vaccination arm	15% transmission rate based on (47-49)
Vaccine efficacy (VE) against SARS-CoV-2 infection and transmission	Constant after 14 days post-first vaccination (calculations do not depend on earlier VE); level is varied
Contact enrollment	For the PCC: 60% of main study participants enroll contact(s) and the average number of contacts enrolled is 2<br>For the CACC: 75% of main study participants enroll contact(s) and the average number of contacts enrolled is 3

6.1 Accrual restrictions

Accrual into the main study cohort will be restricted in order to limit vaccine coverage and potential interference among study participants. Specifically, no more than 1 main study

participant will be eligible for enrollment per sleeping area of a given residence at a given university. Other individuals who are screened for participation may be eligible for enrollment into the PCC cohort; main study enrollment will be first-come, first-served.

6.2 Accrual and sample size calculations

A sample size of 6,000 participants in each treatment arm ensures a high probability that a sufficient number of primary endpoint events, i.e. SARS-CoV-2 infections among baseline seronegative participants in the D29- set and diagnosed after Day 29, will accrue to adequately power analyses addressing both primary objectives as well as key secondary objectives. Specifically, under a 4% SARS-CoV-2 incidence over 16 weeks in the delayed vaccination group (a conservative assumption given recent incidence on US college campuses, see Section 2.5), assuming VE_S = 50% and other assumptions laid out in Table 6-2, 225 primary endpoint events will occur across both arms with probability 0.5 and 214 events will occur with probability 0.8.

Table 6-3 shows the expected number of primary endpoint events, overall and for each treatment group, as the assumed incidence in the delayed vaccination group varies. As shown, the design ensures a sufficient number of primary endpoint events to address a variety of objectives that condition on the primary endpoint. For example, with 4% SARS-CoV-2 incidence in the delayed vaccination group, 75 primary endpoint events are expected in the immediate vaccination group for immune correlates analyses.

Table 6-3 Expected number of primary endpoint events, available for secondary analyses that condition on primary endpoint event status. Calculations assume 6,000 participants per arm and other assumptions in **Table 6-2**, VE_S = 50%, and varying 16-week incidence of SARS-CoV-2 infection in the delayed vaccination group.

**16-Week SARS-CoV-2 Incidence in Placebo Arm**	**Number of Primary Endpoint Events**	
	Both Arms	Immediate Vaccination Arm
1.5%	86	28
2%	143	47
3%	170	56
3.5%	198	66
4%	225	75

The study provides high power to evaluate the vaccine effect on acquisition (VE_S). Figure 6-1 shows the power of the study to detect a given level of VE_S, rejecting H_0: $VE_S \leq 30\%$, based on a 1-sided 0.025-level log-rank test and as a function of the SARS-CoV-2 incidence in the delayed vaccination group. With 4% 16-week incidence, the study has 90% power to detect any $VE_S > 57\%$; with 3% incidence, $VE_S > 60\%$ can be detected with 90% power.

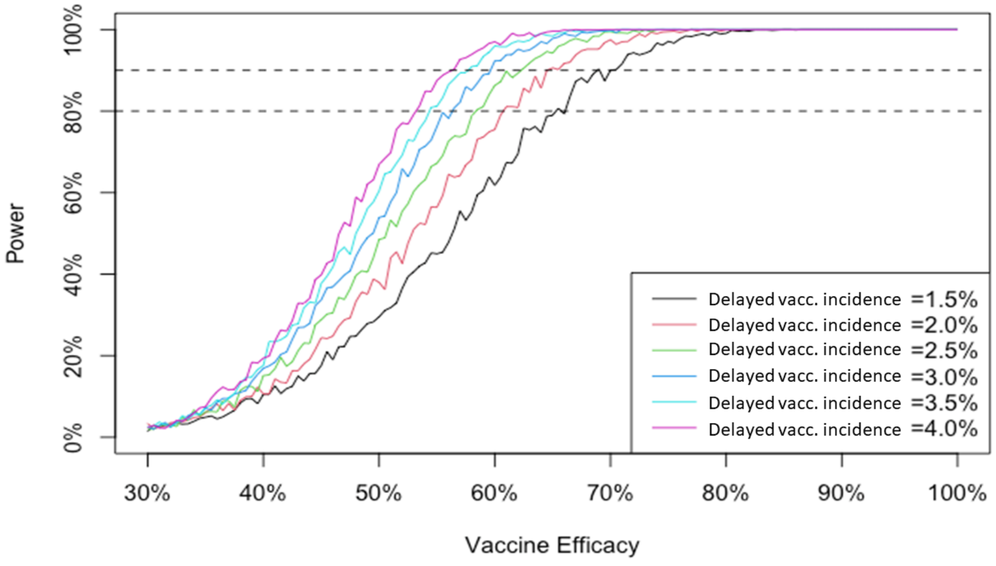

Figure 6-1 Power to detect varying vaccine efficacy (VE) against SARS-CoV-2 infection as a function of the delayed vaccination group incidence over 16 weeks. Simulation-based power calculations assume 6,000 participants in each arm and other assumptions listed in **Table 6-2**. Power based on 1-sided 0.025-level log-rank test and 1,000 simulations.

The proposed study also provides adequate precision with which to estimate VE_S. Table 6-4 shows that, with 225 primary endpoint events, the approximate 95% confidence interval (CI) about an estimated 50% VE_S is (36.9%, 60.4%). More precise inference is obtained under higher VE_S and higher primary endpoint totals.

Table 6-4 Approximate 95% confidence interval around a given estimated VE_S against SARS-CoV-2 infection, comparing the immediate vaccination arm vs. the delayed vaccination arm, as a function of the number of primary endpoint events across the two groups.

Number of primary endpoint events	Approximate 95% CI for estimated VE = 50%	Approximate 95% CI for estimated VE = 70%
200	(36.0%, 60.9%)	(60.7%, 77.2%)
225	(36.9%, 60.4%)	(61.1%, 76.9%)
250	(37.7%, 59.9%)	(61.6%, 76.6%)

6.2.1 Power and precision for evaluating the vaccine effect on VL

Emerging data on acute SARS-CoV-2 infection suggest that with an expected 225 primary endpoint events, the vaccine effect on mean peak log10 viral load among primary endpoint infections can be estimated with adequate precision. Specifically, assuming that peak log10 viral load is normally distributed with a 1.8 standard deviation in both treatment groups, and assuming 50% VE_S, a 1-log reduction in mean peak viral load can be detected with 96.9% power given 225 primary endpoint events; power remains high (93.5%) with 70% VE_S. Table 6-5 shows power to detect a 1-log reduction in peak log viral load as a function of the assumed standard deviation in log peak viral load which is allowed to vary between 1.0 and

2.5. The standard deviation in peak log viral load in a cohort of 68 individuals associated with the National Basketball Association who were diagnosed with acute SARS-CoV-2 infection was 1.4 to 1.8, depending on the set of samples included in the analysis (50). Emerging data from the HCQ-PEP study of ~100 acutely infected household members of recently diagnosed COVID-19 cases suggests a standard deviation in peak log viral load in the range of 2.0-2.2, depending on the set of samples included [data not published]. Table 6-6 shows the approximate 95% CI about an estimated 1-log reduction in mean peak log viral load, demonstrating the precision that is obtained with 225 primary endpoint events. Modest precision is observed with a 1-1.8 standard deviation in the endpoint, with lower precision under a higher standard deviation; precision is also lower under increasing VE_S.

Table 6-5 Power to detect a 1-log reduction in mean peak log viral load comparing primary endpoint infection events between the immediate and delayed vaccination groups, as a function of the assumed standard deviation in peak log viral load in both groups and the assumed level of VE against SARS-CoV-2 infection. Simulation-based power calculations assume 225 primary endpoint events across treatment groups. Power based on 1-sided 0.025-level t-test and 1,000 simulations.

Std. dev. in peak log viral load	VE_S = 50%	VE_S = 70%
**1**	100%	100%
**1.5**	99.6%	97.8%
**1.8**	96.9%	93.5%
**2**	95.6%	86.5%
**2.5**	79.9%	68.7%

Table 6-6 Approximate 95% confidence interval around an estimated 1-log reduction in mean peak log viral load comparing primary endpoint infection events between immediate and delayed vaccination groups, as a function of the assumed standard deviation in peak log viral load in both groups and the assumed level of vaccine efficacy against SARS-CoV-2 infection. Calculations assume 225 primary endpoint events across treatment groups.

Std. dev. in peak log viral load	VE_S = 50%	VE_S = 70%
**1**	(0.72, 1.28)	(0.68, 1.31)
**1.5**	(0.57, 1.41)	(0.51, 1.46)
**1.8**	(0.50, 1.50)	(0.44, 1.57)
**2**	(0.46, 1.57)	(0.36, 1.61)
**2.5**	(0.29, 1.65)	(0.20, 1.74)

6.2.2 Power for evaluating the vaccine effect on secondary transmission

The study has adequate power to evaluate vaccine efficacy against secondary transmission, based on analyses that are conducted among primary endpoint cases and their contacts in the PCC and CACC, i.e. based on conditional analyses, as long as VE against SARS-CoV-2 infection is low. As demonstrated in Figure 6-2, if there is high vaccine efficacy against SARS-CoV-2 infection, power is low to evaluate secondary transmission using a conditional analysis. In this case, an unconditional analysis of VE against secondary transmission is

expected to be more powerful, and also more appropriate given that it reflects that infections blocked have no transmission potential.

We performed simulation-based calculations to explore the power to evaluate VE against secondary transmission based on a conditional analysis. In addition to the assumptions in Table 6-2, we assumed: equal infection surveillance in the PCC and CACC, for simplicity; that PCC contacts acquire infection from outside sources (other than the index case) at the same rate as the delayed vaccination arm; that only baseline seronegative contacts and those in the PCC not infected by outside sources are at risk of secondary transmission; that the number of secondary transmission events among at-risk contacts follows a Poisson distribution in each arm, with VE against secondary transmission reducing the event rate proportionally in the immediate vaccination group. A one-sided 0.025-level likelihood ratio test based on a Poisson regression model was used to evaluate VE against secondary transmission. As shown in Figure 6-2, the study has 90% power to detect a 59% reduction in secondary transmission if VE_S = 50%; a higher 69% reduction in secondary transmission is detectable if VE_S = 70%. Table 6-7 helps explain the power results: it shows the amount of statistical information on which the analysis is based, ie, the median number of primary endpoint events and secondary endpoint events per arm. With 50% VE_S and 50% VE against secondary transmission, we expect 15 secondary transmission events in the immediate vaccination arm and 59 in the delayed arm; the limited number of events is due to the “transmission cascade” whereby under our assumptions only some primary endpoint events enroll contacts into the PCC and CACC, only some contacts are susceptible and retained, and the transmission rate is low (15%).

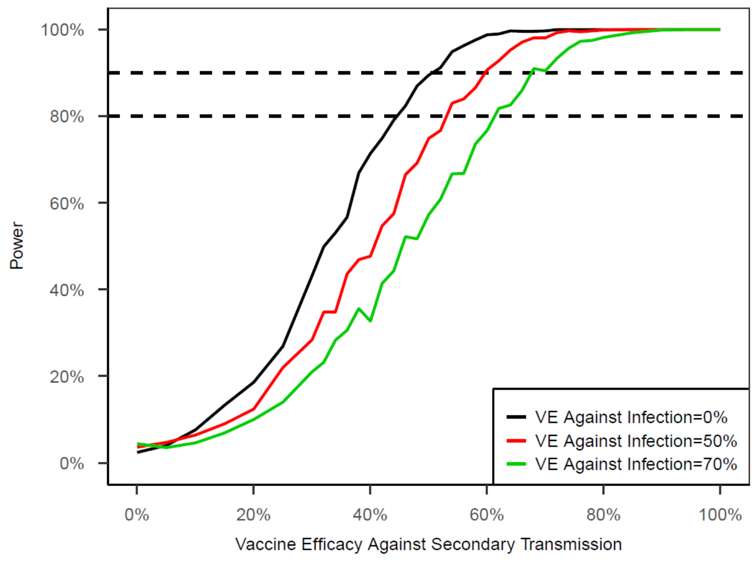

Figure 6-2 Power to detect varying vaccine efficacy against secondary transmission as a function of vaccine efficacy against SARS-CoV-2 infection. Simulation-based power calculations assume 6,000 participants in each arm and other assumptions listed in **Table 6-2**. Power based on 1-sided 0.025-level likelihood ratio test under Poisson regression model and 1,000 simulations.

Table 6-7 Number of primary endpoint events and secondary transmission events in each arm, under varying VE against SARS-CoV-2 infection and varying VE against secondary transmission, under other assumptions laid out in Table 6-2, and based on 1,000 simulations.

	**VE_S = 50%**		**VE_S = 70%**	
VE against secondary transmission	Median no. primary endpoints, Immediate vs. Delayed arms (IQR)	Median no. secondary transmissions, Immediate vs. Delayed arms (IQR)	Median no. primary endpoints, Immediate vs. Delayed arms (IQR)	Median no. secondary transmissions, Immediate vs. Delayed arms (IQR)
VE = 0%	76 (70-82) vs. 150 (142-158)	29 (25-34) vs. 59 (53-66)	45 (41-50) vs. 149 (141-158)	18 (14-21) vs. 59 (53-65)
VE = 40%	76 (70-81) vs. 150 (142-158)	18 (15-21) vs. 59 (53-64)	45 (41-50) vs. 149 (142-158)	11 (8-13) vs. 59 (53-65)
VE = 50%	76 (70-82) vs. 150 (143-159)	15 (12-17) vs. 59 (53-66)	45 (41-50) vs. 150 (142-158)	9 (7-11) vs. 59 (53-65)
VE = 60%	76 (70-82) vs. 150 (142-158)	12 (9-14) vs. 58 (52-65)	45 (41-50) vs. 151 (143-159)	7 (5-9) vs. 59 (53-65)
VE = 70%	75 (70-81) vs. 150 (142-158)	9 (7-11) vs. 58 (52-65)	46 (41-50) vs. 150 (142-159)	5 (4-7) vs. 59 (53-65)

6.3 Role of the Independent Data Monitoring Committee (IDMC)

The study IDMC will provide study oversight and ensure that the study is on-target to meeting its objectives in a timely fashion. The IDMC will receive summaries of study progress throughout the study. Given the short duration of the study and the considerable prior data establishing safety and efficacy of the vaccine, and the status of the EUA vaccine, there will be no pre-specified interim monitoring boundaries for VE or non-efficacy. The IDMC will make recommendations to protocol chairs and lead advisors. The set of metrics, operational targets for them, and potential remedial actions will be laid out in full in the IDMC Charter.

6.4 Randomization

A participant's randomization assignment will be computer generated and provided to the CRS study staff through a Web-based randomization system.

Randomization will be stratified. The randomization will be done in blocks to ensure balance among contemporaneously evaluated vaccine and control arms within levels defined by place of residence.

6.5 Statistical analyses

This section describes the final study analyses which will occur after the last participant has completed the Month 4 visit. All data from enrolled participants will be analyzed according to the initial randomization assignment regardless of how and when vaccinations are received. In the rare instance that a participant receives vaccination at the wrong time, the Statistical Analysis Plan (SAP) will address how to analyze the data. Analyses are modified intent-to-treat in that individuals who are randomized but not enrolled do not contribute data

and hence are excluded. Because of the brief length of time between randomization and enrollment—typically these will happen on the same day—very few such individuals are expected.

Analyses for primary endpoints will be performed using SAS and R. All other descriptive and inferential statistical analyses will be performed using SAS, StatXact, or R statistical software.

In general, no formal multiple comparison adjustments will be employed for multiple secondary endpoints. However, multiplicity adjustments will be made for certain immunogenicity assays, as discussed below, when the assay endpoint is viewed as a collection of hypotheses (eg, testing multiple peptide pools to determine a positive response).

6.5.1 Analysis variables

The analysis variables defined for all participants in the main study cohort consist of baseline participant characteristics, longitudinal measures of risk of infection, SARS-CoV-2 infection and seroconversion and COVID-19 disease status, and immune response biomarkers. For individuals diagnosed with SARS-CoV-2 infection, post-infection variables will include various summaries of VL, the full genome sequence of the infecting virus, and the infection status of and relationship to each of their contacts enrolled in the PCC and CACC. For contacts enrolled in the PCC and CACC, variables consist of baseline participant characteristics, SARS-CoV-2 infection and seroconversion status, risk factors around time of exposure to main study participant (if infected) and relationship to the referring main study participant.

6.5.2 Baseline comparability and comparability over study follow-up

Treatment arms will be compared with respect to baseline participant characteristics using descriptive statistics.

Given that the study is open-label, and the associated potential for differences between arms with respect to retention, compliance with study procedures, ascertainment of endpoints, and exposure to SARS-CoV-2, these measures will be compared between treatment arms over time using methods fully described in the SAP. Imbalances between arms will influence the analysis choices as described below.

6.5.3 Vaccine efficacy (VE) analyses against SARS-CoV-2 infection

The time origin for all analyses of SARS-CoV-2 infection is time of enrollment (ie, Day 0). We define the time of diagnosis of the SARS-CoV-2 infection endpoint as the collection time of the first daily swab with a positive PCR test. The time of diagnosis of lab-confirmed clinical COVID-19 disease is the first time of reported qualifying COVID-19 symptoms that occurs simultaneously or after the diagnosis of the SARS-CoV-2 infection endpoint. The time of diagnosis of asymptomatic SARS-CoV-2 infection is the collection time of the first sample with a positive PCR test result or seroconversion without any prior reporting of symptoms that led to confirmation of a COVID-19 disease endpoint. The time of diagnosis of

SARS-CoV-2 seroconversion is the collection time of the first sample seropositive for SARS-CoV-2, for participants SARS-CoV-2 seronegative at baseline. The failure times of participants never observed to be diagnosed with a given clinical endpoint will be right-censored at the date of last contact at which endpoint status was assessed or study termination.

6.5.3.1 Primary VE Analysis

For the primary analysis, Cox proportional hazards regression will be used to estimate vaccine efficacy against SARS-CoV-2 infection among baseline SARS-CoV-2 seronegative participants in the D29- set, counting only infection events diagnosed post-visit-3 (month 1), as measured by one minus the hazard ratio (HR) in the immediate vs. delayed vaccination arms. Given that the study is open-label, the primary analysis will adjust for an SAP-specified set of baseline variables, or a composite risk score based on them, that are potentially associated with exposure including demographics and behaviors captured using the risk assessment tool. Inference will be based on a 2-sided score-based CI and 2-sided score-test for H0: VE = 0% with a type 1 error rate of 0.05, although primary interest is in estimation as opposed to hypothesis testing and the study is designed to ensure that the lower limit for the CI will lie above 30% with high probability under VE > 60%.The baseline hazard function in the Cox model will be stratified by study site and randomization stratum, which improves precision of the VE estimate and accounts for the fact that SARS-CoV-2 infection incidence may vary widely across study sites. Description of the baseline variables under consideration and of the derivation of a risk score based on them will be described in the study SAP.

The potential impacts of the open-label aspect of the study design will be factored into the choice of efficacy analysis. If there is evidence of differential retention of study participants across arms, or differential adherence to study procedures affecting ascertainment of endpoints such as collection of daily swabs or participation in the university testing system, the analysis will adjust for the differential censoring distribution across arms- as well as potentially informative censoring- as described in the study SAP. At a minimum, these analyses will be performed as sensitivity analyses, complementing the primary analysis described above.

Variables captured using the risk assessment tool and potentially associated with exposure will be compared across arms over study follow-up. If there is evidence that their distributions differ between arms over time, the analysis will additionally adjust for time-dependent exposure-related variables.

The proportional hazards assumption will be evaluated using methods of Grambsch and Therneau (51).

A sensitivity analysis will be conducted to account for the possibility that the university testing programs may capture infections missed in the study swabbing; the primary analysis of VE against SARS-CoV-2 infection will be repeated using all available PCR test results from both daily swabs conducted as part of this protocol and the university testing system.

6.5.3.2 Secondary VE Analyses

The primary analysis is aimed at evaluating vaccine efficacy, i.e. the biological effect of the vaccine on SARS-CoV-2 infection. This parameter can be challenging to evaluate in any efficacy trial, given that with sufficient follow-up and heterogeneity in risk, there is culling of high-risk participants over time and the effect estimated using all follow-up is biased relative to the per-exposure reduction in risk of infection- although importantly the bias is small in the setting of low incidence with short follow-up and realistic heterogeneity assumptions (52). In secondary analyses, vaccine *effectiveness* will be evaluated- that is, the effect of the vaccine on SARS-CoV-2 infection, including both biological and behavioral effects caused by knowledge of vaccine receipt or lack of receipt. Vaccine effectiveness has public health importance, given that in public health practice individuals know whether and when they have received vaccine, and may modify their behavior given this knowledge. To evaluate vaccine effectiveness, the same primary analysis methods will be applied but without adjustment for covariates that are associated with SARS-CoV-2 exposure. Vaccine effectiveness will be evaluated against all the clinical endpoints, e.g. viral load, secondary transmission, COVID-19 disease, and seroconversion.

The primary analysis of VE against SARS-CoV-2 infection will be repeated among participants in the FAS, counting all SARS-CoV-2 infection events after Day 0; in the D29-set but including baseline seropositive participants [Secondary Objective 5]; and finally in the PP set [Secondary Objective 9]. The same methods also apply to evaluating VE against seroconversion [Secondary Objective 2] and VE against COVID-19 disease [Secondary Objective 3]. Additional secondary VE analyses will be conducted where the time of SARS-CoV-2 infection diagnosis is defined as the earliest of the first positive PCR result and the date of university positive test result.

The asymptomatic SARS-CoV-2 infection endpoint [Secondary Endpoint 8] is defined by the PCR tests of daily swabs and serologic diagnostic testing schedule at month 0, 2, 3, and 4 The occurrence of a COVID-19 disease event is a competing risk because the occurrence of COVID-19 disease precludes the possibility of occurrence of a subsequent asymptomatic SARS-CoV-2 infection event. Therefore, VE against asymptomatic SARS-CoV-2 infection will be assessed using methods accounting for COVID-19 disease as a competing risk. The SAP will provide details.

Vaccine efficacy against the infection and disease endpoints as measured by the reduction in the cumulative incidence of the endpoint by Month 4, will also be evaluated using the Nelson-Aalen estimator of cumulative incidence. This analysis does not rely on the proportional hazards assumption and has a simple interpretation that lends itself to understanding of population effects.

6.5.4 VL analyses

Primary analyses of the vaccine effect on VL will focus on the primary VL endpoint, peak log10 viral load, and will condition on primary endpoint event status. Peak log10 viral load will be estimated by the observed maximum log10 viral load measure; secondary VL analyses will employ parametric models to estimate VL at peak. The mean observed log10

viral load will be compared between the immediate and delayed vaccination arms using methods that adjust for baseline covariates and covariates at the time of SARS-CoV-2 infection diagnosis, per the study SAP. The covariate adjustment is used to control for potential post-randomization selection bias, whereby SARS-CoV-2 infected participants in the two treatment arms are not comparable in ways that are associated with the VL endpoint. Importantly, this bias occurs when there are factors that modify VE against SARS-CoV-2 infection *and* that are associated with the VL endpoint. Full details around the analysis plan will be specified in the study SAP.

Secondary analyses of vaccine effects on VL will consider a set of other summary measures of the VL curve, each of which have merits for further characterizing the vaccine effect. These include a model-based estimate of peak log10 viral load, area under the log viral load curve [AUC] calculated using the trapezoidal rule, and area under the portion of the log viral load curve over 10^5 viral copies/mL. Some mathematical modeling studies provide evidence of transmission potential for VL above this threshold (21), but there is considerable uncertainty and therefore other thresholds will be considered. Analysis methods will be the same as for the primary VL endpoint above.

In addition, the above VL endpoints will be evaluated using unconditional analyses, ie, not conditioning on SARS-CoV-2 infection status, among all enrolled participants. Participants not diagnosed with SARS-CoV-2 infection will have an endpoint value defined for analysis, typically a value of zero. The ‘transmission potential’ endpoint [Secondary endpoint 7] is one such endpoint that is defined unconditionally. The merit of unconditional analyses is that they are not subject to potential post-randomization selection bias. A disadvantage of the unconditional analyses is that they may have lower statistical power.

An additional VL endpoint that will be studied is a censored event-time variable, time to VL above 10^5 copies/mL. This endpoint is defined for all enrolled participants and will be analyzed using the same methods used to evaluate VE against SARS-CoV-2 infection, but only counting infections once VL is above 10^5 copies/mL. Additional methods of analysis that allow for the uncertainty in the level of virus that is associated with transmission will be considered.

The study SAP will detail the secondary analysis approaches for the VL endpoints.

6.5.5 Secondary transmission analyses

6.5.5.1 Identifying ‘adjudicated transmission events’

An expert adjudication committee will examine and make a primary determination to identify ‘adjudicated transmission events’ from a main study participant diagnosed with infection via the university testing system (‘index case’) to a contact in the PCC or CACC diagnosed with SARS-CoV-2 infection. Note that the close contact could also be a main study participant in theory, although this is unlikely given the relatively small proportion of each university population that will be recruited to the main study. The adjudication committee will operate under a charter that lays out a pre-specified set of criteria for defining the occurrence and direction of transmission and will be blinded to the treatment assignment of all study

participants and contacts. The endpoint definition will include criteria that need to be met regarding the temporal relationship (eg, evidence that person B's infection occurred after person A's infection, evidence of compatible incubation period), epidemiologic link (evidence of close contact during person A's infectious period), and relatedness of their SARS-CoV-2 viruses.

The adjudication committee will be provided with all relevant data from the study to deduce transmission events from potential transmission pairs. A potential transmission pair is a post-baseline SARS-CoV-2- infected main study index case and their identified contact(s) in the PCC or CACC who have positive SARS-CoV-2 test results within a prespecified period (eg, two weeks) of the index case diagnosis. This will include the type, frequency, and magnitude (eg, duration and setting) of contact between the index case and contact based on risk assessment questionnaires given to the index case and the contact; the timing of diagnosis of infection by PCR for the index case and the contact (defined as the date of specimen collection); the VL trajectories, serology, and symptomatology for the index case and the contact; and summaries of the viral sequences for the index case and the contact including the number of nucleotide differences in the sequences and any clustering and results of phylogenetic analyses (see Section 6.5.5.3). Adjudication of all potential transmission events will occur within a short time frame at the end of the study to ensure decisions are being made using the same background research on transmission and to ensure that the adjudication can take into account all SARS-CoV-2 infection events captured in the main study cohort and both contact cohorts. Primary analyses of the vaccine effect on secondary transmission will rely on the determination of this committee and use the adjudicated transmission events and outcomes in statistical models. These same primary analyses will also only include main study participants diagnosed with infection through the university testing system ('index cases'), since such a diagnosis is the trigger for contact referral. However, secondary analyses may incorporate the full set of main study participants diagnosed with infection either through university testing or through PCR testing of swabs collected through the study, where transmission events from the latter set of cases will be treated as potentially informative missing data, per methods described in the SAP.

6.5.5.2 Analyses of the vaccine effect on secondary transmission

Primary analyses of vaccine efficacy against secondary transmission will include adjudicated transmission events collected from both the PCC and CACC. Both conditional and unconditional analyses will be performed. Conditional analyses will compare the expected number of adjudicated transmission events between index cases in the immediate vs. delayed vaccination arms using Poisson regression, where goodness of fit tests will be used to determine whether an overdispersed Poisson or other alternative distributions fit the data better, per the study SAP. The SAP will specify the baseline participant characteristics and measurements available at the time of primary endpoint diagnosis that are potentially associated with secondary transmission to be included as covariates. The conditional analysis may have greater power than an unconditional analysis, e.g. under low VE against SARS-CoV-2 infection. However, it is subject to the post-randomization selection bias, as discussed in relation to the viral load endpoint. In general, it will be biased if there are factors that modify VE against SARS-CoV-2 infection that are also associated with onward transmission; or if factors associated with onward transmission are modifiers of VE against infection.

A secondary analysis of this endpoint will measure vaccine efficacy against secondary transmission as the percent reduction in the expected number of adjudicated transmission events linked to a participant in the immediate vaccination arm relative to the delayed vaccination. In contrast to the primary transmission analysis that conditions on the main study participant being infected, all main study participants will contribute to the analysis including those who do not become infected to reflect the fact that SARS-CoV-2-uninfected participants do not transmit to others and therefore have 0 transmission events. Let N(t) = number of adjudicated transmission events for a given index case by time t (time since randomization) which is assumed to follow a zero-inflated Poisson distribution with the proportion in the point mass at 0 being time-dependent. The mean of the non-zero point mass component is $\mu e^{(V\gamma)}$, where V indicates randomization assignment (1 immediate vaccination; 0 delayed vaccination). Further denote infection time as T. We can express the expected number of adjudicated transmission events by follow-up time t as

$$E(N(t)|V,T) = 0 * I(t \le T) + \mu e^{(V\gamma)} I(t > T|V)$$
$$E[N(t)|V] = E[E(N(t)|V,T)] = \mu e^{(V\gamma)} S(t|V)$$

where $S(t|V)$ is the survival distribution for infection times. The ratio of expected number of adjudicated transmission events between the two arms can then be expressed as

$$\frac{E[N(t)|V=1]}{E[N(t)|V=0]} = \frac{\mu e^{(1\gamma)} S(t|V=1)}{\mu e^{(0\gamma)} S(t|V=0)} = \frac{e^{\gamma} S(t|V=1)}{S(t|V=0)}.$$

Under the proportional hazards assumption, $\frac{S(t|V=1)}{S(t|V=0)}$ is constant, further simplifying this estimator to

$$e^{\theta} = e^{\gamma} e^{\beta},$$

where β is the log hazard ratio from a Cox proportional hazards model for infection time, θ is a measure of the vaccine's impact on total onward transmission (transmission from participants in main study) and e^{θ} is the relative reduction in the proportion of adjudicated transmission events in the immediate vaccination arm relative to the delayed vaccination arm. This is analogous to the product estimator in the proportional means model (53, 54). Note that this formulation assumes the time between detection in the index case participant and infection of a contact is ignorable. Covariate-adjusted approaches will be detailed in the SAP.

The unconditional analysis retains high power even if there are few breakthrough infections in the immediate vaccination group due to high VE against infection. It also properly reflects that a vaccine is successful at reducing secondary transmission if it either meaningfully reduces acquisition or reduces onward transmission from infected participants. Moreover, by avoiding conditioning on SARS-CoV-2 infection, it avoids the post-randomization selection bias issue.

While analyses that pool adjudicated transmission events from the PCC and CACC will produce valid tests of the null hypothesis of no vaccine effect on secondary transmission,

likelihood-based analyses that take into account the different duration of follow-up of contacts in the PCC vs. the CACC, and the potential left-censoring of contact follow-up in the CACC will be explored and may provide greater accuracy and power (38). Details of these analyses, including methods for checking the underlying assumptions and investigations of their operating characteristics using simulations of social and transmission networks, will be provided in the study SAP.

6.5.5.3 Processing and phylogenetic analysis of SARS-CoV-2 viral sequence data

A central sequencing facility will obtain whole-genome sequences from all infected participants in the main study cohort and all infected participants in the PCC and CACC, based on the nasal swab with the highest viral load (see Section 10.3.2). Details about the sequencing platform and methodology for QC processes will be included in the study SAP. This will include the creation of a global alignment, which will be used for the isolation of genes and the sequence analyses of local regions.

A first step in the analysis of the sequence data will be to determine, for each pair of sequences among infected participants or contacts at a given university, which are identical. Data from GenBank, Global Initiative on Sharing Avian Influenza Data (GSAID), and other public databases suggest that we can expect limited variability in genetic sequences, especially those from a single common ancestor and constrained within a single university community over a 4-month time period. Consensus-based whole genomes that differ by more than two nucleotides can reasonably be ruled out as being transmission events.

A second step will be to conduct distance-based analyses of the sequences, including phylogenetic analysis, to identify potential lineages of infecting viral strains, infection clusters, and potentially linked transmissions. Phylogenetic analyses will also include ‘background’ SARS-CoV-2 sequences with geographical or temporal relevance from publicly available databases, such as GenBank and GISAID. The background sequences will help root the tree and give the tree structure and may lend insight into the identification of clusters and into potential transmission events during the adjudication process.

Details about the phylogenetic and other sequence analyses, including the tree-building methods and evolutionary models to be used, as well as approaches to incorporating data from multiple reads, will be available in the study SAP.

6.5.6 Immunogenicity analyses

6.5.6.1 Vaccine immunogenicity

Immunogenicity will be characterized for the immediate vaccination group only, using data from the Case-Cohort Immunogenicity Set (ccIS). Data from quantitative immunogenicity assays will be summarized using positive response rates and geometric means with 95% CIs, for each timepoint for which an assessment is performed. Data from qualitative (ie, yielding a positive or negative result) assays will be summarized by tabulating the frequency of positive responses for each assay at each timepoint that an assessment is performed. Analyses focus

on the Month 2 time point, the Day 1 time point, and the change in marker response between the two time points. The analyses will evaluate immunogenicity in all immediate vaccination arm participants and separately in subgroups defined by baseline SARS-CoV-2 serostatus. The SAP will describe the complete set of immunogenicity analyses.

6.5.6.2 Immune response marker correlates of risk

A separate Trial Marker SAP will describe the statistical methods and data analysis implementations for assessing immune response markers as correlates of the SARS-CoV-2 infection and peak log viral load study endpoints, as well as various types of correlates/surrogates of protection.

An unblinded statistical analysis by treatment assignment of a primary immunogenicity endpoint may be performed when all participants have completed the Month 2 visit and data are available for analysis from at least 80% of these participants. Similarly, an unblinded statistical analysis by treatment assignment of a secondary or exploratory immunogenicity endpoint may be performed when all participants have completed the corresponding immunogenicity visit and data are available for analysis from at least 80% of these participants. The CoVPN Laboratory Center will review the analysis report prior to distribution to the protocol chairs, NIAID, study product developer, and other key CoVPN members and investigators. Reports for distribution or presentation should use PubIDs and not PTIDs for individual responses. Distribution of reports will be limited to those with a need to know for the purpose of informing future trial-related decisions. The CoVPN leadership must approve any other requests for CoVPN immunogenicity analyses prior to the end of the scheduled follow-up visits.

7 Selection and withdrawal of participants

Participants will be generally healthy adults who comprehend the purpose of the study and have provided written informed consent. Volunteers will be recruited and screened; those determined to be eligible, based on the inclusion and exclusion criteria, will be enrolled in the study. Final eligibility determination will depend on information available at the time of enrollment, medical history questionnaire, physical examinations, and answers to self-administered and/or interview questions.

Investigators should always use good clinical judgment in considering a volunteer's overall fitness for trial participation. Some volunteers may not be appropriate for enrollment even if they meet all inclusion/exclusion criteria. Medical, psychiatric, occupational, or other conditions may make evaluation of safety and/or immunogenicity difficult, and some volunteers may be poor candidates for retention.

Determination of eligibility, taking into account all inclusion and exclusion criteria, must be made within 56 days prior to enrollment unless otherwise noted below.

7.1 Inclusion criteria for Main Cohort

General and Demographic Criteria

1. **Age** of 18 through 26 years.
2. **Enrollment in a participating university** and willingness to be followed for the planned duration of the study.
3. **Agrees to allow study staff to access university SARS-CoV-2 testing** data and outcomes.
4. Ability and willingness to provide **informed consent.**
5. **Assessment of understanding**: volunteer demonstrates understanding of this study; completes a questionnaire prior to first vaccination with demonstration of understanding of all questionnaire items answered incorrectly.
6. **Willing to defer SARS-CoV-2 licensed or EUA vaccine** until visit 6 (Month 4)
7. **Agrees not to enroll in another study** of an investigational research agent until the end of the study.
8. **Access to device and internet** for Telehealth visits.

7.2 Exclusion criteria for Main Cohort

General

1. **Acutely ill or febrile 72 hours prior to or at screening**. Fever is defined as a body temperature ≥ 38.0°C/100.4°F. Volunteers meeting this criterion may be rescheduled within the relevant window periods. Afebrile participants with minor illnesses can be enrolled at the discretion of the investigator.

2. **Blood products,** systemic immunoglobulins, or monoclonal antibodies (including against SARS-CoV-2) received within 90 days before first vaccination.

3. **Investigational research agents** received within 30 days before first vaccination.

4. **Self-reported known history of SARS-CoV-2 infection.**

Vaccines and other Injections

5. **Prior administration of a coronavirus** (SARS-CoV-2, SARS-CoV, MERS-CoV) vaccine or current/planned simultaneous participation in another interventional study to prevent or treat COVID-19.

Immune System

6. **Immunosuppressive medications** received within 168 days before first vaccination (not exclusionary: [1] corticosteroid nasal spray; [2] inhaled corticosteroids; [3] topical corticosteroids for mild, uncomplicated dermatologic condition; or [4] a single course of oral/parenteral prednisone or equivalent at doses ≤ 60 mg/day and length of therapy < 11 days with completion at least 30 days prior to enrollment).

Clinically significant medical conditions

7. **Clinically significant medical condition**, physical examination findings, or past medical history with clinically significant implications for current health. A clinically significant condition or process includes but is not limited to:

   - A process that would affect the immune response (well-controlled human immunodeficiency virus infection is allowed), or

   - Any condition specifically listed among the exclusion criteria below.

8. **Any medical, psychiatric, occupational, or other condition** that, in the judgment of the investigator, would interfere with, or serve as a contraindication to, protocol adherence, assessment of safety or reactogenicity, or a volunteer's ability to give informed consent.

9. **Bleeding disorder** (eg, factor deficiency, coagulopathy, or platelet disorder requiring special precautions).

10. **Asplenia**: any condition resulting in the absence of a functional spleen.

11. **History of angioedema or anaphylaxis**, including to vaccines or vaccine components (not exclusionary: angioedema or anaphylaxis with known trigger and no episodes within five years.).

12. **History of generalized urticaria** within past five years.

7.3 Inclusion criteria for Prospective Close Contact (PCC) cohort

1. **Age** of 18 years or older, at the time of signing the informed consent.

2. Willing and able to provide **informed consent**.

3. Expected to be in **frequent close physical proximity with Main Cohort participant** during the study.

4. **Participating in their university SARS-CoV-2 testing system** and willing to share results.

5. **Access to device and internet** for Telehealth visits.

7.4 Inclusion criteria for Case-ascertained Close Contact (CACC) cohort

1. **Age** of 18 years or older, at the time of signing the informed consent.

2. Willing and able to provide **informed consent**.

3. **Access to device and internet** for Telehealth visits.

4. Willing to share **results of SARS-CoV-2 testing**.

5. Had **close contact with Main Cohort participant with known PCR-confirmed SARS-CoV-2 infection** (eg, index case). Close contacts will have had exposure to the index participant, generally within 72 hours of an index diagnosis, and may include individuals that meet any of the following guidelines:

    - Prolonged close physical proximity with Main Cohort participant within a residence/vehicle/enclosed space without maintaining social distance,

    - Medical staff, first responders, or other care persons who cared for the index case without appropriate personal protective equipment.

    Further information on the definition of close contact can be found in the CoVPN 3006 Study Specific Procedures (SSP).

8 Study product

The protocol schema is shown in Table 1-1.

8.1 Vaccine regimen

Group 1

Treatment 1 (T1): Moderna COVID-19 Vaccine in 100 mcg dose given as 0.5 ml IM into the deltoid muscle on Day 1 and Day 29.

Group 2

Treatment 2 (T2): Moderna COVID-19 Vaccine in 100 mcg dose given as 0.5 ml IM into the deltoid muscle on Day 113 and Day 141.

8.2 Storage, handling, preparation, and administration

Vaccination providers will refer to EUA vaccine instructions accessed and updated at https://www.modernatx.com/covid19vaccine-eua/.

8.3 Acquisition of Moderna COVID-19 Vaccine

Moderna COVID-19 Vaccine will be provided by Moderna, Inc. through the US Government COVID-19 Vaccine Response.

9 Clinical procedures

Procedures are in place so that study visits may be conducted remotely, such as via phone, text message, email, or other electronic means, in lieu of, or in combination with, in-person visits at the clinical research site. Furthermore, some visit procedures may be conducted outside the CRS (see CoVPN 3006 SSP for additional details).

The schedules of clinical procedures are shown in Appendix D, Appendix E, Appendix F and Appendix G.

9.1 Informed consent

Informed consent is the process of working with participants so that they fully understand what will and may happen to them while participating in a research study. The CoVPN informed consent form documents that a participant (1) has been informed about the potential risks, benefits, and alternatives to participation, and (2) is willing to participate in a CoVPN study. Informed consent encompasses all written, verbal, and electronic study information CRS staff provide to the participant, before and during the trial. CRS staff will obtain informed consent of participants according to the CRS's SOP on the informed consent process.

If any new information is learned that might affect the participants' decisions to stay in the trial, this information will be shared with trial participants. If necessary, participants will be asked to provide revised informed consent forms.

9.1.1 Screening consent form

Without a general screening consent, screening for a specific study cannot take place until the site receives protocol registration from NIAID or its designee.

Some CRSs have approval from their IRB/EC and any applicable RE to use a general screening consent form that allows screening for an unspecified SARS-CoV-2 vaccine trial. In this way, CRS staff can continually screen potential participants and, when needed, proceed quickly to obtain protocol-specific enrollment consent. Sites conducting general screening or prescreening approved by their IRB/EC and any applicable RE may use the results from this screening to determine eligibility for this protocol, provided the tests are conducted within the time periods specified in the eligibility criteria.

9.1.2 Protocol-specific consent forms

The protocol-specific consent forms describe the study products to be used and all aspects of protocol participation, including screening and enrollment procedures. A sample protocol-specific consent form for the Main cohort is located in Appendix A. A sample protocol-specific consent form for the PCC cohort is located in Appendix B. A sample protocol-specific consent form for the CACC cohort is located in Appendix C.

Each CRS is responsible for developing a protocol-specific consent form(s) for local use, based on the sample protocol-specific consent forms. The consent form(s) must be developed in accordance with requirements of the following:

- CRS's IRB/EC and any applicable REs
- CRS's institution
- Elements of informed consent as described in Title 45, CFR Part 46 and Title 21 CFR, Part 50, and in ICH E6(R2), Good Clinical Practice: Consolidated Guidance 4.8

Study sites are strongly encouraged to have local community representatives from the target population review their sites-specific consent forms. This review should include, but should not be limited to, issues of cultural competence, local language considerations, and the level of understandability.

The sample informed consent form(s) include(s) instructions for developing specific content.

9.1.3 Assessment of Understanding

Study staff are responsible for ensuring that participants fully understand the study before enrolling them. This process involves reviewing the informed consent form with the participant, allowing time for the participant to reflect on the procedures and issues presented, and answering all questions completely.

An Assessment of Understanding is used to document the participant's understanding of key concepts in this study. Participants must demonstrate understanding of all questions answered incorrectly. This process and the participant's understanding of the key concepts is to be recorded in the eConsent.

9.2 Pre-enrollment procedures for Main Cohort, Immediate and Delayed Vaccination Groups

Screening may occur over the course of several contacts/visits, up to and including before randomization on Day 1. All inclusion and exclusion criteria must be assessed within 56 days before enrollment, unless otherwise specified in the eligibility criteria (or below in this section). Screening results will be reviewed with the volunteer.

After the appropriate informed consent has been obtained and before enrollment, the following procedures are performed:

- Participant will download the electronic diary (eDiary) application to their smartphone or tablet and complete an optional application training. Participant will use eDiary to enter data. Data collection will include:
  - Medical history questionnaire

  - Volunteer demographics in compliance with the NIH Policy on Reporting Race and Ethnicity Data: Subjects in Clinical Research, Aug. 8, 2001 (available at http://grants.nih.gov/grants/guide/notice-files/NOT-OD-01-053.html)
  - Baseline SARS-CoV-2 infection risk information

- Discussion of the eDiary entries done by the participant with the site staff
- Review of materials for possible self-collection of blood by the participant

9.3 Enrollment and vaccination visits for Main Cohort, Immediate Vaccination Group

Once a volunteer has consented to trial participation and is found to meet all eligibility criteria (see Sections 7.1 and 7.2), the CRS requests the randomization assignment via a Web-based randomization system. **Enrollment is simultaneous with randomization**. In general, the time interval between randomization and first vaccination for the Immediate Vaccination Group should not exceed 4 working days. However, circumstances may require a participant's visit to be changed. This may exceed the 4-day randomization time limit. Clinically important lab results will be reviewed with the participant at any visit, as needed.

At all vaccination visits, the following procedures are performed **before vaccination**:

- COVID-19 symptom check (including temperature)
- Specimen collection (see Appendix D)

Following completion of all procedures in the preceding list, and if results indicate that vaccination may proceed, vaccination is prepared and administered per Moderna EUA Fact Sheet and Full Prescribing Information for Vaccination Providers that can be accessed at https://www.modernatx.com/covid19vaccine-eua/).

Additionally, the participants will be:

- Reminded to enter information in eDiary throughout the study
- Provided with a supply of nasal swabs and shown how to swab both nostrils

9.4 Follow-up procedures for Main Cohort, Immediate Vaccination Group

The following procedures are performed **during follow-up per schedule in** Appendix D:

- Participants reminded to enter information in eDiary throughout the study (see Section 9.15)

- Nasal swab collection by participant
- Blood collection by CRS staff, phlebotomist, or participant

9.5 Enrollment (visit 2) and visits 3-5 for Main Cohort, Delayed Vaccination Group

Once a volunteer has consented to trial participation and is found to meet all eligibility criteria (see Sections 7.1 and 7.2), the CRS requests the randomization assignment via a Web-based randomization system. **Enrollment is simultaneous with randomization**. Clinically important lab results will be reviewed with the participant at any visit, as needed.

Before leaving the clinic, the participants will be:

- Reminded to enter information in eDiary throughout the study
- Provided with a supply of nasal swabs and shown how to swab both nostrils

At visits 2 through 5, the following procedures are performed **per schedule in** Appendix E:

- eDiary entries by participant (see Section 9.15)
- Nasal swab collection by participant
- Blood collection by CRS staff, phlebotomist, or participant

9.6 Vaccination visits for Main Cohort, Delayed Vaccination Group

At vaccination visits, the following procedures are performed **before vaccination**:

- COVID-19 symptom check (including temperature)
- Specimen collection (**only at first vaccination** visit, see Appendix E)

Following completion of all procedures in the preceding list, and if results indicate that vaccination may proceed, vaccination is prepared and administered, per Moderna EUA Fact Sheet and Full Prescribing Information for Vaccination Providers that can be accessed at https://www.modernatx.com/covid19vaccine-eua/).

9.7 Procedures for Main Cohort participant diagnosed with a SARS-CoV-2 infection during the study

If a participant is diagnosed with a SARS-CoV-2 infection during the course of the study, CDC guidelines on administration of vaccination will be followed.

Main Cohort participants who are clinically diagnosed with SARS-CoV-2 infection are referred to as clinically diagnosed index cases. They will be instructed to quarantine per local guidelines and the following procedures will be performed:

- eDiary collection of daily symptoms of COVID-19 and assessment of whether clinical care was sought or hospitalization occurred (via self-report and/or request for medical records) through an eDiary for a period of 14 days or until symptoms have resolved, whichever is longer
- Daily nasal swabbing continued per schedule of procedures (see Appendix D)
- Blood collection by phlebotomist or participant for SARS-CoV-2 clinical serology
- Close contacts identification (see Section 9.9)

Following resolution of the quarantine period, participants will continue scheduled study visits. Follow-up duration for participants diagnosed with moderate or severe COVID-19 disease may be adjusted in consultation with the CRS (eg, to avoid interference with participant initiation of SARS-CoV-2 treatment).

9.8 Enrollment and follow-up procedures for Prospective Close Contact (PCC) cohort

Upon enrollment, participants from the Main Cohort will be asked to invite individuals with whom they will be in frequent close physical proximity during the study to join the PCC cohort (See Section 7.3 and CoVPN 3006 SSP for additional information on PCC cohort). New PCC contacts may also be added during the study.

Prospective close contacts will be given referral codes and information about how to enroll in the study as part of the PCC cohort. If they choose to enroll and sign an informed consent, they will be asked to:

- provide access to their routine SARS-CoV-2 testing reports
- enter data weekly through eDiary (see Section 9.15)

If a PCC cohort participant or the Main Cohort participant to whom he/she is connected is diagnosed with a SARS-CoV-2 infection during the course of the study, the PCC cohort participant will be instructed to perform the following procedures (see Appendix F):

- eDiary collection of daily symptoms of COVID-19 and assessment of whether clinical care was sought or hospitalization occurred (via self-report and/or request for medical records) through an eDiary for a period of 14 days or until symptoms have resolved, whichever is longer
- provide nasal swab for 14 days

- self-collect blood samples on day 1 and day 29, relative to the notification of infection

For Main Cohort participants who are also PCC cohort participants related to an index case in another Main cohort participant, the following procedures in addition to those listed in Appendix D may be performed:

- one additional blood collection (self-collection) as soon as possible following the report of the index case
- eDiary collection of information regarding exposure to the Index case
- eDiary collection of daily symptoms of COVID-19 and assessment of whether clinical care was sought or hospitalization occurred (via self-report and/or request for medical records) through an eDiary for a period of 14 days or until symptoms have resolved, whichever is longer

See CoVPN 3006 SSP for additional information on PCC cohort.

9.9 Enrollment and Follow-up procedures for Case-Ascertained Close Contact (CACC) cohort

Participants from the Main Cohort who are clinically diagnosed with SARS-CoV-2 (referred to as the Index case) during the study will be asked to contact any individuals with whom they have been in close contact within about 72 hours of the date of the SARS-CoV-2 diagnosis test. See Section 7.4 and CoVPN 3006 SSP for additional information on case-ascertained close contacts.

Potential CACC cohort participants will be given referral codes and information about how to enroll in the study as part of the CACC cohort, if they choose to, and sign an informed consent.

Once a volunteer has consented to be a part of CACC cohort, the following procedures will be performed (see Appendix G):

- eDiary collection of information regarding exposure to the Index case
- eDiary collection of daily symptoms of COVID-19 and assessment of whether clinical care was sought or hospitalization occurred (via self-report and/or request for medical records) through an eDiary for a period of 14 days or until symptoms have resolved, whichever is longer.
- Outcomes of SARS-CoV-2 testing performed by the university or other facilities
- Nasal swab self-collection from day 1-14
- Blood collection by participant for SARS-CoV-2 clinical serology at day 3 and at day 29

If a CACC participant is diagnosed with a SARS-CoV-2 infection during the course of the study, they will be instructed to track daily symptoms of COVID-19 through an eDiary for a period of 14 days or until symptoms have resolved, whichever is longer.

For Main Cohort participants who are also CACC cohort participants, the following procedures in addition to those listed in Appendix D may be performed:

- one additional blood collection (self-collection) as soon as possible
- eDiary collection of information regarding exposure to the Index case
- eDiary collection of daily symptoms of COVID-19 and assessment of whether clinical care was sought or hospitalization (via self-report and/or request for medical records) occurred through an eDiary for a period of 14 days or until symptoms have resolved, whichever is longer.

9.10 Visit windows and missed visits

Visit windows are included in Appendix H. The procedures for documenting missed visits and out of window visits are described in CoVPN 3006 SSP.

9.11 Early termination visit

In the event of early participant termination, site staff should consider if final specimen collection is appropriate.

9.12 Pregnancy for Main Cohort

For Main cohort participants who have been vaccinated, pregnancies and pregnancy outcomes will be reported via the FDA/CDC Vaccine Adverse Event Reporting System (VAERS) (see Section 11.1).

9.13 SARS-CoV-2 monitoring by universities for Main Cohort

Participating universities have SARS-CoV-2 monitoring plans in place. The monitoring will typically provide approximately twice weekly SARS-CoV-2 testing for enrolled Main Cohort participants. For any positive test result, the institution will inform the student (initiating quarantine), will provide the SARS-CoV-2 test results to the students, and will carry out contact tracing. Participants will be instructed to report positive test results via the eDiary.

9.14 COVID-19 symptom surveillance for Main Cohort

Participants will be monitored for symptoms of COVID-19 throughout the course of the study via a weekly eDiary. These symptoms will be used to establish whether a participant meets criteria for COVID-19 disease as defined by Secondary Endpoint 4. Due to the overlap of symptoms that can result from vaccination and symptoms of COVID-19, monitoring will begin 7 days following the first study product administration. Symptom monitoring will be paused 7 days following the second product administration. If a participant has symptoms consistent with COVID-19, they will be encouraged to seek testing and clinical care through their local testing and care facilities. Results of testing will be reported through the eDiary and participants who have positive test results will follow procedures described in Section 9.7.

9.15 Use of electronic Diary (eDiary) for Clinical Outcomes Assessments

At the time of consent, participants must confirm they will be willing to complete an eDiary collection of study data (see below for details of data to be collected).

The eDiary will be completed using an application downloaded to their smartphone or tablet. This study will utilize the Medidata Patient Cloud Application as the eDiary software. This application allows for real-time data collection directly from participants.

After participants have signed the informed consent, all participants will be instructed to download the eDiary application on their personal smartphone or tablet. The eDiary application has a self-training component, enabling immediate use. The site staff will perform any retraining as necessary.

For Main Cohort participants,

Study participants will record data in the eDiary throughout the study as described in Appendix D and Appendix E, following the prompts provided for data collection. eDiary data collection areas may include:

- Medical history questionnaire
- Volunteer demographics
- Risk assessment for SARS-CoV-2 infection (see Appendix D and Appendix E)
- Surveillance for symptoms of COVID-19 (see Section 9.14)
- Self-collection of samples (eg, nasal swabs) (see Appendix D and Appendix E)
- Outcomes of SARS-CoV-2 testing performed by the University or other facilities (see Appendix D and Appendix E)

- COVID-19 symptom tracking upon incident diagnosis of SARS-CoV-2 infection, including assessments of whether clinical care was sought or hospitalization occurred via self-report and/or request for medical records (see Section 9.7)

Additionally, participants will be reminded through the eDiary of the need to continue practices for reducing acquisition and transmission of SARS-Cov-2 regardless of their vaccination status.

For Prospective Close Contact (PCC) cohort participants,

PCC participants record data in the eDiary as described in Appendix F, following the prompts provided for data collection. eDiary data collection areas may include:

- Collection of demographic information
- Collection of information regarding exposure to the main study participant
- Outcomes of SARS-CoV-2 testing performed by the university or other facilities
- Self-collection of samples (eg, nasal swabs) (see Appendix F)
- COVID-19 symptom tracking upon incident diagnosis of SARS-CoV-2 infection in PCC or main study participant, including assessments of whether clinical care was sought or hospitalization occurred via self-report and/or request for medical records

For Case-Ascertained Close Contact (CACC) cohort participants,

CACC participants will record data in the eDiary as described in Appendix G, following the prompts provided for data collection. eDiary data collection areas may include:

- Collection of demographic information
- Collection of information regarding exposure to the Index case
- Outcomes of SARS-CoV-2 testing performed by the university or other facilities
- COVID-19 symptom tracking, including assessments of whether clinical care was sought or hospitalization occurred via self-report and/or request for medical records
- Self-collection of samples (eg, nasal swabs) (see Appendix G)

10 Laboratory

10.1 CRS laboratory procedures

The CoVPN 3006 SSP and other study materials provide further guidelines for operational issues concerning the clinics and laboratories. These documents include special considerations for phlebotomy and guidelines for general specimen collection, specimen labeling and specimen processing.

Tube types for blood collection are specified in CoVPN 3006 SSP.

Of note, all assays described below are performed as research assays to evaluate the ability of the SARS-CoV-2 virus to induce immune responses in the context of the participants' genetic background and are not approved for use in medical care. Results from these assays are not made available to participants or medical professionals to guide treatment decisions.

10.2 Total blood volume

Required blood collections per visit are shown in Appendix D, Appendix E, Appendix F and Appendix G. The total blood volume drawn for each participant will not exceed 500 mL in any 56-day (8-week) period.

10.3 Endpoint assays

10.3.1 SARS-CoV-2 PCR

Real-time reverse transcription polymerase chain reaction (RT-PCR) assays to detect SARS-CoV-2 will be run on nasal samples collected from study participants. After RNA extraction, specimens will be analyzed by a laboratory developed test using the World Health Organization's E/RdRp primer set, the N1/N2 primer set from the Centers for Disease Control and Prevention, or tests from Hologic (Panther Fusion), DiaSorin (Simplexa), or Roche (cobas). Novel methods to detect SARS-CoV-2 may also be used if applicable.

10.3.2 SARS-CoV-2 viral sequencing

Genome sequencing of SARS-CoV-2 virus may be performed on nasal samples from study participants using a metagenomic approach. RNA from positive specimens is converted to a cDNA library by reverse transcription and the library is sequenced. Resulting consensus sequences are assembled against a SARS-CoV-2 reference genome.

10.3.3 Neutralizing antibody assay (nAb)

SARS-CoV-2–specific nAb assays may be performed on serum samples from study participants. The assay will test neutralization of S-pseudotyped and/or full-genome

recombinant viruses as measured by a reduction in luciferase (Luc) reported gene expression after infection in ACE-2 positive cells.

10.3.4 Binding antibody multiplex assay (BAMA)

SARS-CoV-2 specific total IgG and IgM binding antibodies may be assessed in serum samples. In addition, SARS-CoV-2–specific serum IgA and IgG subclass (IgG1, IgG2, IgG3, and IgG4) antibodies may also be assessed. Epitope specificity by ACE-2 blocking assay and responses to endemic coronavirus proteins may also be assessed.

10.3.5 Antibody avidity (BioLayer Interferometry and BAMA avidity index)

SARS-CoV-2-specific polyclonal serum antibody and IgG subclass avidity may be measured using BAMA with the addition of a dissociation step to calculate the antibody avidity index (BAMA-AI). BLI and/or SPR technologies may also be used to measure antibody avidity.

10.3.6 Fc-Mediated Antibody Function

Additional assays to measure SARS-CoV-2-specific antibody Fc functions may be performed (ie, antibody dependent cellular cytotoxicity [ADCC], antibody-dependent cellular phagocytosis [ADCP], antibody-dependent neutrophil phagocytosis [ADNP], binding antibody FcR array, and S-protein-Expressing Cell Antibody Binding Assay [SECABA]).

10.4 Lab assay portfolio

Additional assays may be performed per the Laboratory Center assay portfolio, which includes immune assessments such as those for cellular, humoral, and innate immune responses, and host genetics. The assay portfolio will be updated periodically to include new assays and adjust qualification levels of existing assays.

10.5 Exploratory studies

Samples may be used for other testing and research related to furthering the understanding of SARS-CoV-2 immunology, virology, or vaccines. In addition, cryopreserved samples may be used to perform additional assays to support standardization and validation of existing or newly developed methods.

10.6 Specimen storage and other use of specimens

The CoVPN stores specimens from all study participants indefinitely, unless a participant requests that specimens be destroyed or if required by IRB/EC, or RE.

Other use of specimens is defined as studies not covered by the protocol or the informed consent forms for the study (see Appendix A, Appendix B, and Appendix C).

This research may relate to SARS-CoV-2, vaccines, coronaviruses, the immune system, and other diseases. This could include genetic testing and, potentially, genome-wide studies. This research is done only to the extent authorized in each study site's informed consent form, or as otherwise authorized under applicable law. Other research on specimens ("other use") will occur only after review and approval by the CoVPN, the IRB/EC of the researcher requesting the specimens, and the IRBs/ECs/REs of the CRSs if required.

As part of consenting for the study, participants document their initial decision to allow or not allow their specimens to be used in other research, and they may change their decision at any time. The participant's initial decision about other use of their specimens, and any later change to that decision, is recorded by their CRS in a Web-based tool that documents their current decisions for other use of their specimens. The CoVPN will only allow other research to be done on specimens from participants who allow such use.

CRSs must notify CoVPN Regulatory Affairs if institutional or local governmental requirements pose a conflict with or impose restrictions on specimen storage or other use of specimens.

10.7 Biohazard containment

The transmission of blood-borne pathogens can occur through contact with contaminated needles, blood, and blood products, and the transmission of SARS-CoV-2 and other respiratory pathogens may occur through contact with contaminated respiratory droplets, aerosols, and other biological materials. Appropriate precautions will be employed by all personnel in the collection, shipping, and handling of all specimens for this study, as currently recommended by local health authorities, the CDC, the NIH, or other applicable agencies.

All dangerous goods materials, including Biological Substances, Category A or Category B, must be transported according to instructions detailed in the International Air Transport Association Dangerous Goods Regulations.

11 Safety Reporting

11.1 FDA/CDC Vaccine Adverse Event Reporting System (VAERS)

The Vaccine Adverse Event Reporting System (VAERS) is co-managed by the U.S. CDC and FDA and serves as the reporting mechanism for adverse events occurring from licensed and EUA vaccines, including the Moderna COVID-19 vaccine being used in this trial. VAERS accepts and analyzes reports of adverse events (AEs) after a person has received a vaccination. It is accessed at https://vaers.hhs.gov/reportevent.html.

Risks associated with receipt of the Moderna COVID-19 Vaccine and AE reporting requirements are described in the "Fact Sheet For Healthcare Providers Administering Vaccine (Vaccination Providers) Emergency Use Authorization (EUA) Of The Moderna COVID-19 Vaccine To Prevent Coronavirus Disease 2019 (COVID-19)", available at https://www.modernatx.com/covid19vaccine-eua/.

All vaccination providers, including those at the CRS, are required to report to VAERS the following adverse events after COVID-19 vaccination, under Emergency Use Authorization (EUA), and other adverse events if later revised by CDC |(https://www.cdc.gov/vaccines/covid-19/vaccination-provider-support.html and https://vaers.hhs.gov/faq.html):

- Vaccine administration errors, whether or not associated with an adverse event (AE)
- Serious AEs regardless of causality. Serious AEs per FDA are defined as:
  - Death;
  - A life-threatening AE;
  - Inpatient hospitalization or prolongation of existing hospitalization;
  - A persistent or significant incapacity or substantial disruption of the ability to conduct normal life functions;
  - A congenital anomaly/birth defect;
  - An important medical event that based on appropriate medical judgement may jeopardize the individual and may require medical or surgical intervention to prevent one of the outcomes listed above.
- Cases of Multisystem Inflammatory Syndrome
- Cases of COVID-19 that result in hospitalization or death

CRS vaccination providers are encouraged to report to VAERS any additional clinically significant AEs following vaccination, even if they are not sure if vaccination caused the event; and are to report any additional select AEs and/or any revised safety reporting

requirements per FDA's conditions of authorized use of vaccine(s) throughout the duration of any COVID-19 Vaccine being authorized under an EUA.

CRS vaccination providers will complete and submit reports to VAERS online at https://vaers.hhs.gov/reportevent.html. Further assistance with reporting to VAERS is accessed by calling 1-800-822-7967. The reports should include the words "Moderna COVID- 19 Vaccine EUA" in the description section of the report.

As indicated in the "Fact Sheet For Healthcare Providers Administering Vaccine (Vaccination Providers) Emergency Use Authorization (EUA) Of The Moderna COVID-19 Vaccine To Prevent Coronavirus Disease 2019 (COVID-19)", vaccination providers (including those at the CRS) are encouraged, to the extent feasible, to report adverse events to Moderna.

11.2 Reporting safety events to v-safe

An additional source of VAERS reports will be through a program administered by the CDC known as v-safe (https://www.cdc.gov/coronavirus/2019-ncov/vaccines/safety/vsafe.html). V-safe is a smartphone-based opt-in program that uses text messaging and web surveys from CDC to check in with vaccine recipients for health problems following COVID-19 vaccination. The system also will provide telephone follow-up to anyone who reports medically significant (important) adverse events. Responses indicating missed work, inability to do normal daily activities, or that the recipient received care from a doctor or other healthcare professional will trigger the VAERS Call Center to reach out to the participant and collect information for a VAERS report, if appropriate. CRS vaccination providers will encourage the study participants receiving the Moderna COVID-19 vaccine to use v-safe.

12 Protocol conduct

This protocol and all actions and activities connected with it will be conducted in compliance with the principles of GCP (ICH E6 (R2)), and according to NIAID and CoVPN policies and procedures as specified in the CoVPN 3006 SSP, NIAID Clinical Research Policies and Standard Procedures Documents including procedures for the following:

- Protocol registration, activation, and implementation;
- Informed consent, screening, and enrollment;
- Study participant reimbursement;
- Clinical and safety assessments;
- Safety monitoring and reporting;
- Data collection, documentation, transfer, and storage;
- Participant confidentiality;
- Study follow-up and close-out;
- Unblinding of staff and participants;
- Quality control;
- Protocol monitoring and compliance;
- Specimen collection, processing, and analysis;
- Exploratory and ancillary studies and sub-studies, and
- Destruction of specimens.

Any policies or procedures that vary from NIAID and CoVPN standards or require additional instructions (eg, instructions for randomization specific to this study) will be described in the CoVPN 3006 SSP.

12.1 Study termination

NIAID reserves the right to terminate or curtail a clinical study for any reason, including but not limited to the following (reference: https://grants.nih.gov/grants/guide/rfa-files/RFA-AI-12-012.html):

- risk to subject safety
- the scientific question is no longer relevant or the objectives will not be met (ie, slow accrual)
- failure to comply with GCP, U.S. Federal regulations, or Terms and Conditions of Award
- occurrence of unforeseen drug safety issues or data from preclinical studies indicate a presence of unanticipated toxicity
- risks that cannot be adequately quantified
- ethical concerns raised by the local community or local medical care/health care authorities
- failure to remedy deficiencies identified through site monitoring
- substandard data
- reaching a major study endpoint substantially before schedule with persuasive statistical significance.

This study may also be terminated early by the determination of the FDA, the United States Department of Health and Human Services Office for Human Research Protections (OHRP). In addition, the conduct of this study at an individual CRS may be terminated by the determination of the IRB/EC and any applicable RE.

12.2 Emergency communication with study participants

As in all clinical research, this study may generate a need to reach participants quickly to avoid imminent harm, or to report study findings that may otherwise concern their health or welfare and their willingness to remain on study.

When such communication is needed, the CRS will request that its IRB/EC and any applicable RE expedite review of the message. If this review cannot be completed in a timeframe consistent with the urgency of the required communication, the site can contact the participant without IRB/EC approval if such communication is necessary to avoid imminent harm to the study participant. The CRS must notify the IRB/EC and any applicable RE of the matter as soon as possible.

13 Version history

The Protocol Team may modify the original version of the protocol. Modifications are made to CoVPN protocols via clarification memos, letters of amendment, or full protocol amendments.

The version history of, and modifications to, Protocol CoVPN 3006 are described below.

Protocol history and modifications

Date: February 17, 2021

Protocol version:1.0
Protocol modification: Not applicable

Original protocol

14 Document references (other than literature citations)

Other documents referred to in this protocol, and containing information relevant to the conduct of this study, include:

- Assessment of Understanding. Accessible through the CoVPN protocol-specific website.
- CDC COVID-19 Vaccination Program Provider Requirements and Support. Available at https://www.cdc.gov/vaccines/covid-19/vaccination-provider-support.html
- CoVPN Certificate of Confidentiality. Accessible through the CoVPN website.
- CoVPN 3006 Study Specific Procedures. Accessible through the CoVPN protocol-specific website.
- Dangerous Goods Regulations (updated annually), International Air Transport Association. Available for purchase at https://www.iata.org/publications/dgr/Pages/index.aspx
- International Council on Harmonisation of Technical Requirements for Pharmaceuticals for Human Use (ICH) E6 (R2), Guideline for Good Clinical Practice: Section 4.8, Informed consent of trial subjects. Available at http://www.ich.org/page/efficacy-guidelines
- Laboratory Center assay portfolio
- Moderna COVID-19 Vaccine EUA Fact Sheet and Full PI for Vaccination Providers. Available at https://www.modernatx.com/covid19vaccine-eua/.
- Participants' Bill of Rights and Responsibilities. Accessible through the CoVPN protocol-specific website.
- NIH Policy on Reporting Race and Ethnicity Data: Subjects in Clinical Research. Available at https://grants.nih.gov/grants/guide/notice-files/NOT-OD-01-053.html
- Title 21, Code of Federal Regulations, Part 50. Available at http://www.accessdata.fda.gov/scripts/cdrh/cfdocs/cfcfr/CFRSearch.cfm?CFRPart=50
- Title 45, Code of Federal Regulations, Part 46 (2018 requirements). Current requirements available at https://www.hhs.gov/ohrp/regulations-and-policy/regulations/45-cfr-46/index.html
- VAERS, Vaccine Adverse Event Reporting System. Available at https://vaers.hhs.gov/reportevent.html

- VAERS, Vaccine Adverse Event Reporting System FAQ. Available at https://vaers.hhs.gov/faq.html

- V-safe After Vaccination Health Checker. Available at https://www.cdc.gov/coronavirus/2019-ncov/vaccines/safety/vsafe.html

See Section 16 for literature cited in the background and statistics sections of this protocol.

15 Acronyms and abbreviations

Ab	antibody
ACIP	Advisory Committee on Immunization Practices
AE	adverse event
AUC	area under the curve
BAMA	binding antibody multiplex assay
CAB	Community Advisory Board
CDC	US Centers for Disease Control and Prevention
CFR	Code of Federal Regulations
CI	confidence intervals
COVID-19	Coronavirus disease 2019
CoVPN	COVID-19 Prevention Network
CRF	case report form
CRPMC	NIAID Clinical Research Products Management Center
CRS	clinical research site
DAIDS	Division of AIDS (US NIH)
DHHS	US Department of Health and Human Services
EAE	adverse events requiring expedited reporting
EC	Ethics Committee
eDiary	Electronic diary
ELISA	enzyme-linked immunosorbent assay
EUA	emergency use authorization
FAS	Full analysis set
FDA	US Food and Drug Administration
Fred Hutch	Fred Hutchinson Cancer Research Center
GCP	Good Clinical Practice
GMT	geometric mean titer
HIPAA	Health Insurance Portability and Accountability Act
HIV	human immunodeficiency virus
IB	Investigator's Brochure
IBC	Institutional Biosafety Committee
ICH	International Council on Harmonisation of Technical Requirements for Pharmaceuticals for Human Use
IQR	Interquartile range
IDMC	Independent Data Monitoring Committee
IM	Intramuscular
IRB	Institutional Review Board
LC	Laboratory Center

LNP	Lipid nanoparticle
LOC	Leadership and Operations Center
mRNA	Messenger ribonucleic acid
nAb	neutralizing antibody
NHP	nonhuman primate
NIAID	National Institute of Allergy and Infectious Diseases (US NIH)
NIH	US National Institutes of Health
OHRP	US Office for Human Research Protections
PCR	polymerase chain reaction
PI	Principal Investigator
RE	regulatory entity
SAP	Statistical Analysis Plan
SCHARP	Statistical Center for HIV/AIDS Research and Prevention
SDMC	statistical and data management center
SSP	Study specific procedures
SOP	Standard operating procedure
UW-VL	University of Washington Virology Laboratory
UW-VSL	University of Washington Virology Specialty Laboratory
VAERS	Vaccine Adverse Event Reporting System
VE	Vaccine efficacy
VE_I	Vaccine efficacy against infectiousness
VEs	Vaccine efficacy against susceptibility
VL	Viral load

16 Literature cited

1. Johns Hopkins Coronavirus Resource Center. 2020 [Available from: https://coronavirus.jhu.edu/map.html.

2. Lipsitch M, Dean NE. Understanding COVID-19 vaccine efficacy. Science. 2020.

3. Mehrotra DV, Janes HE, Fleming TR, Annunziato PW, Neuzil KM, Carpp LN, et al. Clinical Endpoints for Evaluating Efficacy in COVID-19 Vaccine Trials. Ann Intern Med. 2020.

4. Long QX, Tang XJ, Shi QL, Li Q, Deng HJ, Yuan J, et al. Clinical and immunological assessment of asymptomatic SARS-CoV-2 infections. Nat Med. 2020;26(8):1200-4.

5. Salvatore PP, Sula E, Coyle JP, Caruso E, Smith AR, Levine RS, et al. Recent Increase in COVID-19 Cases Reported Among Adults Aged 18-22 Years - United States, May 31-September 5, 2020. MMWR Morb Mortal Wkly Rep. 2020;69(39):1419-24.

6. Boehmer TK, DeVies J, Caruso E, van Santen KL, Tang S, Black CL, et al. Changing Age Distribution of the COVID-19 Pandemic - United States, May-August 2020. MMWR Morb Mortal Wkly Rep. 2020;69(39):1404-9.

7. CDC: Centers for Disease Control and Prevention. COVID-19 Hospitalization and Death by Age 2020 [Available from: https://www.cdc.gov/coronavirus/2019-ncov/covid-data/investigations-discovery/hospitalization-death-by-age.html.

8. Halloran ME, Longini IM, Jr., Struchiner CJ. Design and interpretation of vaccine field studies. Epidemiol Rev. 1999;21(1):73-88.

9. Swan DA, Goyal A, Bracis C, Moore M, Krantz E, Brown E, et al. Vaccines that prevent SARS-CoV-2 transmission may prevent or dampen a spring wave of COVID-19 cases and deaths in 2021. medRxiv. 2020.

10. Swan DA, Bracis C, Janes H, Moore M, Matrajt L, Reeves DB, et al. COVID-19 vaccines that reduce symptoms but do not block infection need higher coverage and faster rollout to achieve population impact. medRxiv. 2020.

11. Datta S, Halloran ME, Longini IM, Jr. Efficiency of estimating vaccine efficacy for susceptibility and infectiousness: randomization by individual versus household. Biometrics. 1999;55(3):792-8.

12. Milani GP, Dioni L, Favero C, Cantone L, Macchi C, Delbue S, et al. Serological follow-up of SARS-CoV-2 asymptomatic subjects. Sci Rep. 2020;10(1):20048.

13. Piccoli L, Park YJ, Tortorici MA, Czudnochowski N, Walls AC, Beltramello M, et al. Mapping Neutralizing and Immunodominant Sites on the SARS-CoV-2 Spike Receptor-

Binding Domain by Structure-Guided High-Resolution Serology. Cell. 2020;183(4):1024-42 e21.

14. Vaccines and Related Biological Products Advisory Committee Meeting. Sponsor Briefing Document, Moderna COVID-19 Vaccine 2020 [Available from: https://www.fda.gov/media/144452/download.

15. Vaccines and Related Biological Products Advisory Committee Meeting. Sponsor Briefing Document Addendum,Moderna COVID-19 Vaccine 2020 [Available from: https://www.fda.gov/media/144453/download.

16. Flood S, King M, Rodgers R, Ruggles S, Warren J. Integrated Public Use Microdata Series, Current Population Survey: version 7.0 [dataset]. 2020.

17. The New York Times. Tracking the Coronavirus at U.S. Colleges and Universities 2020 [Available from: https://www.nytimes.com/interactive/2020/us/covid-college-cases-tracker.html.

18. Business Insider. The 10 biggest coronavirus outbreaks on college campuses across the US since the fall semester began 2020 [Available from: https://www.businessinsider.com/10-colleges-with-fall-coronavirus-outbreaks-1200-cases-or-more-2020-9.

19. Wilson E, Donovan CV, Campbell M, Chai T, Pittman K, Sena AC, et al. Multiple COVID-19 Clusters on a University Campus - North Carolina, August 2020. MMWR Morb Mortal Wkly Rep. 2020;69(39):1416-8.

20. Buitrago-Garcia D, Egli-Gany D, Counotte MJ, Hossmann S, Imeri H, Ipekci AM, et al. Occurrence and transmission potential of asymptomatic and presymptomatic SARS-CoV-2 infections: A living systematic review and meta-analysis. PLoS Med. 2020;17(9):e1003346.

21. Goyal A, Reeves DB, Cardozo-Ojeda EF, Schiffer JT, Mayer BT. Wrong person, place and time: viral load and contact network structure predict SARS-CoV-2 transmission and super-spreading events. medRxiv. 2020.

22. He X, Lau EHY, Wu P, Deng X, Wang J, Hao X, et al. Temporal dynamics in viral shedding and transmissibility of COVID-19. Nat Med. 2020;26(5):672-5.

23. Tindale LC, Stockdale JE, Coombe M, Garlock ES, Lau WYV, Saraswat M, et al. Evidence for transmission of COVID-19 prior to symptom onset. Elife. 2020;9.

24. Kissler SM, Fauver JR, Mack C, Tai C, Shiue KY, Kalinich CC, et al. Viral dynamics of SARS-CoV-2 infection and the predictive value of repeat testing. medRxiv. 2020.

25. CDC: Centers for Disease Control and Prevention. Scientific Brief: Community Use of Cloth Masks to Control the Spread of SARS-CoV-2 2020 [Available from: https://www.cdc.gov/coronavirus/2019-ncov/more/masking-science-sars-cov2.html.

26. Jung CY, Park H, Kim DW, Choi YJ, Kim SW, Chang TI. Clinical Characteristics of Asymptomatic Patients with COVID-19: A Nationwide Cohort Study in South Korea. Int J Infect Dis. 2020;99:266-8.

27. Poteat T, Millett GA, Nelson LE, Beyrer C. Understanding COVID-19 risks and vulnerabilities among black communities in America: the lethal force of syndemics. Ann Epidemiol. 2020;47:1-3.

28. Davies NG, Klepac P, Liu Y, Prem K, Jit M, group CC-w, et al. Age-dependent effects in the transmission and control of COVID-19 epidemics. Nat Med. 2020;26(8):1205-11.

29. Richmond CS, Sabin AP, Jobe DA, Lovrich SD, Kenny PA. SARS-CoV-2 sequencing reveals rapid transmission from college student clusters resulting in morbidity and deaths in vulnerable populations. medRxiv. 2020.

30. Regeneron Pharmaceuticals. Regeneron's REGN-CoV2 antibody cocktail reduced viral levels and improved symptoms in non-hospitalized COVID-19 patients 2020 [Available from: https://newsroom.regeneron.com/news-releases/news-release-details/regenerons-regn-cov2-antibody-cocktail-reduced-viral-levels-and.

31. Chen P, Nirula A, Heller B, Gottlieb RL, Boscia J, Morris J, et al. SARS-CoV-2 Neutralizing Antibody LY-CoV555 in Outpatients with Covid-19. N Engl J Med. 2020.

32. Heneghan C, Brassey J, Jefferson T. SARS-CoV-2 viral load and the severity of COVID-19. The Centre for Evidence-Based Medicine. 2020.

33. Pujadas E, Chaudhry F, McBride R, Richter F, Zhao S, Wajnberg A, et al. SARS-CoV-2 viral load predicts COVID-19 mortality. Lancet Respir Med. 2020;8(9):e70.

34. Westblade LF, Brar G, Pinheiro LC, Paidoussis D, Rajan M, Martin P, et al. SARS-CoV-2 Viral Load Predicts Mortality in Patients with and without Cancer Who Are Hospitalized with COVID-19. Cancer Cell. 2020;38(5):661-71 e2.

35. Letizia AG, Ramos I, Obla A, Goforth C, Weir DL, Ge Y, et al. SARS-CoV-2 Transmission among Marine Recruits during Quarantine. N Engl J Med. 2020.

36. Datta S, Halloran ME, Longini IM, Jr. Augmented HIV vaccine trial design for estimating reduction in infectiousness and protective efficacy. Stat Med. 1998;17(2):185-200.

37. Halloran ME, Struchiner CJ. Causal inference in infectious diseases. Epidemiology. 1995;6(2):142-51.

38. Yang Y, Longini IM, Jr., Halloran ME. Design and Evaluation of Prophylactic Interventions Using Infectious Disease Incidence Data from Close Contact Groups. J R Stat Soc Ser C Appl Stat. 2006;55(3):317-30.

39. Klick B, Leung GM, Cowling BJ. Optimal design of studies of influenza transmission in households. I: case-ascertained studies. Epidemiol Infect. 2012;140(1):106-14.

40. Barnabas RV, Brown ER, Bershteyn A, Stankiewicz Karita HC, Johnston C, Thorpe LE, et al. Hydroxychloroquine as Postexposure Prophylaxis to Prevent Severe Acute Respiratory Syndrome Coronavirus 2 Infection : A Randomized Trial. Ann Intern Med. 2020.

41. Jackson LA, Anderson EJ, Rouphael NG, Roberts PC, Makhene M, Coler RN, et al. An mRNA Vaccine against SARS-CoV-2 - Preliminary Report. N Engl J Med. 2020.

42. Anderson EJ, Rouphael NG, Widge AT, Jackson LA, Roberts PC, Makhene M, et al. Safety and Immunogenicity of SARS-CoV-2 mRNA-1273 Vaccine in Older Adults. N Engl J Med. 2020;383(25):2427-38.

43. Vaccines and Related Biological Products Advisory Committee Meeting. FDA Briefing Document, Moderna COVID-19 Vaccine 2020 [Available from: https://www.fda.gov/media/144434/download.

44. UNAIDS. Ethical considerations in biomedical HIV prevention trials. 2007 7/2007.

45. The National Commission for the Protection of Human Subjects of Biomedical and Behavioral Research. The Belmont Report: Ethical Principles and Guidelines for the Protection of Human Subjects of Research. 1979 4/18/1979.

46. Council for International Organizations of Medical Sciences (CIOMS). International ethical guidelines for biomedical research involving human subjects. Bull Med Ethics. 2002(182):17-23.

47. Sun K, Wang W, Gao L, Wang Y, Luo K, Ren L, et al. Transmission heterogeneities, kinetics, and controllability of SARS-CoV-2. Science. 2021;371(6526).

48. Li F, Li YY, Liu MJ, Fang LQ, Dean NE, Wong GWK, et al. Household transmission of SARS-CoV-2 and risk factors for susceptibility and infectivity in Wuhan: a retrospective observational study. Lancet Infect Dis. 2021.

49. Madewell ZJ, Yang Y, Longini IM, Jr, Halloran ME, Dean NE. Household Transmission of SARS-CoV-2: A Systematic Review and Meta-analysis. JAMA Network Open. 2020;3(12):e2031756-e.

50. Kissler SM, Fauver JR, Mack C, Olesen SW, Tai C, Shiue KY, et al. SARS-CoV-2 viral dynamics in acute infections. medRxiv. 2020.

51. Grambsch PM, Therneau TM. Proportional Hazards Tests and Diagnostics Based on Weighted Residuals. Biometrika. 1994;81(3):515-26.

52. Hernan MA. The hazards of hazard ratios. Epidemiology. 2010;21(1):13-5.

53. Follmann D, Huang CY. Incorporating founder virus information in vaccine field trials. Biometrics. 2015;71(2):386-96.

54. Lin DY. Proportional means regression for censored medical costs. Biometrics. 2000;56(3):775-8.

Appendix A Sample informed consent form for Main cohort

**Sponsor / Study Title:**	**National Institutes of Health, "A randomized controlled study to assess SARS CoV-2 infection, viral shedding, and subsequent potential transmission in university students immunized with Moderna COVID-19 Vaccine"**
**Protocol Number:**	**CoVPN 3006**
**Principal Investigator: (Study Doctor)**	**«PiFullName»**
**Telephone:**	**«IcfPhoneNumber»**
**Address:**	**«PiLocations»**

Thank you for your interest in our research study. It is called vPROTECT or CoVPN 3006. This informed consent form will tell you more about the study. Please read it carefully as you decide if you want to join. If you have questions, please ask us. At the end, we will ask you to answer a few questions to make sure you understand the study.

If you decide to join this study, we will ask you to sign this form electronically. We will offer you a copy of this form to keep.

CoVPN 3006 is a research study. Research is not the same as medical care. The purpose of a research study is to answer scientific questions. We hope that what we learn will help people in the future.

Key information

- Joining this research study is voluntary. It is your choice.

- Our scientific questions are: Does the vaccine protect people from getting infected with a coronavirus called SARS-CoV-2? Does the vaccine prevent people from transmitting SARS-CoV-2 to others?

- If you join, your participation in this study will last for about 5 months.

- If you join, we will ask you to answer questionnaires, get injections, give blood, and take daily swabs of your nose.

  Here are the risks of taking part:

- The most common risk is symptoms such as muscle aches or headaches after getting the injection.

- There is a risk of loss of your personal information.
- There are other, less serious risks. We will tell you more about them later in this consent form.

About the study

The COVID-19 Prevention Network (CoVPN) is doing a study to test a vaccine against SARS-CoV-2. SARS-CoV-2 is the virus that causes the disease called COVID-19. We want to know if the vaccine is able to protect people from getting infected and if getting the vaccine will affect the amount of virus that is in your nose. We also want to know if getting the vaccine will prevent transmitting SARS-CoV-2 to others.

Many people will take part in this study. About 12,000 people will get vaccine injections while about 24,000 will be in the Prospective Close Contact (PCC) group and up to about 1500 more will be in the Case-ascertained Close Contact (CACC) group. These close contact groups will help researchers determine if the vaccine given to you prevents transmission of the SARS-CoV-2 virus to others. We will explain more about this later in this form.

We are asking you to join the Main part of the study in which people get vaccine injections. People who join the Main study will be recruited from universities in the United States (US). The researcher in charge of this study at this clinic is [Insert name of site PI]. The US National Institutes of Health is paying for this study.

1. **This study's vaccine has an Emergency Use Authorization (EUA) for individuals 18 and older.**

The vaccine we will use in this study is called the Moderna COVID-19 Vaccine. The vaccine has been given to over 15,000 people in other research studies and millions more people under EUA. We will give you the most up-to-date "Fact Sheet for Recipients and Caregivers" with more information about the Moderna COVID-19 Vaccine. The person giving you the vaccine can answer any questions you might have about what is in this fact sheet.

We already know that the vaccine can prevent serious COVID-19 disease, but we do not know if the vaccine will work to prevent SARS-CoV-2 infection in others. That is what we are testing in this study.

The Moderna COVID-19 Vaccine is not approved for sale in the United States by the US Food and Drug Administration (FDA).

Although the vaccine is not approved, it has an Emergency Use Authorization (EUA) from the FDA based on its success at preventing disease symptoms from SARS-CoV-2 in studies to date. During a public health emergency, like the COVID-19 pandemic, the FDA can issue an EUA to get vaccines to people faster. When the FDA issues an EUA, they continue to monitor safety.

The vaccine was developed by ModernaTX, Inc. Typical vaccines for viruses are made from a weakened or killed virus, but the Moderna COVID-19 Vaccine is not made from the SARS-CoV-2 virus. The vaccine includes a short segment of messenger ribonucleic acid (mRNA). The mRNA is a genetic code that tells cells how to make a protein. This mRNA is made in a laboratory. When injected into the body, the mRNA vaccine causes some cells to make that viral protein, which can trigger the immune system. Your immune system protects you from disease. If a person is later infected, their immune system remembers the protein from the vaccine which may help it to fight the virus. It is impossible for the vaccine to give you SARS-CoV-2 or COVID-19 infection.

Risks of the Moderna COVID-19 Vaccine:

To date, millions of people have already received the Moderna COVID-19 vaccine. In clinical trials, most people who got the vaccine had some reaction after their injections, especially after the second injection. The reaction to the vaccine didn't affect their daily lives and it went away after two or three days. Most of these people said they had pain in the arm where they got the injection. These people also felt tired, had headaches, muscle and joint pain, and chills. A much smaller number of these people said they had redness or swelling where the needle went in their arm.

The risks of the study vaccine seen so far are the same as what have been seen with most vaccines. Generally, vaccines can cause fever, chills, rash, aches and pains, nausea, headache, dizziness, and feeling tired. Vaccines can also cause pain, redness, swelling, or itching where you got the injection. Most people can still do their planned activities after getting a vaccine. Rarely, people have side effects that limit their normal activities or make them go to the doctor.

Rarely, a vaccine can cause you to have an allergic reaction. You might have a rash, hives, or trouble breathing. Allergic reactions can be life-threatening. Tell us if you have ever had a bad reaction to any injection or vaccine.

There may be other risks that we don't yet know about, even serious ones. We will tell you if we learn about any new risks.

Joining the study

2. **You will receive the vaccine immediately or later.**

   To join this study, you must agree to not get any COVID-19 licensed or emergency-use authorized (EUA) vaccine until the study staff tell you to. Not everyone in this study will get the vaccine immediately. Some people will get the study vaccine 4 months later than the first group of people in the Main study.

   We will compare the results from people who got the vaccine immediately with results from people who got the vaccine later. In this way, we will be able to tell if the vaccine can prevent SARS-CoV-2 infection.

You have a 50/50 chance of getting the vaccine immediately or later. Whether you get the vaccine immediately or a little later is completely random, like flipping a coin.

We have no say in whether you get the vaccine immediately or later.

3. **It is completely up to you whether or not to join this study.**

   Take your time in deciding. If it helps, talk to people you trust, such as your doctor, friends, or family. If you decide not to join this study, or if you leave it after you have joined, it will not change your healthcare or your standing at this university. If you do join, you do not give up your legal rights.

   You cannot be in this study while you are in another study where you get a study product.

   If you choose not to join this study, you may be able to join another study.

4. **If you want to join this study, after you sign this consent, you will download an electronic diary (eDiary) app and take questionnaires in it.**

   We will send you information about how to download the eDiary app onto your phone or tablet. We can help you download the app and show you how to use it.

   To find out if you are eligible to join the study, you will answer questionnaires in the eDiary. Some of the questions will ask about your health history, what medications you take, your age, race, and ethnicity, and where you live. Your answers will tell us about some aspects of your health and if you may be eligible to join the study. It should only take about 10 minutes to complete the questionnaires.

   Your answers to the questions may show you are not eligible to join the study, even if you want to. We might contact you to ask some more questions to further evaluate whether you can join the study.

Being in the study

If you want to join and you are eligible, here is what will happen:

5. **You will be in the study about 5 months.**

   Visits can be done in-person or remotely depending on the type of procedures that need to be done. Visits where you get injections must be done in person. Remote visits will be done whenever possible to reduce potential SARS-CoV-2 exposure.

   You may have to come to the clinic if you have a lab or health issue.

Site: Insert visit lengths.

Visits can last from [#] to [#] hours.

At your first visit, we will give you nasal swab kits and instructions so that you can take daily swabs of your nose. Once you receive your first kit, you will swab your nose every day, and you will record it in the eDiary for the entire study. In all, you will swab both of your nostrils every day for about 4 months. You can ask us questions if you need help. You will return your swabs using drop-off location(s) specified by the clinic or by mail.

If you are in the immediate vaccination group, we will take blood samples in the clinic at both of your injection visits and three more times, one month apart. When we take blood, the amount will depend on the lab tests we need to do. Each time we take your blood, the total amount we will take will not be more than two tablespoons. If for some reason you cannot come to the clinic, you may be asked to self-collect blood using a small device called the Tasso-SST OnDemand. Each time you collect your own blood, the amount will be less than one teaspoon. We will give you everything you need to take the blood and return it to us. We will be looking at how your body responds to the vaccine and if you been exposed to the SARS-CoV-2 virus.

If you are in the later vaccination group, we will take blood samples in the clinic at the first five visits, about one month apart. Each time we take your blood, the total amount we will take will not be more than one teaspoon. If for some reason you cannot come to the clinic, you may be asked to self-collect blood using a small device called the Tasso-SST OnDemand. Each time you collect your own blood, the amount will be less than one teaspoon. We will give you everything you need to take the blood and return it to us. We will be looking to see if you been exposed to the SARS-CoV-2 virus.

The self collection device called the Tasso-SST OnDemand sticks to the skin with a light adhesive. When the button is pressed, a vacuum forms and the device will prick the surface of the skin. The vacuum draws blood out of the capillaries and into a sample pod attached to the bottom of the device.

You will also take a daily questionnaire in the eDiary that asks about the results of your SARS-CoV-2 tests taken at the university or another location. The daily questionnaires will take about 5 minutes to complete. The eDiary will send your phone or tablet a push notification or you will receive a reminder email.

6. **We will give you the vaccine on a schedule.**

You will be in one of 2 groups: The Immediate Vaccination group or the Later Vaccination group. Regardless of which group you are in, you will get 1 injection into your upper arm at 2 separate visits during the study.

We will ask you to sign up for v-safe to report any reactions to the study vaccine. V-safe is a smartphone-based app from the Center for Disease Control (CDC) that uses text messaging and web surveys to check in with you after your COVID-19 vaccination.

Procedures for Immediate Vaccination group	Screening visit(s)	First injection visit	1st month	2nd month	3rd month	4th month
Injection		√	√			
Medical history questionnaire	√	√				
SARS-CoV-2 screening at the University		About 2x per week				
eDiary app	√	Daily through month 4				
Blood drawn		√	√	√	√	√
Nasal swab		Daily through month 4				

Procedures for Later Vaccination group	Screening visit(s)	1st Day	1st month	2nd month	3rd month	First injection visit (4th month)	5th month
Injection						√	√
Medical history questionnaire	√	√					
SARS-CoV-2 screening at the University		About 2x per week					
eDiary app	√	Daily through month 4					√
Blood drawn		√	√	√	√	√	
Nasal swab		Daily through month 4					

7. **We will compensate you for your participation.**

Site: Insert your compensation plan here, e.g. money for number of swabs returned. Remember to include any costs to participants.

8. **In addition to giving you the vaccine:**

   We will ask you to give a referral code to people with whom you expect to be in frequent close physical proximity during the study, like a roommate or co-worker. We want to ask these people to enroll in another part of this study that will help us find out if the vaccine prevents transmission of the SARS-CoV-2 virus. We are calling this group the Prospective Close Contact group or PCC.

   As part of their study participation, they will be asked to complete a weekly eDiary that includes questionnaires like the ones you completed. If you become infected with SARS-CoV-2, we will ask you to share your status with your PCCs. Then your PCC participants

will be asked to fill out additional questionnaires for 2 weeks as well as take nasal swabs and self-collect blood samples.

When considering who to ask to be your PCC, you should think about whether you feel comfortable or safe sharing your infection status with them if you get SARS-CoV-2. If you don't feel comfortable sharing this information with them, you should not ask them to participate as a PCC.

It is possible that someone you know may also be participating in the Main part of the study and might identify you as a close contact. They will not know you are also participating in the study unless you choose to tell them. If someone else identifies you as a close contact, they will give you their referral code. It is okay to be enrolled in both the Main part of the study and the PCC group. If this happens, you will be directed to contact the clinic, and we will ask you to give about a half of a teaspoon of blood and complete one additional eDiary questionnaire.

We may also ask you for the name of someone who can tell us how you are doing if we can't reach you or if you are unable to talk. We may contact you after the main study ends (for example, to tell you about the study results).

We will ask you to continue to follow the university's program for SARS-CoV-2 testing and contact tracing. If you test positive for SARS-CoV-2, please tell us as soon as possible.

9. **The CoVPN will test your samples to see how your body, including your immune system, responds to the study products.**

We will send your samples to labs approved by the CoVPN. Your samples will not be labeled with your name or other identifying information.

Researchers may also do genetic testing related to this study on your samples. Your genes are passed to you from your birth parents. Differences in people's genes can help explain why some people get a disease while others do not. The genetic testing will only involve some of your genes, not all of your genes (your genome). The researchers will study only the genes related to the immune system and coronavirus, and genes that may affect how people get coronavirus.

If you get SARS-CoV-2, the researchers may look at all of the virus' genes that are in your samples. The researchers will use this information to learn more about SARS-CoV-2 and how the virus is impacted by the study vaccine.

The tests the researchers do on your samples are for research purposes, not to check your health. The labs will not give the results to you or this clinic because their tests are not approved for use in making health care decisions. These labs are only approved to do research tests.

When your samples are no longer needed for this study, the CoVPN will continue to store them.

10. **We will do our best to protect your private information.**

US sites: Check Health Insurance Portability and Accountability Act (HIPAA) authorization for conflicts with this section.

We will keep your study records and samples in a secure location. We will label all of your samples and most of your records with a code number, not your name or other personal information. We will not share your name with anyone who does not need to know it.

Your records may also be reviewed by groups who watch over this study. These groups include:

- Study monitors
  - The CoVPN, people who work for it, and companies that help it with this study
  - Some government agencies:
    - The US National Institutes of Health
    - The US Office for Human Research Protections
    - Any regulatory agency that reviews research studies
  - Some committees that make sure we protect your rights and keep you safe:
    - The Independent Data Monitoring Committee
    - Advarra Institutional Review Board (IRB)
    - *Sites may, but are not required to include:* [Insert name of local IRB/EC]
    - *Sites include:* [Insert name of local IBC]

All reviewers will take steps to keep your records private.

We cannot guarantee absolute privacy. If you are found to have a medical condition that we are required to report by law, then some of your information may be shared. For people who have received the vaccine and become pregnant, vaccination providers are required to report the pregnancy and pregnancy outcome to the FDA/CDC Vaccine Adverse Event Reporting System (VAERS).

At this clinic, we have to report the following information:

Site: Include any public health or legal reporting requirements. Bulleted examples should include all appropriate cases (reportable communicable disease, risk of harm to self or others, etc.)

- SARS-CoV-2
- Pneumonia
- [Item 3]
- [Item 4]

US sites: Include the following boxed text. You can remove the box around the text.

We have a Certificate of Confidentiality from the US government to help protect your privacy. With the certificate, we do not have to release information about you to someone who is not connected to the study, such as the courts or police. Sometimes we can't use the certificate. Since the US National Institutes of Health funds this research, we cannot withhold information from it.

We will not share your name or information that can identify you with the CoVPN. The CoVPN may share information from this study with other researchers. Researchers may publish the results of this study.

Site: The text below may not be deleted or changed per FDA requirement. You can remove the box.

A description of this clinical trial will be available on http://www.ClinicalTrials.gov, as required by U.S. Law. This website will not include information that can identify you. At most, the website will include a summary of the results. You can search this website at any time.

11. **We may take you out of the study at any time.**

We may take you out of the study if:

- you do not follow instructions,
- we think that staying in the study might harm you,
- the study is stopped for any reason.

12. **If you get COVID-19, we will ask you to do several things.**

If you find out that you have COVID-19, please let us know right away. We will ask you to quarantine per local guidelines. By agreeing to join this study, you also agree to allow this university to share your test results with us.

We will ask you to tell anyone with whom you have recently had close physical contact that they may have been exposed to COVID-19. We will give you a referral code to give to these contacts so that they can decide if they would like to join the Case Ascertained Close Contact

group (CACC). You may remember at the beginning of this form that we told you that about 1,500 people will be enrolled in the CACC group. The CACC group will help us learn if people who got the study vaccine are less likely to give SARS-CoV-2 to someone else. When considering who to ask to be your CACC, you should think about whether you feel comfortable or safe sharing your infection status with them if you get SARS-CoV-2. If you don't feel comfortable sharing this information with them, you should not ask them to participate as a CACC.

It is possible that someone you know may also be participating in the Main part of the study and might identify you as a close contact. They will not know you are also participating in the study unless you choose to tell them. If someone else identifies you as a close contact, they will give you their referral code. It is okay to be enrolled in both the Main part of the study and the CACC group.

If you find out that you have COVID-19, you will be directed to contact the clinic, and we will ask you to either come to the clinic to have your blood drawn or we will ask you to self-collect less than one teaspoon of blood and complete one additional eDiary questionnaire. It is important that you give the blood sample as soon as possible after finding out the you have COVID-19.

We will ask you to track your COVID-19 symptoms using the eDiary daily for 2 weeks or until the symptoms go away.

After you get better, we will ask you to continue your study participation so that we can monitor your health and safety for the rest of the study.

If you are hospitalized, we will pause your participation. We will ask for your medical records and results from tests you might have received at the hospital. It is unlikely that you will be hospitalized, but to prepare for this, we will ask you to sign and date a medical release of information so we can get records from your doctor or view your hospital records. When you are released from the hospital, please let us know. We will ask you to resume your study participation so that we can continue to monitor your health and safety for the rest of the study.

Site: Modify the following sentence as appropriate. We will not pay for any of your COVID-19 care directly.

Other Risks

13. **There are other risks to being in this study.**

This section describes the other risks and restrictions we know about. There may also be unknown risks, even serious ones. We will tell you if we learn anything new that may affect your willingness to stay in the study.

Risks of routine medical procedures:

In this study, we will do some routine medical procedures. These are taking blood and giving injections. These procedures can cause bruising, pain, fainting, soreness, redness, swelling, itching, a sore, bleeding, and (rarely) muscle damage or infection where you got the injection.

Risks of swabbing your nose:

The feeling of having a small, soft-tipped swab inserted into your nostril and twirled around may be a little uncomfortable, but it should not be painful. There is a small chance there could be some bleeding, but this is unlikely.

Risks to your personal information:

We will take several steps to protect your personal information. Although the risk is very low, it is possible that your personal information could be given to someone who should not have it. If that happened, you could have stress or anxiety.

Risks of genetic testing:

It is unlikely, but the genetic tests done on your samples could show you may be at risk for certain diseases. If others found out, it could lead to discrimination or other problems.

Risk of anxiety/emotional stress:

You may feel anxiety or emotional stress if you experience any of the risks described here. You may feel worried if tests show that you have SARS-CoV-2. Some of the questions we will ask you may make you feel uncomfortable.

Unknown risks:

The vaccine has been shown to prevent adults from getting COVID-19 for at least 2 to 3 months after their injection, but we do not know if it will protect them for any longer. If you get COVID-19, we do not know how the vaccine might affect your illness. It is also unknown if the vaccine will prevent you from giving the virus to someone else. If you get COVID-19, we do not know how the vaccine might affect your illness.

We do not know if getting this vaccine will affect how you respond to any future SARS-CoV-2 vaccines.

We do not know if getting the vaccine will affect pregnancy or breastfeeding. If you have concerns, we recommend that you discuss this with a health care provider.

While you are in this study, you should still wear a mask in public and practice physical distancing.

Benefits

14. **This study may benefit you.**

This study may help you receive a COVID-19 vaccine earlier than you would otherwise be eligible. This study may help researchers find out if the vaccine is able to prevent infection with SARS-CoV-2. If the vaccine later becomes approved and sold, there are no plans to share any money with you.

If you decide not to join this study, you could wait to get the Moderna COVID-19 vaccine or another COVID-19 vaccine in your community. You can also continue to follow local guidelines for SARS-CoV-2 infection. You do not have to join this research study if you don't want to.

Your rights and responsibilities

15. **If you join the study, you have rights and responsibilities.**

You have many rights that we will respect. You also have responsibilities. We list these in the Bill of Rights and Responsibilities for Research. We will give you a copy of it.

Leaving the study

16. **Tell us if you decide to leave the study.**

You are free to leave the study at any time and for any reason. If you want to leave, you will need to tell us. Your care at this clinic, your standing at this university, and your legal rights will not be affected.

Previously collected information about you will remain in the study records and will be included in the analysis of results. Your information can not be removed from the study records.

We may ask you to give a final blood sample or nasal swab.

We believe these steps are important to protecting your health, but it is up to you whether to complete them.

Injuries

Sites: Approval from CoVPN Regulatory Affairs (at CoVPN.core.reg@fredhutch.org) is needed for any change (other than those that the instructions specifically request or those previously approved by CoVPN Regulatory Affairs) to the boxed text. You can remove the box around the text.

17. **If you get sick or injured during the study, contact us immediately.**

Paragraph below for all sites.

Your health is important to us. (*Sites: adjust the following 2 sentences if applicable to the care available at your site)* We will tell you about the care that we can give here. For the care that we cannot provide, we will explain how we will help you get care elsewhere.

2 paragraphs below for all sites.

This research study is covered by a US government program that may provide compensation for a serious injury or death. We will give you a handout with more information about this program. The National Institutes of Health (NIH) does not have a mechanism to provide direct compensation for research related injury.

Some injuries are not physical. For example, you might be harmed emotionally by being in this study. Or you might lose wages because you cannot go to work. However, there are no funds to pay for these kinds of injuries, even if they are study related.

You always have the right to use the court system if you are not satisfied.

18. **When samples are no longer needed for this study, the CoVPN wants to use them in other studies and share them with other researchers.**

The CoVPN calls these samples “extra samples”. The CoVPN will only allow your extra samples to be used in other studies if you agree to this. You will mark your decision at the end of this form.

Do I have to agree? No. You are free to say yes or no, or to change your mind after you sign this form. At your request, the CoVPN will destroy all extra samples that it has. Your decision will not affect your being in this study.

Where are the samples stored? Extra samples are stored in a secure central place called a repository. Your samples will be stored in the CoVPN repository in the United States.

How long will the samples be stored? There is no limit on how long your extra samples will be stored. *[Site: Revise the previous sentence to insert limits if your regulatory authority imposes them.]*

Will I be paid for the use of my samples? No. Researchers may make scientific discoveries or products using your samples. If this happens, there is no plan to share any money with you.

Will I benefit from allowing my samples to be used in other studies? Probably not. Results from these other studies are not given to you, this clinic, or your doctor. They may help other people in the future.

Will the CoVPN sell my samples and information? No, but the CoVPN may share your samples with other researchers. Once the CoVPN shares your samples and information, it may not be able to get them back.

What information is shared with researchers? The samples and information will be labeled with a code number which will not be removed. The key to the code will stay at this clinic. However, some information that the CoVPN shares may be personal, such as your race, ethnicity, sex, and health information from the study.

What kind of studies might be done with my extra samples and information? The studies will be related to vaccines, the immune system, coronavirus, and other diseases. Researchers may also do genetic testing on your samples.

If you agree, researchers may compare all of your genes (your genome) to the genomes of many other people. Researchers look for common patterns of genes to help them understand diseases. The researchers may put the information into a protected database so that other researchers can access it. Your name and other personal information will not be included.

Usually, no one could connect your genome to you as a person. There are rules against this. It’s also really difficult to do. But there is a risk that someone could combine information from your genome and other public information about you and identify you. If others found out, it could lead to discrimination or other problems. The risk of this happening is very small.

Who will have access to my information in studies using my extra samples?

People who may see your information are:

- Researchers who use your extra samples and information for other research
- Government agencies that fund or monitor the research using your extra samples and information
- The researcher’s Institutional Review Board or Ethics Committee
- Any regulatory agency that reviews research studies
- The people who work with the researcher

All of these people will do their best to protect your information. If they publish their research, they will not use your name or identify you personally.

Questions

19. **Whom to contact about this study**

    During the study, if you experience any medical problems, suffer a research-related injury, or have questions, concerns, or complaints about the study, please contact the study doctor at the telephone number listed on the first page of this consent document. If you seek emergency care, or hospitalization is required, alert the treating physician that you are participating in this research study.

    An institutional review board (IRB) is an independent committee established to help protect the rights of research subjects.

    If you have any questions about your rights as a research subject, and/or concerns or complaints regarding this research study, contact:
    by mail:
    Study Subject Adviser
    Advarra IRB
    6940 Columbia Gateway Drive, Suite 110
    Columbia, MD 21046
    or call **toll free**: 877-992-4724
    or by **email**: adviser@advarra.com

    Please reference the following number when contacting the Study Subject Adviser: [Advarra to insert number]

    US sites may include, but are not required to: You may also contact the [name of local IRB/EC] at [insert contact information].

Your permissions and signature

20. **In Section 18, we told you above about possible other uses of your extra samples** and information outside this study. Please choose only one of the options below and write your initials in the box next to it. Whatever you choose, the CoVPN keeps track of your decision about how your samples and information can be used. You can change your mind after signing this form.

☐ I allow my extra samples and information to be used for other studies related to vaccines, the immune system, coronavirus, and other diseases. This may include genetic testing.

OR

☐ I agree to the option above *and* also to allow my extra samples and information to be used in studies that look at my whole genome.

OR

☐ I do not allow my extra samples to be used in any other studies. This includes not allowing genetic testing, or studies that look at my whole genome.

21. **If you agree to join this study, you will need to sign below. Before you sign, make sure of the following:**

- You have read this consent form.
- You feel that you understand what the study is about and what will happen to you if you join. You understand what the possible risks and benefits are.
- You have had your questions answered and know that you can ask more.
- You agree to join this study.

You will not be giving up any of your rights by signing this consent form.

Participant's Name (Print)	Participant's Signature	Date	Time
Clinic Staff Conducting Consent Discussion (Print)	Clinic Staff Signature	Date	Time

Appendix B Sample consent form for Prospective Close Contact (PCC) cohort

**Sponsor / Study Title:**	**National Institutes of Health, "A randomized controlled study to assess SARS CoV-2 infection, viral shedding, and subsequent potential transmission in university students immunized with Moderna COVID-19 Vaccine"**
**Protocol Number:**	**CoVPN 3006**
**Principal Investigator: (Study Doctor)**	**«PiFullName»**
**Telephone:**	**«IcfPhoneNumber»**
**Address:**	**«PiLocations»**

Thank you for your interest in our research study. It is called vPROTECT or CoVPN 3006. This informed consent form will tell you more about the study. Please read it carefully to help you decide if you want to join. If you have questions, please ask us.

If you decide to join this study, we will ask you to sign this form electronically. We will offer you a copy of this form to keep.

CoVPN 3006 is a research study. Research is not the same as medical care. The purpose of a research study is to answer scientific questions. We hope that what we learn will help people in the future.

Key information

- Joining this research study is voluntary. It is your choice.
- Our scientific questions are: Does the study vaccine protect people from getting infected with a coronavirus called SARS-CoV-2? Does the study vaccine prevent people from transmitting SARS-CoV-2 to others?
- If you join, your participation in this study will last for about 5 months.
- If you join, we will ask you to answer weekly eDiary questionnaires. If you or your contact in the main study group becomes SARS-CoV-2 infected, then we will ask you to take daily swabs of your nose, answer daily eDiary questionnaire for 2 weeks, and give 2 blood samples.

  Here are the risks of taking part:

- There is a risk of loss of your personal information.

- There are other, less serious risks. We will tell you more about them later in this consent form.

About the study

The COVID-19 Prevention Network (CoVPN) is doing a study to test a vaccine against SARS-CoV-2. SARS-CoV-2 is the virus that causes the disease called COVID-19. We want to know if the vaccine is able to protect people from getting infected and if getting the vaccine will affect the amount of virus that is in your nose. We also want to know if getting the vaccine will prevent transmitting SARS-CoV-2 to others.

We are asking you to join this study because you have been referred as a close contact with someone who is in the Main study and is getting injections of the vaccine.

Many people will take part in this study. About 12,000 people will get injections while about 24,000 will be in the Prospective Close Contact (PCC) group and up to about 1,500 more will be in the Case-ascertained Close Contact (CACC) group. These close contact groups will help researchers determine if the vaccine prevents transmission of the SARS-CoV-2 virus.

People who join the close contact groups and Main study are being recruited from many universities in the United States (US). You are being asked to participate in the PCC group. The researcher in charge of this study at this clinic is [Insert name of site PI]. The US National Institutes of Health is paying for this study.

Joining the study

1. **It is completely up to you whether or not to join this study.**

   Take your time in deciding. If it helps, talk to people you trust, such as your friends or family. If you decide not to join this study, or if you leave it after you have joined, it will not change your healthcare. If you do join, you do not give up your legal rights.

Being in the study

If you want to join, here is what will happen:

We will send you information about how to download an electronic diary (eDiary) app onto your phone or tablet. We can help you download the app and show you how to use it.

Initially, you will answer a 5-minute questionnaire in the eDiary. It will ask you about things such as your sex, age, race, and ethnicity. You will also answer weekly questionnaires about whether you are sick or if you have tested positive for SARS-CoV-2. If you have tested positive, you will enter the results of your SARS-CoV-2 tests taken at the university or another location into the eDiary.

If you or your contact in the main study group becomes SARS-CoV-2 infected, then we will ask you to take swabs of your nose and answer eDiary questionnaires every day for 2 weeks. You will return your nasal swabs using drop-off location(s) specified by the clinic or by mail.

We will ask you to self-collect a small amount of your blood twice using a small device called the Tasso-SST OnDemand. You will take the first blood sample as soon as possible, and then you will take another sample about a month later. This will be less than one teaspoon of blood taken from one of your upper arms using a small device. We will give you everything you need to take the blood and return it to us.

The self-collection device sticks to the skin with a light adhesive. When the button is pressed, a vacuum forms, and the device will prick the surface of the skin. The vacuum draws blood out of the capillaries and into a sample pod attached to the bottom of the device.

It is important that you continue to follow the local guidelines for SARS-CoV-2 prevention, like wearing a mask and keeping physical distance from others. If you get COVID-19 and are hospitalized, we will pause your participation. We will ask for your medical records and results from tests you might have received at the hospital. It is unlikely that you will be hospitalized, but to prepare for this, we will ask you to sign and date a medical release of information so we can get records from your doctor or view your hospital records. When you are released from the hospital, please let us know. We will ask you to resume your study participation so that we can continue to monitor your health and safety for the rest of the study.

2. **The CoVPN will test your samples to see how your body, including your immune system, responds to the potential infection.**

   We will send your samples to labs approved by the CoVPN. Your samples will not be labeled with your name or other identifying information.

   Researchers may also do genetic testing related to this study on your samples. Your genes are passed to you from your birth parents. Differences in people's genes can help explain why some people get a disease while others do not. The genetic testing will only involve some of your genes, not all of your genes (your genome). The researchers will study only the genes related to the immune system and coronavirus, and genes that may affect how people get coronavirus.

   If you get SARS-CoV-2, the researchers may look at all of the virus' genes that are in your samples. The researchers will use this information to learn more about SARS-CoV-2 and how the virus is impacted by the study vaccine.

   The tests the researchers do on your samples are for research purposes, not to check your health. The labs will not give the results to you or this clinic because their tests are not approved for use in making health care decisions. These labs are only approved to do research tests.

When your samples are no longer needed for this study, the CoVPN will continue to store them.

3. **When samples are no longer needed for this study, the CoVPN wants to use them in other studies and share them with other researchers.**

The CoVPN calls these samples “extra samples”. The CoVPN will only allow your extra samples to be used in other studies if you agree to this. You will mark your decision at the end of this form.

Do I have to agree? No. You are free to say yes or no, or to change your mind after you sign this form. At your request, the CoVPN will destroy all extra samples that it has. Your decision will not affect your being in this study.

Where are the samples stored? Extra samples are stored in a secure central place called a repository. Your samples will be stored in the CoVPN repository in the United States.

How long will the samples be stored? There is no limit on how long your extra samples will be stored. *[Site: Revise the previous sentence to insert limits if your regulatory authority imposes them.]*

Will I be paid for the use of my samples? No. Researchers may make scientific discoveries or products using your samples. If this happens, there is no plan to share any money with you.

Will I benefit from allowing my samples to be used in other studies? Probably not. Results from these other studies are not given to you, this clinic, or your doctor. They may help other people in the future.

Will the CoVPN sell my samples and information? No, but the CoVPN may share your samples with other researchers. Once the CoVPN shares your samples and information, it may not be able to get them back.

What information is shared with researchers? The samples and information will be labeled with a code number which will not be removed. The key to the code will stay at this clinic. However, some information that the CoVPN shares may be personal, such as your race, ethnicity, sex, and health information from the study.

What kind of studies might be done with my extra samples and information? The studies will be related to vaccines, the immune system, coronavirus, and other diseases.

Researchers may also do genetic testing on your samples.

If you agree, researchers may compare all of your genes (your genome) to the genomes of many other people. Researchers look for common patterns of genes to help them understand diseases. The researchers may put the information into a protected database so that other researchers can access it. Your name and other personal information will not be included.

Usually, no one could connect your genome to you as a person. There are rules against this. It's also really difficult to do. But there is a risk that someone could combine information from your genome and other public information about you and identify you. If others found out, it could lead to discrimination or other problems. The risk of this happening is very small.

Who will have access to my information in studies using my extra samples?

People who may see your information are:

- Researchers who use your extra samples and information for other research
- Government agencies that fund or monitor the research using your extra samples and information
- The researcher's Institutional Review Board or Ethics Committee
- Any regulatory agency that reviews research studies
- The people who work with the researcher

All of these people will do their best to protect your information. If they publish their research, they will not use your name or identify you personally.

4. **We will compensate you for your participation.**

Site: Insert your compensation plan here, e.g. money for number of swabs returned. Remember to include any costs to participants.

5. **We will do our best to protect your private information.**

Site: Check HIPAA authorization for conflicts with this section.

Your study records and samples will be kept in a secure location. We will label all of your samples and most of your records with a code number, not your name or other personal information. We will not share your name with anyone who does not need to know it.

Your records may also be reviewed by groups who watch over this study. These groups include:

- Study monitors
- The CoVPN, people who work for it, and companies that help it with this study
- Some government agencies:
    - The US National Institutes of Health

- The US Office for Human Research Protections
- Any regulatory agency that reviews research studies

- Some committees that make sure we protect your rights and keep you safe:
  - The Independent Data Monitoring Committee
  - Advarra Institutional Review Board (IRB)
  - *Sites may but are not required to include:* [Insert name of local IRB/EC]
  - *Sites include:* [Insert name of local IBC]

All reviewers will take steps to keep your records private.

Sites: Include the following boxed text. You can remove the box around the text.

We have a Certificate of Confidentiality from the US government to help protect your privacy. With the certificate, we do not have to release information about you to someone who is not connected to the study, such as the courts or police. Sometimes we can't use the certificate. Since the US National Institutes of Health funds this research, we cannot withhold information from it.

We will not share your name or information that can identify you with the CoVPN. The CoVPN may share information from this study with other researchers. Researchers may publish the results of this study.

Sites: The text below may not be deleted or changed per FDA requirement. You can remove the box.

A description of this clinical trial will be available on http://www.ClinicalTrials.gov, as required by U.S. Law. This website will not include information that can identify you. At most, the website will include a summary of the results. You can search this website at any time.

6. **We may take you out of the study at any time.**

We may take you out of the study if:

- you do not follow instructions,
- we think that staying in the study might harm you,
- the study is stopped for any reason.

Risks

7. **There are some risks to being in this study.**

   This section describes the risks we know about. There may also be unknown risks, even serious ones. We will tell you if we learn anything new that may affect your willingness to stay in the study.

   Risks of blood draws:

   In this study, we will either take your blood or ask you to self-collect your blood with a small device. Taking blood can cause bruising, pain, fainting, soreness, redness, and itching.

   Risks of swabbing your nose:

   The feeling of having a small, soft-tipped swab inserted into your nostril and twirled around may be a little uncomfortable, but it should not be painful. There is a small chance there could be some bleeding, but this is unlikely.

   Risks to your personal information:

   We will take several steps to protect your personal information. Although the risk is very low, it is possible that your personal information could be given to someone who should not have it. If that happened, you could have stress or anxiety.

Benefits

8. **This study may not benefit you**.

   This study may help researchers find out if people who got the vaccine are less likely to give it to people who they have been in close contact with. If the vaccine later becomes approved and sold, there are no plans to share any money with you.

   If you decide not to join this study, you could wait to get the Moderna COVID-19 vaccine or another COVID-19 vaccine in your community. You can also continue to follow local guidelines for SARS-CoV-2 infection. You do not have to join this research study if you don't want to.

Leaving the study

9. **Tell us if you decide to leave the study.**

   You are free to leave the study at any time and for any reason. If you want to leave, you will need to tell us. Your legal rights will not be affected.

Previously collected information about you will remain in the study records and will be included in the analysis of results. Your information can not be removed from the study records.

We may ask you to give a final blood sample or nasal swab.

We believe these steps are important to protecting your health, but it is up to you whether to complete them.

Injuries

Sites: Approval from CoVPN Regulatory Affairs (at CoVPN.core.reg@fredhutch.org) is needed for any change (other than those that the instructions specifically request or those previously approved by CoVPN Regulatory Affairs) to the boxed text. You can remove the box around the text.

10. If you have a life-threatening medical emergency, call 911 right away or seek help immediately.

We do not expect you to get sick or injured as a result of being in this study. If you get COVID-19 while you are in the study, please let us know. *(Sites: adjust the following 2 sentences if applicable to the care available at your site)* We will tell you about the care that we can give here. For the care that we cannot provide, we will explain how we will help you get care elsewhere. You or your health insurance will have to pay for the care you get elsewhere.

Some injuries are not physical. For example, you might be harmed emotionally by being in this study. Or you might lose wages because you cannot go to work. However, there are no funds to pay for these kinds of injuries, even if they are study related.

You always have the right to use the court system if you are not satisfied.

Questions

11. **Whom to contact about this study**

During the study, if you experience any medical problems, suffer a research-related injury, or have questions, concerns, or complaints about the study, please contact the study doctor at the telephone number listed on the first page of this consent document.

An institutional review board (IRB) is an independent committee established to help protect the rights of research subjects.

If you have any questions about your rights as a research subject, and/or concerns or complaints regarding this research study, contact:

by mail:

Study Subject Adviser
Advarra IRB
6940 Columbia Gateway Drive, Suite 110
Columbia, MD 21046

or call **toll free**: 877-992-4724
or by **email**: adviser@advarra.com

Please reference the following number when contacting the Study Subject Adviser: [Advarra to insert number]

US sites may include, but are not required to: You may also contact the [name of local IRB/EC] at [insert contact information].

Your permissions and signature

12. **In Section 3, we told you above about possible other uses of your extra samples** and information outside this study. Please choose only one of the options below and write your initials in the box next to it. Whatever you choose, the CoVPN keeps track of your decision about how your samples and information can be used. You can change your mind after signing this form.

☐ I allow my extra samples and information to be used for other studies related to vaccines, the immune system, coronavirus, and other diseases. This may include genetic testing.

OR

☐ I agree to the option above *and* also to allow my extra samples and information to be used in studies that look at my whole genome.

OR

☐ I do not allow my extra samples to be used in any other studies. This includes not allowing genetic testing, or studies that look at my whole genome.

13. If you agree to join this study, you will need to sign below. Before you sign, make sure of the following:

- You have read this consent form.
- You feel that you understand what the study is about and what will happen to you if you join. You understand what the possible risks and benefits are.
- You agree to join this study.

You will not be giving up any of your rights by signing this consent form.

Participant's Name (Print)	Participant's Signature	Date	Time
Clinic Staff Conducting Consent Discussion (Print)	Clinic Staff Signature	Date	Time

Appendix C Sample consent form for Case-Ascertained Close Contact (CACC) cohort

**Sponsor / Study Title:**	**National Institutes of Health, "A randomized controlled study to assess SARS CoV-2 infection, viral shedding, and subsequent potential transmission in university students immunized with Moderna COVID-19 Vaccine"**
**Protocol Number:**	**CoVPN 3006**
**Principal Investigator: (Study Doctor)**	**«PiFullName»**
**Telephone:**	**«IcfPhoneNumber»**
**Address:**	**«PiLocations»**

Thank you for your interest in our research study. It is called vPROTECT or CoVPN 3006. This informed consent form will tell you more about the study. Please read it carefully to help you decide if you want to join. If you have questions, please ask us.

If you decide to join this study, we will ask you to sign this form electronically. We will offer you a copy of this form to keep.

CoVPN 3006 is a research study. Research is not the same as medical care. The purpose of a research study is to answer scientific questions. We hope that what we learn will help people in the future.

Key information

- Joining this research study is voluntary. It is your choice.
- Our scientific questions are: Does the study vaccine protect people from getting infected with a coronavirus called SARS-CoV-2? Does the study vaccine prevent people from transmitting SARS-CoV-2 to others?
- If you join, your participation in this study will last for about 1 month.
- If you join, we will ask you to answer questionnaires, give blood, and take daily swabs of your nose for 2 weeks.

  Here are the risks of taking part:

- There is a risk of loss of your personal information.

- There are other, less serious risks. We will tell you more about them later in this consent form.

About the study

The COVID-19 Prevention Network (CoVPN) are doing a study to test a vaccine against SARS-CoV-2. SARS-CoV-2 is the virus that causes the disease called COVID-19. We are asking you to join this study because you recently had close physical contact with someone who is in the Main study and who got infected with SARS-CoV-2. We want to find out if the person you had close physical contact might have transmitted SARS-CoV-2 to you.

Many people will take part in this study. About 12,000 people will get injections while about 24,000 will be in the Prospective Close Contact (PCC) group and up to about 1,500 more will be in the Case-ascertained Close Contact (CACC) group. These close contact groups will help researchers determine if the vaccine prevents transmission of the SARS-CoV-2 virus.

People who join the close contact groups and Main study are being recruited from many universities in the United States (US). You are being asked to participate in the CACC group. The researcher in charge of this study at this clinic is [Insert name of site PI]. The US National Institutes of Health is paying for this study.

Joining the study

1. **It is completely up to you whether or not to join this study.**

Take your time in deciding. If it helps, talk to people you trust, such as your friends or family. If you decide not to join this study, or if you leave it after you have joined, it will not change your healthcare. If you do join, you do not give up your legal rights.

Being in the study

If you want to join, here is what will happen:

We will send you information about how to download an electronic diary (eDiary) app onto your phone or tablet. We can help you download the app and show you how to use it.

You will take a 5-minute questionnaire in the eDiary. It will ask you about your contact with the person who tested positive for SARS-CoV-2, as well as other things such as your sex, age, race, and ethnicity. If you have tested positive for SARS-CoV-2, you will enter the results of your SARS-CoV-2 tests taken at the university or another location into the eDiary.

We will ask you to take daily swabs of both of your nostrils and track your COVID-19 symptoms in the eDiary daily for two weeks. You will return your nasal swabs using drop-off location(s) specified by the clinic or by mail.

We will also ask you to collect a small amount of blood from one of your upper arms twice during the study using a small device called the Tasso-SST OnDemand. This will be less than

one teaspoon of blood taken from one of your upper arms using a small device. You will take the first blood sample as soon as possible, and then you will take another sample about a month later. We will give you everything you need to take these samples and return them to us.

The self-collection device sticks to the skin with a light adhesive. When the button is pressed, a vacuum forms, and the device will prick the surface of the skin. The vacuum draws blood out of the capillaries and into a sample pod attached to the bottom of the device.

It is important that you continue to follow the local guidelines for SARS-CoV-2 prevention, like wearing a mask and keeping physical distance from others.

If you are hospitalized, we will pause your participation. We will ask for your medical records and results from tests you might have received at the hospital. It is unlikely that you will be hospitalized, but to prepare for this, we will ask you to sign and date a medical release of information so we can get records from your doctor or view your hospital records. When you are released from the hospital, please let us know. We will ask you to resume your study participation so that we can continue to monitor your health and safety for the rest of the study.

2. **The CoVPN will test your samples to see how your body, including your immune system, responds to the potential infection.**

   We will send your samples to labs approved by the CoVPN. Your samples will not be labeled with your name or other identifying information.

   Researchers may also do genetic testing related to this study on your samples. Your genes are passed to you from your birth parents. Differences in people's genes can help explain why some people get a disease while others do not. The genetic testing will only involve some of your genes, not all of your genes (your genome). The researchers will study only the genes related to the immune system and coronavirus, and genes that may affect how people get coronavirus.

   If you get SARS-CoV-2, the researchers may look at all of the virus' genes that are in your samples. The researchers will use this information to learn more about SARS-CoV-2 and how the virus is impacted by the study vaccine.

   The tests the researchers do on your samples are for research purposes, not to check your health. The labs will not give the results to you or this clinic because their tests are not approved for use in making health care decisions. These labs are only approved to do research tests.

   When your samples are no longer needed for this study, the CoVPN will continue to store them.

3. **When samples are no longer needed for this study, the CoVPN wants to use them in other studies and share them with other researchers.**

   The CoVPN calls these samples “extra samples”. The CoVPN will only allow your extra samples to be used in other studies if you agree to this. You will mark your decision at the end of this form.

   Do I have to agree? No. You are free to say yes or no, or to change your mind after you sign this form. At your request, the CoVPN will destroy all extra samples that it has. Your decision will not affect your being in this study.

   Where are the samples stored? Extra samples are stored in a secure central place called a repository. Your samples will be stored in the CoVPN repository in the United States.

   How long will the samples be stored? There is no limit on how long your extra samples will be stored. *[Site: Revise the previous sentence to insert limits if your regulatory authority imposes them.]*

   Will I be paid for the use of my samples? No. Researchers may make scientific discoveries or products using your samples. If this happens, there is no plan to share any money with you.

   Will I benefit from allowing my samples to be used in other studies? Probably not. Results from these other studies are not given to you, this clinic, or your doctor. They may help other people in the future.

   Will the CoVPN sell my samples and information? No, but the CoVPN may share your samples with other researchers. Once the CoVPN shares your samples and information, it may not be able to get them back.

   What information is shared with researchers? The samples and information will be labeled with a code number which will not be removed. The key to the code will stay at this clinic. However, some information that the CoVPN shares may be personal, such as your race, ethnicity, sex, and health information from the study.

   What kind of studies might be done with my extra samples and information? The studies will be related to vaccines, the immune system, coronavirus, and other diseases.

   Researchers may also do genetic testing on your samples.

   If you agree, researchers may compare all of your genes (your genome) to the genomes of many other people. Researchers look for common patterns of genes to help them understand diseases. The researchers may put the information into a protected database so that other researchers can access it. Your name and other personal information will not be included.

   Usually, no one could connect your genome to you as a person. There are rules against this. It’s also really difficult to do. But there is a risk that someone could combine information from your genome and other public information about you and identify you. If others found

out, it could lead to discrimination or other problems. The risk of this happening is very small.

Who will have access to my information in studies using my extra samples?

People who may see your information are:

- Researchers who use your extra samples and information for other research
- Government agencies that fund or monitor the research using your extra samples and information
- The researcher's Institutional Review Board or Ethics Committee
- Any regulatory agency that reviews research studies
- The people who work with the researcher

All of these people will do their best to protect your information. If they publish their research, they will not use your name or identify you personally.

4. **We will compensate you for your participation.**

Site: Insert your compensation plan here, e.g. money for number of swabs returned. Remember to include any costs to participants.

5. **We will do our best to protect your private information.**

Site: Check HIPAA authorization for conflicts with this section.

Your study records and samples will be kept in a secure location. We will label all of your samples and most of your records with a code number, not your name or other personal information. We will not share your name with anyone who does not need to know it.

Your records may also be reviewed by groups who watch over this study. These groups include:

- Study monitors
- The CoVPN, people who work for it, and companies that help it with this study
- Some government agencies:
    - The US National Institutes of Health
    - The US Office for Human Research Protections

- Any regulatory agency that reviews research studies

- Some committees that make sure we protect your rights and keep you safe:
  - The Independent Data Monitoring Committee
  - Advarra Institutional Review Board (IRB)
  - *Sites may but are not required to include:* [Insert name of local IRB/EC]
  - *Sites include:* [Insert name of local IBC]

All reviewers will take steps to keep your records private.

Sites: Include the following boxed text. You can remove the box around the text.

We have a Certificate of Confidentiality from the US government to help protect your privacy. With the certificate, we do not have to release information about you to someone who is not connected to the study, such as the courts or police. Sometimes we can't use the certificate. Since the US National Institutes of Health funds this research, we cannot withhold information from it.

We will not share your name or information that can identify you with the CoVPN. The CoVPN may share information from this study with other researchers. Researchers may publish the results of this study.

Sites: The text below may not be deleted or changed per FDA requirement. You can remove the box.

A description of this clinical trial will be available on http://www.ClinicalTrials.gov, as required by U.S. Law. This website will not include information that can identify you. At most, the website will include a summary of the results. You can search this website at any time.

6. **We may take you out of the study at any time.**

We may take you out of the study if:

- you do not follow instructions,
- we think that staying in the study might harm you,
- the study is stopped for any reason.

Risks

7. **There are some risks to being in this study.**

   This section describes the risks we know about. There may also be unknown risks, even serious ones. We will tell you if we learn anything new that may affect your willingness to stay in the study.

   Risks of blood draws:

   In this study, we will either take your blood or ask you to self-collect your blood with a small device. Taking blood can cause bruising, pain, fainting, soreness, redness, and itching.

   Risks of swabbing your nose:

   The feeling of having a small, soft-tipped swab inserted into your nostril and twirled around may be a little uncomfortable, but it should not be painful. There is a small chance there could be some bleeding, but this is unlikely.

   Risks to your personal information:

   We will take several steps to protect your personal information. Although the risk is very low, it is possible that your personal information could be given to someone who should not have it. If that happened, you could have stress or anxiety.

Benefits

8. **This study may not benefit you.**

   This study may help researchers find out if people who got the vaccine and later got infected with SARS-CoV-2 are less likely to give it to people who they have been in close contact with. If the vaccine later becomes approved and sold, there are no plans to share any money with you.

   If you decide not to join this study, you could wait to get the Moderna COVID-19 vaccine or another COVID-19 vaccine in your community. You can also continue to follow local guidelines for SARS-CoV-2 infection. You do not have to join this research study if you don't want to.

Leaving the study

9. **Tell us if you decide to leave the study.**

   You are free to leave the study at any time and for any reason. If you want to leave, you will need to tell us. Your legal rights will not be affected.

Previously collected information about you will remain in the study records and will be included in the analysis of results. Your information can not be removed from the study records.

We may ask you to give a final blood sample or nasal swab.

We believe these steps are important to protecting your health, but it is up to you whether to complete them.

Injuries

Sites: Approval from CoVPN Regulatory Affairs (at CoVPN.core.reg@fredhutch.org) is needed for any change (other than those that the instructions specifically request or those previously approved by CoVPN Regulatory Affairs) to the boxed text. You can remove the box around the text.

10. If you have a life-threatening medical emergency, call 911 right away or seek help immediately.

We do not expect you to get sick or injured as a result of being in this study. If you get COVID-19 while you are in the study, please let us know. *(Sites: adjust the following 2 sentences if applicable to the care available at your site)* We will tell you about the care that we can give here. For the care that we cannot provide, we will explain how we will help you get care elsewhere. You or your health insurance will have to pay for the care you get elsewhere.

Some injuries are not physical. For example, you might be harmed emotionally by being in this study. Or you might lose wages because you cannot go to work. However, there are no funds to pay for these kinds of injuries, even if they are study related.

You always have the right to use the court system if you are not satisfied.

Questions

11. **Whom to contact about this study**

During the study, if you experience any medical problems, suffer a research-related injury, or have questions, concerns, or complaints about the study, please contact the study doctor at the telephone number listed on the first page of this consent document.

An institutional review board (IRB) is an independent committee established to help protect the rights of research subjects.

If you have any questions about your rights as a research subject, and/or concerns or complaints regarding this research study, contact:

by mail:

Study Subject Adviser
Advarra IRB
6940 Columbia Gateway Drive, Suite 110
Columbia, MD 21046

or call **toll free**: 877-992-4724
or by **email**: adviser@advarra.com

Please reference the following number when contacting the Study Subject Adviser: [Advarra to insert number]

Sites may include, but are not required to: You may also contact the [name of local IRB/EC] at [insert contact information].

Your permissions and signature

12. In Section 3, we told you above about possible other uses of your extra samples and information outside this study. Please choose only one of the options below and write your initials in the box next to it. Whatever you choose, the CoVPN keeps track of your decision about how your samples and information can be used. You can change your mind after signing this form.

☐ I allow my extra samples and information to be used for other studies related to vaccines, the immune system, coronavirus, and other diseases. This may include genetic testing.

OR

☐ I agree to the option above *and* also to allow my extra samples and information to be used in studies that look at my whole genome.

OR

☐ I do not allow my extra samples to be used in any other studies. This includes not allowing genetic testing, or studies that look at my whole genome.

13. **If you agree to join this study, you will need to sign below. Before you sign, make sure of the following:**

- You have read this consent form.
- You feel that you understand what the study is about and what will happen to you if you join. You understand what the possible risks and benefits are.
- You agree to join this study.

You will not be giving up any of your rights by signing this consent form.

Participant's Name (Print)	Participant's Signature	Date	Time
Clinic Staff Conducting Consent Discussion (Print)	Clinic Staff Signature	Date	Time

Appendix D Procedures for Main Cohort, Immediate Vaccination Group

Visit:	01[1]	02[2]	03[2]	04	05	06	07
Day:		D1	D29	D57	D85	D113	D141
Week:		W0	W4	W8	W12	W16	W20
Month:		M0	M1	M2	M3	M4	M5
Procedure	Scr.	VAC1	VAC2				
**Study procedures**							
Screening consent (if used)	✓	–	–	–	–	–	–
Assessment of understanding	✓	–	–	–	–	–	–
Protocol consent (eConsent)	✓	–	–	–	–	–	–
Medical history questionnaire	✓	✓	–	–	–	–	–
SARS-CoV-2 infection risk assessment	✓	Weekly					
COVID-19 symptom surveillance	–	Weekly starts at week 2, not during week 5					
eDiary[3]	✓	Daily					–
Confirm eligibility	✓	–	–	–	–	–	–
Obtain demographics	✓	–	–	–	–	–	–
Randomize	–	✓	–	–	–	–	–
SARS-CoV-2 screening at the University	–	approximately 2x per week					
**Research Samples[4]**							
Serum for Immunogenicity Assays and Storage	–	✓	✓	✓	✓	–	–
Serum for SARS-CoV-2 clinical serology[5]	–	✓[6]	✓[6]	✓[6]	✓[6]	✓	–
Nasal swab	–	Daily[7]					–
**Vaccination procedures**							
COVID-19 symptom check[8]	–	✓	✓	–	–	–	–
Vaccination	–	✓	✓	–	–	–	–

Greyed out visit column does not apply to this group.
1 Screening may occur over the course of several contacts/visits up to and including day 1, prior to vaccination.
2 Blood draws required at vaccination visits must be performed prior to vaccination.
3 For details about eDiary, please see Section 9.15.
4 From screening to the final study visit, the total blood volume to be collected from each participant will be approximately 132 mL (maximum) with a maximum of approximately 25.5mL collected in a single visit.
5 For blood self-collected by a participant the maximum volume will be less than 1 mL in a single collection.
6 SARS-CoV-2 clinical serology testing may be performed on leftover blood from samples collected for immunogenicity assays. No separate blood sample is needed. If a Main cohort participant is also in the Prospective Close Contact (PCC) cohort related to an index case or in the Case-ascertained Close Contact (CACC) cohort, one additional blood collection (self-collection) will be performed (see table below).
7 Daily swabs will be collected from vaccination through D113. Swabs will be returned via drop-off location(s) specified by the clinic or through the mail.
8 COVID-19 Symptom Check to be performed within 24 hours of vaccination.

The following **additional** procedures will occur for Main cohort, immediate vaccination participants, who fall under any of the following categories:

- tested positive for SARS-CoV-2 from enrollment through Month 4 (see Section 9.7)
- enrolled also in the PCC cohort and are related to an index case (see Section 9.8)
- enrolled also in the CACC cohort (see Section 9.9)

Day relative to notification of infection:	D1-14
**Study procedures**	
eDiary for symptom tracking	daily
**Research Samples**	
Serum for SARS-CoV-2 clinical serology[1]	✓[2]

[1] For blood self-collected by a participant the maximum volume will be less than 1 mL in a single collection.

[2] Sample for serology will be collected as soon as possible following report of positive SARS-CoV-2 test.

In addition, we may ask participants to sign a release to allow us to review their COVID-19 related medical records.

Appendix E Procedures for Main Cohort, Delayed Vaccination Group

Visit:	01[1]	02	03	04	05	06	07
Day:		D1	D29	D57	D85	D113	D141
Week:		W0	W4	W8	W12	W16	W20
Month:		M0	M1	M2	M3	M4	M5
Procedure	Scr.					VAC1	VAC2
**Study procedures**							
Screening consent (if used)	✓	–	–	–	–	–	–
Assessment of understanding	✓	–	–	–	–	–	–
Protocol consent (eConsent)	✓	–	–	–	–	–	–
Medical history questionnaire	✓	✓	–	–	–	–	–
SARS-CoV-2 infection risk assessment	✓	Weekly					
COVID-19 symptom surveillance	–	Weekly					
eDiary[2]	✓	Daily					✓
Confirm eligibility	✓	–	–	–	–	–	–
Obtain demographics	✓	–	–	–	–	–	–
Randomize	–	✓	–	–	–	–	–
SARS-CoV-2 screening at the University	–	approximately 2x per week					
**Research Samples**							
Serum for SARS-CoV-2 clinical serology[3]	–	✓	✓	✓	✓	✓	–
Nasal swab	–	Daily[4]					–
**Vaccination procedures**							
COVID-19 symptom check[5]	–	–	–	–	–	✓	✓
Vaccination	–	–	–	–	–	✓	✓

[1] Screening may occur over the course of several contacts/visits up to and including day 1, prior to randomization.
[2] For details about eDiary, please see Section 9.15.
[3] From screening to the final study visit, the total blood volume to be collected from each participant will be approximately 50 mL (maximum) with a maximum of approximately 10 mL collected in a single visit. For blood self-collected by a participant the maximum volume will be less than 1 mL in a single collection. If a Main cohort participant is also in the Prospective Close Contact (PCC) cohort related to an index case or in the Case-ascertained Close Contact (CACC) cohort, one additional blood collection (self-collection) will be performed (see table below).
[4] Daily swabs will be collected from D1 through D113. Swabs will be returned via drop-off location(s) specified by the clinic or through the mail.
[5] COVID-19 Symptom Check to be performed within 24 hours of vaccination.

The following **additional** procedures will occur for Main cohort, delayed vaccination participants, who fall under any of the following categories:

- tested positive for SARS-CoV-2 from enrollment through Month 4 (see Section 9.7)
- enrolled also in the PCC cohort and are related to an index case (see Section 9.8)
- enrolled also in the CACC cohort (see Section 9.9)

Day relative to notification of infection:	D1-14
**Study procedures**	
eDiary for symptom tracking	daily
**Research Samples**	
Serum for SARS-CoV-2 clinical serology[1]	✓[2]

[1]For blood self-collected by a participant the maximum volume will be less than 1 mL in a single collection.

[2] Sample for serology will be collected as soon as possible following report of positive SARS-CoV-2 test

In addition, we may ask participants to sign a release to allow us to review their COVID-19 related medical records.

Appendix F Procedures for Prospective Close Contact (PCC) cohort

Visit:	**100**	**101**
Day:	D1	D113
Month:	M0	M4
	Enrollment	Follow-up
**Study procedures**		
Protocol consent (eConsent)	✓	–
eDiary[1]	weekly	

[1]For details about eDiary, please see Section 9.15.

If a PCC participant (or their Main study contact) tests positive for SARS-CoV-2, the following **additional** procedures will occur:

Day relative to notification of infection:	D1	D29
**Study procedures**		
Additional eDiary questionnaire	Daily for 14 days	
**Research Samples**		
Serum for SARS-CoV-2 clinical serology[1]	✓	✓
Nasal swab[2]	Daily for 14 days	

[1] For blood self-collected by a participant the maximum volume will be less than 1 mL in a single collection

[2]Swabs will be returned via drop-off location(s) specified by the clinic or through the mail.

In addition, we may ask participants to sign a release to allow us to review their COVID-19 related medical records.

Appendix G Procedures for Case-Ascertained Close Contact Cohort

Visit:	**200**	**201**	**202**
Day:	D1	D3	D29
Month:	M0	M0	M1
	Enrollment	Initial specimen collection	Follow-up
**Study procedures**			
Protocol consent (eConsent)	✓		–
eDiary[1]		✓	✓
**Research Samples**			
Serum for SARS-CoV-2 clinical serology[2]		✓	✓
Nasal swab[3]		Daily (for 14 days)	–

[1]For details about eDiary, please see Section 9.15.
[2] For blood self-collected by a participant the maximum volume will be less than 1 mL in a single collection.
[3]Swabs will be returned via drop-off location(s) specified by the clinic or through the mail.

In addition, we may ask participants to sign a release to allow us to review their COVID-19 related medical records.

Appendix H Visit windows

Main Cohort, Immediate Vaccination, Visit Windows

Visit Number	Visit Type	Lower Allowable Window	Lower Target Day	Target Day[1]	Upper Target Day	Upper Allowable Window
01.0	Screening	-56	-		-	0
02.0	**Enrollment/Randomization**	-	-	1	-	-
03.0	**Vaccination 2**	-	-3	29[2]	+7	+14
04.0	Month 2 Follow-up	-	-7	57	+7	+14
05.0	Month 3 Follow-up	-13	-7	85	+7	+14
06.0	Month 4 Follow-up	-13	-7	113	+14	+56

1. Target dates are relative to Visit 2.0 (Enrollment/Randomization), except for cases described in footnote 2.
2. In the event that Vaccination 1 occurs on a different day than Visit 2.0 (Enrollment/Randomization), Visit 3.0 target dates are relative to date of Vaccination 1.

Main Cohort, Delayed Vaccination, Visit Windows

Visit Number	Visit Type	Lower Allowable Window	Lower Target Day	Target Day[1]	Upper Target Day	Upper Allowable Window
01.0	Screening	-56	-		-	0
02.0	**Enrollment/Randomization**	-	-	1	-	-
03.0	Month 1 Follow-up	-	-3	29	+7	+14
04.0	Month 2 Follow-up	-	-7	57	+7	+14
05.0	Month 3 Follow-up	-13	-7	85	+7	+14
06.0	**Vaccination 1**	-13	-7	113	+14	+28
07.0	**Vaccination 2**[1]	-	-3	141	+7	+14

1. Target dates are relative to Visit 2.0 (Enrollment/Randomization), except visit 7.0, which is relative to visit 6.0.

Case-Ascertained Close Contact (CACC) Cohort Visit Windows

Visit Number	Visit Type	Lower Allowable Window	Lower Target Day	Target Day[1]	Upper Target Day	Upper Allowable Window
200.0	**Enrollment**	-	-	1	-	-
201.0	Initial specimen collection		-2	3	+1	
202.0	Day 29 Follow-up	-	-7	29	+14	+28

1. Target dates are relative to Visit 200.0 (Enrollment).

Appendix I Protocol Signature Page

A randomized controlled study to assess SARS CoV-2 infection, viral shedding, and subsequent potential transmission in university students immunized with Moderna COVID-19 Vaccine

I will conduct the study in accordance with the provisions of this protocol and all applicable protocol-related documents. I agree to conduct this study in compliance with United States (U.S.) Health and Human Service regulations (45 CFR 46); applicable U.S. Food and Drug Administration regulations; standards of the International Council for Harmonisation of Technical Requirements for Pharmaceuticals for Human Use (ICH) Guideline for Good Clinical Practice (E6) (R2); Institutional Review Board/Ethics Committee determinations; all applicable in-country, state, and local laws and regulations; and other applicable requirements (eg, U.S. National Institutes of Health, Division of AIDS) and institutional policies.

Investigator of Record Name (print) Investigator of Record Signature Date

Protocol Number: CoVPN 3006

Protocol Version: Version 1.0

Protocol Date: February 17, 2021

COVID-19
Prevention Network

PROTOCOL

CoVPN 3006

A randomized controlled study to assess SARS CoV-2 infection, viral shedding, and subsequent potential transmission in university students immunized with Moderna COVID-19 Vaccine

DOCUMENT ID 38822

Non-IND Study

CLINICAL TRIAL SPONSORED BY

National Institute of Allergy and Infectious Diseases (NIAID)
National Institutes of Health (NIH)
Department of Health and Human Services (DHHS)
Bethesda, Maryland, USA

February 26, 2021
Final
CoVPN 3006
Version 2.0

Contents

1 Overview 5
1.1 Protocol Team 8
2 Background 10
2.1 Rationale for trial concept 10
2.2 Measures of vaccine efficacy 11
2.3 Importance of understanding vaccine effects on infectiousness and transmissibility 12
2.4 Limitations of the current Phase 3 trials for addressing unanswered questions 15
2.5 Burden of SARS-CoV-2 infection and COVID-19 disease on university campuses 16
2.6 Age and Asymptomatic Infection 17
2.7 Evidence for viral load as a predictor of infectiousness 18
2.8 Evidence for viral load as a marker of COVID-19 disease severity and clinical course 19
2.9 Methods for capturing transmission 19
2.10 Moderna COVID-19 Vaccine 21
2.11 Trial design rationale 21
3 Objectives and endpoints 24
4 Ethical considerations 27
5 IRB/EC review considerations 29
5.1 Minimized risks to participants 29
5.2 Reasonable risk/benefit balance 29
5.3 Equitable participant selection 29
5.4 Appropriate informed consent 30
5.5 Adequate safety monitoring 30
5.6 Protect privacy/confidentiality 30
6 Statistical considerations 32
6.1 Accrual and sample size calculations 34
6.2 Role of the Independent Data Monitoring Committee (IDMC) 38
6.3 Randomization 38
6.4 Statistical analyses 38
7 Selection and withdrawal of participants 47
7.1 Inclusion criteria for Main Cohort 47
7.2 Exclusion criteria for Main Cohort 48
7.3 Inclusion criteria for Prospective Close Contact (PCC) cohort 49
7.4 Inclusion criteria for Case-ascertained Close Contact (CACC) cohort 49
8 Study product 50
8.1 Vaccine regimen 50
8.2 Storage, handling, preparation, and administration 50
8.3 Acquisition of Moderna COVID-19 Vaccine 50
9 Clinical procedures 51

9.1 Informed consent 51
9.2 Pre-enrollment procedures for Main Cohort, Immediate and Delayed Vaccination Groups 52
9.3 Enrollment and vaccination visits for Main Cohort, Immediate Vaccination Group 53
9.4 Follow-up procedures for Main Cohort, Immediate Vaccination Group 53
9.5 Enrollment (visit 2) and visit 4 (Month 2) for Main Cohort, Delayed Vaccination Group 54
9.6 Vaccination visits for Main Cohort, Delayed Vaccination Group 54
9.7 Procedures for Main Cohort participant diagnosed with a SARS-CoV-2 infection during the study 54
9.8 Enrollment and follow-up procedures for Prospective Close Contact (PCC) cohort 55
9.9 Enrollment and Follow-up procedures for Case-Ascertained Close Contact (CACC) cohort 56
9.10 Visit windows and missed visits 57
9.11 Early termination visit 57
9.12 Pregnancy for Main Cohort 57
9.13 SARS-CoV-2 monitoring by universities for Main Cohort 57
9.14 COVID-19 symptom surveillance for Main Cohort 58
9.15 Use of electronic Diary (eDiary) for Clinical Outcomes Assessments 58

10 Laboratory 60
10.1 CRS laboratory procedures 60
10.2 Total blood volume 60
10.3 Endpoint assays 60
10.4 Lab assay portfolio 61
10.5 Exploratory studies 61
10.6 Specimen storage and other use of specimens 61
10.7 Biohazard containment 62

11 Safety Reporting 63
11.1 FDA/CDC Vaccine Adverse Event Reporting System (VAERS) 63
11.2 Reporting safety events to v-safe 64

12 Protocol conduct 65
12.1 Study termination 65
12.2 Emergency communication with study participants 66

13 Version history 67

14 Document references (other than literature citations) 69

15 Acronyms and abbreviations 71

16 Literature cited 73

Appendix A Sample informed consent form for Main cohort 78

Appendix B Sample consent form for Prospective Close Contact (PCC) cohort 94

Appendix C Sample consent form for Case-Ascertained Close Contact (CACC) cohort 104

Appendix D Procedures for Main Cohort, Immediate Vaccination Group 114
Appendix E Procedures for Main Cohort, Delayed Vaccination Group 116
Appendix F Procedures for Prospective Close Contact (PCC) cohort 118
Appendix G Procedures for Case-Ascertained Close Contact Cohort 119
Appendix H Visit windows 120
Appendix I Protocol Signature Page 121

1 Overview

Title

A randomized controlled study to assess SARS CoV-2 infection, viral shedding, and subsequent potential transmission in university students immunized with Moderna COVID-19 Vaccine

Primary objective(s)

Primary objective 1:

To evaluate the efficacy of Moderna COVID-19 Vaccine against SARS-CoV-2 infection (ie, to evaluate vaccine efficacy against infection or VE_S)

Primary objective 2:

To evaluate the effect of Moderna COVID-19 Vaccine on peak nasal viral load as a measure of infection and a proxy of infectiousness

Study product and route of administration

Moderna COVID-19 Vaccine: a lipid nanoparticle (LNP) dispersion of a messenger ribonucleic acid (mRNA) encoding the prefusion stabilized S protein of SARS-CoV-2 formulated in LNPs composed of 4 lipids (1 proprietary and 3 commercially available). The Moderna COVID-19 Vaccine, also known as mRNA-1273, is a suspension for intramuscular injection administered as a series of two doses (0.5 mL each) 1 month apart.

This vaccine has been issued an Emergency Use Authorization (EUA) by the US Food and Drug Administration (FDA) for active immunization to prevent COVID-19 in individuals 18 years and older.

Table 1-1 Schema for the Main Cohort

		Injection schedule in months			
Group	N	0	1	4	5
Immediate Vaccination	6000	Moderna COVID-19 Vaccine	Moderna COVID-19 Vaccine	-	-
Delayed Vaccination	6000	-	-	Moderna COVID-19 Vaccine	Moderna COVID-19 Vaccine
Total	12,000				

Participants

Main Cohort (main study participants)

Approximately 12,000 healthy university student volunteers aged 18 through 26 years who will be followed for 5 months after enrollment; 6,000 randomized to immediate vaccination, 6,000 randomized to delayed vaccination

Prospective Close Contact (PCC) Cohort

Up to approximately 24,000 volunteers subject to university SARS-CoV-2 testing who are in frequent close physical proximity with main study participants

Case-Ascertained Close Contact (CACC) Cohort

Approximately 3 contacts per incident case. Actual number dependent on incidence rate. Up to approximately 1500 volunteers aged 18 years and older who have been in close contact with a SARS-CoV-2 positive case from the Main Cohort

Design

Multicenter, randomized, controlled, open-label trial

Duration per participant

Up to 5 months per participant for the Main Cohort and for the Prospective Close Contact Cohort. 1 month per participant in Case-Ascertained Close Contact Cohort.

Estimated total study duration

7 months (includes enrollment and follow-up).

Core operations

CoVPN Vaccine leadership Group/Core Operations Center, Fred Hutchinson Cancer Research Center (Fred Hutch) (Seattle, Washington, USA)

PPD, Inc. (Wilmington, North Carolina, USA)

Statistical and Data Management Center (SDMC)

Statistical Center for HIV/AIDS Research and Prevention (SCHARP), Fred Hutch (Seattle, Washington, USA)

Laboratory Center (LC)

Endpoint assay laboratories

- University of Washington Virology Laboratory (UW-VL) (Seattle, Washington, USA)
- University of Washington Virology Specialty Laboratory (UW-VSL) (Seattle, Washington, USA)
- Pioneer Hi-Bred International, Inc., a member of the Corteva Agriscience group of Companies (Johnston, Iowa, USA)
- Duke University Medical Center (Durham, North Carolina, USA)
- CoreMedica Labs (Lee's Summit, Missouri, USA)

Study sites

- University clinical research sites (CRSs) across the US that have large-scale asymptomatic SARS-CoV-2 testing programs to be specified in the Site Announcement Memo

Study monitoring

Independent Data Monitoring Committee (IDMC)

1.1 Protocol Team

Protocol leadership

Co-chairs	Kathryn Stephenson<br>Harvard University School of Medicine<br>Beth Israel Deaconess Medical Center<br>617-735-4556<br>kstephen@bidmc.harvard.edu<br><br>Audrey Pettifor<br>Department of Epidemiology<br>Gillings School of Global Public Health<br>University of North Carolina<br>919-966-7439<br>apettif@email.unc.edu	*Protocol Lead Advisors*	Larry Corey<br>CoVPN LOC, Fred Hutch<br>206-667-6770<br>lcorey@fredhutch.org<br><br>Myron Cohen<br>University of North Carolina<br>919-966-2537<br>myron_cohen@med.unc.edu<br><br>James Kublin<br>CoVPN LOC, Fred Hutch<br>206-667-1970<br>jkublin@fredhutch.org
Statisticians	Holly Janes<br>CoVPN SDMC, Fred Hutch<br>206-667-6353<br>hjanes@fredhutch.org<br><br>Elizabeth Brown<br>CoVPN SDMC, Fred Hutch<br>206-667-1731<br>erbrown@fredhutch.org	*Laboratory lead*	John Hural<br>CoVPN Laboratory Program<br>206-667-1683<br>jhural@fredhutch.org
Protocol Team leader	Nicole Grunenberg<br>CoVPN LOC, Fred Hutch<br>206-667-2043<br>ngrunenb@fredhutch.org	*DAIDS Medical Officer*	Catherine Yen<br>DAIDS, NIAID<br>240-292-4783<br>catherine.yen@nih.gov

Other contributors to the original protocol

Core medical monitor	Nicole Grunenberg<br>CoVPN LOC, Fred Hutch	*CoVPN Physician consultant*	Nzeera Ketter<br>CoVPN LOC, Fred Hutch
Laboratory consultants	Alexander Greninger<br>Robert Coombs<br>University of Washington Medical Center (UWMC)<br>Keith Jerome<br>Fred Hutch, UWMC	*Clinical safety specialists*	Megan Jones<br>Maija Andersen<br>CoVPN LOC, Fred Hutch
Laboratory center representatives	Nicole Espy<br>Lisa Sanders<br>Jen Hanke<br>CoVPN Laboratory Center, Fred Hutch	*Clinical trials manager*	Julie Hunt<br>CoVPN LOC, Fred Hutch
Regulatory affairs associate	Megan Brandon<br>CoVPN LOC, Fred Hutch	*Clinical data managers*	Melissa Cummings<br>Vicky Kim<br>CoVPN SDMC, Fred Hutch

Community Advisory Board (CAB) members	John Isaac<br>Boston University	*SDMC Associate Director of Operations*	Jessica Andriesen<br>CoVPN SDMC, Fred Hutch
Statistical research associates	Erika Rudnicki<br>Moni Neradilek<br>CoVPN SDMC, Fred Hutch	*SDMC Associate director of lab science*	April Randhawa<br>CoVPN SDMC, Fred Hutch
Community engagement unit representative	Francisco E Rentas<br>CoVPN LOC, Fred Hutch	*Data Management Director*	Kristine Donaty<br>CoVPN SDMC, Fred Hutch
Project coordinator	Kylie McCloskey<br>CoVPN LOC, Fred Hutch	*Protocol development managers*	Kajari Mondal<br>Meg Trahey<br>CoVPN LOC, Fred Hutch

2 Background

2.1 Rationale for trial concept

The United States has the most infections and deaths from SARS-CoV-2 (the virus that causes Coronavirus disease 2019 [COVID-19]) of any nation in the world (1). Fortunately, there is now hope that the large burden of COVID-19-related illness may lessen in 2021 due to the discovery that two candidate COVID-19 vaccines, including the Moderna COVID-19 vaccine, can prevent symptomatic COVID-19 and severe disease in adults (2, 3). Ongoing Phase 3 trials are not designed to estimate a vaccine's efficacy against all infections, with a critical gap being asymptomatic infections. The current trials are also not designed to evaluate vaccine effects on infectiousness as measured by viral shedding or on onward transmission of infection. For example, at an individual level, it is currently unknown if vaccinated individuals should continue to wear masks or social distance, or if they should be quarantined if exposed to COVID-19. At a policy level, it is unknown if vaccination should be required for certain settings, such as public transit including air travel, places of employment, or school. These questions are especially important given that SARS-CoV-2 is highly infectious compared to other respiratory illnesses such as the flu. Recent data also demonstrate that even individuals with asymptomatic COVID-19 can have high titers of nasal viral shedding and thus may be highly infectious. Furthermore, many individuals with asymptomatic infection develop low level and transient antibodies against SARS-CoV-2 (4), suggesting that trials relying on seroconversion as a marker of infection may miss infections.

While there are many populations at risk for SARS-CoV-2 infection, young people are particularly at risk of acquiring and transmitting SARS-CoV-2. Between August and September 2020, COVID-19 cases among young people aged 18-22 years increased 55% nationally (5) and during June-August 2020, young people aged 20-29 years had the highest incidence of disease in the US, accounting for >20% of all confirmed cases (6). Further, increases in SARS-CoV-2 infection among younger adults in the Southern US preceded increases among older adults by 4-15 days, which confirms the importance of younger adults spreading infection to older, more vulnerable populations (5). At the same time, individuals aged 18-29 account for a tiny percentage (0.5%) of all deaths due to COVID-19 and are very unlikely to develop severe disease (7). These reasons plus the congregate and social living situations of university students and that many universities are regularly testing their student body make them an excellent population to examine. The proposed study is designed to evaluate comprehensively the vaccine efficacy profile of the Moderna COVID-19 vaccine, including its effect on acquisition of infection including asymptomatic infection, on disease and severity of disease, on measures of infectiousness, and transmission of infection.

This study is a 2-arm randomized, open-label trial in university students aged 18 through 26 years that will compare those who receive the Moderna COVID-19 Vaccine upon enrollment (immediate vaccination arm) to those who receive the vaccine 4 months later (delayed vaccination arm). **The study will capture all incident SARS-CoV-2 infections among study participants in real time, by partnering with universities who have implemented intensive testing for SARS-CoV-2 among their students.** The university testing systems

will enable infections among study participants to be captured in real time. In addition, study participants will be asked to collect daily nasal swabs, to enable retrospective ascertainment of the timing of acquisition and to capture the full viral load trajectory for incident infections. To capture secondary transmission events, very close contacts of study participants will be enrolled and surveilled by the university for incident infection over the duration of the study ('prospective contacts'); in addition, diagnosis of infection among a study participant will trigger recruitment of additional participant contacts ('case-ascertained close contacts'). When a study participant tests positive based on university testing, both prospective contacts and case-ascertained close contacts will be asked to collect daily nasal swabs for 14 days to detect incident infection and also will have blood collected for serology. The study will take place over the course of the 2021 spring and summer school terms. At the end of the trial, all participants in the delayed arm will be offered vaccine. The goal of the study is to report results prior to fall 2021 to inform school policy prior to when the new year begins for most educational institutions.

2.2 Measures of vaccine efficacy

Ongoing Phase 3 COVID-19 vaccine trials are designed with the primary objective to evaluate vaccine efficacy against COVID-19 disease, written VE_D, and defined as the multiplicative reduction in risk of disease in the vaccine vs. placebo groups. COVID-19 disease is defined by a protocol-specified set of signs and symptoms of disease coincident with PCR-confirmed SARS-CoV-2 infection. While the reduction in risk of disease is arguably the most relevant vaccine effect to the individual participant, understanding the population effect of a vaccine requires considering the other effects of the vaccine (see Table 2-1) (8). The most fundamental effect of a vaccine is to reduce acquisition of infection, sometimes referred to as 'vaccine efficacy against susceptibility', written VE_S, and measured by the multiplicative reduction in PCR-confirmed infection in the vaccine vs. placebo group. A vaccine that reduces acquisition of infection will provide benefit to the individual (i.e. *direct* effects), as well as benefit to the population by reducing the number of infected and therefore potentially infectious individuals who may expose and infect unvaccinated individuals in the population (i.e. *indirect* effects). Modeling studies suggest that COVID-19 vaccines that reduce acquisition are predicted to have considerably greater population impact, not only reducing disease, hospitalizations, and mortality, but reducing transmission of infection and thereby magnifying their effects (9, 10).

An additional potential indirect effect of a vaccine is its effect on reducing secondary transmission of infection, written VE_I and defined as the multiplicative reduction in the rate of secondary transmission among vaccine vs. placebo individuals who become SARS-CoV-2 infected. A vaccine might reduce transmission through reducing the magnitude and/or duration of viral shedding, by reducing symptoms associated with transmission such as coughing, or by selectively blocking infection with less transmissible viral strains. Evaluating the effect of a COVID-19 vaccine on transmissibility is critical because a vaccine with high efficacy against COVID-19 disease but low efficacy against SARS-CoV-2 infection would necessarily yield a net increase in the number of asymptomatic infections. If individuals who become asymptomatically infected despite having received the vaccine are still infectious,

and given that asymptomatic individuals are less likely to be diagnosed and self-isolate, such a vaccine has the potential to increase transmission and prolong the pandemic (2, 3).

As we prepare this study, we recognize there are other approaches to measuring population level benefit (eg, vaccine effect on infection and transmissibility). These approaches include: observational studies, such as those being conducted in Israel to examine population benefit of the vaccine and those planned by the CDC to examine transmission from vaccinated individuals to household contacts in longitudinal cohorts; and ancillary studies with frequent swabbing on a subset of participants in Phase 3 studies. However, none of these studies will allow for the rigorous capture, through daily nasal swabbing, of the early dynamics of viral replication and the capture of transmission events during the acute period of infection in a randomized trial.

All of these vaccine salutatory effects may depend on a variety of individual characteristics such as age, prior infection with SARS-CoV-2; on virological characteristics; and in the case of VE_I which conditions on infection, may depend on clinical characteristics such as symptomatology.

As will be discussed below in detail, the ongoing Phase 3 trials evaluate VE_D and partially identify VE_S, but do not allow direct estimation of VE_I. In contrast, the proposed study will collect data to enable evaluation of all three vaccine efficacy parameters in the university student population. Importantly, the use of an open-label delayed vaccination arm as opposed to a blinded placebo arm implies that the various effects evaluated will capture not only biological vaccine effects, but also potential behavioral effects associated with vaccination, including potential behavioral disinhibition. Behavioral effects most plausibly influence exposure and therefore infection, but knowledge of vaccine receipt is viewed as less likely to influence disease, viral load, and secondary transmission.

Table 2-1 Population measures of vaccine efficacy that will be evaluated using the proposed study, where a delayed vaccination arm will be used instead of placebo

Efficacy measure	Definition
Vaccine efficacy against SARS-CoV-2 infection (VE_S)	The multiplicative reduction in SARS-CoV-2 infection as measured by PCR of nasal swabs, vaccine vs. placebo
Vaccine efficacy against COVID-19 disease (VE_D)	The multiplicative reduction in symptomatic COVID-19*, vaccine vs. placebo
Vaccine efficacy against viral replication (VE_{VL})	The reduction in SARS-CoV-2 viral burden as measured by PCR of nasal swabs, vaccine vs. placebo
Vaccine efficacy against transmission (VE_I)	The multiplicative reduction in rate of SARS-CoV-2 transmission from infected individuals on the vaccine arm relative to the placebo arm

*Defined by a protocol-specific set of signs and symptoms defined in Section 3.

2.3 Importance of understanding vaccine effects on infectiousness and transmissibility

The most effective way for a vaccine to prevent onward transmission of infection is to prevent acquisition of infection. Vaccine efficacy against infection, VE_s, is a measure of this effect (8). However, a vaccine that does not completely block acquisition of infection may

still reduce onward transmission among individuals who become infected despite having received the vaccine, as measured by VE_I (8, 11). As discussed above, understanding the effect of a COVID-19 vaccine on transmission is critical because a vaccine with high efficacy against COVID-19 disease but low efficacy against SARS-CoV-2 infection would necessarily yield a net increase in the number of asymptomatic infections. If individuals who become asymptomatically infected despite having received the vaccine are still infectious, and given that asymptomatic individuals are less likely to be diagnosed and self-isolate, such a vaccine has the potential to increase transmission and prolong the pandemic (2, 3).

If a COVID-19 vaccine did reduce infectiousness, mathematical modeling suggests that much greater population impact could be achieved. Deterministic compartmental models fit to data from King County, Washington and envisioning vaccine rollout starting December 1, 2020 to 18% of the adult population proportionally across age groups, suggest that while a vaccine that reduces COVID-19 disease by 90% and SARS-CoV-2 infection by 10% reduces total infections in 2021 by 18.1% and deaths by 27.5%, if the vaccine additionally reduces infectiousness by 50% the impact on infections would more than double (to 49.3% reduction) and deaths would be cut in half (to 50.9% reduction) (Swan and Dimitrov et al, in preparation; Figure 2-1).

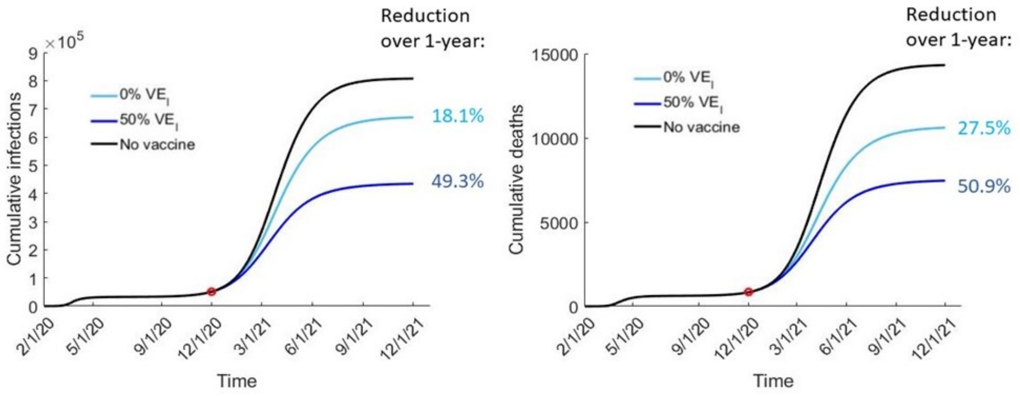

Figure 2-1 COVID-19 vaccine that reduces infectiousness by 50% has greater impact on infections and deaths. Deterministic compartmental model calibrated to King County, WA stratified by age, SARS-CoV-2 infection status, COVID-19 treatment status and vaccination. 800,000 doses vaccine (for 18% of population) starting Dec 1, 2020, allocated proportionally across age groups. Absent vaccination, 20% of infections are asymptomatic; asymptomatic infections are 28% less infectious than symptomatic infections, but more transmissible due to lower rates of diagnosis and quarantine. Both vaccines reduce COVID-19 disease by 90% and SARS-CoV-2 infection by 10%. While one vaccine (light blue) does not reduce infectiousness among individuals who become infected, the other vaccine (dark blue) reduces infectiousness by 50%. As a result, it achieves a much greater reduction in both infections and deaths. (Swan, Dimitrov et al, in preparation).

In the context of limited vaccine supply, mathematical modeling also suggests that optimal allocation of vaccine depends on how well a vaccine reduces infectiousness. Take, for instance, a deterministic compartmental model calibrated to Washington State that uses the following assumptions: that, absent vaccination, 40% of infections are asymptomatic, asymptomatic infections confer equal immunity and are equally infectious, and that there is sufficient vaccine supply for 30% of the population. For a vaccine that reduces COVID-19 disease by 90% but has no effect on infection or infectiousness, the limited supply of vaccine

should be allocated to older adults who have a high burden of disease in order to minimize deaths and peak hospitalizations (Figure 2-2). However, if the vaccine also reduces infectiousness by 60%, minimizing peak hospitalizations is achieved by allocating limited vaccine to the young, while minimizing deaths still requires vaccinating older adults. These results again highlight the critical importance of understanding vaccine effects on acquisition and infectiousness in order to make policy decisions.

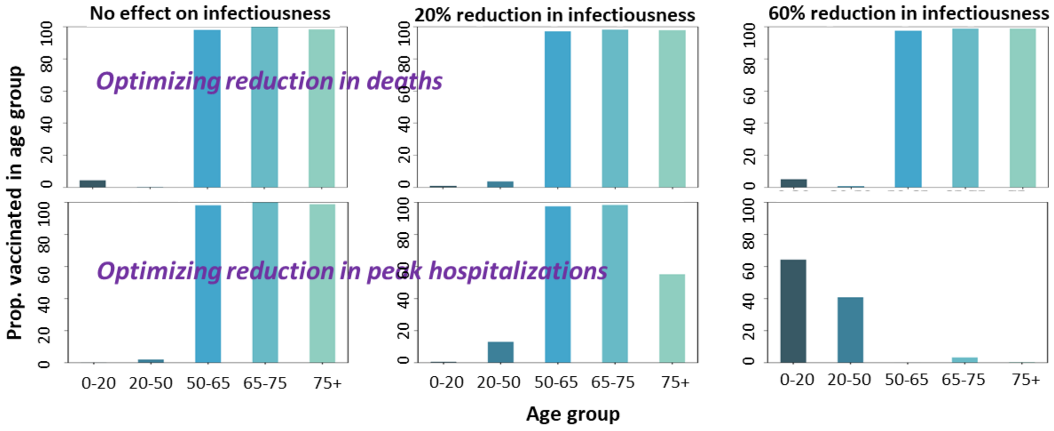

Figure 2-2 Deterministic compartmental model calibrated to Washington State (7.6M people), reflecting US demographics. Model assumes 4.56M doses vaccine (for 30% of population) assuming 10% of the population is previously infected and immune and there are 1000 current infections. Absent vaccination, 40% of infections are asymptomatic; asymptomatic infections confer equal immunity (for at least 6 mo. duration) and are equally infectious. (Matrajt et al, in preparation)

Non-human primate (NHP) data also suggests that elimination of viral shedding by a COVID-19 vaccine is not guaranteed. Table 2-2 shows the comparison of NHP data between candidate COVID-19 vaccines in current Phase 3 trials. While most of the vaccines were shown to prevent viremia within the lower respiratory tract (bronchoalveolar lavage fluid), and thus improve markers of disease severity, three out of the four platforms tested did not eradicate nasal viral shedding in all cases.

Table 2-2 Comparison of NHP data between Janssen Ad26, Moderna RNA, AstraZeneca ChimpAdOx1 and Novavax COVID-19 vaccines

	Ad26<br>One dose<br>1 x10^{11}	mRNA<br>(4 weeks post-dose 2)	AstraZeneca/Oxford<br>2.5x10^{10}<br>0, 4 weeks	Novavax<br>2 doses (0,21)<br>5 mcg, 50 mcg
**Pseudovirus neutralization (GMT)**	408	1,862	5 – 40<br>boost 10 - 160	No
**Live virus assay**	113	3,481		16,026
**Spike protein**	15,000 (not shown)	36,186 (AUC)		335,017
**Lung BAL protection**	Complete	Complete at day 7	75% both prime and boost	Complete
**Nasal protection**	5/6 (83%)	7/8	0%	Complete (12/12)
**T-cell immune response**	Some CD8	No CD8+ T cells	Not reported	CD4 and CD8

Finally, not understanding relative infectivity is a huge gap in translating vaccine use to public health messaging. The demonstration of reduced infectivity may reduce vaccine hesitancy. Public health, medical personnel and organizations may embrace vaccination, as well as users, if the vaccine both provides individual as well as family/community benefit. For individuals, knowing that a vaccine reduces infectivity to household and personal contacts is often a prime motivator for vaccination. Data from this trial can facilitate this messaging as well as help decision-making among individuals about interactions with household members and attending work and school.

2.4 Limitations of the current Phase 3 trials for addressing unanswered questions

Currently, none of the current Phase 3 vaccine efficacy trials will enable robust estimation of vaccine effects on infectiousness. The primary objectives for all trials are to evaluate vaccine efficacy (VE) against COVID-19 disease. Secondary objectives include evaluating VE against seroconversion using infrequent serology (every 1-6 months over 1 year, with more frequent sampling over the first 3 months), but this will not fully capture all infections including those that are asymptomatic. Estimates based on acute infection cohorts (12) suggest that 12-50% of asymptomatic infections might be missed using this sampling, leading to an unknown magnitude and direction of bias in the estimate of VE_S. Data from Long et al. found that 40% of individuals with asymptomatic RT-PCR confirmed SARS-CoV-2 infections were IgG negative at 8 weeks after the acute phase of infection (Figure 2-3) (4). Further, it has been observed that asymptomatic cases have lower IgG titers across all antigens (including NP) (Figure 2-4) (13). In current trials, infectiousness is not captured robustly among those who become infected; viral load (VL) is only measured for those who are symptomatic and only after the onset of symptoms. Data from the Moderna trial where nasal swabs were collected at the time of vaccine administration on days 0 and 28 among all

participants suggest that the vaccine may reduce the level of nasal carriage of virus (14). However, one time point does not robustly capture the full viral dynamics of infection, in particular, asymptomatic infections. The data are suggestive that the vaccine may reduce viral carriage (15). The level of viral shedding prior to the onset of symptoms, when most transmission occurs (16), will not be captured for any infected participants. Importantly, no data in current trials are collected on onward transmission of infection and therefore VE_I cannot be evaluated directly.

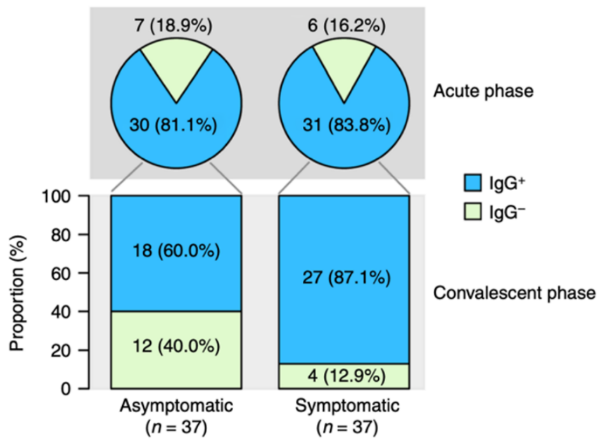

Figure 2-3 40% of asymptomatic individuals were IgG negative by 8 weeks post-acute infection (Long et al.(4).

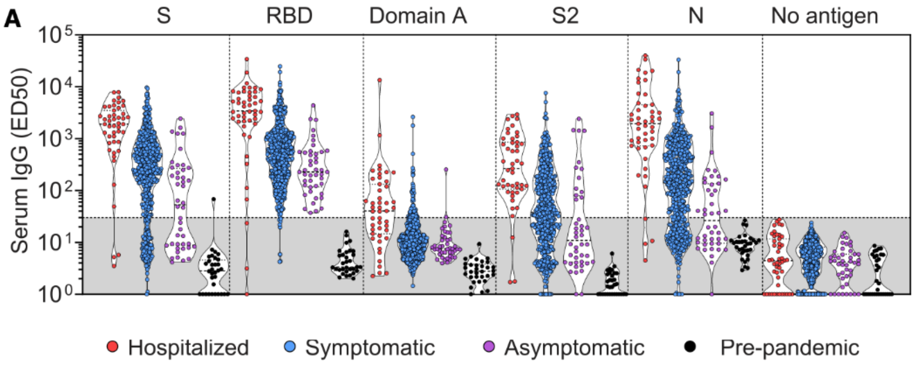

Figure 2-4 Asymptomatic Patients with COVID-19 have Lower IgG Titers Across Multiple Antigens Among Italian Medical Staff N>600 (13).

2.5 Burden of SARS-CoV-2 infection and COVID-19 disease on university campuses

The study will be conducted among students enrolled in universities because these are ideal locations for studying SARS-CoV-2 transmission clusters. Large numbers of cases of COVID-19 have been reported on numerous campuses throughout the US as colleges and universities re-opened in fall 2020. More than 397,000 cases were counted at 1,800+ colleges

and universities in fall 2020 (17, 18). Over the time period July-December 11th, 2020, more than 85 colleges and Universities had more than 1,000 cases. Among these 49 campuses the cumulative attack rate from July to October was on average 6.2%. These estimates are somewhat uncertain given the fact that the number of reported cases for some schools includes faculty, staff and graduate students; however, the number of such individuals is very small in comparison to the number of undergraduates.

Among 15 Association of American Universities (AAU) universities, and using data from the start of semester through September 18, 2020, there was an estimated 3.7% 1-month attack rate. The attack rate appeared to decline over time; it was 1.7% over September 19 to October 20, 2020. Attack rates may be underestimated because the denominator is assumed to be all undergraduates, whereas some schools had only a subset of students on campus. These statistics suggest that a conservative assumption of SARS-CoV-2 incidence among US university students for Fall semester 2020 is 6%. A conservative incidence assumption for Spring and Summer 2021semesters, anticipating potential improvements in testing, contact tracing, social distancing measures, as well as vaccine rollout to priority populations outside the universities, is therefore 3-6%.

The outbreaks of SARS-CoV-2 infection at college campuses have been reported to be associated with students residing in high density housing, socializing off campus, and not following social distancing guidelines (19). In the US, it is estimated that 45 percent of young adults aged 18-22 years were enrolled in colleges and universities in 2019 (16). As such, much of the increase in cases this summer and fall are attributed to college students. The college campuses are associated with a high rate of SARS-CoV-2 infection and resulting asymptomatic carriage due to huge impulse to socialize as well as no fear of severe disease among the young students.

2.6 Age and Asymptomatic Infection

For this study, participants will be enrolled who are 18 to 26 years of age. This age range is chosen because of the high incidence of asymptomatic infection in this population. A considerable body of data suggest that SARS-CoV-2 can be spread by individuals with and without symptoms (20-23). VL data among individuals with acute SARS-CoV-2 infection also indicate that peak VL usually occurs before disease onset (22, 24). This suggests that there is a substantial transmission potential before symptom onset. According to the latest Centers for Disease Control and Prevention (CDC) estimate 40% of SARS-CoV-2 infections are asymptomatic (25). Available data indicate that younger individuals appear more likely to remain asymptomatic following infection than older individuals (26, 27). A recent study done in the UK showed about 80% of children between 10-19 years of age remain asymptomatic (28). As infected young students are less likely to show clinical symptoms, the number of clinical cases reported among young populations would be lower, but the asymptomatic young students could still be capable of transmitting the virus to others (29).

2.7 Evidence for viral load as a predictor of infectiousness

Mathematical modelling in human immunodeficiency virus (HIV) and herpesviruses and now COVID-19 suggests a significant percentage of transmission occurs from high peak VL which in all these diseases is during the asymptomatic period. Models by Josh Schiffer and Ashish Goyal suggest that a person has a 50% chance of infection with exposure to 10^7 SARS-CoV-2 RNA and <5% chance of infection with exposure to 10^5 SARS-CoV-2 RNA (Figure 2-5) (21). The model by Goyal et al (21) suggests that vaccine reduction in peak VL may reduce infectiousness. A 0.6 log reduction corresponds to ~50% VE_I and a 2.5 log reduction to ~90% VE_I (Figure 2-6). This suggests collecting nasal swabs every other day would be required to measure the vaccine effect on peak VL and area under the VL curve. Asymptomatic and pre-symptomatic cases of SARS-CoV-2 appear to play an important role in the onward transmission of the virus.

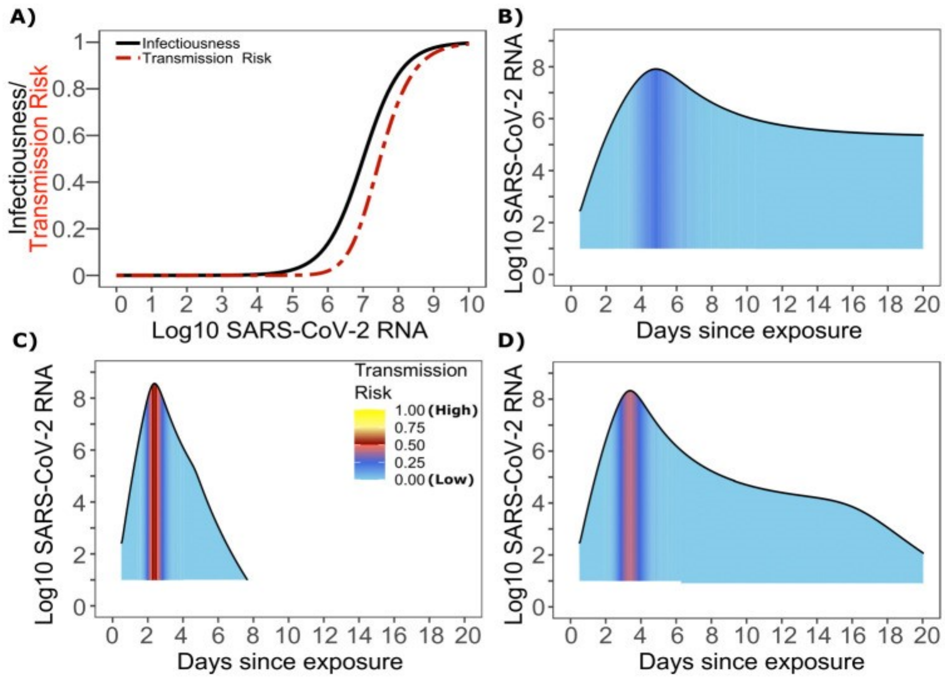

Figure 2-5 SARS-CoV-2 transmission probability as a function of shedding. A. Optimal infectious dose (ID) response curve (infection risk = Pt) and transmission dose (TD) response curve (transmission risk = Pt * Pt) curves for SARS-CoV-2. Transmission probability is a product of two probabilities, contagiousness and infectiousness. B-D. Three simulated viral shedding curves. Heat maps represent risk of transmission at each shedding timepoint given an exposed contact with an uninfected person at that time.

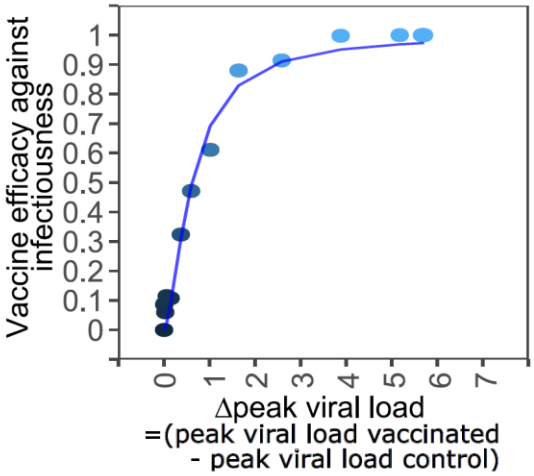

Figure 2-6 Vaccine efficacy against infectiousness versus the change in peak viral load. Model by Goyal et al (personal communication).

2.8 Evidence for viral load as a marker of COVID-19 disease severity and clinical course

Beyond infectiousness, there is a growing body of evidence supporting a linkage between VL and COVID-19 disease outcome. Two Phase 2/3 trials of monoclonal antibodies for COVID-19 treatment in outpatient settings found that VL at enrollment and early after intervention was associated with future clinical outcomes and disease course (30, 31). In the BLAZE-1 trial of intravenous infusion of the LY-CoV555 antibody, on day 7 post-infusion the frequency of hospitalization among patients with a high VL (Ct value of less than 27.5) was 12% (7 of 56 patients), as compared to 0.9% (3 of 340 patients) among those with a lower VL. Numerous observational studies have also identified associations between VL and clinical outcomes (32). The highest quality evidence comes from large cohort studies. In a large hospitalized cohort, Pujadas et al (33) estimated a highly significant 7% increase in mortality per log10 increase in VL at admission, after adjusting for other factors associated with clinical outcomes ($p = 0.0014$, multivariate Cox model). Chen et al (34) confirmed this finding in a larger cohort of patients admitted to three New York City hospitals. These data suggest that VL at and soon after the time of symptom onset may be a useful measure of disease severity.

2.9 Methods for capturing transmission

Reducing transmission is key to ending/controlling the COVID-19 pandemic. Due to vaccination, the number of asymptomatic individuals might increase as vaccine-induced immune responses may lead to VL reduction. However, it is still to be discovered whether those individuals will continue to shed and transmit virus to others. Hence, it is important to identify secondary transmissions from study participants to estimate secondary infections rates by arm and to validate VL as a surrogate for transmission risk. Onward transmission of

SARS-CoV-2 infection from study participants can be identified in combination with sequence-analysis and phylogenetics methods (35).

Estimating vaccine efficacy in reducing transmission (VE_I), requires measuring transmission of SARS-CoV-2 from vaccinated and unvaccinated individuals to others (36, 37). Measuring transmission of infected individuals to uninfected individuals is challenging, especially for transient infections where it may be more difficult to ascertain which infection occurred first. For example, because HIV is not transient and per-exposure transmission is low, resulting in a potentially long time period of exposure, transmission can be studied in sero-discordant sexual partnerships. In a transient infection, ideally both members of the transmission pair are monitored before either is infected so that timing and direction of transmission can be easily determined. Prospectively following a cohort of vaccinated and unvaccinated individuals and their close contacts to observe whether infection is transmitted from study participants to close contacts, or from contacts to study participants, based on frequent testing of both allows for better quantification of the temporality of events regarding infection transmission, if it occurs. To do this, Datta et al. propose augmenting a randomized, controlled vaccine trial with a cohort of close contacts of trial participants. Both trial participants and the contact cohort are followed prospectively for acquisition, allowing for estimation of VE_I. Another approach to capturing transmission events is a case ascertainment design (38, 39). Under this design, contacts of index cases are enrolled and tested as the cases emerge over time. A similar case ascertainment design was used for a recent household study for SARS-CoV-2 prevention. In the HCQ PEP trial, which was a household-randomized, double-blind, controlled trial of hydroxychloroquine postexposure prophylaxis to prevent acquisition of SARS-CoV-2 among close contacts recently exposed to persons diagnosed with SARS-CoV-2 infection, individuals were self-referred from infected individuals and enrolled online and all study procedures were done remotely including informed consent and daily self-nasal swabbing for 14 days (40). A case-ascertained approach is more resource-efficient than a prospective contact approach which requires enrollment of a large number of contacts, most of whom will not become infected nor have an infected index case in the main study. Because contacts are only identified and tested after index case diagnosis, the case-ascertained approach also enables assessment of secondary transmission in contacts not participating in frequent, prospective testing programs. However, the case-ascertained approach has the potential to miss or misclassify transmission events given the gap between diagnosing an index case and identifying, enrolling, and testing a contact. Index cases may also experience stigma when identifying contacts at the time of diagnosis, as opposed to when identifying contacts prospectively and before infection. The feasibility of recruiting and retaining university contacts, and of classifying transmission events among contacts, using the two designs has yet to be evaluated and compared.

This study will employ both the augmented and case ascertainment approaches to studying transmission through enrollment of two contact cohorts, a Prospective Close Contact (PCC) cohort and a Case-Ascertained Close Contact (CACC) cohort. The PCC Cohort will be identified at enrollment as close and sustained contacts of randomized participants (eg, roommates or co-workers). Participants in the PCC cohort will undergo routine screening for SARS-CoV-2 infection as part of their attendance or employment at their university, which will allow the participant and study team to receive the same real-time clinical diagnosis of

COVID-19 during the study as main study participants. Additionally, when a positive case is identified for a main study participant, the participant ('index case') will identify and refer their recent close contacts for enrollment into the CACC cohort. For both cohorts, infection diagnosis of the associated main study participant will trigger intensive short term sample collection (with a potential delay for the CACC cohort due to the need for informed consent and enrollment) including sera followed by daily nasal swabs for 14 days. Assessment of viral sequences, viral load trajectories, antibody detection and epidemiologic information from the infected participant and any positive close contacts in the PCC or CACC will aid in identifying transmission events between the participant and the contact. Details of the enrollment and procedures for both contact cohorts are described below.

2.10 Moderna COVID-19 Vaccine

This trial will utilize vaccine obtained under EUA by the US Government's COVID-19 Vaccine Response.

2.10.1 Clinical studies of Moderna COVID-19 Vaccine

Table 2-3 Summary of clinical studies

ClinicalTrials.gov Identifier	Study number	Phase	N	Dose groups	Route	Schedule	Reference
NCT04283461	P101	1	120	25, 50, 100, or 250 mcg	IM	M0, M1	(41, 42)
NCT04405076	P201	2	600	50 or 100 mcg	IM	M0, M1	(14, 15, 43)
NCT04470427	P301	3	30,351	100 mcg	IM	M0, M1	(14, 15, 43)

2.10.2 Potential risks of Moderna COVID-19 Vaccine

Risks associated with receipt of the Moderna COVID-19 Vaccine are described in the "Fact Sheet For Healthcare Providers Administering Vaccine (Vaccination Providers) Emergency Use Authorization (EUA) Of The Moderna COVID-19 Vaccine To Prevent Coronavirus Disease 2019 (COVID-19)", available at https://www.modernatx.com/covid19vaccine-eua/.

2.11 Trial design rationale

We are proposing a 2-arm randomized, controlled, open-label trial design. The design is augmented by two cohorts of contacts of study participants – the Prospective Close Contact

(PCC) cohort and the Case-Ascertained Close Contact (CACC) cohort – to allow capture of secondary transmission events.

Participants in the main study will self-collect nasal swabs daily in order to capture all incident SARS-CoV-2 infection events over 4 months of follow-up and to capture the full course of viral shedding – from onset of infection to viral clearance – among those with both asymptomatic and symptomatic infection.

To minimize participant burden and maximize study efficiency, as many study procedures as possible will be conducted remotely (not requiring an in-person visit) and electronically, such as initial screening and consent. Participants diagnosed with SARS-CoV-2 infection will complete daily e-diaries to capture symptoms.

Given that primary analyses will be conducted among baseline seronegative participants, the rate of baseline SARS-CoV-2 seropositivity will be monitored operationally with an operational target of no more than 10% for the main study cohort.

2.11.1 Dose and schedule

The latest information regarding Moderna COVID-19 Vaccine dose and administration instructions can be found at https://www.modernatx.com/covid19vaccine-eua/.

For this study, vaccine will be administered at D1 and D29 for the immediate vaccination arm, and at D113 and D141 for the delayed vaccination arm.

2.11.2 Choice of delayed vaccination

There is no licensed SARS-CoV-2 vaccine currently available to serve as a reference control. Currently, there are two SARS-CoV-2 vaccines available in the US under Emergency Use Authorization (EUA), including the Moderna COVID-19 Vaccine being used in this study. Accordingly, a delayed vaccination arm has been chosen as an internal control. No placebo will be used, and participants will not be blinded to randomization assignment. The open-label design has been chosen primarily to achieve operational efficiency and to minimize resource utilization.

With an open-label design, differences between the randomized arms in study endpoints including SARS-CoV-2 infection, viral load, and secondary transmission will capture not only biological effects of vaccination, but potentially behavioral effects influencing exposure, including adherence to study procedures. This is most relevant for the SARS-CoV-2 infection endpoint: if participants who are randomized to immediate vaccination engage in more risk-taking behavior and thus experience higher exposure to SARS-CoV-2 relative to participants randomized to delayed vaccination, ie, there is behavioral disinhibition, this will be captured in the estimate of vaccine efficacy against infection: the biological reduction in incidence of SARS-CoV-2 infection attributable to vaccination will be offset by an increase in incidence due to higher exposure. Therefore, the measure of vaccine efficacy against infection that is estimated in this study is in truth a measure of effectiveness. However, we retain the terminology "vaccine efficacy" for simplicity. Section 6.4.3.1 discusses this issue

in greater detail, including how the statistical analysis will attempt to control for potential imbalances in SARS-CoV-2 exposure between treatment arms when evaluating vaccine efficacy, and how both efficacy and effectiveness measures will be evaluated and compared. We note that endpoints measured after diagnosis of SARS-CoV-2 infection, including SARS-CoV-2 viral load and secondary transmission, are viewed as much less likely to be influenced by participant behavior and knowledge of vaccine receipt.

3 Objectives and endpoints

**Primary Objectives**	**Primary Endpoints**
1. To evaluate the efficacy of Moderna COVID-19 Vaccine against SARS-CoV-2 infection (ie, to evaluate vaccine efficacy against infection or VE_S).	SARS-CoV-2 infection diagnosed by PCR post visit 3 (month 1) among participants who were SARS-CoV-2 seronegative at enrollment and SARS-CoV-2 negative by PCR through visit 3 (month 1)
2. To evaluate the effect of Moderna COVID-19 Vaccine on peak nasal viral load as a measure of infection and a proxy of infectiousness	Peak viral load in nasal samples from participants diagnosed with SARS-CoV-2 infection post visit 3 (month 1), among participants who were SARS-CoV-2 seronegative at enrollment and SARS-CoV-2 negative by PCR through visit 3 (month 1)
**Secondary Objectives**	**Secondary Endpoints**
1. To evaluate the impact of Moderna COVID-19 Vaccine on secondary transmission of SARS-CoV-2 infection, using data for the Prospective Close Contact (PCC) cohort and Case-Ascertained Close Contact (CACC) cohort (ie, to evaluate vaccine efficacy against infectiousness or VE_I)	Number of adjudicated* secondary transmission events in PCC and CACC cohorts from main study participants who were SARS-CoV-2 seronegative at enrollment and SARS-CoV-2 negative by PCR through visit 3 (month 1).<br>*based on questionnaire-based epidemiologic data, antibody and viral dynamics, and viral sequence data
2. To evaluate the efficacy of Moderna COVID-19 Vaccine to prevent serologically confirmed SARS-CoV-2 infection	SARS-CoV-2 antibodies to the nucleocapsid protein post visit 3 (month 1), among participants who were SARS-CoV-2 seronegative at enrollment and SARS-CoV-2 negative by PCR through visit 3 (month 1)
3. To evaluate vaccine efficacy against COVID-19 disease, (ie, to evaluate VE_D) using weekly e-diaries and confirmatory PCR testing	SARS-CoV-2 infection confirmed by PCR and diagnosed post-visit 3 (month 1), among participants SARS-CoV-2 who were seronegative at enrollment and SARS-CoV-2 negative by PCR through visit 3 (month 1), coincident with the following symptoms:<br>• at least TWO of the following systemic symptoms: Fever (≥ 38ºC), chills, myalgia, headache, sore throat,<br>OR<br>at least ONE of the following signs/symptoms: cough, shortness of breath or difficulty breathing, new olfactory or taste disorder, clinical or radiographical evidence of pneumonia, thromboembolic event, myocardial infarction, myocarditis, chilblains, or multi-inflammatory syndrome
4. To evaluate vaccine effect on additional measures of magnitude of viral load over time,	Summary measures of the viral load curve, all evaluated among participants diagnosed with SARS-

e.g. area under the viral load curve, duration of shedding, and time to log10 viral load above 10^5 copies/mL	CoV-2 infection post-visit 3 (month 1) who were SARS-CoV-2 seronegative at enrollment and SARS-CoV-2 negative by PCR through visit 3 (month 1):<br>• Transmission potential endpoint, defined as 0 for an individual without SARS-CoV-2 infection, and peak log10 viral load, for an individual with SARS-CoV-2 infection<br>• Area under log10 viral load curve (AUC)<br>• Area under log10 viral load curve above 10^5 copies/mL<br>• Time from enrollment to viral load above 10^5 copies/mL
5. To evaluate vaccine efficacy against SARS-CoV-2 infection, the vaccine effect on viral load, and the vaccine effect on secondary transmission in all enrolled participants without regard to baseline SARS-CoV-2 serostatus	• SARS-CoV-2 infection diagnosed by PCR [Primary endpoint 1]<br>• Peak log10 viral load in nasal samples from participants who are diagnosed with SARS-CoV-2 infection [Primary endpoint 2]<br>• Number of secondary transmission events in the PCC and CACC [Secondary endpoint 1]
6. To evaluate the immunogenicity of the vaccine	Magnitude and response rate of immune responses to vaccination measured at Months 0, and 2 stratified by baseline SARS-CoV-2 serostatus as measured by binding antibody and neutralization assays in the case-cohort immunogenicity set
7. To evaluate immune responses 1 month post-second vaccination as correlates of risk of SARS-CoV-2 acquisition, viral load, secondary infection, and COVID-19 disease among vaccine recipients in the immediate vaccination group	Magnitude and response rate of immune responses to vaccination in the immediate vaccination group and measured at Months 0 and 2, stratified by baseline SARS-CoV-2 serostatus, as measured by binding antibody and neutralization assays in the case-cohort immunogenicity set
8. To evaluate vaccine efficacy against asymptomatic SARS-CoV-2 infection	SARS-CoV-2 infection by PCR or periodic serology post-visit 3 (month 1) among participants seronegative for SARS-CoV-2 at baseline, SARS-CoV-2 negative by PCR through visit 3 (month 1), and without any prior reporting of symptoms that led to confirmation of a COVID-19 disease endpoint
9. To evaluate vaccine efficacy against SARS-CoV-2 infection and COVID-19 disease and the vaccine effect on viral load and secondary transmission among participants who received all planned immunizations at the designated immunization visits within specific visit windows	Endpoints evaluated among participants who were SARS-CoV-2 seronegative at enrollment and SARS-CoV-2 negative by PCR through visit 3 (month 1) and

	counting SARS-CoV-2 infection events post-visit 3 (month 1)<br>• SARS-CoV-2 infection diagnosed by PCR [Primary endpoint 1]<br>• Peak log10 viral load in nasal samples from participants who are diagnosed with SARS-CoV-2 infection [Primary endpoint 2]<br>• Number of secondary transmission events in the PCC and CACC [Secondary endpoint 1]<br>• SARS-CoV-2 infection coincident with signs and symptoms of COVID-19 listed under Secondary endpoint 3
10. To evaluate the association between measures of the magnitude of viral load over time and secondary transmission of SARS-CoV-2 infection, among individuals with incident SARS-CoV-2 infection in each treatment group, i.e. to conduct analyses aimed at evaluating viral load measures as surrogates of secondary transmission	• Peak log10 viral load [Primary endpoint 2] and other viral load summaries [Secondary endpoints 4]<br>• Number of secondary transmission events in the PCC cohort and CACC cohort [Secondary endpoint 1]
11. To evaluate vaccine effects on peak viral load and on secondary transmission of SARS-CoV-2 infection separately among individuals with asymptomatic vs. symptomatic SARS-CoV-2 infection	• Peak log10 viral load [Primary endpoint 2] and other viral load summaries [Secondary endpoints 4]<br>• Number of secondary transmission events in the PCC cohort and CACC cohort [Secondary endpoint 1]<br>• SARS-CoV-2 infection diagnosed using PCR, with and without signs and symptoms of COVID-19 listed under Secondary endpoint 3

Exploratory Objectives

1. To evaluate the associations between demographic and clinical factors and secondary transmission of SARS-CoV-2 infection and COVID-19 disease, among individuals with incident SARS-CoV-2 infection in the delayed vaccination arm and their contacts in the PCC and CACC
2. To assess if and how vaccine efficacy against SARS-CoV-2 infection, transmission and COVID-19 disease depends on baseline demographic, clinical, socio-economic indicators, and/or behavioral characteristics of study participants
3. To assess if and how vaccine efficacy against SARS-CoV-2 infection and COVID-19 disease depends on genotypic or neutralization phenotypic characteristics of SARS-CoV-2 (sieve analysis)
4. To further evaluate immunogenicity of the vaccine, additional immunogenicity assays may be performed such as antibody (Ab) Avidity and Fc Receptor function assays, based on the CoVPN Laboratory Center assay portfolio
5. To conduct analyses related to furthering the understanding of SARS-CoV-2, immunology, vaccines, and clinical trial conduct

4 Ethical considerations

It is critical that universally accepted ethical guidelines are followed at all sites involved in the conduct of clinical trials. The COVID-19 Prevention Network (CoVPN) have addressed ethical concerns in the following ways:

- CoVPN trials are designed and conducted to enhance the scientific knowledge base necessary to find a preventive vaccine, using methods that are scientifically rigorous and valid, and in accordance with International Council for Harmonisation of Technical Requirements for Pharmaceuticals for Human Use (ICH) and/or other Good Clinical Practice (GCP) guidelines.

- CoVPN scientists and operational staff incorporate the philosophies underlying major codes (44-46), declarations, and other guidance documents relevant to human subjects research into the design and conduct of SARS-CoV-2 vaccine clinical trials.

- CoVPN scientists and operational staff are committed to substantive community input—into the planning, conduct, and follow-up of its research—to help ensure that locally appropriate cultural and linguistic needs of study populations are met.

- The CoVPN provides training so that all participating sites similarly ensure fair participant selection, protect the privacy of research participants, and obtain meaningful informed consent. During the study, participants will have their wellbeing monitored, and to the fullest extent possible, their privacy protected. Participants may withdraw from the study at any time.

- Prior to implementation, CoVPN trials are rigorously reviewed by scientists who are not involved in the conduct of the trials under consideration.

- CoVPN trials are reviewed by local and national regulatory bodies and are conducted in compliance with all applicable national and local regulations.

- The CoVPN designs its research to minimize risk and maximize benefit to both study participants and their local communities. For example, CoVPN protocols provide enhancement of participants' knowledge of SARS-CoV-2 and SARS-CoV-2 prevention, as well as counseling, guidance, and assistance with any social impacts that may result from research participation. CoVPN protocols also include careful medical review of each research participant's health conditions and reactions to study products while in the study.

- CoVPN research aims to benefit local communities by directly addressing the health and SARS-CoV-2 prevention needs of those communities and by strengthening the capacity of the communities through training, support, shared knowledge, and equipment. Researchers involved in CoVPN trials are able to conduct other critical research in their local research settings.

- The CoVPN values the role of in-country Institutional Review Boards (IRBs), Ethics Committees (ECs), and other Regulatory Entities (REs) as custodians responsible for ensuring the ethical conduct of research in each setting.

5 IRB/EC review considerations

US Food and Drug Administration (FDA) and other US federal regulations require IRBs/ECs/REs to ensure that certain requirements are satisfied on initial and continuing review of research (Title 45, Code of Federal Regulations (CFR), Part 46.111(a) 1-7; 21 CFR 56.111(a) 1-7). The following section highlights how this protocol addresses each of these research requirements. Each CoVPN Investigator welcomes IRB/EC/RE questions or concerns regarding these research requirements.

5.1 Minimized risks to participants

45 CFR 46.111 (a) 1 and 21 CFR 56.111 (a) 1: Risks to subjects are minimized.

This protocol minimizes risks to participants by (a) correctly and promptly informing participants about risks so that they can join in partnership with the researcher in recognizing and reporting harms; (b) respecting local/national blood draw limits; (c) performing direct observation of participants postvaccination and reporting information regarding side effects to the FDA/CDC Vaccine Adverse Event Reporting System (VAERS) per EUA guidance; (d) having staff properly trained in administering study procedures that may cause physical harm or psychological distress, such as blood draws, vaccinations, COVID-19 testing and counseling; and (e) providing safety monitoring.

5.2 Reasonable risk/benefit balance

45 CFR 46.111(a) 2 and 21 CFR 56.111(a) 2: Risks to subjects are reasonable in relation to anticipated benefits, if any, to subjects, and the importance of the knowledge that may reasonably be expected to result.

In all public health research, the risk-benefit ratio may be difficult to assess because the benefits to participants in prevention protocols are not as apparent as they would be in treatment protocols, where a study participant may be ill and may have exhausted all conventional treatment options. However, this protocol is designed to minimize the risks to participants while maximizing the potential value of the knowledge it is designed to generate.

5.3 Equitable participant selection

45 CFR 46.111 (a) 3 and 21 CFR 56.111 (a) 3: Subject selection is equitable

This protocol has specific inclusion and exclusion criteria for investigators to follow in admitting participants into the protocol. Participants are selected because of these criteria and not because of positions of vulnerability or privilege. Investigators are required to maintain screening and enrollment logs to document volunteers who screened into and out of the protocol and for what reasons.

5.4 Appropriate informed consent

45 CFR 46.111 (a) 4 and 5 and 21 CFR 56.111 (a) 4 and 5: Informed consent is sought from each prospective subject or the subject's legally authorized representative as required by 45 CFR 46.116 and 21 CFR Part 50; informed consent is appropriately documented as required by 45 CFR 46.117 and 21 CFR 50.27

The protocol specifies that informed consent must be obtained before any study procedures are initiated and assessed throughout the trial (see Section 9.1). Each site is provided training in informed consent by the CoVPN and is required to have a Standard Operating Procedure (SOP) on the informed consent process. The CoVPN requires a signed consent document for documentation, in addition to chart notes or a consent checklist.

5.5 Adequate safety monitoring

45 CFR 46.111 (a) 6 and 21 CFR 56.111 (a) 6: There is adequate provision for monitoring the data collected to ensure the safety of subjects.

Vaccination providers are required to report to VAERS the certain adverse events after COVID-19 vaccination, under Emergency Use Authorization (EUA) and encouraged to report adverse events to ModernaTX |(https://www.cdc.gov/vaccines/covid-19/vaccination-provider-support.html, see Section 11). Additionally, the use of v-safe will be recommended to vaccine recipients (https://www.cdc.gov/coronavirus/2019-ncov/vaccines/safety/vsafe.html, see Section 11), and an Independent Data Monitoring Committee (IDMC) will periodically review study data.

5.6 Protect privacy/confidentiality

45 CFR 46.111 (a) 7 and 21 CFR 56.111 (a) 7: There are adequate provisions to protect the privacy of subjects and maintain the confidentiality of data.

Privacy refers to an individual's right to be free from unauthorized or unreasonable intrusion into his/her private life and the right to control access to individually identifiable information about him/her. The term "privacy" concerns research participants or potential research participants as individuals whereas the term "confidentiality" is used to refer to the treatment of information about those individuals. This protocol respects the privacy of participants by informing them about who will have access to their personal information and study data (see Appendix A, Appendix B and Appendix C). The privacy of participants is protected by assigning unique identifiers in place of the participant's name on study data and specimens. In the United States, research participants in CoVPN protocols are protected by a Certificate of Confidentiality from the US NIH, which can prevent disclosure of study participation even when that information is requested by subpoena. Participants are told of the use and limits of the certificate in the study consent form. In addition, each staff member at each study site in this protocol signs an Agreement on Confidentiality and Use of Data and Specimens with the

CoVPN. In some cases, a comparable confidentiality agreement process may be acceptable. Each study site participating in the protocol is required to have a standard operating procedure on how the staff members will protect the confidentiality of study participants.

6 Statistical considerations

We anticipate enrolling approximately 12,000 students. Participants will be randomized 1:1 to immediate or delayed vaccination, at Months 0 and 1 or Months 4 and 5, respectively. Randomization will be stratified by place of residence, for each university.

Primary analyses of vaccine efficacy against infection (VE_S) and of the vaccine effect on viral load (ΔVL) will be conducted among participants SARS-CoV-2 seronegative at enrollment in the "Day 29 Negative Set", i.e. the set of participants without PCR-confirmed infection on or prior to Day 29; see Table 6-1 for a full definition of the various analysis sets for the study. Primary efficacy analyses will only count SARS-CoV-2 infection events diagnosed post-Day 29. Secondary analyses will be conducted among all participants who receive both immunizations without protocol violation, i.e. the Per-Protocol Set, and in the set of all enrolled participants (the "Full Analysis Set" or FAS), counting infection events diagnosed after enrollment (Day 0). Immunogenicity assessment will be done in the Case-Cohort Immunogenicity Set—a subsample of the participants randomized to immediate vaccination and sampled on the basis of SARS-CoV-2 infection status and baseline characteristics that will be fully described in the study SAP.

The FAS differs from a full Intention-to-Treat set by excluding randomized volunteers who do not enroll. The exclusion of these individuals is justified because the effects of the vaccine are unlikely to influence such exclusion. Because of the brief length of time between randomization and enrollment (ideally these will be on the same day), we expect almost all randomized volunteers to be in the FAS.

All primary and secondary analyses will be done according to the treatment randomly assigned ("as randomized").

While main study participants will be tested using both the real-time university testing system and using PCR testing of stored swabs, it is the latter that will be used to identify SARS-CoV-2 infection events for primary analysis.

The two contact cohorts – the PCC and the CACC-- will both be used for analyses of the vaccine effect on secondary transmission, as described below.

The study is "time-driven", i.e. the primary analysis will occur when all participants have completed the 5 months follow-up (with 4 months/16 weeks of primary follow-up and a final 5 month contact). The number of primary endpoint infection events that accrue in the study is random and will depend on the level of SARS-CoV-2 incidence in each arm.

Table 6-1 Study Analysis Sets and Definitions

Analysis Set	Definition	Analyses
Full Analysis Set (FAS)	Randomized participants who enroll in the study	Secondary efficacy analyses*
Day 29 Negative (D29-) Set	Participants in the FAS without PCR-confirmed infection on or before Day 29	Primary efficacy analyses*
Per-Protocol (PP) Set	Participants in the D29- set who received all planned immunizations at the designated immunization visits within specific visit windows	Secondary efficacy analyses*
Day 29 Negative Transmission Set (D29-TS)	Participants in the D29- set and their contacts in the Prospective and Case Ascertained Contact Cohorts	Primary analyses of secondary transmission*
Case-Cohort Immunogenicity Set (ccIS)	Subset of the FAS that is randomly sampled for measurement of immunological markers and fully described in study SAP	Immunology marker analyses

*These analysis sets include participants without regard to baseline SARS-CoV-2 status. However, primary efficacy analyses will condition on baseline SARS-CoV-2 seronegative status.

Table 6-2 Assumptions underlying study power/sample size calculations, unless otherwise specified. Parameters and their assumed values pertain to participants in the main study cohort.

Parameter	Assumption
Enrollment	Uniform enrollment over 4 weeks
Baseline seroprevalence in main study and PCC and CACC	10% of enrolled participants are baseline SARS-CoV-2 seropositive. This parameter is monitored and controlled operationally (see Section 6.3)
Per-protocol rate	98% of enrolled participants are in the per-protocol set
Loss to follow-up rate in the main study, the PCC and the CACC	5% of participants are lost to follow-up over 16 weeks at a constant rate
SARS-CoV-2 incidence in the delayed vaccination group and incidence un-linked to main study participants in the PCC	Exponential (constant rate) over 16 weeks; primary assumption is 4% over 16 weeks
Secondary transmission rate among susceptible contacts, in the delayed vaccination arm	15% transmission rate based on (47-49)
Vaccine efficacy (VE) against SARS-CoV-2 infection and transmission	Constant after 14 days post-first vaccination (calculations do not depend on earlier VE); level is varied
Contact enrollment	For the PCC: 60% of main study participants enroll contact(s) and the average number of contacts enrolled is 2<br>For the CACC: 75% of main study participants enroll contact(s) and the average number of contacts enrolled is 3

6.1 Accrual and sample size calculations

A sample size of 6,000 participants in each treatment arm ensures a high probability that a sufficient number of primary endpoint events, i.e. SARS-CoV-2 infections among baseline seronegative participants in the D29- set and diagnosed after Day 29, will accrue to adequately power analyses addressing both primary objectives as well as key secondary objectives. Specifically, under a 4% SARS-CoV-2 incidence over 16 weeks in the delayed vaccination group (a conservative assumption given recent incidence on US college campuses, see Section 2.5), assuming VE_S = 50% and other assumptions laid out in Table 6-2, 225 primary endpoint events will occur across both arms with probability 0.5 and 214 events will occur with probability 0.8.

Table 6-3 shows the expected number of primary endpoint events, overall and for each treatment group, as the assumed incidence in the delayed vaccination group varies. As shown, the design ensures a sufficient number of primary endpoint events to address a variety of objectives that condition on the primary endpoint. For example, with 4% SARS-CoV-2 incidence in the delayed vaccination group, 75 primary endpoint events are expected in the immediate vaccination group for immune correlates analyses.

Table 6-3 Expected number of primary endpoint events, available for secondary analyses that condition on primary endpoint event status. Calculations assume 6,000 participants per arm and other assumptions in **Table 6-2**, VE_S = 50%, and varying 16-week incidence of SARS-CoV-2 infection in the delayed vaccination group.

16-Week SARS-CoV-2 Incidence in Placebo Arm	Number of Primary Endpoint Events	
	Both Arms	Immediate Vaccination Arm
1.5%	86	28
2%	143	47
3%	170	56
3.5%	198	66
4%	225	75

The study provides high power to evaluate the vaccine effect on acquisition (VE_S). Figure 6-1 shows the power of the study to detect a given level of VE_S, rejecting H_0: $VE_S \leq 30\%$, based on a 1-sided 0.025-level log-rank test and as a function of the SARS-CoV-2 incidence in the delayed vaccination group. With 4% 16-week incidence, the study has 90% power to detect any $VE_S > 57\%$; with 3% incidence, $VE_S > 60\%$ can be detected with 90% power.

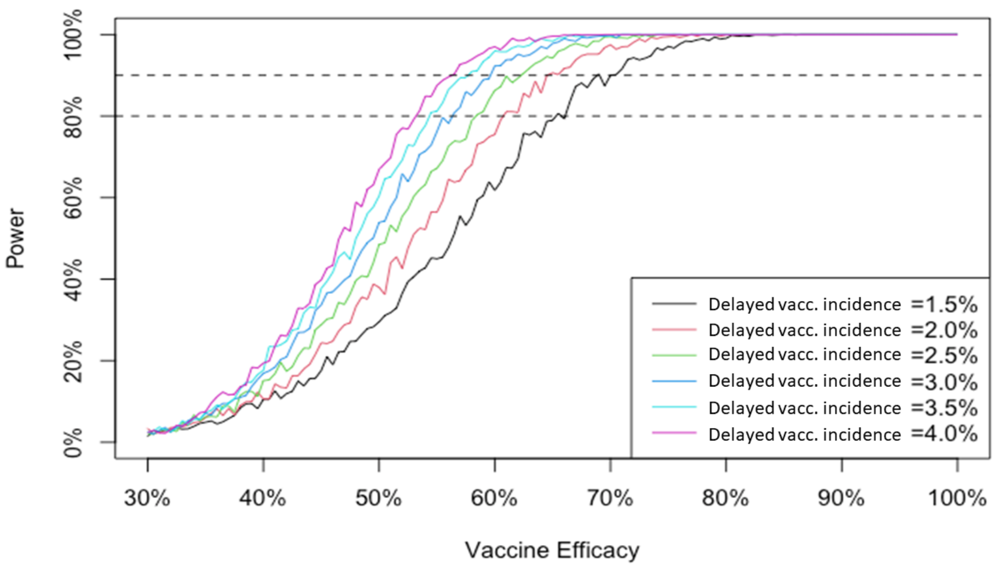

Figure 6-1 Power to detect varying vaccine efficacy (VE) against SARS-CoV-2 infection as a function of the delayed vaccination group incidence over 16 weeks. Simulation-based power calculations assume 6,000 participants in each arm and other assumptions listed in **Table 6-2**. Power based on 1-sided 0.025-level log-rank test and 1,000 simulations.

The proposed study also provides adequate precision with which to estimate VE_S. Table 6-4 shows that, with 225 primary endpoint events, the approximate 95% confidence interval (CI) about an estimated 50% VE_S is (36.9%, 60.4%). More precise inference is obtained under higher VE_S and higher primary endpoint totals.

Table 6-4 Approximate 95% confidence interval around a given estimated VE_S against SARS-CoV-2 infection, comparing the immediate vaccination arm vs. the delayed vaccination arm, as a function of the number of primary endpoint events across the two groups.

Number of primary endpoint events	Approximate 95% CI for estimated VE = 50%	Approximate 95% CI for estimated VE = 70%
200	(36.0%, 60.9%)	(60.7%, 77.2%)
225	(36.9%, 60.4%)	(61.1%, 76.9%)
250	(37.7%, 59.9%)	(61.6%, 76.6%)

6.1.1 Power and precision for evaluating the vaccine effect on VL

Emerging data on acute SARS-CoV-2 infection suggest that with an expected 225 primary endpoint events, the vaccine effect on mean peak log10 viral load among primary endpoint infections can be estimated with adequate precision. Specifically, assuming that peak log10 viral load is normally distributed with a 1.8 standard deviation in both treatment groups, and assuming 50% VE_S, a 1-log reduction in mean peak viral load can be detected with 96.9% power given 225 primary endpoint events; power remains high (93.5%) with 70% VE_S. Table 6-5 shows power to detect a 1-log reduction in peak log viral load as a function of the assumed standard deviation in log peak viral load which is allowed to vary between 1.0 and

2.5. The standard deviation in peak log viral load in a cohort of 68 individuals associated with the National Basketball Association who were diagnosed with acute SARS-CoV-2 infection was 1.4 to 1.8, depending on the set of samples included in the analysis (50). Emerging data from the HCQ-PEP study of ~100 acutely infected household members of recently diagnosed COVID-19 cases suggests a standard deviation in peak log viral load in the range of 2.0-2.2, depending on the set of samples included [data not published]. Table 6-6 shows the approximate 95% CI about an estimated 1-log reduction in mean peak log viral load, demonstrating the precision that is obtained with 225 primary endpoint events. Modest precision is observed with a 1-1.8 standard deviation in the endpoint, with lower precision under a higher standard deviation; precision is also lower under increasing VE_S.

Table 6-5 Power to detect a 1-log reduction in mean peak log viral load comparing primary endpoint infection events between the immediate and delayed vaccination groups, as a function of the assumed standard deviation in peak log viral load in both groups and the assumed level of VE against SARS-CoV-2 infection. Simulation-based power calculations assume 225 primary endpoint events across treatment groups. Power based on 1-sided 0.025-level t-test and 1,000 simulations.

Std. dev. in peak log viral load	VE_S = 50%	VE_S = 70%
**1**	100%	100%
**1.5**	99.6%	97.8%
**1.8**	96.9%	93.5%
**2**	95.6%	86.5%
**2.5**	79.9%	68.7%

Table 6-6 Approximate 95% confidence interval around an estimated 1-log reduction in mean peak log viral load comparing primary endpoint infection events between immediate and delayed vaccination groups, as a function of the assumed standard deviation in peak log viral load in both groups and the assumed level of vaccine efficacy against SARS-CoV-2 infection. Calculations assume 225 primary endpoint events across treatment groups.

Std. dev. in peak log viral load	VE_S = 50%	VE_S = 70%
**1**	(0.72, 1.28)	(0.68, 1.31)
**1.5**	(0.57, 1.41)	(0.51, 1.46)
**1.8**	(0.50, 1.50)	(0.44, 1.57)
**2**	(0.46, 1.57)	(0.36, 1.61)
**2.5**	(0.29, 1.65)	(0.20, 1.74)

6.1.2 Power for evaluating the vaccine effect on secondary transmission

The study has adequate power to evaluate vaccine efficacy against secondary transmission, based on analyses that are conducted among primary endpoint cases and their contacts in the PCC and CACC, i.e. based on conditional analyses, as long as VE against SARS-CoV-2 infection is low. As demonstrated in Figure 6-2, if there is high vaccine efficacy against SARS-CoV-2 infection, power is low to evaluate secondary transmission using a conditional analysis. In this case, an unconditional analysis of VE against secondary transmission is

expected to be more powerful, and also more appropriate given that it reflects that infections blocked have no transmission potential.

We performed simulation-based calculations to explore the power to evaluate VE against secondary transmission based on a conditional analysis. In addition to the assumptions in Table 6-2, we assumed: equal infection surveillance in the PCC and CACC, for simplicity; that PCC contacts acquire infection from outside sources (other than the index case) at the same rate as the delayed vaccination arm; that only baseline seronegative contacts and those in the PCC not infected by outside sources are at risk of secondary transmission; that the number of secondary transmission events among at-risk contacts follows a Poisson distribution in each arm, with VE against secondary transmission reducing the event rate proportionally in the immediate vaccination group. A one-sided 0.025-level likelihood ratio test based on a Poisson regression model was used to evaluate VE against secondary transmission. As shown in Figure 6-2, the study has 90% power to detect a 59% reduction in secondary transmission if VE_S = 50%; a higher 69% reduction in secondary transmission is detectable if VE_S = 70%. Table 6-7 helps explain the power results: it shows the amount of statistical information on which the analysis is based, ie, the median number of primary endpoint events and secondary endpoint events per arm. With 50% VE_S and 50% VE against secondary transmission, we expect 15 secondary transmission events in the immediate vaccination arm and 59 in the delayed arm; the limited number of events is due to the "transmission cascade" whereby under our assumptions only some primary endpoint events enroll contacts into the PCC and CACC, only some contacts are susceptible and retained, and the transmission rate is low (15%).

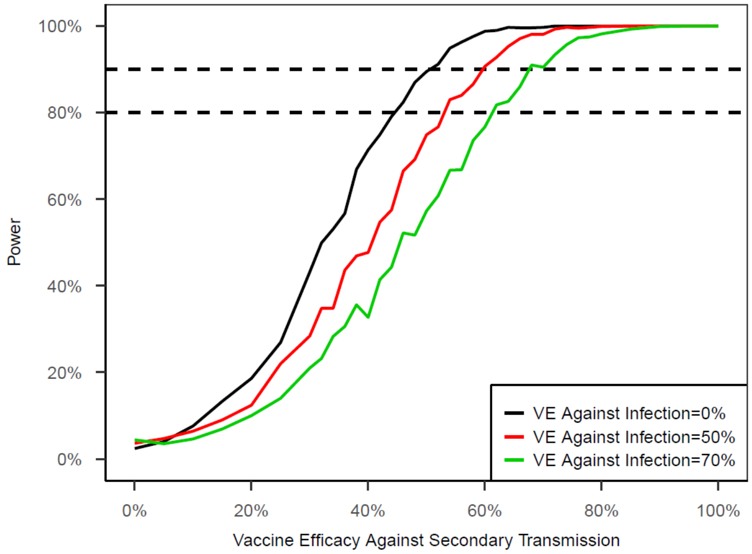

Figure 6-2 Power to detect varying vaccine efficacy against secondary transmission as a function of vaccine efficacy against SARS-CoV-2 infection. Simulation-based power calculations assume 6,000 participants in each arm and other assumptions listed in **Table 6-2**. Power based on 1-sided 0.025-level likelihood ratio test under Poisson regression model and 1,000 simulations.

Table 6-7 Number of primary endpoint events and secondary transmission events in each arm, under varying VE against SARS-CoV-2 infection and varying VE against secondary transmission, under other assumptions laid out in Table 6-2, and based on 1,000 simulations.

	**VE_S = 50%**		**VE_S = 70%**	
VE against secondary transmission	Median no. primary endpoints, Immediate vs. Delayed arms (IQR)	Median no. secondary transmissions, Immediate vs. Delayed arms (IQR)	Median no. primary endpoints, Immediate vs. Delayed arms (IQR)	Median no. secondary transmissions, Immediate vs. Delayed arms (IQR)
VE = 0%	76 (70-82) vs. 150 (142-158)	29 (25-34) vs. 59 (53-66)	45 (41-50) vs. 149 (141-158)	18 (14-21) vs. 59 (53-65)
VE = 40%	76 (70-81) vs. 150 (142-158)	18 (15-21) vs. 59 (53-64)	45 (41-50) vs. 149 (142-158)	11 (8-13) vs. 59 (53-65)
VE = 50%	76 (70-82) vs. 150 (143-159)	15 (12-17) vs. 59 (53-66)	45 (41-50) vs. 150 (142-158)	9 (7-11) vs. 59 (53-65)
VE = 60%	76 (70-82) vs. 150 (142-158)	12 (9-14) vs. 58 (52-65)	45 (41-50) vs. 151 (143-159)	7 (5-9) vs. 59 (53-65)
VE = 70%	75 (70-81) vs. 150 (142-158)	9 (7-11) vs. 58 (52-65)	46 (41-50) vs. 150 (142-159)	5 (4-7) vs. 59 (53-65)

6.2 Role of the Independent Data Monitoring Committee (IDMC)

The study IDMC will provide study oversight and ensure that the study is on-target to meeting its objectives in a timely fashion. The IDMC will receive summaries of study progress throughout the study. Given the short duration of the study and the considerable prior data establishing safety and efficacy of the vaccine, and the status of the EUA vaccine, there will be no pre-specified interim monitoring boundaries for VE or non-efficacy. The IDMC will make recommendations to protocol chairs and lead advisors. The set of metrics, operational targets for them, and potential remedial actions will be laid out in full in the IDMC Charter.

6.3 Randomization

A participant's randomization assignment will be computer generated and provided to the CRS study staff through a Web-based randomization system.

Randomization will be stratified. The randomization will be done in blocks to ensure balance among contemporaneously evaluated vaccine and control arms within levels defined by place of residence.

6.4 Statistical analyses

This section describes the final study analyses which will occur after the last participant has completed the Month 4 visit. All data from enrolled participants will be analyzed according to the initial randomization assignment regardless of how and when vaccinations are received. In the rare instance that a participant receives vaccination at the wrong time, the Statistical Analysis Plan (SAP) will address how to analyze the data. Analyses are modified intent-to-treat in that individuals who are randomized but not enrolled do not contribute data

and hence are excluded. Because of the brief length of time between randomization and enrollment—typically these will happen on the same day—very few such individuals are expected.

Analyses for primary endpoints will be performed using SAS and R. All other descriptive and inferential statistical analyses will be performed using SAS, StatXact, or R statistical software.

In general, no formal multiple comparison adjustments will be employed for multiple secondary endpoints. However, multiplicity adjustments will be made for certain immunogenicity assays, as discussed below, when the assay endpoint is viewed as a collection of hypotheses (eg, testing multiple peptide pools to determine a positive response).

6.4.1 Analysis variables

The analysis variables defined for all participants in the main study cohort consist of baseline participant characteristics, longitudinal measures of risk of infection, SARS-CoV-2 infection and seroconversion and COVID-19 disease status, and immune response biomarkers. For individuals diagnosed with SARS-CoV-2 infection, post-infection variables will include various summaries of VL, the full genome sequence of the infecting virus, and the infection status of and relationship to each of their contacts enrolled in the PCC and CACC. For contacts enrolled in the PCC and CACC, variables consist of baseline participant characteristics, SARS-CoV-2 infection and seroconversion status, risk factors around time of exposure to main study participant (if infected) and relationship to the referring main study participant.

6.4.2 Baseline comparability and comparability over study follow-up

Treatment arms will be compared with respect to baseline participant characteristics using descriptive statistics.

Given that the study is open-label, and the associated potential for differences between arms with respect to retention, compliance with study procedures, ascertainment of endpoints, and variables associated with exposure to SARS-CoV-2, these measures will be compared between treatment arms over time using methods fully described in the SAP. Imbalances between arms will influence the analysis choices as described below.

6.4.3 Vaccine efficacy (VE) analyses against SARS-CoV-2 infection

The time origin for all analyses of SARS-CoV-2 infection is time of enrollment (ie, Day 0). We define the time of diagnosis of the SARS-CoV-2 infection endpoint as the collection time of the first daily swab with a positive PCR test. The time of diagnosis of lab-confirmed clinical COVID-19 disease is the first time of reported qualifying COVID-19 symptoms that occurs simultaneously or after the diagnosis of the SARS-CoV-2 infection endpoint. The time of diagnosis of asymptomatic SARS-CoV-2 infection is the collection time of the first sample with a positive PCR test result or seroconversion without any prior reporting of symptoms that led to confirmation of a COVID-19 disease endpoint. The time of diagnosis of

SARS-CoV-2 seroconversion is the collection time of the first sample seropositive for SARS-CoV-2, for participants SARS-CoV-2 seronegative at baseline; the SAP will specify criteria for defining seroconversion (re-infection) for individuals SARS-CoV-2 seropositive at baseline. The failure times of participants never observed to be diagnosed with a given clinical endpoint will be right-censored at the date of last contact at which endpoint status was assessed or study termination.

6.4.3.1 Primary VE Analysis

For the primary analysis, Cox proportional hazards regression will be used to estimate vaccine efficacy against SARS-CoV-2 infection among baseline SARS-CoV-2 seronegative participants in the D29- set, counting only infection events diagnosed post-visit-3 (month 1), as measured by one minus the hazard ratio (HR) in the immediate vs. delayed vaccination arms. Given that the study is open-label, the primary analysis will adjust for an SAP-specified set of potentially time-varying "risk" variables, or a composite risk score based on them, that are potentially associated with exposure including demographics and behaviors captured using the risk assessment tool. Inference will be based on a 2-sided score-based CI and 2-sided score-test for H0: VE = 0% with a type 1 error rate of 0.05, although primary interest is in estimation as opposed to hypothesis testing and the study is designed to ensure that the lower limit for the CI will lie above 30% with high probability under VE > 60%.The baseline hazard function in the Cox model will be stratified by study site and randomization stratum, which improves precision of the VE estimate and accounts for the fact that SARS-CoV-2 infection incidence may vary widely across study sites. Description of the risk variables under consideration and of the derivation of a risk score based on them will be described in the study SAP.

The potential impacts of the open-label aspect of the study design will be factored into the choice of efficacy analysis. If there is evidence of differential retention of study participants across arms, or differential adherence to study procedures affecting ascertainment of endpoints such as collection of daily swabs or participation in the university testing system, the analysis will adjust for the differential censoring distribution across arms- as well as potentially informative censoring- as described in the study SAP. At a minimum, these analyses will be performed as sensitivity analyses, complementing the primary analysis described above.

Variables captured using the risk assessment tool and potentially associated with exposure will be compared across arms over study follow-up. If there is evidence that their distributions differ between arms over time, sensitivity analysis will additionally adjust for time-dependent exposure-related variables.

The proportional hazards assumption will be evaluated using methods of Grambsch and Therneau (51).

A sensitivity analysis will be conducted to account for the possibility that the university testing programs may capture infections missed in the study swabbing; the primary analysis of VE against SARS-CoV-2 infection will be repeated using all available PCR test results from both daily swabs conducted as part of this protocol and the university testing system.

6.4.3.2 Secondary VE Analyses

The primary analysis is aimed at evaluating vaccine efficacy, i.e. the biological effect of the vaccine on SARS-CoV-2 infection. This parameter can be challenging to evaluate in any efficacy trial, given that with sufficient follow-up and heterogeneity in risk, there is culling of high-risk participants over time and the effect estimated using all follow-up is biased relative to the per-exposure reduction in risk of infection- although importantly the bias is small in the setting of low incidence with short follow-up and realistic heterogeneity assumptions (52). In secondary analyses, vaccine *effectiveness* will be evaluated- that is, the effect of the vaccine on SARS-CoV-2 infection, including both biological and behavioral effects caused by knowledge of vaccine receipt or lack of receipt. Vaccine effectiveness has public health importance, given that in public health practice individuals know whether and when they have received vaccine, and may modify their behavior given this knowledge. To evaluate vaccine effectiveness, the same primary analysis methods will be applied but without adjustment for covariates that are associated with SARS-CoV-2 exposure. Vaccine effectiveness will be evaluated against all the clinical endpoints, e.g. viral load, secondary transmission, COVID-19 disease, and seroconversion.

The primary analysis of VE against SARS-CoV-2 infection will be repeated among participants in the FAS, counting all SARS-CoV-2 infection events after Day 0; in the D29-set but including baseline seropositive participants [Secondary Objective 5]; and finally in the PP set [Secondary Objective 9]. The same methods also apply to evaluating VE against seroconversion [Secondary Objective 2] and VE against COVID-19 disease [Secondary Objective 3]. Additional secondary VE analyses will be conducted where the time of SARS-CoV-2 infection diagnosis is defined as the earliest of the first positive PCR result and the date of university positive test result.

The asymptomatic SARS-CoV-2 infection endpoint [Secondary Endpoint 8] is defined by the PCR tests of daily swabs and serologic diagnostic testing schedule at month 0, 2, and 4 The occurrence of a COVID-19 disease event is a competing risk because the occurrence of COVID-19 disease precludes the possibility of occurrence of a subsequent asymptomatic SARS-CoV-2 infection event. Therefore, VE against asymptomatic SARS-CoV-2 infection will be assessed using methods accounting for COVID-19 disease as a competing risk. The SAP will provide details.

Vaccine efficacy against the infection and disease endpoints as measured by the reduction in the cumulative incidence of the endpoint by Month 4, will also be evaluated using the Nelson-Aalen estimator of cumulative incidence. This analysis does not rely on the proportional hazards assumption and has a simple interpretation that lends itself to understanding of population effects.

6.4.4 VL analyses

Primary analyses of the vaccine effect on VL will focus on the primary VL endpoint, peak log10 viral load, and will condition on primary endpoint event status. Peak log10 viral load will be estimated by the observed maximum log10 viral load measure; secondary VL analyses will employ parametric models to estimate VL at peak. The mean observed log10

viral load will be compared between the immediate and delayed vaccination arms using methods that adjust for baseline covariates and covariates at the time of SARS-CoV-2 infection diagnosis, per the study SAP. The covariate adjustment is used to control for potential post-randomization selection bias, whereby SARS-CoV-2 infected participants in the two treatment arms are not comparable in ways that are associated with the VL endpoint. Importantly, this bias occurs when there are factors that modify VE against SARS-CoV-2 infection *and* that are associated with the VL endpoint. Full details around the analysis plan will be specified in the study SAP.

Secondary analyses of vaccine effects on VL will consider a set of other summary measures of the VL curve, each of which have merits for further characterizing the vaccine effect. These include a model-based estimate of peak log10 viral load, area under the log viral load curve [AUC] calculated using the trapezoidal rule, and area under the portion of the log viral load curve over 10^5 viral copies/mL. Some mathematical modeling studies provide evidence of transmission potential for VL above this threshold (21), but there is considerable uncertainty and therefore other thresholds will be considered. Analysis methods will be the same as for the primary VL endpoint above.

In addition, the above VL endpoints will be evaluated using unconditional analyses, ie, not conditioning on SARS-CoV-2 infection status, among all enrolled participants. Participants not diagnosed with SARS-CoV-2 infection will have an endpoint value defined for analysis, typically a value of zero. The 'transmission potential' endpoint [Secondary endpoint 4] is one such endpoint that is defined unconditionally. The merit of unconditional analyses is that they are not subject to potential post-randomization selection bias. A disadvantage of the unconditional analyses is that they may have lower statistical power.

An additional VL endpoint that will be studied is a censored event-time variable, time to VL above 10^5 copies/mL. This endpoint is defined for all enrolled participants and will be analyzed using the same methods used to evaluate VE against SARS-CoV-2 infection, but only counting infections once VL is above 10^5 copies/mL. Additional methods of analysis that allow for the uncertainty in the level of virus that is associated with transmission will be considered.

The study SAP will detail the secondary analysis approaches for the VL endpoints.

6.4.5 Secondary transmission analyses

6.4.5.1 Identifying 'adjudicated transmission events'

An expert adjudication committee will examine and make a primary determination to identify 'adjudicated transmission events' from a main study participant diagnosed with infection via the university testing system ('index case') to a contact in the PCC or CACC diagnosed with SARS-CoV-2 infection. Note that the close contact could also be a main study participant in theory, although this is unlikely given the relatively small proportion of each university population that will be recruited to the main study. The adjudication committee will operate under a charter that lays out a pre-specified set of criteria for defining the occurrence and direction of transmission and will be blinded to the treatment assignment of all study

participants and contacts. The endpoint definition will include criteria that need to be met regarding the temporal relationship (eg, evidence that person B's infection occurred after person A's infection, evidence of compatible incubation period), epidemiologic link (evidence of close contact during person A's infectious period), and relatedness of their SARS-CoV-2 viruses.

The adjudication committee will be provided with all relevant data from the study to deduce transmission events from potential transmission pairs. A potential transmission pair is a post-baseline SARS-CoV-2- infected main study index case and their identified contact(s) in the PCC or CACC who have positive SARS-CoV-2 test results within a prespecified period (eg, two weeks) of the index case diagnosis. This will include the type, frequency, and magnitude (eg, duration and setting) of contact between the index case and contact based on risk assessment questionnaires given to the index case and the contact; the timing of diagnosis of infection by PCR for the index case and the contact (defined as the date of specimen collection); the VL trajectories, serology, and symptomatology for the index case and the contact; and summaries of the viral sequences for the index case and the contact including the number of nucleotide differences in the sequences and any clustering and results of phylogenetic analyses (see Section 6.4.5.3). Adjudication of all potential transmission events will occur within a short time frame at the end of the study to ensure decisions are being made using the same background research on transmission and to ensure that the adjudication can take into account all SARS-CoV-2 infection events captured in the main study cohort and both contact cohorts. Primary analyses of the vaccine effect on secondary transmission will rely on the determination of this committee and use the adjudicated transmission events and outcomes in statistical models. These same primary analyses will also only include main study participants diagnosed with infection through the university testing system ('index cases'), since such a diagnosis is the trigger for contact referral. However, secondary analyses may incorporate the full set of main study participants diagnosed with infection either through university testing or through PCR testing of swabs collected through the study, where transmission events from the latter set of cases will be treated as potentially informative missing data, per methods described in the SAP.

6.4.5.2 Analyses of the vaccine effect on secondary transmission

Primary analyses of vaccine efficacy against secondary transmission will include adjudicated transmission events collected from both the PCC and CACC. Both conditional and unconditional analyses will be performed. Conditional analyses will compare the expected number of adjudicated transmission events between index cases in the immediate vs. delayed vaccination arms using Poisson regression, where goodness of fit tests will be used to determine whether an overdispersed Poisson or other alternative distributions fit the data better, per the study SAP. The SAP will specify the baseline participant characteristics and measurements available at the time of primary endpoint diagnosis that are potentially associated with secondary transmission to be included as covariates. The conditional analysis may have greater power than an unconditional analysis, e.g. under low VE against SARS-CoV-2 infection. However, it is subject to the post-randomization selection bias, as discussed in relation to the viral load endpoint. In general, it will be biased if there are factors that modify VE against SARS-CoV-2 infection that are also associated with onward transmission; or if factors associated with onward transmission are modifiers of VE against infection.

A secondary analysis of this endpoint will measure vaccine efficacy against secondary transmission as the percent reduction in the expected number of adjudicated transmission events linked to a participant in the immediate vaccination arm relative to the delayed vaccination. In contrast to the primary transmission analysis that conditions on the main study participant being infected, all main study participants will contribute to the analysis including those who do not become infected to reflect the fact that SARS-CoV-2-uninfected participants do not transmit to others and therefore have 0 transmission events. Let N(t) = number of adjudicated transmission events for a given index case by time t (time since randomization) which is assumed to follow a zero-inflated Poisson distribution with the proportion in the point mass at 0 being time-dependent. The mean of the non-zero point mass component is $\mu e^{(V\gamma)}$, where V indicates randomization assignment (1 immediate vaccination; 0 delayed vaccination). Further denote infection time as T. We can express the expected number of adjudicated transmission events by follow-up time t as

$$E(N(t)|V,T) = 0 * I(t \leq T) + \mu e^{(V\gamma)} I(t > T|V)$$
$$E[N(t)|V] = E[E(N(t)|V,T)] = \mu e^{(V\gamma)} S(t|V)$$

where $S(t|V)$ is the survival distribution for infection times. The ratio of expected number of adjudicated transmission events between the two arms can then be expressed as

$$\frac{E[N(t)|V=1]}{E[N(t)|V=0]} = \frac{\mu e^{(1\gamma)} S(t|V=1)}{\mu e^{(0\gamma)} S(t|V=0)} = \frac{e^{\gamma} S(t|V=1)}{S(t|V=0)}.$$

Under the proportional hazards assumption, $\frac{S(t|V=1)}{S(t|V=0)}$ is constant, further simplifying this estimator to

$$e^{\theta} = e^{\gamma} e^{\beta},$$

where β is the log hazard ratio from a Cox proportional hazards model for infection time, θ is a measure of the vaccine's impact on total onward transmission (transmission from participants in main study) and e^{θ} is the relative reduction in the proportion of adjudicated transmission events in the immediate vaccination arm relative to the delayed vaccination arm. This is analogous to the product estimator in the proportional means model (53, 54). Note that this formulation assumes the time between detection in the index case participant and infection of a contact is ignorable. Covariate-adjusted approaches will be detailed in the SAP.

The unconditional analysis retains high power even if there are few breakthrough infections in the immediate vaccination group due to high VE against infection. It also properly reflects that a vaccine is successful at reducing secondary transmission if it either meaningfully reduces acquisition or reduces onward transmission from infected participants. Moreover, by avoiding conditioning on SARS-CoV-2 infection, it avoids the post-randomization selection bias issue.

While analyses that pool adjudicated transmission events from the PCC and CACC will produce valid tests of the null hypothesis of no vaccine effect on secondary transmission,

likelihood-based analyses that take into account the different duration of follow-up of contacts in the PCC vs. the CACC, and the potential left-censoring of contact follow-up in the CACC will be explored and may provide greater accuracy and power (38). Details of these analyses, including methods for checking the underlying assumptions and investigations of their operating characteristics using simulations of social and transmission networks, will be provided in the study SAP.

6.4.5.3 Processing and phylogenetic analysis of SARS-CoV-2 viral sequence data

A central sequencing facility will obtain whole-genome sequences from all infected participants in the main study cohort and all infected participants in the PCC and CACC, based on the nasal swab with the highest viral load (see Section 10.3.2). Details about the sequencing platform and methodology for QC processes will be included in the study SAP. This will include the creation of a global alignment, which will be used for the isolation of genes and the sequence analyses of local regions.

A first step in the analysis of the sequence data will be to determine, for each pair of sequences among infected participants or contacts at a given university, which are identical. Data from GenBank, Global Initiative on Sharing Avian Influenza Data (GSAID), and other public databases suggest that we can expect limited variability in genetic sequences, especially those from a single common ancestor and constrained within a single university community over a 4-month time period. Consensus-based whole genomes that differ by more than two nucleotides can reasonably be ruled out as being transmission events.

A second step will be to conduct distance-based analyses of the sequences, including phylogenetic analysis, to identify potential lineages of infecting viral strains, infection clusters, and potentially linked transmissions. Phylogenetic analyses will also include ‘background’ SARS-CoV-2 sequences with geographical or temporal relevance from publicly available databases, such as GenBank and GISAID. The background sequences will help root the tree and give the tree structure and may lend insight into the identification of clusters and into potential transmission events during the adjudication process.

Details about the phylogenetic and other sequence analyses, including the tree-building methods and evolutionary models to be used, as well as approaches to incorporating data from multiple reads, will be available in the study SAP.

6.4.6 Immunogenicity analyses

6.4.6.1 Vaccine immunogenicity

Immunogenicity will be characterized for the immediate vaccination group only, using data from the Case-Cohort Immunogenicity Set (ccIS). Data from quantitative immunogenicity assays will be summarized using positive response rates and geometric means with 95% CIs, for each timepoint for which an assessment is performed. Data from qualitative (ie, yielding a positive or negative result) assays will be summarized by tabulating the frequency of positive responses for each assay at each timepoint that an assessment is performed. Analyses focus

on the Month 2 time point, the Day 1 time point, and the change in marker response between the two time points. The analyses will evaluate immunogenicity in all immediate vaccination arm participants and separately in subgroups defined by baseline SARS-CoV-2 serostatus. The SAP will describe the complete set of immunogenicity analyses.

6.4.6.2 Immune response marker correlates of risk

A separate Trial Marker SAP will describe the statistical methods and data analysis implementations for assessing immune response markers as correlates of the SARS-CoV-2 infection and peak log viral load study endpoints, as well as various types of correlates/surrogates of protection.

An unblinded statistical analysis by treatment assignment of a primary immunogenicity endpoint may be performed when all participants have completed the Month 2 visit and data are available for analysis from at least 80% of these participants. Similarly, an unblinded statistical analysis by treatment assignment of a secondary or exploratory immunogenicity endpoint may be performed when all participants have completed the corresponding immunogenicity visit and data are available for analysis from at least 80% of these participants. The CoVPN Laboratory Center will review the analysis report prior to distribution to the protocol chairs, NIAID, study product developer, and other key CoVPN members and investigators. Reports for distribution or presentation should use PubIDs and not PTIDs for individual responses. Distribution of reports will be limited to those with a need to know for the purpose of informing future trial-related decisions. The CoVPN leadership must approve any other requests for CoVPN immunogenicity analyses prior to the end of the scheduled follow-up visits.

7 Selection and withdrawal of participants

Participants will be generally healthy adults who comprehend the purpose of the study and have provided written informed consent. Volunteers will be recruited and screened; those determined to be eligible, based on the inclusion and exclusion criteria, will be enrolled in the study. Final eligibility determination will depend on information available at the time of enrollment, medical history questionnaire, physical examinations, and answers to self-administered and/or interview questions.

Investigators should always use good clinical judgment in considering a volunteer's overall fitness for trial participation. Some volunteers may not be appropriate for enrollment even if they meet all inclusion/exclusion criteria. Medical, psychiatric, occupational, or other conditions may make evaluation of safety and/or immunogenicity difficult, and some volunteers may be poor candidates for retention.

Determination of eligibility, taking into account all inclusion and exclusion criteria, must be made within 56 days prior to enrollment unless otherwise noted below.

7.1 Inclusion criteria for Main Cohort

General and Demographic Criteria

1. **Age** of 18 through 26 years.
2. **Enrollment in a participating university** and willingness to be followed for the planned duration of the study.
3. **Agrees to allow study staff to access university SARS-CoV-2 testing** data and outcomes.
4. Ability and willingness to provide **informed consent.**
5. **Assessment of understanding**: volunteer demonstrates understanding of this study; completes a questionnaire prior to first vaccination with demonstration of understanding of all questionnaire items answered incorrectly.
6. **Willing to defer SARS-CoV-2 licensed or EUA vaccine** until visit 6 (Month 4)
7. **Agrees not to enroll in another study** of an investigational research agent until the end of the study.
8. **Access to device and internet** for Telehealth visits.

7.2 Exclusion criteria for Main Cohort

General

1. **Acutely ill or febrile 72 hours prior to or at screening**. Fever is defined as a body temperature ≥ 38.0°C/100.4°F. Volunteers meeting this criterion may be rescheduled within the relevant window periods. Afebrile participants with minor illnesses can be enrolled at the discretion of the investigator.

2. **Blood products,** systemic immunoglobulins, or monoclonal antibodies (including against SARS-CoV-2) received within 90 days before first vaccination.

3. **Investigational research agents** received within 30 days before first vaccination.

4. **Self-reported known history of SARS-CoV-2 infection.**

Vaccines and other Injections

5. **Prior administration of a coronavirus** (SARS-CoV-2, SARS-CoV, MERS-CoV) vaccine or current/planned simultaneous participation in another interventional study to prevent or treat COVID-19.

Immune System

6. **Immunosuppressive medications** received within 168 days before first vaccination (not exclusionary: [1] corticosteroid nasal spray; [2] inhaled corticosteroids; [3] topical corticosteroids for mild, uncomplicated dermatologic condition; or [4] a single course of oral/parenteral prednisone or equivalent at doses ≤ 60 mg/day and length of therapy < 11 days with completion at least 30 days prior to enrollment).

Clinically significant medical conditions

7. **Clinically significant medical condition**, physical examination findings, or past medical history with clinically significant implications for current health. A clinically significant condition or process includes but is not limited to:

    - A process that would affect the immune response (well-controlled human immunodeficiency virus infection is allowed), or

    - Any condition specifically listed among the exclusion criteria below.

8. **Any medical, psychiatric, occupational, or other condition** that, in the judgment of the investigator, would interfere with, or serve as a contraindication to, protocol adherence, assessment of safety or reactogenicity, or a volunteer's ability to give informed consent.

9. **Bleeding disorder** (eg, factor deficiency, coagulopathy, or platelet disorder requiring special precautions).

10. **Asplenia**: any condition resulting in the absence of a functional spleen.

11. **History of angioedema or anaphylaxis**, including to vaccines or vaccine components (not exclusionary: angioedema or anaphylaxis with known trigger and no episodes within five years.).

12. **History of generalized urticaria** within past five years.

7.3 Inclusion criteria for Prospective Close Contact (PCC) cohort

1. **Age** of 18 years or older, at the time of signing the informed consent.

2. Willing and able to provide **informed consent**.

3. Expected to be in **frequent close physical proximity with Main Cohort participant** during the study.

4. **Participating in their university SARS-CoV-2 testing system** and willing to share results.

5. **Access to device and internet** for Telehealth visits.

7.4 Inclusion criteria for Case-ascertained Close Contact (CACC) cohort

1. **Age** of 18 years or older, at the time of signing the informed consent.

2. Willing and able to provide **informed consent**.

3. **Access to device and internet** for Telehealth visits.

4. Willing to share **results of SARS-CoV-2 testing**.

5. Had **close contact with Main Cohort participant with known PCR-confirmed SARS-CoV-2 infection** (eg, index case). Close contacts will have had exposure to the index participant, generally within 72 hours of an index diagnosis, and may include individuals that meet any of the following guidelines:

    - Prolonged close physical proximity with Main Cohort participant within a residence/vehicle/enclosed space without maintaining social distance,

    - Medical staff, first responders, or other care persons who cared for the index case without appropriate personal protective equipment.

    Further information on the definition of close contact can be found in the CoVPN 3006 Study Specific Procedures (SSP).

8 Study product

The protocol schema is shown in Table 1-1.

8.1 Vaccine regimen

Group 1

Treatment 1 (T1): Moderna COVID-19 Vaccine in 100 mcg dose given as 0.5 ml IM into the deltoid muscle on Day 1 and Day 29.

Group 2

Treatment 2 (T2): Moderna COVID-19 Vaccine in 100 mcg dose given as 0.5 ml IM into the deltoid muscle on Day 113 and Day 141.

8.2 Storage, handling, preparation, and administration

Vaccination providers will refer to EUA vaccine instructions accessed and updated at https://www.modernatx.com/covid19vaccine-eua/.

8.3 Acquisition of Moderna COVID-19 Vaccine

Moderna COVID-19 Vaccine will be provided by Moderna, Inc. through the US Government COVID-19 Vaccine Response.

9 Clinical procedures

Procedures are in place so that study visits may be conducted remotely, such as via phone, text message, email, or other electronic means, in lieu of, or in combination with, in-person visits at the clinical research site. Furthermore, some visit procedures may be conducted outside the CRS (see CoVPN 3006 SSP for additional details).

The schedules of clinical procedures are shown in Appendix D, Appendix E, Appendix F and Appendix G.

9.1 Informed consent

Informed consent is the process of working with participants so that they fully understand what will and may happen to them while participating in a research study. The CoVPN informed consent form documents that a participant (1) has been informed about the potential risks, benefits, and alternatives to participation, and (2) is willing to participate in a CoVPN study. Informed consent encompasses all written, verbal, and electronic study information CRS staff provide to the participant, before and during the trial. CRS staff will obtain informed consent of participants according to the CRS's SOP on the informed consent process.

If any new information is learned that might affect the participants' decisions to stay in the trial, this information will be shared with trial participants. If necessary, participants will be asked to provide revised informed consent forms.

9.1.1 Screening consent form

Without a general screening consent, screening for a specific study cannot take place until the site receives protocol registration from NIAID or its designee.

Some CRSs have approval from their IRB/EC and any applicable RE to use a general screening consent form that allows screening for an unspecified SARS-CoV-2 vaccine trial. In this way, CRS staff can continually screen potential participants and, when needed, proceed quickly to obtain protocol-specific enrollment consent. Sites conducting general screening or prescreening approved by their IRB/EC and any applicable RE may use the results from this screening to determine eligibility for this protocol, provided the tests are conducted within the time periods specified in the eligibility criteria.

9.1.2 Protocol-specific consent forms

The protocol-specific consent forms describe the study products to be used and all aspects of protocol participation, including screening and enrollment procedures. A sample protocol-specific consent form for the Main cohort is located in Appendix A. A sample protocol-specific consent form for the PCC cohort is located in Appendix B. A sample protocol-specific consent form for the CACC cohort is located in Appendix C.

Each CRS is responsible for developing a protocol-specific consent form(s) for local use, based on the sample protocol-specific consent forms. The consent form(s) must be developed in accordance with requirements of the following:

- CRS's IRB/EC and any applicable REs
- CRS's institution
- Elements of informed consent as described in Title 45, CFR Part 46 and Title 21 CFR, Part 50, and in ICH E6(R2), Good Clinical Practice: Consolidated Guidance 4.8

Study sites are strongly encouraged to have local community representatives from the target population review their sites-specific consent forms. This review should include, but should not be limited to, issues of cultural competence, local language considerations, and the level of understandability.

The sample informed consent form(s) include(s) instructions for developing specific content.

9.1.3 Assessment of Understanding

Study staff are responsible for ensuring that participants fully understand the study before enrolling them. This process involves reviewing the informed consent form with the participant, allowing time for the participant to reflect on the procedures and issues presented, and answering all questions completely.

An Assessment of Understanding is used to document the participant's understanding of key concepts in this study. Participants must demonstrate understanding of all questions answered incorrectly. This process and the participant's understanding of the key concepts is to be recorded in the eConsent.

9.2 Pre-enrollment procedures for Main Cohort, Immediate and Delayed Vaccination Groups

Screening may occur over the course of several contacts/visits, up to and including before randomization on Day 1. All inclusion and exclusion criteria must be assessed within 56 days before enrollment, unless otherwise specified in the eligibility criteria (or below in this section). Screening results will be reviewed with the volunteer.

After the appropriate informed consent has been obtained and before enrollment, the following procedures are performed:

- Participant will download the electronic diary (eDiary) application to their smartphone or tablet and complete an optional application training. Participant will use eDiary to enter data. Data collection will include:
    - Medical history questionnaire

  - Volunteer demographics in compliance with the NIH Policy on Reporting Race and Ethnicity Data: Subjects in Clinical Research, Aug. 8, 2001 (available at http://grants.nih.gov/grants/guide/notice-files/NOT-OD-01-053.html)
  - Baseline SARS-CoV-2 infection risk information

- Discussion of the eDiary entries done by the participant with the site staff
- Review of materials for possible self-collection of blood by the participant

9.3 Enrollment and vaccination visits for Main Cohort, Immediate Vaccination Group

Once a volunteer has consented to trial participation and is found to meet all eligibility criteria (see Sections 7.1 and 7.2), the CRS requests the randomization assignment via a Web-based randomization system. **Enrollment is simultaneous with randomization**. In general, the time interval between randomization and first vaccination for the Immediate Vaccination Group should not exceed 4 working days. However, circumstances may require a participant's visit to be changed. This may exceed the 4-day randomization time limit.

At vaccination visits, the following procedures are performed **before vaccination**:

- COVID-19 symptom check (including temperature), both vaccination visits
- Specimen collection (**only at first vaccination** visit, see Appendix D)

Following completion of all procedures in the preceding list, and if results indicate that vaccination may proceed, vaccination is prepared and administered per Moderna EUA Fact Sheet and Full Prescribing Information for Vaccination Providers that can be accessed at https://www.modernatx.com/covid19vaccine-eua/).

Additionally, the participants will be:

- Reminded to enter information in eDiary throughout the study
- Provided with a supply of nasal swabs and shown how to swab both nostrils

9.4 Follow-up procedures for Main Cohort, Immediate Vaccination Group

The following procedures are performed **during follow-up per schedule in** Appendix D:

- Participants reminded to enter information in eDiary throughout the study (see Section 9.15)
- Nasal swab collection by participant

- Blood collection by CRS staff, phlebotomist, or participant

9.5 Enrollment (visit 2) and visit 4 (Month 2) for Main Cohort, Delayed Vaccination Group

Once a volunteer has consented to trial participation and is found to meet all eligibility criteria (see Sections 7.1 and 7.2), the CRS requests the randomization assignment via a Web-based randomization system. **Enrollment is simultaneous with randomization**.

Before leaving the clinic, the participants will be:

- Reminded to enter information in eDiary throughout the study
- Provided with a supply of nasal swabs and shown how to swab both nostrils

At visits 2 and 4, the following procedures are performed **per schedule in** Appendix E:

- eDiary entries by participant (see Section 9.15)
- Nasal swab collection by participant
- Blood collection by CRS staff, phlebotomist, or participant

9.6 Vaccination visits for Main Cohort, Delayed Vaccination Group

At vaccination visits, the following procedures are performed **before vaccination**:

- COVID-19 symptom check (including temperature)
- Specimen collection (**only at first vaccination** visit, see Appendix E)

Following completion of all procedures in the preceding list, and if results indicate that vaccination may proceed, vaccination is prepared and administered, per Moderna EUA Fact Sheet and Full Prescribing Information for Vaccination Providers that can be accessed at https://www.modernatx.com/covid19vaccine-eua/).

9.7 Procedures for Main Cohort participant diagnosed with a SARS-CoV-2 infection during the study

If a participant is diagnosed with a SARS-CoV-2 infection during the course of the study, CDC guidelines on administration of vaccination will be followed.

Main Cohort participants who are clinically diagnosed with SARS-CoV-2 infection are referred to as clinically diagnosed index cases. They will be instructed to quarantine per local guidelines and the following procedures will be performed:

- eDiary collection of daily symptoms of COVID-19 and assessment of whether clinical care was sought or hospitalization occurred (via self-report and/or request for medical records) through an eDiary for a period of 14 days or until symptoms have resolved, whichever is longer
- Daily nasal swabbing continued per schedule of procedures (see Appendix D)
- Blood collection by phlebotomist or participant for SARS-CoV-2 clinical serology
- Close contacts identification (see Section 9.9)

Following resolution of the quarantine period, participants will continue scheduled study visits. Follow-up duration for participants diagnosed with moderate or severe COVID-19 disease may be adjusted in consultation with the CRS (eg, to avoid interference with participant initiation of SARS-CoV-2 treatment).

9.8 Enrollment and follow-up procedures for Prospective Close Contact (PCC) cohort

Upon enrollment, participants from the Main Cohort will be asked to invite individuals with whom they will be in frequent close physical proximity during the study to join the PCC cohort (See Section 7.3 and CoVPN 3006 SSP for additional information on PCC cohort). New PCC contacts may also be added during the study.

Prospective close contacts will be given referral codes and information about how to enroll in the study as part of the PCC cohort. If they choose to enroll and sign an informed consent, they will be asked to:

- provide access to their routine SARS-CoV-2 testing reports
- enter data weekly through eDiary (see Section 9.15)

If a PCC cohort participant or the Main Cohort participant to whom he/she is connected is diagnosed with a SARS-CoV-2 infection during the course of the study, the PCC cohort participant will be instructed to perform the following procedures (see Appendix F):

- eDiary collection of daily symptoms of COVID-19 and assessment of whether clinical care was sought or hospitalization occurred (via self-report and/or request for medical records) through an eDiary for a period of 14 days or until symptoms have resolved, whichever is longer
- provide nasal swab for 14 days

- self-collect blood samples on day 1 and day 29, relative to the notification of infection

For Main Cohort participants who are also PCC cohort participants related to an index case in another Main cohort participant, the following procedures in addition to those listed in Appendix D may be performed:

- one additional blood collection (self-collection) as soon as possible following the report of the index case
- eDiary collection of information regarding exposure to the Index case
- eDiary collection of daily symptoms of COVID-19 and assessment of whether clinical care was sought or hospitalization occurred (via self-report and/or request for medical records) through an eDiary for a period of 14 days or until symptoms have resolved, whichever is longer

See CoVPN 3006 SSP for additional information on PCC cohort.

9.9 Enrollment and Follow-up procedures for Case-Ascertained Close Contact (CACC) cohort

Participants from the Main Cohort who are clinically diagnosed with SARS-CoV-2 (referred to as the Index case) during the study will be asked to contact any individuals with whom they have been in close contact within about 72 hours of the date of the SARS-CoV-2 diagnosis test. See Section 7.4 and CoVPN 3006 SSP for additional information on case-ascertained close contacts.

Potential CACC cohort participants will be given referral codes and information about how to enroll in the study as part of the CACC cohort, if they choose to, and sign an informed consent.

Once a volunteer has consented to be a part of CACC cohort, the following procedures will be performed (see Appendix G):

- eDiary collection of information regarding exposure to the Index case
- eDiary collection of daily symptoms of COVID-19 and assessment of whether clinical care was sought or hospitalization occurred (via self-report and/or request for medical records) through an eDiary for a period of 14 days or until symptoms have resolved, whichever is longer.
- Outcomes of SARS-CoV-2 testing performed by the university or other facilities
- Nasal swab self-collection from day 1-14
- Blood collection by participant for SARS-CoV-2 clinical serology at day 3 and at day 29

If a CACC participant is diagnosed with a SARS-CoV-2 infection during the course of the study, they will be instructed to track daily symptoms of COVID-19 through an eDiary for a period of 14 days or until symptoms have resolved, whichever is longer.

For Main Cohort participants who are also CACC cohort participants, the following procedures in addition to those listed in Appendix D may be performed:

- one additional blood collection (self-collection) as soon as possible
- eDiary collection of information regarding exposure to the Index case
- eDiary collection of daily symptoms of COVID-19 and assessment of whether clinical care was sought or hospitalization (via self-report and/or request for medical records) occurred through an eDiary for a period of 14 days or until symptoms have resolved, whichever is longer.

9.10 Visit windows and missed visits

Visit windows are included in Appendix H. The procedures for documenting missed visits and out of window visits are described in CoVPN 3006 SSP.

9.11 Early termination visit

In the event of early participant termination, site staff should consider if final specimen collection is appropriate.

9.12 Pregnancy for Main Cohort

For Main cohort participants who have been vaccinated, pregnancies and pregnancy outcomes will be reported via the FDA/CDC Vaccine Adverse Event Reporting System (VAERS) (see Section 11.1).

9.13 SARS-CoV-2 monitoring by universities for Main Cohort

Participating universities have SARS-CoV-2 monitoring plans in place. The monitoring will typically provide approximately twice weekly SARS-CoV-2 testing for enrolled Main Cohort participants. For any positive test result, the institution will inform the student (initiating quarantine), will provide the SARS-CoV-2 test results to the students, and will carry out contact tracing. Participants will be instructed to report positive test results via the eDiary.

9.14 COVID-19 symptom surveillance for Main Cohort

Participants will be monitored for symptoms of COVID-19 throughout the course of the study via a weekly eDiary. These symptoms will be used to establish whether a participant meets criteria for COVID-19 disease as defined by Secondary Endpoint 4. Due to the overlap of symptoms that can result from vaccination and symptoms of COVID-19, monitoring will begin 7 days following the first study product administration. Symptom monitoring will be paused 7 days following the second product administration. If a participant has symptoms consistent with COVID-19, they will be encouraged to seek testing and clinical care through their local testing and care facilities. Results of testing will be reported through the eDiary and participants who have positive test results will follow procedures described in Section 9.7.

9.15 Use of electronic Diary (eDiary) for Clinical Outcomes Assessments

At the time of consent, participants must confirm they will be willing to complete an eDiary collection of study data (see below for details of data to be collected).

The eDiary will be completed using an application downloaded to their smartphone or tablet. This study will utilize the Medidata Patient Cloud Application as the eDiary software. This application allows for real-time data collection directly from participants.

After participants have signed the informed consent, all participants will be instructed to download the eDiary application on their personal smartphone or tablet. The eDiary application has a self-training component, enabling immediate use. The site staff will perform any retraining as necessary.

For Main Cohort participants,

Study participants will record data in the eDiary throughout the study as described in Appendix D and Appendix E, following the prompts provided for data collection. eDiary data collection areas may include:

- Medical history questionnaire
- Volunteer demographics
- Risk assessment for SARS-CoV-2 infection (see Appendix D and Appendix E)
- Surveillance for symptoms of COVID-19 (see Section 9.14)
- Self-collection of samples (eg, nasal swabs) (see Appendix D and Appendix E)
- Outcomes of SARS-CoV-2 testing performed by the University or other facilities (see Appendix D and Appendix E)

- COVID-19 symptom tracking upon incident diagnosis of SARS-CoV-2 infection, including assessments of whether clinical care was sought or hospitalization occurred via self-report and/or request for medical records (see Section 9.7)

Additionally, participants will be reminded through the eDiary of the need to continue practices for reducing acquisition and transmission of SARS-Cov-2 regardless of their vaccination status.

For Prospective Close Contact (PCC) cohort participants,

PCC participants record data in the eDiary as described in Appendix F, following the prompts provided for data collection. eDiary data collection areas may include:

- Collection of demographic information
- Collection of information regarding exposure to the main study participant
- Outcomes of SARS-CoV-2 testing performed by the university or other facilities
- Self-collection of samples (eg, nasal swabs) (see Appendix F)
- COVID-19 symptom tracking upon incident diagnosis of SARS-CoV-2 infection in PCC or main study participant, including assessments of whether clinical care was sought or hospitalization occurred via self-report and/or request for medical records
- Recording of any COVID-19 vaccines received outside of the study

For Case-Ascertained Close Contact (CACC) cohort participants,

CACC participants will record data in the eDiary as described in Appendix G, following the prompts provided for data collection. eDiary data collection areas may include:

- Collection of demographic information
- Collection of information regarding exposure to the Index case
- Outcomes of SARS-CoV-2 testing performed by the university or other facilities
- COVID-19 symptom tracking, including assessments of whether clinical care was sought or hospitalization occurred via self-report and/or request for medical records
- Self-collection of samples (eg, nasal swabs) (see Appendix G)
- Recording of any COVID-19 vaccines received outside of the study

10 Laboratory

10.1 CRS laboratory procedures

The CoVPN 3006 SSP and other study materials provide further guidelines for operational issues concerning the clinics and laboratories. These documents include special considerations for phlebotomy and guidelines for general specimen collection, specimen labeling and specimen processing.

Tube types for blood collection are specified in CoVPN 3006 SSP.

Of note, all assays described below are performed as research assays to evaluate the ability of the SARS-CoV-2 virus to induce immune responses in the context of the participants' genetic background and are not approved for use in medical care. Results from these assays are not made available to participants or medical professionals to guide treatment decisions.

10.2 Total blood volume

Required blood collections per visit are shown in Appendix D, Appendix E, Appendix F and Appendix G. The total blood volume drawn for each participant will not exceed 500 mL in any 56-day (8-week) period.

10.3 Endpoint assays

10.3.1 SARS-CoV-2 PCR

Real-time reverse transcription polymerase chain reaction (RT-PCR) assays to detect SARS-CoV-2 will be run on nasal samples collected from study participants. After RNA extraction, specimens will be analyzed by a laboratory developed test using the World Health Organization's E/RdRp primer set, the N1/N2 primer set from the Centers for Disease Control and Prevention, or tests from Hologic (Panther Fusion), DiaSorin (Simplexa), or Roche (cobas). Novel methods to detect SARS-CoV-2 may also be used if applicable.

10.3.2 SARS-CoV-2 viral sequencing

Genome sequencing of SARS-CoV-2 virus may be performed on nasal samples from study participants using a metagenomic approach. RNA from positive specimens is converted to a cDNA library by reverse transcription and the library is sequenced. Resulting consensus sequences are assembled against a SARS-CoV-2 reference genome.

10.3.3 Neutralizing antibody assay (nAb)

SARS-CoV-2–specific nAb assays may be performed on serum samples from study participants. The assay will test neutralization of S-pseudotyped and/or full-genome

recombinant viruses as measured by a reduction in luciferase (Luc) reported gene expression after infection in ACE-2 positive cells.

10.3.4 Binding antibody multiplex assay (BAMA)

SARS-CoV-2 specific total IgG and IgM binding antibodies may be assessed in serum samples. In addition, SARS-CoV-2–specific serum IgA and IgG subclass (IgG1, IgG2, IgG3, and IgG4) antibodies may also be assessed. Epitope specificity by ACE-2 blocking assay and responses to endemic coronavirus proteins may also be assessed.

10.3.5 Antibody avidity (BioLayer Interferometry and BAMA avidity index)

SARS-CoV-2-specific polyclonal serum antibody and IgG subclass avidity may be measured using BAMA with the addition of a dissociation step to calculate the antibody avidity index (BAMA-AI). BLI and/or SPR technologies may also be used to measure antibody avidity.

10.3.6 Fc-Mediated Antibody Function

Additional assays to measure SARS-CoV-2-specific antibody Fc functions may be performed (ie, antibody dependent cellular cytotoxicity [ADCC], antibody-dependent cellular phagocytosis [ADCP], antibody-dependent neutrophil phagocytosis [ADNP], binding antibody FcR array, and S-protein-Expressing Cell Antibody Binding Assay [SECABA]).

10.4 Lab assay portfolio

Additional assays may be performed per the Laboratory Center assay portfolio, which includes immune assessments such as those for cellular, humoral, and innate immune responses, and host genetics. The assay portfolio will be updated periodically to include new assays and adjust qualification levels of existing assays.

10.5 Exploratory studies

Samples may be used for other testing and research related to furthering the understanding of SARS-CoV-2 immunology, virology, or vaccines. In addition, cryopreserved samples may be used to perform additional assays to support standardization and validation of existing or newly developed methods.

10.6 Specimen storage and other use of specimens

The CoVPN stores specimens from all study participants indefinitely, unless a participant requests that specimens be destroyed or if required by IRB/EC, or RE.

Other use of specimens is defined as studies not covered by the protocol or the informed consent forms for the study (see Appendix A, Appendix B, and Appendix C).

This research may relate to SARS-CoV-2, vaccines, coronaviruses, the immune system, and other diseases. This could include genetic testing and, potentially, genome-wide studies. This research is done only to the extent authorized in each study site's informed consent form, or as otherwise authorized under applicable law. Other research on specimens ("other use") will occur only after review and approval by the CoVPN, the IRB/EC of the researcher requesting the specimens, and the IRBs/ECs/REs of the CRSs if required.

As part of consenting for the study, participants document their initial decision to allow or not allow their specimens to be used in other research, and they may change their decision at any time. The participant's initial decision about other use of their specimens, and any later change to that decision, is recorded by their CRS in a Web-based tool that documents their current decisions for other use of their specimens. The CoVPN will only allow other research to be done on specimens from participants who allow such use.

CRSs must notify CoVPN Regulatory Affairs if institutional or local governmental requirements pose a conflict with or impose restrictions on specimen storage or other use of specimens.

10.7 Biohazard containment

The transmission of blood-borne pathogens can occur through contact with contaminated needles, blood, and blood products, and the transmission of SARS-CoV-2 and other respiratory pathogens may occur through contact with contaminated respiratory droplets, aerosols, and other biological materials. Appropriate precautions will be employed by all personnel in the collection, shipping, and handling of all specimens for this study, as currently recommended by local health authorities, the CDC, the NIH, or other applicable agencies.

All dangerous goods materials, including Biological Substances, Category A or Category B, must be transported according to instructions detailed in the International Air Transport Association Dangerous Goods Regulations.

11 Safety Reporting

11.1 FDA/CDC Vaccine Adverse Event Reporting System (VAERS)

The Vaccine Adverse Event Reporting System (VAERS) is co-managed by the U.S. CDC and FDA and serves as the reporting mechanism for adverse events occurring from licensed and EUA vaccines, including the Moderna COVID-19 vaccine being used in this trial. VAERS accepts and analyzes reports of adverse events (AEs) after a person has received a vaccination. It is accessed at https://vaers.hhs.gov/reportevent.html.

Risks associated with receipt of the Moderna COVID-19 Vaccine and AE reporting requirements are described in the "Fact Sheet For Healthcare Providers Administering Vaccine (Vaccination Providers) Emergency Use Authorization (EUA) Of The Moderna COVID-19 Vaccine To Prevent Coronavirus Disease 2019 (COVID-19)", available at https://www.modernatx.com/covid19vaccine-eua/.

All vaccination providers, including those at the CRS, are required to report to VAERS the following adverse events after COVID-19 vaccination, under Emergency Use Authorization (EUA), and other adverse events if later revised by CDC |(https://www.cdc.gov/vaccines/covid-19/vaccination-provider-support.html and https://vaers.hhs.gov/faq.html):

- Vaccine administration errors, whether or not associated with an adverse event (AE)
- Serious AEs regardless of causality. Serious AEs per FDA are defined as:
    - Death;
    - A life-threatening AE;
    - Inpatient hospitalization or prolongation of existing hospitalization;
    - A persistent or significant incapacity or substantial disruption of the ability to conduct normal life functions;
    - A congenital anomaly/birth defect;
    - An important medical event that based on appropriate medical judgement may jeopardize the individual and may require medical or surgical intervention to prevent one of the outcomes listed above.
- Cases of Multisystem Inflammatory Syndrome
- Cases of COVID-19 that result in hospitalization or death

CRS vaccination providers are encouraged to report to VAERS any additional clinically significant AEs following vaccination, even if they are not sure if vaccination caused the event; and are to report any additional select AEs and/or any revised safety reporting

requirements per FDA's conditions of authorized use of vaccine(s) throughout the duration of any COVID-19 Vaccine being authorized under an EUA.

CRS vaccination providers will complete and submit reports to VAERS online at https://vaers.hhs.gov/reportevent.html. Further assistance with reporting to VAERS is accessed by calling 1-800-822-7967. The reports should include the words "Moderna COVID- 19 Vaccine EUA" in the description section of the report.

As indicated in the "Fact Sheet For Healthcare Providers Administering Vaccine (Vaccination Providers) Emergency Use Authorization (EUA) Of The Moderna COVID-19 Vaccine To Prevent Coronavirus Disease 2019 (COVID-19)", vaccination providers (including those at the CRS) are encouraged, to the extent feasible, to report adverse events to Moderna.

11.2 Reporting safety events to v-safe

An additional source of VAERS reports will be through a program administered by the CDC known as v-safe (https://www.cdc.gov/coronavirus/2019-ncov/vaccines/safety/vsafe.html). V-safe is a smartphone-based opt-in program that uses text messaging and web surveys from CDC to check in with vaccine recipients for health problems following COVID-19 vaccination. The system also will provide telephone follow-up to anyone who reports medically significant (important) adverse events. Responses indicating missed work, inability to do normal daily activities, or that the recipient received care from a doctor or other healthcare professional will trigger the VAERS Call Center to reach out to the participant and collect information for a VAERS report, if appropriate. CRS vaccination providers will encourage the study participants receiving the Moderna COVID-19 vaccine to use v-safe.

12 Protocol conduct

This protocol and all actions and activities connected with it will be conducted in compliance with the principles of GCP (ICH E6 (R2)), and according to NIAID and CoVPN policies and procedures as specified in the CoVPN 3006 SSP*,* NIAID Clinical Research Policies and Standard Procedures Documents including procedures for the following:

- Protocol registration, activation, and implementation;
- Informed consent, screening, and enrollment;
- Study participant reimbursement;
- Clinical and safety assessments;
- Safety monitoring and reporting;
- Data collection, documentation, transfer, and storage;
- Participant confidentiality;
- Study follow-up and close-out;
- Unblinding of staff and participants;
- Quality control;
- Protocol monitoring and compliance;
- Specimen collection, processing, and analysis;
- Exploratory and ancillary studies and sub-studies, and
- Destruction of specimens.

Any policies or procedures that vary from NIAID and CoVPN standards or require additional instructions (eg, instructions for randomization specific to this study) will be described in the CoVPN 3006 SSP.

12.1 Study termination

NIAID reserves the right to terminate or curtail a clinical study for any reason, including but not limited to the following (reference: https://grants.nih.gov/grants/guide/rfa-files/RFA-AI-12-012.html):

- risk to subject safety
- the scientific question is no longer relevant or the objectives will not be met (ie, slow accrual)
- failure to comply with GCP, U.S. Federal regulations, or Terms and Conditions of Award
- occurrence of unforeseen drug safety issues or data from preclinical studies indicate a presence of unanticipated toxicity
- risks that cannot be adequately quantified
- ethical concerns raised by the local community or local medical care/health care authorities
- failure to remedy deficiencies identified through site monitoring
- substandard data
- reaching a major study endpoint substantially before schedule with persuasive statistical significance.

This study may also be terminated early by the determination of the FDA, the United States Department of Health and Human Services Office for Human Research Protections (OHRP). In addition, the conduct of this study at an individual CRS may be terminated by the determination of the IRB/EC and any applicable RE.

12.2 Emergency communication with study participants

As in all clinical research, this study may generate a need to reach participants quickly to avoid imminent harm, or to report study findings that may otherwise concern their health or welfare and their willingness to remain on study.

When such communication is needed, the CRS will request that its IRB/EC and any applicable RE expedite review of the message. If this review cannot be completed in a timeframe consistent with the urgency of the required communication, the site can contact the participant without IRB/EC approval if such communication is necessary to avoid imminent harm to the study participant. The CRS must notify the IRB/EC and any applicable RE of the matter as soon as possible.

13 Version history

The Protocol Team may modify the original version of the protocol. Modifications are made to CoVPN protocols via clarification memos, letters of amendment, or full protocol amendments.

The version history of, and modifications to, Protocol CoVPN 3006 are described below.

Protocol history and modifications

Date: Error! Use the Home tab to apply Mod Date to the text that you want to appear here.

Protocol version: ***Error! Use the Home tab to apply Mod Version to the text that you want to appear here.***
Protocol modification: ***Error! Use the Home tab to apply Mod Title to the text that you want to appear here.***

Item 1 Revised in Section 3, *Objective and Endpoints,* Section 6, *Statistical considerations,* Section 9, *Clinical procedures*, Appendix A, *Sample informed consent form for the main cohort*, and Appendices D and E, *Procedures for the main cohort*: Month 3 visit removed for both Main cohort groups, M1 visit removed for delayed vaccination group of Main cohort, and Month 1 blood collection removed for immediate vaccination group of Main cohort

Item 2 Added in Section 1, *Overview*: 2 new endpoint laboratories

Item 3 Updated in Section 1.1, *Protocol team*: team members

Item 4 Deleted Section 6.1, *Accrual restrictions*: no accrual restrictions for this study

Item 5 Revised in Section 6.4, *Statistical analyses*: minor clarifying text revisions

Item 6 Corrected in Section 6.4.4.7, *VL analyses,* 3rd paragraph: reference to secondary endpoint number

Item 7 Deleted in Section 9.3, *Enrollment and vaccination visits for Main Cohort, Immediate Vaccination Group* and Section 9.5, *Enrollment (visit 2) and visit 4 (Month 2) for Main Cohort, Delayed Vaccination Group:* sentence regarding clinical lab results

Item 8 Added in Section 9.15, *Use of electronic Diary (eDiary) for Clinical Outcomes Assessments,* and Appendices F through G, *Procedures*: end of study questionnaire regarding COVID vaccines received outside the study

Item 9 Added in Appendices A, B, and C, *Sample informed consent forms*, section “Being in the study”: participants will take an end of study questionnaire

Item 10 Added in Appendix B, *Sample informed consent form for Prospective Close Contact (PCC) cohort*, and Appendix C, *Sample informed consent form for Case-Ascertained Close Contact (CACC) cohort*, section “Being in the study”:

applicable participants will receive SARS-CoV-2 screening at the University about two times per week.

Item 11 Deleted in Appendices A, B, and C, *Sample informed consents,* second sentence*:* reference to alternate study name of "vPROTECT"

Item 12 Added in Appendix A, *Sample informed consent form for the main cohort:* additional information for participant regarding text or email reminders for nasal swabs

Item 13 Deleted in Appendices A, B and C, *Sample informed consents:* reference to "electronically" for signature

Item 14 Deleted in Appendices A, B and C, *Sample informed consents*, signature table: column for denoting time of signature

Item 15 Deleted in Appendix A, *Sample informed consent form for the main cohort*, section 8: redundant information

Item 16 Corrected in in Appendix A, *Sample informed consent form for the main cohort*, section10: VAERS reporting requirements

Item 17 Revised in Appendices F and G: table text and associated footnotes

Item 18 Revised Appendix H, *Visit windows,* Case-Ascertained Close Contact (CACC) cohort, to include applicable Prospective Close Contact (PCC) participants who test positive for SARS-CoV-2 or whose Main Cohort study contact tests positive for SARS-CoV-2

Item 19 Revised throughout protocol: minor grammatical, editorial, and formatting corrections.

Item 20 Updated Title page, Table of Contents, Section 13, *Version history*, and Appendix I*, Protocol signature page*: dates, version number, and contents of this amendment

Date: February 17, 2021

Protocol version:1.0
Protocol modification: Not applicable

Original protocol

14 Document references (other than literature citations)

Other documents referred to in this protocol, and containing information relevant to the conduct of this study, include:

- Assessment of Understanding. Accessible through the CoVPN protocol-specific website.
- CDC COVID-19 Vaccination Program Provider Requirements and Support. Available at https://www.cdc.gov/vaccines/covid-19/vaccination-provider-support.html
- CoVPN Certificate of Confidentiality. Accessible through the CoVPN website.
- CoVPN 3006 Study Specific Procedures. Accessible through the CoVPN protocol-specific website.
- Dangerous Goods Regulations (updated annually), International Air Transport Association. Available for purchase at https://www.iata.org/publications/dgr/Pages/index.aspx
- International Council on Harmonisation of Technical Requirements for Pharmaceuticals for Human Use (ICH) E6 (R2), Guideline for Good Clinical Practice: Section 4.8, Informed consent of trial subjects. Available at http://www.ich.org/page/efficacy-guidelines
- Laboratory Center assay portfolio
- Moderna COVID-19 Vaccine EUA Fact Sheet and Full PI for Vaccination Providers. Available at https://www.modernatx.com/covid19vaccine-eua/.
- Participants' Bill of Rights and Responsibilities. Accessible through the CoVPN protocol-specific website.
- NIH Policy on Reporting Race and Ethnicity Data: Subjects in Clinical Research. Available at https://grants.nih.gov/grants/guide/notice-files/NOT-OD-01-053.html
- Title 21, Code of Federal Regulations, Part 50. Available at http://www.accessdata.fda.gov/scripts/cdrh/cfdocs/cfcfr/CFRSearch.cfm?CFRPart=50
- Title 45, Code of Federal Regulations, Part 46 (2018 requirements). Current requirements available at https://www.hhs.gov/ohrp/regulations-and-policy/regulations/45-cfr-46/index.html
- VAERS, Vaccine Adverse Event Reporting System. Available at https://vaers.hhs.gov/reportevent.html

- VAERS, Vaccine Adverse Event Reporting System FAQ. Available at https://vaers.hhs.gov/faq.html

- V-safe After Vaccination Health Checker. Available at https://www.cdc.gov/coronavirus/2019-ncov/vaccines/safety/vsafe.html

See Section 16 for literature cited in the background and statistics sections of this protocol.

15 Acronyms and abbreviations

Ab	antibody
ACIP	Advisory Committee on Immunization Practices
AE	adverse event
AUC	area under the curve
BAMA	binding antibody multiplex assay
CAB	Community Advisory Board
CDC	US Centers for Disease Control and Prevention
CFR	Code of Federal Regulations
CI	confidence intervals
COVID-19	Coronavirus disease 2019
CoVPN	COVID-19 Prevention Network
CRF	case report form
CRPMC	NIAID Clinical Research Products Management Center
CRS	clinical research site
DAIDS	Division of AIDS (US NIH)
DHHS	US Department of Health and Human Services
EAE	adverse events requiring expedited reporting
EC	Ethics Committee
eDiary	Electronic diary
ELISA	enzyme-linked immunosorbent assay
EUA	emergency use authorization
FAS	Full analysis set
FDA	US Food and Drug Administration
Fred Hutch	Fred Hutchinson Cancer Research Center
GCP	Good Clinical Practice
GMT	geometric mean titer
HIPAA	Health Insurance Portability and Accountability Act
HIV	human immunodeficiency virus
IB	Investigator's Brochure
IBC	Institutional Biosafety Committee
ICH	International Council on Harmonisation of Technical Requirements for Pharmaceuticals for Human Use
IQR	Interquartile range
IDMC	Independent Data Monitoring Committee
IM	Intramuscular
IRB	Institutional Review Board
LC	Laboratory Center

LNP	Lipid nanoparticle
LOC	Leadership and Operations Center
mRNA	Messenger ribonucleic acid
nAb	neutralizing antibody
NHP	nonhuman primate
NIAID	National Institute of Allergy and Infectious Diseases (US NIH)
NIH	US National Institutes of Health
OHRP	US Office for Human Research Protections
PCR	polymerase chain reaction
PI	Principal Investigator
RE	regulatory entity
SAP	Statistical Analysis Plan
SCHARP	Statistical Center for HIV/AIDS Research and Prevention
SDMC	statistical and data management center
SSP	Study specific procedures
SOP	Standard operating procedure
UW-VL	University of Washington Virology Laboratory
UW-VSL	University of Washington Virology Specialty Laboratory
VAERS	Vaccine Adverse Event Reporting System
VE	Vaccine efficacy
VE_I	Vaccine efficacy against infectiousness
VEs	Vaccine efficacy against susceptibility
VL	Viral load

16 Literature cited

1. Johns Hopkins Coronavirus Resource Center. 2020 [Available from: https://coronavirus.jhu.edu/map.html.

2. Lipsitch M, Dean NE. Understanding COVID-19 vaccine efficacy. Science. 2020.

3. Mehrotra DV, Janes HE, Fleming TR, Annunziato PW, Neuzil KM, Carpp LN, et al. Clinical Endpoints for Evaluating Efficacy in COVID-19 Vaccine Trials. Ann Intern Med. 2020.

4. Long QX, Tang XJ, Shi QL, Li Q, Deng HJ, Yuan J, et al. Clinical and immunological assessment of asymptomatic SARS-CoV-2 infections. Nat Med. 2020;26(8):1200-4.

5. Salvatore PP, Sula E, Coyle JP, Caruso E, Smith AR, Levine RS, et al. Recent Increase in COVID-19 Cases Reported Among Adults Aged 18-22 Years - United States, May 31-September 5, 2020. MMWR Morb Mortal Wkly Rep. 2020;69(39):1419-24.

6. Boehmer TK, DeVies J, Caruso E, van Santen KL, Tang S, Black CL, et al. Changing Age Distribution of the COVID-19 Pandemic - United States, May-August 2020. MMWR Morb Mortal Wkly Rep. 2020;69(39):1404-9.

7. CDC: Centers for Disease Control and Prevention. COVID-19 Hospitalization and Death by Age 2020 [Available from: https://www.cdc.gov/coronavirus/2019-ncov/covid-data/investigations-discovery/hospitalization-death-by-age.html.

8. Halloran ME, Longini IM, Jr., Struchiner CJ. Design and interpretation of vaccine field studies. Epidemiol Rev. 1999;21(1):73-88.

9. Swan DA, Goyal A, Bracis C, Moore M, Krantz E, Brown E, et al. Vaccines that prevent SARS-CoV-2 transmission may prevent or dampen a spring wave of COVID-19 cases and deaths in 2021. medRxiv. 2020.

10. Swan DA, Bracis C, Janes H, Moore M, Matrajt L, Reeves DB, et al. COVID-19 vaccines that reduce symptoms but do not block infection need higher coverage and faster rollout to achieve population impact. medRxiv. 2020.

11. Datta S, Halloran ME, Longini IM, Jr. Efficiency of estimating vaccine efficacy for susceptibility and infectiousness: randomization by individual versus household. Biometrics. 1999;55(3):792-8.

12. Milani GP, Dioni L, Favero C, Cantone L, Macchi C, Delbue S, et al. Serological follow-up of SARS-CoV-2 asymptomatic subjects. Sci Rep. 2020;10(1):20048.

13. Piccoli L, Park YJ, Tortorici MA, Czudnochowski N, Walls AC, Beltramello M, et al. Mapping Neutralizing and Immunodominant Sites on the SARS-CoV-2 Spike Receptor-

Binding Domain by Structure-Guided High-Resolution Serology. Cell. 2020;183(4):1024-42 e21.

14. Vaccines and Related Biological Products Advisory Committee Meeting. Sponsor Briefing Document, Moderna COVID-19 Vaccine 2020 [Available from: https://www.fda.gov/media/144452/download.

15. Vaccines and Related Biological Products Advisory Committee Meeting. Sponsor Briefing Document Addendum,Moderna COVID-19 Vaccine 2020 [Available from: https://www.fda.gov/media/144453/download.

16. Flood S, King M, Rodgers R, Ruggles S, Warren J. Integrated Public Use Microdata Series, Current Population Survey: version 7.0 [dataset]. 2020.

17. The New York Times. Tracking the Coronavirus at U.S. Colleges and Universities 2020 [Available from: https://www.nytimes.com/interactive/2020/us/covid-college-cases-tracker.html.

18. Business Insider. The 10 biggest coronavirus outbreaks on college campuses across the US since the fall semester began 2020 [Available from: https://www.businessinsider.com/10-colleges-with-fall-coronavirus-outbreaks-1200-cases-or-more-2020-9.

19. Wilson E, Donovan CV, Campbell M, Chai T, Pittman K, Sena AC, et al. Multiple COVID-19 Clusters on a University Campus - North Carolina, August 2020. MMWR Morb Mortal Wkly Rep. 2020;69(39):1416-8.

20. Buitrago-Garcia D, Egli-Gany D, Counotte MJ, Hossmann S, Imeri H, Ipekci AM, et al. Occurrence and transmission potential of asymptomatic and presymptomatic SARS-CoV-2 infections: A living systematic review and meta-analysis. PLoS Med. 2020;17(9):e1003346.

21. Goyal A, Reeves DB, Cardozo-Ojeda EF, Schiffer JT, Mayer BT. Wrong person, place and time: viral load and contact network structure predict SARS-CoV-2 transmission and super-spreading events. medRxiv. 2020.

22. He X, Lau EHY, Wu P, Deng X, Wang J, Hao X, et al. Temporal dynamics in viral shedding and transmissibility of COVID-19. Nat Med. 2020;26(5):672-5.

23. Tindale LC, Stockdale JE, Coombe M, Garlock ES, Lau WYV, Saraswat M, et al. Evidence for transmission of COVID-19 prior to symptom onset. Elife. 2020;9.

24. Kissler SM, Fauver JR, Mack C, Tai C, Shiue KY, Kalinich CC, et al. Viral dynamics of SARS-CoV-2 infection and the predictive value of repeat testing. medRxiv. 2020.

25. CDC: Centers for Disease Control and Prevention. Scientific Brief: Community Use of Cloth Masks to Control the Spread of SARS-CoV-2 2020 [Available from: https://www.cdc.gov/coronavirus/2019-ncov/more/masking-science-sars-cov2.html.

26. Jung CY, Park H, Kim DW, Choi YJ, Kim SW, Chang TI. Clinical Characteristics of Asymptomatic Patients with COVID-19: A Nationwide Cohort Study in South Korea. Int J Infect Dis. 2020;99:266-8.

27. Poteat T, Millett GA, Nelson LE, Beyrer C. Understanding COVID-19 risks and vulnerabilities among black communities in America: the lethal force of syndemics. Ann Epidemiol. 2020;47:1-3.

28. Davies NG, Klepac P, Liu Y, Prem K, Jit M, group CC-w, et al. Age-dependent effects in the transmission and control of COVID-19 epidemics. Nat Med. 2020;26(8):1205-11.

29. Richmond CS, Sabin AP, Jobe DA, Lovrich SD, Kenny PA. SARS-CoV-2 sequencing reveals rapid transmission from college student clusters resulting in morbidity and deaths in vulnerable populations. medRxiv. 2020.

30. Regeneron Pharmaceuticals. Regeneron's REGN-CoV2 antibody cocktail reduced viral levels and improved symptoms in non-hospitalized COVID-19 patients 2020 [Available from: https://newsroom.regeneron.com/news-releases/news-release-details/regenerons-regn-cov2-antibody-cocktail-reduced-viral-levels-and.

31. Chen P, Nirula A, Heller B, Gottlieb RL, Boscia J, Morris J, et al. SARS-CoV-2 Neutralizing Antibody LY-CoV555 in Outpatients with Covid-19. N Engl J Med. 2020.

32. Heneghan C, Brassey J, Jefferson T. SARS-CoV-2 viral load and the severity of COVID-19. The Centre for Evidence-Based Medicine. 2020.

33. Pujadas E, Chaudhry F, McBride R, Richter F, Zhao S, Wajnberg A, et al. SARS-CoV-2 viral load predicts COVID-19 mortality. Lancet Respir Med. 2020;8(9):e70.

34. Westblade LF, Brar G, Pinheiro LC, Paidoussis D, Rajan M, Martin P, et al. SARS-CoV-2 Viral Load Predicts Mortality in Patients with and without Cancer Who Are Hospitalized with COVID-19. Cancer Cell. 2020;38(5):661-71 e2.

35. Letizia AG, Ramos I, Obla A, Goforth C, Weir DL, Ge Y, et al. SARS-CoV-2 Transmission among Marine Recruits during Quarantine. N Engl J Med. 2020.

36. Datta S, Halloran ME, Longini IM, Jr. Augmented HIV vaccine trial design for estimating reduction in infectiousness and protective efficacy. Stat Med. 1998;17(2):185-200.

37. Halloran ME, Struchiner CJ. Causal inference in infectious diseases. Epidemiology. 1995;6(2):142-51.

38. Yang Y, Longini IM, Jr., Halloran ME. Design and Evaluation of Prophylactic Interventions Using Infectious Disease Incidence Data from Close Contact Groups. J R Stat Soc Ser C Appl Stat. 2006;55(3):317-30.

39. Klick B, Leung GM, Cowling BJ. Optimal design of studies of influenza transmission in households. I: case-ascertained studies. Epidemiol Infect. 2012;140(1):106-14.

40. Barnabas RV, Brown ER, Bershteyn A, Stankiewicz Karita HC, Johnston C, Thorpe LE, et al. Hydroxychloroquine as Postexposure Prophylaxis to Prevent Severe Acute Respiratory Syndrome Coronavirus 2 Infection : A Randomized Trial. Ann Intern Med. 2020.

41. Jackson LA, Anderson EJ, Rouphael NG, Roberts PC, Makhene M, Coler RN, et al. An mRNA Vaccine against SARS-CoV-2 - Preliminary Report. N Engl J Med. 2020.

42. Anderson EJ, Rouphael NG, Widge AT, Jackson LA, Roberts PC, Makhene M, et al. Safety and Immunogenicity of SARS-CoV-2 mRNA-1273 Vaccine in Older Adults. N Engl J Med. 2020;383(25):2427-38.

43. Vaccines and Related Biological Products Advisory Committee Meeting. FDA Briefing Document, Moderna COVID-19 Vaccine 2020 [Available from: https://www.fda.gov/media/144434/download.

44. UNAIDS. Ethical considerations in biomedical HIV prevention trials. 2007 7/2007.

45. The National Commission for the Protection of Human Subjects of Biomedical and Behavioral Research. The Belmont Report: Ethical Principles and Guidelines for the Protection of Human Subjects of Research. 1979 4/18/1979.

46. Council for International Organizations of Medical Sciences (CIOMS). International ethical guidelines for biomedical research involving human subjects. Bull Med Ethics. 2002(182):17-23.

47. Sun K, Wang W, Gao L, Wang Y, Luo K, Ren L, et al. Transmission heterogeneities, kinetics, and controllability of SARS-CoV-2. Science. 2021;371(6526).

48. Li F, Li YY, Liu MJ, Fang LQ, Dean NE, Wong GWK, et al. Household transmission of SARS-CoV-2 and risk factors for susceptibility and infectivity in Wuhan: a retrospective observational study. Lancet Infect Dis. 2021.

49. Madewell ZJ, Yang Y, Longini IM, Jr, Halloran ME, Dean NE. Household Transmission of SARS-CoV-2: A Systematic Review and Meta-analysis. JAMA Network Open. 2020;3(12):e2031756-e.

50. Kissler SM, Fauver JR, Mack C, Olesen SW, Tai C, Shiue KY, et al. SARS-CoV-2 viral dynamics in acute infections. medRxiv. 2020.

51. Grambsch PM, Therneau TM. Proportional Hazards Tests and Diagnostics Based on Weighted Residuals. Biometrika. 1994;81(3):515-26.

52. Hernan MA. The hazards of hazard ratios. Epidemiology. 2010;21(1):13-5.

53. Follmann D, Huang CY. Incorporating founder virus information in vaccine field trials. Biometrics. 2015;71(2):386-96.

54. Lin DY. Proportional means regression for censored medical costs. Biometrics. 2000;56(3):775-8.

Appendix A Sample informed consent form for Main cohort

**Sponsor / Study Title:**	**National Institutes of Health, "A randomized controlled study to assess SARS CoV-2 infection, viral shedding, and subsequent potential transmission in university students immunized with Moderna COVID-19 Vaccine"**
**Protocol Number:**	**CoVPN 3006**
**Principal Investigator: (Study Doctor)**	**«PiFullName»**
**Telephone:**	**«IcfPhoneNumber»**
**Address:**	**«PiLocations»**

Thank you for your interest in our research study. It is called CoVPN 3006. This informed consent form will tell you more about the study. Please read it carefully as you decide if you want to join. If you have questions, please ask us. At the end, we will ask you to answer a few questions to make sure you understand the study.

If you decide to join this study, we will ask you to sign this form. We will offer you a copy of this form to keep.

CoVPN 3006 is a research study. Research is not the same as medical care. The purpose of a research study is to answer scientific questions. We hope that what we learn will help people in the future.

Key information

- Joining this research study is voluntary. It is your choice.
- Our scientific questions are: Does the vaccine protect people from getting infected with a coronavirus called SARS-CoV-2? Does the vaccine prevent people from transmitting SARS-CoV-2 to others?
- If you join, your participation in this study will last for about 5 months.
- If you join, we will ask you to answer questionnaires, get injections, give blood, and take daily swabs of your nose.

  Here are the risks of taking part:

- The most common risk is symptoms such as muscle aches or headaches after getting the injection.

- There is a risk of loss of your personal information.
- There are other, less serious risks. We will tell you more about them later in this consent form.

About the study

The COVID-19 Prevention Network (CoVPN) is doing a study to test a vaccine against SARS-CoV-2. SARS-CoV-2 is the virus that causes the disease called COVID-19. We want to know if the vaccine is able to protect people from getting infected and if getting the vaccine will affect the amount of virus that is in your nose. We also want to know if getting the vaccine will prevent transmitting SARS-CoV-2 to others.

Many people will take part in this study. About 12,000 people will get vaccine injections while about 24,000 will be in the Prospective Close Contact (PCC) group and up to about 1500 more will be in the Case-ascertained Close Contact (CACC) group. These close contact groups will help researchers determine if the vaccine given to you prevents transmission of the SARS-CoV-2 virus to others. We will explain more about this later in this form.

We are asking you to join the Main part of the study in which people get vaccine injections. People who join the Main study will be recruited from universities in the United States (US). The researcher in charge of this study at this clinic is [Insert name of site PI]. The US National Institutes of Health is paying for this study.

1. **This study's vaccine has an Emergency Use Authorization (EUA) for individuals 18 and older.**

The vaccine we will use in this study is called the Moderna COVID-19 Vaccine. The vaccine has been given to over 15,000 people in other research studies and millions more people under EUA. We will give you the most up-to-date "Fact Sheet for Recipients and Caregivers" with more information about the Moderna COVID-19 Vaccine. The person giving you the vaccine can answer any questions you might have about what is in this fact sheet.

We already know that the vaccine can prevent serious COVID-19 disease, but we do not know if the vaccine will work to prevent SARS-CoV-2 infection in others. That is what we are testing in this study.

The Moderna COVID-19 Vaccine is not approved for sale in the United States by the US Food and Drug Administration (FDA).

Although the vaccine is not approved, it has an Emergency Use Authorization (EUA) from the FDA based on its success at preventing disease symptoms from SARS-CoV-2 in studies to date. During a public health emergency, like the COVID-19 pandemic, the FDA can issue an EUA to get vaccines to people faster. When the FDA issues an EUA, they continue to monitor safety.

The vaccine was developed by ModernaTX, Inc. Typical vaccines for viruses are made from a weakened or killed virus, but the Moderna COVID-19 Vaccine is not made from the SARS-CoV-2 virus. The vaccine includes a short segment of messenger ribonucleic acid (mRNA). The mRNA is a genetic code that tells cells how to make a protein. This mRNA is made in a laboratory. When injected into the body, the mRNA vaccine causes some cells to make that viral protein, which can trigger the immune system. Your immune system protects you from disease. If a person is later infected, their immune system remembers the protein from the vaccine which may help it to fight the virus. It is impossible for the vaccine to give you SARS-CoV-2 or COVID-19 infection.

Risks of the Moderna COVID-19 Vaccine:

To date, millions of people have already received the Moderna COVID-19 vaccine. In clinical trials, most people who got the vaccine had some reaction after their injections, especially after the second injection. The reaction to the vaccine didn't affect their daily lives and it went away after two or three days. Most of these people said they had pain in the arm where they got the injection. These people also felt tired, had headaches, muscle and joint pain, and chills. A much smaller number of these people said they had redness or swelling where the needle went in their arm.

The risks of the study vaccine seen so far are the same as what have been seen with most vaccines. Generally, vaccines can cause fever, chills, rash, aches and pains, nausea, headache, dizziness, and feeling tired. Vaccines can also cause pain, redness, swelling, or itching where you got the injection. Most people can still do their planned activities after getting a vaccine. Rarely, people have side effects that limit their normal activities or make them go to the doctor.

Rarely, a vaccine can cause you to have an allergic reaction. You might have a rash, hives, or trouble breathing. Allergic reactions can be life-threatening. Tell us if you have ever had a bad reaction to any injection or vaccine.

There may be other risks that we don't yet know about, even serious ones. We will tell you if we learn about any new risks.

Joining the study

2. **You will receive the vaccine immediately or later.**

To join this study, you must agree to not get any COVID-19 licensed or emergency-use authorized (EUA) vaccine until the study staff tell you to. Not everyone in this study will get the vaccine immediately. Some people will get the study vaccine 4 months later than the first group of people in the Main study.

We will compare the results from people who got the vaccine immediately with results from people who got the vaccine later. In this way, we will be able to tell if the vaccine can prevent SARS-CoV-2 infection.

You have a 50/50 chance of getting the vaccine immediately or later. Whether you get the vaccine immediately or a little later is completely random, like flipping a coin.

We have no say in whether you get the vaccine immediately or later.

3. **It is completely up to you whether or not to join this study.**

   Take your time in deciding. If it helps, talk to people you trust, such as your doctor, friends, or family. If you decide not to join this study, or if you leave it after you have joined, it will not change your healthcare or your standing at this university. If you do join, you do not give up your legal rights.

   You cannot be in this study while you are in another study where you get a study product.

   If you choose not to join this study, you may be able to join another study.

4. **If you want to join this study, after you sign this consent, you will download an electronic diary (eDiary) app and take questionnaires in it.**

   We will send you information about how to download the eDiary app onto your phone or tablet. We can help you download the app and show you how to use it.

   To find out if you are eligible to join the study, you will answer questionnaires in the eDiary. Some of the questions will ask about your health history, what medications you take, your age, race, and ethnicity, and where you live. Your answers will tell us about some aspects of your health and if you may be eligible to join the study. It should only take about 10 minutes to complete the questionnaires.

   Your answers to the questions may show you are not eligible to join the study, even if you want to. We might contact you to ask some more questions to further evaluate whether you can join the study.

Being in the study

If you want to join and you are eligible, here is what will happen:

5. **You will be in the study about 5 months.**

   Visits can be done in-person or remotely depending on the type of procedures that need to be done. Visits where you get injections must be done in person. Remote visits will be done whenever possible to reduce potential SARS-CoV-2 exposure.

   You may have to come to the clinic if you have a lab or health issue.

Site: Insert visit lengths.

Visits can last from [#] to [#] hours.

At your first visit, we will give you nasal swab kits and instructions so that you can take daily swabs of your nose. Once you receive your first kit, you will swab your nose every day, and you will record it in the eDiary for the entire study. You will receive reminders by text message or email each day to do your swabs. In all, you will swab both of your nostrils every day for about 4 months. You can ask us questions if you need help. You will return your swabs using drop-off location(s) specified by the clinic or by mail.

If you are in the immediate vaccination group, we will take blood samples in the clinic at your first injection visit and 2 more times, two months and four months later. When we take blood, the amount will depend on the lab tests we need to do. Each time we take your blood, the total amount we will take will not be more than two tablespoons. If for some reason you cannot come to the clinic, you may be asked to self-collect blood using a small device called the Tasso-SST OnDemand. Each time you collect your own blood, the amount will be less than one teaspoon. We will give you everything you need to take the blood and return it to us. We will be looking at how your body responds to the vaccine and if you been exposed to the SARS-CoV-2 virus.

If you are in the later vaccination group, we will take blood samples in the clinic at the first visit, and again two months and 4 months later. Each time we take your blood, the total amount we will take will not be more than one teaspoon. If for some reason you cannot come to the clinic, you may be asked to self-collect blood using a small device called the Tasso-SST OnDemand. Each time you collect your own blood, the amount will be less than one teaspoon. We will give you everything you need to take the blood and return it to us. We will be looking to see if you been exposed to the SARS-CoV-2 virus.

The self collection device called the Tasso-SST OnDemand sticks to the skin with a light adhesive. When the button is pressed, a vacuum forms and the device will prick the surface of the skin. The vacuum draws blood out of the capillaries and into a sample pod attached to the bottom of the device.

You will also take a daily questionnaire in the eDiary that asks about the results of your SARS-CoV-2 tests taken at the university or another location. The daily questionnaires will take about 5 minutes to complete. You will also take an End of Study questionnaire in the eDiary. The eDiary will send your phone or tablet a push notification or you will receive a reminder email.

6. **We will give you the vaccine on a schedule.**

   You will be in one of 2 groups: The Immediate Vaccination group or the Later Vaccination group. Regardless of which group you are in, you will get 1 injection into your upper arm at 2 separate visits during the study.

   We will ask you to sign up for v-safe to report any reactions to the study vaccine. V-safe is a smartphone-based tool from the Center for Disease Control (CDC) that uses text messaging and web surveys to check in with you after your COVID-19 vaccination.

**Procedures for Immediate Vaccination group**	**Screening visit(s)**	**First injection visit**	**1st month**	**2nd month**	**4th month**
Injection		√	√		
Medical history questionnaire	√	√			
SARS-CoV-2 screening at the University		About 2x per week			
eDiary app	√	Daily through month 4			
Blood drawn		√		√	√
Nasal swab		Daily through month 4			

**Procedures for Later Vaccination group**	**Screening visit(s)**	**1st Day**	**2nd month**	**First injection visit (4th month)**	**5th month**
Injection				√	√
Medical history questionnaire	√	√			
SARS-CoV-2 screening at the University		About 2x per week			
eDiary app	√	Daily through month 4			√
Blood drawn		√	√	√	
Nasal swab		Daily through month 4			

7. **We will compensate you for your participation.**

Site: Insert your compensation plan here, e.g. money for number of swabs returned. Remember to include any costs to participants.

8. **In addition to giving you the vaccine:**

We will ask you to give a referral code to people with whom you expect to be in frequent close physical proximity during the study, like a roommate or co-worker. We want to ask these people to enroll in another part of this study that will help us find out if the vaccine prevents transmission of the SARS-CoV-2 virus. We are calling this group the Prospective Close Contact group or PCC.

As part of their study participation, they will be asked to complete a weekly eDiary that includes questionnaires like the ones you completed. If you become infected with SARS-CoV-2, we will ask you to share your status with your PCCs. Then your PCC participants will be asked to fill out additional questionnaires for 2 weeks as well as take nasal swabs and self-collect blood samples.

When considering who to ask to be your PCC, you should think about whether you feel comfortable or safe sharing your infection status with them if you get SARS-CoV-2. If you don't feel comfortable sharing this information with them, you should not ask them to participate as a PCC.

It is possible that someone you know may also be participating in the Main part of the study and might identify you as a close contact. They will not know you are also participating in the study unless you choose to tell them. If someone else identifies you as a close contact, they will give you their referral code. It is okay to be enrolled in both the Main part of the study and the PCC group.

We may also ask you for the name of someone who can tell us how you are doing if we can't reach you or if you are unable to talk. We may contact you after the main study ends (for example, to tell you about the study results).

We will ask you to continue to follow the university's program for SARS-CoV-2 testing and contact tracing. If you test positive for SARS-CoV-2, please tell us as soon as possible.

9. **The CoVPN will test your samples to see how your body, including your immune system, responds to the Moderna COVID-19 vaccine.**

We will send your samples to labs approved by the CoVPN. Your samples will not be labeled with your name or other identifying information.

Researchers may also do genetic testing related to this study on your samples. Your genes are passed to you from your birth parents. Differences in people's genes can help explain why some people get a disease while others do not. The genetic testing will only involve some of your genes, not all of your genes (your genome). The researchers will study only the genes related to the immune system and coronavirus, and genes that may affect how people get coronavirus.

If you get SARS-CoV-2, the researchers may look at all of the virus' genes that are in your samples. The researchers will use this information to learn more about SARS-CoV-2 and how the virus is impacted by the study vaccine.

The tests the researchers do on your samples are for research purposes, not to check your health. The labs will not give the results to you or this clinic because their tests are not approved for use in making health care decisions. These labs are only approved to do research tests.

When your samples are no longer needed for this study, the CoVPN will continue to store them.

10. **We will do our best to protect your private information.**

US sites: Check Health Insurance Portability and Accountability Act (HIPAA) authorization for conflicts with this section.

We will keep your study records and samples in a secure location. We will label all of your samples and most of your records with a code number, not your name or other personal information. We will not share your name with anyone who does not need to know it.

Your records may also be reviewed by groups who watch over this study. These groups include:

- Study monitors
  - The CoVPN, people who work for it, and companies that help it with this study
  - Some government agencies:
    - The US National Institutes of Health
    - The US Office for Human Research Protections
    - Any regulatory agency that reviews research studies
  - Some committees that make sure we protect your rights and keep you safe:
    - The Independent Data Monitoring Committee
    - Advarra Institutional Review Board (IRB)
    - *Sites may, but are not required to include:* [Insert name of local IRB/EC]
    - *Sites include:* [Insert name of local IBC]

All reviewers will take steps to keep your records private.

We cannot guarantee absolute privacy. If you are found to have a medical condition that we are required to report by law, then some of your information may be shared.

At this clinic, we have to report the following information:

Site: Include any public health or legal reporting requirements. Bulleted examples should include all appropriate cases (reportable communicable disease, risk of harm to self or others, etc.)

- SARS-CoV-2
- Pneumonia
- After receiving the vaccine, we are required to report any of the following that may occur to the FDA/CDC Vaccine Adverse Event Reporting System (VAERS):
  - Vaccine administration errors
  - Serious reaction after receiving the vaccine (for example that results in death, hospitalization, a life-threatening reaction, a birth defect, or other important medical event)
  - A condition called "Multisystem Inflammatory Syndrome"
  - Cases of COVID-19 that result in hospitalization

US sites: Include the following boxed text. You can remove the box around the text.

We have a Certificate of Confidentiality from the US government to help protect your privacy. With the certificate, we do not have to release information about you to someone who is not connected to the study, such as the courts or police. Sometimes we can't use the certificate. Since the US National Institutes of Health funds this research, we cannot withhold information from it.

We will not share your name or information that can identify you with the CoVPN. The CoVPN may share information from this study with other researchers. Researchers may publish the results of this study.

Site: The text below may not be deleted or changed per FDA requirement. You can remove the box.

A description of this clinical trial will be available on http://www.ClinicalTrials.gov, as required by U.S. Law. This website will not include information that can identify you. At most, the website will include a summary of the results. You can search this website at any time.

11. **We may take you out of the study at any time.**

We may take you out of the study if:

- you do not follow instructions,
- we think that staying in the study might harm you,
- the study is stopped for any reason.

12. **If you get COVID-19, we will ask you to do several things.**

If you find out that you have COVID-19, please let us know right away. We will ask you to quarantine per local guidelines. By agreeing to join this study, you also agree to allow this university to share your test results with us.

We will ask you to tell anyone with whom you have recently had close physical contact that they may have been exposed to COVID-19. We will give you a referral code to give to these contacts so that they can decide if they would like to join the Case Ascertained Close Contact group (CACC). You may remember at the beginning of this form that we told you that about 1,500 people will be enrolled in the CACC group. The CACC group will help us learn if people who got the study vaccine are less likely to give SARS-CoV-2 to someone else. When considering who to ask to be your CACC, you should think about whether you feel comfortable or safe sharing your infection status with them if you get SARS-CoV-2. If you don't feel comfortable sharing this information with them, you should not ask them to participate as a CACC.

It is possible that someone you know may also be participating in the Main part of the study and might identify you as a close contact. They will not know you are also participating in the study unless you choose to tell them. If someone else identifies you as a close contact, they will give you their referral code. It is okay to be enrolled in both the Main part of the study and the CACC group.

If you find out that you have COVID-19, you will be directed to contact the clinic, and we will ask you to either come to the clinic to have your blood drawn or we will ask you to self-collect less than one teaspoon of blood and complete one additional eDiary questionnaire. It is important that you give the blood sample as soon as possible after finding out the you have COVID-19.

We will ask you to track your COVID-19 symptoms using the eDiary daily for 2 weeks or until the symptoms go away.

After you get better, we will ask you to continue your study participation so that we can monitor your health and safety for the rest of the study.

If you are hospitalized, we will pause your participation. We will ask for your medical records and results from tests you might have received at the hospital. It is unlikely that you will be hospitalized, but to prepare for this, we will ask you to sign and date a medical release of information so we can get records from your doctor or view your hospital records. When you are released from the hospital, please let us know. We will ask you to resume your study participation so that we can continue to monitor your health and safety for the rest of the study.

Site: Modify the following sentence as appropriate. We will not pay for any of your COVID-19 care directly.

Other Risks

13. **There are other risks to being in this study.**

This section describes the other risks and restrictions we know about. There may also be unknown risks, even serious ones. We will tell you if we learn anything new that may affect your willingness to stay in the study.

Risks of routine medical procedures:

In this study, we will do some routine medical procedures. These are taking blood and giving injections. These procedures can cause bruising, pain, fainting, soreness, redness, swelling, itching, a sore, bleeding, and (rarely) muscle damage or infection where you got the injection.

Risks of swabbing your nose:

The feeling of having a small, soft-tipped swab inserted into your nostril and twirled around may be a little uncomfortable, but it should not be painful. There is a small chance there could be some bleeding, but this is unlikely.

Risks to your personal information:

We will take several steps to protect your personal information. Although the risk is very low, it is possible that your personal information could be given to someone who should not have it. If that happened, you could have stress or anxiety.

Risks of genetic testing:

It is unlikely, but the genetic tests done on your samples could show you may be at risk for certain diseases. If others found out, it could lead to discrimination or other problems.

Risk of anxiety/emotional stress:

You may feel anxiety or emotional stress if you experience any of the risks described here. You may feel worried if tests show that you have SARS-CoV-2. Some of the questions we will ask you may make you feel uncomfortable.

Unknown risks:

The vaccine has been shown to prevent adults from getting COVID-19 for at least 2 to 3 months after their injection, but we do not know if it will protect them for any longer. If you get COVID-19, we do not know how the vaccine might affect your illness. It is also unknown if the vaccine will prevent you from giving the virus to someone else. If you get COVID-19, we do not know how the vaccine might affect your illness.

We do not know if getting this vaccine will affect how you respond to any future SARS-CoV-2 vaccines.

We do not know if getting the vaccine will affect pregnancy or breastfeeding. If you have concerns, we recommend that you discuss this with a health care provider.

While you are in this study, you should still wear a mask in public and practice physical distancing.

Benefits

14. **This study may benefit you.**

    This study may help you receive a COVID-19 vaccine earlier than you would otherwise be eligible. This study may help researchers find out if the vaccine is able to prevent infection with SARS-CoV-2. If the vaccine later becomes approved and sold, there are no plans to share any money with you.

    If you decide not to join this study, you could wait to get the Moderna COVID-19 vaccine or another COVID-19 vaccine in your community. You can also continue to follow local guidelines for SARS-CoV-2 infection. You do not have to join this research study if you don't want to.

Your rights and responsibilities

15. **If you join the study, you have rights and responsibilities.**

    You have many rights that we will respect. You also have responsibilities. We list these in the Bill of Rights and Responsibilities for Research. We will give you a copy of it.

Leaving the study

16. **Tell us if you decide to leave the study.**

    You are free to leave the study at any time and for any reason. If you want to leave, you will need to tell us. Your care at this clinic, your standing at this university, and your legal rights will not be affected.

    Previously collected information about you will remain in the study records and will be included in the analysis of results. Your information can not be removed from the study records.

    We may ask you to give a final blood sample or nasal swab.

    We believe these steps are important to protecting your health, but it is up to you whether to complete them.

Injuries

Sites: Approval from CoVPN Regulatory Affairs (at CoVPN.core.reg@fredhutch.org) is needed for any change (other than those that the instructions specifically request or those previously approved by CoVPN Regulatory Affairs) to the boxed text. You can remove the box around the text.

17. **If you get sick or injured during the study, contact us immediately.**

Paragraph below for all sites.

Your health is important to us. (*Sites: adjust the following 2 sentences if applicable to the care available at your site)* We will tell you about the care that we can give here. For the care that we cannot provide, we will explain how we will help you get care elsewhere.

2 paragraphs below for all sites.

This research study is covered by a US government program that may provide compensation for a serious injury or death. We will give you a handout with more information about this program. The National Institutes of Health (NIH) does not have a mechanism to provide direct compensation for research related injury.

Some injuries are not physical. For example, you might be harmed emotionally by being in this study. Or you might lose wages because you cannot go to work. However, there are no funds to pay for these kinds of injuries, even if they are study related.

You always have the right to use the court system if you are not satisfied.

18. **When samples are no longer needed for this study, the CoVPN wants to use them in other studies and share them with other researchers.**

The CoVPN calls these samples "extra samples". The CoVPN will only allow your extra samples to be used in other studies if you agree to this. You will mark your decision at the end of this form.

Do I have to agree? No. You are free to say yes or no, or to change your mind after you sign this form. At your request, the CoVPN will destroy all extra samples that it has. Your decision will not affect your being in this study.

Where are the samples stored? Extra samples are stored in a secure central place called a repository. Your samples will be stored in the CoVPN repository in the United States.

How long will the samples be stored? There is no limit on how long your extra samples will be stored. *[Site: Revise the previous sentence to insert limits if your regulatory authority imposes them.]*

Will I be paid for the use of my samples? No. Researchers may make scientific discoveries or products using your samples. If this happens, there is no plan to share any money with you.

Will I benefit from allowing my samples to be used in other studies? Probably not. Results from these other studies are not given to you, this clinic, or your doctor. They may help other people in the future.

Will the CoVPN sell my samples and information? No, but the CoVPN may share your samples with other researchers. Once the CoVPN shares your samples and information, it may not be able to get them back.

What information is shared with researchers? The samples and information will be labeled with a code number which will not be removed. The key to the code will stay at this clinic. However, some information that the CoVPN shares may be personal, such as your race, ethnicity, sex, and health information from the study.

What kind of studies might be done with my extra samples and information? The studies will be related to vaccines, the immune system, coronavirus, and other diseases. Researchers may also do genetic testing on your samples.

If you agree, researchers may compare all of your genes (your genome) to the genomes of many other people. Researchers look for common patterns of genes to help them understand diseases. The researchers may put the information into a protected database so that other researchers can access it. Your name and other personal information will not be included.

Usually, no one could connect your genome to you as a person. There are rules against this. It's also really difficult to do. But there is a risk that someone could combine information from your genome and other public information about you and identify you. If others found out, it could lead to discrimination or other problems. The risk of this happening is very small.

Who will have access to my information in studies using my extra samples?

People who may see your information are:

- Researchers who use your extra samples and information for other research
- Government agencies that fund or monitor the research using your extra samples and information
- The researcher's Institutional Review Board or Ethics Committee
- Any regulatory agency that reviews research studies
- The people who work with the researcher

All of these people will do their best to protect your information. If they publish their research, they will not use your name or identify you personally.

Questions

19. **Whom to contact about this study**

During the study, if you experience any medical problems, suffer a research-related injury, or have questions, concerns, or complaints about the study, please contact the study doctor at the telephone number listed on the first page of this consent document. If you seek emergency care, or hospitalization is required, alert the treating physician that you are participating in this research study.

An institutional review board (IRB) is an independent committee established to help protect the rights of research subjects.

If you have any questions about your rights as a research subject, and/or concerns or complaints regarding this research study, contact:
by mail:
Study Subject Adviser
Advarra IRB
6940 Columbia Gateway Drive, Suite 110
Columbia, MD 21046
or call **toll free**: 877-992-4724
or by **email**: adviser@advarra.com

Please reference the following number when contacting the Study Subject Adviser: [Advarra to insert number]

US sites may include, but are not required to: You may also contact the [name of local IRB/EC] at [insert contact information].

Your permissions and signature

20. **In Section 18, we told you above about possible other uses of your extra samples** and information outside this study. Please choose only one of the options below and write your initials in the box next to it. Whatever you choose, the CoVPN keeps track of your decision about how your samples and information can be used. You can change your mind after signing this form.

☐ I allow my extra samples and information to be used for other studies related to vaccines, the immune system, coronavirus, and other diseases. This may include genetic testing.

OR

☐ I agree to the option above *and* also to allow my extra samples and information to be used in studies that look at my whole genome.

OR

☐ I do not allow my extra samples to be used in any other studies. This includes not allowing genetic testing, or studies that look at my whole genome.

21. **If you agree to join this study, you will need to sign below. Before you sign, make sure of the following:**

- You have read this consent form.
- You feel that you understand what the study is about and what will happen to you if you join. You understand what the possible risks and benefits are.
- You have had your questions answered and know that you can ask more.
- You agree to join this study.

You will not be giving up any of your rights by signing this consent form.

Participant's Name (Print)	Participant's Signature	Date
Clinic Staff Conducting Consent Discussion (Print)	Clinic Staff Signature	Date

Appendix B Sample informed consent form for Prospective Close Contact (PCC) cohort

**Sponsor / Study Title:**	**National Institutes of Health, "A randomized controlled study to assess SARS CoV-2 infection, viral shedding, and subsequent potential transmission in university students immunized with Moderna COVID-19 Vaccine"**
**Protocol Number:**	**CoVPN 3006**
**Principal Investigator: (Study Doctor)**	**«PiFullName»**
**Telephone:**	**«IcfPhoneNumber»**
**Address:**	**«PiLocations»**

Thank you for your interest in our research study. It is called CoVPN 3006. This informed consent form will tell you more about the study. Please read it carefully to help you decide if you want to join. If you have questions, please ask us.

If you decide to join this study, we will ask you to sign this form. We will offer you a copy of this form to keep.

CoVPN 3006 is a research study. Research is not the same as medical care. The purpose of a research study is to answer scientific questions. We hope that what we learn will help people in the future.

Key information

- Joining this research study is voluntary. It is your choice.
- Our scientific questions are: Does the study vaccine protect people from getting infected with a coronavirus called SARS-CoV-2? Does the study vaccine prevent people from transmitting SARS-CoV-2 to others?
- If you join, your participation in this study will last for about 5 months.
- If you join, we will ask you to answer weekly eDiary questionnaires. If you or your contact in the main study group becomes SARS-CoV-2 infected, then we will ask you to take daily swabs of your nose, answer daily eDiary questionnaire for 2 weeks, and give 2 blood samples.

  Here are the risks of taking part:

- There is a risk of loss of your personal information.
- There are other, less serious risks. We will tell you more about them later in this consent form.

About the study

The COVID-19 Prevention Network (CoVPN) is doing a study to test a vaccine against SARS-CoV-2. SARS-CoV-2 is the virus that causes the disease called COVID-19. We want to know if the vaccine is able to protect people from getting infected and if getting the vaccine will affect the amount of virus that is in your nose. We also want to know if getting the vaccine will prevent transmitting SARS-CoV-2 to others.

We are asking you to join this study because you have been referred as a close contact with someone who is in the Main study and is getting injections of the vaccine.

Many people will take part in this study. About 12,000 people will get injections while about 24,000 will be in the Prospective Close Contact (PCC) group and up to about 1,500 more will be in the Case-ascertained Close Contact (CACC) group. These close contact groups will help researchers determine if the vaccine prevents transmission of the SARS-CoV-2 virus.

People who join the close contact groups and Main study are being recruited from many universities in the United States (US). You are being asked to participate in the PCC group. The researcher in charge of this study at this clinic is [Insert name of site PI]. The US National Institutes of Health is paying for this study.

Joining the study

1. **It is completely up to you whether or not to join this study.**

   Take your time in deciding. If it helps, talk to people you trust, such as your friends or family. If you decide not to join this study, or if you leave it after you have joined, it will not change your healthcare. If you do join, you do not give up your legal rights.

Being in the study

If you want to join, here is what will happen:

We will send you information about how to download an electronic diary (eDiary) app onto your phone or tablet. We can help you download the app and show you how to use it.

Initially, you will answer a 5-minute questionnaire in the eDiary. It will ask you about things such as your sex, age, race, and ethnicity. You will also answer weekly questionnaires about whether you are sick or if you have tested positive for SARS-CoV-2. You will also take an End of Study questionnaire in the eDiary. If you have tested positive, you will enter the results of your SARS-CoV-2 tests taken at the university or another location into the eDiary.

As part of the study, you will get SARS-CoV-2 testing at your university about twice a week.

If you or your contact in the main study group becomes SARS-CoV-2 infected, then we will ask you to take swabs of your nose and answer eDiary questionnaires every day for 2 weeks. You will return your nasal swabs using drop-off location(s) specified by the clinic or by mail.

We will ask you to self-collect a small amount of your blood twice using a small device called the Tasso-SST OnDemand. You will take the first blood sample as soon as possible, and then you will take another sample about a month later. This will be less than one teaspoon of blood taken from one of your upper arms using a small device. We will give you everything you need to take the blood and return it to us.

The self-collection device sticks to the skin with a light adhesive. When the button is pressed, a vacuum forms, and the device will prick the surface of the skin. The vacuum draws blood out of the capillaries and into a sample pod attached to the bottom of the device.

It is important that you continue to follow the local guidelines for SARS-CoV-2 prevention, like wearing a mask and keeping physical distance from others. If you get COVID-19 and are hospitalized, we will pause your participation. We will ask for your medical records and results from tests you might have received at the hospital. It is unlikely that you will be hospitalized, but to prepare for this, we will ask you to sign and date a medical release of information so we can get records from your doctor or view your hospital records. When you are released from the hospital, please let us know. We will ask you to resume your study participation so that we can continue to monitor your health and safety for the rest of the study.

2. **The CoVPN will test your samples to see how your body, including your immune system, responds to the potential infection.**

We will send your samples to labs approved by the CoVPN. Your samples will not be labeled with your name or other identifying information.

Researchers may also do genetic testing related to this study on your samples. Your genes are passed to you from your birth parents. Differences in people's genes can help explain why some people get a disease while others do not. The genetic testing will only involve some of your genes, not all of your genes (your genome). The researchers will study only the genes related to the immune system and coronavirus, and genes that may affect how people get coronavirus.

If you get SARS-CoV-2, the researchers may look at all of the virus' genes that are in your samples. The researchers will use this information to learn more about SARS-CoV-2 and how the virus is impacted by the study vaccine.

The tests the researchers do on your samples are for research purposes, not to check your health. The labs will not give the results to you or this clinic because their tests are not

approved for use in making health care decisions. These labs are only approved to do research tests.

When your samples are no longer needed for this study, the CoVPN will continue to store them.

3. **When samples are no longer needed for this study, the CoVPN wants to use them in other studies and share them with other researchers.**

   The CoVPN calls these samples "extra samples". The CoVPN will only allow your extra samples to be used in other studies if you agree to this. You will mark your decision at the end of this form.

   Do I have to agree? No. You are free to say yes or no, or to change your mind after you sign this form. At your request, the CoVPN will destroy all extra samples that it has. Your decision will not affect your being in this study.

   Where are the samples stored? Extra samples are stored in a secure central place called a repository. Your samples will be stored in the CoVPN repository in the United States.

   How long will the samples be stored? There is no limit on how long your extra samples will be stored. *[Site: Revise the previous sentence to insert limits if your regulatory authority imposes them.]*

   Will I be paid for the use of my samples? No. Researchers may make scientific discoveries or products using your samples. If this happens, there is no plan to share any money with you.

   Will I benefit from allowing my samples to be used in other studies? Probably not. Results from these other studies are not given to you, this clinic, or your doctor. They may help other people in the future.

   Will the CoVPN sell my samples and information? No, but the CoVPN may share your samples with other researchers. Once the CoVPN shares your samples and information, it may not be able to get them back.

   What information is shared with researchers? The samples and information will be labeled with a code number which will not be removed. The key to the code will stay at this clinic. However, some information that the CoVPN shares may be personal, such as your race, ethnicity, sex, and health information from the study.

   What kind of studies might be done with my extra samples and information? The studies will be related to vaccines, the immune system, coronavirus, and other diseases.

   Researchers may also do genetic testing on your samples.

   If you agree, researchers may compare all of your genes (your genome) to the genomes of many other people. Researchers look for common patterns of genes to help them understand

diseases. The researchers may put the information into a protected database so that other researchers can access it. Your name and other personal information will not be included.

Usually, no one could connect your genome to you as a person. There are rules against this. It's also really difficult to do. But there is a risk that someone could combine information from your genome and other public information about you and identify you. If others found out, it could lead to discrimination or other problems. The risk of this happening is very small.

Who will have access to my information in studies using my extra samples?

People who may see your information are:

- Researchers who use your extra samples and information for other research
- Government agencies that fund or monitor the research using your extra samples and information
- The researcher's Institutional Review Board or Ethics Committee
- Any regulatory agency that reviews research studies
- The people who work with the researcher

All of these people will do their best to protect your information. If they publish their research, they will not use your name or identify you personally.

4. **We will compensate you for your participation.**

Site: Insert your compensation plan here, e.g. money for number of swabs returned. Remember to include any costs to participants.

5. **We will do our best to protect your private information.**

Site: Check HIPAA authorization for conflicts with this section.

Your study records and samples will be kept in a secure location. We will label all of your samples and most of your records with a code number, not your name or other personal information. We will not share your name with anyone who does not need to know it.

Your records may also be reviewed by groups who watch over this study. These groups include:

- Study monitors
- The CoVPN, people who work for it, and companies that help it with this study

- Some government agencies:
  - The US National Institutes of Health
  - The US Office for Human Research Protections
  - Any regulatory agency that reviews research studies
- Some committees that make sure we protect your rights and keep you safe:
  - The Independent Data Monitoring Committee
  - Advarra Institutional Review Board (IRB)
  - *Sites may but are not required to include:* [Insert name of local IRB/EC]
  - *Sites include:* [Insert name of local IBC]

All reviewers will take steps to keep your records private.

Sites: Include the following boxed text. You can remove the box around the text.

We have a Certificate of Confidentiality from the US government to help protect your privacy. With the certificate, we do not have to release information about you to someone who is not connected to the study, such as the courts or police. Sometimes we can't use the certificate. Since the US National Institutes of Health funds this research, we cannot withhold information from it.

We will not share your name or information that can identify you with the CoVPN. The CoVPN may share information from this study with other researchers. Researchers may publish the results of this study.

Sites: The text below may not be deleted or changed per FDA requirement. You can remove the box.

A description of this clinical trial will be available on http://www.ClinicalTrials.gov, as required by U.S. Law. This website will not include information that can identify you. At most, the website will include a summary of the results. You can search this website at any time.

6. **We may take you out of the study at any time.**

We may take you out of the study if:

- you do not follow instructions,
- we think that staying in the study might harm you,

- the study is stopped for any reason.

Risks

7. **There are some risks to being in this study.**

   This section describes the risks we know about. There may also be unknown risks, even serious ones. We will tell you if we learn anything new that may affect your willingness to stay in the study.

   Risks of blood draws:

   In this study, we will either take your blood or ask you to self-collect your blood with a small device. Taking blood can cause bruising, pain, fainting, soreness, redness, and itching.

   Risks of swabbing your nose:

   The feeling of having a small, soft-tipped swab inserted into your nostril and twirled around may be a little uncomfortable, but it should not be painful. There is a small chance there could be some bleeding, but this is unlikely.

   Risks to your personal information:

   We will take several steps to protect your personal information. Although the risk is very low, it is possible that your personal information could be given to someone who should not have it. If that happened, you could have stress or anxiety.

Benefits

8. **This study may not benefit you**.

   This study may help researchers find out if people who got the vaccine are less likely to give it to people who they have been in close contact with. If the vaccine later becomes approved and sold, there are no plans to share any money with you.

   If you decide not to join this study, you could wait to get the Moderna COVID-19 vaccine or another COVID-19 vaccine in your community. You can also continue to follow local guidelines for SARS-CoV-2 infection. You do not have to join this research study if you don't want to.

Leaving the study

9. **Tell us if you decide to leave the study.**

   You are free to leave the study at any time and for any reason. If you want to leave, you will need to tell us. Your legal rights will not be affected.

Previously collected information about you will remain in the study records and will be included in the analysis of results. Your information can not be removed from the study records.

We may ask you to give a final blood sample or nasal swab.

We believe these steps are important to protecting your health, but it is up to you whether to complete them.

Injuries

Sites: Approval from CoVPN Regulatory Affairs (at CoVPN.core.reg@fredhutch.org) is needed for any change (other than those that the instructions specifically request or those previously approved by CoVPN Regulatory Affairs) to the boxed text. You can remove the box around the text.

10. If you have a life-threatening medical emergency, call 911 right away or seek help immediately.

We do not expect you to get sick or injured as a result of being in this study. If you get COVID-19 while you are in the study, please let us know. *(Sites: adjust the following 2 sentences if applicable to the care available at your site)* We will tell you about the care that we can give here. For the care that we cannot provide, we will explain how we will help you get care elsewhere. You or your health insurance will have to pay for the care you get elsewhere.

Some injuries are not physical. For example, you might be harmed emotionally by being in this study. Or you might lose wages because you cannot go to work. However, there are no funds to pay for these kinds of injuries, even if they are study related.

You always have the right to use the court system if you are not satisfied.

Questions

11. **Whom to contact about this study**

During the study, if you experience any medical problems, suffer a research-related injury, or have questions, concerns, or complaints about the study, please contact the study doctor at the telephone number listed on the first page of this consent document.

An institutional review board (IRB) is an independent committee established to help protect the rights of research subjects.

If you have any questions about your rights as a research subject, and/or concerns or complaints regarding this research study, contact:

by mail:

Study Subject Adviser
Advarra IRB
6940 Columbia Gateway Drive, Suite 110
Columbia, MD 21046

or call **toll free**: 877-992-4724
or by **email**: adviser@advarra.com

Please reference the following number when contacting the Study Subject Adviser: [Advarra to insert number]

US sites may include, but are not required to: You may also contact the [name of local IRB/EC] at [insert contact information].

Your permissions and signature

12. **In Section 3, we told you above about possible other uses of your extra samples** and information outside this study. Please choose only one of the options below and write your initials in the box next to it. Whatever you choose, the CoVPN keeps track of your decision about how your samples and information can be used. You can change your mind after signing this form.

☐	I allow my extra samples and information to be used for other studies related to vaccines, the immune system, coronavirus, and other diseases. This may include genetic testing.
**OR**	
☐	I agree to the option above *and* also to allow my extra samples and information to be used in studies that look at my whole genome.
**OR**	
☐	I do not allow my extra samples to be used in any other studies. This includes not allowing genetic testing, or studies that look at my whole genome.

13. If you agree to join this study, you will need to sign below. Before you sign, make sure of the following:

- You have read this consent form.
- You feel that you understand what the study is about and what will happen to you if you join. You understand what the possible risks and benefits are.
- You agree to join this study.

You will not be giving up any of your rights by signing this consent form.

Participant's Name (Print)	Participant's Signature	Date
Clinic Staff Conducting Consent Discussion (Print)	Clinic Staff Signature	Date

Appendix C Sample informed consent form for Case-Ascertained Close Contact (CACC) cohort

**Sponsor / Study Title:**	**National Institutes of Health, "A randomized controlled study to assess SARS CoV-2 infection, viral shedding, and subsequent potential transmission in university students immunized with Moderna COVID-19 Vaccine"**
**Protocol Number:**	**CoVPN 3006**
**Principal Investigator: (Study Doctor)**	**«PiFullName»**
**Telephone:**	**«IcfPhoneNumber»**
**Address:**	**«PiLocations»**

Thank you for your interest in our research study. It is called CoVPN 3006. This informed consent form will tell you more about the study. Please read it carefully to help you decide if you want to join. If you have questions, please ask us.

If you decide to join this study, we will ask you to sign this form. We will offer you a copy of this form to keep.

CoVPN 3006 is a research study. Research is not the same as medical care. The purpose of a research study is to answer scientific questions. We hope that what we learn will help people in the future.

Key information

- Joining this research study is voluntary. It is your choice.
- Our scientific questions are: Does the study vaccine protect people from getting infected with a coronavirus called SARS-CoV-2? Does the study vaccine prevent people from transmitting SARS-CoV-2 to others?
- If you join, your participation in this study will last for about 1 month.
- If you join, we will ask you to answer questionnaires, give blood, and take daily swabs of your nose for 2 weeks.

Here are the risks of taking part:

- There is a risk of loss of your personal information.

- There are other, less serious risks. We will tell you more about them later in this consent form.

About the study

The COVID-19 Prevention Network (CoVPN) are doing a study to test a vaccine against SARS-CoV-2. SARS-CoV-2 is the virus that causes the disease called COVID-19. We are asking you to join this study because you recently had close physical contact with someone who is in the Main study and who got infected with SARS-CoV-2. We want to find out if the person you had close physical contact might have transmitted SARS-CoV-2 to you.

Many people will take part in this study. About 12,000 people will get injections while about 24,000 will be in the Prospective Close Contact (PCC) group and up to about 1,500 more will be in the Case-ascertained Close Contact (CACC) group. These close contact groups will help researchers determine if the vaccine prevents transmission of the SARS-CoV-2 virus.

People who join the close contact groups and Main study are being recruited from many universities in the United States (US). You are being asked to participate in the CACC group. The researcher in charge of this study at this clinic is [Insert name of site PI]. The US National Institutes of Health is paying for this study.

Joining the study

1. **It is completely up to you whether or not to join this study.**

   Take your time in deciding. If it helps, talk to people you trust, such as your friends or family. If you decide not to join this study, or if you leave it after you have joined, it will not change your healthcare. If you do join, you do not give up your legal rights.

Being in the study

If you want to join, here is what will happen:

We will send you information about how to download an electronic diary (eDiary) app onto your phone or tablet. We can help you download the app and show you how to use it.

You will take a 5-minute questionnaire in the eDiary. It will ask you about your contact with the person who tested positive for SARS-CoV-2, as well as other things such as your sex, age, race, and ethnicity. You will also take an End of Study questionnaire in the eDiary. If you have tested positive for SARS-CoV-2, you will enter the results of your SARS-CoV-2 tests taken at the university or another location into the eDiary.

If you are affiliated with the University, you may get SARS-CoV-2 testing about twice a week at the university.

We will ask you to take daily swabs of both of your nostrils and track your COVID-19 symptoms in the eDiary daily for two weeks. You will return your nasal swabs using drop-off location(s) specified by the clinic or by mail.

We will also ask you to collect a small amount of blood from one of your upper arms twice during the study using a small device called the Tasso-SST OnDemand. This will be less than one teaspoon of blood taken from one of your upper arms using a small device. You will take the first blood sample as soon as possible, and then you will take another sample about a month later. We will give you everything you need to take these samples and return them to us.

The self-collection device sticks to the skin with a light adhesive. When the button is pressed, a vacuum forms, and the device will prick the surface of the skin. The vacuum draws blood out of the capillaries and into a sample pod attached to the bottom of the device.

It is important that you continue to follow the local guidelines for SARS-CoV-2 prevention, like wearing a mask and keeping physical distance from others.

If you are hospitalized, we will pause your participation. We will ask for your medical records and results from tests you might have received at the hospital. It is unlikely that you will be hospitalized, but to prepare for this, we will ask you to sign and date a medical release of information so we can get records from your doctor or view your hospital records. When you are released from the hospital, please let us know. We will ask you to resume your study participation so that we can continue to monitor your health and safety for the rest of the study.

2. **The CoVPN will test your samples to see how your body, including your immune system, responds to the potential infection.**

   We will send your samples to labs approved by the CoVPN. Your samples will not be labeled with your name or other identifying information.

   Researchers may also do genetic testing related to this study on your samples. Your genes are passed to you from your birth parents. Differences in people's genes can help explain why some people get a disease while others do not. The genetic testing will only involve some of your genes, not all of your genes (your genome). The researchers will study only the genes related to the immune system and coronavirus, and genes that may affect how people get coronavirus.

   If you get SARS-CoV-2, the researchers may look at all of the virus' genes that are in your samples. The researchers will use this information to learn more about SARS-CoV-2 and how the virus is impacted by the study vaccine.

   The tests the researchers do on your samples are for research purposes, not to check your health. The labs will not give the results to you or this clinic because their tests are not approved for use in making health care decisions. These labs are only approved to do research tests.

When your samples are no longer needed for this study, the CoVPN will continue to store them.

3. **When samples are no longer needed for this study, the CoVPN wants to use them in other studies and share them with other researchers.**

The CoVPN calls these samples “extra samples”. The CoVPN will only allow your extra samples to be used in other studies if you agree to this. You will mark your decision at the end of this form.

Do I have to agree? No. You are free to say yes or no, or to change your mind after you sign this form. At your request, the CoVPN will destroy all extra samples that it has. Your decision will not affect your being in this study.

Where are the samples stored? Extra samples are stored in a secure central place called a repository. Your samples will be stored in the CoVPN repository in the United States.

How long will the samples be stored? There is no limit on how long your extra samples will be stored. *[Site: Revise the previous sentence to insert limits if your regulatory authority imposes them.]*

Will I be paid for the use of my samples? No. Researchers may make scientific discoveries or products using your samples. If this happens, there is no plan to share any money with you.

Will I benefit from allowing my samples to be used in other studies? Probably not. Results from these other studies are not given to you, this clinic, or your doctor. They may help other people in the future.

Will the CoVPN sell my samples and information? No, but the CoVPN may share your samples with other researchers. Once the CoVPN shares your samples and information, it may not be able to get them back.

What information is shared with researchers? The samples and information will be labeled with a code number which will not be removed. The key to the code will stay at this clinic. However, some information that the CoVPN shares may be personal, such as your race, ethnicity, sex, and health information from the study.

What kind of studies might be done with my extra samples and information? The studies will be related to vaccines, the immune system, coronavirus, and other diseases.

Researchers may also do genetic testing on your samples.

If you agree, researchers may compare all of your genes (your genome) to the genomes of many other people. Researchers look for common patterns of genes to help them understand diseases. The researchers may put the information into a protected database so that other researchers can access it. Your name and other personal information will not be included.

Usually, no one could connect your genome to you as a person. There are rules against this. It's also really difficult to do. But there is a risk that someone could combine information from your genome and other public information about you and identify you. If others found out, it could lead to discrimination or other problems. The risk of this happening is very small.

Who will have access to my information in studies using my extra samples?

People who may see your information are:

- Researchers who use your extra samples and information for other research
- Government agencies that fund or monitor the research using your extra samples and information
- The researcher's Institutional Review Board or Ethics Committee
- Any regulatory agency that reviews research studies
- The people who work with the researcher

All of these people will do their best to protect your information. If they publish their research, they will not use your name or identify you personally.

4. **We will compensate you for your participation.**

Site: Insert your compensation plan here, e.g. money for number of swabs returned. Remember to include any costs to participants.

5. **We will do our best to protect your private information.**

Site: Check HIPAA authorization for conflicts with this section.

Your study records and samples will be kept in a secure location. We will label all of your samples and most of your records with a code number, not your name or other personal information. We will not share your name with anyone who does not need to know it.

Your records may also be reviewed by groups who watch over this study. These groups include:

- Study monitors
- The CoVPN, people who work for it, and companies that help it with this study
- Some government agencies:
  - The US National Institutes of Health

  - The US Office for Human Research Protections
  - Any regulatory agency that reviews research studies

- Some committees that make sure we protect your rights and keep you safe:
  - The Independent Data Monitoring Committee
  - Advarra Institutional Review Board (IRB)
  - *Sites may but are not required to include:* [Insert name of local IRB/EC]
  - *Sites include:* [Insert name of local IBC]

All reviewers will take steps to keep your records private.

Sites: Include the following boxed text. You can remove the box around the text.

We have a Certificate of Confidentiality from the US government to help protect your privacy. With the certificate, we do not have to release information about you to someone who is not connected to the study, such as the courts or police. Sometimes we can’t use the certificate. Since the US National Institutes of Health funds this research, we cannot withhold information from it.

We will not share your name or information that can identify you with the CoVPN. The CoVPN may share information from this study with other researchers. Researchers may publish the results of this study.

Sites: The text below may not be deleted or changed per FDA requirement. You can remove the box.

A description of this clinical trial will be available on http://www.ClinicalTrials.gov, as required by U.S. Law. This website will not include information that can identify you. At most, the website will include a summary of the results. You can search this website at any time.

6. **We may take you out of the study at any time.**

We may take you out of the study if:

- you do not follow instructions,
- we think that staying in the study might harm you,
- the study is stopped for any reason.

Risks

7. **There are some risks to being in this study.**

   This section describes the risks we know about. There may also be unknown risks, even serious ones. We will tell you if we learn anything new that may affect your willingness to stay in the study.

   Risks of blood draws:

   In this study, we will either take your blood or ask you to self-collect your blood with a small device. Taking blood can cause bruising, pain, fainting, soreness, redness, and itching.

   Risks of swabbing your nose:

   The feeling of having a small, soft-tipped swab inserted into your nostril and twirled around may be a little uncomfortable, but it should not be painful. There is a small chance there could be some bleeding, but this is unlikely.

   Risks to your personal information:

   We will take several steps to protect your personal information. Although the risk is very low, it is possible that your personal information could be given to someone who should not have it. If that happened, you could have stress or anxiety.

Benefits

8. **This study may not benefit you.**

   This study may help researchers find out if people who got the vaccine and later got infected with SARS-CoV-2 are less likely to give it to people who they have been in close contact with. If the vaccine later becomes approved and sold, there are no plans to share any money with you.

   If you decide not to join this study, you could wait to get the Moderna COVID-19 vaccine or another COVID-19 vaccine in your community. You can also continue to follow local guidelines for SARS-CoV-2 infection. You do not have to join this research study if you don't want to.

Leaving the study

9. **Tell us if you decide to leave the study.**

   You are free to leave the study at any time and for any reason. If you want to leave, you will need to tell us. Your legal rights will not be affected.

Previously collected information about you will remain in the study records and will be included in the analysis of results. Your information can not be removed from the study records.

We may ask you to give a final blood sample or nasal swab.

We believe these steps are important to protecting your health, but it is up to you whether to complete them.

Injuries

Sites: Approval from CoVPN Regulatory Affairs (at CoVPN.core.reg@fredhutch.org) is needed for any change (other than those that the instructions specifically request or those previously approved by CoVPN Regulatory Affairs) to the boxed text. You can remove the box around the text.

10. If you have a life-threatening medical emergency, call 911 right away or seek help immediately.

We do not expect you to get sick or injured as a result of being in this study. If you get COVID-19 while you are in the study, please let us know. *(Sites: adjust the following 2 sentences if applicable to the care available at your site)* We will tell you about the care that we can give here. For the care that we cannot provide, we will explain how we will help you get care elsewhere. You or your health insurance will have to pay for the care you get elsewhere.

Some injuries are not physical. For example, you might be harmed emotionally by being in this study. Or you might lose wages because you cannot go to work. However, there are no funds to pay for these kinds of injuries, even if they are study related.

You always have the right to use the court system if you are not satisfied.

Questions

11. **Whom to contact about this study**

During the study, if you experience any medical problems, suffer a research-related injury, or have questions, concerns, or complaints about the study, please contact the study doctor at the telephone number listed on the first page of this consent document.

An institutional review board (IRB) is an independent committee established to help protect the rights of research subjects.

If you have any questions about your rights as a research subject, and/or concerns or complaints regarding this research study, contact:

by mail:

Study Subject Adviser
Advarra IRB
6940 Columbia Gateway Drive, Suite 110
Columbia, MD 21046

or call **toll free**: 877-992-4724
or by **email**: adviser@advarra.com

Please reference the following number when contacting the Study Subject Adviser: [Advarra to insert number]

Sites may include, but are not required to: You may also contact the [name of local IRB/EC] at [insert contact information].

Your permissions and signature

12. In Section 3, we told you above about possible other uses of your extra samples and information outside this study. Please choose only one of the options below and write your initials in the box next to it. Whatever you choose, the CoVPN keeps track of your decision about how your samples and information can be used. You can change your mind after signing this form.

☐ I allow my extra samples and information to be used for other studies related to vaccines, the immune system, coronavirus, and other diseases. This may include genetic testing.

OR

☐ I agree to the option above *and* also to allow my extra samples and information to be used in studies that look at my whole genome.

OR

☐ I do not allow my extra samples to be used in any other studies. This includes not allowing genetic testing, or studies that look at my whole genome.

13. **If you agree to join this study, you will need to sign below. Before you sign, make sure of the following:**

- You have read this consent form.
- You feel that you understand what the study is about and what will happen to you if you join. You understand what the possible risks and benefits are.
- You agree to join this study.

You will not be giving up any of your rights by signing this consent form.

Participant's Name (Print)	Participant's Signature	Date
Clinic Staff Conducting Consent Discussion (Print)	Clinic Staff Signature	Date

Appendix D Procedures for Main Cohort, Immediate Vaccination Group

Visit:	01[1]	02[2]	03	04	05	06
Day:		D1	D29	D57	D113	D141
Week:		W0	W4	W8	W16	W20
Month:		M0	M1	M2	M4	M5
Procedure	Scr.	VAC1	VAC2			
**Study procedures**						
Screening consent (if used)	✓	–	–	–	–	–
Assessment of understanding	✓	–	–	–	–	–
Protocol consent (eConsent)	✓	–	–	–	–	–
Medical history questionnaire[3]	✓	✓	–	–	–	–
SARS-CoV-2 infection risk assessment[3]	✓	Weekly				
COVID-19 symptom surveillance[3]	–	Weekly starts at week 2, not during week 5				
Confirm eligibility	✓	–	–	–	–	–
Obtain demographics[3]	✓	–	–	–	–	–
Randomize	–	✓	–	–	–	–
SARS-CoV-2 screening at the University[3]	–	approximately 2x per week				
End of study questionnaire[3]	–	–	–	–	✓	–
**Research Samples[4]**						
Serum for Immunogenicity Assays and Storage	–	✓	–	✓	–	–
Serum for SARS-CoV-2 clinical serology[5]	–	✓[6]	–	✓[6]	✓	–
Nasal swab	–	Daily[7]				–
**Vaccination procedures**						
COVID-19 symptom check[8]	–	✓	✓	–	–	–
Vaccination	–	✓	✓	–	–	–

Greyed out visit column does not apply to this group.
1 Screening may occur over the course of several contacts/visits up to and including day 1, prior to vaccination.
2 Blood draws required at vaccination visits must be performed prior to vaccination.
3 Will be captured via eDiary, please see Section 9.15.
4 From screening to the final study visit, the total blood volume to be collected from each participant will be approximately 40 mL (maximum) with a maximum of approximately 17 mL collected in a single visit.
5 For blood self-collected by a participant the maximum volume will be less than 1 mL in a single collection.
6 SARS-CoV-2 clinical serology testing may be performed on leftover blood from samples collected for immunogenicity assays. No separate blood sample is needed. If a Main cohort participant is also in the Prospective Close Contact (PCC) cohort related to an index case or in the Case-ascertained Close Contact (CACC) cohort, one additional blood collection (self-collection) will be performed (see table below).
7 Daily swabs will be collected from vaccination through D113. Swabs will be returned via drop-off location(s) specified by the clinic or through the mail.
8 COVID-19 Symptom Check to be performed within 24 hours prior to vaccination.

The following **additional** procedures will occur for Main cohort, immediate vaccination participants, who fall under any of the following categories:

- tested positive for SARS-CoV-2 from enrollment through Month 4 (see Section 9.7)
- enrolled also in the PCC cohort and are related to an index case (see Section 9.8)
- enrolled also in the CACC cohort (see Section 9.9)

Day relative to notification of infection:	D1-14
**Study procedures**	
Symptom tracking via eDiary	daily
**Research Samples**	
Serum for SARS-CoV-2 clinical serology[1]	✓[2]

[1] For blood self-collected by a participant the maximum volume will be less than 1 mL in a single collection.

[2] Sample for serology will be collected as soon as possible following report of positive SARS-CoV-2 test.

In addition, we may ask participants to sign a release to allow us to review their COVID-19 related medical records.

Appendix E Procedures for Main Cohort, Delayed Vaccination Group

Visit:	**01**[1]	**02**	**03**	**04**	**05**	**06**
Day:		D1	D29	D57	D113	D141
Week:		W0	W4	W8	W16	W20
Month:		M0	M1	M2	M4	M5
Procedure	Scr.				VAC1	VAC2
**Study procedures**						
Screening consent (if used)	✓	–		–	–	–
Assessment of understanding	✓	–		–	–	–
Protocol consent (eConsent)	✓	–		–	–	–
Medical history questionnaire[2]	✓	✓		–	–	–
SARS-CoV-2 infection risk assessment[2]	✓	Weekly				–
COVID-19 symptom surveillance[2]	–	Weekly				–
Confirm eligibility	✓	–		–	–	–
Obtain demographics[2]	✓	–		–	–	–
Randomize	–	✓		–	–	–
SARS-CoV-2 screening at the University[2]	–	approximately 2x per week				–
End of study questionnaire[2]	–	–		–	–	✓
**Research Samples**						
Serum for SARS-CoV-2 clinical serology[3]	–	✓		✓	✓	–
Nasal swab	–	Daily[4]				–
**Vaccination procedures**						
COVID-19 symptom check[5]	–	–		–	✓	✓
Vaccination	–	–		–	✓	✓

Greyed out visit does not apply to this group.

1 Screening may occur over the course of several contacts/visits up to and including day 1, prior to randomization.
2 Will be captured via eDiary, please see Section 9.15.
3 From screening to the final study visit, the total blood volume to be collected from each participant will be approximately 15 mL (maximum) with a maximum of approximately 5 mL collected in a single visit. For blood self-collected by a participant the maximum volume will be less than 1 mL in a single collection. If a Main cohort participant is also in the Prospective Close Contact (PCC) cohort related to an index case or in the Case-ascertained Close Contact (CACC) cohort, one additional blood collection (self-collection) will be performed (see table below).
4 Daily swabs will be collected from D1 through D113. Swabs will be returned via drop-off location(s) specified by the clinic or through the mail.
5 COVID-19 Symptom Check to be performed within 24 hours prior to vaccination.

The following **additional** procedures will occur for Main cohort, delayed vaccination participants, who fall under any of the following categories:

- tested positive for SARS-CoV-2 from enrollment through Month 4 (see Section 9.7)
- enrolled also in the PCC cohort and are related to an index case (see Section 9.8)
- enrolled also in the CACC cohort (see Section 9.9)

Day relative to notification of infection:	D1-14
**Study procedures**	
Symptom tracking via eDiary	daily
**Research Samples**	
Serum for SARS-CoV-2 clinical serology[1]	✓[2]

[1]For blood self-collected by a participant the maximum volume will be less than 1 mL in a single collection.
[2] Sample for serology will be collected as soon as possible following report of positive SARS-CoV-2 test

In addition, we may ask participants to sign a release to allow us to review their COVID-19 related medical records.

Appendix F Procedures for Prospective Close Contact (PCC) cohort

Visit:	**100**	**101**
Day:	D1	D113
Month:	M0	M4
	Enrollment	Follow-up
**Study procedures**		
Protocol consent (eConsent)	✓	–
Obtain demographics[1]	✓	–
SARS-CoV-2 screening at the University[1]	approximately 2x per week	
End of study questionnaire[1]	–	✓

[1]Will be captured via eDiary, please see Section 9.15.

If a PCC participant (or their Main study contact) tests positive for SARS-CoV-2, the following **additional** procedures will occur:

Day relative to notification of infection:	D1	D29
**Study procedures**		
Report of exposure[1]	✓	
Symptom tracking[1]	Daily for 14 days	–
**Research Samples**		
Serum for SARS-CoV-2 clinical serology[2]	✓	✓
Nasal swab[3]	Daily for 14 days	–

[1] Will be captured via eDiary , please see Section 9.15.
[2]For blood self-collected by a participant the maximum volume will be less than 1 mL in a single collection. Sample for serology will be collected as soon as possible following report of positive SARS-CoV-2 test.
[3]Swabs will be returned via drop-off location(s) specified by the clinic or through the mail. Nasal swabs will be collected as soon as possible following report of positive SARS-CoV-2 test.

In addition, we may ask participants to sign a release to allow us to review their COVID-19 related medical records.

Appendix G Procedures for Case-Ascertained Close Contact Cohort

Visit:	**200**	**201**	**202**
Day:	D1	D1-D3	D29
Month:	M0	M0	M1
	Enrollment	Initial specimen collection	Follow-up
**Study procedures**			
Protocol consent (eConsent)	✓	–	–
Collection of demographic information[1]	✓	–	–
Report of exposure[1]	✓	–	–
Symptom tracking[1]		Daily for 14 days	–
SARS-CoV-2 screening at the University if applicable[1]		approximately 2x per week	
End of study questionnaire[1]	–	–	✓
**Research Samples**			
Serum for SARS-CoV-2 clinical serology[2]		✓	✓
Nasal swab[3]		Daily for 14 days	–

[1]Will be captured via eDiary, please see Section 9.15.
[2] For blood self-collected by a participant the maximum volume will be less than 1 mL in a single collection. Sample for serology will be collected as soon as possible following report of positive SARS-CoV-2 test (ie, as early as Day 1).
[3]Swabs will be returned via drop-off location(s) specified by the clinic or through the mail. Nasal swabs will be collected as soon as possible following report of positive SARS-CoV-2 test (ie, as early as Day 1).

In addition, we may ask participants to sign a release to allow us to review their COVID-19 related medical records.

Appendix H Visit windows

Main Cohort, Immediate Vaccination, Visit Windows

Visit Number	Visit Type	Lower Allowable Window	Lower Target Day	Target Day[1]	Upper Target Day	Upper Allowable Window
01.0	Screening	-56	-		-	0
02.0	**Enrollment/Randomization**	-	-	1	-	-
03.0	**Vaccination 2**	-	-3	29[2]	+7	+14
04.0	Month 2 Follow-up	-	-7	57	+7	+14
05.0	Month 4 Follow-up	-13	-7	113	+14	+56

1. Target dates are relative to Visit 2.0 (Enrollment/Randomization), except for cases described in footnote 2.
2. In the event that Vaccination 1 occurs on a different day than Visit 2.0 (Enrollment/Randomization), Visit 3.0 target dates are relative to date of Vaccination 1.

Main Cohort, Delayed Vaccination, Visit Windows

Visit Number	Visit Type	Lower Allowable Window	Lower Target Day	Target Day[1]	Upper Target Day	Upper Allowable Window
01.0	Screening	-56	-		-	0
02.0	**Enrollment/Randomization**	-	-	1	-	-
04.0	Month 2 Follow-up	-	-7	57	+7	+14
05.0	**Vaccination 1**	-13	-7	113	+14	+28
06.0	**Vaccination 2**[1]	-	-3	141	+7	+14

1. Target dates are relative to Visit 2.0 (Enrollment/Randomization), except visit 06.0, which is relative to visit 05.0.

Case-Ascertained Close Contact (CACC) Cohort and applicable Prospective Close Contact (PCC) participants[1],Visit Windows

Visit Number	Visit Type	Lower Allowable Window	Lower Target Day	Target Day[2]	Upper Target Day	Upper Allowable Window
200.0	**Enrollment**	-	-	1	-	-
201.0	Initial specimen collection		-2	3	+1	
202.0	Day 29 Follow-up	-	-7	29	+14	+28

1. Applicable to PCC participants when they (or their Main study contact) tests positive for SARS-CoV-2.
2. Target dates are relative to Visit 200.0 (Enrollment).

Appendix I Protocol Signature Page

A randomized controlled study to assess SARS CoV-2 infection, viral shedding, and subsequent potential transmission in university students immunized with Moderna COVID-19 Vaccine

I will conduct the study in accordance with the provisions of this protocol and all applicable protocol-related documents. I agree to conduct this study in compliance with United States (U.S.) Health and Human Service regulations (45 CFR 46); applicable U.S. Food and Drug Administration regulations; standards of the International Council for Harmonisation of Technical Requirements for Pharmaceuticals for Human Use (ICH) Guideline for Good Clinical Practice (E6) (R2); Institutional Review Board/Ethics Committee determinations; all applicable in-country, state, and local laws and regulations; and other applicable requirements (eg, U.S. National Institutes of Health, Division of AIDS) and institutional policies.

Investigator of Record Name (print)	Investigator of Record Signature	Date

Protocol Number: CoVPN 3006

Protocol Version: Version 2.0

Protocol Date: February 26, 2021

COVID-19
Prevention Network

PROTOCOL

CoVPN 3006

A randomized controlled study to assess SARS CoV-2 infection, viral shedding, and subsequent potential transmission in university students immunized with Moderna COVID-19 Vaccine

DOCUMENT ID 38822

Non-IND Study

CLINICAL TRIAL SPONSORED BY

National Institute of Allergy and Infectious Diseases (NIAID)
National Institutes of Health (NIH)
Department of Health and Human Services (DHHS)
Bethesda, Maryland, USA

April 12, 2021
Final
CoVPN 3006
Version 3.0

Contents

1 Overview 5
1.1 Protocol Team 8
2 Background 10
2.1 Rationale for trial concept 10
2.2 Measures of vaccine efficacy 11
2.3 Importance of understanding vaccine effects on infectiousness and transmissibility 12
2.4 Limitations of the current Phase 3 trials for addressing unanswered questions 15
2.5 Burden of SARS-CoV-2 infection and COVID-19 disease among students 17
2.6 Age and Asymptomatic Infection 18
2.7 Evidence for viral load as a predictor of infectiousness 18
2.8 Evidence for viral load as a marker of COVID-19 disease severity and clinical course 20
2.9 Methods for capturing transmission 20
2.10 Moderna COVID-19 Vaccine 21
2.11 Trial design rationale 22
3 Objectives and endpoints 24
4 Ethical considerations 28
5 IRB/EC review considerations 30
5.1 Minimized risks to participants 30
5.2 Reasonable risk/benefit balance 30
5.3 Equitable participant selection 30
5.4 Appropriate informed consent 31
5.5 Adequate safety monitoring 31
5.6 Protect privacy/confidentiality 31
6 Statistical considerations 33
6.1 Accrual and sample size calculations 35
6.2 Role of the Independent Data Monitoring Committee (IDMC) 40
6.3 Randomization 40
6.4 Statistical analyses 40
7 Selection and withdrawal of participants 49
7.1 Inclusion criteria for Main Cohort 49
7.2 Exclusion criteria for Main Cohort 50
7.3 Inclusion criteria for Prospective Close Contact (PCC) cohort 51
7.4 Inclusion criteria for Case-ascertained Close Contact (CACC) cohort 51
8 Study product 52
8.1 Vaccine regimen 52
8.2 Storage, handling, preparation, and administration 52
8.3 Acquisition of Moderna COVID-19 Vaccine 52
9 Clinical procedures 53
9.1 Informed consent 53

9.2 Pre-enrollment procedures for Main Cohort, Immediate and Delayed Vaccination Groups 54
9.3 Enrollment and vaccination visits for Main Cohort, Immediate Vaccination Group 55
9.4 Follow-up procedures for Main Cohort, Immediate Vaccination Group 55
9.5 Enrollment (visit 2) and visit 4 (Month 2) for Main Cohort, Delayed Vaccination Group 56
9.6 Vaccination visits for Main Cohort, Delayed Vaccination Group 56
9.7 Procedures for Main Cohort participant diagnosed with a SARS-CoV-2 infection during the study 57
9.8 Enrollment and follow-up procedures for Prospective Close Contact (PCC) cohort 57
9.9 Enrollment and Follow-up procedures for Case-Ascertained Close Contact (CACC) cohort 58
9.10 Visit windows and missed visits 59
9.11 Discontinuing vaccination for a participant who receives a COVID-19 Vaccine outside the study 59
9.12 Early termination visit 59
9.13 Pregnancy for Main Cohort 60
9.14 SARS-CoV-2 monitoring by schools for Main Cohort 60
9.15 COVID-19 symptom surveillance for Main Cohort 60
9.16 Use of electronic Diary (eDiary) for Clinical Outcomes Assessments 60

10 Laboratory 63
10.1 CRS laboratory procedures 63
10.2 Total blood volume 63
10.3 Endpoint assays 63
10.4 Lab assay portfolio 64
10.5 Exploratory studies 64
10.6 Specimen storage and other use of specimens 65
10.7 Biohazard containment 65

11 Safety Reporting 66
11.1 FDA/CDC Vaccine Adverse Event Reporting System (VAERS) 66
11.2 Reporting safety events to v-safe 67

12 Protocol conduct 68
12.1 Study termination 68
12.2 Emergency communication with study participants 69

13 Version history 70

14 Document references (other than literature citations) 74

15 Acronyms and abbreviations 76

16 Literature cited 78

Appendix A Sample informed consent form for Main cohort 83

Appendix B Sample informed consent form for Prospective Close Contact (PCC) cohort101

Appendix C Sample informed consent form for Case-Ascertained Close Contact (CACC) cohort 112
Appendix D Procedures for Main Cohort, Immediate Vaccination Group 123
Appendix E Procedures for Main Cohort, Delayed Vaccination Group 125
Appendix F Procedures for Prospective Close Contact (PCC) cohort...........................127
Appendix G Procedures for Case-Ascertained Close Contact Cohort............................128
Appendix H Visit windows...129
Appendix I Protocol Signature Page 130

1 Overview

Title

A randomized controlled study to assess SARS CoV-2 infection, viral shedding, and subsequent potential transmission in university students immunized with Moderna COVID-19 Vaccine

Primary objective(s)

Primary objective 1:

To evaluate the efficacy of Moderna COVID-19 Vaccine against SARS-CoV-2 infection (ie, to evaluate vaccine efficacy against infection or VE_S)

Primary objective 2:

To evaluate the effect of Moderna COVID-19 Vaccine on peak nasal viral load as a measure of infection and a proxy of infectiousness

Study product and route of administration

Moderna COVID-19 Vaccine: a lipid nanoparticle (LNP) dispersion of a messenger ribonucleic acid (mRNA) encoding the prefusion stabilized S protein of SARS-CoV-2 formulated in LNPs composed of 4 lipids (1 proprietary and 3 commercially available). The Moderna COVID-19 Vaccine, also known as mRNA-1273, is a suspension for intramuscular injection administered as a series of two doses (0.5 mL each) 1 month apart.

This vaccine has been issued an Emergency Use Authorization (EUA) by the US Food and Drug Administration (FDA) for active immunization to prevent COVID-19 in individuals 18 years and older.

Table 1-1 Schema for the Main Cohort

		Injection schedule in months			
Group	N	0	1	4	5
Immediate Vaccination	6000	Moderna COVID-19 Vaccine	Moderna COVID-19 Vaccine	-	-
Delayed Vaccination	6000	-	-	Moderna COVID-19 Vaccine	Moderna COVID-19 Vaccine
Total	12,000				

Participants

Main Cohort (main study participants)

Approximately 12,000 healthy student volunteers enrolled in post-secondary school classes, aged 18 through 26 years, who will be followed for 5 months after enrollment; 6,000 randomized to immediate vaccination, 6,000 randomized to delayed vaccination

Prospective Close Contact (PCC) Cohort

Up to approximately 24,000 volunteers who are in frequent close physical proximity with main study participants

Case-Ascertained Close Contact (CACC) Cohort

Approximately 3 contacts per incident case. Actual number dependent on incidence rate. Up to approximately 1500 volunteers aged 18 years and older who have been in close contact with a SARS-CoV-2 positive case from the Main Cohort

Design

Multicenter, randomized, controlled, open-label trial

Duration per participant

Up to 5 months per participant for the Main Cohort and for the Prospective Close Contact Cohort. 1 month per participant in Case-Ascertained Close Contact Cohort.

Estimated total study duration

7 months (includes enrollment and follow-up).

Core operations

CoVPN Vaccine leadership Group/Core Operations Center, Fred Hutchinson Cancer Research Center (Fred Hutch) (Seattle, Washington, USA)

PPD, Inc. (Wilmington, North Carolina, USA)

Statistical and Data Management Center (SDMC)

Statistical Center for HIV/AIDS Research and Prevention (SCHARP), Fred Hutch (Seattle, Washington, USA)

Laboratory Center (LC)

Endpoint assay laboratories

- University of Washington Virology Laboratory (UW-VL) (Seattle, Washington, USA)
- University of Washington Virology Specialty Laboratory (UW-VSL) (Seattle, Washington, USA)
- Pioneer Hi-Bred International, Inc., a member of the Corteva Agriscience group of Companies (Johnston, Iowa, USA)
- Duke University Medical Center (Durham, North Carolina, USA)
- CoreMedica Labs (Lee's Summit, Missouri, USA)

Study sites

University clinical research sites (CRSs) across the US and CoVPN CRSs in proximity to post-secondary educational institutions

Study monitoring

Independent Data Monitoring Committee (IDMC)

1.1 Protocol Team

Protocol leadership

Co-chairs

Kathryn Stephenson
Harvard University School of Medicine
Beth Israel Deaconess Medical Center
617-735-4556
kstephen@bidmc.harvard.edu

Audrey Pettifor
Department of Epidemiology
Gillings School of Global Public Health
University of North Carolina
919-966-7439
apettif@email.unc.edu

Jasmine Marcelin
Division of Infectious Diseases
University of Nebraska Medical Center
402-559-8650
jasmine.marcelin@unmc.edu

Protocol Lead Advisors

Larry Corey
CoVPN LOC, Fred Hutch
206-667-6770
lcorey@fredhutch.org

Myron Cohen
University of North Carolina
919-966-2537
myron_cohen@med.unc.edu

James Kublin
CoVPN LOC, Fred Hutch
206-667-1970
jkublin@fredhutch.org

Statisticians

Holly Janes
CoVPN SDMC, Fred Hutch
206-667-6353
hjanes@fredhutch.org

Elizabeth Brown
CoVPN SDMC, Fred Hutch
206-667-1731
erbrown@fredhutch.org

Laboratory lead

John Hural
CoVPN Laboratory Program
206-667-1683
jhural@fredhutch.org

Protocol Team leader

Nicole Grunenberg
CoVPN LOC, Fred Hutch
206-667-2043
ngrunenb@fredhutch.org

DAIDS Medical Officer

Catherine Yen
DAIDS, NIAID
240-292-4783
catherine.yen@nih.gov

Other contributors to the original protocol

Core medical monitor	Nicole Grunenberg<br>CoVPN LOC, Fred Hutch	*CoVPN Physician consultant*	Nzeera Ketter<br>CoVPN LOC, Fred Hutch
Laboratory consultants	Alexander Greninger<br>Robert Coombs<br>University of Washington Medical Center (UWMC)<br><br>Keith Jerome<br>Fred Hutch, UWMC	*Clinical safety specialists*	Megan Jones<br>Maija Anderson<br>CoVPN LOC, Fred Hutch
Laboratory center representatives	Nicole Espy<br>Lisa Sanders<br>Jen Hanke<br>CoVPN Laboratory Center, Fred Hutch	*Clinical trials manager*	Julie Hunt<br>CoVPN LOC, Fred Hutch
Regulatory affairs associate	Megan Brandon<br>CoVPN LOC, Fred Hutch	*Clinical data managers*	Melissa Cummings<br>Vicky Kim<br>CoVPN SDMC, Fred Hutch
Community Advisory Board (CAB) members	John Isaac<br>Boston University	*SDMC Associate Director of Operations*	Jessica Andriesen<br>CoVPN SDMC, Fred Hutch
Statistical research associates	Erika Rudnicki<br>Moni Neradilek<br>CoVPN SDMC, Fred Hutch	*SDMC Associate director of lab science*	April Randhawa<br>CoVPN SDMC, Fred Hutch
Community engagement unit representative	Francisco E Rentas<br>CoVPN LOC, Fred Hutch	*Data Management Director*	Kristine Donaty<br>CoVPN SDMC, Fred Hutch
Project coordinator	Kylie McCloskey<br>CoVPN LOC, Fred Hutch	*Protocol development managers*	Kajari Mondal<br>Meg Trahey<br>CoVPN LOC, Fred Hutch

2 Background

2.1 Rationale for trial concept

The United States has the most infections and deaths from SARS-CoV-2 (the virus that causes Coronavirus disease 2019 [COVID-19]) of any nation in the world (1). Fortunately, there is now hope that the large burden of COVID-19-related illness may lessen in 2021 due to the discovery that candidate COVID-19 vaccines, including the Moderna COVID-19 Vaccine, can prevent symptomatic COVID-19 and severe disease in adults (2, 3). Ongoing Phase 3 trials are not designed to estimate a vaccine's efficacy against all infections, with a critical gap being asymptomatic infections. The current trials are also not designed to evaluate vaccine effects on infectiousness as measured by viral shedding or on onward transmission of infection. For example, at an individual level, it is currently unknown if vaccinated individuals should continue to wear masks or social distance, or if they should be quarantined if exposed to COVID-19. At a policy level, it is unknown if vaccination should be required for certain settings, such as public transit including air travel, places of employment, or school. These questions are especially important given that SARS-CoV-2 is highly infectious compared to other respiratory illnesses such as the flu. Recent data also demonstrate that even individuals with asymptomatic COVID-19 can have high titers of nasal viral shedding and thus may be highly infectious. Furthermore, many individuals with asymptomatic infection develop low level and transient antibodies against SARS-CoV-2 (4), suggesting that trials relying on seroconversion as a marker of infection may miss infections.

While there are many populations at risk for SARS-CoV-2 infection, young people are particularly at risk of acquiring and transmitting SARS-CoV-2. Between August and September 2020, COVID-19 cases among young people aged 18-22 years increased 55% nationally (5) and during June-August 2020, young people aged 20-29 years had the highest incidence of disease in the US, accounting for >20% of all confirmed cases (6). Further, increases in SARS-CoV-2 infection among younger adults in the Southern US preceded increases among older adults by 4-15 days, which confirms the importance of younger adults spreading infection to older, more vulnerable populations (5). At the same time, individuals aged 18-29 account for a tiny percentage (0.5%) of all deaths due to COVID-19 and are very unlikely to develop severe disease (7). These reasons plus the congregate and social living situations of students and that many schools are regularly testing their student body make them an excellent population to examine. The proposed study is designed to evaluate comprehensively the vaccine efficacy profile of the Moderna COVID-19 Vaccine, including its effect on acquisition of infection including asymptomatic infection, on disease and severity of disease, on measures of infectiousness, and transmission of infection.

This study is a 2-arm randomized, open-label trial in students aged 18 through 26 years that will compare those who receive the Moderna COVID-19 Vaccine upon enrollment (immediate vaccination arm) to those who receive the vaccine 4 months later (delayed vaccination arm). **The study will capture all incident SARS-CoV-2 infections among study participants in real time, by establishing real-time testing for SARS-CoV-2 on study and augmenting with all available data from school testing or community testing.**

Study participants will be asked to collect daily nasal swabs, to enable ascertainment of the timing of acquisition and to capture the full viral load trajectory for incident infections. To capture secondary transmission events, very close contacts of study participants will be enrolled who may undergo school or other community testing for incident infection over the duration of the study ('prospective contacts'); in addition, diagnosis of infection among a study participant will trigger recruitment of additional participant contacts ('case-ascertained close contacts'). When a study participant tests positive based on testing of study-collected nasal swabs, school testing, or community testing, both prospective contacts and case-ascertained close contacts will be asked to collect daily nasal swabs for 14 days to detect incident infection and also will have blood collected for serology. The study will take place over the course of the 2021 spring and summer school terms. At the end of the trial, all participants in the delayed arm will be offered vaccine. The goal of the study is to report results prior to fall 2021 to inform school policy prior to when the new year begins for most educational institutions.

2.2 Measures of vaccine efficacy

Ongoing Phase 3 COVID-19 vaccine trials are designed with the primary objective to evaluate vaccine efficacy against COVID-19 disease, written VE_D, and defined as the multiplicative reduction in risk of disease in the vaccine vs. placebo groups. COVID-19 disease is defined by a protocol-specified set of signs and symptoms of disease coincident with PCR-confirmed SARS-CoV-2 infection. While the reduction in risk of disease is arguably the most relevant vaccine effect to the individual participant, understanding the population effect of a vaccine requires considering the other effects of the vaccine (see Table 2-1) (8). The most fundamental effect of a vaccine is to reduce acquisition of infection, sometimes referred to as 'vaccine efficacy against susceptibility', written VE_S, and measured by the multiplicative reduction in PCR-confirmed infection in the vaccine vs. placebo group. A vaccine that reduces acquisition of infection will provide benefit to the individual (i.e. *direct* effects), as well as benefit to the population by reducing the number of infected and therefore potentially infectious individuals who may expose and infect unvaccinated individuals in the population (i.e. *indirect* effects). Modeling studies suggest that COVID-19 vaccines that reduce acquisition are predicted to have considerably greater population impact, not only reducing disease, hospitalizations, and mortality, but reducing transmission of infection and thereby magnifying their effects (9, 10).

An additional potential indirect effect of a vaccine is its effect on reducing secondary transmission of infection, written VE_I and defined as the multiplicative reduction in the rate of secondary transmission among vaccine vs. placebo individuals who become SARS-CoV-2 infected. A vaccine might reduce transmission through reducing the magnitude and/or duration of viral shedding, by reducing symptoms associated with transmission such as coughing, or by selectively blocking infection with less transmissible viral strains. Evaluating the effect of a COVID-19 vaccine on transmissibility is critical because a vaccine with high efficacy against COVID-19 disease but low efficacy against SARS-CoV-2 infection would necessarily yield a net increase in the number of asymptomatic infections. If individuals who become asymptomatically infected despite having received the vaccine are still infectious,

and given that asymptomatic individuals are less likely to be diagnosed and self-isolate, such a vaccine has the potential to increase transmission and prolong the pandemic (2, 3).

As we prepare this study, we recognize there are other approaches to measuring population level benefit (eg, vaccine effect on infection and transmissibility). These approaches include: observational studies, such as those being conducted in Israel to examine population benefit of the vaccine and those planned by the CDC to examine transmission from vaccinated individuals to household contacts in longitudinal cohorts; and ancillary studies with frequent swabbing on a subset of participants in Phase 3 studies. However, none of these studies will allow for the rigorous capture, through daily nasal swabbing, of the early dynamics of viral replication and the capture of transmission events during the acute period of infection in a randomized trial.

All of these vaccine salutatory effects may depend on a variety of individual characteristics such as age, prior infection with SARS-CoV-2; on virological characteristics; and in the case of VE_I which conditions on infection, may depend on clinical characteristics such as symptomatology.

As will be discussed below in detail, the ongoing Phase 3 trials evaluate VE_D and partially identify VE_S, but do not allow direct estimation of VE_I. In contrast, the proposed study will collect data to enable evaluation of all three vaccine efficacy parameters in the student population. Importantly, the use of an open-label delayed vaccination arm as opposed to a blinded placebo arm, implies that the various effects evaluated will capture not only biological vaccine effects, but also potential behavioral effects associated with vaccination, including potential behavioral disinhibition. Behavioral effects most plausibly influence exposure and therefore infection, but knowledge of vaccine receipt is viewed as less likely to influence disease, viral load, and secondary transmission.

Table 2-1 Population measures of vaccine efficacy that will be evaluated using the proposed study, where a delayed vaccination arm will be used instead of placebo

Efficacy measure	Definition
Vaccine efficacy against SARS-CoV-2 infection (VE_S)	The multiplicative reduction in SARS-CoV-2 infection as measured by PCR of nasal swabs, vaccine vs. placebo
Vaccine efficacy against COVID-19 disease (VE_D)	The multiplicative reduction in symptomatic COVID-19*, vaccine vs. placebo
Vaccine efficacy against viral replication (VE_{VL})	The reduction in SARS-CoV-2 viral burden as measured by PCR of nasal swabs, vaccine vs. placebo
Vaccine efficacy against transmission (VE_I)	The multiplicative reduction in rate of SARS-CoV-2 transmission from infected individuals on the vaccine arm relative to the placebo arm

*Defined by a protocol-specific set of signs and symptoms defined in Section 3.

2.3 Importance of understanding vaccine effects on infectiousness and transmissibility

The most effective way for a vaccine to prevent onward transmission of infection is to prevent acquisition of infection. Vaccine efficacy against infection, VE_s, is a measure of this effect (8). However, a vaccine that does not completely block acquisition of infection may

still reduce onward transmission among individuals who become infected despite having received the vaccine, as measured by VE_I (8, 11). As discussed above, understanding the effect of a COVID-19 vaccine on transmission is critical because a vaccine with high efficacy against COVID-19 disease but low efficacy against SARS-CoV-2 infection would necessarily yield a net increase in the number of asymptomatic infections. If individuals who become asymptomatically infected despite having received the vaccine are still infectious, and given that asymptomatic individuals are less likely to be diagnosed and self-isolate, such a vaccine has the potential to increase transmission and prolong the pandemic (2, 3).

If a COVID-19 vaccine did reduce infectiousness, mathematical modeling suggests that much greater population impact could be achieved. Deterministic compartmental models fit to data from King County, Washington and envisioning vaccine rollout starting December 1, 2020 to 18% of the adult population proportionally across age groups, suggest that while a vaccine that reduces COVID-19 disease by 90% and SARS-CoV-2 infection by 10% reduces total infections in 2021 by 18.1% and deaths by 27.5%, if the vaccine additionally reduces infectiousness by 50% the impact on infections would more than double (to 49.3% reduction) and deaths would be cut in half (to 50.9% reduction) (Swan and Dimitrov et al, in preparation; Figure 2-1).

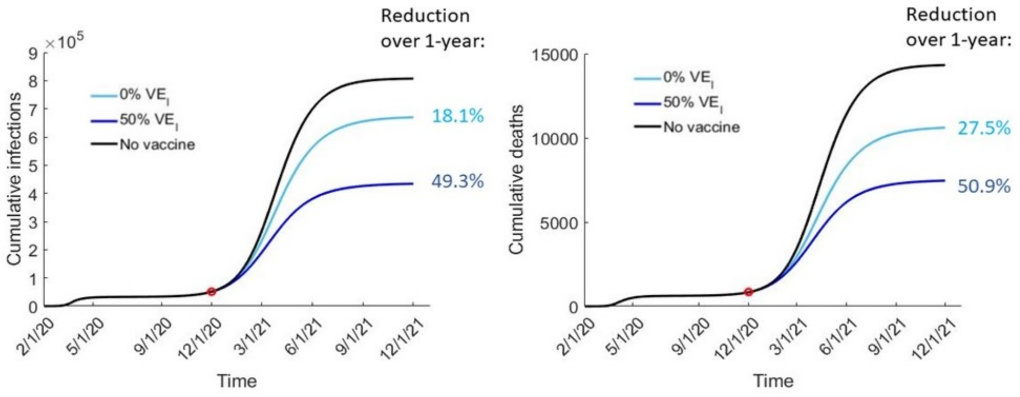

Figure 2-1 COVID-19 vaccine that reduces infectiousness by 50% has greater impact on infections and deaths. Deterministic compartmental model calibrated to King County, WA stratified by age, SARS-CoV-2 infection status, COVID-19 treatment status and vaccination. 800,000 doses vaccine (for 18% of population) starting Dec 1, 2020, allocated proportionally across age groups. Absent vaccination, 20% of infections are asymptomatic; asymptomatic infections are 28% less infectious than symptomatic infections, but more transmissible due to lower rates of diagnosis and quarantine. Both vaccines reduce COVID-19 disease by 90% and SARS-CoV-2 infection by 10%. While one vaccine (light blue) does not reduce infectiousness among individuals who become infected, the other vaccine (dark blue) reduces infectiousness by 50%. As a result, it achieves a much greater reduction in both infections and deaths. (Swan, Dimitrov et al, in preparation).

In the context of limited vaccine supply, mathematical modeling also suggests that optimal allocation of vaccine depends on how well a vaccine reduces infectiousness. Take, for instance, a deterministic compartmental model calibrated to Washington State that uses the following assumptions: that, absent vaccination, 40% of infections are asymptomatic, asymptomatic infections confer equal immunity and are equally infectious, and that there is sufficient vaccine supply for 30% of the population. For a vaccine that reduces COVID-19 disease by 90% but has no effect on infection or infectiousness, the limited supply of vaccine

should be allocated to older adults who have a high burden of disease in order to minimize deaths and peak hospitalizations (Figure 2-2). However, if the vaccine also reduces infectiousness by 60%, minimizing peak hospitalizations is achieved by allocating limited vaccine to the young, while minimizing deaths still requires vaccinating older adults. These results again highlight the critical importance of understanding vaccine effects on acquisition and infectiousness in order to make policy decisions.

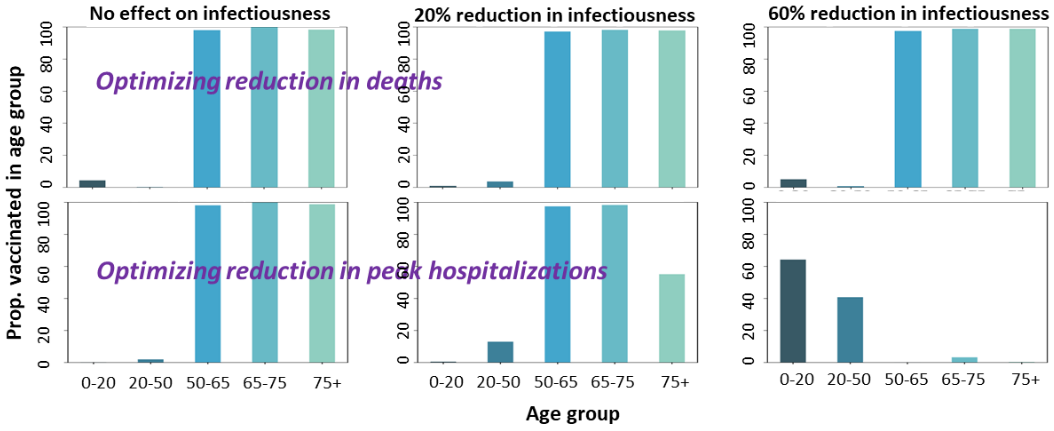

Figure 2-2 Deterministic compartmental model calibrated to Washington State (7.6M people), reflecting US demographics. Model assumes 4.56M doses vaccine (for 30% of population) assuming 10% of the population is previously infected and immune and there are 1000 current infections. Absent vaccination, 40% of infections are asymptomatic; asymptomatic infections confer equal immunity (for at least 6 mo. duration) and are equally infectious. (Matrajt et al, in preparation)

Non-human primate (NHP) data also suggests that elimination of viral shedding by a COVID-19 vaccine is not guaranteed. Table 2-2 shows the comparison of NHP data between candidate COVID-19 vaccines in current Phase 3 trials. While most of the vaccines were shown to prevent viremia within the lower respiratory tract (bronchoalveolar lavage fluid), and thus improve markers of disease severity, three out of the four platforms tested did not eradicate nasal viral shedding in all cases.

Table 2-2 Comparison of NHP data between Janssen Ad26, Moderna RNA, AstraZeneca ChimpAdOx1 and Novavax COVID-19 vaccines

	Ad26<br>One dose<br>1 x10^{11}	mRNA<br>(4 weeks post-dose 2)	AstraZeneca/Oxford<br>2.5x10^{10}<br>0, 4 weeks	Novavax<br>2 doses (0,21)<br>5 mcg, 50 mcg
**Pseudovirus neutralization (GMT)**	408	1,862	5 – 40<br>boost 10 - 160	No
**Live virus assay**	113	3,481		16,026
**Spike protein**	15,000<br>(not shown)	36,186 (AUC)		335,017
**Lung BAL protection**	Complete	Complete at day 7	75% both prime and boost	Complete
**Nasal protection**	5/6 (83%)	7/8	0%	Complete (12/12)
**T-cell immune response**	Some CD8	No CD8+ T cells	Not reported	CD4 and CD8

Finally, not understanding relative infectivity is a huge gap in translating vaccine use to public health messaging. The demonstration of reduced infectivity may reduce vaccine hesitancy. Public health, medical personnel and organizations may embrace vaccination, as well as users, if the vaccine both provides individual as well as family/community benefit. For individuals, knowing that a vaccine reduces infectivity to household and personal contacts is often a prime motivator for vaccination. Data from this trial can facilitate this messaging as well as help decision-making among individuals about interactions with household members and attending work and school.

2.4 Limitations of the current Phase 3 trials for addressing unanswered questions

Currently, none of the current Phase 3 vaccine efficacy trials will enable robust estimation of vaccine effects on infectiousness. The primary objectives for all trials are to evaluate vaccine efficacy (VE) against COVID-19 disease. Secondary objectives include evaluating VE against seroconversion using infrequent serology (every 1-6 months over 1 year, with more frequent sampling over the first 3 months), but this will not fully capture all infections including those that are asymptomatic. Estimates based on acute infection cohorts (12) suggest that 12-50% of asymptomatic infections might be missed using this sampling, leading to an unknown magnitude and direction of bias in the estimate of VE_S. Data from Long et al. found that 40% of individuals with asymptomatic RT-PCR confirmed SARS-CoV-2 infections were IgG negative at 8 weeks after the acute phase of infection (Figure 2-3) (4). Further, it has been observed that asymptomatic cases have lower IgG titers across all

antigens (including NP) (Figure 2-4) (13). In current trials, infectiousness is not captured robustly among those who become infected; viral load (VL) is only measured for those who are symptomatic and only after the onset of symptoms. Data from the Moderna trial where nasal swabs were collected at the time of vaccine administration on days 0 and 28 among all participants suggest that the vaccine may reduce the level of nasal carriage of virus (14). However, one time point does not robustly capture the full viral dynamics of infection, in particular, asymptomatic infections. The data are suggestive that the vaccine may reduce viral carriage (15). The level of viral shedding prior to the onset of symptoms, when most transmission occurs (16), will not be captured for any infected participants. Importantly, no data in current trials are collected on onward transmission of infection and therefore VE_I cannot be evaluated directly.

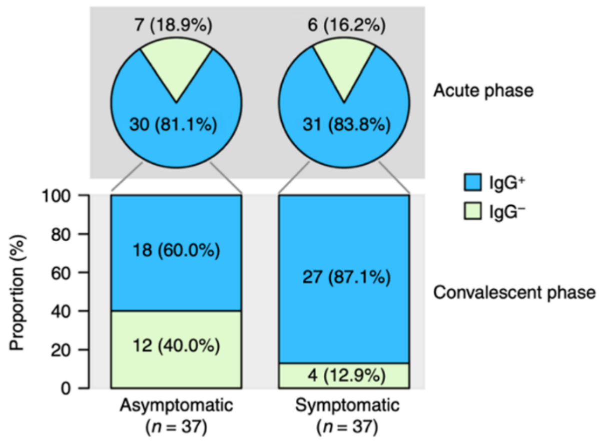

Figure 2-3 40% of asymptomatic individuals were IgG negative by 8 weeks post-acute infection (Long et al.(4).

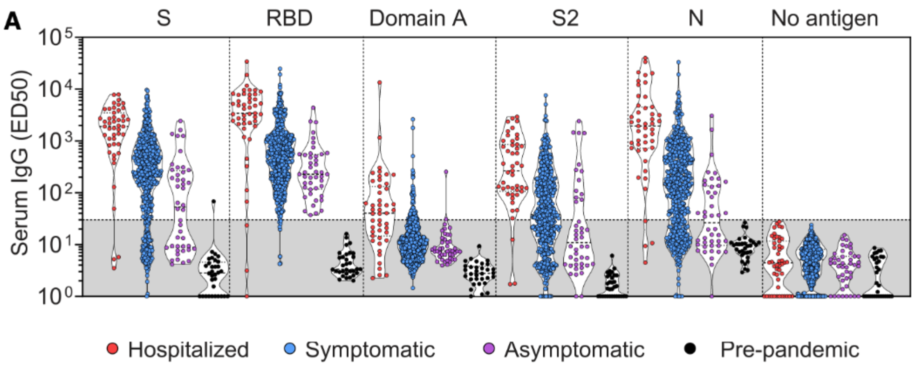

Figure 2-4 Asymptomatic Patients with COVID-19 have Lower IgG Titers Across Multiple Antigens Among Italian Medical Staff N>600 (13).

2.5 Burden of SARS-CoV-2 infection and COVID-19 disease among students

The study will be conducted among students enrolled in post-secondary educational institutions (eg, universities, vocational and technical colleges) because these are ideal locations for studying SARS-CoV-2 transmission clusters. Large numbers of cases of COVID-19 have been reported on numerous campuses throughout the US as colleges and universities re-opened in fall 2020. More than 397,000 cases were counted at 1,800+ colleges and universities in fall 2020 (17, 18). Over the time period July-December 11th, 2020, more than 85 colleges and Universities had more than 1,000 cases. Among these 49 campuses the cumulative attack rate from July to October was on average 6.2%. These estimates are somewhat uncertain given the fact that the number of reported cases for some schools includes faculty, staff and graduate students; however, the number of such individuals is very small in comparison to the number of undergraduates.

Among 15 Association of American Universities (AAU) universities, and using data from the start of semester through September 18, 2020, there was an estimated 3.7% 1-month attack rate. The attack rate appeared to decline over time; it was 1.7% over September 19 to October 20, 2020. Attack rates may be underestimated because the denominator is assumed to be all undergraduates, whereas some schools had only a subset of students on campus. These statistics suggest that a conservative assumption of SARS-CoV-2 incidence among US university students for Fall semester 2020 is 6%. A conservative incidence assumption for Spring and Summer 2021semesters, anticipating potential improvements in testing, contact tracing, social distancing measures, as well as vaccine rollout to priority populations outside the universities, is therefore 3-6%.

The outbreaks of SARS-CoV-2 infection at college campuses have been reported to be associated with students residing in high density housing, socializing off campus, and not following social distancing guidelines (19). In the US, it is estimated that 45 percent of young adults aged 18-22 years were enrolled in colleges and universities in 2019 (16). As such, much of the increase in cases this summer and fall are attributed to college students. The college campuses are associated with a high rate of SARS-CoV-2 infection and resulting asymptomatic carriage due to huge impulse to socialize as well as no fear of severe disease among the young students.

2.6 Age and Asymptomatic Infection

For this study, participants will be enrolled who are 18 to 26 years of age. This age range is chosen because of the high incidence of asymptomatic infection in this population. A considerable body of data suggest that SARS-CoV-2 can be spread by individuals with and without symptoms (20-23). VL data among individuals with acute SARS-CoV-2 infection also indicate that peak VL usually occurs before disease onset (22, 24). This suggests that there is a substantial transmission potential before symptom onset. According to the latest Centers for Disease Control and Prevention (CDC) estimate 40% of SARS-CoV-2 infections are asymptomatic (25). Available data indicate that younger individuals appear more likely to remain asymptomatic following infection than older individuals (26, 27). A recent study done in the UK showed about 80% of children between 10-19 years of age remain asymptomatic (28). As infected young students are less likely to show clinical symptoms, the number of clinical cases reported among young populations would be lower, but the asymptomatic young students could still be capable of transmitting the virus to others (29).

2.7 Evidence for viral load as a predictor of infectiousness

Mathematical modelling in human immunodeficiency virus (HIV) and herpesviruses and now COVID-19 suggests a significant percentage of transmission occurs from high peak VL which in all these diseases is during the asymptomatic period. Models by Josh Schiffer and Ashish Goyal suggest that a person has a 50% chance of infection with exposure to 10^7 SARS-CoV-2 RNA and <5% chance of infection with exposure to 10^5 SARS-CoV-2 RNA (Figure 2-5) (21). The model by Goyal et al (21) suggests that vaccine reduction in peak VL may reduce infectiousness. A 0.6 log reduction corresponds to ~50% VE_I and a 2.5 log

reduction to ~90% VE_I (Figure 2-6). This suggests collecting nasal swabs every other day would be required to measure the vaccine effect on peak VL and area under the VL curve. Asymptomatic and pre-symptomatic cases of SARS-CoV-2 appear to play an important role in the onward transmission of the virus.

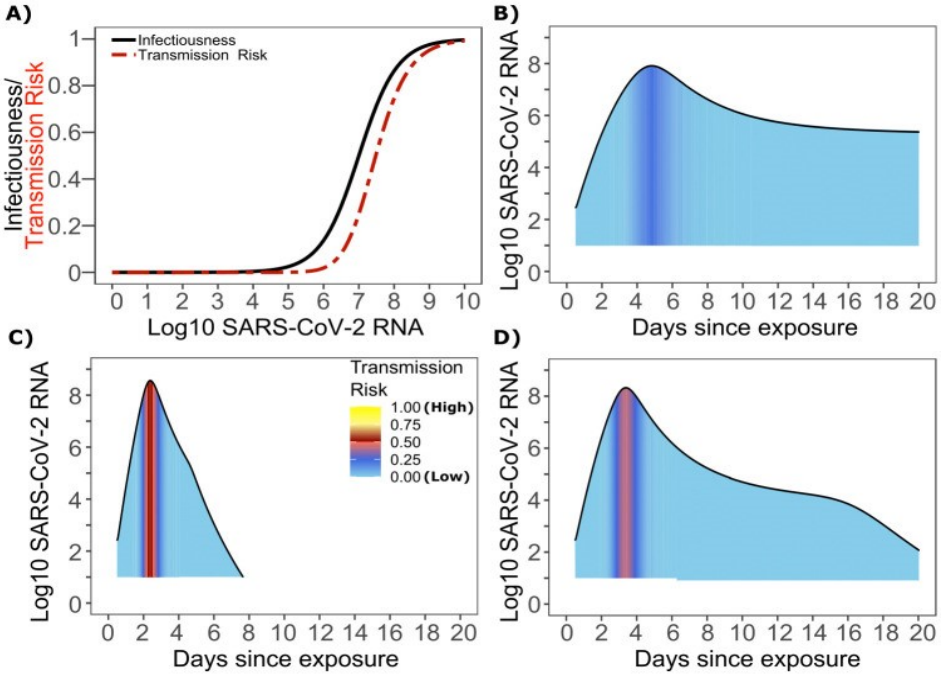

Figure 2-5 SARS-CoV-2 transmission probability as a function of shedding. A. Optimal infectious dose (ID) response curve (infection risk = Pt) and transmission dose (TD) response curve (transmission risk = Pt * Pt) curves for SARS-CoV-2. Transmission probability is a product of two probabilities, contagiousness and infectiousness. B-D. Three simulated viral shedding curves. Heat maps represent risk of transmission at each shedding timepoint given an exposed contact with an uninfected person at that time.

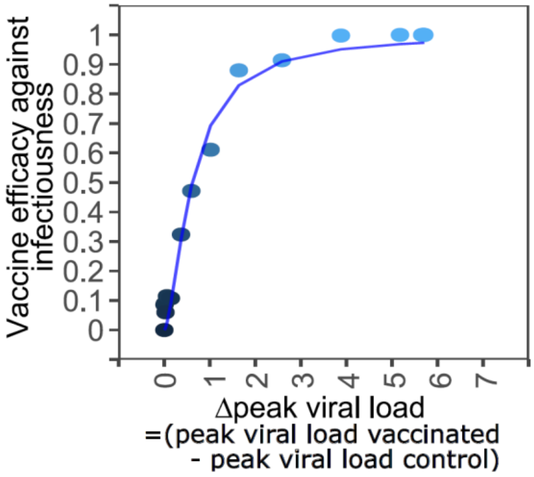

Figure 2-6 Vaccine efficacy against infectiousness versus the change in peak viral load. Model by Goyal et al (personal communication).

2.8 Evidence for viral load as a marker of COVID-19 disease severity and clinical course

Beyond infectiousness, there is a growing body of evidence supporting a linkage between VL and COVID-19 disease outcome. Two Phase 2/3 trials of monoclonal antibodies for COVID-19 treatment in outpatient settings found that VL at enrollment and early after intervention was associated with future clinical outcomes and disease course (30, 31). In the BLAZE-1 trial of intravenous infusion of the LY-CoV555 antibody, on day 7 post-infusion the frequency of hospitalization among patients with a high VL (Ct value of less than 27.5) was 12% (7 of 56 patients), as compared to 0.9% (3 of 340 patients) among those with a lower VL. Numerous observational studies have also identified associations between VL and clinical outcomes (32). The highest quality evidence comes from large cohort studies. In a large hospitalized cohort, Pujadas et al (33) estimated a highly significant 7% increase in mortality per log10 increase in VL at admission, after adjusting for other factors associated with clinical outcomes ($p = 0.0014$, multivariate Cox model). Chen et al (34) confirmed this finding in a larger cohort of patients admitted to three New York City hospitals. These data suggest that VL at and soon after the time of symptom onset may be a useful measure of disease severity.

2.9 Methods for capturing transmission

Reducing transmission is key to ending/controlling the COVID-19 pandemic. Due to vaccination, the number of asymptomatic individuals might increase as vaccine-induced immune responses may lead to VL reduction. However, it is still to be discovered whether those individuals will continue to shed and transmit virus to others. Hence, it is important to identify secondary transmissions from study participants to estimate secondary infections rates by arm and to validate VL as a surrogate for transmission risk. Onward transmission of SARS-CoV-2 infection from study participants can be identified in combination with sequence-analysis and phylogenetics methods (35).

Estimating vaccine efficacy in reducing transmission (VE_I), requires measuring transmission of SARS-CoV-2 from vaccinated and unvaccinated individuals to others (36, 37). Measuring transmission of infected individuals to uninfected individuals is challenging, especially for transient infections where it may be more difficult to ascertain which infection occurred first. For example, because HIV is not transient and per-exposure transmission is low, resulting in a potentially long time period of exposure, transmission can be studied in sero-discordant sexual partnerships. In a transient infection, ideally both members of the transmission pair are monitored before either is infected so that timing and direction of transmission can be easily determined. Prospectively following a cohort of vaccinated and unvaccinated individuals and their close contacts to observe whether infection is transmitted from study participants to close contacts, or from contacts to study participants, based on frequent testing of both allows for better quantification of the temporality of events regarding infection transmission, if it occurs. To do this, Datta et al. propose augmenting a randomized, controlled vaccine trial with a cohort of close contacts of trial participants. Both trial participants and the contact cohort are followed prospectively for acquisition, allowing for estimation of VE_I. Another approach to capturing transmission events is a case ascertainment

design (38, 39). Under this design, contacts of index cases are enrolled and tested as the cases emerge over time. A similar case ascertainment design was used for a recent household study for SARS-CoV-2 prevention. In the HCQ PEP trial, which was a household-randomized, double-blind, controlled trial of hydroxychloroquine postexposure prophylaxis to prevent acquisition of SARS-CoV-2 among close contacts recently exposed to persons diagnosed with SARS-CoV-2 infection, individuals were self-referred from infected individuals and enrolled online and all study procedures were done remotely including informed consent and daily self-nasal swabbing for 14 days (40). A case-ascertained approach is more resource-efficient than a prospective contact approach which requires enrollment of a large number of contacts, most of whom will not become infected nor have an infected index case in the main study. Because contacts are only identified and tested after index case diagnosis, the case-ascertained approach also enables assessment of secondary transmission in contacts not participating in frequent, prospective testing programs. However, the case-ascertained approach has the potential to miss or misclassify transmission events given the gap between diagnosing an index case and identifying, enrolling, and testing a contact. Index cases may also experience stigma when identifying contacts at the time of diagnosis, as opposed to when identifying contacts prospectively and before infection. The feasibility of recruiting and retaining contacts of students, and of classifying transmission events among contacts, using the prospective and case-ascertained designs has yet to be evaluated and compared.

This study will employ both the augmented and case ascertainment approaches to studying transmission through enrollment of two contact cohorts, a Prospective Close Contact (PCC) cohort and a Case-Ascertained Close Contact (CACC) cohort. The PCC Cohort will be identified at enrollment as close and sustained contacts of randomized participants (eg, roommates or co-workers). Participants in the PCC cohort may undergo routine screening for SARS-CoV-2 infection as part of their attendance or employment at their school, which may allow the participant and study team to receive real-time clinical diagnosis of COVID-19 during the study. Additionally, when a positive case is identified for a main study participant, the participant ('index case') will identify and refer their recent close contacts for enrollment into the CACC cohort. For both cohorts, infection diagnosis of the associated main study participant will trigger intensive short term sample collection (with a potential delay for the CACC cohort due to the need for informed consent and enrollment) including sera followed by daily nasal swabs for 14 days. Assessment of viral sequences, viral load trajectories, antibody detection and epidemiologic information from the infected participant and any positive close contacts in the PCC or CACC will aid in identifying transmission events between the participant and the contact. Details of the enrollment and procedures for both contact cohorts are described below.

2.10 Moderna COVID-19 Vaccine

This trial will utilize vaccine obtained under EUA by the US Government's COVID-19 Vaccine Response and allocated for this trial.

2.10.1 Clinical studies of Moderna COVID-19 Vaccine

Table 2-3 Summary of clinical studies

ClinicalTrials.gov Identifier	Study number	Phase	N	Dose groups	Route	Schedule	Reference
NCT04283461	P101	1	120	25, 50, 100, or 250 mcg	IM	M0, M1	(41, 42)
NCT04405076	P201	2	600	50 or 100 mcg	IM	M0, M1	(14, 15, 43)
NCT04470427	P301	3	30,351	100 mcg	IM	M0, M1	(14, 15, 43)

2.10.2 Potential risks of Moderna COVID-19 Vaccine

Risks associated with receipt of the Moderna COVID-19 Vaccine are described in the "Fact Sheet For Healthcare Providers Administering Vaccine (Vaccination Providers) Emergency Use Authorization (EUA) Of The Moderna COVID-19 Vaccine To Prevent Coronavirus Disease 2019 (COVID-19)", available at https://www.modernatx.com/covid19vaccine-eua/.

2.11 Trial design rationale

We are proposing a 2-arm randomized, controlled, open-label trial design. The design is augmented by two cohorts of contacts of study participants – the Prospective Close Contact (PCC) cohort and the Case-Ascertained Close Contact (CACC) cohort – to allow capture of secondary transmission events.

Participants in the main study will self-collect nasal swabs daily in order to capture all incident SARS-CoV-2 infection events over 4 months of follow-up and to capture the full course of viral shedding – from onset of infection to viral clearance – among those with both asymptomatic and symptomatic infection.

To minimize participant burden and maximize study efficiency, as many study procedures as possible will be conducted remotely (not requiring an in-person visit) and electronically, such as initial screening and consent. Participants diagnosed with SARS-CoV-2 infection will complete daily e-diaries to capture symptoms.

Given that primary analyses will be conducted among baseline seronegative participants, the rate of baseline SARS-CoV-2 seropositivity will be monitored operationally with an operational target of no more than 10% for the main study cohort.

2.11.1 Dose and schedule

The latest information regarding Moderna COVID-19 Vaccine dose and administration instructions can be found at https://www.modernatx.com/covid19vaccine-eua/.

For this study, vaccine will be administered at D1 and D29 for the immediate vaccination arm, and at D113 and D141 for the delayed vaccination arm.

2.11.2 Choice of delayed vaccination

There is no licensed SARS-CoV-2 vaccine currently available to serve as a reference control. Currently, there are several SARS-CoV-2 vaccines available in the US under Emergency Use Authorization (EUA), including the Moderna COVID-19 Vaccine being used in this study. Accordingly, a delayed vaccination arm has been chosen as an internal control. No placebo will be used, and participants will not be blinded to randomization assignment. The open-label design has been chosen primarily to achieve operational efficiency and to minimize resource utilization. Main study participants will be asked throughout the study about the timing and type of any outside vaccination, and contacts in the PCC and CACC will be queried about COVID-19 vaccine receipt. Main study participants receiving outside vaccination will remain on-study and follow the same study procedures after vaccination. Section 6.4.3.1 describes how the analysis of vaccine efficacy will take into account outside vaccination.

No placebo will be used, and participants will not be blinded to randomization assignment. The open-label design has been chosen primarily to achieve operational efficiency and to minimize resource utilization. With an open-label design, differences between the randomized arms in study endpoints including SARS-CoV-2 infection, viral load, and secondary transmission will capture not only biological effects of vaccination, but potentially behavioral effects influencing exposure, including adherence to study procedures. This is most relevant for the SARS-CoV-2 infection endpoint: if participants who are randomized to immediate vaccination engage in more risk-taking behavior and thus experience higher exposure to SARS-CoV-2 relative to participants randomized to delayed vaccination, ie, there is behavioral disinhibition, this will be captured in the estimate of vaccine efficacy against infection: the biological reduction in incidence of SARS-CoV-2 infection attributable to vaccination will be offset by an increase in incidence due to higher exposure. Therefore, the measure of vaccine efficacy against infection that is estimated in this study is in truth a measure of effectiveness. However, we retain the terminology "vaccine efficacy" for simplicity. Section 6.4.3.1 discusses this issue in greater detail, including how the statistical analysis will attempt to control for potential imbalances in SARS-CoV-2 exposure between treatment arms when evaluating vaccine efficacy, and how both efficacy and effectiveness measures will be evaluated and compared. We note that endpoints measured after diagnosis of SARS-CoV-2 infection, including SARS-CoV-2 viral load and secondary transmission, are viewed as much less likely to be influenced by participant behavior and knowledge of vaccine receipt.

3 Objectives and endpoints

**Primary Objectives**	**Primary Endpoints**
1. To evaluate the efficacy of Moderna COVID-19 Vaccine against SARS-CoV-2 infection (ie, to evaluate vaccine efficacy against infection or VE_S).	SARS-CoV-2 infection diagnosed by PCR post visit 3 (month 1) among participants who were SARS-CoV-2 seronegative at enrollment and SARS-CoV-2 negative by PCR through visit 3 (month 1)
2. To evaluate the effect of Moderna COVID-19 Vaccine on peak nasal viral load as a measure of infection and a proxy of infectiousness	Peak viral load in nasal samples from participants diagnosed with SARS-CoV-2 infection post visit 3 (month 1), among participants who were SARS-CoV-2 seronegative at enrollment and SARS-CoV-2 negative by PCR through visit 3 (month 1)
**Secondary Objectives**	**Secondary Endpoints**
1. To evaluate the impact of Moderna COVID-19 Vaccine on secondary transmission of SARS-CoV-2 infection, using data for the Prospective Close Contact (PCC) cohort and Case-Ascertained Close Contact (CACC) cohort (ie, to evaluate vaccine efficacy against infectiousness or VE_I)	Number of adjudicated* secondary transmission events in PCC and CACC cohorts from main study participants who were SARS-CoV-2 seronegative at enrollment and SARS-CoV-2 negative by PCR through visit 3 (month 1).<br><br>*based on questionnaire-based epidemiologic data, antibody and viral dynamics, and viral sequence data
2. To evaluate the efficacy of Moderna COVID-19 Vaccine to prevent serologically confirmed SARS-CoV-2 infection	SARS-CoV-2 antibodies to the nucleocapsid protein post visit 3 (month 1), among participants who were SARS-CoV-2 seronegative at enrollment and SARS-CoV-2 negative by PCR through visit 3 (month 1)
3. To evaluate vaccine efficacy against COVID-19 disease, (ie, to evaluate VE_D) using weekly e-diaries and confirmatory PCR testing	SARS-CoV-2 infection confirmed by PCR and diagnosed post-visit 3 (month 1), among participants SARS-CoV-2 who were seronegative at enrollment and SARS-CoV-2 negative by PCR through visit 3 (month 1), coincident with the following symptoms:<br><br>• at least TWO of the following systemic symptoms: Fever (≥ 38ºC), chills, myalgia, headache, sore throat,<br><br>OR<br><br>at least ONE of the following signs/symptoms: cough, shortness of breath or difficulty breathing, new olfactory or taste disorder, clinical or radiographical evidence of pneumonia, thromboembolic event,

	myocardial infarction, myocarditis, chilblains, or multi-inflammatory syndrome
4. To evaluate vaccine effect on additional measures of magnitude of viral load over time, e.g. area under the viral load curve, duration of shedding, and time to log10 viral load above 10^5 copies/mL	Summary measures of the viral load curve, all evaluated among participants diagnosed with SARS-CoV-2 infection post-visit 3 (month 1) who were SARS-CoV-2 seronegative at enrollment and SARS-CoV-2 negative by PCR through visit 3 (month 1):<br>• Transmission potential endpoint, defined as 0 for an individual without SARS-CoV-2 infection, and peak log10 viral load, for an individual with SARS-CoV-2 infection<br>• Area under log10 viral load curve (AUC)<br>• Duration of viral shedding (viral load above assay limit of detection)<br>• Area under log10 viral load curve above 10^5 copies/mL<br>• Duration of viral shedding above 10^5 copies/mL<br>• Time from enrollment to viral load above 10^5 copies/mL
5. To evaluate vaccine efficacy against SARS-CoV-2 infection, the vaccine effect on viral load, and the vaccine effect on secondary transmission in all enrolled participants without regard to baseline SARS-CoV-2 serostatus	• SARS-CoV-2 infection diagnosed by PCR [Primary endpoint 1]<br>• Peak log10 viral load in nasal samples from participants who are diagnosed with SARS-CoV-2 infection [Primary endpoint 2]<br>• Number of secondary transmission events in the PCC and CACC [Secondary endpoint 1]
6. To evaluate the immunogenicity of the vaccine	Magnitude and response rate of immune responses to vaccination measured at Months 0, and 2 stratified by baseline SARS-CoV-2 serostatus as measured by binding antibody and neutralization assays in the case-cohort immunogenicity set
7. To evaluate immune responses 1 month post-second vaccination as correlates of risk of SARS-CoV-2 acquisition, viral load, secondary infection, and COVID-19 disease among vaccine recipients in the immediate vaccination group	Magnitude and response rate of immune responses to vaccination in the immediate vaccination group and measured at Months 0 and 2, stratified by baseline SARS-CoV-2 serostatus, as measured by binding antibody and neutralization assays in the case-cohort immunogenicity set
8. To evaluate vaccine efficacy against asymptomatic SARS-CoV-2 infection	SARS-CoV-2 infection by PCR or periodic serology post-visit 3 (month 1) among participants seronegative for SARS-CoV-2 at baseline, SARS-CoV-2 negative

	by PCR through visit 3 (month 1), and without any prior reporting of symptoms that led to confirmation of a COVID-19 disease endpoint
9. To evaluate vaccine efficacy against SARS-CoV-2 infection and COVID-19 disease and the vaccine effect on viral load and secondary transmission among participants who received all planned immunizations at the designated immunization visits within specific visit windows	Endpoints evaluated among participants who were SARS-CoV-2 seronegative at enrollment and SARS-CoV-2 negative by PCR through visit 3 (month 1) and counting SARS-CoV-2 infection events post-visit 3 (month 1)<br>• SARS-CoV-2 infection diagnosed by PCR [Primary endpoint 1]<br>• Peak log10 viral load in nasal samples from participants who are diagnosed with SARS-CoV-2 infection [Primary endpoint 2]<br>• Number of secondary transmission events in the PCC and CACC [Secondary endpoint 1]<br>• SARS-CoV-2 infection coincident with signs and symptoms of COVID-19 listed under Secondary endpoint 3
10. To evaluate the association between measures of the magnitude of viral load over time and secondary transmission of SARS-CoV-2 infection, among individuals with incident SARS-CoV-2 infection in each treatment group, i.e. to conduct analyses aimed at evaluating viral load measures as surrogates of secondary transmission	• Peak log10 viral load [Primary endpoint 2] and other viral load summaries [Secondary endpoints 4]<br>• Number of secondary transmission events in the PCC cohort and CACC cohort [Secondary endpoint 1]
11. To evaluate vaccine effects on peak viral load and on secondary transmission of SARS-CoV-2 infection separately among individuals with asymptomatic vs. symptomatic SARS-CoV-2 infection	• Peak log10 viral load [Primary endpoint 2] and other viral load summaries [Secondary endpoints 4]<br>• Number of secondary transmission events in the PCC cohort and CACC cohort [Secondary endpoint 1]<br>• SARS-CoV-2 infection diagnosed using PCR, with and without signs and symptoms of COVID-19 listed under Secondary endpoint 3

Exploratory Objectives

1. To evaluate the associations between demographic and clinical factors and secondary transmission of SARS-CoV-2 infection and COVID-19 disease, among individuals with incident SARS-CoV-2 infection in the delayed vaccination arm and their contacts in the PCC and CACC
2. To assess if and how vaccine efficacy against SARS-CoV-2 infection, transmission and COVID-19 disease depends on baseline demographic, clinical, socio-economic indicators, and/or behavioral characteristics of study participants

3. To assess if and how vaccine efficacy against SARS-CoV-2 infection and COVID-19 disease depends on genotypic or neutralization phenotypic characteristics of SARS-CoV-2 (sieve analysis)
4. To further evaluate immunogenicity of the vaccine, additional immunogenicity assays may be performed such as antibody (Ab) Avidity and Fc Receptor function assays, based on the CoVPN Laboratory Center assay portfolio
5. To conduct analyses related to furthering the understanding of SARS-CoV-2, immunology, vaccines, and clinical trial conduct

4 Ethical considerations

It is critical that universally accepted ethical guidelines are followed at all sites involved in the conduct of clinical trials. The COVID-19 Prevention Network (CoVPN) have addressed ethical concerns in the following ways:

- CoVPN trials are designed and conducted to enhance the scientific knowledge base necessary to find a preventive vaccine, using methods that are scientifically rigorous and valid, and in accordance with International Council for Harmonisation of Technical Requirements for Pharmaceuticals for Human Use (ICH) and/or other Good Clinical Practice (GCP) guidelines.

- CoVPN scientists and operational staff incorporate the philosophies underlying major codes (44-46), declarations, and other guidance documents relevant to human subjects research into the design and conduct of SARS-CoV-2 vaccine clinical trials.

- CoVPN scientists and operational staff are committed to substantive community input—into the planning, conduct, and follow-up of its research—to help ensure that locally appropriate cultural and linguistic needs of study populations are met.

- The CoVPN provides training so that all participating sites similarly ensure fair participant selection, protect the privacy of research participants, and obtain meaningful informed consent. During the study, participants will have their wellbeing monitored, and to the fullest extent possible, their privacy protected. Participants may withdraw from the study at any time.

- Prior to implementation, CoVPN trials are rigorously reviewed by scientists who are not involved in the conduct of the trials under consideration.

- CoVPN trials are reviewed by local and national regulatory bodies and are conducted in compliance with all applicable national and local regulations.

- The CoVPN designs its research to minimize risk and maximize benefit to both study participants and their local communities. For example, CoVPN protocols provide enhancement of participants' knowledge of SARS-CoV-2 and SARS-CoV-2 prevention, as well as counseling, guidance, and assistance with any social impacts that may result from research participation. CoVPN protocols also include careful medical review of each research participant's health conditions and reactions to study products while in the study.

- CoVPN research aims to benefit local communities by directly addressing the health and SARS-CoV-2 prevention needs of those communities and by strengthening the capacity of the communities through training, support, shared knowledge, and equipment. Researchers involved in CoVPN trials are able to conduct other critical research in their local research settings.

- The CoVPN values the role of in-country Institutional Review Boards (IRBs), Ethics Committees (ECs), and other Regulatory Entities (REs) as custodians responsible for ensuring the ethical conduct of research in each setting.

5 IRB/EC review considerations

US Food and Drug Administration (FDA) and other US federal regulations require IRBs/ECs/REs to ensure that certain requirements are satisfied on initial and continuing review of research (Title 45, Code of Federal Regulations (CFR), Part 46.111(a) 1-7; 21 CFR 56.111(a) 1-7). The following section highlights how this protocol addresses each of these research requirements. Each CoVPN Investigator welcomes IRB/EC/RE questions or concerns regarding these research requirements.

5.1 Minimized risks to participants

45 CFR 46.111 (a) 1 and 21 CFR 56.111 (a) 1: Risks to subjects are minimized.

This protocol minimizes risks to participants by (a) correctly and promptly informing participants about risks so that they can join in partnership with the researcher in recognizing and reporting harms; (b) respecting local/national blood draw limits; (c) performing direct observation of participants postvaccination and reporting information regarding side effects to the FDA/CDC Vaccine Adverse Event Reporting System (VAERS) per EUA guidance; (d) having staff properly trained in administering study procedures that may cause physical harm or psychological distress, such as blood draws, vaccinations, COVID-19 testing and counseling; and (e) providing safety monitoring.

5.2 Reasonable risk/benefit balance

45 CFR 46.111(a) 2 and 21 CFR 56.111(a) 2: Risks to subjects are reasonable in relation to anticipated benefits, if any, to subjects, and the importance of the knowledge that may reasonably be expected to result.

In all public health research, the risk-benefit ratio may be difficult to assess because the benefits to participants in prevention protocols are not as apparent as they would be in treatment protocols, where a study participant may be ill and may have exhausted all conventional treatment options. However, this protocol is designed to minimize the risks to participants while maximizing the potential value of the knowledge it is designed to generate.

5.3 Equitable participant selection

45 CFR 46.111 (a) 3 and 21 CFR 56.111 (a) 3: Subject selection is equitable

This protocol has specific inclusion and exclusion criteria for investigators to follow in admitting participants into the protocol. Participants are selected because of these criteria and not because of positions of vulnerability or privilege. Investigators are required to maintain screening and enrollment logs to document volunteers who screened into and out of the protocol and for what reasons.

5.4 Appropriate informed consent

45 CFR 46.111 (a) 4 and 5 and 21 CFR 56.111 (a) 4 and 5: Informed consent is sought from each prospective subject or the subject's legally authorized representative as required by 45 CFR 46.116 and 21 CFR Part 50; informed consent is appropriately documented as required by 45 CFR 46.117 and 21 CFR 50.27

The protocol specifies that informed consent must be obtained before any study procedures are initiated and assessed throughout the trial (see Section 9.1). Each site is provided training in informed consent by the CoVPN and is required to have a Standard Operating Procedure (SOP) on the informed consent process. The CoVPN requires a signed consent document for documentation, in addition to chart notes or a consent checklist.

5.5 Adequate safety monitoring

45 CFR 46.111 (a) 6 and 21 CFR 56.111 (a) 6: There is adequate provision for monitoring the data collected to ensure the safety of subjects.

Vaccination providers are required to report to VAERS the certain adverse events after COVID-19 vaccination, under Emergency Use Authorization (EUA) and encouraged to report adverse events to ModernaTX |(https://www.cdc.gov/vaccines/covid-19/vaccination-provider-support.html, see Section 11). Additionally, the use of v-safe will be recommended to vaccine recipients (https://www.cdc.gov/coronavirus/2019-ncov/vaccines/safety/vsafe.html, see Section 11), and an Independent Data Monitoring Committee (IDMC) will periodically review study data.

5.6 Protect privacy/confidentiality

45 CFR 46.111 (a) 7 and 21 CFR 56.111 (a) 7: There are adequate provisions to protect the privacy of subjects and maintain the confidentiality of data.

Privacy refers to an individual's right to be free from unauthorized or unreasonable intrusion into his/her private life and the right to control access to individually identifiable information about him/her. The term "privacy" concerns research participants or potential research participants as individuals whereas the term "confidentiality" is used to refer to the treatment of information about those individuals. This protocol respects the privacy of participants by informing them about who will have access to their personal information and study data (see Appendix A, Appendix B and Appendix C). The privacy of participants is protected by assigning unique identifiers in place of the participant's name on study data and specimens. In the United States, research participants in CoVPN protocols are protected by a Certificate of Confidentiality from the US NIH, which can prevent disclosure of study participation even when that information is requested by subpoena. Participants are told of the use and limits of the certificate in the study consent form. In addition, each staff member at each study site in this protocol signs an Agreement on Confidentiality and Use of Data and Specimens with the

CoVPN. In some cases, a comparable confidentiality agreement process may be acceptable. Each study site participating in the protocol is required to have a standard operating procedure on how the staff members will protect the confidentiality of study participants.

6 Statistical considerations

We anticipate enrolling approximately 12,000 students. Participants will be randomized 1:1 to immediate or delayed vaccination, at Months 0 and 1 or Months 4 and 5, respectively.

Primary analyses of vaccine efficacy against infection (VE_S) and of the vaccine effect on viral load (ΔVL) will be conducted among participants SARS-CoV-2 seronegative at enrollment in the "Day 29 Negative Set", i.e. the set of participants without PCR-confirmed infection on or prior to Day 29; see Table 6-1 for a full definition of the various analysis sets for the study. Primary efficacy analyses will only count SARS-CoV-2 infection events diagnosed post-Day 29. Secondary analyses will be conducted among all participants who receive both immunizations without protocol violation, i.e. the Per-Protocol Set, and in the set of all enrolled participants (the "Full Analysis Set" or FAS), counting infection events diagnosed after enrollment (Day 1). Immunogenicity assessment will be done in the Case-Cohort Immunogenicity Set—a subsample of the participants randomized to immediate vaccination and sampled on the basis of SARS-CoV-2 infection status and baseline characteristics that will be fully described in the study SAP.

The FAS differs from a full Intention-to-Treat set by excluding randomized volunteers who do not enroll. The exclusion of these individuals is justified because the effects of the vaccine are unlikely to influence such exclusion. Because of the brief length of time between randomization and enrollment (ideally these will be on the same day), we expect almost all randomized volunteers to be in the FAS.

All primary and secondary analyses will be done according to the treatment randomly assigned ("as randomized").

Main study participants will be tested using real-time PCR testing of swabs, and these data will be used to identify SARS-CoV-2 infection events for primary analysis. Some main study participants may also receive SARS-CoV-2 testing through university testing systems, or through outside providers, and will be asked to report any positive test results to the study team. Any positive SARS-CoV-2 test results for main study participants – based on PCR testing of study swabs or based on other testing systems indicative of acute infection– will trigger swabbing of contacts in the PCC and enrollment and swabbing of contacts in the CACC. Secondary analyses of vaccine efficacy will include SARS-CoV-2 infection events diagnosed through any testing system – either by PCR testing of study swabs or through outside testing.

The two contact cohorts – the PCC and the CACC – will both be used for analyses of the vaccine effect on secondary transmission, as described below.

The study is "time-driven", i.e. the primary analysis will occur when all participants have completed the 4 months follow-up. The number of primary endpoint infection events that accrue in the study is random and will depend on the level of SARS-CoV-2 incidence in each arm.

Table 6-1 Study Analysis Sets and Definitions

Analysis Set	Definition	Analyses
Full Analysis Set (FAS)	Randomized participants who enroll in the study	Secondary efficacy analyses*
Day 29 Negative (D29-) Set	Participants in the FAS without PCR-confirmed infection on or before Day 29[¥]	Primary efficacy analyses*
Per-Protocol (PP) Set	Participants in the D29- set who received all planned immunizations at the designated immunization visits within specific visit windows	Secondary efficacy analyses*
Day 29 Negative Transmission Set (D29-TS)	Participants in the D29- set and their contacts in the Prospective and Case Ascertained Contact Cohorts	Primary analyses of secondary transmission*
Case-Cohort Immunogenicity Set (ccIS)	Subset of the FAS that is randomly sampled for measurement of immunological markers and fully described in study SAP	Immunology marker analyses

*These analysis sets include participants without regard to baseline SARS-CoV-2 status. However, primary efficacy analyses will condition on baseline SARS-CoV-2 seronegative status.
[¥] Note that Day 29 is the second vaccination visit (visit 3) for participants on the immediate vaccination arm. The SAP will specify the definition of Day 29 for participants on the delayed vaccination arm.

Table 6-2 Assumptions underlying study power/sample size calculations, unless otherwise specified. Parameters and their assumed values pertain to participants in the main study cohort.

Parameter	Assumption
Enrollment	Uniform enrollment over 4 weeks
Baseline seroprevalence in main study and PCC and CACC	10% of enrolled participants are baseline SARS-CoV-2 seropositive. This parameter is monitored and controlled operationally (see Section 6.3)
Per-protocol rate	98% of enrolled participants are in the per-protocol set
Loss to follow-up rate in the main study, the PCC and the CACC	5% of participants are lost to follow-up over 16 weeks at a constant rate
SARS-CoV-2 incidence in the delayed vaccination group and incidence un-linked to main study participants in the PCC	Exponential (constant rate) over 16 weeks; primary assumption is 4% over 16 weeks
Secondary transmission rate among susceptible contacts, in the delayed vaccination arm	15% transmission rate based on (47-49)

Vaccine efficacy (VE) against SARS-CoV-2 infection and transmission	Constant after 14 days post-first vaccination (calculations do not depend on earlier VE); level is varied
Contact enrollment	For the PCC: 60% of main study participants enroll contact(s) and the average number of contacts enrolled is 2<br><br>For the CACC: 75% of main study participants enroll contact(s) and the average number of contacts enrolled is 3

6.1 Accrual and sample size calculations

A sample size of 6,000 participants in each treatment arm ensures a high probability that a sufficient number of primary endpoint events, i.e. SARS-CoV-2 infections among baseline seronegative participants in the D29- set and diagnosed after Day 29, will accrue to adequately power analyses addressing both primary objectives as well as key secondary objectives. Specifically, under a 4% SARS-CoV-2 incidence over 16 weeks in the delayed vaccination group (a conservative assumption given recent incidence on US college campuses, see Section 2.5), assuming VE_S = 50% and other assumptions laid out in Table 6-2, 225 primary endpoint events will occur across both arms with probability 0.5 and 214 events will occur with probability 0.8.

Table 6-3 shows the expected number of primary endpoint events, overall and for each treatment group, as the assumed incidence in the delayed vaccination group varies. As shown, the design ensures a sufficient number of primary endpoint events to address a variety of objectives that condition on the primary endpoint. For example, with 4% SARS-CoV-2 incidence in the delayed vaccination group, 75 primary endpoint events are expected in the immediate vaccination group for immune correlates analyses.

Table 6-3 Expected number of primary endpoint events, available for secondary analyses that condition on primary endpoint event status. Calculations assume 6,000 participants per arm and other assumptions in **Table 6-2**, VE_S = 50%, and varying 16-week incidence of SARS-CoV-2 infection in the delayed vaccination group.

**16-Week SARS-CoV-2 Incidence in Placebo Arm**	**Number of Primary Endpoint Events**	
	Both Arms	Immediate Vaccination Arm
1.5%	86	28
2%	143	47
3%	170	56
3.5%	198	66
4%	225	75

The study provides high power to evaluate the vaccine effect on acquisition (VE_S). Figure 6-1 shows the power of the study to detect a given level of VE_S, rejecting H_0: $VE_S \leq 30\%$, based on a 1-sided 0.025-level log-rank test and as a function of the SARS-CoV-2 incidence in the delayed vaccination group. With 4% 16-week incidence, the study has 90% power to detect any $VE_S > 57\%$; with 3% incidence, $VE_S > 60\%$ can be detected with 90% power.

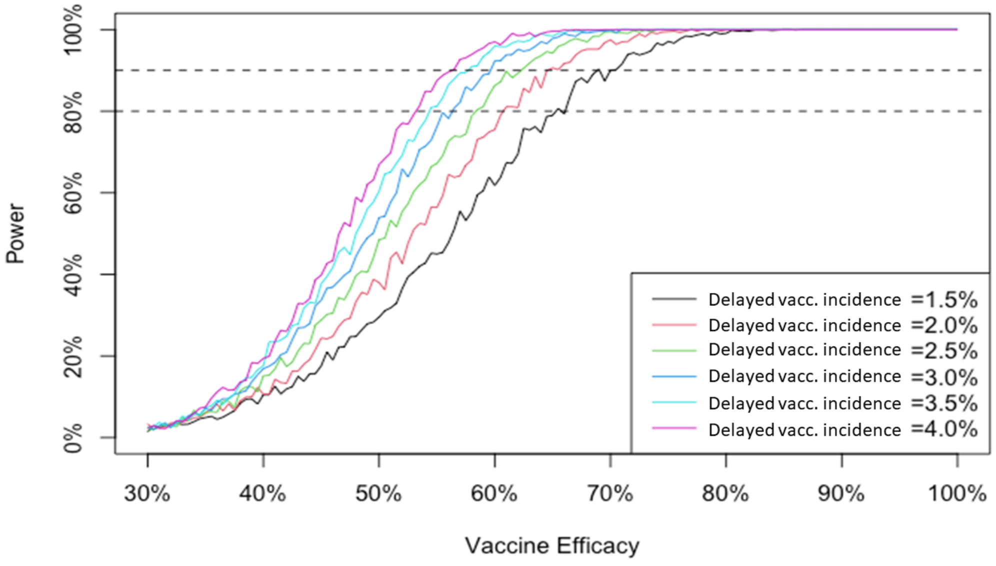

Figure 6-1 Power to detect varying vaccine efficacy (VE) against SARS-CoV-2 infection as a function of the delayed vaccination group incidence over 16 weeks. Simulation-based power calculations assume 6,000 participants in each arm and other assumptions listed in **Table 6-2**. Power based on 1-sided 0.025-level log-rank test and 1,000 simulations.

The proposed study also provides adequate precision with which to estimate VE_S. Table 6-4 shows that, with 225 primary endpoint events, the approximate 95% confidence interval (CI) about an estimated 50% VE_S is (36.9%, 60.4%). More precise inference is obtained under higher VE_S and higher primary endpoint totals.

Table 6-4 Approximate 95% confidence interval around a given estimated VE_S against SARS-CoV-2 infection, comparing the immediate vaccination arm vs. the delayed vaccination arm, as a function of the number of primary endpoint events across the two groups.

Number of primary endpoint events	Approximate 95% CI for estimated VE = 50%	Approximate 95% CI for estimated VE = 70%
200	(36.0%, 60.9%)	(60.7%, 77.2%)
225	(36.9%, 60.4%)	(61.1%, 76.9%)
250	(37.7%, 59.9%)	(61.6%, 76.6%)

6.1.1 Power and precision for evaluating the vaccine effect on VL

Emerging data on acute SARS-CoV-2 infection suggest that with an expected 225 primary endpoint events, the vaccine effect on mean peak log10 viral load among primary endpoint infections can be estimated with adequate precision. Specifically, assuming that peak log10 viral load is normally distributed with a 1.8 standard deviation in both treatment groups, and assuming 50% VE_S, a 1-log reduction in mean peak viral load can be detected with 96.9% power given 225 primary endpoint events; power remains high (93.5%) with 70% VE_S. Table 6-5 shows power to detect a 1-log reduction in peak log viral load as a function of the assumed standard deviation in log peak viral load which is allowed to vary between 1.0 and 2.5. The standard deviation in peak log viral load in a cohort of 68 individuals associated with the National Basketball Association who were diagnosed with acute SARS-CoV-2 infection was 1.4 to 1.8, depending on the set of samples included in the analysis (50). Emerging data from the HCQ-PEP study of ~100 acutely infected household members of recently diagnosed COVID-19 cases suggests a standard deviation in peak log viral load in the range of 2.0-2.2, depending on the set of samples included [data not published]. Table 6-6 shows the approximate 95% CI about an estimated 1-log reduction in mean peak log viral load, demonstrating the precision that is obtained with 225 primary endpoint events. Modest precision is observed with a 1-1.8 standard deviation in the endpoint, with lower precision under a higher standard deviation; precision is also lower under increasing VE_S.

Table 6-5 Power to detect a 1-log reduction in mean peak log viral load comparing primary endpoint infection events between the immediate and delayed vaccination groups, as a function of the assumed standard deviation in peak log viral load in both groups and the assumed level of VE against SARS-CoV-2 infection. Simulation-based power calculations assume 225 primary endpoint events across treatment groups. Power based on 1-sided 0.025-level t-test and 1,000 simulations.

Std. dev. in peak log viral load	VE_S = 50%	VE_S = 70%
**1**	100%	100%
**1.5**	99.6%	97.8%
**1.8**	96.9%	93.5%
**2**	95.6%	86.5%
**2.5**	79.9%	68.7%

Table 6-6 Approximate 95% confidence interval around an estimated 1-log reduction in mean peak log viral load comparing primary endpoint infection events between immediate and delayed vaccination groups, as a function of the assumed standard deviation in peak log viral load in both groups and the assumed level of vaccine efficacy against SARS-CoV-2 infection. Calculations assume 225 primary endpoint events across treatment groups.

Std. dev. in peak log viral load	VE_S = 50%	VE_S = 70%
**1**	(0.72, 1.28)	(0.68, 1.31)
**1.5**	(0.57, 1.41)	(0.51, 1.46)
**1.8**	(0.50, 1.50)	(0.44, 1.57)
**2**	(0.46, 1.57)	(0.36, 1.61)
**2.5**	(0.29, 1.65)	(0.20, 1.74)

6.1.2 Power for evaluating the vaccine effect on secondary transmission

The study has adequate power to evaluate vaccine efficacy against secondary transmission, based on analyses that are conducted among primary endpoint cases and their contacts in the PCC and CACC, i.e. based on conditional analyses, as long as VE against SARS-CoV-2 infection is low. As demonstrated in Figure 6-2, if there is high vaccine efficacy against SARS-CoV-2 infection, power is low to evaluate secondary transmission using a conditional analysis. In this case, an unconditional analysis of VE against secondary transmission is expected to be more powerful, and also more appropriate given that it reflects that infections blocked have no transmission potential.

We performed simulation-based calculations to explore the power to evaluate VE against secondary transmission based on a conditional analysis. In addition to the assumptions in Table 6-2, we assumed: equal infection surveillance in the PCC and CACC, for simplicity; that PCC contacts acquire infection from outside sources (other than the index case) at the same rate as the delayed vaccination arm; that only baseline seronegative contacts and those in the PCC not infected by outside sources are at risk of secondary transmission; that the number of secondary transmission events among at-risk contacts follows a Poisson distribution in each arm, with VE against secondary transmission reducing the event rate proportionally in the immediate vaccination group. A one-sided 0.025-level likelihood ratio test based on a Poisson regression model was used to evaluate VE against secondary transmission. As shown in Figure 6-2, the study has 90% power to detect a 59% reduction in secondary transmission if VE_S = 50%; a higher 69% reduction in secondary transmission is detectable if VE_S = 70%. Table 6-7 helps explain the power results: it shows the amount of statistical information on which the analysis is based, ie, the median number of primary endpoint events and secondary endpoint events per arm. With 50% VE_S and 50% VE against secondary transmission, we expect 15 secondary transmission events in the immediate vaccination arm and 59 in the delayed arm; the limited number of events is due to the "transmission cascade" whereby under our assumptions only some primary endpoint events

enroll contacts into the PCC and CACC, only some contacts are susceptible and retained, and the transmission rate is low (15%).

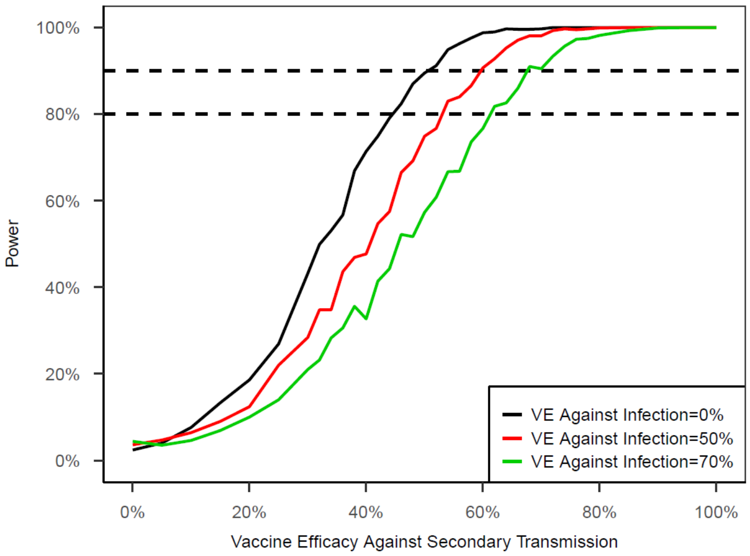

Figure 6-2 Power to detect varying vaccine efficacy against secondary transmission as a function of vaccine efficacy against SARS-CoV-2 infection. Simulation-based power calculations assume 6,000 participants in each arm and other assumptions listed in Table 6-2. Power based on 1-sided 0.025-level likelihood ratio test under Poisson regression model and 1,000 simulations.

Table 6-7 Number of primary endpoint events and secondary transmission events in each arm, under varying VE against SARS-CoV-2 infection and varying VE against secondary transmission, under other assumptions laid out in Table 6-2, and based on 1,000 simulations.

	**VE_S = 50%**		**VE_S = 70%**	
VE against secondary transmission	Median no. primary endpoints, Immediate vs. Delayed arms (IQR)	Median no. secondary transmissions, Immediate vs. Delayed arms (IQR)	Median no. primary endpoints, Immediate vs. Delayed arms (IQR)	Median no. secondary transmissions, Immediate vs. Delayed arms (IQR)
VE = 0%	76 (70-82)<br>vs. 150 (142-158)	29 (25-34)<br>vs. 59 (53-66)	45 (41-50)<br>vs. 149 (141-158)	18 (14-21)<br>vs. 59 (53-65)
VE = 40%	76 (70-81)<br>vs. 150 (142-158)	18 (15-21)<br>vs. 59 (53-64)	45 (41-50)<br>vs. 149 (142-158)	11 (8-13)<br>vs. 59 (53-65)
VE = 50%	76 (70-82)<br>vs. 150 (143-159)	15 (12-17)<br>vs. 59 (53-66)	45 (41-50)<br>vs. 150 (142-158)	9 (7-11)<br>vs. 59 (53-65)
VE = 60%	76 (70-82)<br>vs. 150 (142-158)	12 (9-14)<br>vs. 58 (52-65)	45 (41-50)<br>vs. 151 (143-159)	7 (5-9)<br>vs. 59 (53-65)
VE = 70%	75 (70-81)<br>vs. 150 (142-158)	9 (7-11)<br>vs. 58 (52-65)	46 (41-50)<br>vs. 150 (142-159)	5 (4-7)<br>vs. 59 (53-65)

6.2 Role of the Independent Data Monitoring Committee (IDMC)

The study IDMC will provide study oversight and ensure that the study is on-target to meeting its objectives in a timely fashion. The IDMC will receive summaries of study progress throughout the study. Given the short duration of the study and the considerable prior data establishing safety and efficacy of the vaccine, and the status of the EUA vaccine, there will be no pre-specified interim monitoring boundaries for VE or non-efficacy. The IDMC will make recommendations to protocol chairs and lead advisors. The set of metrics, operational targets for them, and potential remedial actions will be laid out in full in the IDMC Charter.

6.3 Randomization

A participant's randomization assignment will be computer generated and provided to the CRS study staff through a Web-based randomization system.

Randomization will be stratified. The randomization will be done in blocks to ensure balance among contemporaneously evaluated immediate and delayed vaccination arms within levels defined by study site and type of residence.

6.4 Statistical analyses

This section describes the final study analyses which will occur after the last participant has completed the Month 4 visit. All data from enrolled participants will be analyzed according to the initial randomization assignment regardless of how and when vaccinations are received. In the rare instance that a participant receives vaccination at the wrong time, the Statistical Analysis Plan (SAP) will address how to analyze the data. Analyses are modified intent-to-treat in that individuals who are randomized but not enrolled do not contribute data and hence are excluded. Because of the brief length of time between randomization and enrollment—typically these will happen on the same day—very few such individuals are expected.

Analyses for primary endpoints will be performed using SAS and R. All other descriptive and inferential statistical analyses will be performed using SAS, StatXact, or R statistical software.

In general, no formal multiple comparison adjustments will be employed for multiple secondary endpoints. However, multiplicity adjustments will be made for certain immunogenicity assays, as discussed below, when the assay endpoint is viewed as a collection of hypotheses (eg, testing multiple peptide pools to determine a positive response).

6.4.1 Analysis variables

The analysis variables defined for all participants in the main study cohort consist of baseline participant characteristics, longitudinal measures of risk of infection, SARS-CoV-2 infection

and seroconversion and COVID-19 disease status, and immune response biomarkers. For individuals diagnosed with SARS-CoV-2 infection, post-infection variables will include various summaries of VL, the full genome sequence of the infecting virus, and the infection status of and relationship to each of their contacts enrolled in the PCC and CACC. For contacts enrolled in the PCC and CACC, variables consist of baseline participant characteristics, SARS-CoV-2 infection and seroconversion status, risk factors around time of exposure to main study participant (if infected) and relationship to the referring main study participant.

6.4.2 Baseline comparability and comparability over study follow-up

Treatment arms will be compared with respect to baseline participant characteristics using descriptive statistics.

Given that the study is open-label, and the associated potential for differences between arms with respect to retention, compliance with study procedures, ascertainment of endpoints, and variables associated with exposure to SARS-CoV-2, these measures will be compared between treatment arms over time using methods fully described in the SAP. Imbalances between arms will influence the analysis choices as described below.

6.4.3 Vaccine efficacy (VE) analyses against SARS-CoV-2 infection

The time origin for all analyses of SARS-CoV-2 infection is Day 1. We define the time of diagnosis of the SARS-CoV-2 infection endpoint as the collection time of the first daily swab with a positive PCR test. The time of diagnosis of lab-confirmed clinical COVID-19 disease is the first time of reported qualifying COVID-19 symptoms that occurs simultaneously or after the diagnosis of the SARS-CoV-2 infection endpoint. The time of diagnosis of asymptomatic SARS-CoV-2 infection is the collection time of the first sample with a positive PCR test result or seroconversion without any prior reporting of symptoms that led to confirmation of a COVID-19 disease endpoint. The time of diagnosis of SARS-CoV-2 seroconversion is the collection time of the first sample seropositive for SARS-CoV-2, for participants SARS-CoV-2 seronegative at baseline; the SAP will specify criteria for defining seroconversion (re-infection) for individuals SARS-CoV-2 seropositive at baseline. The failure times of participants never observed to be diagnosed with a given clinical endpoint will be right-censored at the date of last contact at which endpoint status was assessed or study termination. The failure times of participants reporting receipt of COVID-19 vaccination outside the study will be right-censored at the date of first outside vaccination.

6.4.3.1 Primary VE Analysis

For the primary analysis, Cox proportional hazards regression will be used to estimate vaccine efficacy against SARS-CoV-2 infection among baseline SARS-CoV-2 seronegative participants in the D29- set, counting only infection events diagnosed post-Day 29, as measured by one minus the hazard ratio (HR) in the immediate vs. delayed vaccination arms. To improve precision, the primary analysis will adjust for an SAP-specified set of “risk” variables, or a composite risk score based on them, that are potentially associated with SARS-CoV-2 exposure including demographics and behaviors captured using the risk

assessment tool. Inference will be based on a 2-sided score-based CI and 2-sided score-test for H0: VE = 0% with a type 1 error rate of 0.05, although primary interest is in estimation as opposed to hypothesis testing and the study is designed to ensure that the lower limit for the CI will lie above 30% with high probability under VE > 60%.The baseline hazard function in the Cox model will be stratified by study site and randomization stratum, which improves precision of the VE estimate and accounts for the fact that SARS-CoV-2 infection incidence may vary widely across study sites. Description of the risk variables under consideration and of the derivation of a risk score based on them will be described in the study SAP.

The potential impacts of the open-label aspect of the study design will be factored into the choice of efficacy analysis. If there is evidence of differential retention of study participants across arms, or differential adherence to study procedures affecting ascertainment of endpoints such as collection of daily swabs, the analysis will adjust for the differential censoring distribution across arms- as well as potentially informative censoring- as described in the study SAP. At a minimum, these analyses will be performed as sensitivity analyses, complementing the primary analysis described above.

Variables captured using the risk assessment tool and potentially associated with exposure will be compared across arms over study follow-up. If there is evidence that their distributions differ between arms over time, sensitivity analysis will additionally adjust for time-dependent exposure-related variables.

The proportional hazards assumption will be evaluated using methods of Grambsch and Therneau (51).

6.4.3.2 Secondary VE Analyses

The primary analysis is aimed at evaluating vaccine efficacy, i.e. the biological effect of the vaccine on SARS-CoV-2 infection. This parameter can be challenging to evaluate in any efficacy trial, given that with sufficient follow-up and heterogeneity in risk, there is culling of high-risk participants over time and the effect estimated using all follow-up is biased relative to the per-exposure reduction in risk of infection- although importantly the bias is small in the setting of low incidence with short follow-up and realistic heterogeneity assumptions (52). In secondary analyses, vaccine *effectiveness* will be evaluated- that is, the effect of the vaccine on SARS-CoV-2 infection, including both biological and behavioral effects caused by knowledge of vaccine receipt or lack of receipt. Vaccine effectiveness has public health importance, given that in public health practice individuals know whether and when they have received vaccine, and may modify their behavior given this knowledge. To evaluate vaccine effectiveness, the same primary analysis methods will be applied but without adjustment for covariates that are associated with SARS-CoV-2 exposure. Vaccine effectiveness will be evaluated against all the clinical endpoints, e.g. viral load, secondary transmission, COVID-19 disease, and seroconversion.

The primary analysis of VE against SARS-CoV-2 infection will be repeated among participants in the FAS, counting all SARS-CoV-2 infection events after Day 1; in the D29-set but including baseline seropositive participants [Secondary Objective 5]; and finally in the PP set [Secondary Objective 9]. The same methods also apply to evaluating VE against

seroconversion [Secondary Objective 2] and VE against COVID-19 disease [Secondary Objective 3]. Additional secondary VE analyses will be conducted where the time of SARS-CoV-2 infection diagnosis is defined as the earliest of the first positive SARS-CoV-2 test result based on either PCR testing of nasal swabs or external SARS-CoV-2 testing.

While the primary analysis of VE against SARS-CoV-2 infection will censor participants who receive outside COVID-19 vaccination at the time of first outside vaccination, a secondary analysis will be performed that includes infection events and person-time that accrue following outside vaccination with the Moderna COVID-19 Vaccine. Participants receiving outside vaccination other than Moderna COVID-19 Vaccine will continue to be censored at the time of outside vaccination. SARS-CoV-2 infection events and person-time occurring early in the study – before Day 29 – will be included in this analysis. A time-varying Cox model will be used for inference, per methods in the study SAP.

A sensitivity analysis will be conducted that combines PCR testing of study swabs with outside test results to determine the endpoint of infection. Details will appear in the SAP. The asymptomatic SARS-CoV-2 infection endpoint [Secondary Endpoint 8] is defined by the PCR tests of daily swabs and serologic diagnostic testing schedule at month 0, 2, and 4. The occurrence of a COVID-19 disease event is a competing risk because the occurrence of COVID-19 disease precludes the possibility of occurrence of a subsequent asymptomatic SARS-CoV-2 infection event. Therefore, VE against asymptomatic SARS-CoV-2 infection will be assessed using methods accounting for COVID-19 disease as a competing risk. The SAP will provide details.

Vaccine efficacy against the infection and disease endpoints as measured by the reduction in the cumulative incidence of the endpoint by Month 4, will also be evaluated using the Nelson-Aalen estimator of cumulative incidence. This analysis does not rely on the proportional hazards assumption and has a simple interpretation that lends itself to understanding of population effects.

6.4.4 VL analyses

Primary analyses of the vaccine effect on VL will focus on the primary VL endpoint, peak log10 viral load, and will condition on primary endpoint event status. Peak log10 viral load will be estimated by the observed maximum log10 viral load measure; secondary VL analyses will employ parametric models to estimate VL at peak. The mean observed log10 viral load will be compared between the immediate and delayed vaccination arms using methods that adjust for baseline covariates and covariates at the time of SARS-CoV-2 infection diagnosis, per the study SAP. The covariate adjustment is used to control for potential post-randomization selection bias, whereby SARS-CoV-2 infected participants in the two treatment arms are not comparable in ways that are associated with the VL endpoint. Importantly, this bias occurs when there are factors that modify VE against SARS-CoV-2 infection *and* that are associated with the VL endpoint. Full details around the analysis plan will be specified in the study SAP.

Secondary analyses of vaccine effects on VL will consider a set of other summary measures of the VL curve, each of which have merits for further characterizing the vaccine effect.

These include a model-based estimate of peak log10 viral load, duration of detection of virus [estimated empirically or alternatively based on a model fit to the viral load curve], area under the log viral load curve [AUC] calculated using the trapezoidal rule, and area under the portion of the log viral load curve over 10^5 viral copies/mL. Some mathematical modeling studies provide evidence of transmission potential for VL above this threshold (21), but there is considerable uncertainty and therefore other thresholds will be considered. Analysis methods will be the same as for the primary VL endpoint above.

In addition, the above VL endpoints will be evaluated using unconditional analyses, ie, not conditioning on SARS-CoV-2 infection status, among all enrolled participants. Participants not diagnosed with SARS-CoV-2 infection will have an endpoint value defined for analysis, typically a value of zero. The 'transmission potential' endpoint [Secondary endpoint 4] is one such endpoint that is defined unconditionally. The merit of unconditional analyses is that they are not subject to potential post-randomization selection bias. A disadvantage of the unconditional analyses is that they may have lower statistical power.

An additional VL endpoint that will be studied is a censored event-time variable, time to VL above 10^5 copies/mL. This endpoint is defined for all enrolled participants and will be analyzed using the same methods used to evaluate VE against SARS-CoV-2 infection, but only counting infections once VL is above 10^5 copies/mL. Additional methods of analysis that allow for the uncertainty in the level of virus that is associated with transmission will be considered.

Sensitivity analyses for VL will include data from all SARS-CoV-2 infected participants diagnosed based on PCR testing of study swabs or based on outside testing.

The study SAP will detail the secondary analysis approaches for the VL endpoints.

6.4.5 Secondary transmission analyses

6.4.5.1 Identifying 'adjudicated transmission events'

An expert adjudication committee will examine and make a primary determination to identify 'adjudicated transmission events' from a main study participant diagnosed with infection ('index case') to a contact in the PCC or CACC diagnosed with SARS-CoV-2 infection. Note that the close contact could also be a main study participant in theory, although this is unlikely given the relatively small proportion of each community that will be recruited to the main study. The adjudication committee will operate under a charter that lays out a pre-specified set of criteria for defining the occurrence and direction of transmission and will be blinded to the treatment assignment of all study participants and contacts. The endpoint definition will include criteria that need to be met regarding the temporal relationship (eg, evidence that person B's infection occurred after person A's infection, evidence of compatible incubation period), epidemiologic link (evidence of close contact during person A's infectious period), and relatedness of their SARS-CoV-2 viruses.

The adjudication committee will be provided with all relevant data from the study to deduce transmission events from potential transmission pairs. A potential transmission pair is a post-

baseline SARS-CoV-2- infected main study index case and their identified contact(s) in the PCC or CACC who have positive SARS-CoV-2 test results within a prespecified period (eg, two weeks) of the index case diagnosis. This will include the type, frequency, and magnitude (eg, duration and setting) of contact between the index case and contact based on risk assessment questionnaires given to the index case and the contact; the timing of diagnosis of infection by PCR for the index case and the contact (defined as the date of specimen collection); the VL trajectories, serology, and symptomatology for the index case and the contact; and summaries of the viral sequences for the index case and the contact including the number of nucleotide differences in the sequences and any clustering and results of phylogenetic analyses (see Section 6.4.5.3). Adjudication of all potential transmission events will occur within a short time frame at the end of the study to ensure decisions are being made using the same background research on transmission and to ensure that the adjudication can take into account all SARS-CoV-2 infection events captured in the main study cohort and both contact cohorts. Primary analyses of the vaccine effect on secondary transmission will rely on the determination of this committee and use the adjudicated transmission events and outcomes in statistical models. These same primary analyses will also only include main study participants diagnosed with infection through PCR testing of study swabs. However, sensitivity analyses will incorporate data from the full set of main study participants diagnosed with infection either through outside testing or through PCR testing of swabs collected through the study per methods described in the SAP.

6.4.5.2 Analyses of the vaccine effect on secondary transmission

Primary analyses of vaccine efficacy against secondary transmission will include adjudicated transmission events collected from both the PCC and CACC. Both conditional and unconditional analyses will be performed. Conditional analyses will compare the expected number of adjudicated transmission events between index cases in the immediate vs. delayed vaccination arms using Poisson regression, where goodness of fit tests will be used to determine whether an overdispersed Poisson or other alternative distributions fit the data better, per the study SAP. The SAP will specify the baseline participant characteristics and measurements available at the time of primary endpoint diagnosis that are potentially associated with secondary transmission to be included as covariates. The conditional analysis may have greater power than an unconditional analysis, e.g. under low VE against SARS-CoV-2 infection. However, it is subject to the post-randomization selection bias, as discussed in relation to the viral load endpoint. In general, it will be biased if there are factors that modify VE against SARS-CoV-2 infection that are also associated with onward transmission; or if factors associated with onward transmission are modifiers of VE against infection.

A secondary analysis of this endpoint will measure vaccine efficacy against secondary transmission as the percent reduction in the expected number of adjudicated transmission events linked to a participant in the immediate vaccination arm relative to the delayed vaccination. In contrast to the primary transmission analysis that conditions on the main study participant being infected, all main study participants will contribute to the analysis including those who do not become infected to reflect the fact that SARS-CoV-2-uninfected participants do not transmit to others and therefore have 0 transmission events. Let N = number of adjudicated transmission events for a given index case which is assumed to follow a zero-inflated Poisson distribution with the mean of the non-zero point mass component is

$\mu e^{(V\gamma)}$, where V indicates randomization assignment (1 immediate vaccination; 0 delayed vaccination). Further let β denote the log hazard ratio from a Cox proportional hazards model for infection time. Then the ratio of the expected number of adjudicated transmission events linked to a participant in the immediate vaccination arm relative to the delayed vaccination can be expressed as

$$e^{\theta} = e^{\gamma} e^{\beta},$$

where θ is a measure of the vaccine's impact on total onward transmission (transmission from participants in main study) and e^{θ} is the relative reduction in the proportion of adjudicated transmission events in the immediate vaccination arm relative to the delayed vaccination arm. This is analogous to the product estimator in the proportional means model (53, 54). Note that this formulation assumes the time between detection in the index case participant and infection of a contact is ignorable. Covariate-adjusted approaches will be detailed in the SAP.

The unconditional analysis retains high power even if there are few breakthrough infections in the immediate vaccination group due to high VE against infection. It also properly reflects that a vaccine is successful at reducing secondary transmission if it either meaningfully reduces acquisition or reduces onward transmission from infected participants. Moreover, by avoiding conditioning on SARS-CoV-2 infection, it avoids the post-randomization selection bias issue.

While analyses that pool adjudicated transmission events from the PCC and CACC will produce valid tests of the null hypothesis of no vaccine effect on secondary transmission, likelihood-based analyses that take into account the different duration of follow-up of contacts in the PCC vs. the CACC, and the potential left-censoring of contact follow-up in the CACC will be explored and may provide greater accuracy and power (38). Details of these analyses, including methods for checking the underlying assumptions and investigations of their operating characteristics using simulations of social and transmission networks, will be provided in the study SAP.

6.4.5.3 Processing and phylogenetic analysis of SARS-CoV-2 viral sequence data

A central sequencing facility will obtain whole-genome sequences from all infected participants in the main study cohort and all infected participants in the PCC and CACC, based on the nasal swab with the highest viral load (see Section 10.3.2). Details about the sequencing platform and methodology for QC processes will be included in the study SAP. This will include the creation of a global alignment, which will be used for the isolation of genes and the sequence analyses of local regions.

A first step in the analysis of the sequence data will be to determine, for each pair of sequences among infected participants or contacts within a given study community, which are identical. Data from GenBank, Global Initiative on Sharing Avian Influenza Data (GSAID), and other public databases suggest that we can expect limited variability in genetic sequences, especially those from a single common ancestor and constrained within a single

study community over a 4-month time period. Consensus-based whole genomes that differ by more than two nucleotides can reasonably be ruled out as being transmission events.

A second step will be to conduct distance-based analyses of the sequences, including phylogenetic analysis, to identify potential lineages of infecting viral strains, infection clusters, and potentially linked transmissions. Phylogenetic analyses will also include 'background' SARS-CoV-2 sequences with geographical or temporal relevance from publicly available databases, such as GenBank and GISAID. The background sequences will help root the tree and give the tree structure and may lend insight into the identification of clusters and into potential transmission events during the adjudication process.

Details about the phylogenetic and other sequence analyses, including the tree-building methods and evolutionary models to be used, as well as approaches to incorporating data from multiple reads, will be available in the study SAP.

6.4.6 Immunogenicity analyses

6.4.6.1 Vaccine immunogenicity

Immunogenicity will be characterized for the immediate vaccination group only, using data from the Case-Cohort Immunogenicity Set (ccIS). Data from quantitative immunogenicity assays will be summarized using positive response rates and geometric means with 95% CIs, for each timepoint for which an assessment is performed. Data from qualitative (ie, yielding a positive or negative result) assays will be summarized by tabulating the frequency of positive responses for each assay at each timepoint that an assessment is performed. Analyses focus on the Month 2 time point, the Day 1 time point, and the change in marker response between the two time points. The analyses will evaluate immunogenicity in all immediate vaccination arm participants and separately in subgroups defined by baseline SARS-CoV-2 serostatus. The SAP will describe the complete set of immunogenicity analyses.

6.4.6.2 Immune response marker correlates of risk

A separate Trial Marker SAP will describe the statistical methods and data analysis implementations for assessing immune response markers as correlates of the SARS-CoV-2 infection and peak log viral load study endpoints, as well as various types of correlates/surrogates of protection.

An unblinded statistical analysis by treatment assignment of a primary immunogenicity endpoint may be performed when all participants have completed the Month 2 visit and data are available for analysis from at least 80% of these participants. Similarly, an unblinded statistical analysis by treatment assignment of a secondary or exploratory immunogenicity endpoint may be performed when all participants have completed the corresponding immunogenicity visit and data are available for analysis from at least 80% of these participants. The CoVPN Laboratory Center will review the analysis report prior to distribution to the protocol chairs, NIAID, study product developer, and other key CoVPN members and investigators. Reports for distribution or presentation should use PubIDs and

not PTIDs for individual responses. Distribution of reports will be limited to those with a need to know for the purpose of informing future trial-related decisions. The CoVPN leadership must approve any other requests for CoVPN immunogenicity analyses prior to the end of the scheduled follow-up visits.

7 Selection and withdrawal of participants

Participants will be generally healthy adults who comprehend the purpose of the study and have provided written informed consent. Volunteers will be recruited and screened; those determined to be eligible, based on the inclusion and exclusion criteria, will be enrolled in the study. Final eligibility determination will depend on information available at the time of enrollment, medical history questionnaire, physical examinations, and answers to self-administered and/or interview questions.

Investigators should always use good clinical judgment in considering a volunteer's overall fitness for trial participation. Some volunteers may not be appropriate for enrollment even if they meet all inclusion/exclusion criteria. Medical, psychiatric, occupational, or other conditions may make evaluation of safety and/or immunogenicity difficult, and some volunteers may be poor candidates for retention.

Determination of eligibility, taking into account all inclusion and exclusion criteria, must be made within 56 days prior to enrollment unless otherwise noted below.

7.1 Inclusion criteria for Main Cohort

General and Demographic Criteria

1. **Age** of 18 through 26 years.

2. **Enrollment in a post-secondary educational institution** and willingness to be followed for the planned duration of the study.

3. **Agrees to allow study staff to access school SARS-CoV-2 testing** data and outcomes, if applicable.

4. Ability and willingness to provide **informed consent.**

5. **Assessment of understanding**: volunteer demonstrates understanding of this study; completes a questionnaire prior to first vaccination with demonstration of understanding of all questionnaire items answered incorrectly.

6. **Willing to be randomized to either immediate or delayed vaccination group and comply with planned study procedures.**

7. **Agrees not to enroll in another study** of an investigational research agent until the end of the study.

8. **Access to device and internet** for Telehealth visits.

7.2 Exclusion criteria for Main Cohort

General

1. **Acutely ill 72 hours prior to or at screening**. Volunteers meeting this criterion may be rescheduled within the relevant window periods. Participants with minor illnesses can be enrolled at the discretion of the investigator.

2. **Blood products,** systemic immunoglobulins, or monoclonal antibodies (including against SARS-CoV-2) received within 90 days before first vaccination.

3. **Investigational research agents** received within 30 days before first vaccination.

4. **Self-reported known history of SARS-CoV-2 infection.**

Vaccines and other Injections

5. **Prior administration of a coronavirus** (SARS-CoV-2, SARS-CoV, MERS-CoV) vaccine or current/planned simultaneous participation in another interventional study to prevent or treat COVID-19.

Immune System

6. **Immunosuppressive medications** received within 168 days before first vaccination (not exclusionary: [1] corticosteroid nasal spray; [2] inhaled corticosteroids; [3] topical corticosteroids for mild, uncomplicated dermatologic condition; or [4] a single course of oral/parenteral prednisone or equivalent at doses $\leq$ 60 mg/day and length of therapy < 11 days with completion at least 30 days prior to enrollment).

Clinically significant medical conditions

7. **Clinically significant medical condition**, physical examination findings, or past medical history with clinically significant implications for current health. A clinically significant condition or process includes but is not limited to:

   - A process that would affect the immune response (well-controlled human immunodeficiency virus infection is allowed), or

   - Any condition specifically listed among the exclusion criteria below.

8. **Any medical, psychiatric, occupational, or other condition** that, in the judgment of the investigator, would interfere with, or serve as a contraindication to, protocol adherence, assessment of safety or reactogenicity, or a volunteer's ability to give informed consent.

9. **Bleeding disorder** (eg, factor deficiency, coagulopathy, or platelet disorder requiring special precautions).

10. **Asplenia**: any condition resulting in the absence of a functional spleen.

11. **History of angioedema or anaphylaxis**, including to vaccines or vaccine components (not exclusionary: angioedema or anaphylaxis with known trigger and no episodes within five years.).

12. **History of generalized urticaria** within past five years.

7.3 Inclusion criteria for Prospective Close Contact (PCC) cohort

1. **Age** of 18 years or older, at the time of signing the informed consent.

2. Willing and able to provide **informed consent**.

3. Expected to be in **frequent close physical proximity with Main Cohort participant** during the study.

4. **Willing to share results of SARS-CoV-2 testing**.

5. **Access to device and internet** for Telehealth visits.

7.4 Inclusion criteria for Case-ascertained Close Contact (CACC) cohort

1. **Age** of 18 years or older, at the time of signing the informed consent.

2. Willing and able to provide **informed consent**.

3. **Access to device and internet** for Telehealth visits.

4. Willing to share **results of SARS-CoV-2 testing**.

5. Had **close contact with Main Cohort participant with known PCR-confirmed SARS-CoV-2 infection** (eg, index case). Close contacts will have had exposure to the index participant, generally within 72 hours of an index diagnosis, and may include individuals that meet any of the following guidelines:

   - Prolonged close physical proximity with Main Cohort participant within a residence/vehicle/enclosed space without maintaining social distance,

   - Medical staff, first responders, or other care persons who cared for the index case without appropriate personal protective equipment.

   Further information on the definition of close contact can be found in the CoVPN 3006 Study Specific Procedures (SSP).

8 Study product

The protocol schema is shown in Table 1-1.

8.1 Vaccine regimen

Group 1

Treatment 1 (T1): Moderna COVID-19 Vaccine in 100 mcg dose given as 0.5 ml IM into the deltoid muscle on Day 1 and Day 29.

Group 2

Treatment 2 (T2): Moderna COVID-19 Vaccine in 100 mcg dose given as 0.5 ml IM into the deltoid muscle on Day 113 and Day 141.

8.2 Storage, handling, preparation, and administration

Vaccination providers will refer to EUA vaccine instructions accessed and updated at https://www.modernatx.com/covid19vaccine-eua/.

8.3 Acquisition of Moderna COVID-19 Vaccine

Emergency use authorized Moderna COVID-19 Vaccine will be provided by Moderna, Inc. through the US Government COVID-19 Vaccine Response and allocated for this trial.

9 Clinical procedures

Procedures are in place so that study visits may be conducted remotely, such as via phone, text message, email, or other electronic means, in lieu of, or in combination with, in-person visits at the clinical research site. Furthermore, some visit procedures may be conducted outside the CRS (see CoVPN 3006 SSP for additional details).

The schedules of clinical procedures are shown in Appendix D, Appendix E, Appendix F and Appendix G.

9.1 Informed consent

Informed consent is the process of working with participants so that they fully understand what will and may happen to them while participating in a research study. The CoVPN informed consent form documents that a participant (1) has been informed about the potential risks, benefits, and alternatives to participation, and (2) is willing to participate in a CoVPN study. Informed consent encompasses all written, verbal, and electronic study information CRS staff provide to the participant, before and during the trial. CRS staff will obtain informed consent of participants according to the CoVPN 3006 SSP.

If any new information is learned that might affect the participants' decisions to stay in the trial, this information will be shared with trial participants. If necessary, participants will be asked to provide revised informed consent forms.

9.1.1 Screening consent form

Without a general screening consent, screening for a specific study cannot take place until the site receives protocol registration from NIAID or its designee.

Some CRSs have approval from their IRB/EC and any applicable RE to use a general screening consent form that allows screening for an unspecified SARS-CoV-2 vaccine trial. In this way, CRS staff can continually screen potential participants and, when needed, proceed quickly to obtain protocol-specific enrollment consent. Sites conducting general screening or prescreening approved by their IRB/EC and any applicable RE may use the results from this screening to determine eligibility for this protocol, provided the tests are conducted within the time periods specified in the eligibility criteria.

9.1.2 Protocol-specific consent forms

The protocol-specific consent forms describe the study products to be used and all aspects of protocol participation, including screening and enrollment procedures. A sample protocol-specific consent form for the Main cohort is located in Appendix A. A sample protocol-specific consent form for the PCC cohort is located in Appendix B. A sample protocol-specific consent form for the CACC cohort is located in Appendix C.

Each CRS is responsible for developing a protocol-specific consent form(s) for local use, based on the sample protocol-specific consent forms. The consent form(s) must be developed in accordance with requirements of the following:

- CRS's IRB/EC and any applicable REs
- CRS's institution
- Elements of informed consent as described in Title 45, CFR Part 46 and Title 21 CFR, Part 50, and in ICH E6(R2), Good Clinical Practice: Consolidated Guidance 4.8

Study sites are strongly encouraged to have local community representatives from the target population review their sites-specific consent forms. This review should include, but should not be limited to, issues of cultural competence, local language considerations, and the level of understandability.

The sample informed consent form(s) include(s) instructions for developing specific content.

9.1.3 Assessment of Understanding

Study staff are responsible for ensuring that participants fully understand the study before enrolling them. This process involves reviewing the informed consent form with the participant, allowing time for the participant to reflect on the procedures and issues presented, and answering all questions completely.

An Assessment of Understanding is used to document the participant's understanding of key concepts in this study. Participants must demonstrate understanding of all questions answered incorrectly. This process and the participant's understanding of the key concepts is to be recorded in the eConsent.

9.2 Pre-enrollment procedures for Main Cohort, Immediate and Delayed Vaccination Groups

Screening may occur over the course of several contacts/visits, up to and including before randomization on Day 1. All inclusion and exclusion criteria must be assessed within 56 days before enrollment, unless otherwise specified in the eligibility criteria (or below in this section). Screening results will be reviewed with the volunteer.

After the appropriate informed consent has been obtained and before enrollment, the following procedures are performed:

- Participant will download the electronic diary (eDiary) application to their smartphone or tablet and complete an optional application training. Participant will use eDiary to enter data. Data collection will include:
    - Medical history questionnaire

  - Volunteer demographics in compliance with the NIH Policy on Reporting Race and Ethnicity Data: Subjects in Clinical Research, Aug. 8, 2001 (available at http://grants.nih.gov/grants/guide/notice-files/NOT-OD-01-053.html)
  - Baseline SARS-CoV-2 infection risk information

- Discussion of the eDiary entries done by the participant with the site staff
- Review of materials for possible self-collection of blood by the participant

9.3 Enrollment and vaccination visits for Main Cohort, Immediate Vaccination Group

Once a volunteer has consented to trial participation and is found to meet all eligibility criteria (see Sections 7.1 and 7.2), the CRS requests the randomization assignment via a Web-based randomization system. **Enrollment is simultaneous with randomization**. In general, the time interval between randomization and completion of all study activities for Visit 2 should not exceed approximately 7 working days. However, circumstances may require a participant's visit to be changed. This may exceed the 7-day randomization time limit recommendation.

At vaccination visits, the following procedures are performed **before vaccination**:

- COVID-19 symptom check (including temperature), both vaccination visits
- Specimen collection (**only at first vaccination** visit, see Appendix D)

Following completion of all procedures in the preceding list, and if results indicate that vaccination may proceed, vaccination is prepared and administered per Moderna EUA Fact Sheet and Full Prescribing Information for Vaccination Providers that can be accessed at https://www.modernatx.com/covid19vaccine-eua/).

Additionally, the participants will be:

- Reminded to enter information in eDiary throughout the study
- Provided with a supply of nasal swabs and shown how to swab both nostrils

9.4 Follow-up procedures for Main Cohort, Immediate Vaccination Group

The following procedures are performed **during follow-up per schedule in** Appendix D:

- Participants reminded to enter information in eDiary throughout the study (see Section 9.16)

- Nasal swab collection by participant
- Blood collection by CRS staff, phlebotomist, or participant

9.5 Enrollment (visit 2) and visit 4 (Month 2) for Main Cohort, Delayed Vaccination Group

Once a volunteer has consented to trial participation and is found to meet all eligibility criteria (see Sections 7.1 and 7.2), the CRS requests the randomization assignment via a Web-based randomization system. **Enrollment is simultaneous with randomization**. In general, the time interval between randomization and completion of all study activities for Visit 2 should not exceed approximately 7 working days. However, circumstances may require a participant's visit to be changed. This may exceed the 7-day randomization time limit recommendation.

Before leaving the clinic, the participants will be:

- Reminded to enter information in eDiary throughout the study
- Provided with a supply of nasal swabs and shown how to swab both nostrils

At visits 2 and 4, the following procedures are performed **per schedule in** Appendix E:

- eDiary entries by participant (see Section 9.16)
- Nasal swab collection by participant
- Blood collection by CRS staff, phlebotomist, or participant

9.6 Vaccination visits for Main Cohort, Delayed Vaccination Group

At vaccination visits, the following procedures are performed **before vaccination**:

- COVID-19 symptom check (including temperature)
- Specimen collection (**only at first vaccination** visit, see Appendix E)

Following completion of all procedures in the preceding list, and if results indicate that vaccination may proceed, vaccination is prepared and administered, per Moderna EUA Fact Sheet and Full Prescribing Information for Vaccination Providers that can be accessed at https://www.modernatx.com/covid19vaccine-eua/).

9.7 Procedures for Main Cohort participant diagnosed with a SARS-CoV-2 infection during the study

If a participant is diagnosed with a SARS-CoV-2 infection during the course of the study, CDC guidelines on administration of vaccination will be followed.

Main Cohort participants who are clinically diagnosed with SARS-CoV-2 infection (from any test result indicative of acute infection) are referred to as clinically diagnosed index cases. They will be instructed to isolate per local guidelines and the following procedures will be performed:

- eDiary collection of daily symptoms of COVID-19 and assessment of whether clinical care was sought or hospitalization occurred (via self-report and/or request for medical records) through an eDiary for a period of 14 days or until symptoms have resolved, whichever is longer
- Daily nasal swabbing continued per schedule of procedures (see Appendix D)
- Blood collection by phlebotomist or participant for SARS-CoV-2 clinical serology
- Close contacts identification (see Section 9.9)

Following resolution of the isolation period, participants will continue scheduled study visits. Follow-up duration for participants diagnosed with moderate or severe COVID-19 disease may be adjusted in consultation with the CRS (eg, to avoid interference with participant initiation of SARS-CoV-2 treatment).

9.8 Enrollment and follow-up procedures for Prospective Close Contact (PCC) cohort

Upon enrollment, participants from the Main Cohort will be asked to invite individuals with whom they will be in frequent close physical proximity during the study to join the PCC cohort (See Section 7.3 and CoVPN 3006 SSP for additional information on PCC cohort). New PCC contacts may also be added during the study.

Prospective close contacts will be given referral codes and information about how to enroll in the study as part of the PCC cohort. If they choose to enroll and sign an informed consent, they will be asked to:

- provide access to their routine SARS-CoV-2 testing reports, if applicable
- enter data weekly through eDiary (see Section 9.16)

If a PCC cohort participant or the Main Cohort participant to whom he/she is connected is diagnosed with a SARS-CoV-2 infection during the course of the study, the PCC cohort participant will be instructed to perform the following procedures (see Appendix F):

- eDiary collection of daily symptoms of COVID-19 and assessment of whether clinical care was sought or hospitalization occurred (via self-report and/or request for medical records) through an eDiary for a period of 14 days or until symptoms have resolved, whichever is longer

- provide nasal swab for 14 days

- self-collect blood samples on day 1 and day 29, relative to the notification of infection

For Main Cohort participants who are also PCC cohort participants related to an index case in another Main cohort participant, the following procedures in addition to those listed in Appendix D may be performed:

- one additional blood collection (self-collection) as soon as possible following the report of the index case

- eDiary collection of information regarding exposure to the Index case

- eDiary collection of daily symptoms of COVID-19 and assessment of whether clinical care was sought or hospitalization occurred (via self-report and/or request for medical records) through an eDiary for a period of 14 days or until symptoms have resolved, whichever is longer

See CoVPN 3006 SSP for additional information on PCC cohort.

9.9 Enrollment and Follow-up procedures for Case-Ascertained Close Contact (CACC) cohort

Participants from the Main Cohort who are clinically diagnosed with SARS-CoV-2 (referred to as the Index case) during the study will be asked to contact any individuals with whom they have been in close contact within about 72 hours of the date of the SARS-CoV-2 diagnosis test. See Section 7.4 and CoVPN 3006 SSP for additional information on case-ascertained close contacts.

Potential CACC cohort participants will be given referral codes and information about how to enroll in the study as part of the CACC cohort, if they choose to, and sign an informed consent.

Once a volunteer has consented to be a part of CACC cohort, the following procedures will be performed (see Appendix G):

- eDiary collection of information regarding exposure to the Index case

- eDiary collection of daily symptoms of COVID-19 and assessment of whether clinical care was sought or hospitalization occurred (via self-report and/or request for medical

records) through an eDiary for a period of 14 days or until symptoms have resolved, whichever is longer.

- Outcomes of SARS-CoV-2 testing performed by the school or other facilities
- Nasal swab self-collection from day 1-14
- Blood collection by participant for SARS-CoV-2 clinical serology at day 3 and at day 29

If a CACC participant is diagnosed with a SARS-CoV-2 infection during the course of the study, they will be instructed to track daily symptoms of COVID-19 through an eDiary for a period of 14 days or until symptoms have resolved, whichever is longer.

For Main Cohort participants who are also CACC cohort participants, the following procedures in addition to those listed in Appendix D may be performed:

- one additional blood collection (self-collection) as soon as possible
- eDiary collection of information regarding exposure to the Index case
- eDiary collection of daily symptoms of COVID-19 and assessment of whether clinical care was sought or hospitalization (via self-report and/or request for medical records) occurred through an eDiary for a period of 14 days or until symptoms have resolved, whichever is longer.

9.10 Visit windows and missed visits

Visit windows are included in Appendix H. The procedures for documenting missed visits and out of window visits are described in CoVPN 3006 SSP.

9.11 Discontinuing vaccination for a participant who receives a COVID-19 Vaccine outside the study

In the event a participant receives an EUA or licensed COVID-19 vaccine outside the study, an individual participant's study vaccinations will be permanently discontinued. Participants should be counseled on the importance of continuing with the study and strongly encouraged to participate in follow-up visits and protocol-related procedures per the protocol for the remainder of the trial, unless medically contraindicated.

9.12 Early termination visit

In the event of early participant termination, site staff should consider if final specimen collection is appropriate.

9.13 Pregnancy for Main Cohort

For Main cohort participants who have been vaccinated, pregnancies and pregnancy outcomes will be reported via the FDA/CDC Vaccine Adverse Event Reporting System (VAERS) (see Section 11.1).

9.14 SARS-CoV-2 monitoring by schools for Main Cohort

For participating schools that have SARS-CoV-2 monitoring plans in place, monitoring will typically provide approximately twice weekly SARS-CoV-2 testing for enrolled Main Cohort participants. For any positive test result, the institution will inform the student (initiating isolation), will provide the SARS-CoV-2 test results to the students, and will carry out contact tracing. Participants will be instructed to report positive test results via the eDiary.

9.15 COVID-19 symptom surveillance for Main Cohort

Participants will be monitored for symptoms of COVID-19 throughout the course of the study via a weekly eDiary. These symptoms will be used to establish whether a participant meets criteria for COVID-19 disease as defined by Secondary Endpoint 4. Due to the overlap of symptoms that can result from vaccination and symptoms of COVID-19, monitoring will begin 7 days following the first study product administration. Symptom monitoring will be paused 7 days following the second product administration. If a participant has symptoms consistent with COVID-19, they will be encouraged to seek testing and clinical care through their local testing and care facilities. Results of testing will be reported through the eDiary and participants who have positive test results will follow procedures described in Section 9.7.

9.16 Use of electronic Diary (eDiary) for Clinical Outcomes Assessments

At the time of consent, participants must confirm they will be willing to complete an eDiary collection of study data (see below for details of data to be collected).

The eDiary will be completed using an application downloaded to their smartphone or tablet. This study will utilize the Medidata Patient Cloud Application as the eDiary software. This application allows for real-time data collection directly from participants.

After participants have signed the informed consent, all participants will be instructed to download the eDiary application on their personal smartphone or tablet. The eDiary application has a self-training component, enabling immediate use. The site staff will perform any retraining as necessary.

For Main Cohort participants,

Study participants will record data in the eDiary throughout the study as described in Appendix D and Appendix E, following the prompts provided for data collection. eDiary data collection areas may include:

- Medical history questionnaire
- Volunteer demographics
- Risk assessment for SARS-CoV-2 infection (see Appendix D and Appendix E)
- Surveillance for symptoms of COVID-19 (see Section 9.15)
- Self-collection of samples (eg, nasal swabs) (see Appendix D and Appendix E)
- Outcomes of SARS-CoV-2 testing performed by the school or other facilities (see Appendix D and Appendix E)
- COVID-19 symptom tracking upon incident diagnosis of SARS-CoV-2 infection, including assessments of whether clinical care was sought or hospitalization occurred via self-report and/or request for medical records (see Section 9.7)

Additionally, participants will be reminded through the eDiary of the need to continue practices for reducing acquisition and transmission of SARS-Cov-2 regardless of their vaccination status.

For Prospective Close Contact (PCC) cohort participants,

PCC participants record data in the eDiary as described in Appendix F, following the prompts provided for data collection. eDiary data collection areas may include:

- Collection of demographic information
- Collection of information regarding exposure to the main study participant
- Outcomes of SARS-CoV-2 testing performed by the school or other facilities
- Self-collection of samples (eg, nasal swabs) (see Appendix F)
- COVID-19 symptom tracking upon incident diagnosis of SARS-CoV-2 infection in PCC or main study participant, including assessments of whether clinical care was sought or hospitalization occurred via self-report and/or request for medical records
- Recording of any COVID-19 vaccines received outside of the study

For Case-Ascertained Close Contact (CACC) cohort participants,

CACC participants will record data in the eDiary as described in Appendix G, following the prompts provided for data collection. eDiary data collection areas may include:

- Collection of demographic information
- Collection of information regarding exposure to the Index case
- Outcomes of SARS-CoV-2 testing performed by the school or other facilities
- COVID-19 symptom tracking, including assessments of whether clinical care was sought or hospitalization occurred via self-report and/or request for medical records
- Self-collection of samples (eg, nasal swabs) (see Appendix G)
- Recording of any COVID-19 vaccines received outside of the study

10 Laboratory

10.1 CRS laboratory procedures

The CoVPN 3006 SSP and other study materials provide further guidelines for operational issues concerning the clinics and laboratories. These documents include special considerations for phlebotomy and guidelines for general specimen collection, specimen labeling and specimen processing.

Tube types for blood collection are specified in CoVPN 3006 SSP.

Of note, all assays described below, with the exception of SARS-CoV-2 PCR, are performed as research assays to evaluate the ability of the SARS-CoV-2 virus to induce immune responses in the context of the participants' genetic background and are not approved for use in medical care. Results from these research assays are not made available to participants or medical professionals to guide treatment decisions.

10.2 Total blood volume

Required blood collections per visit are shown in Appendix D, Appendix E, Appendix F and Appendix G. The total blood volume drawn for each participant will not exceed 500 mL in any 56-day (8-week) period.

10.3 Endpoint assays

10.3.1 SARS-CoV-2 PCR

Real-time reverse transcription polymerase chain reaction (RT-PCR) assays to detect SARS-CoV-2 will be run on nasal samples collected from study participants. After RNA extraction, specimens will be analyzed by a laboratory developed test using the World Health Organization's E/RdRp primer set, the N1/N2 primer set from the Centers for Disease Control and Prevention, or tests from Hologic (Panther Fusion), DiaSorin (Simplexa), or Roche (cobas). Novel methods to detect SARS-CoV-2 may also be used if applicable.

10.3.2 SARS-CoV-2 viral sequencing

Genome sequencing of SARS-CoV-2 virus may be performed on nasal samples from study participants using a metagenomic approach. RNA from positive specimens is converted to a cDNA library by reverse transcription and the library is sequenced. Resulting consensus sequences are assembled against a SARS-CoV-2 reference genome.

10.3.3 Neutralizing antibody assay (nAb)

SARS-CoV-2–specific nAb assays may be performed on serum samples from study participants. The assay will test neutralization of S-pseudotyped and/or full-genome recombinant viruses as measured by a reduction in luciferase (Luc) reported gene expression after infection in ACE-2 positive cells.

10.3.4 Binding antibody multiplex assay (BAMA)

SARS-CoV-2 specific total IgG and IgM binding antibodies may be assessed in serum samples. In addition, SARS-CoV-2–specific serum IgA and IgG subclass (IgG1, IgG2, IgG3, and IgG4) antibodies may also be assessed. Epitope specificity by ACE-2 blocking assay and responses to endemic coronavirus proteins may also be assessed.

10.3.5 Antibody avidity (BioLayer Interferometry and BAMA avidity index)

SARS-CoV-2-specific polyclonal serum antibody and IgG subclass avidity may be measured using BAMA with the addition of a dissociation step to calculate the antibody avidity index (BAMA-AI). BLI and/or SPR technologies may also be used to measure antibody avidity.

10.3.6 Fc-Mediated Antibody Function

Additional assays to measure SARS-CoV-2-specific antibody Fc functions may be performed (ie, antibody dependent cellular cytotoxicity [ADCC], antibody-dependent cellular phagocytosis [ADCP], antibody-dependent neutrophil phagocytosis [ADNP], binding antibody FcR array, and S-protein-Expressing Cell Antibody Binding Assay [SECABA]).

10.4 Lab assay portfolio

Additional assays may be performed per the Laboratory Center assay portfolio, which includes immune assessments such as those for cellular, humoral, and innate immune responses, and host genetics. The assay portfolio will be updated periodically to include new assays and adjust qualification levels of existing assays.

10.5 Exploratory studies

Samples may be used for other testing and research related to furthering the understanding of SARS-CoV-2 immunology, virology, or vaccines. In addition, cryopreserved samples may be used to perform additional assays to support standardization and validation of existing or newly developed methods.

10.6 Specimen storage and other use of specimens

The CoVPN stores specimens from all study participants indefinitely, unless a participant requests that specimens be destroyed or if required by IRB/EC, or RE.

Other use of specimens is defined as studies not covered by the protocol or the informed consent forms for the study (see Appendix A, Appendix B, and Appendix C).

This research may relate to SARS-CoV-2, vaccines, coronaviruses, the immune system, and other diseases. This could include genetic testing and, potentially, genome-wide studies. This research is done only to the extent authorized in each study site's informed consent form, or as otherwise authorized under applicable law. Other research on specimens ("other use") will occur only after review and approval by the CoVPN, the IRB/EC of the researcher requesting the specimens, and the IRBs/ECs/REs of the CRSs if required.

As part of consenting for the study, participants document their initial decision to allow or not allow their specimens to be used in other research, and they may change their decision at any time. The participant's initial decision about other use of their specimens, and any later change to that decision, is recorded by their CRS in a Web-based tool that documents their current decisions for other use of their specimens. The CoVPN will only allow other research to be done on specimens from participants who allow such use.

CRSs must notify CoVPN Regulatory Affairs if institutional or local governmental requirements pose a conflict with or impose restrictions on specimen storage or other use of specimens.

10.7 Biohazard containment

The transmission of blood-borne pathogens can occur through contact with contaminated needles, blood, and blood products, and the transmission of SARS-CoV-2 and other respiratory pathogens may occur through contact with contaminated respiratory droplets, aerosols, and other biological materials. Appropriate precautions will be employed by all personnel in the collection, shipping, and handling of all specimens for this study, as currently recommended by local health authorities, the CDC, the NIH, or other applicable agencies.

All dangerous goods materials, including Biological Substances, Category A or Category B, must be transported according to instructions detailed in the International Air Transport Association Dangerous Goods Regulations.

11 Safety Reporting

11.1 FDA/CDC Vaccine Adverse Event Reporting System (VAERS)

The Vaccine Adverse Event Reporting System (VAERS) is co-managed by the U.S. CDC and FDA and serves as the reporting mechanism for adverse events occurring from licensed and EUA vaccines, including the Moderna COVID-19 vaccine being used in this trial. VAERS accepts and analyzes reports of adverse events (AEs) after a person has received a vaccination. It is accessed at https://vaers.hhs.gov/reportevent.html.

Risks associated with receipt of the Moderna COVID-19 Vaccine and AE reporting requirements are described in the “Fact Sheet For Healthcare Providers Administering Vaccine (Vaccination Providers) Emergency Use Authorization (EUA) Of The Moderna COVID-19 Vaccine To Prevent Coronavirus Disease 2019 (COVID-19)”, available at https://www.modernatx.com/covid19vaccine-eua/.

All vaccination providers, including those at the CRS, are required to report to VAERS the following adverse events after COVID-19 vaccination, under Emergency Use Authorization (EUA), and other adverse events if later revised by CDC |(https://www.cdc.gov/vaccines/covid-19/vaccination-provider-support.html and https://vaers.hhs.gov/faq.html):

- Vaccine administration errors, whether or not associated with an adverse event (AE)
- Serious AEs regardless of causality. Serious AEs per FDA are defined as:
    - Death;
    - A life-threatening AE;
    - Inpatient hospitalization or prolongation of existing hospitalization;
    - A persistent or significant incapacity or substantial disruption of the ability to conduct normal life functions;
    - A congenital anomaly/birth defect;
    - An important medical event that based on appropriate medical judgement may jeopardize the individual and may require medical or surgical intervention to prevent one of the outcomes listed above.
- Cases of Multisystem Inflammatory Syndrome
- Cases of COVID-19 that result in hospitalization or death

CRS vaccination providers are encouraged to report to VAERS any additional clinically significant AEs following vaccination, even if they are not sure if vaccination caused the event; and are to report any additional select AEs and/or any revised safety reporting

requirements per FDA's conditions of authorized use of vaccine(s) throughout the duration of any COVID-19 Vaccine being authorized under an EUA.

CRS vaccination providers will complete and submit reports to VAERS online at https://vaers.hhs.gov/reportevent.html. Further assistance with reporting to VAERS is accessed by calling 1-800-822-7967. The reports should include the words "Moderna COVID- 19 Vaccine EUA" in the description section of the report.

As indicated in the "Fact Sheet For Healthcare Providers Administering Vaccine (Vaccination Providers) Emergency Use Authorization (EUA) Of The Moderna COVID-19 Vaccine To Prevent Coronavirus Disease 2019 (COVID-19)", vaccination providers (including those at the CRS) are encouraged, to the extent feasible, to report adverse events to Moderna.

11.2 Reporting safety events to v-safe

An additional source of VAERS reports will be through a program administered by the CDC known as v-safe (https://www.cdc.gov/coronavirus/2019-ncov/vaccines/safety/vsafe.html). V-safe is a smartphone-based opt-in program that uses text messaging and web surveys from CDC to check in with vaccine recipients for health problems following COVID-19 vaccination. The system also will provide telephone follow-up to anyone who reports medically significant (important) adverse events. Responses indicating missed work, inability to do normal daily activities, or that the recipient received care from a doctor or other healthcare professional will trigger the VAERS Call Center to reach out to the participant and collect information for a VAERS report, if appropriate. CRS vaccination providers will encourage the study participants receiving the Moderna COVID-19 vaccine to use v-safe.

12 Protocol conduct

This protocol and all actions and activities connected with it will be conducted in compliance with the principles of GCP (ICH E6 (R2)), and according to NIAID and CoVPN policies and procedures as specified in the CoVPN 3006 SSP*,* NIAID Clinical Research Policies and Standard Procedures Documents including procedures for the following:

- Protocol registration, activation, and implementation;
- Informed consent, screening, and enrollment;
- Study participant reimbursement;
- Clinical and safety assessments;
- Safety monitoring and reporting;
- Data collection, documentation, transfer, and storage;
- Participant confidentiality;
- Study follow-up and close-out;
- Unblinding of staff and participants;
- Quality control;
- Protocol monitoring and compliance;
- Specimen collection, processing, and analysis;
- Exploratory and ancillary studies and sub-studies, and
- Destruction of specimens.

Any policies or procedures that vary from NIAID and CoVPN standards or require additional instructions (eg, instructions for randomization specific to this study) will be described in the CoVPN 3006 SSP.

12.1 Study termination

NIAID reserves the right to terminate or curtail a clinical study for any reason, including but not limited to the following (reference: https://grants.nih.gov/grants/guide/rfa-files/RFA-AI-12-012.html):

- risk to subject safety
- the scientific question is no longer relevant or the objectives will not be met (ie, slow accrual)
- failure to comply with GCP, U.S. Federal regulations, or Terms and Conditions of Award
- occurrence of unforeseen drug safety issues or data from preclinical studies indicate a presence of unanticipated toxicity
- risks that cannot be adequately quantified
- ethical concerns raised by the local community or local medical care/health care authorities
- failure to remedy deficiencies identified through site monitoring
- substandard data
- reaching a major study endpoint substantially before schedule with persuasive statistical significance.

This study may also be terminated early by the determination of the FDA, the United States Department of Health and Human Services Office for Human Research Protections (OHRP). In addition, the conduct of this study at an individual CRS may be terminated by the determination of the IRB/EC and any applicable RE.

12.2 Emergency communication with study participants

As in all clinical research, this study may generate a need to reach participants quickly to avoid imminent harm, or to report study findings that may otherwise concern their health or welfare and their willingness to remain on study.

When such communication is needed, the CRS will request that its IRB/EC and any applicable RE expedite review of the message. If this review cannot be completed in a timeframe consistent with the urgency of the required communication, the site can contact the participant without IRB/EC approval if such communication is necessary to avoid imminent harm to the study participant. The CRS must notify the IRB/EC and any applicable RE of the matter as soon as possible.

13 Version history

The Protocol Team may modify the original version of the protocol. Modifications are made to CoVPN protocols via clarification memos, letters of amendment, or full protocol amendments.

The version history of, and modifications to, Protocol CoVPN 3006 are described below.

Protocol history and modifications

Date: April 12, 2021

Protocol version: 3.0
Protocol modification: Full protocol amendment 2

Item 1 Added in Section 1, *Overview* and Section 6, *Statistical considerations*: Clinical Research Sites (CRSs) that can participate in the study expanded to include non-university affiliated CoVPN Clinical Research Sites (CRSs)

Item 2 Revised in Section 1, *Overview*, Section 2, *Background*, Section 6, *Statistical considerations*, Section 7.1, *Inclusion criteria for main cohort*, Section 9, *Clinical procedures*, and Appendix A, *Sample informed consent form for the Main cohort*: definition of participant expanded to include students enrolled in any post-secondary educational institution

Item 3 Revised in Section 1, *Overview*, Section 2, *Background*, Section 6, *Statistical considerations,* Section 7.3*, Inclusion criteria for Prospective Close Contact (PCC) cohort,* Section 9*, Clinical procedures,* and Appendices A, B, D, E, F: University SARS-CoV-2 testing is not required

Item 4 Revised in Section 9.3*, Enrollment and vaccination visits for Main Cohort, Immediate Vaccination Group*, Section 9.5, *Enrollment (visit 2) and visit 4 (Month 2) for Main Cohort, Delayed Vaccination Group* and Appendix H, *Visit windows*: maximum recommended time between randomization and completion of Visit 2 activities increased from 4 days to 7 days

Item 5 Clarified in Section 2, *Background*, Section 6, *Statistical considerations*, and Section 9.7, *Procedures for Main Cohort participant diagnosed with a SARS-CoV-2 infection during the study*: any SARS-CoV-2 test result indicative of acute infection (on or off study) obtained from a Main cohort participant will trigger “index-case” activities

Item 6 Clarified in Section 2, *Background*, Section 6.4.3, *Vaccine efficacy (VE) analyses against SARS-CoV-2 infection*, Section 7.1, *Inclusion criteria for main cohort*, Section 9, *Clinical procedures*, Appendix A, *Sample informed consent form for Main cohort*, and Appendix B, *Sample informed consent form for Prospective Close Contact (PCC) cohort*: participants can choose to obtain an EUA or licensed COVID-19 vaccine outside the study

Item 7 Added to Section 3, *Objectives and endpoints*: 2 endpoints for the duration of viral shedding specified in corresponding secondary objective 4

Item 8 Clarified in Section 6, *Statistical considerations, 7th paragraph:* primary analysis occurs when participants complete 4 months of follow-up

Item 9 Clarified in Table 6-1, *Study analysis sets and definitions*: Day 29 is visit 3 for immediate vaccination arm and will be define in Statistical analysis plan (SAP) for delayed vaccination arm

Item 10 Revised in Section 6.3, *Randomization*: randomization evaluated within levels defined by study site and type of residence

Item 11 Clarified in Section 6.4.3.1, *Primary VE Analysis*: rationale for choice of analysis

Item 12 Revised in Section 6.4.5.2, *Analyses of the vaccine effect on secondary transmission*: details of the derivation

Item 13 Revised in Section 7.2, Exclusion criterion #1 for Main Cohort: removed febrile assessment

Item 14 Clarified in Section 2.10, *Moderna COVID-19 Vaccine*, and Section 8.3, *Acquisition of Moderna COVID-19 Vaccine*: Moderna COVID-19 Vaccine provided under EUA and allocated for this trial

Item 15 Corrected in Section 9, *Clinical procedures*: CRS staff will obtain informed consent of participants according to the COVPN 3006 SSP

Item 16 Corrected in Appendix A, *Sample informed consent form for Main cohort:* blood will be collected 3 times for delayed vaccination arm

Item 17 Added to Appendix B, *Sample informed consent form for Prospective Close Contact (PCC)* cohort: COVID-19 care is not paid for by the study

Item 18 Added to Appendices A, B, C, *Sample informed consent forms*: information regarding employee/family member participation

Item 19 Added to Appendices A, B, C, *Sample informed consent forms*: terms of use for data collection via web-based app

Item 20 Deleted in Appendix B, *Sample informed consent form for Prospective Close Contact (PCC)* cohort, and Appendix C, Sample informed consent form for Case-Ascertained Close Contact (CACC) cohort, item 8: text referring to vaccine receipt

Item 21 Added to Appendix H, *Visit windows for CACC:* upper allowable window for blood specimen collection

Item 22 Added changes per Protocol v 2.0, Letter of Amendment 1

Item 23 Updated Title page and Section 13, *Version history*: contents of this amendment

Item 24 Updated Section 1.1, *Protocol team*: deletion of Site Principal Investigator representative

Item 25 Throughout the protocol: minor editorial changes, clarifications, and corrections

Date: March 10, 2021

Protocol version: Version 2.0
Protocol modification: Letter of Amendment 1

Item 1 Added in Section 10.1, *CRS laboratory procedures*, Appendices A, B, and C, *Sample informed consent forms*: return of SARS-CoV-2 nasal swab testing results to participants

Item 2 Added to Section 1.1, *Protocol team*: Jasmine Marcelin, co-chair and Jeffrey L Carson, Site Principal Investigator representative

Date: February 26, 2021

Protocol version: 2.0
Protocol modification: Full protocol amendment 1

Item 1 Revised in Section 3, *Objective and Endpoints,* Section 6, *Statistical considerations,* Section 9, *Clinical procedures*, Appendix A, *Sample informed consent form for the main cohort*, and Appendices D and E, *Procedures for the main cohort*: Month 3 visit removed for both Main cohort groups, M1 visit removed for delayed vaccination group of Main cohort, and Month 1 blood collection removed for immediate vaccination group of Main cohort

Item 2 Added in Section 1, *Overview*: 2 new endpoint laboratories

Item 3 Updated in Section 1.1, *Protocol team*: team members

Item 4 Deleted Section 6.1, *Accrual restrictions*: no accrual restrictions for this study

Item 5 Revised in Section 6.4, *Statistical analyses*: minor clarifying text revisions

Item 6 Corrected in Section 6.4.4.7, *VL analyses,* 3rd paragraph: reference to secondary endpoint number

Item 7 Deleted in Section 9.3, *Enrollment and vaccination visits for Main Cohort, Immediate Vaccination Group* and Section 9.5, *Enrollment (visit 2) and visit 4 (Month 2) for Main Cohort, Delayed Vaccination Group:* sentence regarding clinical lab results

Item 8 Added in Section 9.15, *Use of electronic Diary (eDiary) for Clinical Outcomes Assessments,* and Appendices F through G, *Procedures*: end of study questionnaire regarding COVID vaccines received outside the study

Item 9 Added in Appendices A, B, and C, *Sample informed consent forms*, section “Being in the study”: participants will take an end of study questionnaire

Item 10 Added in Appendix B, *Sample informed consent form for Prospective Close Contact (PCC) cohort*, and Appendix C, *Sample informed consent form for Case-Ascertained Close Contact (CACC) cohort*, section "Being in the study": applicable participants will receive SARS-CoV-2 screening at the University about two times per week.

Item 11 Deleted in Appendices A, B, and C, *Sample informed consents,* second sentence*:* reference to alternate study name of "vPROTECT"

Item 12 Added in Appendix A, *Sample informed consent form for the main cohort:* additional information for participant regarding text or email reminders for nasal swabs

Item 13 Deleted in Appendices A, B and C, *Sample informed consents:* reference to "electronically" for signature

Item 14 Deleted in Appendices A, B and C, *Sample informed consents*, signature table: column for denoting time of signature

Item 15 Deleted in Appendix A, *Sample informed consent form for the main cohort*, section 8: redundant information

Item 16 Corrected in in Appendix A, *Sample informed consent form for the main cohort*, section10: VAERS reporting requirements

Item 17 Revised in Appendices F and G: table text and associated footnotes

Item 18 Revised Appendix H, *Visit windows,* Case-Ascertained Close Contact (CACC) cohort, to include applicable Prospective Close Contact (PCC) participants who test positive for SARS-CoV-2 or whose Main Cohort study contact tests positive for SARS-CoV-2

Item 19 Revised throughout protocol: minor grammatical, editorial, and formatting corrections.

Item 20 Updated Title page, Table of Contents, Section 13, *Version history*, and Appendix I*, Protocol signature page*: dates, version number, and contents of this amendment

Date: February 17, 2021

Protocol version:1.0

Protocol modification: Not applicable

Original protocol

14 Document references (other than literature citations)

Other documents referred to in this protocol, and containing information relevant to the conduct of this study, include:

- Assessment of Understanding. Accessible through the CoVPN protocol-specific website.
- CDC COVID-19 Vaccination Program Provider Requirements and Support. Available at https://www.cdc.gov/vaccines/covid-19/vaccination-provider-support.html
- CoVPN Certificate of Confidentiality. Accessible through the CoVPN website.
- CoVPN 3006 Study Specific Procedures. Accessible through the CoVPN protocol-specific website.
- Dangerous Goods Regulations (updated annually), International Air Transport Association. Available for purchase at https://www.iata.org/publications/dgr/Pages/index.aspx
- International Council on Harmonisation of Technical Requirements for Pharmaceuticals for Human Use (ICH) E6 (R2), Guideline for Good Clinical Practice: Section 4.8, Informed consent of trial subjects. Available at http://www.ich.org/page/efficacy-guidelines
- Laboratory Center assay portfolio
- Moderna COVID-19 Vaccine EUA Fact Sheet and Full PI for Vaccination Providers. Available at https://www.modernatx.com/covid19vaccine-eua/.
- Participants' Bill of Rights and Responsibilities. Accessible through the CoVPN protocol-specific website.
- NIH Policy on Reporting Race and Ethnicity Data: Subjects in Clinical Research. Available at https://grants.nih.gov/grants/guide/notice-files/NOT-OD-01-053.html
- Title 21, Code of Federal Regulations, Part 50. Available at http://www.accessdata.fda.gov/scripts/cdrh/cfdocs/cfcfr/CFRSearch.cfm?CFRPart=50
- Title 45, Code of Federal Regulations, Part 46 (2018 requirements). Current requirements available at https://www.hhs.gov/ohrp/regulations-and-policy/regulations/45-cfr-46/index.html
- VAERS, Vaccine Adverse Event Reporting System. Available at https://vaers.hhs.gov/reportevent.html

- VAERS, Vaccine Adverse Event Reporting System FAQ. Available at https://vaers.hhs.gov/faq.html

- V-safe After Vaccination Health Checker. Available at https://www.cdc.gov/coronavirus/2019-ncov/vaccines/safety/vsafe.html

See Section 16 for literature cited in the background and statistics sections of this protocol.

15 Acronyms and abbreviations

Ab	antibody
ACIP	Advisory Committee on Immunization Practices
AE	adverse event
AUC	area under the curve
BAMA	binding antibody multiplex assay
CAB	Community Advisory Board
CDC	US Centers for Disease Control and Prevention
CFR	Code of Federal Regulations
CI	confidence intervals
COVID-19	Coronavirus disease 2019
CoVPN	COVID-19 Prevention Network
CRF	case report form
CRPMC	NIAID Clinical Research Products Management Center
CRS	clinical research site
DAIDS	Division of AIDS (US NIH)
DHHS	US Department of Health and Human Services
EAE	adverse events requiring expedited reporting
EC	Ethics Committee
eDiary	Electronic diary
ELISA	enzyme-linked immunosorbent assay
EUA	emergency use authorization
FAS	Full analysis set
FDA	US Food and Drug Administration
Fred Hutch	Fred Hutchinson Cancer Research Center
GCP	Good Clinical Practice
GMT	geometric mean titer
HIPAA	Health Insurance Portability and Accountability Act
HIV	human immunodeficiency virus
IB	Investigator's Brochure
IBC	Institutional Biosafety Committee
ICH	International Council on Harmonisation of Technical Requirements for Pharmaceuticals for Human Use
IQR	Interquartile range
IDMC	Independent Data Monitoring Committee
IM	Intramuscular
IRB	Institutional Review Board
LC	Laboratory Center

LNP	Lipid nanoparticle
LOC	Leadership and Operations Center
mRNA	Messenger ribonucleic acid
nAb	neutralizing antibody
NHP	nonhuman primate
NIAID	National Institute of Allergy and Infectious Diseases (US NIH)
NIH	US National Institutes of Health
OHRP	US Office for Human Research Protections
PCR	polymerase chain reaction
PI	Principal Investigator
RE	regulatory entity
SAP	Statistical Analysis Plan
SCHARP	Statistical Center for HIV/AIDS Research and Prevention
SDMC	statistical and data management center
SSP	Study specific procedures
SOP	Standard operating procedure
UW-VL	University of Washington Virology Laboratory
UW-VSL	University of Washington Virology Specialty Laboratory
VAERS	Vaccine Adverse Event Reporting System
VE	Vaccine efficacy
VE_I	Vaccine efficacy against infectiousness
VEs	Vaccine efficacy against susceptibility
VL	Viral load

16 Literature cited

1. Johns Hopkins Coronavirus Resource Center. 2020 [Available from: https://coronavirus.jhu.edu/map.html.

2. Lipsitch M, Dean NE. Understanding COVID-19 vaccine efficacy. Science. 2020.

3. Mehrotra DV, Janes HE, Fleming TR, Annunziato PW, Neuzil KM, Carpp LN, et al. Clinical Endpoints for Evaluating Efficacy in COVID-19 Vaccine Trials. Ann Intern Med. 2020.

4. Long QX, Tang XJ, Shi QL, Li Q, Deng HJ, Yuan J, et al. Clinical and immunological assessment of asymptomatic SARS-CoV-2 infections. Nat Med. 2020;26(8):1200-4.

5. Salvatore PP, Sula E, Coyle JP, Caruso E, Smith AR, Levine RS, et al. Recent Increase in COVID-19 Cases Reported Among Adults Aged 18-22 Years - United States, May 31-September 5, 2020. MMWR Morb Mortal Wkly Rep. 2020;69(39):1419-24.

6. Boehmer TK, DeVies J, Caruso E, van Santen KL, Tang S, Black CL, et al. Changing Age Distribution of the COVID-19 Pandemic - United States, May-August 2020. MMWR Morb Mortal Wkly Rep. 2020;69(39):1404-9.

7. CDC: Centers for Disease Control and Prevention. COVID-19 Hospitalization and Death by Age 2020 [Available from: https://www.cdc.gov/coronavirus/2019-ncov/covid-data/investigations-discovery/hospitalization-death-by-age.html.

8. Halloran ME, Longini IM, Jr., Struchiner CJ. Design and interpretation of vaccine field studies. Epidemiol Rev. 1999;21(1):73-88.

9. Swan DA, Goyal A, Bracis C, Moore M, Krantz E, Brown E, et al. Vaccines that prevent SARS-CoV-2 transmission may prevent or dampen a spring wave of COVID-19 cases and deaths in 2021. medRxiv. 2020.

10. Swan DA, Bracis C, Janes H, Moore M, Matrajt L, Reeves DB, et al. COVID-19 vaccines that reduce symptoms but do not block infection need higher coverage and faster rollout to achieve population impact. medRxiv. 2020.

11. Datta S, Halloran ME, Longini IM, Jr. Efficiency of estimating vaccine efficacy for susceptibility and infectiousness: randomization by individual versus household. Biometrics. 1999;55(3):792-8.

12. Milani GP, Dioni L, Favero C, Cantone L, Macchi C, Delbue S, et al. Serological follow-up of SARS-CoV-2 asymptomatic subjects. Sci Rep. 2020;10(1):20048.

13. Piccoli L, Park YJ, Tortorici MA, Czudnochowski N, Walls AC, Beltramello M, et al. Mapping Neutralizing and Immunodominant Sites on the SARS-CoV-2 Spike Receptor-

Binding Domain by Structure-Guided High-Resolution Serology. Cell. 2020;183(4):1024-42 e21.

14. Vaccines and Related Biological Products Advisory Committee Meeting. Sponsor Briefing Document, Moderna COVID-19 Vaccine 2020 [Available from: https://www.fda.gov/media/144452/download.

15. Vaccines and Related Biological Products Advisory Committee Meeting. Sponsor Briefing Document Addendum,Moderna COVID-19 Vaccine 2020 [Available from: https://www.fda.gov/media/144453/download.

16. Flood S, King M, Rodgers R, Ruggles S, Warren J. Integrated Public Use Microdata Series, Current Population Survey: version 7.0 [dataset]. 2020.

17. The New York Times. Tracking the Coronavirus at U.S. Colleges and Universities 2020 [Available from: https://www.nytimes.com/interactive/2020/us/covid-college-cases-tracker.html.

18. Business Insider. The 10 biggest coronavirus outbreaks on college campuses across the US since the fall semester began 2020 [Available from: https://www.businessinsider.com/10-colleges-with-fall-coronavirus-outbreaks-1200-cases-or-more-2020-9.

19. Wilson E, Donovan CV, Campbell M, Chai T, Pittman K, Sena AC, et al. Multiple COVID-19 Clusters on a University Campus - North Carolina, August 2020. MMWR Morb Mortal Wkly Rep. 2020;69(39):1416-8.

20. Buitrago-Garcia D, Egli-Gany D, Counotte MJ, Hossmann S, Imeri H, Ipekci AM, et al. Occurrence and transmission potential of asymptomatic and presymptomatic SARS-CoV-2 infections: A living systematic review and meta-analysis. PLoS Med. 2020;17(9):e1003346.

21. Goyal A, Reeves DB, Cardozo-Ojeda EF, Schiffer JT, Mayer BT. Wrong person, place and time: viral load and contact network structure predict SARS-CoV-2 transmission and super-spreading events. medRxiv. 2020.

22. He X, Lau EHY, Wu P, Deng X, Wang J, Hao X, et al. Temporal dynamics in viral shedding and transmissibility of COVID-19. Nat Med. 2020;26(5):672-5.

23. Tindale LC, Stockdale JE, Coombe M, Garlock ES, Lau WYV, Saraswat M, et al. Evidence for transmission of COVID-19 prior to symptom onset. Elife. 2020;9.

24. Kissler SM, Fauver JR, Mack C, Tai C, Shiue KY, Kalinich CC, et al. Viral dynamics of SARS-CoV-2 infection and the predictive value of repeat testing. medRxiv. 2020.

25. CDC: Centers for Disease Control and Prevention. Scientific Brief: Community Use of Cloth Masks to Control the Spread of SARS-CoV-2 2020 [Available from: https://www.cdc.gov/coronavirus/2019-ncov/more/masking-science-sars-cov2.html.

26. Jung CY, Park H, Kim DW, Choi YJ, Kim SW, Chang TI. Clinical Characteristics of Asymptomatic Patients with COVID-19: A Nationwide Cohort Study in South Korea. Int J Infect Dis. 2020;99:266-8.

27. Poteat T, Millett GA, Nelson LE, Beyrer C. Understanding COVID-19 risks and vulnerabilities among black communities in America: the lethal force of syndemics. Ann Epidemiol. 2020;47:1-3.

28. Davies NG, Klepac P, Liu Y, Prem K, Jit M, group CC-w, et al. Age-dependent effects in the transmission and control of COVID-19 epidemics. Nat Med. 2020;26(8):1205-11.

29. Richmond CS, Sabin AP, Jobe DA, Lovrich SD, Kenny PA. SARS-CoV-2 sequencing reveals rapid transmission from college student clusters resulting in morbidity and deaths in vulnerable populations. medRxiv. 2020.

30. Regeneron Pharmaceuticals. Regeneron's REGN-CoV2 antibody cocktail reduced viral levels and improved symptoms in non-hospitalized COVID-19 patients 2020 [Available from: https://newsroom.regeneron.com/news-releases/news-release-details/regenerons-regn-cov2-antibody-cocktail-reduced-viral-levels-and.

31. Chen P, Nirula A, Heller B, Gottlieb RL, Boscia J, Morris J, et al. SARS-CoV-2 Neutralizing Antibody LY-CoV555 in Outpatients with Covid-19. N Engl J Med. 2020.

32. Heneghan C, Brassey J, Jefferson T. SARS-CoV-2 viral load and the severity of COVID-19. The Centre for Evidence-Based Medicine. 2020.

33. Pujadas E, Chaudhry F, McBride R, Richter F, Zhao S, Wajnberg A, et al. SARS-CoV-2 viral load predicts COVID-19 mortality. Lancet Respir Med. 2020;8(9):e70.

34. Westblade LF, Brar G, Pinheiro LC, Paidoussis D, Rajan M, Martin P, et al. SARS-CoV-2 Viral Load Predicts Mortality in Patients with and without Cancer Who Are Hospitalized with COVID-19. Cancer Cell. 2020;38(5):661-71 e2.

35. Letizia AG, Ramos I, Obla A, Goforth C, Weir DL, Ge Y, et al. SARS-CoV-2 Transmission among Marine Recruits during Quarantine. N Engl J Med. 2020.

36. Datta S, Halloran ME, Longini IM, Jr. Augmented HIV vaccine trial design for estimating reduction in infectiousness and protective efficacy. Stat Med. 1998;17(2):185-200.

37. Halloran ME, Struchiner CJ. Causal inference in infectious diseases. Epidemiology. 1995;6(2):142-51.

38. Yang Y, Longini IM, Jr., Halloran ME. Design and Evaluation of Prophylactic Interventions Using Infectious Disease Incidence Data from Close Contact Groups. J R Stat Soc Ser C Appl Stat. 2006;55(3):317-30.

39. Klick B, Leung GM, Cowling BJ. Optimal design of studies of influenza transmission in households. I: case-ascertained studies. Epidemiol Infect. 2012;140(1):106-14.

40. Barnabas RV, Brown ER, Bershteyn A, Stankiewicz Karita HC, Johnston C, Thorpe LE, et al. Hydroxychloroquine as Postexposure Prophylaxis to Prevent Severe Acute Respiratory Syndrome Coronavirus 2 Infection : A Randomized Trial. Ann Intern Med. 2020.

41. Jackson LA, Anderson EJ, Rouphael NG, Roberts PC, Makhene M, Coler RN, et al. An mRNA Vaccine against SARS-CoV-2 - Preliminary Report. N Engl J Med. 2020.

42. Anderson EJ, Rouphael NG, Widge AT, Jackson LA, Roberts PC, Makhene M, et al. Safety and Immunogenicity of SARS-CoV-2 mRNA-1273 Vaccine in Older Adults. N Engl J Med. 2020;383(25):2427-38.

43. Vaccines and Related Biological Products Advisory Committee Meeting. FDA Briefing Document, Moderna COVID-19 Vaccine 2020 [Available from: https://www.fda.gov/media/144434/download.

44. UNAIDS. Ethical considerations in biomedical HIV prevention trials. 2007 7/2007.

45. The National Commission for the Protection of Human Subjects of Biomedical and Behavioral Research. The Belmont Report: Ethical Principles and Guidelines for the Protection of Human Subjects of Research. 1979 4/18/1979.

46. Council for International Organizations of Medical Sciences (CIOMS). International ethical guidelines for biomedical research involving human subjects. Bull Med Ethics. 2002(182):17-23.

47. Sun K, Wang W, Gao L, Wang Y, Luo K, Ren L, et al. Transmission heterogeneities, kinetics, and controllability of SARS-CoV-2. Science. 2021;371(6526).

48. Li F, Li YY, Liu MJ, Fang LQ, Dean NE, Wong GWK, et al. Household transmission of SARS-CoV-2 and risk factors for susceptibility and infectivity in Wuhan: a retrospective observational study. Lancet Infect Dis. 2021.

49. Madewell ZJ, Yang Y, Longini IM, Jr, Halloran ME, Dean NE. Household Transmission of SARS-CoV-2: A Systematic Review and Meta-analysis. JAMA Network Open. 2020;3(12):e2031756-e.

50. Kissler SM, Fauver JR, Mack C, Olesen SW, Tai C, Shiue KY, et al. SARS-CoV-2 viral dynamics in acute infections. medRxiv. 2020.

51. Grambsch PM, Therneau TM. Proportional Hazards Tests and Diagnostics Based on Weighted Residuals. Biometrika. 1994;81(3):515-26.

52. Hernan MA. The hazards of hazard ratios. Epidemiology. 2010;21(1):13-5.

53. Follmann D, Huang CY. Incorporating founder virus information in vaccine field trials. Biometrics. 2015;71(2):386-96.

54. Lin DY. Proportional means regression for censored medical costs. Biometrics. 2000;56(3):775-8.

Appendix A Sample informed consent form for Main cohort

**Sponsor / Study Title:**	**National Institutes of Health, "A randomized controlled study to assess SARS CoV-2 infection, viral shedding, and subsequent potential transmission in university students immunized with Moderna COVID-19 Vaccine"**
**Protocol Number:**	**CoVPN 3006**
**Principal Investigator: (Study Doctor)**	**«PiFullName»**
**Telephone:**	**«IcfPhoneNumber»**
**Address:**	**«PiLocations»**

Thank you for your interest in our research study. It is called CoVPN 3006. This informed consent form will tell you more about the study. Please read it carefully as you decide if you want to join. If you have questions, please ask us. At the end, we will ask you to answer a few questions to make sure you understand the study.

If you decide to join this study, we will ask you to sign this form. We will offer you a copy of this form to keep.

CoVPN 3006 is a research study. Research is not the same as medical care. The purpose of a research study is to answer scientific questions. We hope that what we learn will help people in the future.

Key information

- Joining this research study is voluntary. It is your choice.
- Our scientific questions are: Does the vaccine protect people from getting infected with a coronavirus called SARS-CoV-2? Does the vaccine prevent people from transmitting SARS-CoV-2 to others?
- If you join, your participation in this study will last for about 5 months.

- If you join, we will ask you to answer questionnaires, get injections, give blood, and take daily swabs of your nose.

  Here are the risks of taking part:

- The most common risk is symptoms such as muscle aches or headaches after getting the injection.
- There is a risk of loss of your personal information.
- There are other, less serious risks. We will tell you more about them later in this consent form.

About the study

The COVID-19 Prevention Network (CoVPN) is doing a study to test a vaccine against SARS-CoV-2. SARS-CoV-2 is the virus that causes the disease called COVID-19. We want to know if the vaccine is able to protect people from getting infected and if getting the vaccine will affect the amount of virus that is in your nose. We also want to know if getting the vaccine will prevent transmitting SARS-CoV-2 to others.

Many people will take part in this study. About 12,000 people will get vaccine injections while about 24,000 will be in the Prospective Close Contact (PCC) group and up to about 1500 more will be in the Case-ascertained Close Contact (CACC) group. These close contact groups will help researchers determine if the vaccine given to you prevents transmission of the SARS-CoV-2 virus to others. We will explain more about this later in this form.

We are asking you to join the Main part of the study in which people get vaccine injections. People who join the Main study will be recruited from schools in the United States (US). The researcher in charge of this study at this clinic is [Insert name of site PI]. The US National Institutes of Health is paying for this study.

1. **This study's vaccine has an Emergency Use Authorization (EUA) for individuals 18 and older.**

The vaccine we will use in this study is called the Moderna COVID-19 Vaccine. The vaccine has been given to over 15,000 people in other research studies and millions more people under EUA. We will give you the most up-to-date "Fact Sheet for Recipients and Caregivers" with more information about the Moderna COVID-19 Vaccine. The person giving you the vaccine can answer any questions you might have about what is in this fact sheet.

We already know that the vaccine can prevent serious COVID-19 disease, but we do not know if the vaccine will work to prevent SARS-CoV-2 infection in others. That is what we are testing in this study.

The Moderna COVID-19 Vaccine is not approved for sale in the United States by the US Food and Drug Administration (FDA).

Although the vaccine is not approved, it has an Emergency Use Authorization (EUA) from the FDA based on its success at preventing disease symptoms from SARS-CoV-2 in studies to date. During a public health emergency, like the COVID-19 pandemic, the FDA can issue an EUA to get vaccines to people faster. When the FDA issues an EUA, they continue to monitor safety.

The vaccine was developed by ModernaTX, Inc. Typical vaccines for viruses are made from a weakened or killed virus, but the Moderna COVID-19 Vaccine is not made from the SARS-CoV-2 virus. The vaccine includes a short segment of messenger ribonucleic acid (mRNA). The mRNA is a genetic code that tells cells how to make a protein. This mRNA is made in a laboratory. When injected into the body, the mRNA vaccine causes some cells to make that viral protein, which can trigger the immune system. Your immune system protects you from disease. If a person is later infected, their immune system remembers the protein from the vaccine which may help it to fight the virus. It is impossible for the vaccine to give you SARS-CoV-2 or COVID-19 infection.

Risks of the Moderna COVID-19 Vaccine:

To date, millions of people have already received the Moderna COVID-19 vaccine. In clinical trials, most people who got the vaccine had some reaction after their injections, especially after the second injection. The reaction to the vaccine didn’t affect their daily lives and it went away after two or three days. Most of these people said they had pain in the arm where they got the injection. These people also felt tired, had headaches, muscle and joint pain, and chills. A much smaller number of these people said they had redness or swelling where the needle went in their arm.

The risks of the study vaccine seen so far are the same as what have been seen with most vaccines. Generally, vaccines can cause fever, chills, rash, aches and pains, nausea, headache, dizziness, and feeling tired. Vaccines can also cause pain, redness, swelling, or itching where you got the injection. Most people can still do their planned activities after getting a vaccine. Rarely, people have side effects that limit their normal activities or make them go to the doctor.

Rarely, a vaccine can cause you to have an allergic reaction. You might have a rash, hives, or trouble breathing. Allergic reactions can be life-threatening. Tell us if you have ever had a bad reaction to any injection or vaccine.

There may be other risks that we don’t yet know about, even serious ones. We will tell you if we learn about any new risks.

Joining the study

2. **You will receive the vaccine immediately or later.**

   Not everyone in this study will get the vaccine immediately. Some people will get the study vaccine 4 months later than the first group of people in the Main study.

   We will compare the results from people who got the vaccine immediately with results from people who got the vaccine later. In this way, we will be able to tell if the vaccine can prevent SARS-CoV-2 infection.

   You have a 50/50 chance of getting the vaccine immediately or later. Whether you get the vaccine immediately or a little later is completely random, like flipping a coin.

   We have no say in whether you get the vaccine immediately or later.

3. **It is completely up to you whether or not to join this study.**

   Take your time in deciding. If it helps, talk to people you trust, such as your doctor, friends, or family. If you decide not to join this study, or if you leave it after you have joined, it will not change your healthcare or your standing at your school. If you do join, you do not give up your legal rights.

   You cannot be in this study while you are in another study where you get a study product.

   If you choose not to join this study, you may be able to join another study.

   If you are an employee or relative of an employee of a participating school, you are under no obligation to participate in this study. You may withdraw from the study at any time and for any reason, and your decision will not have any effect on your/your family member's performance appraisal or employment at the school. You may refuse to participate or you may withdraw from the study at any time without penalty or anyone blaming you.

   If you are a student, your participation will not place you in good favor with the study doctor or other faculty (for example, receiving better grades, recommendations, employment). Also, not participating in this study will not adversely affect your relationship with the study doctor or other faculty.

4. **If you want to join this study, after you sign this consent, you will download an electronic diary (eDiary) app and take questionnaires in it.**

   We will send you information about how to download the eDiary app onto your phone or tablet. We can help you download the app and show you how to use it.

To find out if you are eligible to join the study, you will answer questionnaires in the eDiary. Some of the questions will ask about your health history, what medications you take, your age, race, and ethnicity, and where you live. Your answers will tell us about some aspects of your health and if you may be eligible to join the study. It should only take about 10 minutes to complete the questionnaires.

Your answers to the questions may show you are not eligible to join the study, even if you want to. We might contact you to ask some more questions to further evaluate whether you can join the study.

In order to use the app you will be asked to agree to the Terms of Use and Privacy Policy which will appear on your mobile device's screen when you first start using the app. If you decide that you do not want to agree, then you should not participate in the research. While using the app, data about you including personal health information, other communication data, and internet usage will be collected and transmitted to the researchers and to the app developer. A complete description of this data collection and sharing is found in the Privacy Policy. Transmission of information via the internet is not completely secure, so there is a small risk of unintentional release of your information and safeguards are in place to protect your personal information. While the Terms of Use may include statements limiting your rights if you are harmed in this study, you do not release the investigator, sponsor, institution, or agents from responsibility for mistakes, and these statements do not apply to the use of the app in this research study.

Being in the study

If you want to join and you are eligible, here is what will happen:

5. **You will be in the study about 5 months.**

Visits can be done in-person or remotely depending on the type of procedures that need to be done. Visits where you get injections must be done in person. Remote visits will be done whenever possible to reduce potential SARS-CoV-2 exposure.

You may have to come to the clinic if you have a lab or health issue.

Site: Insert visit lengths.

Visits can last from [#] to [#] hours.

At your first visit, we will give you nasal swab kits and instructions so that you can take daily swabs of your nose. Once you receive your first kit, you will swab your nose every day, and you will record it in the eDiary for the entire study. You will receive reminders by text message or email each day to do your swabs. In all, you will swab both of your nostrils every day for about 4 months. You can ask us questions if you need help. You will return your swabs using drop-off location(s) specified by the clinic or by mail.

If you are in the immediate vaccination group, we will take blood samples in the clinic at your first injection visit and 2 more times, two months and four months later. When we take blood, the amount will depend on the lab tests we need to do. Each time we take your blood, the total amount we will take will not be more than two tablespoons. If for some reason you cannot come to the clinic, you may be asked to self-collect blood using a small device called the Tasso-SST OnDemand. Each time you collect your own blood, the amount will be less than one teaspoon. We will give you everything you need to take the blood and return it to us. We will be looking at how your body responds to the vaccine and if you been exposed to the SARS-CoV-2 virus.

If you are in the later vaccination group, we will take blood samples in the clinic at the first visit, and 2 more times, two months and four months later. Each time we take your blood, the total amount we will take will not be more than one teaspoon. If for some reason you cannot come to the clinic, you may be asked to self-collect blood using a small device called the Tasso-SST OnDemand. Each time you collect your own blood, the amount will be less than one teaspoon. We will give you everything you need to take the blood and return it to us. We will be looking to see if you been exposed to the SARS-CoV-2 virus.

The self-collection device called the Tasso-SST OnDemand sticks to the skin with a light adhesive. When the button is pressed, a vacuum forms and the device will prick the surface of the skin. The vacuum draws blood out of the capillaries and into a sample pod attached to the bottom of the device.

You will also take a daily questionnaire in the eDiary that asks about the results of your SARS-CoV-2 tests taken at school or another location. The daily questionnaires will take about 5 minutes to complete. You will also take an End of Study questionnaire in the eDiary. The eDiary will send your phone or tablet a push notification or you will receive a reminder email.

6. **We will give you the vaccine on a schedule.**

You will be in one of 2 groups: The Immediate Vaccination group or the Later Vaccination group. Regardless of which group you are in, you will get 1 injection into your upper arm at 2 separate visits during the study.

We will ask you to sign up for v-safe to report any reactions to the study vaccine. V-safe is a smartphone-based tool from the Center for Disease Control (CDC) that uses text messaging and web surveys to check in with you after your COVID-19 vaccination.

**Procedures for Immediate Vaccination group**	**Screening visit(s)**	**First injection visit**	**1st month**	**2nd month**	**4th month**
Injection		√	√		
Medical history questionnaire	√	√			
SARS-CoV-2 screening at school, if applicable		About 2x per week			
eDiary app	√	Daily through month 4			
Blood drawn		√		√	√
Nasal swab		Daily through month 4			

**Procedures for Later Vaccination group**	**Screening visit(s)**	**1st Day**	**2nd month**	**First injection visit (4th month)**	**5th month**
Injection				√	√
Medical history questionnaire	√	√			
SARS-CoV-2 screening at school, if applicable		About 2x per week			
eDiary app	√	Daily through month 4			√
Blood drawn		√	√	√	
Nasal swab		Daily through month 4			

7. **We will compensate you for your participation.**

Site: Insert your compensation plan here, e.g. money for number of swabs returned. Remember to include any costs to participants.

8. **In addition to giving you the vaccine:**

We will ask you to give a referral code to people with whom you expect to be in frequent close physical proximity during the study, like a roommate or co-worker. We want to ask these people to enroll in another part of this study that will help us find out if the vaccine

prevents transmission of the SARS-CoV-2 virus. We are calling this group the Prospective Close Contact group or PCC.

As part of their study participation, they will be asked to complete a weekly eDiary that includes questionnaires like the ones you completed. If you become infected with SARS-CoV-2, we will ask you to share your status with your PCCs. Then your PCC participants will be asked to fill out additional questionnaires for 2 weeks as well as take nasal swabs and self-collect blood samples.

When considering who to ask to be your PCC, you should think about whether you feel comfortable or safe sharing your infection status with them if you get SARS-CoV-2. If you don't feel comfortable sharing this information with them, you should not ask them to participate as a PCC.

It is possible that someone you know may also be participating in the Main part of the study and might identify you as a close contact. They will not know you are also participating in the study unless you choose to tell them. If someone else identifies you as a close contact, they will give you their referral code. It is okay to be enrolled in both the Main part of the study and the PCC group.

We may also ask you for the name of someone who can tell us how you are doing if we can't reach you or if you are unable to talk. We may contact you after the main study ends (for example, to tell you about the study results).

We will ask you to continue to follow any school program for SARS-CoV-2 testing and contact tracing, if your school offers one. If you test positive for SARS-CoV-2, based on any SARS-CoV-2 testing you receive, please tell us as soon as possible.

9. **The CoVPN will test your samples to see how your body, including your immune system, responds to the Moderna COVID-19 vaccine.**

We will send your samples to labs approved by the CoVPN. Your samples will not be labeled with your name or other identifying information.

Researchers may also do genetic testing related to this study on your samples. Your genes are passed to you from your birth parents. Differences in people's genes can help explain why some people get a disease while others do not. The genetic testing will only involve some of your genes, not all of your genes (your genome). The researchers will study only the genes related to the immune system and coronavirus, and genes that may affect how people get coronavirus.

If you get SARS-CoV-2, the researchers may look at all of the virus' genes that are in your samples. The researchers will use this information to learn more about SARS-CoV-2 and how the virus is impacted by the study vaccine.

The tests the researchers do on your samples are for research purposes, not to check your health. However, the nasal swab testing you do as part of this study may tell us if you are SARS-CoV-2 positive. Nasal swab test results from the study will be provided to the sites and be made available to you. You may be asked to get a confirmatory test. For all other lab tests, we will not provide the results to you or the site.

When your samples are no longer needed for this study, the CoVPN will continue to store them.

10. **We will do our best to protect your private information.**

US sites: Check Health Insurance Portability and Accountability Act (HIPAA) authorization for conflicts with this section.

We will keep your study records and samples in a secure location. We will label all of your samples and most of your records with a code number, not your name or other personal information. We will not share your name with anyone who does not need to know it.

Your records may also be reviewed by groups who watch over this study. These groups include:

- Study monitors
  - The CoVPN, people who work for it, and companies that help it with this study
  - Some government agencies:
    - The US National Institutes of Health
    - The US Office for Human Research Protections
    - Any regulatory agency that reviews research studies
  - Some committees that make sure we protect your rights and keep you safe:
    - The Independent Data Monitoring Committee
    - Advarra Institutional Review Board (IRB)
    - *Sites may, but are not required to include:* [Insert name of local IRB/EC]
    - *Sites include:* [Insert name of local IBC]

All reviewers will take steps to keep your records private.

We cannot guarantee absolute privacy. If you are found to have a medical condition that we are required to report by law, then some of your information may be shared.

At this clinic, we have to report the following information:

Site: Include any public health or legal reporting requirements. Bulleted examples should include all appropriate cases (reportable communicable disease, risk of harm to self or others, etc.)

- SARS-CoV-2
- Pneumonia
- After receiving the vaccine, we are required to report any of the following that may occur to the FDA/CDC Vaccine Adverse Event Reporting System (VAERS):
  - Vaccine administration errors
  - Serious reaction after receiving the vaccine (for example that results in death, hospitalization, a life-threatening reaction, a birth defect, or other important medical event)
  - A condition called "Multisystem Inflammatory Syndrome"
  - Cases of COVID-19 that result in hospitalization

US sites: Include the following boxed text. You can remove the box around the text.

We have a Certificate of Confidentiality from the US government to help protect your privacy. With the certificate, we do not have to release information about you to someone who is not connected to the study, such as the courts or police. Sometimes we can't use the certificate. Since the US National Institutes of Health funds this research, we cannot withhold information from it.

We will not share your name or information that can identify you with the CoVPN. The CoVPN may share information from this study with other researchers. Researchers may publish the results of this study.

Site: The text below may not be deleted or changed per FDA requirement. You can remove the box.

A description of this clinical trial will be available on http://www.ClinicalTrials.gov, as required by U.S. Law. This Web site will not include information that can identify you. At most, the Web site will include a summary of the results. You can search this website at any time.

11. **We may take you out of the study at any time.**

    We may take you out of the study if:

    - you do not follow instructions,
    - we think that staying in the study might harm you,
    - the study is stopped for any reason.

12. **If you get COVID-19, we will ask you to do several things.**

    If you find out that you have COVID-19, please let us know right away. We will ask you to quarantine per local guidelines. By agreeing to join this study, you also agree to share any SARS-CoV-2 test results with us.

    We will ask you to tell anyone with whom you have recently had close physical contact that they may have been exposed to COVID-19. We will give you a referral code to give to these contacts so that they can decide if they would like to join the Case Ascertained Close Contact group (CACC). You may remember at the beginning of this form that we told you that about 1,500 people will be enrolled in the CACC group. The CACC group will help us learn if people who got the study vaccine are less likely to give SARS-CoV-2 to someone else. When considering who to ask to be your CACC, you should think about whether you feel comfortable or safe sharing your infection status with them if you get SARS-CoV-2. If you don't feel comfortable sharing this information with them, you should not ask them to participate as a CACC.

    It is possible that someone you know may also be participating in the Main part of the study and might identify you as a close contact. They will not know you are also participating in the study unless you choose to tell them. If someone else identifies you as a close contact, they will give you their referral code. It is okay to be enrolled in both the Main part of the study and the CACC group.

    If you find out that you have COVID-19, you will be directed to contact the clinic, and we will ask you to either come to the clinic to have your blood drawn or we will ask you to self-collect less than one teaspoon of blood and complete one additional eDiary questionnaire. It is important that you give the blood sample as soon as possible after finding out the you have COVID-19.

    We will ask you to track your COVID-19 symptoms using the eDiary daily for 2 weeks or until the symptoms go away.

    After you get better, we will ask you to continue your study participation so that we can monitor your health and safety for the rest of the study.

If you are hospitalized, we will pause your participation. We will ask for your medical records and results from tests you might have received at the hospital. It is unlikely that you will be hospitalized, but to prepare for this, we will ask you to sign and date a medical release of information so we can get records from your doctor or view your hospital records. When you are released from the hospital, please let us know. We will ask you to resume your study participation so that we can continue to monitor your health and safety for the rest of the study.

Site: Modify the following sentence as appropriate. We will not pay for any of your COVID-19 care directly.

13. **If you receive an EUA or licensed COVID-19 vaccine outside the study we will stop your on-study vaccinations but you may continue in the study.**

    If you receive an EUA or licensed COVID-19 vaccine outside the study, please let us know. We will ask you to tell us what vaccine you received and when you received it. You may continue to participate in follow-up visits and procedures like swabbing your nose and completing eDiary entries. Your continued participation is valuable to help us meet our study goals.

Other Risks

14. **There are other risks to being in this study.**

    This section describes the other risks and restrictions we know about. There may also be unknown risks, even serious ones. We will tell you if we learn anything new that may affect your willingness to stay in the study.

    Risks of routine medical procedures:

    In this study, we will do some routine medical procedures. These are taking blood and giving injections. These procedures can cause bruising, pain, fainting, soreness, redness, swelling, itching, a sore, bleeding, and (rarely) muscle damage or infection where you got the injection.

    Risks of swabbing your nose:

    The feeling of having a small, soft-tipped swab inserted into your nostril and twirled around may be a little uncomfortable, but it should not be painful. There is a small chance there could be some bleeding, but this is unlikely.

    Risks to your personal information:

We will take several steps to protect your personal information. Although the risk is very low, it is possible that your personal information could be given to someone who should not have it. If that happened, you could have stress or anxiety.

Risks of genetic testing:

It is unlikely, but the genetic tests done on your samples could show you may be at risk for certain diseases. If others found out, it could lead to discrimination or other problems.

Risk of anxiety/emotional stress:

You may feel anxiety or emotional stress if you experience any of the risks described here. You may feel worried if tests show that you have SARS-CoV-2. Some of the questions we will ask you may make you feel uncomfortable.

Unknown risks:

The vaccine has been shown to prevent adults from getting COVID-19 for at least 2 to 3 months after their injection, but we do not know if it will protect them for any longer. If you get COVID-19, we do not know how the vaccine might affect your illness. It is also unknown if the vaccine will prevent you from giving the virus to someone else. If you get COVID-19, we do not know how the vaccine might affect your illness.

We do not know if getting this vaccine will affect how you respond to any future SARS-CoV-2 vaccines.

We do not know if getting the vaccine will affect pregnancy or breastfeeding. If you have concerns, we recommend that you discuss this with a health care provider.

While you are in this study, you should still wear a mask in public and practice physical distancing.

Benefits

15. **This study may not benefit you.**

This study may help researchers find out if the vaccine is able to prevent infection with SARS-CoV-2. If the vaccine later becomes approved and sold, there are no plans to share any money with you.

If you decide not to join this study, you could get the Moderna COVID-19 vaccine or another COVID-19 vaccine in your community. You can also continue to follow local guidelines for SARS-CoV-2 infection. You do not have to join this research study if you don’t want to.

Your rights and responsibilities

16. **If you join the study, you have rights and responsibilities.**

    You have many rights that we will respect. You also have responsibilities. We list these in the Bill of Rights and Responsibilities for Research. We will give you a copy of it.

Leaving the study

17. **Tell us if you decide to leave the study.**

    You are free to leave the study at any time and for any reason. If you want to leave, you will need to tell us. Your care at this clinic, your standing at your school, and your legal rights will not be affected.

    Previously collected information about you will remain in the study records and will be included in the analysis of results. Your information can not be removed from the study records.

    We may ask you to give a final blood sample or nasal swab.

    We believe these steps are important to protecting your health, but it is up to you whether to complete them.

Injuries

Sites: Approval from CoVPN Regulatory Affairs (at CoVPN.core.reg@fredhutch.org) is needed for any change (other than those that the instructions specifically request or those previously approved by CoVPN Regulatory Affairs) to the boxed text. You can remove the box around the text.

18. **If you get sick or injured during the study, contact us immediately.**

Paragraph below for all sites.

Your health is important to us. *(Sites: adjust the following 2 sentences if applicable to the care available at your site)* We will tell you about the care that we can give here. For the care that we cannot provide, we will explain how we will help you get care elsewhere.

2 paragraphs below for all sites.

This research study is covered by a US government program that may provide compensation for a serious injury or death. We will give you a handout with more information about this

program. The National Institutes of Health (NIH) does not have a mechanism to provide direct compensation for research related injury.

Some injuries are not physical. For example, you might be harmed emotionally by being in this study. Or you might lose wages because you cannot go to work. However, there are no funds to pay for these kinds of injuries, even if they are study related.

You always have the right to use the court system if you are not satisfied.

19. **When samples are no longer needed for this study, the CoVPN wants to use them in other studies and share them with other researchers.**

The CoVPN calls these samples "extra samples". The CoVPN will only allow your extra samples to be used in other studies if you agree to this. You will mark your decision at the end of this form.

Do I have to agree? No. You are free to say yes or no, or to change your mind after you sign this form. At your request, the CoVPN will destroy all extra samples that it has. Your decision will not affect your being in this study.

Where are the samples stored? Extra samples are stored in a secure central place called a repository. Your samples will be stored in the CoVPN repository in the United States.

How long will the samples be stored? There is no limit on how long your extra samples will be stored. *[Site: Revise the previous sentence to insert limits if your regulatory authority imposes them.]*

Will I be paid for the use of my samples? No. Researchers may make scientific discoveries or products using your samples. If this happens, there is no plan to share any money with you.

Will I benefit from allowing my samples to be used in other studies? Probably not. Results from these other studies are not given to you, this clinic, or your doctor. They may help other people in the future.

Will the CoVPN sell my samples and information? No, but the CoVPN may share your samples with other researchers. Once the CoVPN shares your samples and information, it may not be able to get them back.

What information is shared with researchers? The samples and information will be labeled with a code number which will not be removed. The key to the code will stay at this clinic. However, some information that the CoVPN shares may be personal, such as your race, ethnicity, sex, and health information from the study.

What kind of studies might be done with my extra samples and information? The studies will be related to vaccines, the immune system, coronavirus, and other diseases. Researchers may also do genetic testing on your samples.

If you agree, researchers may compare all of your genes (your genome) to the genomes of many other people. Researchers look for common patterns of genes to help them understand diseases. The researchers may put the information into a protected database so that other researchers can access it. Your name and other personal information will not be included.

Usually, no one could connect your genome to you as a person. There are rules against this. It's also really difficult to do. But there is a risk that someone could combine information from your genome and other public information about you and identify you. If others found out, it could lead to discrimination or other problems. The risk of this happening is very small.

Who will have access to my information in studies using my extra samples?

People who may see your information are:

- Researchers who use your extra samples and information for other research
- Government agencies that fund or monitor the research using your extra samples and information
- The researcher's Institutional Review Board or Ethics Committee
- Any regulatory agency that reviews research studies
- The people who work with the researcher

All of these people will do their best to protect your information. If they publish their research, they will not use your name or identify you personally.

Questions

20. **Whom to contact about this study**

During the study, if you experience any medical problems, suffer a research-related injury, or have questions, concerns, or complaints about the study, please contact the study doctor at the telephone number listed on the first page of this consent document. If you seek emergency care, or hospitalization is required, alert the treating physician that you are participating in this research study.

An institutional review board (IRB) is an independent committee established to help protect the rights of research subjects.

If you have any questions about your rights as a research subject, and/or concerns or complaints regarding this research study, contact:
by mail:

Study Subject Adviser

Advarra IRB

6940 Columbia Gateway Drive, Suite 110

Columbia, MD 21046

or call **toll free**: 877-992-4724
or by **email**: adviser@advarra.com

Please reference the following number when contacting the Study Subject Adviser: Pro00049375

US sites may include, but are not required to: You may also contact the [name of local IRB/EC] at [insert contact information].

Your permissions and signature

21. **In Section 18, we told you above about possible other uses of your extra samples** and information outside this study. Please choose only one of the options below and write your initials in the box next to it. Whatever you choose, the CoVPN keeps track of your decision about how your samples and information can be used. You can change your mind after signing this form.

☐ I allow my extra samples and information to be used for other studies related to vaccines, the immune system, coronavirus, and other diseases. This may include genetic testing.

OR

☐ I agree to the option above *and* also to allow my extra samples and information to be used in studies that look at my whole genome.

OR

☐ I do not allow my extra samples to be used in any other studies. This includes not allowing genetic testing, or studies that look at my whole genome.

22. **If you agree to join this study, you will need to sign below. Before you sign, make sure of the following:**

- You have read this consent form.
- You feel that you understand what the study is about and what will happen to you if you join. You understand what the possible risks and benefits are.
- You have had your questions answered and know that you can ask more.
- You agree to join this study.

You will not be giving up any of your rights by signing this consent form.

Participant's Name (Print)	Participant's Signature	Date
Clinic Staff Conducting Consent Discussion (Print)	Clinic Staff Signature	Date

Appendix B Sample informed consent form for Prospective Close Contact (PCC) cohort

**Sponsor / Study Title:**	**National Institutes of Health, "A randomized controlled study to assess SARS CoV-2 infection, viral shedding, and subsequent potential transmission in university students immunized with Moderna COVID-19 Vaccine"**
**Protocol Number:**	**CoVPN 3006**
**Principal Investigator: (Study Doctor)**	**«PiFullName»**
**Telephone:**	**«IcfPhoneNumber»**
**Address:**	**«PiLocations»**

Thank you for your interest in our research study. It is called CoVPN 3006. This informed consent form will tell you more about the study. Please read it carefully to help you decide if you want to join. If you have questions, please ask us.

If you decide to join this study, we will ask you to sign this form. We will offer you a copy of this form to keep.

CoVPN 3006 is a research study. Research is not the same as medical care. The purpose of a research study is to answer scientific questions. We hope that what we learn will help people in the future.

Key information

- Joining this research study is voluntary. It is your choice.
- Our scientific questions are: Does the study vaccine protect people from getting infected with a coronavirus called SARS-CoV-2? Does the study vaccine prevent people from transmitting SARS-CoV-2 to others?
- If you join, your participation in this study will last for about 5 months.

- If you join, we will ask you to answer weekly eDiary questionnaires. If you or your contact in the main study group becomes SARS-CoV-2 infected, then we will ask you to take daily swabs of your nose, answer daily eDiary questionnaire for 2 weeks, and give 2 blood samples.

  Here are the risks of taking part:

- There is a risk of loss of your personal information.

- There are other, less serious risks. We will tell you more about them later in this consent form.

About the study

The COVID-19 Prevention Network (CoVPN) is doing a study to test a vaccine against SARS-CoV-2. SARS-CoV-2 is the virus that causes the disease called COVID-19. We want to know if the vaccine is able to protect people from getting infected and if getting the vaccine will affect the amount of virus that is in your nose. We also want to know if getting the vaccine will prevent transmitting SARS-CoV-2 to others.

We are asking you to join this study because you have been referred as a close contact with someone who is in the Main study and is getting injections of the vaccine.

Many people will take part in this study. About 12,000 people will get injections while about 24,000 will be in the Prospective Close Contact (PCC) group and up to about 1,500 more will be in the Case-ascertained Close Contact (CACC) group. These close contact groups will help researchers determine if the vaccine prevents transmission of the SARS-CoV-2 virus.

People who join the close contact groups and Main study are being recruited from many different schools in the United States (US). You are being asked to participate in the PCC group. The researcher in charge of this study at this clinic is [Insert name of site PI]. The US National Institutes of Health is paying for this study.

Joining the study

1. **It is completely up to you whether or not to join this study.**

   Take your time in deciding. If it helps, talk to people you trust, such as your friends or family. If you decide not to join this study, or if you leave it after you have joined, it will not change your healthcare. If you do join, you do not give up your legal rights.

   If you are an employee or relative of an employee of the school, you are under no obligation to participate in this study. You may withdraw from the study at any time and for any reason, and your decision will not have any effect on your/your family member's performance

appraisal or employment at the school. You may refuse to participate or you may withdraw from the study at any time without penalty or anyone blaming you.

If you are a student, your participation will not place you in good favor with the study doctor or other faculty (for example, receiving better grades, recommendations, employment). Also, not participating in this study will not adversely affect your relationship with the study doctor or other faculty.

Being in the study

If you want to join, here is what will happen:

We will send you information about how to download an electronic diary (eDiary) app onto your phone or tablet. We can help you download the app and show you how to use it. In order to use the app you will be asked to agree to the Terms of Use and Privacy Policy which will appear on your mobile device's screen when you first start using the app. If you decide that you do not want to agree, then you should not participate in the research. While using the app, data about you including personal health information, other communication data, and internet usage will be collected and transmitted to the researchers and to the app developer. A complete description of this data collection and sharing is found in the Privacy Policy. Transmission of information via the internet is not completely secure, so there is a small risk of unintentional release of your information and safeguards are in place to protect your personal information. While the Terms of Use may include statements limiting your rights if you are harmed in this study, you do not release the investigator, sponsor, institution, or agents from responsibility for mistakes, and these statements do not apply to the use of the app in this research study.

Initially, you will answer a 5-minute questionnaire in the eDiary. It will ask you about things such as your sex, age, race, and ethnicity. You will also answer weekly questionnaires about whether you are sick or if you have tested positive for SARS-CoV-2. You will also take an End of Study questionnaire in the eDiary. If you have tested positive, you will enter the results of your SARS-CoV-2 tests taken at school or another location into the eDiary.

As part of the study, you may get SARS-CoV-2 testing at your school about twice a week.

If you or your contact in the main study group becomes SARS-CoV-2 infected, then we will ask you to take swabs of your nose and answer eDiary questionnaires every day for 2 weeks. You will return your nasal swabs using drop-off location(s) specified by the clinic or by mail.

We will ask you to self-collect a small amount of your blood twice using a small device called the Tasso-SST OnDemand. You will take the first blood sample as soon as possible, and then you will take another sample about a month later. This will be less than one teaspoon of blood taken from one of your upper arms using a small device. We will give you everything you need to take the blood and return it to us.

The self-collection device sticks to the skin with a light adhesive. When the button is pressed, a vacuum forms, and the device will prick the surface of the skin. The vacuum draws blood out of the capillaries and into a sample pod attached to the bottom of the device.

It is important that you continue to follow the local guidelines for SARS-CoV-2 prevention, like wearing a mask and keeping physical distance from others. If you get COVID-19 and are hospitalized, we will pause your participation. We will ask for your medical records and results from tests you might have received at the hospital. It is unlikely that you will be hospitalized, but to prepare for this, we will ask you to sign and date a medical release of information so we can get records from your doctor or view your hospital records. When you are released from the hospital, please let us know. We will ask you to resume your study participation so that we can continue to monitor your health and safety for the rest of the study.

We will not pay for any of your COVID-19 care directly.

2. **The CoVPN will test your samples to see how your body, including your immune system, responds to the potential infection.**

   We will send your samples to labs approved by the CoVPN. Your samples will not be labeled with your name or other identifying information.

   Researchers may also do genetic testing related to this study on your samples. Your genes are passed to you from your birth parents. Differences in people's genes can help explain why some people get a disease while others do not. The genetic testing will only involve some of your genes, not all of your genes (your genome). The researchers will study only the genes related to the immune system and coronavirus, and genes that may affect how people get coronavirus.

   If you get SARS-CoV-2, the researchers may look at all of the virus' genes that are in your samples. The researchers will use this information to learn more about SARS-CoV-2 and how the virus is impacted by the study vaccine.

   The tests the researchers do on your samples are for research purposes, not to check your health. However, the nasal swab testing you may do as part of this study may tell us if you are SARS-CoV-2 positive. Nasal swab test results from the study will be provided to the sites and be made available to you. You may be asked to get a confirmatory test. For all other lab tests, we will not provide the results to you or the site.

   When your samples are no longer needed for this study, the CoVPN will continue to store them.

3. **When samples are no longer needed for this study, the CoVPN wants to use them in other studies and share them with other researchers.**

The CoVPN calls these samples “extra samples”. The CoVPN will only allow your extra samples to be used in other studies if you agree to this. You will mark your decision at the end of this form.

Do I have to agree? No. You are free to say yes or no, or to change your mind after you sign this form. At your request, the CoVPN will destroy all extra samples that it has. Your decision will not affect your being in this study.

Where are the samples stored? Extra samples are stored in a secure central place called a repository. Your samples will be stored in the CoVPN repository in the United States.

How long will the samples be stored? There is no limit on how long your extra samples will be stored. *[Site: Revise the previous sentence to insert limits if your regulatory authority imposes them.]*

Will I be paid for the use of my samples? No. Researchers may make scientific discoveries or products using your samples. If this happens, there is no plan to share any money with you.

Will I benefit from allowing my samples to be used in other studies? Probably not. Results from these other studies are not given to you, this clinic, or your doctor. They may help other people in the future.

Will the CoVPN sell my samples and information? No, but the CoVPN may share your samples with other researchers. Once the CoVPN shares your samples and information, it may not be able to get them back.

What information is shared with researchers? The samples and information will be labeled with a code number which will not be removed. The key to the code will stay at this clinic. However, some information that the CoVPN shares may be personal, such as your race, ethnicity, sex, and health information from the study.

What kind of studies might be done with my extra samples and information? The studies will be related to vaccines, the immune system, coronavirus, and other diseases.

Researchers may also do genetic testing on your samples.

If you agree, researchers may compare all of your genes (your genome) to the genomes of many other people. Researchers look for common patterns of genes to help them understand diseases. The researchers may put the information into a protected database so that other researchers can access it. Your name and other personal information will not be included.

Usually, no one could connect your genome to you as a person. There are rules against this. It's also really difficult to do. But there is a risk that someone could combine information from your genome and other public information about you and identify you. If others found out, it could lead to discrimination or other problems. The risk of this happening is very small.

Who will have access to my information in studies using my extra samples?

People who may see your information are:

- Researchers who use your extra samples and information for other research
- Government agencies that fund or monitor the research using your extra samples and information
- The researcher's Institutional Review Board or Ethics Committee
- Any regulatory agency that reviews research studies
- The people who work with the researcher

All of these people will do their best to protect your information. If they publish their research, they will not use your name or identify you personally.

4. **We will compensate you for your participation.**

Site: Insert your compensation plan here, e.g. money for number of swabs returned. Remember to include any costs to participants.

5. **We will do our best to protect your private information.**

Site: Check HIPAA authorization for conflicts with this section.

Your study records and samples will be kept in a secure location. We will label all of your samples and most of your records with a code number, not your name or other personal information. We will not share your name with anyone who does not need to know it.

Your records may also be reviewed by groups who watch over this study. These groups include:

- Study monitors
- The CoVPN, people who work for it, and companies that help it with this study
- Some government agencies:

  - The US National Institutes of Health
  - The US Office for Human Research Protections
  - Any regulatory agency that reviews research studies
- Some committees that make sure we protect your rights and keep you safe:
  - The Independent Data Monitoring Committee
  - Advarra Institutional Review Board (IRB)
  - *Sites may but are not required to include:* [Insert name of local IRB/EC]
  - *Sites include:* [Insert name of local IBC]

All reviewers will take steps to keep your records private.

Sites: Include the following boxed text. You can remove the box around the text.

We have a Certificate of Confidentiality from the US government to help protect your privacy. With the certificate, we do not have to release information about you to someone who is not connected to the study, such as the courts or police. Sometimes we can't use the certificate. Since the US National Institutes of Health funds this research, we cannot withhold information from it.

We will not share your name or information that can identify you with the CoVPN. The CoVPN may share information from this study with other researchers. Researchers may publish the results of this study.

Sites: The text below may not be deleted or changed per FDA requirement. You can remove the box.

A description of this clinical trial will be available on http://www.ClinicalTrials.gov, as required by U.S. Law. This Web site will not include information that can identify you. At most, the Web site will include a summary of the results. You can search this website at any time.

6. **We may take you out of the study at any time.**

We may take you out of the study if:

- you do not follow instructions,
- we think that staying in the study might harm you,

- the study is stopped for any reason.

Risks

7. **There are some risks to being in this study.**

   This section describes the risks we know about. There may also be unknown risks, even serious ones. We will tell you if we learn anything new that may affect your willingness to stay in the study.

   Risks of blood draws:

   In this study, we will either take your blood or ask you to self-collect your blood with a small device. Taking blood can cause bruising, pain, fainting, soreness, redness, and itching.

   Risks of swabbing your nose:

   The feeling of having a small, soft-tipped swab inserted into your nostril and twirled around may be a little uncomfortable, but it should not be painful. There is a small chance there could be some bleeding, but this is unlikely.

   Risks to your personal information:

   We will take several steps to protect your personal information. Although the risk is very low, it is possible that your personal information could be given to someone who should not have it. If that happened, you could have stress or anxiety.

Benefits

8. **This study may not benefit you**.

   This study may help researchers find out if people who got the vaccine are less likely to give it to people who they have been in close contact with. If the vaccine later becomes approved and sold, there are no plans to share any money with you.

   You do not have to join this research study if you don't want to.

Leaving the study

9. **Tell us if you decide to leave the study.**

   You are free to leave the study at any time and for any reason. If you want to leave, you will need to tell us. Your legal rights will not be affected.

Previously collected information about you will remain in the study records and will be included in the analysis of results. Your information can not be removed from the study records.

We may ask you to give a final blood sample or nasal swab.

We believe these steps are important to protecting your health, but it is up to you whether to complete them.

Injuries

Sites: Approval from CoVPN Regulatory Affairs (at CoVPN.core.reg@fredhutch.org) is needed for any change (other than those that the instructions specifically request or those previously approved by CoVPN Regulatory Affairs) to the boxed text. You can remove the box around the text.

10. If you have a life-threatening medical emergency, call 911 right away or seek help immediately.

We do not expect you to get sick or injured as a result of being in this study. If you get COVID-19 while you are in the study, please let us know. *(Sites: adjust the following 2 sentences if applicable to the care available at your site)* We will tell you about the care that we can give here. For the care that we cannot provide, we will explain how we will help you get care elsewhere. You or your health insurance will have to pay for the care you get elsewhere.

Some injuries are not physical. For example, you might be harmed emotionally by being in this study. Or you might lose wages because you cannot go to work. However, there are no funds to pay for these kinds of injuries, even if they are study related.

You always have the right to use the court system if you are not satisfied.

Questions

11. **Whom to contact about this study**

During the study, if you experience any medical problems, suffer a research-related injury, or have questions, concerns, or complaints about the study, please contact the study doctor at the telephone number listed on the first page of this consent document.

An institutional review board (IRB) is an independent committee established to help protect the rights of research subjects.

If you have any questions about your rights as a research subject, and/or concerns or complaints regarding this research study, contact:

by mail:

Study Subject Adviser

Advarra IRB

6940 Columbia Gateway Drive, Suite 110

Columbia, MD 21046

or call **toll free**: 877-992-4724
or by **email**: adviser@advarra.com

Please reference the following number when contacting the Study Subject Adviser: Pro00049375

US sites may include, but are not required to: You may also contact the [name of local IRB/EC] at [insert contact information].

Your permissions and signature

12. **In Section 3, we told you above about possible other uses of your extra samples** and information outside this study. Please choose only one of the options below and write your initials in the box next to it. Whatever you choose, the CoVPN keeps track of your decision about how your samples and information can be used. You can change your mind after signing this form.

☐ I allow my extra samples and information to be used for other studies related to vaccines, the immune system, coronavirus, and other diseases. This may include genetic testing.

OR

☐ I agree to the option above *and* also to allow my extra samples and information to be used in studies that look at my whole genome.

OR

☐ I do not allow my extra samples to be used in any other studies. This includes not allowing genetic testing, or studies that look at my whole genome.

13. If you agree to join this study, you will need to sign below. Before you sign, make sure of the following:

- You have read this consent form.

- You feel that you understand what the study is about and what will happen to you if you join. You understand what the possible risks and benefits are.
- You agree to join this study.

You will not be giving up any of your rights by signing this consent form.

Participant's Name (Print)	Participant's Signature	Date
Clinic Staff Conducting Consent Discussion (Print)	Clinic Staff Signature	Date

Appendix C Sample informed consent form for Case-Ascertained Close Contact (CACC) cohort

**Sponsor / Study Title:**	**National Institutes of Health, “A randomized controlled study to assess SARS CoV-2 infection, viral shedding, and subsequent potential transmission in university students immunized with Moderna COVID-19 Vaccine”**
**Protocol Number:**	**CoVPN 3006**
**Principal Investigator: (Study Doctor)**	**«PiFullName»**
**Telephone:**	**«IcfPhoneNumber»**
**Address:**	**«PiLocations»**

Thank you for your interest in our research study. It is called CoVPN 3006. This informed consent form will tell you more about the study. Please read it carefully to help you decide if you want to join. If you have questions, please ask us.

If you decide to join this study, we will ask you to sign this form. We will offer you a copy of this form to keep.

CoVPN 3006 is a research study. Research is not the same as medical care. The purpose of a research study is to answer scientific questions. We hope that what we learn will help people in the future.

Key information

- Joining this research study is voluntary. It is your choice.
- Our scientific questions are: Does the study vaccine protect people from getting infected with a coronavirus called SARS-CoV-2? Does the study vaccine prevent people from transmitting SARS-CoV-2 to others?
- If you join, your participation in this study will last for about 1 month.

- If you join, we will ask you to answer questionnaires, give blood, and take daily swabs of your nose for 2 weeks.

  Here are the risks of taking part:

- There is a risk of loss of your personal information.

- There are other, less serious risks. We will tell you more about them later in this consent form.

About the study

The COVID-19 Prevention Network (CoVPN) are doing a study to test a vaccine against SARS-CoV-2. SARS-CoV-2 is the virus that causes the disease called COVID-19. We are asking you to join this study because you recently had close physical contact with someone who is in the Main study and who got infected with SARS-CoV-2. We want to find out if the person you had close physical contact might have transmitted SARS-CoV-2 to you.

Many people will take part in this study. About 12,000 people will get injections while about 24,000 will be in the Prospective Close Contact (PCC) group and up to about 1,500 more will be in the Case-ascertained Close Contact (CACC) group. These close contact groups will help researchers determine if the vaccine prevents transmission of the SARS-CoV-2 virus.

People who join the close contact groups and Main study are being recruited from many different schools in the United States (US). You are being asked to participate in the CACC group. The researcher in charge of this study at this clinic is [Insert name of site PI]. The US National Institutes of Health is paying for this study.

Joining the study

1. **It is completely up to you whether or not to join this study.**

   Take your time in deciding. If it helps, talk to people you trust, such as your friends or family. If you decide not to join this study, or if you leave it after you have joined, it will not change your healthcare. If you do join, you do not give up your legal rights.

   If you are an employee or relative of an employee of the school, you are under no obligation to participate in this study. You may withdraw from the study at any time and for any reason, and your decision will not have any effect on your/your family member's performance appraisal or employment at the school. You may refuse to participate or you may withdraw from the study at any time without penalty or anyone blaming you.

   If you are a student, your participation will not place you in good favor with the study doctor or other faculty (for example, receiving better grades, recommendations, employment). Also,

not participating in this study will not adversely affect your relationship with the study doctor or other faculty.

Being in the study

If you want to join, here is what will happen:

We will send you information about how to download an electronic diary (eDiary) app onto your phone or tablet. We can help you download the app and show you how to use it. In order to use the app you will be asked to agree to the Terms of Use and Privacy Policy which will appear on your mobile device's screen when you first start using the app. If you decide that you do not want to agree, then you should not participate in the research. While using the app, data about you including personal health information, other communication data, and internet usage will be collected and transmitted to the researchers and to the app developer. A complete description of this data collection and sharing is found in the Privacy Policy. Transmission of information via the internet is not completely secure, so there is a small risk of unintentional release of your information and safeguards are in place to protect your personal information. While the Terms of Use may include statements limiting your rights if you are harmed in this study, you do not release the investigator, sponsor, institution, or agents from responsibility for mistakes, and these statements do not apply to the use of the app in this research study.

You will take a 5-minute questionnaire in the eDiary. It will ask you about your contact with the person who tested positive for SARS-CoV-2, as well as other things such as your sex, age, race, and ethnicity. You will also take an End of Study questionnaire in the eDiary. If you have tested positive for SARS-CoV-2, you will enter the results of your SARS-CoV-2 tests taken at school or another location into the eDiary.

If you are affiliated with a school that provides SARS-CoV-2 testing, you may get tested about twice a week.

We will ask you to take daily swabs of both of your nostrils and track your COVID-19 symptoms in the eDiary daily for two weeks. You will return your nasal swabs using drop-off location(s) specified by the clinic or by mail.

We will also ask you to collect a small amount of blood from one of your upper arms twice during the study using a small device called the Tasso-SST OnDemand. This will be less than one teaspoon of blood taken from one of your upper arms using a small device. You will take the first blood sample as soon as possible, and then you will take another sample about a month later. We will give you everything you need to take these samples and return them to us.

The self-collection device sticks to the skin with a light adhesive. When the button is pressed, a vacuum forms, and the device will prick the surface of the skin. The vacuum draws blood out of the capillaries and into a sample pod attached to the bottom of the device.

It is important that you continue to follow the local guidelines for SARS-CoV-2 prevention, like wearing a mask and keeping physical distance from others.

If you are hospitalized, we will pause your participation. We will ask for your medical records and results from tests you might have received at the hospital. It is unlikely that you will be hospitalized, but to prepare for this, we will ask you to sign and date a medical release of information so we can get records from your doctor or view your hospital records. When you are released from the hospital, please let us know. We will ask you to resume your study participation so that we can continue to monitor your health and safety for the rest of the study.

2. **The CoVPN will test your samples to see how your body, including your immune system, responds to the potential infection.**

   We will send your samples to labs approved by the CoVPN. Your samples will not be labeled with your name or other identifying information.

   Researchers may also do genetic testing related to this study on your samples. Your genes are passed to you from your birth parents. Differences in people's genes can help explain why some people get a disease while others do not. The genetic testing will only involve some of your genes, not all of your genes (your genome). The researchers will study only the genes related to the immune system and coronavirus, and genes that may affect how people get coronavirus.

   If you get SARS-CoV-2, the researchers may look at all of the virus' genes that are in your samples. The researchers will use this information to learn more about SARS-CoV-2 and how the virus is impacted by the study vaccine.

   The tests the researchers do on your samples are for research purposes, not to check your health. However, the nasal swab testing you do as part of this study may tell us if you are SARS-CoV-2 positive. Nasal swab test results from the study will be provided to the sites and be made available to you. You may be asked to get a confirmatory test. For all other lab tests, we will not provide the results to you or the site.

   When your samples are no longer needed for this study, the CoVPN will continue to store them.

3. **When samples are no longer needed for this study, the CoVPN wants to use them in other studies and share them with other researchers.**

   The CoVPN calls these samples "extra samples". The CoVPN will only allow your extra samples to be used in other studies if you agree to this. You will mark your decision at the end of this form.

Do I have to agree? No. You are free to say yes or no, or to change your mind after you sign this form. At your request, the CoVPN will destroy all extra samples that it has. Your decision will not affect your being in this study.

Where are the samples stored? Extra samples are stored in a secure central place called a repository. Your samples will be stored in the CoVPN repository in the United States.

How long will the samples be stored? There is no limit on how long your extra samples will be stored. *[Site: Revise the previous sentence to insert limits if your regulatory authority imposes them.]*

Will I be paid for the use of my samples? No. Researchers may make scientific discoveries or products using your samples. If this happens, there is no plan to share any money with you.

Will I benefit from allowing my samples to be used in other studies? Probably not. Results from these other studies are not given to you, this clinic, or your doctor. They may help other people in the future.

Will the CoVPN sell my samples and information? No, but the CoVPN may share your samples with other researchers. Once the CoVPN shares your samples and information, it may not be able to get them back.

What information is shared with researchers? The samples and information will be labeled with a code number which will not be removed. The key to the code will stay at this clinic. However, some information that the CoVPN shares may be personal, such as your race, ethnicity, sex, and health information from the study.

What kind of studies might be done with my extra samples and information? The studies will be related to vaccines, the immune system, coronavirus, and other diseases.

Researchers may also do genetic testing on your samples.

If you agree, researchers may compare all of your genes (your genome) to the genomes of many other people. Researchers look for common patterns of genes to help them understand diseases. The researchers may put the information into a protected database so that other researchers can access it. Your name and other personal information will not be included.

Usually, no one could connect your genome to you as a person. There are rules against this. It’s also really difficult to do. But there is a risk that someone could combine information from your genome and other public information about you and identify you. If others found out, it could lead to discrimination or other problems. The risk of this happening is very small.

Who will have access to my information in studies using my extra samples?

People who may see your information are:

- Researchers who use your extra samples and information for other research
- Government agencies that fund or monitor the research using your extra samples and information
- The researcher's Institutional Review Board or Ethics Committee
- Any regulatory agency that reviews research studies
- The people who work with the researcher

All of these people will do their best to protect your information. If they publish their research, they will not use your name or identify you personally.

4. **We will compensate you for your participation.**

Site: Insert your compensation plan here, e.g. money for number of swabs returned. Remember to include any costs to participants.

5. **We will do our best to protect your private information.**

Site: Check HIPAA authorization for conflicts with this section.

Your study records and samples will be kept in a secure location. We will label all of your samples and most of your records with a code number, not your name or other personal information. We will not share your name with anyone who does not need to know it.

Your records may also be reviewed by groups who watch over this study. These groups include:

- Study monitors
- The CoVPN, people who work for it, and companies that help it with this study
- Some government agencies:
  - The US National Institutes of Health
  - The US Office for Human Research Protections
  - Any regulatory agency that reviews research studies
- Some committees that make sure we protect your rights and keep you safe:

- The Independent Data Monitoring Committee
- Advarra Institutional Review Board (IRB)
- *Sites may but are not required to include:* [Insert name of local IRB/EC]
- *Sites include:* [Insert name of local IBC]

All reviewers will take steps to keep your records private.

Sites: Include the following boxed text. You can remove the box around the text.

We have a Certificate of Confidentiality from the US government to help protect your privacy. With the certificate, we do not have to release information about you to someone who is not connected to the study, such as the courts or police. Sometimes we can't use the certificate. Since the US National Institutes of Health funds this research, we cannot withhold information from it.

We will not share your name or information that can identify you with the CoVPN. The CoVPN may share information from this study with other researchers. Researchers may publish the results of this study.

Sites: The text below may not be deleted or changed per FDA requirement. You can remove the box.

A description of this clinical trial will be available on http://www.ClinicalTrials.gov, as required by U.S. Law. This website will not include information that can identify you. At most, the website will include a summary of the results. You can search this website at any time.

6. **We may take you out of the study at any time.**

We may take you out of the study if:

- you do not follow instructions,
- we think that staying in the study might harm you,
- the study is stopped for any reason.

Risks

7. **There are some risks to being in this study.**

   This section describes the risks we know about. There may also be unknown risks, even serious ones. We will tell you if we learn anything new that may affect your willingness to stay in the study.

   Risks of blood draws:

   In this study, we will either take your blood or ask you to self-collect your blood with a small device. Taking blood can cause bruising, pain, fainting, soreness, redness, and itching.

   Risks of swabbing your nose:

   The feeling of having a small, soft-tipped swab inserted into your nostril and twirled around may be a little uncomfortable, but it should not be painful. There is a small chance there could be some bleeding, but this is unlikely.

   Risks to your personal information:

   We will take several steps to protect your personal information. Although the risk is very low, it is possible that your personal information could be given to someone who should not have it. If that happened, you could have stress or anxiety.

Benefits

8. **This study may not benefit you.**

   This study may help researchers find out if people who got the vaccine and later got infected with SARS-CoV-2 are less likely to give it to people who they have been in close contact with. If the vaccine later becomes approved and sold, there are no plans to share any money with you.

   You do not have to join this research study if you don't want to.

Leaving the study

9. **Tell us if you decide to leave the study.**

   You are free to leave the study at any time and for any reason. If you want to leave, you will need to tell us. Your legal rights will not be affected.

Previously collected information about you will remain in the study records and will be included in the analysis of results. Your information can not be removed from the study records.

We may ask you to give a final blood sample or nasal swab.

We believe these steps are important to protecting your health, but it is up to you whether to complete them.

Injuries

Sites: Approval from CoVPN Regulatory Affairs (at CoVPN.core.reg@fredhutch.org) is needed for any change (other than those that the instructions specifically request or those previously approved by CoVPN Regulatory Affairs) to the boxed text. You can remove the box around the text.

10. If you have a life-threatening medical emergency, call 911 right away or seek help immediately.

We do not expect you to get sick or injured as a result of being in this study. If you get COVID-19 while you are in the study, please let us know. *(Sites: adjust the following 2 sentences if applicable to the care available at your site)* We will tell you about the care that we can give here. For the care that we cannot provide, we will explain how we will help you get care elsewhere. You or your health insurance will have to pay for the care you get elsewhere.

Some injuries are not physical. For example, you might be harmed emotionally by being in this study. Or you might lose wages because you cannot go to work. However, there are no funds to pay for these kinds of injuries, even if they are study related.

You always have the right to use the court system if you are not satisfied.

Questions

11. **Whom to contact about this study**

During the study, if you experience any medical problems, suffer a research-related injury, or have questions, concerns, or complaints about the study, please contact the study doctor at the telephone number listed on the first page of this consent document.

An institutional review board (IRB) is an independent committee established to help protect the rights of research subjects.

If you have any questions about your rights as a research subject, and/or concerns or complaints regarding this research study, contact:

by mail:

Study Subject Adviser

Advarra IRB

6940 Columbia Gateway Drive, Suite 110

Columbia, MD 21046

or call **toll free**: 877-992-4724
or by **email**: adviser@advarra.com

Please reference the following number when contacting the Study Subject Adviser: Pro00049375

Sites may include, but are not required to: You may also contact the [name of local IRB/EC] at [insert contact information].

Your permissions and signature

12. In Section 3, we told you above about possible other uses of your extra samples and information outside this study. Please choose only one of the options below and write your initials in the box next to it. Whatever you choose, the CoVPN keeps track of your decision about how your samples and information can be used. You can change your mind after signing this form.

☐ I allow my extra samples and information to be used for other studies related to vaccines, the immune system, coronavirus, and other diseases. This may include genetic testing.

OR

☐ I agree to the option above *and* also to allow my extra samples and information to be used in studies that look at my whole genome.

OR

☐ I do not allow my extra samples to be used in any other studies. This includes not allowing genetic testing, or studies that look at my whole genome.

13. **If you agree to join this study, you will need to sign below. Before you sign, make sure of the following:**

- You have read this consent form.

- You feel that you understand what the study is about and what will happen to you if you join. You understand what the possible risks and benefits are.
- You agree to join this study.

You will not be giving up any of your rights by signing this consent form.

Participant's Name (Print)	Participant's Signature	Date
Clinic Staff Conducting Consent Discussion (Print)	Clinic Staff Signature	Date

Appendix D Procedures for Main Cohort, Immediate Vaccination Group

Visit:	01[1]	02[2]	03	04	05	06
Day:		D1	D29	D57	D113	D141
Week:		W0	W4	W8	W16	W20
Month:		M0	M1	M2	M4	M5
Procedure	Scr.	VAC1	VAC2			
**Study procedures**						
Screening consent (if used)	✓	–	–	–	–	–
Assessment of understanding	✓	–	–	–	–	–
Protocol consent (eConsent)	✓	–	–	–	–	–
Medical history questionnaire[3]	✓	✓	–	–	–	–
SARS-CoV-2 infection risk assessment[3]	✓	Weekly				
COVID-19 symptom surveillance[3]	–	Weekly starts at week 2, not during week 5				
Confirm eligibility	✓	–	–	–	–	–
Obtain demographics[3]	✓	–	–	–	–	–
Randomize	–	✓	–	–	–	–
SARS-CoV-2 screening at school, if applicable[3]	–	approximately 2x per week				
End of study questionnaire[3]	–	–	–	–	✓	–
**Research Samples[4]**						
Serum for Immunogenicity Assays and Storage	–	✓	–	✓	–	–
Serum for SARS-CoV-2 clinical serology[5]	–	✓[6]	–	✓[6]	✓	–
Nasal swab	–	Daily[7]				–
**Vaccination procedures**						
COVID-19 symptom check[8]	–	✓	✓	–	–	–
Vaccination	–	✓	✓	–	–	–

Greyed out visit column does not apply to this group.
1 Screening may occur over the course of several contacts/visits up to and including day 1, prior to vaccination.
2 Blood draws required at vaccination visits must be performed prior to vaccination.
3 Will be captured via eDiary, please see Section 9.16.
4 From screening to the final study visit, the total blood volume to be collected from each participant will be approximately 40 mL (maximum) with a maximum of approximately 17 mL collected in a single visit.

5 For blood self-collected by a participant the maximum volume will be less than 1 mL in a single collection.

6 SARS-CoV-2 clinical serology testing may be performed on leftover blood from samples collected for immunogenicity assays. No separate blood sample is needed. If a Main cohort participant is also in the Prospective Close Contact (PCC) cohort related to an index case or in the Case-ascertained Close Contact (CACC) cohort, one additional blood collection (self-collection) will be performed (see table below).

7 Daily swabs will be collected from vaccination through D113. Swabs will be returned via drop-off location(s) specified by the clinic or through the mail.

8 COVID-19 Symptom Check to be performed within 24 hours prior to vaccination.

The following **additional** procedures will occur for Main cohort, immediate vaccination participants, who fall under any of the following categories:

- tested positive for SARS-CoV-2 from enrollment through Month 4 (see Section 9.7)
- enrolled also in the PCC cohort and are related to an index case (see Section 9.8)
- enrolled also in the CACC cohort (see Section 9.9)

Day relative to notification of infection:	D1-14
**Study procedures**	
Symptom tracking via eDiary	daily
**Research Samples**	
Serum for SARS-CoV-2 clinical serology[1]	✓[2]

[1] For blood self-collected by a participant the maximum volume will be less than 1 mL in a single collection.
[2] Sample for serology will be collected as soon as possible following report of positive SARS-CoV-2 test.

In addition, we may ask participants to sign a release to allow us to review their COVID-19 related medical records.

Appendix E Procedures for Main Cohort, Delayed Vaccination Group

Visit:	01[1]	02	03	04	05	06
Day:		D1	D29	D57	D113	D141
Week:		W0	W4	W8	W16	W20
Month:		M0	M1	M2	M4	M5
Procedure	Scr.				VAC1	VAC2
**Study procedures**						
Screening consent (if used)	✓	–		–	–	–
Assessment of understanding	✓	–		–	–	–
Protocol consent (eConsent)	✓	–		–	–	–
Medical history questionnaire[2]	✓	✓		–	–	–
SARS-CoV-2 infection risk assessment[2]	✓	Weekly				–
COVID-19 symptom surveillance[2]	–	Weekly				–
Confirm eligibility	✓	–		–	–	–
Obtain demographics[2]	✓	–		–	–	–
Randomize	–	✓		–	–	–
SARS-CoV-2 screening at school, if applicable[2]	–	approximately 2x per week				–
End of study questionnaire[2]	–	–		–	–	✓
**Research Samples**						
Serum for SARS-CoV-2 clinical serology[3]	–	✓		✓	✓	–
Nasal swab	–	Daily[4]				–
**Vaccination procedures**						
COVID-19 symptom check[5]	–	–		–	✓	✓
Vaccination	–	–		–	✓	✓

Greyed out visit does not apply to this group.

1 Screening may occur over the course of several contacts/visits up to and including day 1, prior to randomization.
2 Will be captured via eDiary, please see Section 9.16.
3 From screening to the final study visit, the total blood volume to be collected from each participant will be approximately 15 mL (maximum) with a maximum of approximately 5 mL collected in a single visit. For blood self-collected by a participant the maximum volume will be less than 1 mL in a

single collection. If a Main cohort participant is also in the Prospective Close Contact (PCC) cohort related to an index case or in the Case-ascertained Close Contact (CACC) cohort, one additional blood collection (self-collection) will be performed (see table below).

4 Daily swabs will be collected from D1 through D113. Swabs will be returned via drop-off location(s) specified by the clinic or through the mail.

5 COVID-19 Symptom Check to be performed within 24 hours prior to vaccination.

The following **additional** procedures will occur for Main cohort, delayed vaccination participants, who fall under any of the following categories:

- tested positive for SARS-CoV-2 from enrollment through Month 4 (see Section 9.7)
- enrolled also in the PCC cohort and are related to an index case (see Section 9.8)
- enrolled also in the CACC cohort (see Section 9.9)

Day relative to notification of infection:	D1-14
**Study procedures**	
Symptom tracking via eDiary	daily
**Research Samples**	
Serum for SARS-CoV-2 clinical serology[1]	✓[2]

[1]For blood self-collected by a participant the maximum volume will be less than 1 mL in a single collection.
[2] Sample for serology will be collected as soon as possible following report of positive SARS-CoV-2 test

In addition, we may ask participants to sign a release to allow us to review their COVID-19 related medical records.

Appendix F Procedures for Prospective Close Contact (PCC) cohort

Visit:	**100**	**101**
Day:	D1	D113
Month:	M0	M4
	Enrollment	Follow-up
**Study procedures**		
Protocol consent (eConsent)	✓	–
Obtain demographics[1]	✓	–
SARS-CoV-2 screening at school, if applicable[1]	approximately 2x per week	
End of study questionnaire[1]	–	✓

[1]Will be captured via eDiary, please see Section 9.16.

If a PCC participant (or their Main study contact) tests positive for SARS-CoV-2, the following **additional** procedures will occur:

Day relative to notification of infection:	D1	D29
**Study procedures**		
Report of exposure[1]	✓	
Symptom tracking[1]	Daily for 14 days	–
**Research Samples**		
Serum for SARS-CoV-2 clinical serology[2]	✓	✓
Nasal swab[3]	Daily for 14 days	–

[1] Will be captured via eDiary , please see Section 9.16.
[2]For blood self-collected by a participant the maximum volume will be less than 1 mL in a single collection. Sample for serology will be collected as soon as possible following report of positive SARS-CoV-2 test.
[3]Swabs will be returned via drop-off location(s) specified by the clinic or through the mail. Nasal swabs will be collected as soon as possible following report of positive SARS-CoV-2 test.

In addition, we may ask participants to sign a release to allow us to review their COVID-19 related medical records.

Appendix G Procedures for Case-Ascertained Close Contact Cohort

Visit:	**200**	**201**	**202**
Day:	D1	D1-D3	D29
Month:	M0	M0	M1
	Enrollment	Initial specimen collection	Follow-up
**Study procedures**			
Protocol consent (eConsent)	✓	–	–
Collection of demographic information[1]	✓	–	–
Report of exposure[1]	✓	–	–
Symptom tracking[1]		Daily for 14 days	–
SARS-CoV-2 screening at the school if applicable[1]		approximately 2x per week	
End of study questionnaire[1]	–	–	✓
**Research Samples**			
Serum for SARS-CoV-2 clinical serology[2]		✓	✓
Nasal swab[3]		Daily for 14 days	–

[1]Will be captured via eDiary, please see Section 9.16.

[2] For blood self-collected by a participant the maximum volume will be less than 1 mL in a single collection. Sample for serology will be collected as soon as possible following report of positive SARS-CoV-2 test (ie, as early as Day 1).

[3]Swabs will be returned via drop-off location(s) specified by the clinic or through the mail. Nasal swabs will be collected as soon as possible following report of positive SARS-CoV-2 test (ie, as early as Day 1).

In addition, we may ask participants to sign a release to allow us to review their COVID-19 related medical records.

Appendix H Visit windows

Main Cohort, Immediate Vaccination, Visit Windows

Visit Number	Visit Type	Lower Allowable Window	Lower Target Day	Target Day[1]	Upper Target Day	Upper Allowable Window
01.0	Screening	-56	-		-	0
02.0	**Enrollment/Randomization**[2]	-	-	1	-	-
03.0	**Vaccination 2**	-	-3	29[3]	+7	+14
04.0	Month 2 Follow-up	-	-7	57	+7	+14
05.0	Month 4 Follow-up	-13	-7	113	+14	+56

1.Target dates are relative to Visit 2.0 (Enrollment/Randomization), except for cases described in footnote 3.

2. Completion of post enrollment activities may occur up to approximately 7 days post enrollment (see Section 9.3) .

3. In the event that Vaccination 1 occurs on a different day than Visit 2.0 (Enrollment/Randomization), Visit 3.0 target dates are relative to date of Vaccination 1.

Main Cohort, Delayed Vaccination, Visit Windows

Visit Number	Visit Type	Lower Allowable Window	Lower Target Day	Target Day[1]	Upper Target Day	Upper Allowable Window
01.0	Screening	-56	-		-	0
02.0	**Enrollment/Randomization**[2]	-	-	1	-	-
04.0	Month 2 Follow-up	-	-7	57	+7	+14
05.0	**Vaccination 1**	-13	-7	113	+14	+28
06.0	**Vaccination 2**[1]	-	-3	141	+7	+14

1. Target dates are relative to Visit 2.0 (Enrollment/Randomization), except visit 06.0, which is relative to visit 05.0.

2. Completion of post enrollment activities may occur up to approximately 7 days post enrollment (see Section 9.5) .

Case-Ascertained Close Contact (CACC) Cohort and applicable Prospective Close Contact (PCC) participants[1], Visit Windows

Visit Number	Visit Type	Lower Allowable Window	Lower Target Day	Target Day[2]	Upper Target Day	Upper Allowable Window
200.0	**Enrollment**	-	-	1	-	-
201.0	Initial blood specimen collection		-2	3	+1	+4
202.0	Day 29 Follow-up	-	-7	29	+14	+28

1. Applicable to PCC participants when they (or their Main study contact) tests positive for SARS-CoV-2.

2. Target dates are relative to Visit 200.0 (Enrollment).

Appendix I Protocol Signature Page

A randomized controlled study to assess SARS CoV-2 infection, viral shedding, and subsequent potential transmission in university students immunized with Moderna COVID-19 Vaccine

I will conduct the study in accordance with the provisions of this protocol and all applicable protocol-related documents. I agree to conduct this study in compliance with United States (U.S.) Health and Human Service regulations (45 CFR 46); applicable U.S. Food and Drug Administration regulations; standards of the International Council for Harmonisation of Technical Requirements for Pharmaceuticals for Human Use (ICH) Guideline for Good Clinical Practice (E6) (R2); Institutional Review Board/Ethics Committee determinations; all applicable in-country, state, and local laws and regulations; and other applicable requirements (eg, U.S. National Institutes of Health, Division of AIDS) and institutional policies.

Investigator of Record Name (print) Investigator of Record Signature Date

Protocol Number: CoVPN 3006

Protocol Version: Version 3.0

Protocol Date: April 12, 2021

COVID-19
Prevention Network

PROTOCOL

CoVPN 3006

A randomized controlled study to assess SARS CoV-2 infection, viral shedding, and subsequent potential transmission in individuals immunized with Moderna COVID-19 Vaccine

DOCUMENT ID 38822

Non-IND Study

CLINICAL TRIAL SPONSORED BY

National Institute of Allergy and Infectious Diseases (NIAID)
National Institutes of Health (NIH)
Department of Health and Human Services (DHHS)
Bethesda, Maryland, USA

May 20, 2021
Final
CoVPN 3006
Version 4.0

Contents

1 Overview 5
 1.1 Protocol Team 8

2 Background 10
 2.1 Rationale for trial concept 10
 2.2 Measures of vaccine efficacy 11
 2.3 Importance of understanding vaccine effects on infectiousness and transmissibility 13
 2.4 Limitations of the current Phase 3 trials for addressing unanswered questions 16
 2.5 Burden of SARS-CoV-2 infection and COVID-19 disease among students 18
 2.6 Age and Asymptomatic Infection 19
 2.7 Evidence for viral load as a predictor of infectiousness 20
 2.8 Evidence for viral load as a marker of COVID-19 disease severity and clinical course 21
 2.9 Methods for capturing transmission 21
 2.10 Moderna COVID-19 Vaccine 23
 2.11 Trial design rationale 24

3 Objectives and endpoints 28

4 Ethical considerations 32

5 IRB/EC review considerations 34
 5.1 Minimized risks to participants 34
 5.2 Reasonable risk/benefit balance 34
 5.3 Equitable participant selection 35
 5.4 Appropriate informed consent 35
 5.5 Adequate safety monitoring 35
 5.6 Protect privacy/confidentiality 36

6 Statistical considerations 37
 6.1 Accrual and sample size calculations 39
 6.2 Role of the Independent Data Monitoring Committee (IDMC) 44
 6.3 Randomization 45
 6.4 Statistical analyses 45

7 Selection and withdrawal of participants 54
 7.1 Inclusion criteria for Main Cohort, Immediate Vaccination Group and Standard of Care Group 54
 7.2 Exclusion criteria for Main Cohort, Immediate Vaccination Group and Standard of Care Group 55
 7.3 Inclusion criteria for Main cohort, Vaccine Declined Group 56
 7.4 Exclusion criteria for Main cohort, Vaccine Declined Group 56
 7.5 Inclusion criteria for Prospective Close Contact (PCC) cohort 57
 7.6 Inclusion criteria for Case-ascertained Close Contact (CACC) cohort 57

8 Study product 58
8.1 Vaccine regimen 58
8.2 Storage, handling, preparation, and administration 58
8.3 Acquisition of Moderna COVID-19 Vaccine 58
9 Clinical procedures 60
9.1 Informed consent 60
9.2 Pre-enrollment procedures for Main Cohort 62
9.3 Enrollment and vaccination visits for Main Cohort, Immediate Vaccination Group 62
9.4 Follow-up procedures for Main Cohort, Immediate Vaccination Group 63
9.5 Enrollment (visit 2) and Month 2 (visit 4) Visits for Main Cohort, Standard of Care and Vaccine Declined Groups 63
9.6 Month 4 (visit 5) and Month 5 (visit 6) Visits for Main Cohort, Standard of Care Group and Vaccine Declined Group 64
9.7 Procedures for Main Cohort participant diagnosed with a SARS-CoV-2 infection during the study 65
9.8 Enrollment and follow-up procedures for Prospective Close Contact (PCC) cohort 65
9.9 Enrollment and Follow-up procedures for Case-Ascertained Close Contact (CACC) cohort 66
9.10 Visit windows and missed visits 67
9.11 Discontinuing vaccination for a participant who receives a COVID-19 Vaccine outside the study 67
9.12 Early termination visit 68
9.13 Pregnancy for Main Cohort 68
9.14 SARS-CoV-2 monitoring by schools for Main Cohort, if applicable 68
9.15 COVID-19 symptom surveillance for Main Cohort 68
9.16 Use of electronic Diary (eDiary) for Clinical Outcomes Assessments 68
10 Laboratory 71
10.1 CRS laboratory procedures 71
10.2 Total blood volume 71
10.3 Endpoint assays 71
10.4 Lab assay portfolio 72
10.5 Exploratory studies 72
10.6 Specimen storage and other use of specimens 73
10.7 Biohazard containment 73
11 Safety Reporting 74
11.1 FDA/CDC Vaccine Adverse Event Reporting System (VAERS) 74
11.2 Reporting safety events to v-safe 75
12 Protocol conduct 76
12.1 Study termination 76
12.2 Emergency communication with study participants 77
13 Version history 78
14 Document references (other than literature citations) 84

15 Acronyms and abbreviations....86

16 Literature cited....88

Appendix A Sample informed consent form for Main cohort, Immediate Vaccination and Standard of Care Groups....93

Appendix B Sample informed consent form for Main cohort, Vaccine Declined Group 112

Appendix C Sample informed consent form for Prospective Close Contact (PCC) cohort 135

Appendix D Sample informed consent form for Case-Ascertained Close Contact (CACC) cohort....147

Appendix E Procedures for Main Cohort, Immediate Vaccination Group....159

Appendix F Procedures for Main Cohort, Standard of Care and Vaccine Declined Groups....161

Appendix G Procedures for Prospective Close Contact (PCC) cohort....163

Appendix H Procedures for Case-Ascertained Close Contact Cohort....164

Appendix I Visit windows....165

Appendix J Protocol Signature Page....166

1 Overview

Title

A randomized controlled study to assess SARS CoV-2 infection, viral shedding, and subsequent potential transmission in individuals immunized with Moderna COVID-19 Vaccine

Primary objective(s)

Primary objective 1:

To evaluate the efficacy of Moderna COVID-19 Vaccine against SARS-CoV-2 infection (ie, to evaluate vaccine efficacy against infection or VE_S)

Primary objective 2:

To evaluate the effect of Moderna COVID-19 Vaccine on peak nasal viral load as a measure of infection and a proxy of infectiousness

Study product and route of administration

Moderna COVID-19 Vaccine: a lipid nanoparticle (LNP) dispersion of a messenger ribonucleic acid (mRNA) encoding the prefusion stabilized S protein of SARS-CoV-2 formulated in LNPs composed of 4 lipids (1 proprietary and 3 commercially available). The Moderna COVID-19 Vaccine, also known as mRNA-1273, is a suspension for intramuscular injection administered as a series of two doses (0.5 mL each) 1 month apart.

This vaccine has been issued an Emergency Use Authorization (EUA) by the US Food and Drug Administration (FDA) for active immunization to prevent COVID-19 in individuals 18 years and older.

Table 1-1 Schema for the Main Cohort

		Injection schedule in months			
Group	N	0	1	4	5
Randomized Groups					
Immediate Vaccination[1]	Up to 6000	Moderna COVID-19 Vaccine	Moderna COVID-19 Vaccine	-	-
Standard of Care[2]	Up to 6000	-	-	Moderna COVID-19 Vaccine[3]	Moderna COVID-19 Vaccine[3]
Observational Group					
Vaccine Declined[4]	Up to 6000	-	-	-[4]	-[4]
Total	Up to 18,000				

[1] Immediate Vaccination Group: participants receive Moderna COVID-19 vaccine on enrollment and are given recommendations to follow federal, state, and local guidelines on COVID-19 precautions following vaccination, such as masking, physical distancing, isolation and quarantine.
[2] Standard of Care Group: participants are given recommendations to follow federal, state, and local guidelines on COVID-19 vaccination and COVID-19 precautions, such as masking, physical distancing, isolation and quarantine.
[3] Vaccine will be offered if participant has not received vaccine outside of the study.
[4] Vaccine Declined Group includes participants who prefer not to be vaccinated. If requested, participant will be offered vaccine if they have not received vaccine outside of the study

Participants

Main Cohort (main study participants)

Up to approximately 18,000 healthy volunteers, aged 18 through 29 years, who will be followed for up to 5 months after enrollment; up to 6,000 randomized to Immediate Vaccination Group, up to 6,000 randomized to Standard of Care Group, and up to 6,000 non-randomized to Vaccine Declined Group.

Prospective Close Contact (PCC) Cohort

Up to approximately 36,000 volunteers who are in frequent close physical proximity with main study participants

Case-Ascertained Close Contact (CACC) Cohort

Approximately 3 contacts per incident case. Actual number dependent on incidence rate. Up to approximately 2250 volunteers aged 18 years and older who have been in close contact with a SARS-CoV-2 positive case from the Main Cohort

Design

Multicenter, randomized, controlled, open-label trial augmented by an observational cohort

Duration per participant

Up to 5 months per participant for the Main Cohort and for the Prospective Close Contact Cohort. 1 month per participant in Case-Ascertained Close Contact Cohort.

Estimated total study duration

9 months (includes enrollment and follow-up).

Core operations

CoVPN Vaccine leadership Group/Core Operations Center, Fred Hutchinson Cancer Research Center (Fred Hutch) (Seattle, Washington, USA)

PPD, Inc. (Wilmington, North Carolina, USA)

Statistical and Data Management Center (SDMC)

Statistical Center for HIV/AIDS Research and Prevention (SCHARP), Fred Hutch (Seattle, Washington, USA)

Laboratory Center (LC)

Endpoint assay laboratories

- University of Washington Virology Laboratory (UW-VL) (Seattle, Washington, USA)
- University of Washington Virology Specialty Laboratory (UW-VSL) (Seattle, Washington, USA)
- Pioneer Hi-Bred International, Inc., a member of the Corteva Agriscience group of Companies (Johnston, Iowa, USA)
- Duke University Medical Center (Durham, North Carolina, USA)
- CoreMedica Labs (Lee's Summit, Missouri, USA)

Study sites

Clinical research sites (CRSs) across the US

Study monitoring

Independent Data Monitoring Committee (IDMC)

1.1 Protocol Team

Protocol leadership

Co-chairs

Kathryn Stephenson
Harvard University School of Medicine
Beth Israel Deaconess Medical Center
617-735-4556
kstephen@bidmc.harvard.edu

Audrey Pettifor
Department of Epidemiology
Gillings School of Global Public Health
University of North Carolina
919-966-7439
apettif@email.unc.edu

Jasmine Marcelin
Division of Infectious Diseases
University of Nebraska Medical Center
402-559-8650
jasmine.marcelin@unmc.edu

Protocol Lead Advisors

Larry Corey
CoVPN LOC, Fred Hutch
206-667-6770
lcorey@fredhutch.org

Myron Cohen
University of North Carolina
919-966-2537
myron_cohen@med.unc.edu

James Kublin
CoVPN LOC, Fred Hutch
206-667-1970
jkublin@fredhutch.org

Statisticians

Holly Janes
CoVPN SDMC, Fred Hutch
206-667-6353
hjanes@fredhutch.org

Elizabeth Brown
CoVPN SDMC, Fred Hutch
206-667-1731
erbrown@fredhutch.org

Laboratory lead

John Hural
CoVPN Laboratory Program
206-667-1683
jhural@fredhutch.org

Protocol Team leader

Nicole Grunenberg
CoVPN LOC, Fred Hutch
206-667-2043
ngrunenb@fredhutch.org

DAIDS Medical Officer

Catherine Yen
DAIDS, NIAID
240-292-4783
catherine.yen@nih.gov

Other contributors to the original protocol

Core medical monitor	Nicole Grunenberg<br>CoVPN LOC, Fred Hutch	*CoVPN Physician consultant*	Nzeera Ketter<br>CoVPN LOC, Fred Hutch
Laboratory consultants	Alexander Greninger<br>Robert Coombs<br>University of Washington Medical Center (UWMC)<br><br>Keith Jerome<br>Fred Hutch, UWMC	*Clinical safety specialists*	Megan Jones<br>Maija Anderson<br>CoVPN LOC, Fred Hutch
Laboratory center representatives	Nicole Espy<br>Lisa Sanders<br>Jen Hanke<br>CoVPN Laboratory Center, Fred Hutch	*Clinical trials manager*	Julie Hunt<br>CoVPN LOC, Fred Hutch
Regulatory affairs associate	Megan Brandon<br>CoVPN LOC, Fred Hutch	*Clinical data managers*	Melissa Cummings<br>Vicky Kim<br>CoVPN SDMC, Fred Hutch
Community Advisory Board (CAB) members	John Isaac<br>Boston University	*SDMC Associate Director of Operations*	Jessica Andriesen<br>CoVPN SDMC, Fred Hutch
Statistical research associates	Erika Rudnicki<br>Moni Neradilek<br>CoVPN SDMC, Fred Hutch	*SDMC Associate director of lab science*	April Randhawa<br>CoVPN SDMC, Fred Hutch
Community engagement unit representative	Francisco E Rentas<br>CoVPN LOC, Fred Hutch	*Data Management Director*	Kristine Donaty<br>CoVPN SDMC, Fred Hutch
Project coordinator	Kylie McCloskey<br>CoVPN LOC, Fred Hutch	*Protocol development managers*	Kajari Mondal<br>Meg Trahey<br>CoVPN LOC, Fred Hutch

2 Background

2.1 Rationale for trial concept

The United States has the most infections and deaths from SARS-CoV-2 (the virus that causes Coronavirus disease 2019 [COVID-19]) of any nation in the world (1). Fortunately, there is now hope that the large burden of COVID-19-related illness may lessen in 2021 due to the discovery that candidate COVID-19 vaccines, including the Moderna COVID-19 Vaccine, can prevent symptomatic COVID-19 and severe disease in adults (2, 3). Ongoing Phase 3 trials are not designed to estimate a vaccine's efficacy against all infections, with a critical gap being asymptomatic infections. The current trials are also not designed to evaluate vaccine effects on infectiousness as measured by viral shedding or on onward transmission of infection. For example, at an individual level, it is currently unknown if vaccinated individuals should continue to wear masks or social distance, or if they should be quarantined if exposed to COVID-19. At a policy level, it is unknown if vaccination should be required for certain settings, such as public transit including air travel, places of employment, or school. These questions are especially important given that SARS-CoV-2 is highly infectious compared to other respiratory illnesses such as the flu. Recent data also demonstrate that even individuals with asymptomatic COVID-19 can have high titers of nasal viral shedding and thus may be highly infectious. Furthermore, many individuals with asymptomatic infection develop low level and transient antibodies against SARS-CoV-2 (4), suggesting that trials relying on seroconversion as a marker of infection may miss infections.

While there are many populations at risk for SARS-CoV-2 infection, young people are particularly at risk of acquiring and transmitting SARS-CoV-2. Between August and September 2020, COVID-19 cases among young people aged 18-22 years increased 55% nationally (5) and during June-August 2020, young people aged 20-29 years had the highest incidence of disease in the US, accounting for >20% of all confirmed cases (6). Further, increases in SARS-CoV-2 infection among younger adults in the Southern US preceded increases among older adults by 4-15 days, which confirms the importance of younger adults spreading infection to older, more vulnerable populations (5). At the same time, individuals aged 18-29 account for a tiny percentage (0.5%) of all deaths due to COVID-19 and are very unlikely to develop severe disease (7). These reasons plus the congregate and social living situations of students and that many schools are regularly testing their student body make them an excellent population to examine. The proposed study is designed to evaluate comprehensively the vaccine efficacy profile of the Moderna COVID-19 Vaccine, including its effect on acquisition of infection including asymptomatic infection, on disease and severity of disease, on measures of infectiousness, and transmission of infection.

This study is a 2-arm randomized, open-label trial in adults aged 18 through 29 years augmented with an observational cohort that will compare those who are randomized to receive the Moderna COVID-19 Vaccine upon enrollment (Immediate Vaccination Group) to those who are randomized to receive the vaccine 4 months later (Standard of Care Group) and those who prefer not to be vaccinated (Vaccine Declined Group). **The study will capture all incident SARS-CoV-2 infections among study participants in real time, by establishing real-time testing for SARS-CoV-2 on study and augmenting with all available data from school testing or community testing.** Study participants will be asked to collect daily nasal swabs, to enable ascertainment of the timing of acquisition and to capture the full viral load trajectory for incident infections. To capture secondary transmission events, very close contacts of study participants will be enrolled who may undergo school or other community testing for incident infection over the duration of the study ('prospective contacts'); in addition, diagnosis of infection among a study participant will trigger recruitment of additional participant contacts ('case-ascertained close contacts'). When a study participant tests positive based on testing of study-collected nasal swabs, school testing, or community testing, both prospective contacts and case-ascertained close contacts will be asked to collect daily nasal swabs for 14 days to detect incident infection and also will have blood collected for serology. At the end of the trial, all participants in the Standard of Care and Vaccine Declined Groups will be offered vaccine if they have not already been vaccinated in their communities. The goal of the study is to report results to inform public policy including guidance on masking and physical distancing indoors.

2.2 Measures of vaccine efficacy

Ongoing Phase 3 COVID-19 vaccine trials are designed with the primary objective to evaluate vaccine efficacy against COVID-19 disease, written VE_D, and defined as the multiplicative reduction in risk of disease in the vaccine vs. placebo groups. COVID-19 disease is defined by a protocol-specified set of signs and symptoms of disease coincident with PCR-confirmed SARS-CoV-2 infection. While the reduction in risk of disease is arguably the most relevant vaccine effect to the individual participant, understanding the population effect of a vaccine requires considering the other effects of the vaccine (see Table 2-1) (8). The most fundamental effect of a vaccine is to reduce acquisition of infection, sometimes referred to as 'vaccine efficacy against susceptibility', written VE_S, and measured by the multiplicative reduction in PCR-confirmed infection in the vaccine vs. placebo group. A vaccine that reduces acquisition of infection will provide benefit to the individual (i.e. *direct* effects), as well as benefit to the population by reducing the number of infected and therefore potentially infectious individuals who may expose and infect unvaccinated individuals in the population (i.e. *indirect* effects). Modeling studies suggest that COVID-19 vaccines that reduce acquisition are predicted to have considerably greater population impact, not only

reducing disease, hospitalizations, and mortality, but reducing transmission of infection and thereby magnifying their effects (9, 10).

An additional potential indirect effect of a vaccine is its effect on reducing secondary transmission of infection, written VE_I and defined as the multiplicative reduction in the rate of secondary transmission among vaccine vs. placebo individuals who become SARS-CoV-2 infected. A vaccine might reduce transmission through reducing the magnitude and/or duration of viral shedding, by reducing symptoms associated with transmission such as coughing, or by selectively blocking infection with less transmissible viral strains. Evaluating the effect of a COVID-19 vaccine on transmissibility is critical because a vaccine with high efficacy against COVID-19 disease but low efficacy against SARS-CoV-2 infection would necessarily yield a net increase in the number of asymptomatic infections. If individuals who become asymptomatically infected despite having received the vaccine are still infectious, and given that asymptomatic individuals are less likely to be diagnosed and self-isolate, such a vaccine has the potential to increase transmission and prolong the pandemic (2, 3).

As we prepare this study, we recognize there are other approaches to measuring population level benefit (eg, vaccine effect on infection and transmissibility). These approaches include: observational studies, such as those being conducted in Israel to examine population benefit of the vaccine and those planned by the CDC to examine transmission from vaccinated individuals to household contacts in longitudinal cohorts; and ancillary studies with frequent swabbing on a subset of participants in Phase 3 studies. However, none of these studies will allow for the rigorous capture, through daily nasal swabbing, of the early dynamics of viral replication and the capture of transmission events during the acute period of infection in a randomized trial.

All of these vaccine salutatory effects may depend on a variety of individual characteristics such as age, prior infection with SARS-CoV-2; on virological characteristics; and in the case of VE_I which conditions on infection, may depend on clinical characteristics such as symptomatology.

As will be discussed below in detail, the ongoing Phase 3 trials evaluate VE_D and partially identify VE_S, but do not allow direct estimation of VE_I. In contrast, the proposed study will collect data to enable evaluation of all three vaccine efficacy parameters in the participant population. Importantly, the use of an open-label Standard of Care Group as opposed to a blinded placebo arm, implies that the various effects evaluated will capture not only biological vaccine effects, but also potential behavioral effects associated with vaccination, including potential behavioral disinhibition. Behavioral effects most plausibly influence exposure and therefore infection, but knowledge of vaccine receipt is viewed as less likely to influence disease, viral load, and secondary transmission.

Table 2-1 Population measures of vaccine efficacy that will be evaluated using the proposed study, where a Standard of Care Group will be used instead of placebo

Efficacy measure	Definition
Vaccine efficacy against SARS-CoV-2 infection (VE_S)	The multiplicative reduction in SARS-CoV-2 infection as measured by PCR of nasal swabs, vaccine vs. placebo
Vaccine efficacy against COVID-19 disease (VE_D)	The multiplicative reduction in symptomatic COVID-19*, vaccine vs. placebo
Vaccine efficacy against viral replication (VE_{VL})	The reduction in SARS-CoV-2 viral burden as measured by PCR of nasal swabs, vaccine vs. placebo
Vaccine efficacy against transmission (VE_I)	The multiplicative reduction in rate of SARS-CoV-2 transmission from infected individuals on the vaccine arm relative to the placebo arm

*Defined by a protocol-specific set of signs and symptoms defined in Section 3.

2.3 Importance of understanding vaccine effects on infectiousness and transmissibility

The most effective way for a vaccine to prevent onward transmission of infection is to prevent acquisition of infection. Vaccine efficacy against infection, VE_s, is a measure of this effect (8). However, a vaccine that does not completely block acquisition of infection may still reduce onward transmission among individuals who become infected despite having received the vaccine, as measured by VE_I (8, 11). As discussed above, understanding the effect of a COVID-19 vaccine on transmission is critical because a vaccine with high efficacy against COVID-19 disease but low efficacy against SARS-CoV-2 infection would necessarily yield a net increase in the number of asymptomatic infections. If individuals who become asymptomatically infected despite having received the vaccine are still infectious, and given that asymptomatic individuals are less likely to be diagnosed and self-isolate, such a vaccine has the potential to increase transmission and prolong the pandemic (2, 3).

If a COVID-19 vaccine did reduce infectiousness, mathematical modeling suggests that much greater population impact could be achieved. Deterministic compartmental models fit to data from King County, Washington and envisioning vaccine rollout starting December 1, 2020 to 18% of the adult population proportionally across age groups, suggest that while a vaccine that reduces COVID-19 disease by 90% and SARS-CoV-2 infection by 10% reduces total infections in 2021 by 18.1% and deaths by 27.5%, if the vaccine additionally reduces infectiousness by 50% the impact on infections would more than double (to 49.3% reduction) and deaths would be cut in half (to 50.9% reduction) (Swan and Dimitrov et al, in preparation; Figure 2-1).

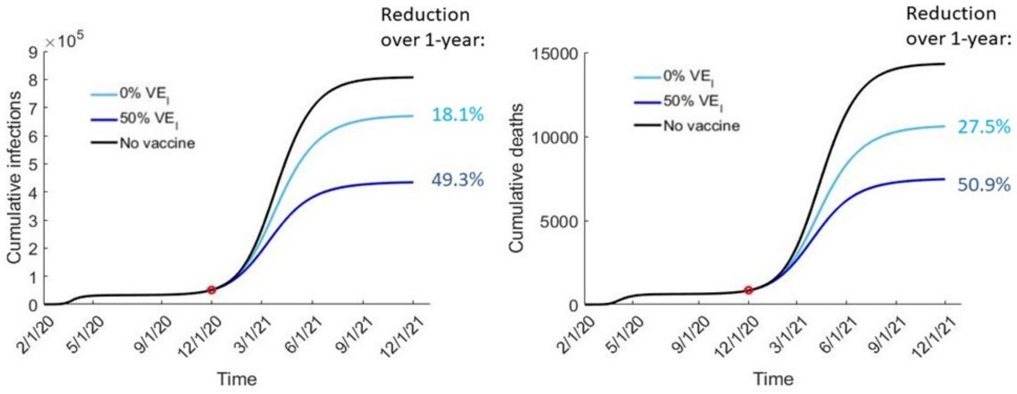

Figure 2-1 COVID-19 vaccine that reduces infectiousness by 50% has greater impact on infections and deaths. Deterministic compartmental model calibrated to King County, WA stratified by age, SARS-CoV-2 infection status, COVID-19 treatment status and vaccination. 800,000 doses vaccine (for 18% of population) starting Dec 1, 2020, allocated proportionally across age groups. Absent vaccination, 20% of infections are asymptomatic; asymptomatic infections are 28% less infectious than symptomatic infections, but more transmissible due to lower rates of diagnosis and quarantine. Both vaccines reduce COVID-19 disease by 90% and SARS-CoV-2 infection by 10%. While one vaccine (light blue) does not reduce infectiousness among individuals who become infected, the other vaccine (dark blue) reduces infectiousness by 50%. As a result, it achieves a much greater reduction in both infections and deaths. (Swan, Dimitrov et al, in preparation).

In the context of limited vaccine supply, mathematical modeling also suggests that optimal allocation of vaccine depends on how well a vaccine reduces infectiousness. Take, for instance, a deterministic compartmental model calibrated to Washington State that uses the following assumptions: that, absent vaccination, 40% of infections are asymptomatic, asymptomatic infections confer equal immunity and are equally infectious, and that there is sufficient vaccine supply for 30% of the population. For a vaccine that reduces COVID-19 disease by 90% but has no effect on infection or infectiousness, the limited supply of vaccine should be allocated to older adults who have a high burden of disease in order to minimize deaths and peak hospitalizations (Figure 2-2). However, if the vaccine also reduces infectiousness by 60%, minimizing peak hospitalizations is achieved by allocating limited vaccine to the young, while minimizing deaths still requires vaccinating older adults. These results again highlight the critical importance of understanding vaccine effects on acquisition and infectiousness in order to make policy decisions.

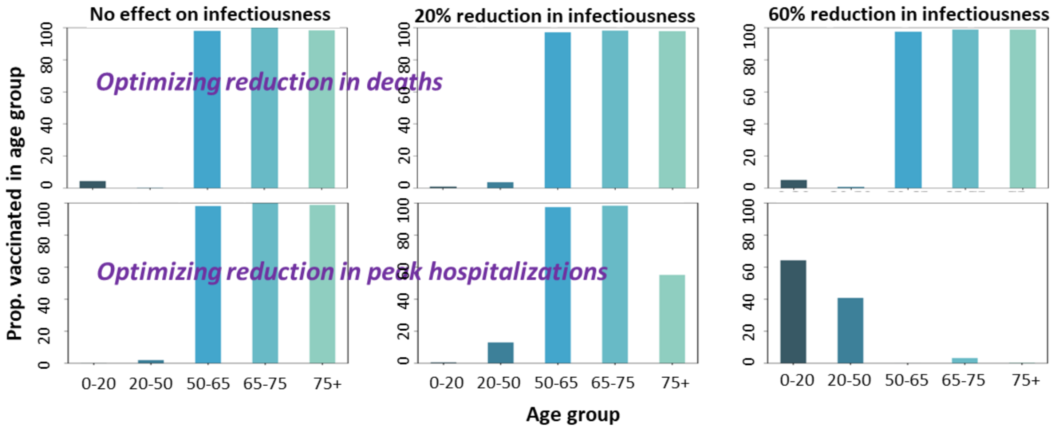

Figure 2-2 Deterministic compartmental model calibrated to Washington State (7.6M people), reflecting US demographics. Model assumes 4.56M doses vaccine (for 30% of population) assuming 10% of the population is previously infected and immune and there are 1000 current infections. Absent vaccination, 40% of infections are asymptomatic; asymptomatic infections confer equal immunity (for at least 6 mo. duration) and are equally infectious. (Matrajt et al, in preparation)

Non-human primate (NHP) data also suggests that elimination of viral shedding by a COVID-19 vaccine is not guaranteed. Table 2-2 shows the comparison of NHP data between candidate COVID-19 vaccines in current Phase 3 trials. While most of the vaccines were shown to prevent viremia within the lower respiratory tract (bronchoalveolar lavage fluid), and thus improve markers of disease severity, three out of the four platforms tested did not eradicate nasal viral shedding in all cases.

Table 2-2 Comparison of NHP data between Janssen Ad26, Moderna RNA, AstraZeneca ChimpAdOx1 and Novavax COVID-19 vaccines

	**Ad26 One dose 1 x10^{11}**	**mRNA (4 weeks post-dose 2)**	**AstraZeneca/Oxford 2.5x10^{10} 0, 4 weeks**	**Novavax 2 doses (0,21) 5 mcg, 50 mcg**
**Pseudovirus neutralization (GMT)**	408	1,862	5 – 40<br>boost 10 - 160	No
**Live virus assay**	113	3,481		16,026
**Spike protein**	15,000 (not shown)	36,186 (AUC)		335,017
**Lung BAL protection**	Complete	Complete at day 7	75% both prime and boost	Complete
**Nasal protection**	5/6 (83%)	7/8	0%	Complete (12/12)
**T-cell immune response**	Some CD8	No CD8+ T cells	Not reported	CD4 and CD8

Finally, not understanding relative infectivity is a huge gap in translating vaccine use to public health messaging. The demonstration of reduced infectivity may reduce vaccine hesitancy. Public health, medical personnel and organizations may embrace vaccination, as well as users, if the vaccine both provides individual as well as family/community benefit. For individuals, knowing that a vaccine reduces infectivity to household and personal contacts is often a prime motivator for vaccination. Data from this trial can facilitate this messaging as well as help decision-making among individuals about interactions with household members and attending work and school.

2.4 Limitations of the current Phase 3 trials for addressing unanswered questions

Currently, none of the current Phase 3 vaccine efficacy trials will enable robust estimation of vaccine effects on infectiousness. The primary objectives for all trials are to evaluate vaccine efficacy (VE) against COVID-19 disease. Secondary objectives include evaluating VE against seroconversion using infrequent serology (every 1-6 months over 1 year, with more frequent sampling over the first 3 months), but this will not fully capture all infections including those that are asymptomatic. Estimates based on acute infection cohorts (12) suggest that 12-50% of asymptomatic infections might be missed using this sampling, leading to an unknown magnitude and direction of bias in the estimate of VE_S. Data from Long et al. found that 40% of individuals with asymptomatic RT-PCR confirmed SARS-CoV-2 infections were IgG negative at 8 weeks after the acute phase of infection (Figure 2-3) (4). Further, it has been observed that asymptomatic cases have lower IgG titers across all antigens (including NP) (Figure 2-4) (13). In

current trials, infectiousness is not captured robustly among those who become infected; viral load (VL) is only measured for those who are symptomatic and only after the onset of symptoms. Data from the Moderna trial where nasal swabs were collected at the time of vaccine administration on days 0 and 28 among all participants suggest that the vaccine may reduce the level of nasal carriage of virus (14). However, one time point does not robustly capture the full viral dynamics of infection, in particular, asymptomatic infections. The data are suggestive that the vaccine may reduce viral carriage (15). The level of viral shedding prior to the onset of symptoms, when most transmission occurs (16), will not be captured for any infected participants. Importantly, no data in current trials are collected on onward transmission of infection and therefore VE_I cannot be evaluated directly.

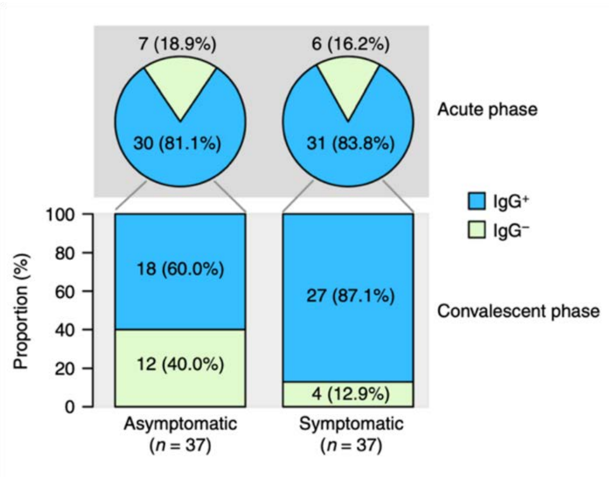

Figure 2-3 40% of asymptomatic individuals were IgG negative by 8 weeks post-acute infection (Long et al.(4).

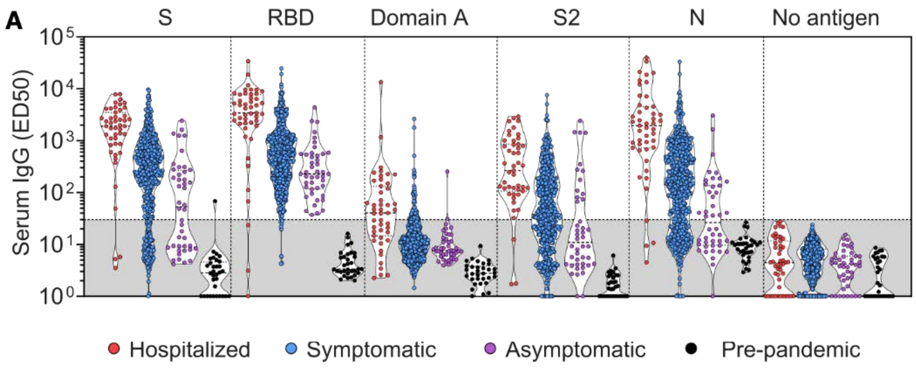

Figure 2-4 Asymptomatic Patients with COVID-19 have Lower IgG Titers Across Multiple Antigens Among Italian Medical Staff N>600 (13).

2.5 Burden of SARS-CoV-2 infection and COVID-19 disease among students

The study will be conducted among eligible adults 18 through 29 years of age including students enrolled in post-secondary educational institutions (eg, universities, vocational and technical colleges) because these are ideal locations for studying SARS-CoV-2 transmission clusters. Large numbers of cases of COVID-19 have been reported on numerous campuses throughout the US as colleges and universities re-opened in fall 2020. More than 397,000 cases were counted at 1,800+ colleges and universities in fall 2020 (17, 18). Over the time period July-December 11th, 2020, more than 85 colleges and Universities had more than 1,000 cases. Among these 49 campuses the cumulative attack rate from July to October was on average 6.2%. These estimates are somewhat uncertain given the fact that the number of reported cases for some schools includes faculty,

staff and graduate students; however, the number of such individuals is very small in comparison to the number of undergraduates.

Among 15 Association of American Universities (AAU) universities, and using data from the start of semester through September 18, 2020, there was an estimated 3.7% 1-month attack rate. The attack rate appeared to decline over time; it was 1.7% over September 19 to October 20, 2020. Attack rates may be underestimated because the denominator is assumed to be all undergraduates, whereas some schools had only a subset of students on campus. These statistics suggest that a conservative assumption of SARS-CoV-2 incidence among US university students for Fall semester 2020 is 6%. A conservative incidence assumption for Spring and Summer 2021semesters, anticipating potential improvements in testing, contact tracing, social distancing measures, as well as vaccine rollout to priority populations outside the universities, is therefore 3-6%.

The outbreaks of SARS-CoV-2 infection at college campuses have been reported to be associated with students residing in high density housing, socializing off campus, and not following social distancing guidelines (19). In the US, it is estimated that 45 percent of young adults aged 18-22 years were enrolled in colleges and universities in 2019 (16). As such, much of the increase in cases this summer and fall are attributed to college students. The college campuses are associated with a high rate of SARS-CoV-2 infection and resulting asymptomatic carriage due to huge impulse to socialize as well as no fear of severe disease among the young students.

2.6 Age and Asymptomatic Infection

For this study, participants will be enrolled who are 18 through 29 years of age. This age range is chosen because of the high incidence of asymptomatic infection in this population. A considerable body of data suggest that SARS-CoV-2 can be spread by individuals with and without symptoms (20-23). VL data among individuals with acute SARS-CoV-2 infection also indicate that peak VL usually occurs before disease onset (22, 24). This suggests that there is a substantial transmission potential before symptom onset. According to the latest Centers for Disease Control and Prevention (CDC) estimate 40% of SARS-CoV-2 infections are asymptomatic (25). Available data indicate that younger individuals appear more likely to remain asymptomatic following infection than older individuals (26, 27). A recent study done in the UK showed about 80% of children between 10-19 years of age remain asymptomatic (28). As infected young students are less likely to show clinical symptoms, the number of clinical cases reported among young populations would be lower, but the asymptomatic young students could still be capable of transmitting the virus to others (29).

2.7 Evidence for viral load as a predictor of infectiousness

Mathematical modelling in human immunodeficiency virus (HIV) and herpesviruses and now COVID-19 suggests a significant percentage of transmission occurs from high peak VL which in all these diseases is during the asymptomatic period. Models by Josh Schiffer and Ashish Goyal suggest that a person has a 50% chance of infection with exposure to 10^7 SARS-CoV-2 RNA and <5% chance of infection with exposure to 10^5 SARS-CoV-2 RNA (Figure 2-5) (21). The model by Goyal et al (21) suggests that vaccine reduction in peak VL may reduce infectiousness. A 0.6 log reduction corresponds to ~50% VE_I and a 2.5 log reduction to ~90% VE_I (Figure 2-6). This suggests collecting nasal swabs every other day would be required to measure the vaccine effect on peak VL and area under the VL curve. Asymptomatic and pre-symptomatic cases of SARS-CoV-2 appear to play an important role in the onward transmission of the virus.

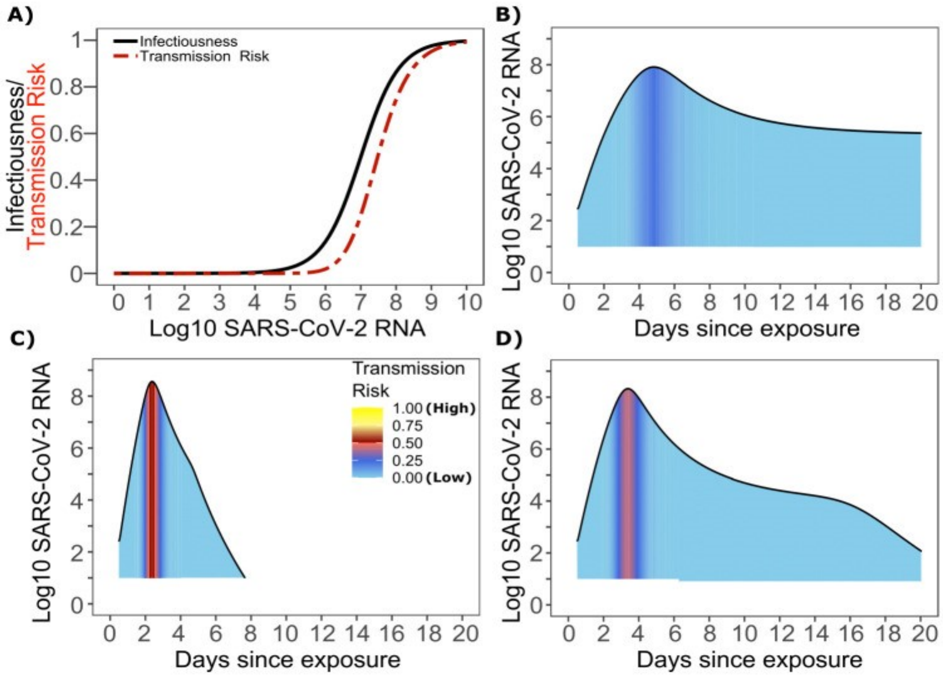

Figure 2-5 SARS-CoV-2 transmission probability as a function of shedding. A. Optimal infectious dose (ID) response curve (infection risk = Pt) and transmission dose (TD) response curve (transmission risk = Pt * Pt) curves for SARS-CoV-2. Transmission probability is a product of two probabilities, contagiousness and infectiousness. B-D. Three simulated viral shedding curves. Heat maps represent risk of transmission at each shedding timepoint given an exposed contact with an uninfected person at that time.

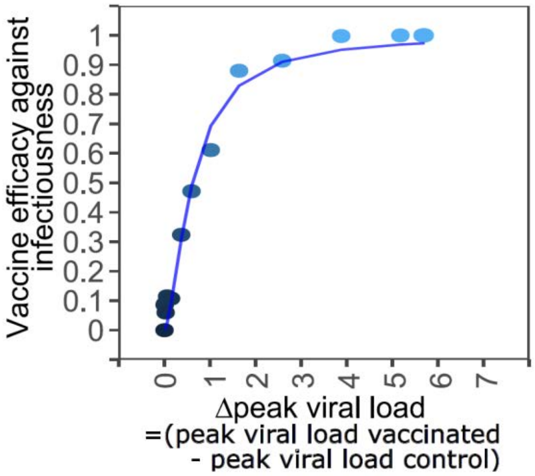

Figure 2-6 Vaccine efficacy against infectiousness versus the change in peak viral load. Model by Goyal et al (personal communication).

2.8 Evidence for viral load as a marker of COVID-19 disease severity and clinical course

Beyond infectiousness, there is a growing body of evidence supporting a linkage between VL and COVID-19 disease outcome. Two Phase 2/3 trials of monoclonal antibodies for COVID-19 treatment in outpatient settings found that VL at enrollment and early after intervention was associated with future clinical outcomes and disease course (30, 31). In the BLAZE-1 trial of intravenous infusion of the LY-CoV555 antibody, on day 7 post-infusion the frequency of hospitalization among patients with a high VL (Ct value of less than 27.5) was 12% (7 of 56 patients), as compared to 0.9% (3 of 340 patients) among those with a lower VL. Numerous observational studies have also identified associations between VL and clinical outcomes (32). The highest quality evidence comes from large cohort studies. In a large hospitalized cohort, Pujadas et al (33) estimated a highly significant 7% increase in mortality per log10 increase in VL at admission, after adjusting for other factors associated with clinical outcomes ($p = 0.0014$, multivariate Cox model). Chen et al (34) confirmed this finding in a larger cohort of patients admitted to three New York City hospitals. These data suggest that VL at and soon after the time of symptom onset may be a useful measure of disease severity.

2.9 Methods for capturing transmission

Reducing transmission is key to ending/controlling the COVID-19 pandemic. Due to vaccination, the number of asymptomatic individuals might increase as vaccine-induced immune responses may lead to VL reduction. However, it is still to be discovered whether those individuals will continue to shed and transmit

virus to others. Hence, it is important to identify secondary transmissions from study participants to estimate secondary infections rates by arm and to validate VL as a surrogate for transmission risk. Onward transmission of SARS-CoV-2 infection from study participants can be identified in combination with sequence-analysis and phylogenetics methods (35).

Estimating vaccine efficacy in reducing transmission (VE_I), requires measuring transmission of SARS-CoV-2 from vaccinated and unvaccinated individuals to others (36, 37). Measuring transmission of infected individuals to uninfected individuals is challenging, especially for transient infections where it may be more difficult to ascertain which infection occurred first. For example, because HIV is not transient and per-exposure transmission is low, resulting in a potentially long time period of exposure, transmission can be studied in sero-discordant sexual partnerships. In a transient infection, ideally both members of the transmission pair are monitored before either is infected so that timing and direction of transmission can be easily determined. Prospectively following a cohort of vaccinated and unvaccinated individuals and their close contacts to observe whether infection is transmitted from study participants to close contacts, or from contacts to study participants, based on frequent testing of both allows for better quantification of the temporality of events regarding infection transmission, if it occurs. To do this, Datta et al. propose augmenting a randomized, controlled vaccine trial with a cohort of close contacts of trial participants. Both trial participants and the contact cohort are followed prospectively for acquisition, allowing for estimation of VE_I. Another approach to capturing transmission events is a case ascertainment design (38, 39). Under this design, contacts of index cases are enrolled and tested as the cases emerge over time. A similar case ascertainment design was used for a recent household study for SARS-CoV-2 prevention. In the HCQ PEP trial, which was a household-randomized, double-blind, controlled trial of hydroxychloroquine postexposure prophylaxis to prevent acquisition of SARS-CoV-2 among close contacts recently exposed to persons diagnosed with SARS-CoV-2 infection, individuals were self-referred from infected individuals and enrolled online and all study procedures were done remotely including informed consent and daily self-nasal swabbing for 14 days (40). A case-ascertained approach is more resource-efficient than a prospective contact approach which requires enrollment of a large number of contacts, most of whom will not become infected nor have an infected index case in the main study. Because contacts are only identified and tested after index case diagnosis, the case-ascertained approach also enables assessment of secondary transmission in contacts not participating in frequent, prospective testing programs. However, the case-ascertained approach has the potential to miss or misclassify transmission events given the gap between diagnosing an index case and identifying, enrolling, and testing a contact. Index cases may also experience stigma when identifying contacts at the time of diagnosis, as opposed to when identifying contacts prospectively and before infection. The feasibility of recruiting and retaining contacts of young adults, and of classifying transmission events among contacts,

using the prospective and case-ascertained designs has yet to be evaluated and compared.

This study will employ both the augmented and case ascertainment approaches to studying transmission through enrollment of two contact cohorts, a Prospective Close Contact (PCC) cohort and a Case-Ascertained Close Contact (CACC) cohort. The PCC Cohort will be identified at enrollment as close and sustained contacts of main study participants (eg, roommates or co-workers). Participants in the PCC cohort may undergo routine screening for SARS-CoV-2 infection as part of their attendance at their school or location of employment, which may allow the participant and study team to receive real-time clinical diagnosis of COVID-19 during the study. Additionally, when a positive case is identified for a main study participant, the participant ('index case') will identify and refer their recent close contacts for enrollment into the CACC cohort. For both cohorts, infection diagnosis of the associated main study participant will trigger intensive short term sample collection (with a potential delay for the CACC cohort due to the need for informed consent and enrollment) including sera followed by daily nasal swabs for 14 days. Assessment of viral sequences, viral load trajectories, antibody detection and epidemiologic information from the infected participant and any positive close contacts in the PCC or CACC will aid in identifying transmission events between the participant and the contact. Details of the enrollment and procedures for both contact cohorts are described below.

2.10 Moderna COVID-19 Vaccine

This trial will utilize vaccine obtained under EUA by the US Government's COVID-19 Vaccine Response and allocated for this trial. Participants may also access Moderna COVID-19 EUA vaccine via other sources (see Section 8.3).

2.10.1 Clinical studies of Moderna COVID-19 Vaccine

Table 2-3 Summary of clinical studies

ClinicalTrials.gov Identifier	Study number	Phase	N	Dose groups	Route	Schedule	Reference
NCT04283461	P101	1	120	25, 50, 100, or 250 mcg	IM	M0, M1	(41, 42)
NCT04405076	P201	2	600	50 or 100 mcg	IM	M0, M1	(14, 15, 43)
NCT04470427	P301	3	30,351	100 mcg	IM	M0, M1	(14, 15, 43)

2.10.2 Potential risks of Moderna COVID-19 Vaccine

Risks associated with receipt of the Moderna COVID-19 Vaccine are described in the "Fact Sheet For Healthcare Providers Administering Vaccine (Vaccination Providers) Emergency Use Authorization (EUA) Of The Moderna COVID-19 Vaccine To Prevent Coronavirus Disease 2019 (COVID-19)", available at https://www.modernatx.com/covid19vaccine-eua/.

2.11 Trial design rationale

We are proposing a 2-arm randomized, controlled, open-label trial, augmented with an observational cohort. Individuals indifferent to receipt and timing of COVID-19 vaccination will be randomized to immediate vaccination at Months 0 and 1, or standard of care with vaccination given at Months 4 and 5, if not received off-study previously. Individuals who prefer not to be vaccinated will be enrolled in an observational 'Vaccine Declined' group; all these individuals are considered part of the 'Main cohort' (Figure 2-7). The design also includes two cohorts of contacts of main study cohort participants – the Prospective Close Contact (PCC) cohort and the Case-Ascertained Close Contact (CACC) cohort – to allow capture of secondary transmission events.

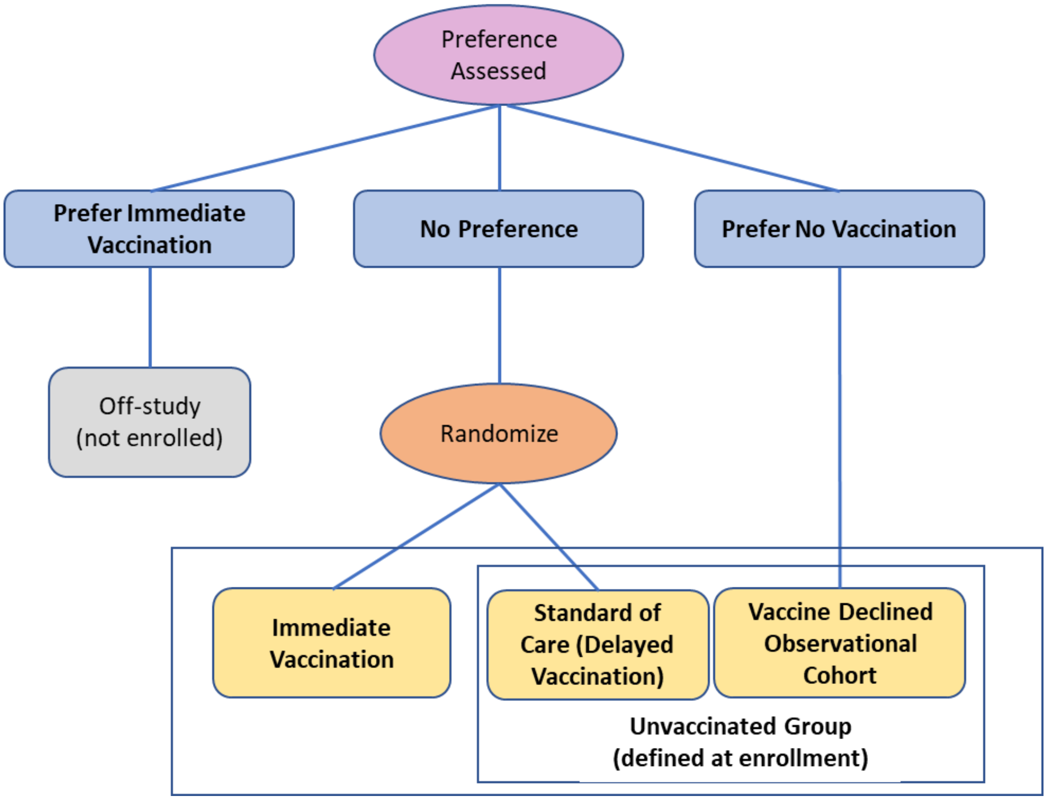

Figure 2-7 Main cohort study design

Participants in the main study will self-collect nasal swabs daily in order to capture all incident SARS-CoV-2 infection events over 4 months of follow-up and to capture the full course of viral shedding – from onset of infection to viral clearance – among those with both asymptomatic and symptomatic infection.

To minimize participant burden and maximize study efficiency, as many study procedures as possible will be conducted remotely (not requiring an in-person visit) and electronically, such as initial screening and consent. Participants diagnosed with SARS-CoV-2 infection will complete daily e-diaries to capture symptoms.

Given that primary analyses will be conducted among baseline seronegative participants, the rate of baseline SARS-CoV-2 seropositivity will be monitored operationally with an operational target of no more than 10% for the main study cohort.

2.11.1 Dose and schedule

The latest information regarding Moderna COVID-19 Vaccine dose and administration instructions can be found at https://www.modernatx.com/covid19vaccine-eua/.

For this study, vaccine will be administered at D1 and D29 for the immediate vaccination arm, and at D113 and D141 for the Standard of Care Group (if they have not been vaccinated outside the study). Vaccine will also be offered to the Vaccine Declined Group at D113 and D141.

2.11.2 Choice of Standard of Care Group

There is no licensed SARS-CoV-2 vaccine currently available to serve as a reference control. Currently, there are several SARS-CoV-2 vaccines available in the US under Emergency Use Authorization (EUA), including the Moderna COVID-19 Vaccine being used in this study. Accordingly, a Standard of Care Group has been chosen as an internal control. No placebo will be used, and participants will not be blinded to randomization assignment. The open-label design has been chosen primarily to achieve operational efficiency and to minimize resource utilization. Main study participants will be asked throughout the study about the timing and type of any outside vaccination, and contacts in the PCC and CACC will be queried about COVID-19 vaccine receipt. Main study participants receiving outside vaccination will remain on-study and follow the same study procedures after vaccination. Section 6.4.3.1 describes how the analysis of vaccine efficacy will take into account outside vaccination.

Randomized participants will not be blinded to randomization assignment. The open-label design has been chosen primarily to achieve operational efficiency and to minimize resource utilization. With an open-label design, differences between the randomized arms in study endpoints including SARS-CoV-2 infection, viral load, and secondary transmission will capture not only biological effects of vaccination, but potentially behavioral effects influencing exposure, including adherence to study procedures. This is most relevant for the SARS-CoV-2 infection endpoint: if participants who are randomized to immediate vaccination engage in more risk-taking behavior and thus experience higher exposure to SARS-CoV-2 relative to participants randomized to delayed vaccination, ie, there is behavioral disinhibition, this will be captured in the estimate of vaccine efficacy against infection: the biological reduction in incidence of SARS-CoV-2 infection attributable to vaccination will be offset by an increase in incidence due to higher exposure. Therefore, the measure of vaccine efficacy against infection that is estimated in this study is in truth a measure of effectiveness. However, we retain the terminology "vaccine efficacy" for simplicity. Section 6.4.3.1 discusses this issue in greater detail, including how the statistical analysis will attempt to control for potential imbalances in SARS-CoV-2 exposure between treatment arms when evaluating vaccine efficacy, and how both efficacy and effectiveness measures will be evaluated and compared. We note that endpoints measured after diagnosis of SARS-CoV-2 infection, including SARS-CoV-2 viral load and secondary transmission, are viewed as much less likely to be influenced by participant behavior and knowledge of vaccine receipt.

2.11.3 Rationale for Vaccine Declined Group

A cohort of individuals who prefer not to receive COVID-19 vaccination and decline randomization will be enrolled as a second internal control group in addition to the Standard of Care arm. The purpose of this group is to provide supplementary information on study endpoints, including SARS-CoV-2 incidence, SARS-CoV-2 viral load, and secondary transmission, absent COVID-19 vaccination. The 'Vaccine Declined' group will be enrolled concurrently with the randomized study participants, and at the same study sites, and in this fashion variability in the endpoints due to space and time will be minimized. Section 6.4 and the Statistical Analysis Plan will detail methods that will be used to control for other potential differences between participants who are randomized versus those in the Vaccine Declined group.

3 Objectives and endpoints

Primary Objectives	Primary Endpoints
1. To evaluate the efficacy of Moderna COVID-19 Vaccine against SARS-CoV-2 infection (ie, to evaluate vaccine efficacy against infection or VE_S).	SARS-CoV-2 infection diagnosed by PCR among participants who were SARS-CoV-2 seronegative at enrollment
2. To evaluate the effect of Moderna COVID-19 Vaccine on peak nasal viral load as a measure of infection and a proxy of infectiousness	Peak viral load in nasal samples from participants diagnosed with SARS-CoV-2 infection among participants who were SARS-CoV-2 seronegative at enrollment
**Secondary Objectives**	**Secondary Endpoints**
1. To evaluate the impact of Moderna COVID-19 Vaccine on secondary transmission of SARS-CoV-2 infection, using data for the Prospective Close Contact (PCC) cohort and Case-Ascertained Close Contact (CACC) cohort (ie, to evaluate vaccine efficacy against infectiousness or VE_I)	Number of adjudicated* secondary transmission events in PCC and CACC cohorts from main study participants who were SARS-CoV-2 seronegative at enrollment.<br>*based on questionnaire-based epidemiologic data, antibody and viral dynamics, and viral sequence data
2. To evaluate the efficacy of Moderna COVID-19 Vaccine to prevent serologically confirmed SARS-CoV-2 infection	SARS-CoV-2 antibodies to the nucleocapsid protein post visit 3 (month 1), among participants who were SARS-CoV-2 seronegative at enrollment
3. To evaluate vaccine efficacy against COVID-19 disease, (ie, to evaluate VE_D) using weekly e-diaries and confirmatory PCR testing	SARS-CoV-2 infection confirmed by PCR among participants who were SARS-CoV-2 seronegative at enrollment, coincident with the following symptoms:<br>• at least TWO of the following systemic symptoms: Fever (≥ 38ºC), chills, myalgia, headache, sore throat,<br>OR<br>at least ONE of the following signs/symptoms: cough, shortness of breath or difficulty breathing, new olfactory or taste disorder, clinical or radiographical evidence of pneumonia, thromboembolic event, myocardial infarction, myocarditis, chilblains, or multi-inflammatory syndrome

4. To evaluate vaccine effect on additional measures of magnitude of viral load over time, e.g. area under the viral load curve, duration of shedding, and time to log10 viral load above 10^5 copies/mL	Summary measures of the viral load curve, all evaluated among participants diagnosed with SARS-CoV-2 infection who were SARS-CoV-2 seronegative at enrollment:<br>• Transmission potential endpoint, defined as 0 for an individual without SARS-CoV-2 infection, and peak log10 viral load, for an individual with SARS-CoV-2 infection<br>• Area under log10 viral load curve (AUC)<br>• Duration of viral shedding (viral load above assay limit of detection)<br>• Area under log10 viral load curve above 10^5 copies/mL<br>• Duration of viral shedding above 10^5 copies/mL<br>• Time from enrollment to viral load above 10^5 copies/mL
5. To evaluate vaccine efficacy against SARS-CoV-2 infection, the vaccine effect on viral load, and the vaccine effect on secondary transmission in all enrolled participants without regard to baseline SARS-CoV-2 serostatus	• SARS-CoV-2 infection diagnosed by PCR [Primary endpoint 1]<br>• Peak log10 viral load in nasal samples from participants who are diagnosed with SARS-CoV-2 infection [Primary endpoint 2]<br>• Number of secondary transmission events in the PCC and CACC [Secondary endpoint 1]
6. To evaluate the immunogenicity of the vaccine	Magnitude and response rate of immune responses to vaccination measured at Months 0, and 2 stratified by baseline SARS-CoV-2 serostatus as measured by binding antibody and neutralization assays in the case-cohort immunogenicity set
7. To evaluate immune responses 1 month post-second vaccination as correlates of risk of SARS-CoV-2 acquisition, viral load, secondary infection, and COVID-19 disease among vaccine recipients in the Immediate Vaccination Group	Magnitude and response rate of immune responses to vaccination in the Immediate Vaccination Group and measured at Months 0 and 2, stratified by baseline SARS-CoV-2 serostatus, as measured by binding antibody and neutralization assays in the case-cohort immunogenicity set
8. To evaluate vaccine efficacy against asymptomatic SARS-CoV-2 infection	SARS-CoV-2 infection by PCR or periodic serology among participants seronegative for SARS-CoV-2 at baseline and without any prior reporting of symptoms that led to confirmation of a COVID-19 disease endpoint

9. To evaluate vaccine efficacy against SARS-CoV-2 infection and COVID-19 disease and the vaccine effect on viral load and secondary transmission among participants who received all planned immunizations at the designated immunization visits within specific visit windows	Endpoints evaluated among participants who were SARS-CoV-2 seronegative at enrollment<br>• SARS-CoV-2 infection diagnosed by PCR [Primary endpoint 1]<br>• Peak log10 viral load in nasal samples from participants who are diagnosed with SARS-CoV-2 infection [Primary endpoint 2]<br>• Number of secondary transmission events in the PCC and CACC [Secondary endpoint 1]<br>• SARS-CoV-2 infection coincident with signs and symptoms of COVID-19 listed under Secondary endpoint 3
10. To evaluate the association between measures of the magnitude of viral load over time and secondary transmission of SARS-CoV-2 infection, among individuals with incident SARS-CoV-2 infection in each treatment group, i.e. to conduct analyses aimed at evaluating viral load measures as surrogates of secondary transmission	• Peak log10 viral load [Primary endpoint 2] and other viral load summaries [Secondary endpoints 4]<br>• Number of secondary transmission events in the PCC cohort and CACC cohort [Secondary endpoint 1]
11. To evaluate vaccine effects on peak viral load and on secondary transmission of SARS-CoV-2 infection separately among individuals with asymptomatic vs. symptomatic SARS-CoV-2 infection	• Peak log10 viral load [Primary endpoint 2] and other viral load summaries [Secondary endpoints 4]<br>• Number of secondary transmission events in the PCC cohort and CACC cohort [Secondary endpoint 1]<br>• SARS-CoV-2 infection diagnosed using PCR, with and without signs and symptoms of COVID-19 listed under Secondary endpoint 3

Exploratory Objectives

1. To evaluate the associations between demographic and clinical factors and secondary transmission of SARS-CoV-2 infection and COVID-19 disease, among individuals with incident SARS-CoV-2 infection in the main study cohort and their contacts in the PCC and CACC
2. To assess if and how vaccine efficacy against SARS-CoV-2 infection, transmission and COVID-19 disease depends on baseline demographic, clinical, socio-economic indicators, and/or behavioral characteristics of study participants
3. To assess if and how vaccine efficacy against SARS-CoV-2 infection and COVID-19 disease depends on genotypic or neutralization phenotypic characteristics of SARS-CoV-2 (sieve analysis)

4. To further evaluate immunogenicity of the vaccine, additional immunogenicity assays may be performed such as antibody (Ab) Avidity and Fc Receptor function assays, based on the CoVPN Laboratory Center assay portfolio
5. To conduct analyses related to furthering the understanding of SARS-CoV-2, immunology, vaccines, and clinical trial conduct

4 Ethical considerations

It is critical that universally accepted ethical guidelines are followed at all sites involved in the conduct of clinical trials. The COVID-19 Prevention Network (CoVPN) have addressed ethical concerns in the following ways:

- CoVPN trials are designed and conducted to enhance the scientific knowledge base necessary to find a preventive vaccine, using methods that are scientifically rigorous and valid, and in accordance with International Council for Harmonisation of Technical Requirements for Pharmaceuticals for Human Use (ICH) and/or other Good Clinical Practice (GCP) guidelines.

- CoVPN scientists and operational staff incorporate the philosophies underlying major codes (44-46), declarations, and other guidance documents relevant to human subjects research into the design and conduct of SARS-CoV-2 vaccine clinical trials.

- CoVPN scientists and operational staff are committed to substantive community input—into the planning, conduct, and follow-up of its research—to help ensure that locally appropriate cultural and linguistic needs of study populations are met.

- The CoVPN provides training so that all participating sites similarly ensure fair participant selection, protect the privacy of research participants, and obtain meaningful informed consent. During the study, participants will have their wellbeing monitored, and to the fullest extent possible, their privacy protected. Participants may withdraw from the study at any time.

- Prior to implementation, CoVPN trials are rigorously reviewed by scientists who are not involved in the conduct of the trials under consideration.

- CoVPN trials are reviewed by local and national regulatory bodies and are conducted in compliance with all applicable national and local regulations.

- The CoVPN designs its research to minimize risk and maximize benefit to both study participants and their local communities. For example, CoVPN protocols provide enhancement of participants' knowledge of SARS-CoV-2 and SARS-CoV-2 prevention, as well as counseling, guidance, and assistance with any social impacts that may result from research participation. CoVPN protocols also include careful medical review of each research participant's health conditions and reactions to study products while in the study.

- CoVPN research aims to benefit local communities by directly addressing the health and SARS-CoV-2 prevention needs of those communities and by strengthening the capacity of the communities through training, support,

shared knowledge, and equipment. Researchers involved in CoVPN trials are able to conduct other critical research in their local research settings.

- The CoVPN values the role of in-country Institutional Review Boards (IRBs), Ethics Committees (ECs), and other Regulatory Entities (REs) as custodians responsible for ensuring the ethical conduct of research in each setting.

5 IRB/EC review considerations

US Food and Drug Administration (FDA) and other US federal regulations require IRBs/ECs/REs to ensure that certain requirements are satisfied on initial and continuing review of research (Title 45, Code of Federal Regulations (CFR), Part 46.111(a) 1-7; 21 CFR 56.111(a) 1-7). The following section highlights how this protocol addresses each of these research requirements. Each CoVPN Investigator welcomes IRB/EC/RE questions or concerns regarding these research requirements.

5.1 Minimized risks to participants

45 CFR 46.111 (a) 1 and 21 CFR 56.111 (a) 1: Risks to subjects are minimized.

This protocol minimizes risks to participants by (a) correctly and promptly informing participants about risks so that they can join in partnership with the researcher in recognizing and reporting harms; (b) respecting local/national blood draw limits; (c) performing direct observation of participants postvaccination and reporting information regarding side effects to the FDA/CDC Vaccine Adverse Event Reporting System (VAERS) per EUA guidance; (d) having staff properly trained in administering study procedures that may cause physical harm or psychological distress, such as blood draws, vaccinations, COVID-19 testing and counseling; and (e) providing safety monitoring.

5.2 Reasonable risk/benefit balance

45 CFR 46.111(a) 2 and 21 CFR 56.111(a) 2: Risks to subjects are reasonable in relation to anticipated benefits, if any, to subjects, and the importance of the knowledge that may reasonably be expected to result.

In all public health research, the risk-benefit ratio may be difficult to assess because the benefits to participants in prevention protocols are not as apparent as they would be in treatment protocols, where a study participant may be ill and may have exhausted all conventional treatment options. However, this protocol is designed to minimize the risks to participants while maximizing the potential value of the knowledge it is designed to generate.

5.3 Equitable participant selection

45 CFR 46.111 (a) 3 and 21 CFR 56.111 (a) 3: Subject selection is equitable

This protocol has specific inclusion and exclusion criteria for investigators to follow in admitting participants into the protocol. Participants are selected because of these criteria and not because of positions of vulnerability or privilege. Investigators are required to maintain screening and enrollment logs to document volunteers who screened into and out of the protocol and for what reasons.

5.4 Appropriate informed consent

45 CFR 46.111 (a) 4 and 5 and 21 CFR 56.111 (a) 4 and 5: Informed consent is sought from each prospective subject or the subject's legally authorized representative as required by 45 CFR 46.116 and 21 CFR Part 50; informed consent is appropriately documented as required by 45 CFR 46.117 and 21 CFR 50.27

The protocol specifies that informed consent must be obtained before any study procedures are initiated and assessed throughout the trial (see Section 9.1). Each site is provided training in informed consent by the CoVPN and is required to have a Standard Operating Procedure (SOP) on the informed consent process. The CoVPN requires a signed consent document for documentation, in addition to chart notes or a consent checklist.

5.5 Adequate safety monitoring

45 CFR 46.111 (a) 6 and 21 CFR 56.111 (a) 6: There is adequate provision for monitoring the data collected to ensure the safety of subjects.

Vaccination providers are required to report to VAERS the certain adverse events after COVID-19 vaccination, under Emergency Use Authorization (EUA) and encouraged to report adverse events to ModernaTX |(https://www.cdc.gov/vaccines/covid-19/vaccination-provider-support.html, see Section 11). Additionally, the use of v-safe will be recommended to vaccine recipients (https://www.cdc.gov/coronavirus/2019-ncov/vaccines/safety/vsafe.html, see Section 11), and an Independent Data Monitoring Committee (IDMC) will periodically review study data.

5.6 Protect privacy/confidentiality

45 CFR 46.111 (a) 7 and 21 CFR 56.111 (a) 7: There are adequate provisions to protect the privacy of subjects and maintain the confidentiality of data.

Privacy refers to an individual's right to be free from unauthorized or unreasonable intrusion into his/her private life and the right to control access to individually identifiable information about him/her. The term "privacy" concerns research participants or potential research participants as individuals whereas the term "confidentiality" is used to refer to the treatment of information about those individuals. This protocol respects the privacy of participants by informing them about who will have access to their personal information and study data (see Appendix A, Appendix B, Appendix C and Appendix D). The privacy of participants is protected by assigning unique identifiers in place of the participant's name on study data and specimens. In the United States, research participants in CoVPN protocols are protected by a Certificate of Confidentiality from the US NIH, which can prevent disclosure of study participation even when that information is requested by subpoena. Participants are told of the use and limits of the certificate in the study consent form. In addition, each staff member at each study site in this protocol signs an Agreement on Confidentiality and Use of Data and Specimens with the CoVPN. In some cases, a comparable confidentiality agreement process may be acceptable. Each study site participating in the protocol is required to have a standard operating procedure on how the staff members will protect the confidentiality of study participants.

6 Statistical considerations

With an overall goal of accruing 48,000 person-months divided evenly between vaccinated and unvaccinated follow-up time, we anticipate enrolling up to 18,000 participants. Up to 12,000 participants will be randomized 1:1 to Immediate Vaccination Group (at Months 0 and 1) or Standard of Care Group, with vaccination given at Months 4 and 5 if not received off-study previously. Up to an additional 6,000 participants will be enrolled into the Vaccine Declined Group and will also be offered vaccine at Months 4 and 5. Operational monitoring of SARS-CoV-2 incidence, retention, outside COVID-19 vaccination, adherence to study procedures, and enrollment and testing of contacts in the PCC and CACC – *pooled over main study participant groups*-- will guide the choice of total number randomized and total number in the Vaccine Declined Group. Guidelines will be specified in the study SAP.

Primary analyses of vaccine efficacy against infection (VE_S) and of the vaccine effect on viral load (ΔVL) will be conducted among all main study participants SARS-CoV-2 seronegative at enrollment. These analyses will be supported by analyses in the “Day 29 Negative Set”--the set of participants without PCR-confirmed infection on or prior to Day 29—that only count SARS-CoV-2 infection events diagnosed post-Day 29; see Table 6-1 for a full definition of the various analysis sets for the study. Secondary analyses will be conducted among all participants who receive both immunizations, ie, the Per-Protocol Set. Immunogenicity assessment will be done in the Case-Cohort Immunogenicity Set—a subsample of the participants randomized to immediate vaccination and sampled on the basis of SARS-CoV-2 infection status and baseline characteristics that will be fully described in the study SAP.

The FAS differs from a full Intention-to-Treat set by excluding randomized volunteers who do not enroll. The exclusion of these individuals is justified because the effects of the vaccine are unlikely to influence such exclusion. Because of the brief length of time between randomization and enrollment (ideally these will be on the same day), we expect almost all randomized volunteers to be in the FAS.

All primary and secondary analyses will be done according to enrollment group, ie, Immediate Vaccination Group, Standard of Care Group, or Vaccine Declined Group.

Main study participants will be tested using real-time PCR testing of swabs, and these data will be used to identify SARS-CoV-2 infection events for primary analysis. Some main study participants may also receive SARS-CoV-2 testing through university testing systems, or through outside providers, and will be asked to report any positive test results to the study team. Any positive SARS-CoV-2 test results for main study participants – based on PCR testing of study swabs or based on other testing systems indicative of acute infection– will trigger

swabbing of contacts in the PCC and enrollment and swabbing of contacts in the CACC. Secondary analyses of vaccine efficacy will include SARS-CoV-2 infection events diagnosed through any testing system – either by PCR testing of study swabs or through outside testing.

The two contact cohorts – the PCC and the CACC – will both be used for analyses of the vaccine effect on secondary transmission, as described below.

The study is "time-driven", ie, the primary analysis will occur when all participants have completed the 4 months follow-up. The number of primary endpoint infection events that accrue in the study is random and will depend on the level of SARS-CoV-2 incidence in each arm.

Table 6-1 Study Analysis Sets and Definitions

Analysis Set	Definition	Analyses
Full Analysis Set (FAS)	Main Study participants who enroll in the study	Primary efficacy analyses*
Day 29 Negative (D29-) Set	Participants in the FAS without PCR-confirmed infection on or before Day 29¥	Primary efficacy analyses*
Per-Protocol (PP) Set	Participants in the D29- set who received all planned immunizations at the designated immunization visits within specific visit windows	Secondary efficacy analyses*
Day 29 Negative Transmission Set (D29-TS)	Participants in the D29- set and their contacts in the Prospective and Case Ascertained Contact Cohorts	Primary analyses of secondary transmission*
Case-Cohort Immunogenicity Set (ccIS)	Subset of the FAS that is randomly sampled for measurement of immunological markers and fully described in study SAP	Immunology marker analyses

*These analysis sets include participants without regard to baseline SARS-CoV-2 status. However, primary
efficacy analyses will condition on baseline SARS-CoV-2 seronegative status.
¥ Note that Day 29 is the second vaccination visit (visit 3) for participants on the Immediate Vaccination Group. The SAP will specify the definition of Day 29 for participants in the Standard of Care Group and in the Vaccine Declined Group.

Table 6-2 Assumptions underlying study power/sample size calculations, unless otherwise specified. Parameters and their assumed values pertain to participants in the main study cohort.

Parameter	Assumption
Enrollment	Uniform enrollment over 4 weeks
Baseline seroprevalence in main study and PCC and CACC	10% of enrolled participants are baseline SARS-CoV-2 seropositive. This parameter is monitored and controlled operationally (see Section 6.3)
Per-protocol rate	98% of enrolled participants are in the per-protocol set
Loss to follow-up rate in the main study, the PCC and the CACC	5% of participants are lost to follow-up over 16 weeks at a constant rate
SARS-CoV-2 incidence absent vaccination	Exponential (constant rate) over 16 weeks; primary assumption is 4% over 16 weeks

Secondary transmission rate among susceptible contacts absent vaccination	15% transmission rate based on (47-49)
Vaccine efficacy (VE) against SARS-CoV-2 infection and transmission	Constant after 14 days post-first vaccination (calculations do not depend on earlier VE); level is varied
Contact enrollment	For the PCC: 60% of main study participants enroll contact(s) and the average number of contacts enrolled is 2<br>For the CACC: 75% of main study participants enroll contact(s) and the average number of contacts enrolled is 3

6.1 Accrual and sample size calculations

Sample size calculations were performed assuming that 48,000 person-months of follow-up will accrue, evenly divided between vaccinated and unvaccinated follow-up time and subject to a small 5% loss-to-follow-up rate. This person-time may come entirely from 12,000 participants randomized to Immediate Vaccination Group versus Standard of Care Group and followed for 4 months (16 weeks), or, if there is insufficient unvaccinated follow-up among those randomized to Standard of Care, it will be supplemented with unvaccinated follow-up for participants in the Vaccine Declined Group. The power calculations apply, regardless of where the unvaccinated follow-up comes from. For this reason, we refer to the two groups being compared as "vaccinated" and "unvaccinated", where the former group consists of participants randomized to immediate vaccination and the latter group may include both participants randomized to Standard of Care Group and participants enrolled in the Vaccine Declined Group (see Figure 2-7).

Power calculations also focused conservatively on numbers of infections that accrue post-Day 29, although as mentioned above, primary analyses will be conducted both in the FAS, counting all follow-up and infection events post-enrollment; and in the Day 29 negative set, counting only follow-up and infection events post-Day 29.

With 48,000 person-months of follow-up, evenly divided between vaccinated and unvaccinated follow-up, there is a high probability that a sufficient number of post-Day 29 infection events, ie, SARS-CoV-2 infections among baseline seronegative participants in the D29- set and diagnosed after Day 29, will accrue to adequately power analyses addressing both primary objectives as well as key secondary objectives. Specifically, with 24,000 person-months of vaccinated follow-up and 24,000 person-months of unvaccinated follow-up, under a 4% SARS-CoV-2 incidence absent vaccination over 16 weeks (a conservative assumption for US college campuses, see Section 2.5, and supported by unpublished modeling within the CoVPN), assuming VE_S = 50% and other assumptions laid out in Table 6-2, a total of 225 post-Day 29 infection events will occur across vaccinated and unvaccinated groups with probability 0.5 and 214 events will occur with probability 0.8.

Table 6-3 shows the expected number of post-Day 29 infection events in the main study cohort overall and in the vaccinated group, as the assumed incidence absent vaccination varies. As shown, the design ensures a sufficient number of post-Day 29 infection events to address a variety of objectives that condition on post-Day 29 infection. For example, with 4% SARS-CoV-2 incidence absent vaccination, 75 post-Day 29 infection events are expected in the vaccinated group for immune correlates analyses.

Table 6-3 Expected number of post-Day 29 infection events, available for secondary analyses that condition on event status. Calculations assume 24,000 person-months of vaccinated follow-up and 24,000 person-months of unvaccinated follow-up and other assumptions in **Table 6-2,** VE_S = 50%, and varying 16-week incidence of SARS-CoV-2 infection absent vaccination.

**16-Week SARS-CoV-2 Background (unvaccinated) Incidence**	**Post-Day 29 Infection Events**	
	Vaccinated and Unvaccinated Groups Combined	Vaccinated Group
1.5%	86	28
2%	143	47
3%	170	56
3.5%	198	66
4%	225	75

The study provides high power to evaluate the vaccine effect on acquisition (VE_S). Figure 6-1 shows the power of the study to detect a given level of VE_S, rejecting H_0: $VE_S \leq 30\%$, based on a 1-sided 0.025-level log-rank test and as a function of the SARS-CoV-2 incidence absent vaccination. With 4% 16-week incidence, the study has 90% power to detect any $VE_S > 57\%$; with 3% incidence, $VE_S > 60\%$ can be detected with 90% power.

The proposed study also provides adequate precision with which to estimate VE_S. Table 6-4 shows that, with 225 post-Day 29 infection events, the approximate 95% confidence interval (CI) about an estimated 50% VE_S is (36.9%, 60.4%). More precise inference is obtained under higher VE_S and higher post-Day 29 infection totals.

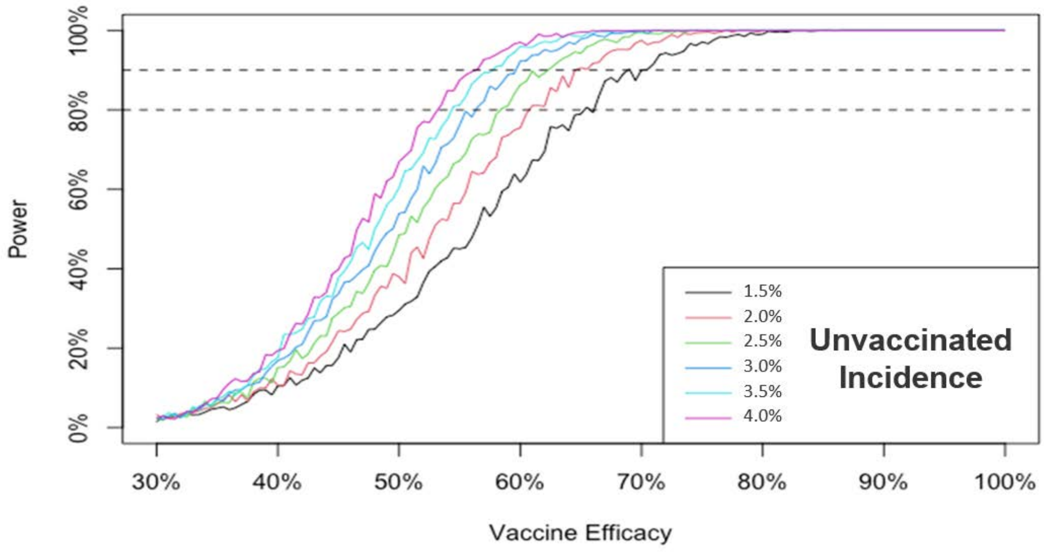

Figure 6-1 Power to detect varying vaccine efficacy (VE) against SARS-CoV-2 infection as a function of incidence absent vaccination over 16 weeks. Simulation-based power calculations assume 24,000 person-months of vaccinated and 24,000 person-months of unvaccinated follow-upand other assumptions listed in **Table 6-2**. Power based on 1-sided 0.025-level log-rank test and 1,000 simulations.

Table 6-4 Approximate 95% confidence interval around a given estimated VEs against SARS-CoV-2 infection, comparing vaccinated and unvaccinated groups as a function of the number of post-Day 29 infection events in the vaccinated and unvaccinated groups. Calculations assume 24,000 person-months of follow-up in each group and other assumptions in **Table 6-2**.

Number of post-Day 29 infection events	Approximate 95% CI for estimated VE = 50%	Approximate 95% CI for estimated VE = 70%
200	(36.0%, 60.9%)	(60.7%, 77.2%)
225	(36.9%, 60.4%)	(61.1%, 76.9%)
250	(37.7%, 59.9%)	(61.6%, 76.6%)

6.1.1 Power and precision for evaluating the vaccine effect on VL

Emerging data on acute SARS-CoV-2 infection suggest that with an expected 225 post-Day 29 infection events, the vaccine effect on mean peak log10 viral load among post-Day 29 infection events can be estimated with adequate precision. Specifically, assuming that peak log10 viral load is normally distributed with a 1.8 standard deviation in both treatment groups, and assuming 50% VEs, a 1-log reduction in mean peak viral load can be detected with 96.9% power given 225 post-Day 29 infection events; power remains high (93.5%) with 70% VEs. Table 6-5 shows power to detect a 1-log reduction in peak log viral load as a function of the assumed standard deviation in log peak viral load which is allowed to vary between 1.0 and 2.5. The standard deviation in peak log viral load in a cohort of

68 individuals associated with the National Basketball Association who were diagnosed with acute SARS-CoV-2 infection was 1.4 to 1.8, depending on the set of samples included in the analysis (50). Emerging data from the HCQ-PEP study of ~100 acutely infected household members of recently diagnosed COVID-19 cases suggests a standard deviation in peak log viral load in the range of 2.0-2.2, depending on the set of samples included [data not published]. Table 6-6 shows the approximate 95% CI about an estimated 1-log reduction in mean peak log viral load, demonstrating the precision that is obtained with 225 post-Day 29 infection events. Modest precision is observed with a 1-1.8 standard deviation in the endpoint, with lower precision under a higher standard deviation; precision is also lower under increasing VE_S.

Table 6-5 Power to detect a 1-log reduction in mean peak log viral load comparing post-Day 29 infection events between vaccinated and unvaccinated groups, as a function of the assumed standard deviation in peak log viral load in both groups and the assumed level of VE against SARS-CoV-2 infection. Simulation-based power calculations assume 225 post-Day 29 infection events across vaccinated and unvaccinated groups. Power based on 1-sided 0.025-level t-test and 1,000 simulations.

Std. dev. in peak log viral load	VE_S = 50%	VE_S = 70%
**1**	100%	100%
**1.5**	99.6%	97.8%
**1.8**	96.9%	93.5%
**2**	95.6%	86.5%
**2.5**	79.9%	68.7%

Table 6-6 Approximate 95% confidence interval around an estimated 1-log reduction in mean peak log viral load comparing post-Day 29 infection events between vaccinated and unvaccinated groups, as a function of the assumed standard deviation in peak log viral load in both groups and the assumed level of vaccine efficacy against SARS-CoV-2 infection. Calculations assume 225 post-Day 29 infection events across vaccinated and unvaccinated groups.

Std. dev. in peak log viral load	VE_S = 50%	VE_S = 70%
**1**	(0.72, 1.28)	(0.68, 1.31)
**1.5**	(0.57, 1.41)	(0.51, 1.46)
**1.8**	(0.50, 1.50)	(0.44, 1.57)
**2**	(0.46, 1.57)	(0.36, 1.61)
**2.5**	(0.29, 1.65)	(0.20, 1.74)

6.1.2 Power for evaluating the vaccine effect on secondary transmission

The study has adequate power to evaluate vaccine efficacy against secondary transmission, based on analyses that are conducted among post-Day 29 infection events in the Main study cohort and their contacts in the PCC and CACC, ie, based on conditional analyses, as long as VE against SARS-CoV-2 infection is low. As demonstrated in Figure 6-2, if there is high vaccine efficacy against

SARS-CoV-2 infection, power is low to evaluate secondary transmission using a conditional analysis. In this case, an unconditional analysis of VE against secondary transmission is planned and is expected to be more powerful, and also more appropriate given that it reflects that infections blocked have no transmission potential.

We performed simulation-based calculations to explore the power to evaluate VE against secondary transmission based on a conditional analysis that compares secondary transmission events among contacts of vaccinated and unvaccinated main study participants. In addition to the assumptions in Table 6-2, we assumed: equal infection surveillance in the PCC and CACC; that PCC contacts acquire infection from outside sources (other than the index case) at the same rate as the Standard of Care Group; that only baseline seronegative contacts and those in the PCC not infected by outside sources are at risk of secondary transmission; that the number of secondary transmission events among at-risk contacts follows a Poisson distribution in each arm, with VE against secondary transmission reducing the event rate proportionally given vaccination. A one-sided 0.025-level likelihood ratio test based on a Poisson regression model was used to evaluate VE against secondary transmission. As shown in Figure 6-2, the study has 90% power to detect a 59% reduction in secondary transmission if VE_S = 50%; a higher 69% reduction in secondary transmission is detectable if VE_S = 70%. Table 6-7 helps explain the power results: it shows the amount of statistical information on which the analysis is based, ie, the median number of post-Day 29 infection events and associated secondary transmission events in the vaccinated and unvaccinated groups. With 50% VE_S and 50% VE against secondary transmission, we expect 15 secondary transmission events in the vaccinated group and 59 in the unvaccinated group; the limited number of events is due to the "transmission cascade" whereby under our assumptions only some SARS-CoV-2 infected participants enroll contacts into the PCC and CACC, only some contacts are susceptible and retained, and the transmission rate is low (15%).

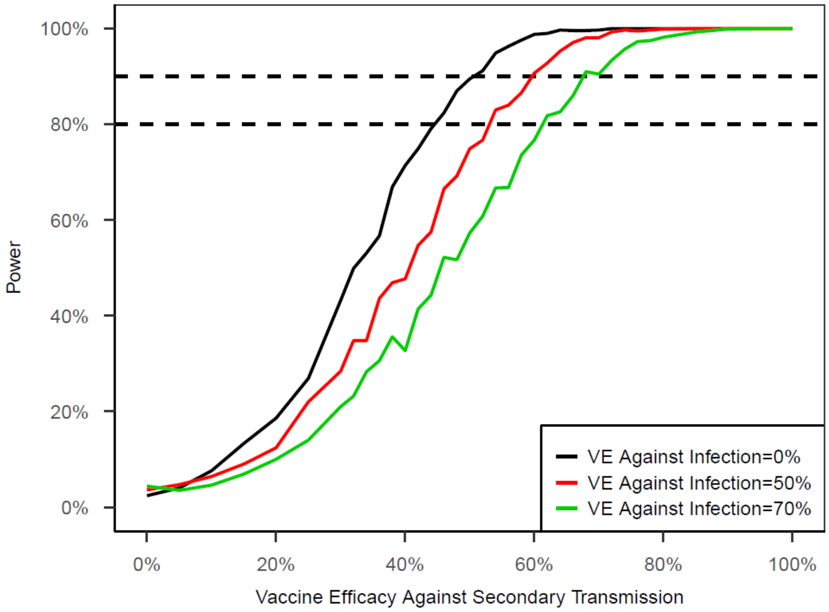

Figure 6-2 Power to detect varying vaccine efficacy against secondary transmission as a function of vaccine efficacy against SARS-CoV-2 infection. Simulation-based power calculations assume 24,000 person-months of vaccinated follow-up and 24,000 person-months of unvaccinated follow-up and other assumptions listed in **Table 6-2**. Power based on 1-sided 0.025-level likelihood ratio test under Poisson regression model and 1,000 simulations.

Table 6-7 Number of post-Day 29 infection events and associated secondary transmission events in the vaccinated and unvaccinated groups, under varying VE against SARS-CoV-2 infection and varying VE against secondary transmission, under other assumptions laid out in Table 6-2, and based on 1,000 simulations.

	**VEs = 50%**		**VEs = 70%**	
VE against secondary transmission	Median no. post-Day 29 infection events, Vaccinated vs. Unvaccinated Groups (IQR)	Median no. secondary transmissions, Vaccinated vs. Unvaccinated Groups (IQR)	Median no. post-Day 29 infection events, Vaccinated vs. Unvaccinated Groups (IQR)	Median no. secondary transmissions, Vaccinated vs. Unvaccinated Groups (IQR)
VE = 0%	76 (70-82) vs. 150 (142-158)	29 (25-34) vs. 59 (53-66)	45 (41-50) vs. 149 (141-158)	18 (14-21) vs. 59 (53-65)
VE = 40%	76 (70-81) vs. 150 (142-158)	18 (15-21) vs. 59 (53-64)	45 (41-50) vs. 149 (142-158)	11 (8-13) vs. 59 (53-65)
VE = 50%	76 (70-82) vs. 150 (143-159)	15 (12-17) vs. 59 (53-66)	45 (41-50) vs. 150 (142-158)	9 (7-11) vs. 59 (53-65)
VE = 60%	76 (70-82) vs. 150 (142-158)	12 (9-14) vs. 58 (52-65)	45 (41-50) vs. 151 (143-159)	7 (5-9) vs. 59 (53-65)
VE = 70%	75 (70-81) vs. 150 (142-158)	9 (7-11) vs. 58 (52-65)	46 (41-50) vs. 150 (142-159)	5 (4-7) vs. 59 (53-65)

6.2 Role of the Independent Data Monitoring Committee (IDMC)

The study IDMC will provide study oversight and ensure that the study is on-target to meeting its objectives in a timely fashion. The IDMC will receive summaries of study progress throughout the study. Given the short duration of the study and the considerable prior data establishing safety and efficacy of the

vaccine, and the status of the EUA vaccine, there will be no pre-specified interim monitoring boundaries for VE or non-efficacy. The IDMC will make recommendations to protocol chairs and lead advisors. The set of metrics, operational targets for them, and potential remedial actions will be laid out in full in the IDMC Charter.

6.3 Randomization

A participant's randomization assignment will be computer generated and provided to the CRS study staff through a Web-based randomization system.

Randomization will be stratified. The randomization will be done in blocks to ensure balance among contemporaneously evaluated Immediate Vaccination and Standard of Care arms within levels defined by study site and type of residence.

6.4 Statistical analyses

This section describes the final study analyses which will occur after the last main study participant has completed the Month 4 visit. All data from enrolled participants will be analyzed according to the initial group assignment regardless of how and when in-study and outside-study vaccinations are received. Follow-up and endpoints that accrue post-outside-study-vaccination will be censored for primary and secondary analyses. In the rare instance that a participant receives study vaccination at the wrong time, the Statistical Analysis Plan (SAP) will address how to analyze the data. Analyses are modified intent-to-treat in that individuals who are randomized but not enrolled do not contribute data and hence are excluded. Because of the brief length of time between randomization and enrollment, few such individuals are expected.

The SAP will specify how data from the Vaccine Declined group will contribute to assessing primary and secondary objectives. Wherever possible, analyses will limit to data collected on randomized participants, and will entail comparing outcomes between those randomized to Immediate Vaccination or Standard of Care. However, if operational data on enrollment, retention, adherence to study procedures suggest that there will be insufficient unvaccinated follow-up for the Standard of Care Group to permit a specific analysis, data from the Standard of Care Group will be combined with data from the Vaccine Declined Group, with methods fully pre-specified in the SAP.

Analyses for primary endpoints will be performed using SAS and R. All other descriptive and inferential statistical analyses will be performed using SAS, StatXact, or R statistical software.

In general, no formal multiple comparison adjustments will be employed for multiple secondary endpoints. However, multiplicity adjustments will be made for

certain immunogenicity assays, as discussed below, when the assay endpoint is viewed as a collection of hypotheses (eg, testing multiple peptide pools to determine a positive response).

6.4.1 Analysis variables

The analysis variables defined for all participants in the main study cohort consist of baseline participant characteristics, longitudinal measures of risk of infection, SARS-CoV-2 infection and seroconversion and COVID-19 disease status, and immune response biomarkers. For individuals diagnosed with SARS-CoV-2 infection, post-infection variables will include various summaries of VL, the full genome sequence of the infecting virus, and the infection status of and relationship to each of their contacts enrolled in the PCC and CACC. For contacts enrolled in the PCC and CACC, variables consist of baseline participant characteristics, SARS-CoV-2 infection and seroconversion status, risk factors around time of exposure to main study participant (if infected) and relationship to the referring main study participant.

6.4.2 Baseline comparability and comparability over study follow-up

Groups of main study participants will be compared with respect to baseline participant characteristics using descriptive statistics.

Given that the study is open-label, and the associated potential for differences between randomized arms with respect to retention, compliance with study procedures, ascertainment of endpoints, and variables associated with exposure to SARS-CoV-2, these measures will be compared between randomized arms over time using methods fully described in the SAP. Given that participants self-select into the Vaccine Declined Group, similar methods will be used to evaluate potential differences between this group and those randomized to Immediate vaccination or Standard of Care. Imbalances between groups will influence the analysis choices as described below.

6.4.3 Vaccine efficacy (VE) analyses against SARS-CoV-2 infection

The time origin for all analyses of SARS-CoV-2 infection is enrollment. We define the time of diagnosis of the SARS-CoV-2 infection endpoint as the collection time of the first daily swab with a positive PCR test. The time of diagnosis of lab-confirmed clinical COVID-19 disease is the first time of reported qualifying COVID-19 symptoms that occurs simultaneously or after the diagnosis of the SARS-CoV-2 infection endpoint. The time of diagnosis of asymptomatic SARS-CoV-2 infection is the collection time of the first sample with a positive PCR test result or seroconversion without any prior or subsequent reporting of symptoms that led to confirmation of a COVID-19 disease endpoint. The time of diagnosis of SARS-CoV-2 seroconversion is the collection time of the first sample seropositive for SARS-CoV-2, for participants SARS-CoV-2 seronegative at baseline; the SAP will specify criteria for defining seroconversion (re-infection)

for individuals SARS-CoV-2 seropositive at baseline. The failure times of participants never observed to be diagnosed with a given clinical endpoint will be right-censored at the date of last contact at which endpoint status was assessed or study termination. The failure times of participants reporting receipt of COVID-19 vaccination outside the study will be right-censored at the date of first outside vaccination.

6.4.3.1 Primary VE Analysis

For the primary analysis, Cox proportional hazards regression will be used to estimate vaccine efficacy against SARS-CoV-2 infection among baseline SARS-CoV-2 seronegative participants, as measured by one minus the hazard ratio (HR) in the Immediate Vaccination vs. Standard of Care arms. This analysis that restricts attention to randomized participants will be supported by a co-primary analysis that combines data from the Standard of Care arm with data from the Vaccine Declined Group. An additional supportive analysis will restrict to the D29- set, counting only infection events diagnosed post-Day 29. These analyses supporting the primary objectives of the study will be detailed in the SAP.To improve precision when comparing randomized groups, and to avoid bias when including non-randomized participants, primary analyses will adjust for an SAP-specified set of “risk” variables, or a composite risk score based on them, that are potentially associated with SARS-CoV-2 exposure including demographics and behaviors captured using the risk assessment tool. Inference will be based on a 2-sided score-based CI and 2-sided score-test for H0: VE = 0% with a type 1 error rate of 0.05, although primary interest is in estimation as opposed to hypothesis testing and the study is designed to ensure that the lower limit for the CI will lie above 30% with high probability under VE > 60%.The baseline hazard function in the Cox model will be stratified by study site and randomization stratum, which improves precision of the VE estimate and accounts for the fact that SARS-CoV-2 infection incidence may vary widely across study sites. Description of the risk variables under consideration and of the derivation of a risk score based on them will be described in the study SAP.

The potential impacts of the open-label and observational aspects of the study design will be factored into the choice of efficacy analysis. If there is evidence of differential retention of study participants across groups, or differential adherence to study procedures affecting ascertainment of endpoints such as collection of daily swabs, the analyses will adjust for the differential censoring distribution across groups- as well as potentially informative censoring- as described in the study SAP. At a minimum, these analyses will be performed as sensitivity analyses, complementing the primary analyses described above.

Variables captured using the risk assessment tool and potentially associated with exposure will be compared across groups over study follow-up. If there is evidence that their distributions differ between groups over time, sensitivity analysis will additionally adjust for time-dependent exposure-related variables.

The proportional hazards assumption will be evaluated using methods of Grambsch and Therneau (51).

6.4.3.2 Secondary VE Analyses

The primary analysis of VE against SARS-CoV-2 infection will be repeated in the FAS and D29- sets but including baseline seropositive participants [Secondary Objective 5]; and in the PP set [Secondary Objective 9]. The same methods also apply to evaluating VE against seroconversion [Secondary Objective 2] and VE against COVID-19 disease [Secondary Objective 3]. Additional secondary VE analyses will be conducted where the time of SARS-CoV-2 infection diagnosis is defined as the earliest of the first positive SARS-CoV-2 test result based on either PCR testing of nasal swabs or external SARS-CoV-2 testing.

While the primary analysis of VE against SARS-CoV-2 infection will censor the follow-up time of participants who receive outside COVID-19 vaccination at the time of first outside vaccination, a secondary analysis will be performed that includes infection events and person-time that accrue following outside vaccination with the Moderna COVID-19 Vaccine. Participants receiving outside vaccination other than Moderna COVID-19 Vaccine will continue to be censored at the time of outside vaccination. A time-varying Cox model will be used for inference, per methods in the study SAP.

The asymptomatic SARS-CoV-2 infection endpoint [Secondary Endpoint 8] is defined by the PCR tests of daily swabs and serologic diagnostic testing schedule at month 0, 2, and 4. The occurrence of a COVID-19 disease event is a competing risk because the occurrence of COVID-19 disease precludes the possibility of occurrence of a subsequent asymptomatic SARS-CoV-2 infection event. Therefore, VE against asymptomatic SARS-CoV-2 infection will be assessed using methods accounting for COVID-19 disease as a competing risk. The SAP will provide details.

Vaccine efficacy against the infection and disease endpoints as measured by the reduction in the cumulative incidence of the endpoint by Month 4, will also be evaluated using the Nelson-Aalen estimator of cumulative incidence. This analysis does not rely on the proportional hazards assumption and has a simple interpretation that lends itself to understanding of population effects.

6.4.4 VL analyses

Primary analyses of the vaccine effect on VL will focus on the primary VL endpoint, peak log10 viral load, and will condition on SARS-CoV-2 infection status. Peak log10 viral load will be estimated by the observed maximum log10 viral load measure; secondary VL analyses will employ parametric models to estimate VL at peak. The mean observed log10 viral load will be compared between the Immediate Vaccination and Standard of Care arms. This analysis that restricts attention to randomized participants will be supported by a co-primary

analysis that combines data from the Standard of Care arm with data from the Vaccine Declined Group. Both analyses will use methods that adjust for baseline covariates and covariates at the time of SARS-CoV-2 infection diagnosis, per the study SAP. The covariate adjustment is used to control for potential post-randomization selection bias and lack of comparability between randomized participants and those in the observed cohort, whereby SARS-CoV-2 infected participants in the vaccinated and unvaccinated groups are not comparable in ways that are associated with the VL endpoint. Importantly, this bias occurs when there are factors that modify VE against SARS-CoV-2 infection *and* that are associated with the VL endpoint. An additional supportive analysis will restrict to SARS-CoV-2 infections in the D29- set. Full details around the analysis plan will be specified in the study SAP.

Secondary analyses of vaccine effects on VL will consider a set of other summary measures of the VL curve, each of which have merits for further characterizing the vaccine effect. These include a model-based estimate of peak log10 viral load, duration of detection of virus [estimated empirically or alternatively based on a model fit to the viral load curve], area under the log viral load curve [AUC] calculated using the trapezoidal rule, and area under the portion of the log viral load curve over 10^5 viral copies/mL. Some mathematical modeling studies provide evidence of transmission potential for VL above this threshold (21), but there is considerable uncertainty and therefore other thresholds will be considered. Analysis methods will be the same as for the primary VL endpoint above.

In addition, the above VL endpoints will be evaluated using unconditional analyses, ie, not conditioning on SARS-CoV-2 infection status, among all enrolled main study participants. Participants not diagnosed with SARS-CoV-2 infection will have an endpoint value defined for analysis, typically a value of zero. The ‘transmission potential’ endpoint [Secondary endpoint 4] is one such endpoint that is defined unconditionally. The merit of unconditional analyses is that they are not subject to potential post-randomization selection bias. A disadvantage of the unconditional analyses is that they may have lower statistical power.

An additional VL endpoint that will be studied is a censored event-time variable, time to VL above 10^5 copies/mL. This endpoint is defined for all enrolled main study participants and will be analyzed using the same methods used to evaluate VE against SARS-CoV-2 infection, but only counting infections once VL is above 10^5 copies/mL. Additional methods of analysis that allow for the uncertainty in the level of virus that is associated with transmission will be considered.

Sensitivity analyses for VL will include data from all SARS-CoV-2 infected participants diagnosed based on PCR testing of study swabs or based on outside testing.

The study SAP will detail the secondary analysis approaches for the VL endpoints.

6.4.5 Secondary transmission analyses

6.4.5.1 Identifying ‘adjudicated transmission events’

An expert adjudication committee will examine and make a primary determination to identify ‘adjudicated transmission events’ from a main study participant diagnosed with infection (‘index case’) to a contact in the PCC or CACC diagnosed with SARS-CoV-2 infection. Note that the close contact could also be a main study participant in theory, although this is unlikely given the relatively small proportion of each community that will be recruited to the main study. The adjudication committee will operate under a charter that lays out a pre-specified set of criteria for defining the occurrence and direction of transmission and will be blinded to the enrollment group of all study participants and contacts. The endpoint definition will include criteria that need to be met regarding the temporal relationship (eg, evidence that person B’s infection occurred after person A’s infection, evidence of compatible incubation period), epidemiologic link (evidence of close contact during person A’s infectious period), and relatedness of their SARS-CoV-2 viruses.

The adjudication committee will be provided with all relevant data from the study to deduce transmission events from potential transmission pairs. A potential transmission pair is a post-baseline SARS-CoV-2- infected main study index case and their identified contact(s) in the PCC or CACC who have positive SARS-CoV-2 test results within a prespecified period (eg, two weeks) of the index case diagnosis. This will include the type, frequency, and magnitude (eg, duration and setting) of contact between the index case and contact based on risk assessment questionnaires given to the index case and the contact; the timing of diagnosis of infection by PCR for the index case and the contact (defined as the date of specimen collection); the VL trajectories, serology, and symptomatology for the index case and the contact; and summaries of the viral sequences for the index case and the contact including the number of nucleotide differences in the sequences and any clustering and results of phylogenetic analyses (see Section 6.4.5.3). Adjudication of all potential transmission events will occur within a short time frame at the end of the study to ensure decisions are being made using the same background research on transmission and to ensure that the adjudication can take into account all SARS-CoV-2 infection events captured in the main study cohort and both contact cohorts. Primary analyses of the vaccine effect on secondary transmission will rely on the determination of this committee and use the adjudicated transmission events and outcomes in statistical models. These same primary analyses will also only include main study participants diagnosed with infection through PCR testing of study swabs. However, sensitivity analyses will incorporate data from the full set of main study participants diagnosed with infection either through outside testing or through PCR testing of swabs collected through the study per methods described in the SAP.

6.4.5.2 Analyses of the vaccine effect on secondary transmission

Primary analyses of vaccine efficacy against secondary transmission will include adjudicated transmission events collected from both the PCC and CACC. Both conditional and unconditional analyses will be performed. Conditional analyses will compare the expected number of adjudicated transmission events between index cases in the Immediate Vaccination Group vs. Standard of Care Group. This analysis that restricts attention to randomized participants will be supported by a co-primary analysis that combines data from the Standard of Care Group with data from the Vaccine Declined Group. An additional supportive analysis will restrict to SARS-CoV-2 infection events in the D29- set. These primary analyses will use Poisson regression, where goodness of fit tests will be used to determine whether an overdispersed Poisson or other alternative distributions fit the data better, per the study SAP. Analyses using the observational cohort cannot assume balanced mixing with susceptible contacts between vaccinated and unvaccinated groups and will adjust for number and type of contacts. The SAP will specify the baseline participant characteristics and measurements available at the time of SARS-CoV-2 infection diagnosis that are potentially associated with secondary transmission to be included as covariates. The conditional analysis may have greater power than an unconditional analysis, eg, under low VE against SARS-CoV-2 infection. However, it is subject to the post-randomization selection bias, as discussed in relation to the viral load endpoint. In general, it will be biased if there are factors that modify VE against SARS-CoV-2 infection that are also associated with onward transmission; or if factors associated with onward transmission are modifiers of VE against infection.

A secondary analysis of this endpoint will measure vaccine efficacy against secondary transmission as the percent reduction in the expected number of adjudicated transmission events linked to a participant in the Immediate Vaccination Group relative to the Standard of Care Group. In contrast to the primary transmission analysis that conditions on the main study participant being infected, all main study participants will contribute to the analysis including those who do not become infected to reflect the fact that SARS-CoV-2-uninfected participants do not transmit to others and therefore have 0 transmission events. Let N = number of adjudicated transmission events for a given index case which is assumed to follow a zero-inflated Poisson distribution with the mean of the non-zero point mass component is $\mu e^{(V\gamma)}$, where V indicates randomization assignment (1 Immediate Vaccination; 0 Standard of Care). Further let β denote the log hazard ratio from a Cox proportional hazards model for infection time. Then the ratio of the expected number of adjudicated transmission events linked to a participant in the Immediate Vaccination Group relative to the Standard of Care Group can be expressed as

$$e^{\theta} = e^{\gamma} e^{\beta},$$

where θ is a measure of the vaccine's impact on total onward transmission (transmission from participants in main study) and e^{θ} is the relative reduction in the proportion of adjudicated transmission events in the Immediate Vaccination Group relative to the Standard of Care Group. This is analogous to the product estimator in the proportional means model (53, 54). Note that this formulation assumes the time between detection in the index case participant and infection of a contact is ignorable. Covariate-adjusted approaches will be detailed in the SAP. The analysis that restricts attention to randomized participants will be supported by an analysis that combines data from the Standard of Care Group with data from the Vaccine Declined Group.

The unconditional analysis retains high power even if there are few breakthrough infections in the Immediate Vaccination Group due to high VE against infection. It also properly reflects that a vaccine is successful at reducing secondary transmission if it either meaningfully reduces acquisition or reduces onward transmission from infected participants. Moreover, by avoiding conditioning on SARS-CoV-2 infection, it avoids the post-randomization selection bias issue.

6.4.5.3 Processing and phylogenetic analysis of SARS-CoV-2 viral sequence data

A central sequencing facility will obtain whole-genome sequences from all infected participants in the main study cohort and all infected participants in the PCC and CACC, based on the nasal swab with the highest viral load (see Section 10.3.2). Details about the sequencing platform and methodology for QC processes will be included in the study SAP. This will include the creation of a global alignment, which will be used for the isolation of genes and the sequence analyses of local regions.

A first step in the analysis of the sequence data will be to determine, for each pair of sequences among infected participants or contacts within a given study community, which are identical. Data from GenBank, Global Initiative on Sharing Avian Influenza Data (GISAID), and other public databases suggest that we can expect limited variability in genetic sequences, especially those from a single common ancestor and constrained within a single study community over a 4-month time period. Consensus-based whole genomes that differ by more than two nucleotides can reasonably be ruled out as being transmission events.

A second step will be to conduct distance-based analyses of the sequences, including phylogenetic analysis, to identify potential lineages of infecting viral strains, infection clusters, and potentially linked transmissions. Phylogenetic analyses will also include 'background' SARS-CoV-2 sequences with geographical or temporal relevance from publicly available databases, such as GenBank and GISAID. The background sequences will help root the tree and give the tree structure and may lend insight into the identification of clusters and into potential transmission events during the adjudication process.

Details about the phylogenetic and other sequence analyses, including the tree-building methods and evolutionary models to be used, as well as approaches to incorporating data from multiple reads, will be available in the study SAP.

6.4.6 Immunogenicity analyses

6.4.6.1 Vaccine immunogenicity

Immunogenicity will be characterized for the immediate vaccination group only, using data from the Case-Cohort Immunogenicity Set (ccIS). Data from quantitative immunogenicity assays will be summarized using positive response rates and geometric means with 95% CIs, for each timepoint for which an assessment is performed. Data from qualitative (ie, yielding a positive or negative result) assays will be summarized by tabulating the frequency of positive responses for each assay at each timepoint that an assessment is performed. Analyses focus on the Month 2 time point, the Day 1 time point, and the change in marker response between the two time points. The analyses will evaluate immunogenicity in all immediate vaccination arm participants and separately in subgroups defined by baseline SARS-CoV-2 serostatus. The SAP will describe the complete set of immunogenicity analyses.

6.4.6.2 Immune response marker correlates of risk

A separate Trial Marker SAP will describe the statistical methods and data analysis implementations for assessing immune response markers as correlates of the SARS-CoV-2 infection and peak log viral load study endpoints, as well as various types of correlates/surrogates of protection.

An unblinded statistical analysis by treatment assignment of a primary immunogenicity endpoint may be performed when all participants have completed the Month 2 visit and data are available for analysis from at least 80% of these participants. Similarly, an unblinded statistical analysis by treatment assignment of a secondary or exploratory immunogenicity endpoint may be performed when all participants have completed the corresponding immunogenicity visit and data are available for analysis from at least 80% of these participants. The CoVPN Laboratory Center will review the analysis report prior to distribution to the protocol chairs, NIAID, study product developer, and other key CoVPN members and investigators. Reports for distribution or presentation should use PubIDs and not PTIDs for individual responses. Distribution of reports will be limited to those with a need to know for the purpose of informing future trial-related decisions. The CoVPN leadership must approve any other requests for CoVPN immunogenicity analyses prior to the end of the scheduled follow-up visits.

7 Selection and withdrawal of participants

Participants will be generally healthy adults who comprehend the purpose of the study and have provided written informed consent. Volunteers will be recruited and screened; those determined to be eligible, based on the inclusion and exclusion criteria, will be enrolled in the study. Final eligibility determination will depend on information available at the time of enrollment, medical history questionnaire, physical examinations, and answers to self-administered and/or interview questions.

Investigators should always use good clinical judgment in considering a volunteer's overall fitness for trial participation. Some volunteers may not be appropriate for enrollment even if they meet all inclusion/exclusion criteria. Medical, psychiatric, occupational, or other conditions may make evaluation of safety and/or immunogenicity difficult, and some volunteers may be poor candidates for retention.

Determination of eligibility, taking into account all inclusion and exclusion criteria, must be made within 56 days prior to enrollment unless otherwise noted below.

7.1 Inclusion criteria for Main Cohort, Immediate Vaccination Group and Standard of Care Group

General and Demographic Criteria

1. **Age** of 18 through 29 years.

2. **Willingness to be followed** for the planned duration of the study.

3. **Agrees to allow study staff to access school SARS-CoV-2 testing** data and outcomes, if applicable.

4. Ability and willingness to provide **informed consent.**

5. **Assessment of understanding**: volunteer demonstrates understanding of this study; completes a questionnaire with demonstration of understanding of all questionnaire items answered incorrectly.

6. **Willing to be randomized to either immediate vaccination or standard of care group and comply with planned study procedures.**

7. **Agrees not to enroll in another study** of an investigational research agent until the end of the study.

8. **Access to device and internet** for completion of study procedures.

7.2 Exclusion criteria for Main Cohort, Immediate Vaccination Group and Standard of Care Group

General

1. **Acutely ill 72 hours prior to or at screening**. Volunteers meeting this criterion may be rescheduled within the relevant window periods. Participants with minor illnesses can be enrolled at the discretion of the investigator.

2. **Blood products,** systemic immunoglobulins, or monoclonal antibodies (including against SARS-CoV-2) received within 90 days before first vaccination.

3. **Investigational research agents** received within 30 days before first vaccination.

4. **Self-reported known history of SARS-CoV-2 infection.**

Vaccines and other Injections

5. **Prefers to receive COVID-19 vaccination immediately.** (These volunteers to be referred to community resources for vaccination).

6. **Prior administration of a coronavirus** (SARS-CoV-2, SARS-CoV, MERS-CoV) vaccine or current/planned simultaneous participation in another interventional study to prevent or treat COVID-19.

Immune System

7. **Immunosuppressive medications** received within 168 days before first vaccination (not exclusionary: [1] corticosteroid nasal spray; [2] inhaled corticosteroids; [3] topical corticosteroids for mild, uncomplicated dermatologic condition; or [4] a single course of oral/parenteral prednisone or equivalent at doses $\leq$ 60 mg/day and length of therapy < 11 days with completion at least 30 days prior to enrollment).

Clinically significant medical conditions

8. **Clinically significant medical condition**, physical examination findings, or past medical history with clinically significant implications for current health. A clinically significant condition or process includes but is not limited to:

   - A process that would affect the immune response (well-controlled human immunodeficiency virus infection is allowed), or

   - Any condition specifically listed among the exclusion criteria below.

9. **Any medical, psychiatric, occupational, or other condition** that, in the judgment of the investigator, would interfere with, or serve as a contraindication

to, protocol adherence, assessment of safety or reactogenicity, or a volunteer's ability to give informed consent.

10. **Bleeding disorder** (eg, factor deficiency, coagulopathy, or platelet disorder requiring special precautions).

11. **Asplenia**: any condition resulting in the absence of a functional spleen.

12. **History of angioedema or anaphylaxis**, including to vaccines or vaccine components (not exclusionary: angioedema or anaphylaxis with known trigger and no episodes within five years.).

13. **History of generalized urticaria** within past five years.

7.3 Inclusion criteria for Main cohort, Vaccine Declined Group

General and Demographic Criteria

1. **Age** of 18 through 29 years.

2. Ability and willingness to provide **informed consent.**

3. **Prefers not to receive COVID-19 vaccine**.

4. **Willingness to be followed** for the planned duration of the study.

5. **Assessment of understanding**: volunteer demonstrates understanding of this study; completes a questionnaire with demonstration of understanding of all questionnaire items answered incorrectly.

6. **Access to device and internet** for completion of study procedures.

7.4 Exclusion criteria for Main cohort, Vaccine Declined Group

1. **Prior administration of a coronavirus** (SARS-CoV-2, SARS-CoV, MERS-CoV) **vaccine** or current/planned simultaneous participation in another interventional study to prevent or treat COVID-19 (participation in studies of other investigational research agents allowed).

2. **Any medical, psychiatric, occupational, or other condition** that, in the judgment of the investigator, would interfere with, or serve as a contraindication to, protocol adherence, or a volunteer's ability to give informed consent.

7.5 Inclusion criteria for Prospective Close Contact (PCC) cohort

1. **Age** of 18 years or older, at the time of signing the informed consent.
2. Willing and able to provide **informed consent**.
3. Expected to be in **frequent close physical proximity with Main Cohort participant** during the study.
4. **Willing to share results of SARS-CoV-2 testing**.
5. **Access to device and internet** for completion of study procedures.

7.6 Inclusion criteria for Case-ascertained Close Contact (CACC) cohort

1. **Age** of 18 years or older, at the time of signing the informed consent.
2. Willing and able to provide **informed consent**.
3. **Access to device and internet** for completion of study procedures.
4. Willing to share **results of SARS-CoV-2 testing**.
5. Had **close contact with Main Cohort participant with known PCR-confirmed SARS-CoV-2 infection** (eg, index case). Close contacts will have had exposure to the index participant, generally within 72 hours of an index diagnosis, and may include individuals that meet any of the following guidelines:

   - Prolonged close physical proximity with Main Cohort participant within a residence/vehicle/enclosed space without maintaining social distance,
   - Medical staff, first responders, or other care persons who cared for the index case without appropriate personal protective equipment.

   Further information on the definition of close contact can be found in the CoVPN 3006 Study Specific Procedures (SSP).

8 Study product

The protocol schema is shown in Table 1-1.

Definitions of on-study and outside of study COVID-19 vaccination:

- On-study: occurred as directed by the protocol procedures (i.e. procedures occurring within the visit windows) with Moderna COVID-19 vaccine for the cohort to which the participant has been randomized or assigned

- Outside of study: any COVID-19 vaccination that was administered at a time outside of protocol-specified visit windows for the cohort to which the participant has been randomized or assigned

Moderna COVID-19 Vaccine provided via the US Government study-allocated supply can only be administered to CoVPN 3006 study participants in an on-study manner. Exceptions may be made for Main Cohort study participants who become infected with SARS-CoV-2 after enrollment. Such exceptions require approval of CoVPN 3006 Protocol leadership.

8.1 Vaccine regimen

Immediate Vaccination Group

Treatment 1 (T1): Moderna COVID-19 Vaccine in 100 mcg dose given as 0.5 ml IM into the deltoid muscle on Day 1 and Day 29.

Standard of Care Group and Vaccine Declined Group who accept vaccine offer at M4

Treatment 2 (T2): Moderna COVID-19 Vaccine in 100 mcg dose given as 0.5 ml IM into the deltoid muscle on Day 113 and Day 141.

8.2 Storage, handling, preparation, and administration

Vaccination providers will refer to EUA vaccine instructions accessed and updated at https://www.modernatx.com/covid19vaccine-eua/.

8.3 Acquisition of Moderna COVID-19 Vaccine

Emergency use authorized Moderna COVID-19 Vaccine will be provided by Moderna, Inc. through the US Government COVID-19 Vaccine Response and allocated for this trial. Participants may also access Moderna COVID-19 EUA vaccine via other sources for on-study vaccinations if the administration fee is

either paid for by the study or is not billed to any party. Examples of how vaccinations may be administered to study participants includethe following:

- At the CRS using study-allocated vaccine supply
- At a non-CRS location using study-allocated vaccine supply with administration fees funded by the study
- At a pharmacy administering EUA Moderna COVID-19 vaccine via Federal Retail Pharmacy Partnership (FRPP) supply with administration fees funded by the study

Please see the CoVPN 3006 SSP for additional examples and details.

9 Clinical procedures

Procedures are in place so that study visits may be conducted remotely, such as via phone, text message, email, or other electronic means, in lieu of, or in combination with, in-person visits at the clinical research site. Furthermore, some visit procedures may be conducted outside the CRS (see CoVPN 3006 SSP for additional details).

The schedules of clinical procedures are shown in Appendix E, Appendix F, Appendix G and Appendix H.

9.1 Informed consent

Informed consent is the process of working with participants so that they fully understand what will and may happen to them while participating in a research study. The CoVPN informed consent form documents that a participant (1) has been informed about the potential risks, benefits, and alternatives to participation, and (2) is willing to participate in a CoVPN study. Informed consent encompasses all written, verbal, and electronic study information CRS staff provide to the participant, before and during the trial. CRS staff will obtain informed consent of participants according to the CoVPN 3006 SSP.

If any new information is learned that might affect the participants' decisions to stay in the trial, this information will be shared with trial participants. If necessary, participants will be asked to provide revised informed consent forms.

9.1.1 Screening consent form

Without a general screening consent, screening for a specific study cannot take place until the site receives protocol registration from NIAID or its designee.

Some CRSs have approval from their IRB/EC and any applicable RE to use a general screening consent form that allows screening for an unspecified SARS-CoV-2 vaccine trial. In this way, CRS staff can continually screen potential participants and, when needed, proceed quickly to obtain protocol-specific enrollment consent. Sites conducting general screening or prescreening approved by their IRB/EC and any applicable RE may use the results from this screening to determine eligibility for this protocol, provided the tests are conducted within the time periods specified in the eligibility criteria.

9.1.2 Protocol-specific consent forms

The protocol-specific consent forms describe the study products to be used and all aspects of protocol participation, including screening and enrollment procedures. Sample protocol-specific consent forms for the Main cohort are located in Appendix A and Appendix B. A sample protocol-specific consent form for the

PCC cohort is located in Appendix C. A sample protocol-specific consent form for the CACC cohort is located in Appendix D.

The consent forms for the PCC and CACC cohorts may be managed centrally by the CoVPN. This will allow for a streamlined approach to consenting these individuals, given the referral mechanism to participate in the study, in addition to the remote nature of their study participation. In this instance, the centralized consents and consenting process will be approved by the IRB/EC at the protocol-level.

For consent forms that the CoVPN does not manage centrally, each CRS is responsible for developing protocol-specific consent form(s) for local use, based on the sample protocol-specific consent forms. The consent form(s) must be developed in accordance with requirements of the following:

- CRS's IRB/EC and any applicable REs
- CRS's institution
- Elements of informed consent as described in Title 45, CFR Part 46 and Title 21 CFR, Part 50, and in ICH E6(R2), Good Clinical Practice: Consolidated Guidance 4.8

Study sites are strongly encouraged to have local community representatives from the target population review their sites-specific consent forms. This review should include, but should not be limited to, issues of cultural competence, local language considerations, and the level of understandability.

The sample informed consent form(s) include(s) instructions for developing specific content.

9.1.3 Assessment of Understanding

Study staff are responsible for ensuring that participants fully understand the study before enrolling them. This process involves reviewing the informed consent form with the participant, allowing time for the participant to reflect on the procedures and issues presented, and answering all questions completely.

An Assessment of Understanding is used to document the participant's understanding of key concepts in this study. Participants must demonstrate understanding of all questions answered incorrectly. This process and the participant's understanding of the key concepts is to be recorded in the eConsent.

9.2 Pre-enrollment procedures for Main Cohort

Screening may occur over the course of several contacts/visits, up to and including before enrollment. All inclusion and exclusion criteria must be assessed within 56 days before enrollment, unless otherwise specified in the eligibility criteria (or below in this section). Screening results will be reviewed with the volunteer.

After the appropriate informed consent has been obtained and before enrollment, the following procedures are performed:

- Participant will download the electronic diary (eDiary) application to their smartphone or tablet and complete an optional application training. Participant will use eDiary to enter data. Data collection will include:
  - Medical history questionnaire
  - Volunteer demographics in compliance with the NIH Policy on Reporting Race and Ethnicity Data: Subjects in Clinical Research, Aug. 8, 2001 (available at http://grants.nih.gov/grants/guide/notice-files/NOT-OD-01-053.html)
  - Baseline SARS-CoV-2 infection risk information
- Discussion of the eDiary entries done by the participant with the site staff
- Review of materials for possible self-collection of blood by the participant

9.3 Enrollment and vaccination visits for Main Cohort, Immediate Vaccination Group

Once a volunteer has consented to trial participation and is found to meet all eligibility criteria (see Sections 7.1 and 7.2), the CRS requests the randomization assignment via a Web-based randomization system. **Enrollment is simultaneous with randomization**. In general, the time interval between randomization and completion of all study activities for Visit 2 should not exceed approximately 7 working days. However, circumstances may require a participant's visit to be changed. This may exceed the 7-day randomization time limit recommendation.

At vaccination visits, the following procedures are performed **before vaccination**:

- COVID-19 symptom check, both vaccination visits
- Specimen collection (**only at first vaccination** visit, see Appendix E)

Following completion of all procedures in the preceding list, and if results indicate that vaccination may proceed, vaccination is prepared and administered per Moderna EUA Fact Sheet and Full Prescribing Information for Vaccination Providers that can be accessed at https://www.modernatx.com/covid19vaccine-eua/).

Additionally, the participants will be:

- Reminded to enter information in eDiary throughout the study
- Provided with a supply of nasal swabs and shown how to swab both nostrils

9.4 Follow-up procedures for Main Cohort, Immediate Vaccination Group

The following procedures are performed **during follow-up per schedule in** Appendix E:

- Participants reminded to enter information in eDiary throughout the study (see Section 9.16)
- Nasal swab collection by participant
- Blood collection by CRS staff, phlebotomist, or participant
- End of study questionnaire

9.5 Enrollment (visit 2) and Month 2 (visit 4) Visits for Main Cohort, Standard of Care and Vaccine Declined Groups

Randomization applies to **Standard of Care participants** only: Once a volunteer has consented to trial participation and is found to meet all eligibility criteria (see Sections 7.1 and 7.2), the CRS requests the randomization assignment via a Web-based randomization system. **Enrollment is simultaneous with randomization**. In general, the time interval between randomization and completion of all study activities for Visit 2 should not exceed approximately 7 working days. However, circumstances may require a participant's visit to be changed. This may exceed the 7-day randomization time limit recommendation.

For **Vaccine Declined participants**: Once a volunteer has consented to trial participation and is found to meet all eligibility criteria (see Sections 7.3, and 7.4) they are considered enrolled.

For Standard of Care and Vaccine Declined participants: before leaving the clinic, the participants will be:

- Reminded to enter information in eDiary throughout the study
- Provided with a supply of nasal swabs and shown how to swab both nostrils

At visits 2 and 4, the following procedures are performed **per schedule in** Appendix F:

- eDiary entries by participant (see Section 9.16)
- Nasal swab collection by participant
- Blood collection by CRS staff, phlebotomist, or participant

9.6 Month 4 (visit 5) and Month 5 (visit 6) Visits for Main Cohort, Standard of Care Group and Vaccine Declined Group

For those participants who have not received a COVID-19 vaccine outside the study and who request vaccination:

At vaccination visits the following procedures are performed **before vaccination**:

- COVID-19 symptom check, both visits
- Specimen collection (**only at Month 4** visit, see Appendix F)

Following completion of all procedures in the preceding list, and if results indicate that vaccination may proceed, vaccination is prepared and administered, per Moderna EUA Fact Sheet and Full Prescribing Information for Vaccination Providers that can be accessed at https://www.modernatx.com/covid19vaccine-eua/).

- End of study questionnaire at Month 5 (Visit 6)

For participants who have already received COVID-19 vaccination outside of study prior to Month 4 (Visit 5) visit or who decline vaccination, the following procedures are performed at Visit 5; Visit 6 will not occur (see Appendix F):

- Specimen collection
- End of Study questionnaire

9.7 Procedures for Main Cohort participant diagnosed with a SARS-CoV-2 infection during the study

If a participant is diagnosed with a SARS-CoV-2 infection during the course of the study, CDC guidelines on administration of vaccination will be followed.

Main Cohort participants who are clinically diagnosed with SARS-CoV-2 infection (from any test result indicative of acute infection) are referred to as clinically diagnosed index cases. They will be instructed to isolate per local guidelines and the following procedures will be performed:

- eDiary collection of daily symptoms of COVID-19 and assessment of whether clinical care was sought or hospitalization occurred (via self-report and/or request for medical records) through an eDiary for a period of 14 days or until symptoms have resolved, whichever is longer
- Daily nasal swabbing continued per schedule of procedures (see Appendix E)
- Blood collection by phlebotomist or participant for SARS-CoV-2 clinical serology
- Close contacts identification (see Section 9.9)

Following resolution of the isolation period, participants will continue scheduled study visits. Follow-up duration for participants diagnosed with moderate or severe COVID-19 disease may be adjusted in consultation with the CRS (eg, to avoid interference with participant initiation of SARS-CoV-2 treatment).

9.8 Enrollment and follow-up procedures for Prospective Close Contact (PCC) cohort

Upon enrollment, participants from the Main Cohort will be asked to invite individuals with whom they will be in frequent close physical proximity during the study to join the PCC cohort (See Section 7.5 and CoVPN 3006 SSP for additional information on PCC cohort). New PCC contacts may also be added during the study.

Prospective close contacts will be given referral codes and information about how to enroll in the study as part of the PCC cohort. If they choose to enroll and sign an informed consent, they will be asked to:

- provide access to their routine SARS-CoV-2 testing reports, if applicable
- enter data weekly through eDiary (see Section 9.16)

If a PCC cohort participant or the Main Cohort participant to whom he/she is connected is diagnosed with a SARS-CoV-2 infection during the course of the study, the PCC cohort participant will be instructed to perform the following procedures (see Appendix G):

- eDiary collection of daily symptoms of COVID-19 and assessment of whether clinical care was sought or hospitalization occurred (via self-report and/or request for medical records) through an eDiary for a period of 14 days or until symptoms have resolved, whichever is longer
- provide nasal swab for 14 days
- self-collect blood samples on day 1 and day 29, relative to the notification of infection

For Main Cohort participants who are also PCC cohort participants related to an index case in another Main cohort participant, the following procedures in addition to those listed in Appendix E may be performed:

- one additional blood collection (self-collection) as soon as possible following the report of the index case
- eDiary collection of information regarding exposure to the Index case
- eDiary collection of daily symptoms of COVID-19 and assessment of whether clinical care was sought or hospitalization occurred (via self-report and/or request for medical records) through an eDiary for a period of 14 days or until symptoms have resolved, whichever is longer

See CoVPN 3006 SSP for additional information on PCC cohort.

9.9 Enrollment and Follow-up procedures for Case-Ascertained Close Contact (CACC) cohort

Participants from the Main Cohort who are clinically diagnosed with SARS-CoV-2 (referred to as the Index case) during the study will be asked to contact any individuals with whom they have been in close contact within about 72 hours of the date of the SARS-CoV-2 diagnosis test. See Section 7.6 and CoVPN 3006 SSP for additional information on case-ascertained close contacts.

Potential CACC cohort participants will be given referral codes and information about how to enroll in the study as part of the CACC cohort, if they choose to, and sign an informed consent.

Once a volunteer has consented to be a part of CACC cohort, the following procedures will be performed (see Appendix H):

- eDiary collection of information regarding exposure to the Index case
- eDiary collection of daily symptoms of COVID-19 and assessment of whether clinical care was sought or hospitalization occurred (via self-report and/or request for medical records) through an eDiary for a period of 14 days or until symptoms have resolved, whichever is longer.
- Outcomes of SARS-CoV-2 testing performed by the school or other facilities
- Nasal swab self-collection from day 1-14
- Blood collection by participant for SARS-CoV-2 clinical serology at day 3 and at day 29

If a CACC participant is diagnosed with a SARS-CoV-2 infection during the course of the study, they will be instructed to track daily symptoms of COVID-19 through an eDiary for a period of 14 days or until symptoms have resolved, whichever is longer.

For Main Cohort participants who are also CACC cohort participants, the following procedures in addition to those listed in Appendix E may be performed:

- one additional blood collection (self-collection) as soon as possible
- eDiary collection of information regarding exposure to the Index case
- eDiary collection of daily symptoms of COVID-19 and assessment of whether clinical care was sought or hospitalization (via self-report and/or request for medical records) occurred through an eDiary for a period of 14 days or until symptoms have resolved, whichever is longer.

9.10 Visit windows and missed visits

Visit windows are included in Appendix I. The procedures for documenting missed visits and out of window visits are described in CoVPN 3006 SSP.

9.11 Discontinuing vaccination for a participant who receives a COVID-19 Vaccine outside the study

In the event a participant receives an EUA or licensed COVID-19 vaccine outside the study, an individual participant's study vaccinations will be permanently discontinued. Participants should be counseled on the importance of continuing with the study and strongly encouraged to participate in follow-up visits and protocol-related procedures per the protocol for the remainder of the trial, unless medically contraindicated.

9.12 Early termination visit

In the event of early participant termination, site staff should consider if final specimen collection is appropriate.

9.13 Pregnancy for Main Cohort

For Main cohort participants who have been vaccinated, pregnancies and pregnancy outcomes will be reported via the FDA/CDC Vaccine Adverse Event Reporting System (VAERS) (see Section 11.1).

9.14 SARS-CoV-2 monitoring by schools for Main Cohort, if applicable

For participating schools that have SARS-CoV-2 monitoring plans in place, monitoring will typically provide approximately twice weekly SARS-CoV-2 testing for enrolled Main Cohort participants. For any positive test result, the institution will inform the student (initiating isolation), will provide the SARS-CoV-2 test results to the students, and will carry out contact tracing if applicable. Participants will be instructed to report positive test results via the eDiary.

9.15 COVID-19 symptom surveillance for Main Cohort

Participants will be monitored for symptoms of COVID-19 throughout the course of the study via a weekly eDiary. These symptoms will be used to establish whether a participant meets criteria for COVID-19 disease as defined by Secondary Endpoint 4. If a participant has symptoms consistent with COVID-19, they will be encouraged to seek testing and clinical care through their local testing and care facilities. Results of testing will be reported through the eDiary and participants who have positive test results will follow procedures described in Section 9.7.

For Immediate Vaccination Group only:due to the overlap of symptoms that can result from vaccination and symptoms of COVID-19, monitoring will begin 7 days following the first study product administration. Symptom monitoring will be paused 7 days following the second product administration.

9.16 Use of electronic Diary (eDiary) for Clinical Outcomes Assessments

At the time of consent, participants must confirm they will be willing to complete an eDiary collection of study data (see below for details of data to be collected).

The eDiary will be completed using an application downloaded to their smartphone or tablet. This study will utilize the Medidata Patient Cloud

Application as the eDiary software. This application allows for real-time data collection directly from participants.

After participants have signed the informed consent, all participants will be instructed to download the eDiary application on their personal smartphone or tablet. The eDiary application has a self-training component, enabling immediate use. The site staff will perform any retraining as necessary.

For Main Cohort participants,

Study participants will record data in the eDiary throughout the study as described in Appendix E and Appendix F, following the prompts provided for data collection. eDiary data collection areas may include:

- Medical history questionnaire
- Volunteer demographics
- Risk assessment for SARS-CoV-2 infection (see Appendix E and Appendix F)
- Surveillance for symptoms of COVID-19 (see Section 9.15)
- Self-collection of samples (eg, nasal swabs) (see Appendix E and Appendix F)
- Outcomes of SARS-CoV-2 testing performed by the school or other facilities (see Appendix E and Appendix F)
- COVID-19 symptom tracking upon incident diagnosis of SARS-CoV-2 infection, including assessments of whether clinical care was sought or hospitalization occurred via self-report and/or request for medical records (see Section 9.7)

Additionally, participants will be reminded through the eDiary of the need to continue practices for reducing acquisition and transmission of SARS-Cov-2 regardless of their vaccination status.

For Prospective Close Contact (PCC) cohort participants,

PCC participants record data in the eDiary as described in Appendix G, following the prompts provided for data collection. eDiary data collection areas may include:

- Collection of demographic information
- Collection of information regarding exposure to the main study participant
- Outcomes of SARS-CoV-2 testing performed by the school or other facilities

- Self-collection of samples (eg, nasal swabs) (see Appendix G)
- COVID-19 symptom tracking upon incident diagnosis of SARS-CoV-2 infection in PCC or main study participant, including assessments of whether clinical care was sought or hospitalization occurred via self-report and/or request for medical records
- Recording of any COVID-19 vaccines received outside of the study

For Case-Ascertained Close Contact (CACC) cohort participants,

CACC participants will record data in the eDiary as described in Appendix H, following the prompts provided for data collection. eDiary data collection areas may include:

- Collection of demographic information
- Collection of information regarding exposure to the Index case
- Outcomes of SARS-CoV-2 testing performed by the school or other facilities
- COVID-19 symptom tracking, including assessments of whether clinical care was sought or hospitalization occurred via self-report and/or request for medical records
- Self-collection of samples (eg, nasal swabs) (see Appendix H)
- Recording of any COVID-19 vaccines received outside of the study

10 Laboratory

10.1 CRS laboratory procedures

The CoVPN 3006 SSP and other study materials provide further guidelines for operational issues concerning the clinics and laboratories. These documents include special considerations for phlebotomy and guidelines for general specimen collection, specimen labeling and specimen processing.

Tube types for blood collection are specified in CoVPN 3006 SSP.

Of note, all assays described below, with the exception of SARS-CoV-2 PCR, are performed as research assays to evaluate the ability of the SARS-CoV-2 virus to induce immune responses in the context of the participants' genetic background and are not approved for use in medical care. Results from these research assays are not made available to participants or medical professionals to guide treatment decisions.

10.2 Total blood volume

Required blood collections per visit are shown in Appendix E, Appendix F, Appendix G and Appendix H. The total blood volume drawn for each participant will not exceed 500 mL in any 56-day (8-week) period.

10.3 Endpoint assays

10.3.1 SARS-CoV-2 PCR

Real-time reverse transcription polymerase chain reaction (RT-PCR) assays to detect SARS-CoV-2 will be run on nasal samples collected from study participants. After RNA extraction, specimens will be analyzed by a laboratory developed test using the World Health Organization's E/RdRp primer set, the N1/N2 primer set from the Centers for Disease Control and Prevention, or tests from Hologic (Panther Fusion), DiaSorin (Simplexa), or Roche (cobas). Novel methods to detect SARS-CoV-2 may also be used if applicable.

10.3.2 SARS-CoV-2 viral sequencing

Genome sequencing of SARS-CoV-2 virus may be performed on nasal samples from study participants using a metagenomic approach. RNA from positive specimens is converted to a cDNA library by reverse transcription and the library is sequenced. Resulting consensus sequences are assembled against a SARS-CoV-2 reference genome.

10.3.3 Neutralizing antibody assay (nAb)

SARS-CoV-2–specific nAb assays may be performed on serum samples from study participants. The assay will test neutralization of S-pseudotyped and/or full-genome recombinant viruses as measured by a reduction in luciferase (Luc) reported gene expression after infection in ACE-2 positive cells.

10.3.4 Binding antibody multiplex assay (BAMA)

SARS-CoV-2 specific total IgG and IgM binding antibodies may be assessed in serum samples. In addition, SARS-CoV-2–specific serum IgA and IgG subclass (IgG1, IgG2, IgG3, and IgG4) antibodies may also be assessed. Epitope specificity by ACE-2 blocking assay and responses to endemic coronavirus proteins may also be assessed.

10.3.5 Antibody avidity (BioLayer Interferometry and BAMA avidity index)

SARS-CoV-2-specific polyclonal serum antibody and IgG subclass avidity may be measured using BAMA with the addition of a dissociation step to calculate the antibody avidity index (BAMA-AI). BLI and/or SPR technologies may also be used to measure antibody avidity.

10.3.6 Fc-Mediated Antibody Function

Additional assays to measure SARS-CoV-2-specific antibody Fc functions may be performed (ie, antibody dependent cellular cytotoxicity [ADCC], antibody-dependent cellular phagocytosis [ADCP], antibody-dependent neutrophil phagocytosis [ADNP], binding antibody FcR array, and S-protein-Expressing Cell Antibody Binding Assay [SECABA]).

10.4 Lab assay portfolio

Additional assays may be performed per the Laboratory Center assay portfolio, which includes immune assessments such as those for cellular, humoral, and innate immune responses, and host genetics. The assay portfolio will be updated periodically to include new assays and adjust qualification levels of existing assays.

10.5 Exploratory studies

Samples may be used for other testing and research related to furthering the understanding of SARS-CoV-2 immunology, virology, or vaccines. In addition, cryopreserved samples may be used to perform additional assays to support standardization and validation of existing or newly developed methods.

10.6 Specimen storage and other use of specimens

The CoVPN stores specimens from all study participants indefinitely, unless a participant requests that specimens be destroyed or if required by IRB/EC, or RE.

Other use of specimens is defined as studies not covered by the protocol or the informed consent forms for the study (see Appendix A, Appendix B, Appendix C, and Appendix D).

This research may relate to SARS-CoV-2, vaccines, coronaviruses, the immune system, and other diseases. This could include genetic testing and, potentially, genome-wide studies. This research is done only to the extent authorized in each study site's informed consent form, or as otherwise authorized under applicable law. Other research on specimens ("other use") will occur only after review and approval by the CoVPN, the IRB/EC of the researcher requesting the specimens, and the IRBs/ECs/REs of the CRSs if required.

As part of consenting for the study, participants document their initial decision to allow or not allow their specimens to be used in other research, and they may change their decision at any time. The participant's initial decision about other use of their specimens, and any later change to that decision, is recorded by their CRS in a Web-based tool that documents their current decisions for other use of their specimens. The CoVPN will only allow other research to be done on specimens from participants who allow such use.

CRSs must notify CoVPN Regulatory Affairs if institutional or local governmental requirements pose a conflict with or impose restrictions on specimen storage or other use of specimens.

10.7 Biohazard containment

The transmission of blood-borne pathogens can occur through contact with contaminated needles, blood, and blood products, and the transmission of SARS-CoV-2 and other respiratory pathogens may occur through contact with contaminated respiratory droplets, aerosols, and other biological materials. Appropriate precautions will be employed by all personnel in the collection, shipping, and handling of all specimens for this study, as currently recommended by local health authorities, the CDC, the NIH, or other applicable agencies.

All dangerous goods materials, including Biological Substances, Category A or Category B, must be transported according to instructions detailed in the International Air Transport Association Dangerous Goods Regulations.

11 Safety Reporting

11.1 FDA/CDC Vaccine Adverse Event Reporting System (VAERS)

The Vaccine Adverse Event Reporting System (VAERS) is co-managed by the U.S. CDC and FDA and serves as the reporting mechanism for adverse events occurring from licensed and EUA vaccines, including the Moderna COVID-19 vaccine being used in this trial. VAERS accepts and analyzes reports of adverse events (AEs) after a person has received a vaccination. It is accessed at https://vaers.hhs.gov/reportevent.html.

Risks associated with receipt of the Moderna COVID-19 Vaccine and AE reporting requirements are described in the "Fact Sheet For Healthcare Providers Administering Vaccine (Vaccination Providers) Emergency Use Authorization (EUA) Of The Moderna COVID-19 Vaccine To Prevent Coronavirus Disease 2019 (COVID-19)", available at https://www.modernatx.com/covid19vaccine-eua/.

All vaccination providers, including those at the CRS, are required to report to VAERS the following adverse events after COVID-19 vaccination, under Emergency Use Authorization (EUA), and other adverse events if later revised by CDC |(https://www.cdc.gov/vaccines/covid-19/vaccination-provider-support.html and https://vaers.hhs.gov/faq.html):

- Vaccine administration errors, whether or not associated with an adverse event (AE)
- Serious AEs regardless of causality. Serious AEs per FDA are defined as:
  - Death;
  - A life-threatening AE;
  - Inpatient hospitalization or prolongation of existing hospitalization;
  - A persistent or significant incapacity or substantial disruption of the ability to conduct normal life functions;
  - A congenital anomaly/birth defect;
  - An important medical event that based on appropriate medical judgement may jeopardize the individual and may require medical or surgical intervention to prevent one of the outcomes listed above.
- Cases of Multisystem Inflammatory Syndrome
- Cases of COVID-19 that result in hospitalization or death

CRS vaccination providers are encouraged to report to VAERS any additional clinically significant AEs following vaccination, even if they are not sure if vaccination caused the event; and are to report any additional select AEs and/or any revised safety reporting requirements per FDA's conditions of authorized use of vaccine(s) throughout the duration of any COVID-19 Vaccine being authorized under an EUA.

CRS vaccination providers will complete and submit reports to VAERS online at https://vaers.hhs.gov/reportevent.html. Further assistance with reporting to VAERS is accessed by calling 1-800-822-7967. The reports should include the words "Moderna COVID- 19 Vaccine EUA" in the description section of the report.

As indicated in the "Fact Sheet For Healthcare Providers Administering Vaccine (Vaccination Providers) Emergency Use Authorization (EUA) Of The Moderna COVID-19 Vaccine To Prevent Coronavirus Disease 2019 (COVID-19)", vaccination providers (including those at the CRS) are encouraged, to the extent feasible, to report adverse events to Moderna.

11.2 Reporting safety events to v-safe

An additional source of VAERS reports will be through a program administered by the CDC known as v-safe (https://www.cdc.gov/coronavirus/2019-ncov/vaccines/safety/vsafe.html). V-safe is a smartphone-based opt-in program that uses text messaging and web surveys from CDC to check in with vaccine recipients for health problems following COVID-19 vaccination. The system also will provide telephone follow-up to anyone who reports medically significant (important) adverse events. Responses indicating missed work, inability to do normal daily activities, or that the recipient received care from a doctor or other healthcare professional will trigger the VAERS Call Center to reach out to the participant and collect information for a VAERS report, if appropriate. CRS vaccination providers will encourage the study participants receiving the Moderna COVID-19 vaccine to use v-safe.

12 Protocol conduct

This protocol and all actions and activities connected with it will be conducted in compliance with the principles of GCP (ICH E6 (R2)), and according to NIAID and CoVPN policies and procedures as specified in the CoVPN 3006 SSP, NIAID Clinical Research Policies and Standard Procedures Documents including procedures for the following:

- Protocol registration, activation, and implementation;
- Informed consent, screening, and enrollment;
- Study participant reimbursement;
- Clinical and safety assessments;
- Safety monitoring and reporting;
- Data collection, documentation, transfer, and storage;
- Participant confidentiality;
- Study follow-up and close-out;
- Unblinding of staff and participants;
- Quality control;
- Protocol monitoring and compliance;
- Specimen collection, processing, and analysis;
- Exploratory and ancillary studies and sub-studies, and
- Destruction of specimens.

Any policies or procedures that vary from NIAID and CoVPN standards or require additional instructions (eg, instructions for randomization specific to this study) will be described in the CoVPN 3006 SSP.

12.1 Study termination

NIAID reserves the right to terminate or curtail a clinical study for any reason, including but not limited to the following (reference: https://grants.nih.gov/grants/guide/rfa-files/RFA-AI-12-012.html):

- risk to subject safety
- the scientific question is no longer relevant or the objectives will not be met (ie, slow accrual)
- failure to comply with GCP, U.S. Federal regulations, or Terms and Conditions of Award
- occurrence of unforeseen drug safety issues or data from preclinical studies indicate a presence of unanticipated toxicity
- risks that cannot be adequately quantified
- ethical concerns raised by the local community or local medical care/health care authorities
- failure to remedy deficiencies identified through site monitoring
- substandard data
- reaching a major study endpoint substantially before schedule with persuasive statistical significance.

This study may also be terminated early by the determination of the FDA, the United States Department of Health and Human Services Office for Human Research Protections (OHRP). In addition, the conduct of this study at an individual CRS may be terminated by the determination of the IRB/EC and any applicable RE.

12.2 Emergency communication with study participants

As in all clinical research, this study may generate a need to reach participants quickly to avoid imminent harm, or to report study findings that may otherwise concern their health or welfare and their willingness to remain on study.

When such communication is needed, the CRS will request that its IRB/EC and any applicable RE expedite review of the message. If this review cannot be completed in a timeframe consistent with the urgency of the required communication, the site can contact the participant without IRB/EC approval if such communication is necessary to avoid imminent harm to the study participant. The CRS must notify the IRB/EC and any applicable RE of the matter as soon as possible.

13 Version history

The Protocol Team may modify the original version of the protocol. Modifications are made to CoVPN protocols via clarification memos, letters of amendment, or full protocol amendments.

The version history of, and modifications to, Protocol CoVPN 3006 are described below.

Protocol history and modifications

Date: May 20, 2021

Protocol version: Version 4.0
Protocol modification: Full Protocol Amendment 3

Item 1 Revised in Title page, Section 1, Overview, Section 2, Background, Section 6, Statistical considerations, Section 7.1, Inclusion criteria for main cohort, Appendices A, C, and D, Sample informed consent forms, and Appendices E-H, Procedures: definition of Main cohort participant expanded to include non-student adults aged 18 through 29

Item 2 Revised throughout the protocol: delayed vaccination group renamed standard of care group

Item 3 Added in Section 1, Overview, Section 2, Background, Section 6, Statistical considerations, Section 7.1 and 7.2, Inclusion and Exclusion criteria for Main Cohort, Immediate Vaccination and Standard of Care groups, Section 7.3, Inclusion criteria for Main cohort, Vaccine Declined group, Section 7.4, Exclusion criteria for Main cohort, Vaccine Declined Group, Section 9, Clinical procedures, Appendix B, Sample informed consent form for Main cohort, Vaccine Declined Group, Appendices A, C and D, Sample informed consent forms, and Appendix I, Visit windows: new observational group, “Vaccine Declined” group, of up to 6000 participants

Item 4 Added in Section 7.2, Exclusion criteria for Main Cohort, Immediate Vaccination Group and Standard of Care Group: exclusion for volunteers who prefer to be vaccinated immediately

Item 5 Revised in Section 1, Overview and Appendices A, C, and D, Sample informed consent forms: number of potential participants in close contact cohorts increased

Item 6 Updated in Section 1, Overview: increased estimated total study duration from 7 months to 9 months

Item 7 Clarified in Section 2.10, Moderna COVID-19 Vaccine, and Section 8.3, Acquisition of Moderna COVID-19 Vaccine: participants may also access Moderna COVID-19 EUA vaccine via other sources

Item 8 Revised in Section 3, Objectives and endpoints and Section 6, Statistical considerations: inclusion of all SARS-CoV-2 infection events in analysis for primary endpoints and secondary endpoints 1-5

Item 9	Revised in Section 3, Objectives and endpoints, exploratory objective 1: expanded to main study cohort
Item 10	Revised in Section 7.1, Inclusion criteria for Main Cohort, Immediate Vaccination Group and Standard of Care Group: timing of completion of assessment of understanding
Item 11	Revised in Sections 7.1, 7.3, 7.5, and 7.6, Inclusion criteria for all groups: access to device and internet is needed for completion of study procedures
Item 12	Added in Section 8, Study product: definitions of on-study and outside of study vaccination
Item 13	Clarified in Section 8, Study product: study allocated vaccine can only be administered to study participants
Item 14	Added in Section 8.3, Acquisition of Moderna COVID-19 Vaccine: participants can access Moderna COVID-19 EUA vaccine via other sources for on-study vaccinations if the administration fee is either paid for by the study or is not billed to any party
Item 15	Deleted from Section 9.3, Enrollment and vaccination visits for Main Cohort, Immediate Vaccination Group and Section 9.6, Month 4 (visit 5) and Month 5 (visit 6) Visits for Main Cohort, Standard of Care Group and Vaccine Declined Group: parenthetical reference to temperature in COVID-19 symptom check
Item 16	Added in Section 9, Clinical Procedures, and Appendix F, Procedures for Main Cohort, Standard of Care and Vaccine Declined Groups: end of study questionnaires as procedures
Item 17	Added in Section 9.1.2, Protocol-specific consent forms: consent forms for close contact cohorts may be managed centrally by the CoVPN
Item 18	Clarified in Section 9.15, COVID-19 symptom surveillance for Main Cohort: symptom overlap between COVID-19 and vaccination is only applicable to Immediate Vaccination Group
Item 19	Corrected in Appendix A, Sample informed consent form for the Main cohort, Immediate Vaccination and Standard of Care Groups, Appendix C, Sample informed consent form for Prospective Close Contact (PCC) cohort and Appendix D, Sample informed consent form for Case-Ascertained Close Contact (CACC) cohort: frequency of eDiary entries.
Item 20	Added in Appendix A, Sample informed consent form for the Main cohort, Immediate Vaccination and Standard of Care Groups: we may ask Main cohort participants to provide contact information for potential close contact cohort volunteers
Item 21	Clarified in Appendix A, Sample informed consent form for the Main cohort, Immediate Vaccination and Standard of Care Groups: Month 4 will be the last study visit for Standard of Care participants who receive COVID-19 vaccine outside the study prior to Month 4
Item 22	Added in Appendix A, Sample informed consent form for the Main cohort, Immediate Vaccination and Standard of Care Groups: the risk of delaying COVID-19 vaccination

Item 23 Added in Appendix C, Sample informed consent form for Prospective Close Contact (PCC) cohort and Appendix D, Sample informed consent form for Case-Ascertained Close Contact (CACC) cohort: new section "Risks of COVID-19"

Item 24 Deleted from Appendices A, C and D, Sample informed consent forms: sentence that states we will not share your name or identifying information with the CoVPN

Item 25 Clarified in Appendix A, Sample informed consent form for the Main cohort, Immediate Vaccination and Standard of Care Groups: benefits of COVID-19 vaccination

Item 26 Added in Appendices A, C, and D, Sample informed consent forms: participants will be prompted to scan the barcode on nasal swabs

Item 27 Updated in Appendices A, C, and D, Sample informed consent forms: recommendation to follow federal, state, and local guidelines for SARS-CoV-2 prevention

Item 28 Updated in Appendices A, C, and D, Sample informed consent forms: contact information for Advarra IRB

Item 29 Updated in Appendix A, Sample informed consent form for the Main cohort, Immediate Vaccination and Standard of Care Groups: Moderna COVID-19 Vaccine, risks and number of vaccinated people

Item 30 Updated Title page and Section 13, Version history: contents of this amendment

Item 31 Throughout the protocol: minor editorial changes, clarifications, and corrections

Date: April 12, 2021

Protocol version: 3.0
Protocol modification: Full protocol amendment 2

Item 1 Added in Section 1, *Overview* and Section 6, *Statistical considerations*: Clinical Research Sites (CRSs) that can participate in the study expanded to include non-university affiliated CoVPN Clinical Research Sites (CRSs)

Item 2 Revised in Section 1, *Overview*, Section 2, *Background*, Section 6, *Statistical considerations*, Section 7.1, *Inclusion criteria for main cohort*, Section 9, *Clinical procedures*, and Appendix A, *Sample informed consent form for the Main cohort*: definition of participant expanded to include students enrolled in any post-secondary educational institution

Item 3 Revised in Section 1, *Overview*, Section 2, *Background*, Section 6, *Statistical considerations,* Section 7.3*, Inclusion criteria for Prospective Close Contact (PCC) cohort,* Section 9*, Clinical procedures,* and Appendices A, B, D, E, F: University SARS-CoV-2 testing is not required

Item 4 Revised in Section 9.3*, Enrollment and vaccination visits for Main Cohort, Immediate Vaccination Group*, Section 9.5, *Enrollment (visit 2) and visit 4 (Month 2) for Main Cohort, Delayed Vaccination Group* and Appendix H,

Visit windows: maximum recommended time between randomization and completion of Visit 2 activities increased from 4 days to 7 days

Item 5 Clarified in Section 2, *Background*, Section 6, *Statistical considerations*, and Section 9.7, *Procedures for Main Cohort participant diagnosed with a SARS-CoV-2 infection during the study*: any SARS-CoV-2 test result indicative of acute infection (on or off study) obtained from a Main cohort participant will trigger "index-case" activities

Item 6 Clarified in Section 2, *Background*, Section 6.4.3, *Vaccine efficacy (VE) analyses against SARS-CoV-2 infection*, Section 7.1, *Inclusion criteria for main cohort*, Section 9, *Clinical procedures*, Appendix A, *Sample informed consent form for Main cohort*, and Appendix B, *Sample informed consent form for Prospective Close Contact (PCC) cohort*: participants can choose to obtain an EUA or licensed COVID-19 vaccine outside the study

Item 7 Added to Section 3, *Objectives and endpoints*: 2 endpoints for the duration of viral shedding specified in corresponding secondary objective 4

Item 8 Clarified in Section 6, *Statistical considerations, 7th paragraph:* primary analysis occurs when participants complete 4 months of follow-up

Item 9 Clarified in Table 6-1, *Study analysis sets and definitions*: Day 29 is visit 3 for immediate vaccination arm and will be define in Statistical analysis plan (SAP) for delayed vaccination arm

Item 10 Revised in Section 6.3, *Randomization*: randomization evaluated within levels defined by study site and type of residence

Item 11 Clarified in Section 6.4.3.1, *Primary VE Analysis*: rationale for choice of analysis

Item 12 Revised in Section 6.4.5.2, *Analyses of the vaccine effect on secondary transmission*: details of the derivation

Item 13 Revised in Section 7.2, Exclusion criterion #1 for Main Cohort: removed febrile assessment

Item 14 Clarified in Section 2.10, *Moderna COVID-19 Vaccine*, and Section 8.3, *Acquisition of Moderna COVID-19 Vaccine*: Moderna COVID-19 Vaccine provided under EUA and allocated for this trial

Item 15 Corrected in Section 9, *Clinical procedures*: CRS staff will obtain informed consent of participants according to the COVPN 3006 SSP

Item 16 Corrected in Appendix A, *Sample informed consent form for Main cohort:* blood will be collected 3 times for delayed vaccination arm

Item 17 Added to Appendix B, *Sample informed consent form for Prospective Close Contact (PCC)* cohort: COVID-19 care is not paid for by the study

Item 18 Added to Appendices A, B, C, *Sample informed consent forms*: information regarding employee/family member participation

Item 19 Added to Appendices A, B, C, *Sample informed consent forms*: terms of use for data collection via web-based app

Item 20 Deleted in Appendix B, *Sample informed consent form for Prospective Close Contact (PCC)* cohort, and Appendix C, Sample informed consent

form for Case-Ascertained Close Contact (CACC) cohort, item 8: text referring to vaccine receipt

Item 21 Added to Appendix H, *Visit windows for CACC:* upper allowable window for blood specimen collection

Item 22 Added changes per Protocol v 2.0, Letter of Amendment 1

Item 23 Updated Title page and Section 13, *Version history*: contents of this amendment

Item 24 Updated Section 1.1, *Protocol team*: deletion of Site Principal Investigator representative

Item 25 Throughout the protocol: minor editorial changes, clarifications, and corrections

Date: March 10, 2021

Protocol version: Version 2.0
Protocol modification: Letter of Amendment 1

Item 1 Added in Section 10.1, *CRS laboratory procedures*, Appendices A, B, and C, *Sample informed consent forms*: return of SARS-CoV-2 nasal swab testing results to participants

Item 2 Added to Section 1.1, *Protocol team*: Jasmine Marcelin, co-chair and Jeffrey L Carson, Site Principal Investigator representative

Date: February 26, 2021

Protocol version: 2.0
Protocol modification: Full protocol amendment 1

Item 1 Revised in Section 3, *Objective and Endpoints,* Section 6, *Statistical considerations,* Section 9, *Clinical procedures*, Appendix A, *Sample informed consent form for the main cohort*, and Appendices D and E, *Procedures for the main cohort*: Month 3 visit removed for both Main cohort groups, M1 visit removed for delayed vaccination group of Main cohort, and Month 1 blood collection removed for immediate vaccination group of Main cohort

Item 2 Added in Section 1, *Overview*: 2 new endpoint laboratories

Item 3 Updated in Section 1.1, *Protocol team*: team members

Item 4 Deleted Section 6.1, *Accrual restrictions*: no accrual restrictions for this study

Item 5 Revised in Section 6.4, *Statistical analyses*: minor clarifying text revisions

Item 6 Corrected in Section 6.4.4.7, *VL analyses,* 3rd paragraph: reference to secondary endpoint number

Item 7 Deleted in Section 9.3, *Enrollment and vaccination visits for Main Cohort, Immediate Vaccination Group* and Section 9.5, *Enrollment (visit 2) and visit 4 (Month 2) for Main Cohort, Delayed Vaccination Group:* sentence regarding clinical lab results

Item 8 Added in Section 9.15, *Use of electronic Diary (eDiary) for Clinical Outcomes Assessments,* and Appendices F through G, *Procedures*: end of study questionnaire regarding COVID vaccines received outside the study

Item 9 Added in Appendices A, B, and C, *Sample informed consent forms*, section "Being in the study": participants will take an end of study questionnaire

Item 10 Added in Appendix B, *Sample informed consent form for Prospective Close Contact (PCC) cohort*, and Appendix C, *Sample informed consent form for Case-Ascertained Close Contact (CACC) cohort*, section "Being in the study": applicable participants will receive SARS-CoV-2 screening at the University about two times per week.

Item 11 Deleted in Appendices A, B, and C, *Sample informed consents,* second sentence*:* reference to alternate study name of "vPROTECT"

Item 12 Added in Appendix A, *Sample informed consent form for the main cohort:* additional information for participant regarding text or email reminders for nasal swabs

Item 13 Deleted in Appendices A, B and C, *Sample informed consents:* reference to "electronically" for signature

Item 14 Deleted in Appendices A, B and C, *Sample informed consents*, signature table: column for denoting time of signature

Item 15 Deleted in Appendix A, *Sample informed consent form for the main cohort*, section 8: redundant information

Item 16 Corrected in in Appendix A, *Sample informed consent form for the main cohort*, section10: VAERS reporting requirements

Item 17 Revised in Appendices F and G: table text and associated footnotes

Item 18 Revised Appendix H, *Visit windows,* Case-Ascertained Close Contact (CACC) cohort, to include applicable Prospective Close Contact (PCC) participants who test positive for SARS-CoV-2 or whose Main Cohort study contact tests positive for SARS-CoV-2

Item 19 Revised throughout protocol: minor grammatical, editorial, and formatting corrections.

Item 20 Updated Title page, Table of Contents, Section 13, *Version history*, and Appendix I*, Protocol signature page*: dates, version number, and contents of this amendment

Date: February 17, 2021

Protocol version:1.0
Protocol modification: Not applicable

Original protocol

14 Document references (other than literature citations)

Other documents referred to in this protocol, and containing information relevant to the conduct of this study, include:

- Assessment of Understanding. Accessible through the CoVPN protocol-specific website.
- CDC COVID-19 Vaccination Program Provider Requirements and Support. Available at https://www.cdc.gov/vaccines/covid-19/vaccination-provider-support.html
- CoVPN Certificate of Confidentiality. Accessible through the CoVPN website.
- CoVPN 3006 Study Specific Procedures. Accessible through the CoVPN protocol-specific website.
- Dangerous Goods Regulations (updated annually), International Air Transport Association. Available for purchase at https://www.iata.org/publications/dgr/Pages/index.aspx
- International Council on Harmonisation of Technical Requirements for Pharmaceuticals for Human Use (ICH) E6 (R2), Guideline for Good Clinical Practice: Section 4.8, Informed consent of trial subjects. Available at http://www.ich.org/page/efficacy-guidelines
- Laboratory Center assay portfolio
- Moderna COVID-19 Vaccine EUA Fact Sheet and Full PI for Vaccination Providers. Available at https://www.modernatx.com/covid19vaccine-eua/.
- Participants' Bill of Rights and Responsibilities. Accessible through the CoVPN protocol-specific website.
- NIH Policy on Reporting Race and Ethnicity Data: Subjects in Clinical Research. Available at https://grants.nih.gov/grants/guide/notice-files/NOT-OD-01-053.html
- Title 21, Code of Federal Regulations, Part 50. Available at http://www.accessdata.fda.gov/scripts/cdrh/cfdocs/cfcfr/CFRSearch.cfm?CFRPart=50

- Title 45, Code of Federal Regulations, Part 46 (2018 requirements). Current requirements available at https://www.hhs.gov/ohrp/regulations-and-policy/regulations/45-cfr-46/index.html

- VAERS, Vaccine Adverse Event Reporting System. Available at https://vaers.hhs.gov/reportevent.html

- VAERS, Vaccine Adverse Event Reporting System FAQ. Available at https://vaers.hhs.gov/faq.html

- V-safe After Vaccination Health Checker. Available at https://www.cdc.gov/coronavirus/2019-ncov/vaccines/safety/vsafe.html

See Section 16 for literature cited in the background and statistics sections of this protocol.

15 Acronyms and abbreviations

Ab	antibody
ACIP	Advisory Committee on Immunization Practices
AE	adverse event
AUC	area under the curve
BAMA	binding antibody multiplex assay
CAB	Community Advisory Board
CDC	US Centers for Disease Control and Prevention
CFR	Code of Federal Regulations
CI	confidence intervals
COVID-19	Coronavirus disease 2019
CoVPN	COVID-19 Prevention Network
CRF	case report form
CRPMC	NIAID Clinical Research Products Management Center
CRS	clinical research site
DAIDS	Division of AIDS (US NIH)
DHHS	US Department of Health and Human Services
EAE	adverse events requiring expedited reporting
EC	Ethics Committee
eDiary	Electronic diary
ELISA	enzyme-linked immunosorbent assay
EUA	emergency use authorization
FAS	Full analysis set
FDA	US Food and Drug Administration
Fred Hutch	Fred Hutchinson Cancer Research Center
GCP	Good Clinical Practice
GMT	geometric mean titer
HIPAA	Health Insurance Portability and Accountability Act
HIV	human immunodeficiency virus
IB	Investigator's Brochure
IBC	Institutional Biosafety Committee
ICH	International Council on Harmonisation of Technical Requirements for Pharmaceuticals for Human Use
IQR	Interquartile range
IDMC	Independent Data Monitoring Committee
IM	Intramuscular
IRB	Institutional Review Board
LC	Laboratory Center

LNP	Lipid nanoparticle
LOC	Leadership and Operations Center
mRNA	Messenger ribonucleic acid
nAb	neutralizing antibody
NHP	nonhuman primate
NIAID	National Institute of Allergy and Infectious Diseases (US NIH)
NIH	US National Institutes of Health
OHRP	US Office for Human Research Protections
PCR	polymerase chain reaction
PI	Principal Investigator
RE	regulatory entity
SAP	Statistical Analysis Plan
SCHARP	Statistical Center for HIV/AIDS Research and Prevention
SDMC	statistical and data management center
SSP	Study specific procedures
SOP	Standard operating procedure
UW-VL	University of Washington Virology Laboratory
UW-VSL	University of Washington Virology Specialty Laboratory
VAERS	Vaccine Adverse Event Reporting System
VE	Vaccine efficacy
VE_I	Vaccine efficacy against infectiousness
VEs	Vaccine efficacy against susceptibility
VL	Viral load

16 Literature cited

1. Johns Hopkins Coronavirus Resource Center. 2020 [Available from: https://coronavirus.jhu.edu/map.html.

2. Lipsitch M, Dean NE. Understanding COVID-19 vaccine efficacy. Science. 2020.

3. Mehrotra DV, Janes HE, Fleming TR, Annunziato PW, Neuzil KM, Carpp LN, et al. Clinical Endpoints for Evaluating Efficacy in COVID-19 Vaccine Trials. Ann Intern Med. 2020.

4. Long QX, Tang XJ, Shi QL, Li Q, Deng HJ, Yuan J, et al. Clinical and immunological assessment of asymptomatic SARS-CoV-2 infections. Nat Med. 2020;26(8):1200-4.

5. Salvatore PP, Sula E, Coyle JP, Caruso E, Smith AR, Levine RS, et al. Recent Increase in COVID-19 Cases Reported Among Adults Aged 18-22 Years - United States, May 31-September 5, 2020. MMWR Morb Mortal Wkly Rep. 2020;69(39):1419-24.

6. Boehmer TK, DeVies J, Caruso E, van Santen KL, Tang S, Black CL, et al. Changing Age Distribution of the COVID-19 Pandemic - United States, May-August 2020. MMWR Morb Mortal Wkly Rep. 2020;69(39):1404-9.

7. CDC: Centers for Disease Control and Prevention. COVID-19 Hospitalization and Death by Age 2020 [Available from: https://www.cdc.gov/coronavirus/2019-ncov/covid-data/investigations-discovery/hospitalization-death-by-age.html.

8. Halloran ME, Longini IM, Jr., Struchiner CJ. Design and interpretation of vaccine field studies. Epidemiol Rev. 1999;21(1):73-88.

9. Swan DA, Goyal A, Bracis C, Moore M, Krantz E, Brown E, et al. Vaccines that prevent SARS-CoV-2 transmission may prevent or dampen a spring wave of COVID-19 cases and deaths in 2021. medRxiv. 2020.

10. Swan DA, Bracis C, Janes H, Moore M, Matrajt L, Reeves DB, et al. COVID-19 vaccines that reduce symptoms but do not block infection need higher coverage and faster rollout to achieve population impact. medRxiv. 2020.

11. Datta S, Halloran ME, Longini IM, Jr. Efficiency of estimating vaccine efficacy for susceptibility and infectiousness: randomization by individual versus household. Biometrics. 1999;55(3):792-8.

12. Milani GP, Dioni L, Favero C, Cantone L, Macchi C, Delbue S, et al. Serological follow-up of SARS-CoV-2 asymptomatic subjects. Sci Rep. 2020;10(1):20048.

13. Piccoli L, Park YJ, Tortorici MA, Czudnochowski N, Walls AC, Beltramello M, et al. Mapping Neutralizing and Immunodominant Sites on the SARS-CoV-2 Spike Receptor-Binding Domain by Structure-Guided High-Resolution Serology. Cell. 2020;183(4):1024-42 e21.

14. Vaccines and Related Biological Products Advisory Committee Meeting. Sponsor Briefing Document, Moderna COVID-19 Vaccine 2020 [Available from: https://www.fda.gov/media/144452/download.

15. Vaccines and Related Biological Products Advisory Committee Meeting. Sponsor Briefing Document Addendum,Moderna COVID-19 Vaccine 2020 [Available from: https://www.fda.gov/media/144453/download.

16. Flood S, King M, Rodgers R, Ruggles S, Warren J. Integrated Public Use Microdata Series, Current Population Survey: version 7.0 [dataset]. 2020.

17. The New York Times. Tracking the Coronavirus at U.S. Colleges and Universities 2020 [Available from: https://www.nytimes.com/interactive/2020/us/covid-college-cases-tracker.html.

18. Business Insider. The 10 biggest coronavirus outbreaks on college campuses across the US since the fall semester began 2020 [Available from: https://www.businessinsider.com/10-colleges-with-fall-coronavirus-outbreaks-1200-cases-or-more-2020-9.

19. Wilson E, Donovan CV, Campbell M, Chai T, Pittman K, Sena AC, et al. Multiple COVID-19 Clusters on a University Campus - North Carolina, August 2020. MMWR Morb Mortal Wkly Rep. 2020;69(39):1416-8.

20. Buitrago-Garcia D, Egli-Gany D, Counotte MJ, Hossmann S, Imeri H, Ipekci AM, et al. Occurrence and transmission potential of asymptomatic and presymptomatic SARS-CoV-2 infections: A living systematic review and meta-analysis. PLoS Med. 2020;17(9):e1003346.

21. Goyal A, Reeves DB, Cardozo-Ojeda EF, Schiffer JT, Mayer BT. Wrong person, place and time: viral load and contact network structure predict SARS-CoV-2 transmission and super-spreading events. medRxiv. 2020.

22. He X, Lau EHY, Wu P, Deng X, Wang J, Hao X, et al. Temporal dynamics in viral shedding and transmissibility of COVID-19. Nat Med. 2020;26(5):672-5.

23. Tindale LC, Stockdale JE, Coombe M, Garlock ES, Lau WYV, Saraswat M, et al. Evidence for transmission of COVID-19 prior to symptom onset. Elife. 2020;9.

24. Kissler SM, Fauver JR, Mack C, Tai C, Shiue KY, Kalinich CC, et al. Viral dynamics of SARS-CoV-2 infection and the predictive value of repeat testing. medRxiv. 2020.

25. CDC: Centers for Disease Control and Prevention. Scientific Brief: Community Use of Cloth Masks to Control the Spread of SARS-CoV-2 2020 [Available from: https://www.cdc.gov/coronavirus/2019-ncov/more/masking-science-sars-cov2.html.

26. Jung CY, Park H, Kim DW, Choi YJ, Kim SW, Chang TI. Clinical Characteristics of Asymptomatic Patients with COVID-19: A Nationwide Cohort Study in South Korea. Int J Infect Dis. 2020;99:266-8.

27. Poteat T, Millett GA, Nelson LE, Beyrer C. Understanding COVID-19 risks and vulnerabilities among black communities in America: the lethal force of syndemics. Ann Epidemiol. 2020;47:1-3.

28. Davies NG, Klepac P, Liu Y, Prem K, Jit M, group CC-w, et al. Age-dependent effects in the transmission and control of COVID-19 epidemics. Nat Med. 2020;26(8):1205-11.

29. Richmond CS, Sabin AP, Jobe DA, Lovrich SD, Kenny PA. SARS-CoV-2 sequencing reveals rapid transmission from college student clusters resulting in morbidity and deaths in vulnerable populations. medRxiv. 2020.

30. Regeneron Pharmaceuticals. Regeneron's REGN-CoV2 antibody cocktail reduced viral levels and improved symptoms in non-hospitalized COVID-19 patients 2020 [Available from: https://newsroom.regeneron.com/news-releases/news-release-details/regenerons-regn-cov2-antibody-cocktail-reduced-viral-levels-and.

31. Chen P, Nirula A, Heller B, Gottlieb RL, Boscia J, Morris J, et al. SARS-CoV-2 Neutralizing Antibody LY-CoV555 in Outpatients with Covid-19. N Engl J Med. 2020.

32. Heneghan C, Brassey J, Jefferson T. SARS-CoV-2 viral load and the severity of COVID-19. The Centre for Evidence-Based Medicine. 2020.

33. Pujadas E, Chaudhry F, McBride R, Richter F, Zhao S, Wajnberg A, et al. SARS-CoV-2 viral load predicts COVID-19 mortality. Lancet Respir Med. 2020;8(9):e70.

34. Westblade LF, Brar G, Pinheiro LC, Paidoussis D, Rajan M, Martin P, et al. SARS-CoV-2 Viral Load Predicts Mortality in Patients with and without Cancer Who Are Hospitalized with COVID-19. Cancer Cell. 2020;38(5):661-71 e2.

35. Letizia AG, Ramos I, Obla A, Goforth C, Weir DL, Ge Y, et al. SARS-CoV-2 Transmission among Marine Recruits during Quarantine. N Engl J Med. 2020.

36. Datta S, Halloran ME, Longini IM, Jr. Augmented HIV vaccine trial design for estimating reduction in infectiousness and protective efficacy. Stat Med. 1998;17(2):185-200.

37. Halloran ME, Struchiner CJ. Causal inference in infectious diseases. Epidemiology. 1995;6(2):142-51.

38. Yang Y, Longini IM, Jr., Halloran ME. Design and Evaluation of Prophylactic Interventions Using Infectious Disease Incidence Data from Close Contact Groups. J R Stat Soc Ser C Appl Stat. 2006;55(3):317-30.

39. Klick B, Leung GM, Cowling BJ. Optimal design of studies of influenza transmission in households. I: case-ascertained studies. Epidemiol Infect. 2012;140(1):106-14.

40. Barnabas RV, Brown ER, Bershteyn A, Stankiewicz Karita HC, Johnston C, Thorpe LE, et al. Hydroxychloroquine as Postexposure Prophylaxis to Prevent Severe Acute Respiratory Syndrome Coronavirus 2 Infection : A Randomized Trial. Ann Intern Med. 2020.

41. Jackson LA, Anderson EJ, Rouphael NG, Roberts PC, Makhene M, Coler RN, et al. An mRNA Vaccine against SARS-CoV-2 - Preliminary Report. N Engl J Med. 2020.

42. Anderson EJ, Rouphael NG, Widge AT, Jackson LA, Roberts PC, Makhene M, et al. Safety and Immunogenicity of SARS-CoV-2 mRNA-1273 Vaccine in Older Adults. N Engl J Med. 2020;383(25):2427-38.

43. Vaccines and Related Biological Products Advisory Committee Meeting. FDA Briefing Document, Moderna COVID-19 Vaccine 2020 [Available from: https://www.fda.gov/media/144434/download.

44. UNAIDS. Ethical considerations in biomedical HIV prevention trials. 2007 7/2007.

45. The National Commission for the Protection of Human Subjects of Biomedical and Behavioral Research. The Belmont Report: Ethical Principles

and Guidelines for the Protection of Human Subjects of Research. 1979 4/18/1979.

46. Council for International Organizations of Medical Sciences (CIOMS). International ethical guidelines for biomedical research involving human subjects. Bull Med Ethics. 2002(182):17-23.

47. Sun K, Wang W, Gao L, Wang Y, Luo K, Ren L, et al. Transmission heterogeneities, kinetics, and controllability of SARS-CoV-2. Science. 2021;371(6526).

48. Li F, Li YY, Liu MJ, Fang LQ, Dean NE, Wong GWK, et al. Household transmission of SARS-CoV-2 and risk factors for susceptibility and infectivity in Wuhan: a retrospective observational study. Lancet Infect Dis. 2021.

49. Madewell ZJ, Yang Y, Longini IM, Jr, Halloran ME, Dean NE. Household Transmission of SARS-CoV-2: A Systematic Review and Meta-analysis. JAMA Network Open. 2020;3(12):e2031756-e.

50. Kissler SM, Fauver JR, Mack C, Olesen SW, Tai C, Shiue KY, et al. SARS-CoV-2 viral dynamics in acute infections. medRxiv. 2020.

51. Grambsch PM, Therneau TM. Proportional Hazards Tests and Diagnostics Based on Weighted Residuals. Biometrika. 1994;81(3):515-26.

52. Hernan MA. The hazards of hazard ratios. Epidemiology. 2010;21(1):13-5.

53. Follmann D, Huang CY. Incorporating founder virus information in vaccine field trials. Biometrics. 2015;71(2):386-96.

54. Lin DY. Proportional means regression for censored medical costs. Biometrics. 2000;56(3):775-8.

Appendix A Sample informed consent form for Main cohort, Immediate Vaccination and Standard of Care Groups

**Sponsor / Study Title:**	**National Institutes of Health, “A randomized controlled study to assess SARS CoV-2 infection, viral shedding, and subsequent potential transmission in individuals immunized with Moderna COVID-19 Vaccine”**
**Protocol Number:**	**CoVPN 3006**
**Principal Investigator: (Study Doctor)**	**«PiFullName»**
**Telephone:**	**«IcfPhoneNumber»**
**Address:**	**«PiLocations»**

Thank you for your interest in our research study. It is called CoVPN 3006. This informed consent form will tell you more about the study. Please read it carefully as you decide if you want to join. If you have questions, please ask us. At the end, we will ask you to answer a few questions to make sure you understand the study.

If you decide to join this study, we will ask you to sign this form. We will offer you a copy of this form to keep.

CoVPN 3006 is a research study. Research is not the same as medical care. The purpose of a research study is to answer scientific questions. We hope that what we learn will help people in the future.

Key information

- Joining this research study is voluntary. It is your choice.
- Our scientific questions are: Does the vaccine protect people from getting infected with a coronavirus called SARS-CoV-2? Does the vaccine prevent people from transmitting SARS-CoV-2 to others?
- If you join, your participation in this study will last for about 5 months.
- If you join, we will ask you to answer questions, get injections, give blood, and take daily swabs of your nose.

  Here are the risks of taking part:

- The most common risk is symptoms such as muscle aches or headaches after getting the injection.
- There is a risk of loss of your personal information.
- There are other, less serious risks. We will tell you more about them later in this consent form.

About the study

The COVID-19 Prevention Network (CoVPN) is doing a study to test a vaccine against SARS-CoV-2. SARS-CoV-2 is the virus that causes the disease called COVID-19. We want to know if the vaccine is able to protect people from getting infected and if getting the vaccine will affect the amount of virus that is in your nose. We also want to know if getting the vaccine will prevent transmitting SARS-CoV-2 to others.

Many young adults will take part in this study around the US. This study has 5 groups. The first 3 groups, called the Immediate Vaccination, Standard of Care, and Vaccine Declined groups, will be in the Main Study.

- The Immediate Vaccination and Standard of Care groups will have about 12,000 people that will get vaccine injections either immediately or 4 months later. In this study, “standard of care” refers to all federal, state, and local recommendations regarding COVID-19 prevention, including COVID-19 vaccination, masking, social distancing, isolation and quarantine.
- The Vaccine Declined group will have about 6,000 people who prefer not to be vaccinated and will be followed for 4 months.
- The last two groups are close contacts of the Main Study: the Prospective Close Contact (PCC) group will have up to 36,000 people, and the Case-ascertained Close Contact (CACC) group will have up to 2250 people. These close contact groups will help researchers determine if the vaccine prevents transmission of the SARS-CoV-2 virus to others. We will explain more about this later in this form.

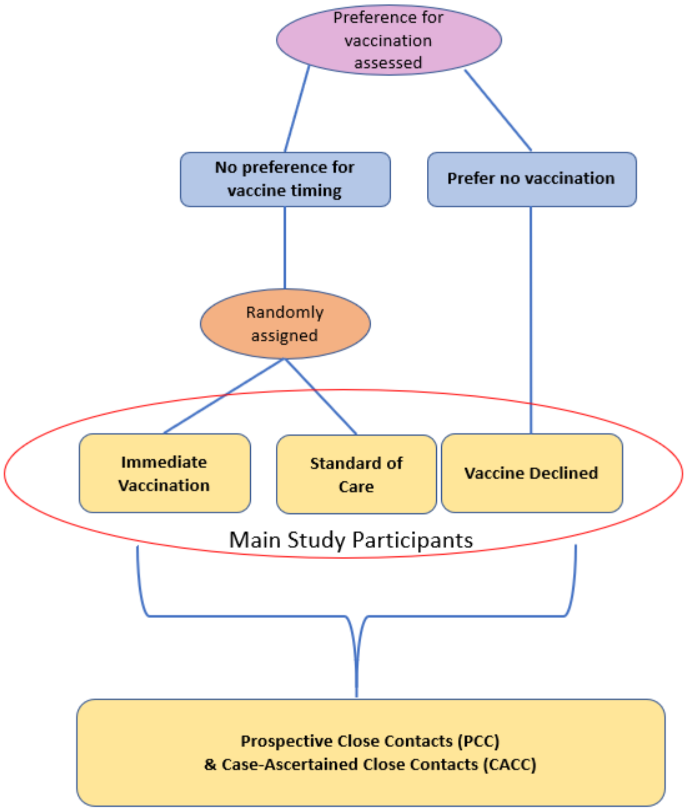

You have indicated that you are agreeable to getting vaccinated for COVID-19 as recommended by the Centers for Disease Control and Prevention (CDC) and have no preference as to the timing of vaccination.

The researcher in charge of this study at this clinic is [Insert name of site PI]. The US National Institutes of Health is paying for this study.

1. **This study's vaccine has an Emergency Use Authorization (EUA) for individuals 18 and older.**

   The vaccine we will use in this study is called the Moderna COVID-19 Vaccine. The vaccine has been given to over 15,000 people in other research studies and over 100 million people under EUA. We will give you the most up-to-date "Fact Sheet for Recipients and Caregivers" with more information about the Moderna COVID-19 Vaccine. The person giving you the vaccine can answer any questions you might have about what is in this fact sheet.

   We already know that the vaccine can prevent serious COVID-19 disease, but we do not know if the vaccine will work to prevent SARS-CoV-2 infection in others. That is what we are testing in this study.

The Moderna COVID-19 Vaccine is not approved for sale in the United States by the US Food and Drug Administration (FDA).

Although the vaccine is not approved, it has an Emergency Use Authorization (EUA) from the FDA based on its success at preventing disease symptoms from SARS-CoV-2 in other studies to date. During a public health emergency, like the COVID-19 pandemic, the FDA can issue an EUA to get vaccines to people faster. When the FDA issues an EUA, they continue to monitor safety.

The vaccine was developed by ModernaTX, Inc. Typical vaccines for viruses are made from a weakened or killed virus, but the Moderna COVID-19 Vaccine is not made from the SARS-CoV-2 virus. The vaccine includes a short segment of messenger ribonucleic acid (mRNA). The mRNA is a genetic code that tells cells how to make a protein. This mRNA is made in a laboratory. When injected into the body, the mRNA vaccine causes some cells to make that viral protein, which can trigger the immune system. Your immune system protects you from disease. If a person is later infected, their immune system remembers the protein from the vaccine which may help it to fight the virus. It is impossible for the vaccine to give you SARS-CoV-2 or COVID-19 infection.

Risks of the Moderna COVID-19 Vaccine:

To date, more than 100 million people have already received the Moderna COVID-19 Vaccine. In clinical trials, most people who got the vaccine had some reaction after their injections, especially after the second injection. The reaction to the vaccine didn’t affect their daily lives and it went away after two or three days. Most of these people said they had pain in the arm where they got the injection. These people also felt tired, had headaches, muscle and joint pain, and chills. A much smaller number of these people said they had redness or swelling where the needle went in their arm. Some people had swelling of the lymph nodes in the same arm of the injection.

The risks of the study vaccine seen so far are the same as what have been seen with most vaccines. Generally, vaccines can cause fever, chills, rash, aches and pains, nausea, headache, dizziness, and feeling tired. Vaccines can also cause pain, redness, swelling, or itching where you got the injection. Most people can still do their planned activities after getting a vaccine. Rarely, people have side effects that limit their normal activities or make them go to the doctor.

Rarely, a vaccine can cause you to have an allergic reaction. You might have a rash, hives, swelling of your face and throat, a fast heartbeat, dizziness and weakness, or trouble breathing. Allergic reactions can be life-threatening. Tell us if you have ever had a bad reaction to any injection or vaccine.

There may be other risks that we don’t yet know about, even serious ones. We will tell you if we learn about any new risks.

Joining the study

2. **You will receive the vaccine immediately or later.**

   Some people will get the study vaccine immediately. Other people will get the study vaccine 4 months later.

   We will compare the results from people who got the vaccine immediately with results from people who got the vaccine later. In this way, we will be able to tell if the vaccine can prevent SARS-CoV-2 infection.

   You have a 50/50 chance of getting the vaccine immediately or later. Whether you get the vaccine immediately or a little later is completely random, like flipping a coin.

   We have no say in whether you get the vaccine immediately or later.

3. **It is completely up to you whether or not to join this study.**

   Take your time in deciding. If it helps, talk to people you trust, such as your doctor, friends, or family. If you decide not to join this study, or if you leave it after you have joined, it will not change your healthcare. If you do join, you do not give up your legal rights.

   You cannot be in this study while you are in another study where you get a study product.

   If you choose not to join this study, you may be able to join another study.

   If you have a preference to be vaccinated immediately, you have the option to not join the study and instead receive vaccination outside of the study right away.

   If you have a preference to not be vaccinated at all, you have the option to join the "Vaccine Declined" observational group.

   If you are an employee or relative of an employee of the study site, you are under no obligation to participate in this study. You may withdraw from the study at any time and for any reason, and your decision will not have any effect on your/your family member's performance appraisal or employment at the study site. You may refuse to participate or you may withdraw from the study at any time without penalty or anyone blaming you.

   If you are a student, your participation will not place you in good favor with the study doctor or other faculty (for example, receiving better grades, recommendations, employment). Also, not participating in this study will not adversely affect your relationship with the study doctor or other faculty.

4. **If you want to join this study, after you sign this consent, you will download an electronic diary (eDiary) app and take questionnaires in it.**

We will send you information about how to download the eDiary app onto your phone or tablet. We can help you download the app and show you how to use it.

To find out if you are eligible to join the study, you will answer questionnaires in the eDiary. Some of the questions will ask about your health history, what medications you take, your age, race, and ethnicity, and where you live. Your answers will tell us about some aspects of your health and if you may be eligible to join the study. It should only take about 10 minutes to complete the questionnaires.

Your answers to the questions may show you are not eligible to join the study, even if you want to. We might contact you to ask some more questions to further evaluate whether you can join the study.

In order to use the app you will be asked to agree to the Terms of Use and Privacy Policy which will appear on your mobile device's screen when you first start using the app. If you decide that you do not want to agree, then you should not participate in the research. While using the app, data about you including personal health information, other communication data, and internet usage will be collected and transmitted to the researchers and to the app developer. A complete description of this data collection and sharing is found in the Privacy Policy. Transmission of information via the internet is not completely secure, so there is a small risk of unintentional release of your information and safeguards are in place to protect your personal information. While the Terms of Use may include statements limiting your rights if you are harmed in this study, you do not release the investigator, sponsor, institution, or agents from responsibility for mistakes, and these statements do not apply to the use of the app in this research study.

Being in the study

If you want to join and you are eligible, here is what will happen:

You will be in the study about 5 months.

Visits can be done in-person or remotely depending on the type of procedures that need to be done. Visits where you get injections must be done in person. Remote visits will be done whenever possible to reduce potential SARS-CoV-2 exposure.

You may have to come to the clinic if you have a lab or health issue.

Site: Insert visit lengths.

Visits can last from [#] to [#] hours.

At your first visit, we will give you nasal swab kits and instructions so that you can take daily swabs of your nose. Once you receive your first kit, and for four months of the study, you will swab your nose every day, and you will be prompted by text or email to scan the barcode on the swab. You can ask us questions if you need help. You will return your swabs using drop-off location(s) specified by the clinic or by mail.

If you are in the immediate vaccination group, we will take blood samples in the clinic at your first injection visit and 2 more times, two months and four months later. When we take blood, the amount will depend on the lab tests we need to do. Each time we take your blood, the total amount we will take will not be more than two tablespoons. If for some reason you cannot come to the clinic, you may be asked to self-collect blood using a small device called the Tasso-SST OnDemand. Each time you collect your own blood, the amount will be less than one teaspoon. We will give you everything you need to take the blood and return it to us. We will be looking at how your body responds to the vaccine and if you been exposed to the SARS-CoV-2 virus.

If you are in the Standard of Care group (the later vaccination group), we will take blood samples in the clinic at the first visit, and 2 more times, two months and four months later. Each time we take your blood, the total amount we will take will not be more than one teaspoon. If for some reason you cannot come to the clinic, you may be asked to self-collect blood using a small device called the Tasso-SST OnDemand. Each time you collect your own blood, the amount will be less than one teaspoon. We will give you everything you need to take the blood and return it to us. We will be looking to see if you been exposed to the SARS-CoV-2 virus.

The self-collection device called the Tasso-SST OnDemand sticks to the skin with a light adhesive. When the button is pressed, a vacuum forms and the device will prick the surface of the skin. The vacuum draws blood out of the capillaries and into a sample pod attached to the bottom of the device.

You will also take questionnaires in the eDiary that ask about the results of any SARS-CoV-2 tests you may have had. The questionnaires will take about 5 minutes to complete. You will also take an End of Study questionnaire in the eDiary. The eDiary will send your phone or tablet a push notification or you will receive a reminder email.

5. **We will give you the vaccine on a schedule.**

You will be in one of 2 groups: The Immediate Vaccination group or the Standard of Care group. In the Standard of Care group, you will be offered vaccine at Month 4 and 5 if you have not received a vaccine already by then. Regardless of which group you are in, you will get 1 injection into your upper arm at 2 separate visits during the study.

We will ask you to sign up for v-safe to report any reactions to the study vaccine. V-safe is a smartphone-based tool from the Center for Disease Control (CDC) that uses text messaging and web surveys to check in with you after your COVID-19 vaccination.

Procedures for Immediate Vaccination group	Screening visit(s)	First injection visit	1st month	2nd month	4th month
Injection		√	√		
Medical history questionnaire	√	√			
SARS CoV-2 testing questionnaire		About 2x per week			
eDiary app	√	About 2x per week through month 4			
Blood drawn		√		√	√
Nasal swab		Daily through month 4			

Procedures for Standard of Care group	Screening visit(s)	1st Day	2nd month	First injection visit (4th month)	5th month
Injection				√	√
Medical history questionnaire	√	√			
SARS CoV-2 testing questionnaire		About 2x per week			
eDiary app	√	About 2x per week through month 4			√
Blood drawn		√	√	√	
Nasal swab		Daily through month 4			

6. **We will compensate you for your participation.**

Site: Insert your compensation plan here, e.g. money for number of swabs returned. Remember to include any costs to participants.

7. **In addition to giving you the vaccine:**

   We will ask you to give a referral code to people with whom you expect to be in frequent close physical proximity during the study, like a roommate or co-worker. We want to ask these people to enroll in another part of this study that will help us find out if the vaccine prevents transmission of the SARS-CoV-2 virus. We may also ask you to provide us with their contact information so that we can reach out to them directly. We are calling this group the Prospective Close Contact group or PCC.

   As part of their study participation, they will be asked to complete questionnaires like the ones you completed in the eDiary. If you become infected with SARS-CoV-2, we will ask you to share your status with your PCCs. Then your PCC participants will be asked to fill out additional questionnaires for 2 weeks as well as take nasal swabs and self-collect blood samples.

   When considering who to ask to be your PCC, you should think about whether you feel comfortable or safe sharing your infection status with them if you get SARS-CoV-2. If you don't feel comfortable sharing this information with them, you should not ask them to participate as a PCC.

   It is possible that someone you know may also be participating in the Main part of the study and might identify you as a close contact. They will not know you are also participating in the study unless you choose to tell them. If someone else identifies you as a close contact, they will give you their referral code. It is okay to be enrolled in both the Main part of the study and the PCC group.

   We may also ask you for the name of someone who can tell us how you are doing if we can't reach you or if you are unable to talk. We may contact you after the main study ends (for example, to tell you about the study results).

   We will ask you to continue to follow any school or employer program for SARS-CoV-2 testing and contact tracing, if applicable. If you test positive for SARS-CoV-2, based on any SARS-CoV-2 testing you receive, please tell us as soon as possible.

8. **The CoVPN will test your samples to see how your body, including your immune system, responds to the Moderna COVID-19 vaccine.**

   We will send your samples to labs approved by the CoVPN. Your samples will not be labeled with your name or other identifying information.

   Researchers may also do genetic testing related to this study on your samples. Your genes are passed to you from your birth parents. Differences in people's genes can help explain why some people get a disease while others do not. The genetic testing will only involve some of your genes, not all of your genes (your

genome). The researchers will study only the genes related to the immune system and coronavirus, and genes that may affect how people get coronavirus.

If you get SARS-CoV-2, the researchers may look at all of the virus' genes that are in your samples. The researchers will use this information to learn more about SARS-CoV-2 and how the virus is impacted by the study vaccine.

The tests the researchers do on your samples are for research purposes, not to check your health. However, the nasal swab testing you do as part of this study may tell us if you are SARS-CoV-2 positive. Nasal swab test results from the study will be provided to the sites and be made available to you. You may be asked to get a confirmatory test. For all other lab tests, we will not provide the results to you or the site.

When your samples are no longer needed for this study, the CoVPN will continue to store them.

9. **We will do our best to protect your private information.**

US sites: Check Health Insurance Portability and Accountability Act (HIPAA) authorization for conflicts with this section.

We will keep your study records and samples in a secure location. We will label all of your samples and most of your records with a code number, not your name or other personal information. We will not share your name with anyone who does not need to know it.

Your records may also be reviewed by groups who watch over this study. These groups include:

- Study monitors
  - The CoVPN, people who work for it, and companies that help it with this study
  - Some government agencies:
    - The US National Institutes of Health
    - The US Office for Human Research Protections
    - Any regulatory agency that reviews research studies
  - Some committees that make sure we protect your rights and keep you safe:
    - The Independent Data Monitoring Committee

- Advarra Institutional Review Board (IRB)
- *Sites may, but are not required to include:* [Insert name of local IRB/EC]
- *Sites include:* [Insert name of local IBC]

All reviewers will take steps to keep your records private.

We cannot guarantee absolute privacy. If you are found to have a medical condition that we are required to report by law, then some of your information may be shared.

At this clinic, we have to report the following information:

Site: Include any public health or legal reporting requirements. Bulleted examples should include all appropriate cases (reportable communicable disease, risk of harm to self or others, etc.)

- SARS-CoV-2
- Pneumonia
- After receiving the vaccine, we are required to report any of the following that may occur to the FDA/CDC Vaccine Adverse Event Reporting System (VAERS):
  - Vaccine administration errors
  - Serious reaction after receiving the vaccine (for example that results in death, hospitalization, a life-threatening reaction, a birth defect, or other important medical event)
  - A condition called "Multisystem Inflammatory Syndrome"
  - Cases of COVID-19 that result in hospitalization

US sites: Include the following boxed text. You can remove the box around the text.

We have a Certificate of Confidentiality from the US government to help protect your privacy. With the certificate, we do not have to release information about you to someone who is not connected to the study, such as the courts or police. Sometimes we can't use the certificate. Since the US National Institutes of Health funds this research, we cannot withhold information from it.

The CoVPN may share information from this study with other researchers. Researchers may publish the results of this study.

Site: The text below may not be deleted or changed per FDA requirement. You can remove the box.

A description of this clinical trial will be available on http://www.ClinicalTrials.gov, as required by U.S. Law. This Web site will not include information that can identify you. At most, the Web site will include a summary of the results. You can search this website at any time.

10. **We may take you out of the study at any time.**

We may take you out of the study if:

- you do not follow instructions,
- we think that staying in the study might harm you,
- the study is stopped for any reason.

11. **If you get COVID-19, we will ask you to do several things.**

If you find out that you have COVID-19, please let us know right away. We will ask you to quarantine per local guidelines. By agreeing to join this study, you also agree to share any SARS-CoV-2 test results with us.

We will ask you to tell anyone with whom you have recently had close physical contact that they may have been exposed to COVID-19. We will give you a referral code to give to these contacts so that they can decide if they would like to join the Case Ascertained Close Contact group (CACC). We may also ask you to provide us with their contact information so that we can reach out to them directly. You may remember at the beginning of this form that we told you that about 2,250 people will be enrolled in the CACC group. The CACC group will help us learn if people who got the study vaccine are less likely to give SARS-CoV-2 to someone else. When considering who to ask to be your CACC, you should think about whether you feel comfortable or safe sharing your infection status with them if you get SARS-CoV-2. If you don't feel comfortable sharing this information with them, you should not ask them to participate as a CACC.

It is possible that someone you know may also be participating in the Main part of the study and might identify you as a close contact. They will not know you are also participating in the study unless you choose to tell them. If someone else identifies you as a close contact, they will give you their referral code. It is okay to be enrolled in both the Main part of the study and the CACC group.

If you find out that you have COVID-19, you will be directed to contact the clinic, and we will ask you to either come to the clinic to have your blood drawn or we will ask you to self-collect less than one teaspoon of blood and complete one additional eDiary questionnaire. It is important that you give the blood sample as soon as possible after finding out the you have COVID-19.

We will ask you to track your COVID-19 symptoms using the eDiary daily for 2 weeks or until the symptoms go away.

After you get better, we will ask you to continue your study participation so that we can monitor your health and safety for the rest of the study.

If you are hospitalized, we will pause your participation. We will ask for your medical records and results from tests you might have received at the hospital. It is unlikely that you will be hospitalized, but to prepare for this, we will ask you to sign and date a medical release of information so we can get records from your doctor or view your hospital records. When you are released from the hospital, please let us know. We will ask you to resume your study participation so that we can continue to monitor your health and safety for the rest of the study.

Site: Modify the following sentence as appropriate. We will not pay for any of your COVID-19 care directly.

12. **If you receive an EUA or licensed COVID-19 vaccine outside the study, we will stop your on-study vaccinations but you may continue in the study.**

If you receive an EUA or licensed COVID-19 vaccine outside the study, please let us know. We will ask you to tell us what vaccine you received and when you received it. You may continue to participate in follow-up visits and procedures like swabbing your nose and completing eDiary entries. Your continued participation is valuable to help us meet our study goals.

If you are in the Standard of Care group and receive COVID-19 vaccination outside the study prior to your Month 4 visit, that Month 4 visit will be your last visit and can happen remotely. At this visit, we will ask you for a final blood sample (the sample can be self-collected using Tasso-SST OnDemand) and to complete an End of Study questionnaire.

Other Risks

13. **There are other risks to being in this study.**

This section describes the other risks and restrictions we know about. There may also be unknown risks, even serious ones. We will tell you if we learn anything new that may affect your willingness to stay in the study.

Risks of routine medical procedures:

In this study, we will do some routine medical procedures. These are taking blood and giving injections. These procedures can cause bruising, pain, fainting, soreness, redness, swelling, itching, a sore, bleeding, and (rarely) muscle damage or infection where you got the injection.

Risks of swabbing your nose:

The feeling of having a small, soft-tipped swab inserted into your nostril and twirled around may be a little uncomfortable, but it should not be painful. There is a small chance there could be some bleeding, but this is unlikely.

Risks to your personal information:

We will take several steps to protect your personal information. Although the risk is very low, it is possible that your personal information could be given to someone who should not have it. If that happened, you could have stress or anxiety.

Risks of genetic testing:

It is unlikely, but the genetic tests done on your samples could show you may be at risk for certain diseases. If others found out, it could lead to discrimination or other problems.

Risk of anxiety/emotional stress:

You may feel anxiety or emotional stress if you experience any of the risks described here. You may feel worried if tests show that you have SARS-CoV-2. Some of the questions we will ask you may make you feel uncomfortable.

Risk of delaying COVID-19 vaccine:

COVID-19 vaccines are highly effective at preventing COVID-19 illness. The Centers for Disease Control and Prevention (CDC) recommend that you receive a COVID-19 vaccine if it is made available to you. By agreeing to potentially delay getting the vaccine you may be at risk for serious illness if you get SARS-CoV-2 infection.

Unknown risks:

The vaccine has been shown to prevent adults from getting COVID-19 for at least 6 months after their injection, but we do not know if it will protect them for any longer. If you get COVID-19, we do not know how the vaccine might affect your illness. It is also unknown if the vaccine will prevent you from giving the virus to someone else. If you get COVID-19, we do not know how the vaccine might affect your illness.

We do not know if getting this vaccine will affect how you respond to any future SARS-CoV-2 vaccines.

We do not know if getting the vaccine will affect pregnancy or breastfeeding. If you have concerns, we recommend that you discuss this with a health care provider.

While you are in this study, you should still follow federal, state, and local guidelines for SARS-CoV-2 prevention, which may include wearing a mask and keeping physical distance from others. If you get sick, you should follow COVID-19 precautions and isolate or self-quarantine.

Benefits

14. **This study may not benefit you.**

COVID-19 vaccination helps protect people from getting sick or severely ill with COVID-19. This study may help researchers find out if the vaccine is able to prevent infection with SARS-CoV-2. COVID-19 vaccination is an important tool to help stop the COVID-19 pandemic. The CDC recommends you get a COVID-19 vaccine as soon as one is available to you.

If the vaccine later becomes approved and sold, there are no plans to share any money with you.

If you decide not to join this study, you could get the Moderna COVID-19 vaccine or another COVID-19 vaccine in your community. You can also continue to follow federal, state, and local guidelines for SARS-CoV-2 prevention. You do not have to join this research study if you don't want to.

Your rights and responsibilities

15. **If you join the study, you have rights and responsibilities.**

You have many rights that we will respect. You also have responsibilities. We list these in the Bill of Rights and Responsibilities for Research. We will give you a copy of it.

Leaving the study

16. **Tell us if you decide to leave the study.**

You are free to leave the study at any time and for any reason. If you want to leave, you will need to tell us. Your care at this clinic, your standing at your school, and your legal rights will not be affected.

Previously collected information about you will remain in the study records and will be included in the analysis of results. Your information can not be removed from the study records.

We may ask you to give a final blood sample or nasal swab.

We believe these steps are important to protecting your health, but it is up to you whether to complete them.

Injuries

Sites: Approval from CoVPN Regulatory Affairs (at CoVPN.core.reg@fredhutch.org) is needed for any change (other than those that the instructions specifically request or those previously approved by CoVPN Regulatory Affairs) to the boxed text. You can remove the box around the text.

17. **If you get sick or injured during the study, contact us immediately.**

Paragraph below for all sites.

Your health is important to us. (*Sites: adjust the following 2 sentences if applicable to the care available at your site)* We will tell you about the care that we can give here. For the care that we cannot provide, we will explain how we will help you get care elsewhere.

2 paragraphs below for all sites.

This research study is covered by a US government program that may provide compensation for a serious injury or death. We will give you a handout with more information about this program. The National Institutes of Health (NIH) does not have a mechanism to provide direct compensation for research related injury.

Some injuries are not physical. For example, you might be harmed emotionally by being in this study. Or you might lose wages because you cannot go to work. However, there are no funds to pay for these kinds of injuries, even if they are study related.

You always have the right to use the court system if you are not satisfied.

18. **When samples are no longer needed for this study, the CoVPN wants to use them in other studies and share them with other researchers.**

The CoVPN calls these samples "extra samples". The CoVPN will only allow your extra samples to be used in other studies if you agree to this. You will mark your decision at the end of this form.

Do I have to agree? No. You are free to say yes or no, or to change your mind after you sign this form. At your request, the CoVPN will destroy all extra samples that it has. Your decision will not affect your being in this study.

Where are the samples stored? Extra samples are stored in a secure central place called a repository. Your samples will be stored in the CoVPN repository in the United States.

How long will the samples be stored? There is no limit on how long your extra samples will be stored. *[Site: Revise the previous sentence to insert limits if your regulatory authority imposes them.]*

Will I be paid for the use of my samples? No. Researchers may make scientific discoveries or products using your samples. If this happens, there is no plan to share any money with you.

Will I benefit from allowing my samples to be used in other studies? Probably not. Results from these other studies are not given to you, this clinic, or your doctor. They may help other people in the future.

Will the CoVPN sell my samples and information? No, but the CoVPN may share your samples with other researchers. Once the CoVPN shares your samples and information, it may not be able to get them back.

What information is shared with researchers? The samples and information will be labeled with a code number which will not be removed. The key to the code will stay at this clinic. However, some information that the CoVPN shares may be personal, such as your race, ethnicity, sex, and health information from the study.

What kind of studies might be done with my extra samples and information? The studies will be related to vaccines, the immune system, coronavirus, and other diseases. Researchers may also do genetic testing on your samples.

If you agree, researchers may compare all of your genes (your genome) to the genomes of many other people. Researchers look for common patterns of genes to help them understand diseases. The researchers may put the information into a protected database so that other researchers can access it. Your name and other personal information will not be included.

Usually, no one could connect your genome to you as a person. There are rules against this. It's also really difficult to do. But there is a risk that someone could combine information from your genome and other public information about you and identify you. If others found out, it could lead to discrimination or other problems. The risk of this happening is very small.

Who will have access to my information in studies using my extra samples?

People who may see your information are:

- Researchers who use your extra samples and information for other research
- Government agencies that fund or monitor the research using your extra samples and information
- The researcher's Institutional Review Board or Ethics Committee

- Any regulatory agency that reviews research studies
- The people who work with the researcher

All of these people will do their best to protect your information. If they publish their research, they will not use your name or identify you personally.

Questions

19. **Whom to contact about this study**

During the study, if you experience any medical problems, suffer a research-related injury, or have questions, concerns, or complaints about the study, please contact the study doctor at the telephone number listed on the first page of this consent document. If you seek emergency care, or hospitalization is required, alert the treating physician that you are participating in this research study.

An institutional review board (IRB) is an independent committee established to help protect the rights of research subjects.

If you have any questions about your rights as a research subject, and/or concerns or complaints regarding this research study, contact:

by mail:
Study Subject Adviser
Advarra IRB
6100 Merriweather Dr., Suite 600Columbia, MD 21044
or call **toll free**: 877-992-4724
or by **email**: adviser@advarra.com

Please reference the following number when contacting the Study Subject Adviser: Pro00049375

US sites may include, but are not required to: You may also contact the [name of local IRB/EC] at [insert contact information].

Your permissions and signature

20. **In Section 18, we told you above about possible other uses of your extra samples** and information outside this study. Please choose only one of the options below and write your initials in the box next to it. Whatever you choose, the CoVPN keeps track of your decision about how your samples and information can be used. You can change your mind after signing this form.

☐ I allow my extra samples and information to be used for other studies related to vaccines, the immune system, coronavirus, and other diseases. This may include genetic testing.

OR

☐ I agree to the option above *and* also to allow my extra samples and information to be used in studies that look at my whole genome.

OR

☐ I do not allow my extra samples to be used in any other studies. This includes not allowing genetic testing, or studies that look at my whole genome.

21. **If you agree to join this study, you will need to sign below. Before you sign, make sure of the following:**

- You have read this consent form.
- You feel that you understand what the study is about and what will happen to you if you join. You understand what the possible risks and benefits are.
- You have had your questions answered and know that you can ask more.
- You agree to join this study.
- You will not be giving up any of your rights by signing this consent form.

Participant's Name (Print)	Participant's Signature	Date
Clinic Staff Conducting Consent Discussion (Print)	Clinic Staff Signature	Date

Appendix B Sample informed consent form for Main cohort, Vaccine Declined Group

**Sponsor / Study Title:**	**National Institutes of Health, "A randomized controlled study to assess SARS CoV-2 infection, viral shedding, and subsequent potential transmission in individuals immunized with Moderna COVID-19 Vaccine"**
**Protocol Number:**	**CoVPN 3006**
**Principal Investigator: (Study Doctor)**	**«PiFullName»**
**Telephone:**	**«IcfPhoneNumber»**
**Address:**	**«PiLocations»**

Thank you for your interest in our research study. It is called CoVPN 3006. This informed consent form will tell you more about the study. Please read it carefully as you decide if you want to join. If you have questions, please ask us. At the end, we will ask you to answer a few questions to make sure you understand the study.

If you decide to join this study, we will ask you to sign this form. We will offer you a copy of this form to keep.

CoVPN 3006 is a research study. Research is not the same as medical care. The purpose of a research study is to answer scientific questions. We hope that what we learn will help people in the future.

Key information

- Joining this research study is voluntary. It is your choice.
- Our scientific questions are: Does the vaccine protect people from getting infected with a coronavirus called SARS-CoV-2? Does the vaccine prevent people from transmitting SARS-CoV-2 to others?
- If you join, your participation in this study will last for about 4-5 months.
- If you join, we will ask you to answer questionnaires, give blood, and take daily swabs of your nose.

  Here are the risks of taking part:

- There is a risk of loss of your personal information.
- There are other, less serious risks. We will tell you more about them later in this consent form.

About the study

The COVID-19 Prevention Network (CoVPN) is doing a study to test a vaccine against SARS-CoV-2. SARS-CoV-2 is the virus that causes the disease called COVID-19. We want to know if the vaccine is able to protect people from getting infected and if getting the vaccine will affect the amount of virus that is in your nose. We also want to know if getting the vaccine will prevent transmitting SARS-CoV-2 to others.

Many young adults will take part in this study around the US. This study has 5 groups. The first 3 groups, called the Immediate Vaccination, Standard of Care, and Vaccine Declined groups, will be in the Main Study.

- The Immediate Vaccination and Standard of Care groups will have about 12,000 people that will get vaccine injections either immediately or 4 months later. In this study, “standard of care” refers to all federal, state, and local recommendations regarding COVID-19 prevention, including COVID-19 vaccination, masking, social distancing, isolation and quarantine.
- The Vaccine Declined group will have about 6,000 people who prefer not to be vaccinated and will be followed for 4 months.
- The last two groups are close contacts of the Main Study: the Prospective Close Contact (PCC) group will have up to 36,000 people, and the Case-ascertained Close Contact (CACC) group will have up to 2250 people. These close contact groups will help researchers determine if the vaccine prevents transmission of the SARS-CoV-2 virus to others. We will explain more about this later in this form.

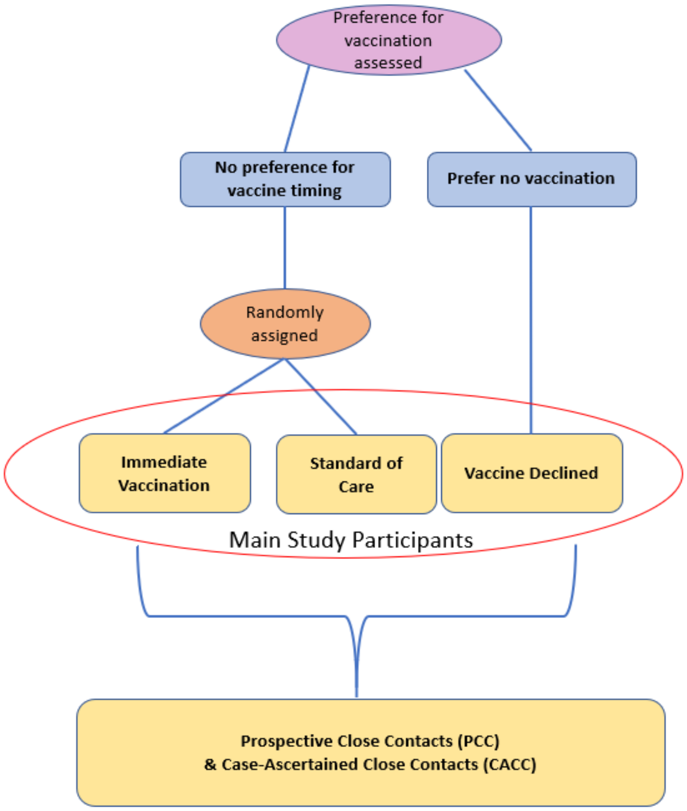

Because you have indicated that you prefer not to receive a COVID-19 vaccine, we are asking you to join the Main part of the study in which people are followed for 4 months but do <u>not</u> receive vaccine injections (Vaccine Declined group). If at the end of the 4 months you indicate that you prefer to be vaccinated, the Moderna COVID-19 vaccine will be offered to you. If you choose to take up the offer of vaccine, you will continue in the study another month to receive both recommended vaccine doses. The researcher in charge of this study at this clinic is [Insert name of site PI]. The US National Institutes of Health is paying for this study.

Joining the study

1. **It is completely up to you whether or not to join this study.**

   Take your time in deciding. If it helps, talk to people you trust, such as your doctor, friends, or family. If you decide not to join this study, or if you leave it after you have joined, it will not change your healthcare. If you do join, you do not give up your legal rights.

You cannot be in this study while you are in another study where you get a study product.

If you choose not to join this study, you may be able to join another study. If at any time you decide you want to get vaccinated, you are free to do so. This may be in this study (beginning at Month 4) or may be elsewhere at any time. You can also get SARS-CoV-2 tested in your community.

If you are an employee or relative of an employee of a participating site, you are under no obligation to participate in this study. You may withdraw from the study at any time and for any reason, and your decision will not have any effect on your/your family member's performance appraisal or employment at the study site. You may refuse to participate or you may withdraw from the study at any time without penalty or anyone blaming you.

If you are a student, your participation will not place you in good favor with the study doctor or other faculty (for example, receiving better grades, recommendations, employment). Also, not participating in this study will not adversely affect your relationship with the study doctor or other faculty.

2. **If you want to join this study, after you sign this consent, you will download an electronic diary (eDiary) app and take questionnaires in it.**

We will send you information about how to download the eDiary app onto your phone or tablet. We can help you download the app and show you how to use it.

To find out if you are eligible to join the study, you will answer questionnaires in the eDiary. Some of the questions will ask about your health history, what medications you take, your age, race, and ethnicity, and where you live. Your answers will tell us about some aspects of your health and if you may be eligible to join the study. It should only take about 10 minutes to complete the questionnaires.

Your answers to the questions may show you are not eligible to join the study, even if you want to. We might contact you to ask some more questions to further evaluate whether you can join the study.

In order to use the app you will be asked to agree to the Terms of Use and Privacy Policy which will appear on your mobile device's screen when you first start using the app. If you decide that you do not want to agree, then you should not participate in the research. While using the app, data about you including personal health information, other communication data, and internet usage will be collected and transmitted to the researchers and to the app developer. A complete description of this data collection and sharing is found in the Privacy Policy. Transmission of information via the internet is not completely secure, so there is a small risk of unintentional release of your information and safeguards are in place to protect your personal information. While the Terms of Use may include

statements limiting your rights if you are harmed in this study, you do not release the investigator, sponsor, institution, or agents from responsibility for mistakes, and these statements do not apply to the use of the app in this research study.

Being in the study

If you want to join and you are eligible, here is what will happen:

3. **You will be in the study about 4-5 months.**

Visits can be done in-person or remotely depending on the type of procedures that need to be done. If you decide to get vaccine from the study site, visits where you get injections must be done in person. Remote visits will be done whenever possible to reduce potential SARS-CoV-2 exposure.

You may have to come to the clinic if you have a lab or health issue.

Site: Insert visit lengths.

Visits can last from [#] to [#] hours.

At your first visit, we will give you nasal swab kits and instructions so that you can take daily swabs of your nose. Once you receive your first kit, and for four months of the study, you will swab your nose every day, and you will be prompted by text or email to scan the barcode on the swab. The questionnaires will take about 5 minutes to complete. You can ask us questions if you need help. You will return your swabs using drop-off location(s) specified by the clinic or by mail.

We will take blood samples in the clinic at the first visit, and 2 more times, two months and four months later. Each time we take your blood, the total amount we will take will not be more than one teaspoon. If for some reason you cannot come to the clinic, you may be asked to self-collect blood using a small device called the Tasso-SST OnDemand. Each time you collect your own blood, the amount will be less than one teaspoon. We will give you everything you need to take the blood and return it to us. We will be looking to see if you been exposed to the SARS-CoV-2 virus. The self-collection device called the Tasso-SST OnDemand sticks to the skin with a light adhesive. When the button is pressed, a vacuum forms and the device will prick the surface of the skin. The vacuum draws blood out of the capillaries and into a sample pod attached to the bottom of the device.

You will also take questionnaires in the eDiary that ask about the results of any SARS-CoV-2 tests you may have had. The questionnaires will take about 5 minutes to complete. You will also take an End of Study questionnaire in the eDiary. The eDiary will send your phone or tablet a push notification or you will receive a reminder email.

**Procedures for Vaccine Declined Group**	**Screening visit(s)**	**1st Day**	**2nd month**	**4th month**
Medical history questionnaire	√	√		
SARS-CoV-2 testing questionnaire		About 2x per week		
eDiary app	√	About 2x per week through month 4		
Blood drawn		√	√	√
Nasal swab		Daily through month 4		

4. **At the end of 4 months the study staff will ask you if want to receive the Moderna COVID-19 vaccine.**

   If you still prefer not to be vaccinated, your study participation will end at Month 4, which will be the 5th visit.

   If you prefer to be vaccinated, you will be consented with another different consent form, and your study participation will last an additional month.

5. **We will compensate you for your participation.**

Site: Insert your compensation plan here, e.g. money for number of swabs returned. Remember to include any costs to participants.

6. **We will ask you to refer your close contacts.**

   We will ask you to give a referral code to people with whom you expect to be in frequent close physical proximity during the study, like a roommate or co-worker. We want to ask these people to enroll in another part of this study that will help us find out if the vaccine prevents transmission of the SARS-CoV-2 virus. We may also ask you to provide us with their contact information so that we can reach out to them directly. We are calling this group the Prospective Close Contact group or PCC.

   As part of their study participation, they will be asked to complete a weekly eDiary that includes questionnaires like the ones you completed. If you become infected with SARS-CoV-2, we will ask you to share your status with your PCCs. Then your PCC participants will be asked to fill out additional questionnaires for 2 weeks as well as take nasal swabs and self-collect blood samples.

   When considering who to ask to be your PCC, you should think about whether you feel comfortable or safe sharing your infection status with them if you get

SARS-CoV-2. If you don't feel comfortable sharing this information with them, you should not ask them to participate as a PCC.

It is possible that someone you know may also be participating in the Main part of the study and might identify you as a close contact. They will not know you are also participating in the study unless you choose to tell them. If someone else identifies you as a close contact, they will give you their referral code. It is okay to be enrolled in both the Main part of the study and the PCC group.

We may also ask you for the name of someone who can tell us how you are doing if we can't reach you or if you are unable to talk. We may contact you after the main study ends (for example, to tell you about the study results).

We will ask you to continue to follow any school or employer program for SARS-CoV-2 testing and contact tracing, if applicable. If you test positive for SARS-CoV-2, based on any SARS-CoV-2 testing you receive, please tell us as soon as possible.

7. **The CoVPN will test your samples to see how your body, including your immune system, responds to a potential infection.**

We will send your samples to labs approved by the CoVPN. Your samples will not be labeled with your name or other identifying information.

Researchers may also do genetic testing related to this study on your samples. Your genes are passed to you from your birth parents. Differences in people's genes can help explain why some people get a disease while others do not. The genetic testing will only involve some of your genes, not all of your genes (your genome). The researchers will study only the genes related to the immune system and coronavirus, and genes that may affect how people get coronavirus.

If you get SARS-CoV-2, the researchers may look at all of the virus' genes that are in your samples. The researchers will use this information to learn more about SARS-CoV-2 and how the virus is impacted by the study vaccine.

The tests the researchers do on your samples are for research purposes, not to check your health. However, the nasal swab testing you do as part of this study may tell us if you are SARS-CoV-2 positive. Nasal swab test results from the study will be provided to the sites and be made available to you. You may be asked to get a confirmatory test. For all other lab tests, we will not provide the results to you or the site.

When your samples are no longer needed for this study, the CoVPN will continue to store them.

8. **We will do our best to protect your private information.**

US sites: Check Health Insurance Portability and Accountability Act (HIPAA) authorization for conflicts with this section.

We will keep your study records and samples in a secure location. We will label all of your samples and most of your records with a code number, not your name or other personal information. We will not share your name with anyone who does not need to know it.

Your records may also be reviewed by groups who watch over this study. These groups include:

- Study monitors
  - The CoVPN, people who work for it, and companies that help it with this study
  - Some government agencies:
    - The US National Institutes of Health
    - The US Office for Human Research Protections
    - Any regulatory agency that reviews research studies
  - Some committees that make sure we protect your rights and keep you safe:
    - The Independent Data Monitoring Committee
    - Advarra Institutional Review Board (IRB)
    - *Sites may, but are not required to include:* [Insert name of local IRB/EC]
    - *Sites include:* [Insert name of local IBC]

All reviewers will take steps to keep your records private.

We cannot guarantee absolute privacy. If you are found to have a medical condition that we are required to report by law, then some of your information may be shared.

At this clinic, we have to report the following information:

Site: Include any public health or legal reporting requirements. Bulleted examples should include all appropriate cases (reportable communicable disease, risk of harm to self or others, etc.)

- SARS-CoV-2
- Pneumonia

US sites: Include the following boxed text. You can remove the box around the text.

We have a Certificate of Confidentiality from the US government to help protect your privacy. With the certificate, we do not have to release information about you to someone who is not connected to the study, such as the courts or police. Sometimes we can't use the certificate. Since the US National Institutes of Health funds this research, we cannot withhold information from it.

The CoVPN may share information from this study with other researchers. Researchers may publish the results of this study.

Site: The text below may not be deleted or changed per FDA requirement. You can remove the box.

A description of this clinical trial will be available on http://www.ClinicalTrials.gov, as required by U.S. Law. This Web site will not include information that can identify you. At most, the Web site will include a summary of the results. You can search this website at any time.

9. **We may take you out of the study at any time.**

We may take you out of the study if:

- you do not follow instructions,
- we think that staying in the study might harm you,
- the study is stopped for any reason.

10. **If you get COVID-19, we will ask you to do several things.**

If you find out that you have COVID-19, please let us know right away. We will ask you to quarantine per local guidelines. By agreeing to join this study, you also agree to share any SARS-CoV-2 test results with us.

We will ask you to tell anyone with whom you have recently had close physical contact that they may have been exposed to COVID-19. We will give you a referral code to give to these contacts so that they can decide if they would like to join the Case Ascertained Close Contact group (CACC). We may also ask you to provide us with their contact information so that we can reach out to them directly. You may remember at the beginning of this form that we told you that about 2250 people will be enrolled in the CACC group. The CACC group will help us learn if people who got the study vaccine are less likely to give SARS-

CoV-2 to someone else. When considering who to ask to be your CACC, you should think about whether you feel comfortable or safe sharing your infection status with them if you get SARS-CoV-2. If you don't feel comfortable sharing this information with them, you should not ask them to participate as a CACC.

It is possible that someone you know may also be participating in the Main part of the study and might identify you as a close contact. They will not know you are also participating in the study unless you choose to tell them. If someone else identifies you as a close contact, they will give you their referral code. It is okay to be enrolled in both the Main part of the study and the CACC group.

If you find out that you have COVID-19, you will be directed to contact the clinic, and we will ask you to either come to the clinic to have your blood drawn or we will ask you to self-collect less than one teaspoon of blood and complete one additional eDiary questionnaire. It is important that you give the blood sample as soon as possible after finding out the you have COVID-19.

We will ask you to track your COVID-19 symptoms using the eDiary daily for 2 weeks or until the symptoms go away.

After you get better, we will ask you to continue your study participation so that we can monitor your health and safety for the rest of the study.

If you are hospitalized, we will pause your participation. We will ask for your medical records and results from tests you might have received at the hospital. It is unlikely that you will be hospitalized, but to prepare for this, we will ask you to sign and date a medical release of information so we can get records from your doctor or view your hospital records. When you are released from the hospital, please let us know. We will ask you to resume your study participation so that we can continue to monitor your health and safety for the rest of the study.

Site: Modify the following sentence as appropriate. We will not pay for any of your COVID-19 care directly.

11. **If you receive an EUA or licensed COVID-19 vaccine outside the study, please tell us.**

If you receive an EUA or licensed COVID-19 vaccine outside the study, please let us know. We will ask you to tell us what vaccine you received and when you received it. You may continue to participate in follow-up visits and procedures like swabbing your nose and completing eDiary entries. Your continued participation is valuable to help us meet our study goals.

If you have received COVID-19 vaccination outside the study prior to your Month 4 visit, that Month 4 visit will be your last visit and can happen remotely. At this visit, we will ask you for a final blood sample (the sample can be self-

collected using Tasso-SST OnDemand) and to complete an End of Study questionnaire.

Other Risks

12. **There are other risks to being in this study.**

This section describes the other risks and restrictions we know about. There may also be unknown risks, even serious ones. We will tell you if we learn anything new that may affect your willingness to stay in the study.

Risks of routine medical procedures:

In this study, we will do some routine medical procedures. These are taking blood and giving injections. These procedures can cause bruising, pain, fainting, soreness, redness, swelling, itching, a sore, bleeding, and (rarely) muscle damage or infection where you got the injection.

Risks of swabbing your nose:

The feeling of having a small, soft-tipped swab inserted into your nostril and twirled around may be a little uncomfortable, but it should not be painful. There is a small chance there could be some bleeding, but this is unlikely.

Risks to your personal information:

We will take several steps to protect your personal information. Although the risk is very low, it is possible that your personal information could be given to someone who should not have it. If that happened, you could have stress or anxiety.

Risks of genetic testing:

It is unlikely, but the genetic tests done on your samples could show you may be at risk for certain diseases. If others found out, it could lead to discrimination or other problems.

Risk of anxiety/emotional stress:

You may feel anxiety or emotional stress if you experience any of the risks described here. You may feel worried if tests show that you have SARS-CoV-2. Some of the questions we will ask you may make you feel uncomfortable.

Risk of declining COVID-19 vaccine:

The Centers for Disease Control and Prevention (CDC) recommends that you receive a COVID-19 vaccine if it is made available to you. COVID-19 vaccines

are highly effective at preventing serious illness. By not getting the vaccine, you may be at risk for serious illness if you get SARS-CoV-2 infection.

While you are in this study, you should still follow federal, state, and local guidelines for SARS-CoV-2 prevention, which may include wearing a mask and keeping physical distance from others. If you get sick, you should follow COVID-19 precautions and isolate or self-quarantine.

Benefits

13. **This study may not benefit you.**

This study may help researchers find out if the vaccine is able to prevent infection with SARS-CoV-2. If the vaccine later becomes approved and sold, there are no plans to share any money with you.

The nasal swab testing you do as part of this study may tell us if you are SARS-CoV-2 positive. Nasal swab test results from the study will be provided to the sites and be made available to you. You can still get nasal swab testing for SARS-CoV-2 at any time outside the study.

Whether or not you join this study, you could get a COVID-19 vaccine in your community. You can also continue to follow local guidelines for SARS-CoV-2 infection. You do not have to join this research study if you don't want to.

Your rights and responsibilities

14. **If you join the study, you have rights and responsibilities.**

You have many rights that we will respect. You also have responsibilities. We list these in the Bill of Rights and Responsibilities for Research. We will give you a copy of it.

Leaving the study

15. **Tell us if you decide to leave the study.**

You are free to leave the study at any time and for any reason. If you want to leave, you will need to tell us. Your care at this clinic, your standing at your school, and your legal rights will not be affected.

Previously collected information about you will remain in the study records and will be included in the analysis of results. Your information can not be removed from the study records.

We may ask you to give a final blood sample or nasal swab.

We believe these steps are important to protecting your health, but it is up to you whether to complete them.

Injuries

Sites: Approval from CoVPN Regulatory Affairs (at CoVPN.core.reg@fredhutch.org) is needed for any change (other than those that the instructions specifically request or those previously approved by CoVPN Regulatory Affairs) to the boxed text. You can remove the box around the text.

16. **If you get sick or injured during the study, contact us immediately.**

Paragraph below for all sites.

Your health is important to us. (*Sites: adjust the following 2 sentences if applicable to the care available at your site)* We will tell you about the care that we can give here. For the care that we cannot provide, we will explain how we will help you get care elsewhere.

The National Institutes of Health (NIH) does not have a mechanism to provide direct compensation for research related injury.

Some injuries are not physical. For example, you might be harmed emotionally by being in this study. Or you might lose wages because you cannot go to work. However, there are no funds to pay for these kinds of injuries, even if they are study related.

You always have the right to use the court system if you are not satisfied.

17. **When samples are no longer needed for this study, the CoVPN wants to use them in other studies and share them with other researchers.**

The CoVPN calls these samples "extra samples". The CoVPN will only allow your extra samples to be used in other studies if you agree to this. You will mark your decision at the end of this form.

Do I have to agree? No. You are free to say yes or no, or to change your mind after you sign this form. At your request, the CoVPN will destroy all extra samples that it has. Your decision will not affect your being in this study.

Where are the samples stored? Extra samples are stored in a secure central place called a repository. Your samples will be stored in the CoVPN repository in the United States.

How long will the samples be stored? There is no limit on how long your extra samples will be stored. *[Site: Revise the previous sentence to insert limits if your regulatory authority imposes them.]*

Will I be paid for the use of my samples? No. Researchers may make scientific discoveries or products using your samples. If this happens, there is no plan to share any money with you.

Will I benefit from allowing my samples to be used in other studies? Probably not. Results from these other studies are not given to you, this clinic, or your doctor. They may help other people in the future.

Will the CoVPN sell my samples and information? No, but the CoVPN may share your samples with other researchers. Once the CoVPN shares your samples and information, it may not be able to get them back.

What information is shared with researchers? The samples and information will be labeled with a code number which will not be removed. The key to the code will stay at this clinic. However, some information that the CoVPN shares may be personal, such as your race, ethnicity, sex, and health information from the study.

What kind of studies might be done with my extra samples and information? The studies will be related to vaccines, the immune system, coronavirus, and other diseases. Researchers may also do genetic testing on your samples.

If you agree, researchers may compare all of your genes (your genome) to the genomes of many other people. Researchers look for common patterns of genes to help them understand diseases. The researchers may put the information into a protected database so that other researchers can access it. Your name and other personal information will not be included.

Usually, no one could connect your genome to you as a person. There are rules against this. It's also really difficult to do. But there is a risk that someone could combine information from your genome and other public information about you and identify you. If others found out, it could lead to discrimination or other problems. The risk of this happening is very small.

Who will have access to my information in studies using my extra samples?

People who may see your information are:

- Researchers who use your extra samples and information for other research
- Government agencies that fund or monitor the research using your extra samples and information
- The researcher's Institutional Review Board or Ethics Committee

- Any regulatory agency that reviews research studies
- The people who work with the researcher

All of these people will do their best to protect your information. If they publish their research, they will not use your name or identify you personally.

Questions

18. **Whom to contact about this study**

During the study, if you experience any medical problems, suffer a research-related injury, or have questions, concerns, or complaints about the study, please contact the study doctor at the telephone number listed on the first page of this consent document. If you seek emergency care, or hospitalization is required, alert the treating physician that you are participating in this research study.

An institutional review board (IRB) is an independent committee established to help protect the rights of research subjects.

If you have any questions about your rights as a research subject, and/or concerns or complaints regarding this research study, contact:
by mail:
Study Subject Adviser
Advarra IRB
6100 Merriweather Dr., Suite 600
Columbia, MD 21044
or call **toll free**: 877-992-4724
or by **email**: adviser@advarra.com

Please reference the following number when contacting the Study Subject Adviser: Pro00049375

US sites may include, but are not required to: You may also contact the [name of local IRB/EC] at [insert contact information].

Your permissions and signature

19. **In Section 18, we told you above about possible other uses of your extra samples** and information outside this study. Please choose only one of the options below and write your initials in the box next to it. Whatever you choose, the CoVPN keeps track of your decision about how your samples and information can be used. You can change your mind after signing this form.

☐ I allow my extra samples and information to be used for other studies related to vaccines, the immune system, coronavirus, and other diseases. This may include genetic testing.

OR

☐ I agree to the option above *and* also to allow my extra samples and information to be used in studies that look at my whole genome.

OR

☐ I do not allow my extra samples to be used in any other studies. This includes not allowing genetic testing, or studies that look at my whole genome.

20. **If you agree to join this study, you will need to sign below. Before you sign, make sure of the following:**

    - You have read this consent form.
    - You feel that you understand what the study is about and what will happen to you if you join. You understand what the possible risks and benefits are.
    - You have had your questions answered and know that you can ask more.
    - You agree to join this study.

    You will not be giving up any of your rights by signing this consent form.

Participant's Name (Print)	Participant's Signature	Date
Clinic Staff Conducting Consent Discussion (Print)	Clinic Staff Signature	Date

Addendum Sample informed consent form for Vaccine Declined Group participants who decide to take up Moderna COVID-19 Vaccine on study

**Sponsor / Study Title:**	**National Institutes of Health, "A randomized controlled study to assess SARS CoV-2 infection, viral shedding, and subsequent potential transmission in individuals immunized with Moderna COVID-19 Vaccine"**
**Protocol Number:**	**CoVPN 3006**
**Principal Investigator: (Study Doctor)**	**«PiFullName»**
**Telephone:**	**«IcfPhoneNumber»**
**Address:**	**«PiLocations»**

Thank you for your interest in getting the Moderna COVID-19 Vaccine through the research study, CoVPN 3006. Previously you agreed to join this study because you did not want to get the vaccine. This informed consent addendum will give you more information about getting the Moderna COVID-19 Vaccine. The information in the informed consent you previously signed still applies. If you have questions, please ask us.

After reviewing this informed consent addendum, if you decide to get the Moderna COVID-19 vaccine in this study, we will ask you to sign this form. We will offer you a copy of this form to keep.

1. **This study's vaccine has an Emergency Use Authorization (EUA) for individuals 18 and older.**

   The vaccine we use in this study is called the Moderna COVID-19 Vaccine. The vaccine has been given to over 15,000 people in other research studies and over 100 million people under EUA. We will give you the most up-to-date "Fact Sheet for Recipients and Caregivers" with more information about the Moderna COVID-19 Vaccine. The person giving you the vaccine can answer any questions you might have about what is in this fact sheet.

   We already know that the vaccine can prevent serious COVID-19 disease, but we do not know if the vaccine will work to prevent SARS-CoV-2 infection in others. That is what we are testing in this study.

   The Moderna COVID-19 Vaccine is not approved for sale in the United States by the US Food and Drug Administration (FDA).

Although the vaccine is not approved, it has an Emergency Use Authorization (EUA) from the FDA based on its success at preventing disease symptoms from SARS-CoV-2 in other studies to date. During a public health emergency, like the COVID-19 pandemic, the FDA can issue an EUA to get vaccines to people faster. When the FDA issues an EUA, they continue to monitor safety.

The vaccine was developed by ModernaTX, Inc. Typical vaccines for viruses are made from a weakened or killed virus, but the Moderna COVID-19 Vaccine is not made from the SARS-CoV-2 virus. The vaccine includes a short segment of messenger ribonucleic acid (mRNA). The mRNA is a genetic code that tells cells how to make a protein. This mRNA is made in a laboratory. When injected into the body, the mRNA vaccine causes some cells to make that viral protein, which can trigger the immune system. Your immune system protects you from disease. If a person is later infected, their immune system remembers the protein from the vaccine which may help it to fight the virus. It is impossible for the vaccine to give you SARS-CoV-2 or COVID-19 infection.

Risks of the Moderna COVID-19 Vaccine:

To date, more than 100 million people have already received the Moderna COVID-19 Vaccine. In clinical trials, most people who got the vaccine had some reaction after their injections, especially after the second injection. The reaction to the vaccine didn't affect their daily lives and it went away after two or three days. Most of these people said they had pain in the arm where they got the injection. These people also felt tired, had headaches, muscle and joint pain, and chills. A much smaller number of these people said they had redness or swelling where the needle went in their arm. Some people had swelling of the lymph nodes in the same arm of the injection.

The risks of the study vaccine seen so far are the same as what have been seen with most vaccines. Generally, vaccines can cause fever, chills, rash, aches and pains, nausea, headache, dizziness, and feeling tired. Vaccines can also cause pain, redness, swelling, or itching where you got the injection. Most people can still do their planned activities after getting a vaccine. Rarely, people have side effects that limit their normal activities or make them go to the doctor.

Rarely, a vaccine can cause you to have an allergic reaction. You might have a rash, hives, swelling of your face and throat, a fast heartbeat, dizziness and weakness, or trouble breathing. Allergic reactions can be life-threatening. Tell us if you have ever had a bad reaction to any injection or vaccine.

There may be other risks that we don't yet know about, even serious ones. We will tell you if we learn about any new risks.

Unknown risks:

The vaccine has been shown to prevent adults from getting COVID-19 for at least 6 months after their injection, but we do not know if it will protect them for any longer. If you get COVID-19, we do not know how the vaccine might affect your illness. It is also unknown if the vaccine will prevent you from giving the virus to someone else. If you get COVID-19, we do not know how the vaccine might affect your illness.

We do not know if getting this vaccine will affect how you respond to any future SARS-CoV-2 vaccines.

We do not know if getting the vaccine will affect pregnancy or breastfeeding. If you have concerns, we recommend that you discuss this with a health care provider.

While you are in this study, you should still follow federal, state, and local guidelines for SARS-CoV-2 prevention, which may include wearing a mask and keeping physical distance from others. If you get sick, you should follow COVID-19 precautions and isolate or self-quarantine.

Agreeing to get the vaccine

2. **You will receive the vaccine on a schedule.**

If you choose, and if you have not received a vaccine by your Month 4 study visit and choose to be vaccinated, you will get the 1st injection of the vaccine at your Month 4 study visit. Study staff will schedule you to get your 2nd injection about 4 weeks later. In total, you will get 1 injection in your upper arm at 2 separate visits during the study.

We will ask you to sign up for v-safe to report any reactions to the study vaccine. V-safe is a smartphone-based tool from the Center for Disease Control (CDC) that uses text messaging and web surveys to check in with you after your COVID-19 vaccination.

3. **We will do our best to protect your private information.**

US sites: Check Health Insurance Portability and Accountability Act (HIPAA) authorization for conflicts with this section.

We will keep your study records and samples in a secure location. We will label all of your samples and most of your records with a code number, not your name or other personal information. We will not share your name with anyone who does not need to know it.

Your records may also be reviewed by groups who watch over this study. These groups include:

- Study monitors
- The CoVPN, people who work for it, and companies that help it with this study
- Some government agencies:
  - The US National Institutes of Health
  - The US Office for Human Research Protections
  - Any regulatory agency that reviews research studies
- Some committees that make sure we protect your rights and keep you safe:
  - The Independent Data Monitoring Committee
  - Advarra Institutional Review Board (IRB)
  - *Sites may, but are not required to include:* [Insert name of local IRB/EC]

All reviewers will take steps to keep your records private.

We cannot guarantee absolute privacy. If you are found to have a medical condition that we are required to report by law, then some of your information may be shared.

At this clinic, we have to report the following information:

Site: Include any public health or legal reporting requirements. Bulleted examples should include all appropriate cases (reportable communicable disease, risk of harm to self or others, etc.)

- SARS-CoV-2
- Pneumonia
- After you receive the vaccine, we are required to report any of the following to the FDA/CDC Vaccine Adverse Event Reporting System (VAERS):
  - Vaccine administration errors
  - Serious reaction after receiving the vaccine (for example that results in death, hospitalization, a life-threatening reaction, a birth defect, or other important medical event)
  - A condition called "Multisystem Inflammatory Syndrome"
  - Cases of COVID-19 that result in hospitalization

US sites: Include the following boxed text. You can remove the box around the text.

We have a Certificate of Confidentiality from the US government to help protect your privacy. With the certificate, we do not have to release information about you to someone who is not connected to the study, such as the courts or police. Sometimes we can't use the certificate. Since the US National Institutes of Health funds this research, we cannot withhold information from it.

The CoVPN may share information from this study with other researchers. Researchers may publish the results of this study.

4. It is completely up to you whether or not to get the vaccine in this study.

Take your time in deciding. If it helps, talk to people you trust, such as your doctor, friends, or family. If you decide not to get vaccine in this study, or if you decide to get it outside of this study, it will not change your healthcare.

If you decide not to get vaccine in this study, you could get the Moderna COVID-19 vaccine or another COVID-19 vaccine in your community. You can also continue to follow local guidelines for SARS-CoV-2 infection. You do not have to get vaccine in this study if you don't want to.

If you are an employee or relative of an employee of the study site, you are under no obligation to get vaccine in this study. You may decline to get vaccine from the study at any time and for any reason, and your decision will not have any effect on your/your family member's performance appraisal or employment at the study site. If you change your mind at any time, there will be no penalty, and no one will get mad at you.

If you are a student, your decision to get vaccine in this study will not place you in good favor with the study doctor or other faculty (for example, receiving better grades, recommendations, employment). Also, not getting vaccine in this study will not adversely affect your relationship with the study doctor or other faculty.

5. We will compensate you for getting the vaccine.

Site: Insert your compensation plan here, e.g. money for number of study visits completed. Remember to include any costs to participants.

Benefits

6. This study may not benefit you.

COVID-19 vaccination helps protect people from getting sick or severely ill with COVID-19. This study may help researchers find out if the vaccine is able to prevent infection with SARS-CoV-2. COVID-19 vaccination is an important tool to help stop the COVID-19 pandemic. The CDC recommends you get a COVID-19 vaccine as soon as one is available to you.

If the vaccine later becomes approved and sold, there are no plans to share any money with you.

Injuries

Sites: Approval from CoVPN Regulatory Affairs (at CoVPN.core.reg@fredhutch.org) is needed for any change (other than those that the instructions specifically request or those previously approved by CoVPN Regulatory Affairs) to the boxed text. You can remove the box around the text.

7. If you get sick or injured during the study, contact us immediately.

Paragraph below for all sites.

Your health is important to us. (*Sites: adjust the following 2 sentences if applicable to the care available at your site)* We will tell you about the care that we can give here. For the care that we cannot provide, we will explain how we will help you get care elsewhere.

2 paragraphs below for all sites.

This research study is covered by a US government program that may provide compensation for a serious injury or death. We will give you a handout with more information about this program. The National Institutes of Health (NIH) does not have a mechanism to provide direct compensation for research related injury.

Some injuries are not physical. For example, you might be harmed emotionally by being in this study. Or you might lose wages because you cannot go to work. However, there are no funds to pay for these kinds of injuries, even if they are study related.

You always have the right to use the court system if you are not satisfied.

Questions

8. Whom to contact about this study

During the study, if you experience any medical problems, suffer a research-related injury, or have questions, concerns, or complaints about the study, please contact the study doctor at the telephone number listed on the first page of this consent document. If you seek emergency care, or hospitalization is required, alert the treating physician that you are participating in this research study.

An institutional review board (IRB) is an independent committee established to help protect the rights of research subjects.

If you have any questions about your rights as a research subject, and/or concerns or complaints regarding this research study, contact:

by mail:

Study Subject Adviser
Advarra IRB
6100 Merriweather Dr., Suite 600Columbia, MD 21044

or call **toll free**: 877-992-4724
or by **email**: adviser@advarra.com

Please reference the following number when contacting the Study Subject Adviser: Pro00049375

US sites may include, but are not required to: You may also contact the [name of local IRB/EC] at [insert contact information].

Your permissions and signature

9. If you agree to join this study, you will need to sign below. Before you sign, make sure of the following:

- You have read this consent form.
- You feel that you understand what the study is about and what will happen to you if you join. You understand what the possible risks and benefits are.
- You have had your questions answered and know that you can ask more.
- You agree to join this study.
- You will not be giving up any of your rights by signing this consent form.

Participant's Name (Print)	Participant's Signature	Date
Clinic Staff Conducting Consent Discussion (Print)	Clinic Staff Signature	Date

Appendix C Sample informed consent form for Prospective Close Contact (PCC) cohort

**Sponsor / Study Title:**	**National Institutes of Health, "A randomized controlled study to assess SARS CoV-2 infection, viral shedding, and subsequent potential transmission in individuals immunized with Moderna COVID-19 Vaccine"**
**Protocol Number:**	**CoVPN 3006**
**Principal Investigator: (Study Doctor)**	**«PiFullName»**
**Telephone:**	**«IcfPhoneNumber»**
**Address:**	**«PiLocations»**

Thank you for your interest in our research study. It is called CoVPN 3006. This informed consent form will tell you more about the study. Please read it carefully to help you decide if you want to join. If you have questions, please ask us.

If you decide to join this study, we will ask you to sign this form. We will offer you a copy of this form to keep.

CoVPN 3006 is a research study. Research is not the same as medical care. The purpose of a research study is to answer scientific questions. We hope that what we learn will help people in the future.

Key information

- Joining this research study is voluntary. It is your choice.
- Our scientific questions are: Does the study vaccine protect people from getting infected with a coronavirus called SARS-CoV-2? Does the study vaccine prevent people from transmitting SARS-CoV-2 to others?
- If you join, your participation in this study will last for about 5 months.
- If you join, we will ask you to answer eDiary questionnaires. If you or your contact in the main study group becomes SARS-CoV-2 infected, then we will ask you to take daily swabs of your nose, answer daily eDiary questionnaire for 2 weeks, and give 2 blood samples.

  Here are the risks of taking part:

- There is a risk of loss of your personal information.

- There are other, less serious risks. We will tell you more about them later in this consent form.

About the study

The COVID-19 Prevention Network (CoVPN) is doing a study to test a vaccine against SARS-CoV-2. SARS-CoV-2 is the virus that causes the disease called COVID-19. We want to know if the vaccine is able to protect people from getting infected and if getting the vaccine will affect the amount of virus that is in your nose. We also want to know if getting the vaccine will prevent transmitting SARS-CoV-2 to others.

We are asking you to join this study because you have been referred as a close contact with someone who is in the Main study.

Many young adults will take part in this study around the US. This study has 5 groups. The first 3 groups, called the Immediate Vaccination, Standard of Care, and Vaccine Declined groups, will be in the Main Study.

- The Immediate Vaccination and Standard of Care groups will have about 12,000 people that will get vaccine injections either immediately or 4 months later. In this study, "standard of care" refers to all federal, state, and local recommendations regarding COVID-19 prevention, including COVID-19 vaccination, masking, social distancing, isolation and quarantine.

- The Vaccine Declined group will have about 6,000 people who prefer not to be vaccinated and will be followed for 4 months.

- The last two groups are close contacts of the Main Study: the Prospective Close Contact (PCC) group will have up to 36,000 people, and the Case-ascertained Close Contact (CACC) group will have up to 2250 people. These close contact groups will help researchers determine if the vaccine prevents transmission of the SARS-CoV-2 virus to others. We will explain more about this later in this form.

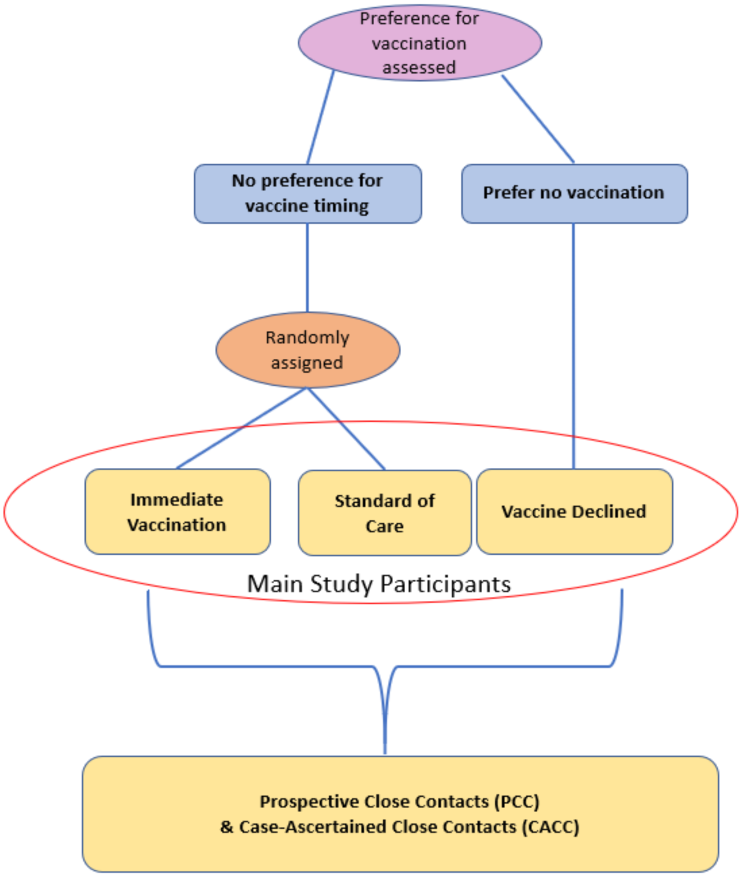

You are being asked to participate in the PCC group. The researcher in charge of this study at this clinic is [Insert name of site PI]. The US National Institutes of Health is paying for this study.

Joining the study

1. **It is completely up to you whether or not to join this study.**

   Take your time in deciding. If it helps, talk to people you trust, such as your friends or family. If you decide not to join this study, or if you leave it after you have joined, it will not change your healthcare. If you do join, you do not give up your legal rights.

   If you are an employee or relative of an employee of the study site, you are under no obligation to participate in this study. You may withdraw from the study at any time and for any reason, and your decision will not have any effect on your/your family member's performance appraisal or employment at the study site. You may refuse to participate or you may withdraw from the study at any time without penalty or anyone blaming you.

If you are a student, your participation will not place you in good favor with the study doctor or other faculty (for example, receiving better grades, recommendations, employment). Also, not participating in this study will not adversely affect your relationship with the study doctor or other faculty.

Being in the study

If you want to join, here is what will happen:

We will send you information about how to download an electronic diary (eDiary) app onto your phone or tablet. We can help you download the app and show you how to use it. In order to use the app you will be asked to agree to the Terms of Use and Privacy Policy which will appear on your mobile device's screen when you first start using the app. If you decide that you do not want to agree, then you should not participate in the research. While using the app, data about you including personal health information, other communication data, and internet usage will be collected and transmitted to the researchers and to the app developer. A complete description of this data collection and sharing is found in the Privacy Policy. Transmission of information via the internet is not completely secure, so there is a small risk of unintentional release of your information and safeguards are in place to protect your personal information. While the Terms of Use may include statements limiting your rights if you are harmed in this study, you do not release the investigator, sponsor, institution, or agents from responsibility for mistakes, and these statements do not apply to the use of the app in this research study.

Initially, you will answer a 5-minute questionnaire in the eDiary. It will ask you about things such as your sex, age, race, and ethnicity. During the study, you will also answer questionnaires about whether you are sick or if you have tested positive for SARS-CoV-2. You will take an End of Study questionnaire in the eDiary at your last study visit. If you are a student, you may get SARS-CoV-2 testing at your school about twice a week. If you have tested positive, you will enter the results of your SARS-CoV-2 tests taken at school or another location into the eDiary.

If you or your contact in the main study group becomes SARS-CoV-2 infected, then we will ask you to take swabs of your nose and answer eDiary questionnaires every day for 2 weeks. You will be prompted by text or email to scan the barcode on the swab. You will return your nasal swabs using drop-off location(s) specified by the clinic or by mail.

We will ask you to self-collect a small amount of your blood twice using a small device called the Tasso-SST OnDemand. You will take the first blood sample as soon as possible once you begin the daily swabs, and then you will take another sample about a month later. This will be less than one teaspoon of blood taken from one of your upper arms using a small device. We will give you everything you need to take the blood and return it to us.

The self-collection device sticks to the skin with a light adhesive. When the button is pressed, a vacuum forms, and the device will prick the surface of the skin. The vacuum draws blood out of the capillaries and into a sample pod attached to the bottom of the device.

It is important that you continue to follow the federal, state, and local guidelines for SARS-CoV-2 prevention, which may include wearing a mask and keeping physical distance from others. If you get COVID-19 and are hospitalized, we will pause your participation. We will ask for your medical records and results from tests you might have received at the hospital. It is unlikely that you will be hospitalized, but to prepare for this, we will ask you to sign and date a medical release of information so we can get records from your doctor or view your hospital records. When you are released from the hospital, please let us know. We will ask you to resume your study participation so that we can continue to monitor your health and safety for the rest of the study.

We will not pay for any of your COVID-19 care directly.

2. **The CoVPN will test your samples to see how your body, including your immune system, responds to the potential infection.**

   We will send your samples to labs approved by the CoVPN. Your samples will not be labeled with your name or other identifying information.

   Researchers may also do genetic testing related to this study on your samples. Your genes are passed to you from your birth parents. Differences in people's genes can help explain why some people get a disease while others do not. The genetic testing will only involve some of your genes, not all of your genes (your genome). The researchers will study only the genes related to the immune system and coronavirus, and genes that may affect how people get coronavirus.

   If you get SARS-CoV-2, the researchers may look at all of the virus' genes that are in your samples. The researchers will use this information to learn more about SARS-CoV-2 and how the virus is impacted by the study vaccine.

   The tests the researchers do on your samples are for research purposes, not to check your health. However, the nasal swab testing you may do as part of this study may tell us if you are SARS-CoV-2 positive. Nasal swab test results from the study will be provided to the sites and be made available to you. You may be asked to get a confirmatory test. For all other lab tests, we will not provide the results to you or the site.

   When your samples are no longer needed for this study, the CoVPN will continue to store them.

3. **When samples are no longer needed for this study, the CoVPN wants to use them in other studies and share them with other researchers.**

The CoVPN calls these samples "extra samples". The CoVPN will only allow your extra samples to be used in other studies if you agree to this. You will mark your decision at the end of this form.

Do I have to agree? No. You are free to say yes or no, or to change your mind after you sign this form. At your request, the CoVPN will destroy all extra samples that it has. Your decision will not affect your being in this study.

Where are the samples stored? Extra samples are stored in a secure central place called a repository. Your samples will be stored in the CoVPN repository in the United States.

How long will the samples be stored? There is no limit on how long your extra samples will be stored. *[Site: Revise the previous sentence to insert limits if your regulatory authority imposes them.]*

Will I be paid for the use of my samples? No. Researchers may make scientific discoveries or products using your samples. If this happens, there is no plan to share any money with you.

Will I benefit from allowing my samples to be used in other studies? Probably not. Results from these other studies are not given to you, this clinic, or your doctor. They may help other people in the future.

Will the CoVPN sell my samples and information? No, but the CoVPN may share your samples with other researchers. Once the CoVPN shares your samples and information, it may not be able to get them back.

What information is shared with researchers? The samples and information will be labeled with a code number which will not be removed. The key to the code will stay at this clinic. However, some information that the CoVPN shares may be personal, such as your race, ethnicity, sex, and health information from the study.

What kind of studies might be done with my extra samples and information? The studies will be related to vaccines, the immune system, coronavirus, and other diseases.

Researchers may also do genetic testing on your samples.

If you agree, researchers may compare all of your genes (your genome) to the genomes of many other people. Researchers look for common patterns of genes to help them understand diseases. The researchers may put the information into a protected database so that other researchers can access it. Your name and other personal information will not be included.

Usually, no one could connect your genome to you as a person. There are rules against this. It's also really difficult to do. But there is a risk that someone could combine information from your genome and other public information about you and identify you. If others found out, it could lead to discrimination or other problems. The risk of this happening is very small.

Who will have access to my information in studies using my extra samples?

People who may see your information are:

- Researchers who use your extra samples and information for other research
- Government agencies that fund or monitor the research using your extra samples and information
- The researcher's Institutional Review Board or Ethics Committee
- Any regulatory agency that reviews research studies
- The people who work with the researcher

All of these people will do their best to protect your information. If they publish their research, they will not use your name or identify you personally.

4. **We will compensate you for your participation.**

Site: Insert your compensation plan here, e.g. money for number of swabs returned. Remember to include any costs to participants.

5. **We will do our best to protect your private information.**

Site: Check HIPAA authorization for conflicts with this section.

Your study records and samples will be kept in a secure location. We will label all of your samples and most of your records with a code number, not your name or other personal information. We will not share your name with anyone who does not need to know it.

Your records may also be reviewed by groups who watch over this study. These groups include:

- Study monitors
- The CoVPN, people who work for it, and companies that help it with this study
- Some government agencies:

  - The US National Institutes of Health
  - The US Office for Human Research Protections
  - Any regulatory agency that reviews research studies
- Some committees that make sure we protect your rights and keep you safe:
  - The Independent Data Monitoring Committee
  - Advarra Institutional Review Board (IRB)
  - *Sites may but are not required to include:* [Insert name of local IRB/EC]
  - *Sites include:* [Insert name of local IBC]

All reviewers will take steps to keep your records private.

Sites: Include the following boxed text. You can remove the box around the text.

We have a Certificate of Confidentiality from the US government to help protect your privacy. With the certificate, we do not have to release information about you to someone who is not connected to the study, such as the courts or police. Sometimes we can’t use the certificate. Since the US National Institutes of Health funds this research, we cannot withhold information from it.

The CoVPN may share information from this study with other researchers. Researchers may publish the results of this study.

Sites: The text below may not be deleted or changed per FDA requirement. You can remove the box.

A description of this clinical trial will be available on http://www.ClinicalTrials.gov, as required by U.S. Law. This Web site will not include information that can identify you. At most, the Web site will include a summary of the results. You can search this website at any time.

6. **We may take you out of the study at any time.**

We may take you out of the study if:

- you do not follow instructions,
- we think that staying in the study might harm you,
- the study is stopped for any reason.

Risks

7. **There are some risks to being in this study.**

   This section describes the risks we know about. There may also be unknown risks, even serious ones. We will tell you if we learn anything new that may affect your willingness to stay in the study.

   Risks of blood draws:

   In this study, we will either take your blood or ask you to self-collect your blood with a small device. Taking blood can cause bruising, pain, fainting, soreness, redness, and itching.

   Risks of swabbing your nose:

   The feeling of having a small, soft-tipped swab inserted into your nostril and twirled around may be a little uncomfortable, but it should not be painful. There is a small chance there could be some bleeding, but this is unlikely.

   Risks to your personal information:

   We will take several steps to protect your personal information. Although the risk is very low, it is possible that your personal information could be given to someone who should not have it. If that happened, you could have stress or anxiety.

   Risks of COVID-19:

   While you are in this study, you should still follow federal, state, and local guidelines for SARS-CoV-2 prevention, which may include wearing a mask and keeping physical distance from others. If you get sick, you should follow COVID-19 precautions and isolate or self-quarantine.

Benefits

8. **This study may not benefit you**.

   This study may help researchers find out if people who got the vaccine are less likely to give it to people who they have been in close contact with. If the vaccine later becomes approved and sold, there are no plans to share any money with you.

   You do not have to join this research study if you don’t want to.

Leaving the study

9. **Tell us if you decide to leave the study.**

   You are free to leave the study at any time and for any reason. If you want to leave, you will need to tell us. Your legal rights will not be affected.

   Previously collected information about you will remain in the study records and will be included in the analysis of results. Your information can not be removed from the study records.

   We may ask you to give a final blood sample or nasal swab.

   We believe these steps are important to protecting your health, but it is up to you whether to complete them.

Injuries

Sites: Approval from CoVPN Regulatory Affairs (at CoVPN.core.reg@fredhutch.org) is needed for any change (other than those that the instructions specifically request or those previously approved by CoVPN Regulatory Affairs) to the boxed text. You can remove the box around the text.

10. **If you have a life-threatening medical emergency, call 911 right away or seek help immediately.**

We do not expect you to get sick or injured as a result of being in this study. If you get COVID-19 while you are in the study, please let us know. *(Sites: adjust the following 2 sentences if applicable to the care available at your site)* We will tell you about the care that we can give here. For the care that we cannot provide, we will explain how we will help you get care elsewhere. You or your health insurance will have to pay for the care you get elsewhere.

Some injuries are not physical. For example, you might be harmed emotionally by being in this study. Or you might lose wages because you cannot go to work. However, there are no funds to pay for these kinds of injuries, even if they are study related.

You always have the right to use the court system if you are not satisfied.

Questions

11. **Whom to contact about this study**

    During the study, if you experience any medical problems, suffer a research-related injury, or have questions, concerns, or complaints about the study, please

contact the study doctor at the telephone number listed on the first page of this consent document.

An institutional review board (IRB) is an independent committee established to help protect the rights of research subjects.

If you have any questions about your rights as a research subject, and/or concerns or complaints regarding this research study, contact:
by mail:

Study Subject Adviser
Advarra IRB
6100 Merriweather Dr., Suite 600
Columbia, MD 21044

or call **toll free**: 877-992-4724
or by **email**: adviser@advarra.com

Please reference the following number when contacting the Study Subject Adviser: Pro00049375

US sites may include, but are not required to: You may also contact the [name of local IRB/EC] at [insert contact information].

Your permissions and signature

12. **In Section 3, we told you above about possible other uses of your extra samples** and information outside this study. Please choose only one of the options below and write your initials in the box next to it. Whatever you choose, the CoVPN keeps track of your decision about how your samples and information can be used. You can change your mind after signing this form.

☐ I allow my extra samples and information to be used for other studies related to vaccines, the immune system, coronavirus, and other diseases. This may include genetic testing.

OR

☐ I agree to the option above *and* also to allow my extra samples and information to be used in studies that look at my whole genome.

OR

☐ I do not allow my extra samples to be used in any other studies. This includes not allowing genetic testing, or studies that look at my whole genome.

13. If you agree to join this study, you will need to sign below. Before you sign, make sure of the following:

    - You have read this consent form.
    - You feel that you understand what the study is about and what will happen to you if you join. You understand what the possible risks and benefits are.
    - You agree to join this study.

    You will not be giving up any of your rights by signing this consent form.

Participant's Name (Print)	Participant's Signature	Date
Clinic Staff Conducting Consent Discussion (Print)	Clinic Staff Signature	Date

Appendix D Sample informed consent form for Case-Ascertained Close Contact (CACC) cohort

**Sponsor / Study Title:**	**National Institutes of Health, "A randomized controlled study to assess SARS CoV-2 infection, viral shedding, and subsequent potential transmission in individuals immunized with Moderna COVID-19 Vaccine"**
**Protocol Number:**	**CoVPN 3006**
**Principal Investigator: (Study Doctor)**	**«PiFullName»**
**Telephone:**	**«IcfPhoneNumber»**
**Address:**	**«PiLocations»**

Thank you for your interest in our research study. It is called CoVPN 3006. This informed consent form will tell you more about the study. Please read it carefully to help you decide if you want to join. If you have questions, please ask us.

If you decide to join this study, we will ask you to sign this form. We will offer you a copy of this form to keep.

CoVPN 3006 is a research study. Research is not the same as medical care. The purpose of a research study is to answer scientific questions. We hope that what we learn will help people in the future.

Key information

- Joining this research study is voluntary. It is your choice.
- Our scientific questions are: Does the study vaccine protect people from getting infected with a coronavirus called SARS-CoV-2? Does the study vaccine prevent people from transmitting SARS-CoV-2 to others?
- If you join, your participation in this study will last for about 1 month.
- If you join, we will ask you to answer questionnaires, give blood, and take daily swabs of your nose for 2 weeks.

  Here are the risks of taking part:

- There is a risk of loss of your personal information.

- There are other, less serious risks. We will tell you more about them later in this consent form.

About the study

The COVID-19 Prevention Network (CoVPN) are doing a study to test a vaccine against SARS-CoV-2. SARS-CoV-2 is the virus that causes the disease called COVID-19. We are asking you to join this study because you recently had close physical contact with someone who is in the Main study and who got infected with SARS-CoV-2. We want to find out if the person you had close physical contact might have transmitted SARS-CoV-2 to you.

Many young adults will take part in this study around the US. This study has 5 groups. The first 3 groups, called the Immediate Vaccination, Standard of Care, and Vaccine Declined groups, will be in the Main Study.

- The Immediate Vaccination and Standard of Care groups will have about 12,000 people that will get vaccine injections either immediately or 4 months later. In this study, "standard of care" refers to all federal, state, and local recommendations regarding COVID-19 prevention, including COVID-19 vaccination, masking, social distancing, isolation and quarantine.

- The Vaccine Declined group will have about 6,000 people who prefer not to be vaccinated and will be followed for 4 months.

- The last two groups are close contacts of the Main Study: the Prospective Close Contact (PCC) group will have up to 36,000 people, and the Case-ascertained Close Contact (CACC) group will have up to 2250 people. These close contact groups will help researchers determine if the vaccine prevents transmission of the SARS-CoV-2 virus to others. We will explain more about this later in this form.

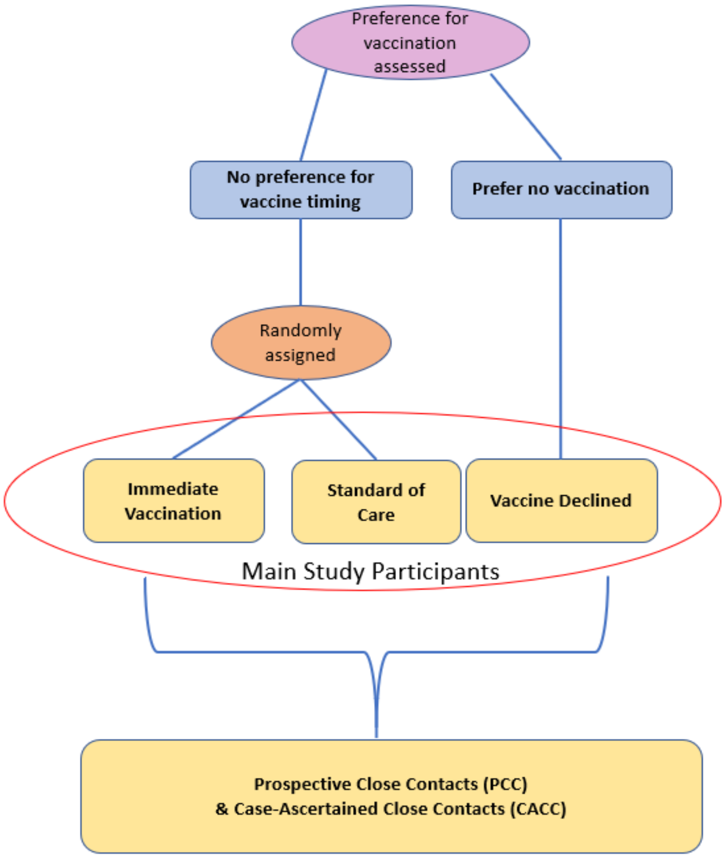

You are being asked to participate in the CACC group. The researcher in charge of this study at this clinic is [Insert name of site PI]. The US National Institutes of Health is paying for this study.

Joining the study

1. **It is completely up to you whether or not to join this study.**

   Take your time in deciding. If it helps, talk to people you trust, such as your friends or family. If you decide not to join this study, or if you leave it after you have joined, it will not change your healthcare. If you do join, you do not give up your legal rights.

   If you are an employee or relative of an employee of the study site, you are under no obligation to participate in this study. You may withdraw from the study at any time and for any reason, and your decision will not have any effect on your/your family member's performance appraisal or employment at the study site. You may refuse to participate or you may withdraw from the study at any time without penalty or anyone blaming you.

   If you are a student, your participation will not place you in good favor with the study doctor or other faculty (for example, receiving better grades,

recommendations, employment). Also, not participating in this study will not adversely affect your relationship with the study doctor or other faculty.

Being in the study

If you want to join, here is what will happen:

We will send you information about how to download an electronic diary (eDiary) app onto your phone or tablet. We can help you download the app and show you how to use it. In order to use the app you will be asked to agree to the Terms of Use and Privacy Policy which will appear on your mobile device's screen when you first start using the app. If you decide that you do not want to agree, then you should not participate in the research. While using the app, data about you including personal health information, other communication data, and internet usage will be collected and transmitted to the researchers and to the app developer. A complete description of this data collection and sharing is found in the Privacy Policy. Transmission of information via the internet is not completely secure, so there is a small risk of unintentional release of your information and safeguards are in place to protect your personal information. While the Terms of Use may include statements limiting your rights if you are harmed in this study, you do not release the investigator, sponsor, institution, or agents from responsibility for mistakes, and these statements do not apply to the use of the app in this research study.

You will take a 5-minute questionnaire in the eDiary. It will ask you about your contact with the person who tested positive for SARS-CoV-2, as well as other things such as your sex, age, race, and ethnicity. You will also take an End of Study questionnaire in the eDiary. If you have tested positive for SARS-CoV-2, you will enter the results of your SARS-CoV-2 tests taken at school or another location into the eDiary.

If you are affiliated with a school that provides SARS-CoV-2 testing, you may get tested about twice a week.

We will ask you to take daily swabs of both of your nostrils and track your COVID-19 symptoms in the eDiary daily for two weeks. You will be prompted by text or email to scan the barcode on the swab. You will return your nasal swabs using drop-off location(s) specified by the clinic or by mail.

We will also ask you to collect a small amount of blood from one of your upper arms twice during the study using a small device called the Tasso-SST OnDemand. This will be less than one teaspoon of blood taken from one of your upper arms using a small device. You will take the first blood sample as soon as possible, and then you will take another sample about a month later. We will give you everything you need to take these samples and return them to us.

The self-collection device sticks to the skin with a light adhesive. When the button is pressed, a vacuum forms, and the device will prick the surface of the skin. The vacuum draws blood out of the capillaries and into a sample pod attached to the bottom of the device.

It is important that you continue to follow the federal, state, and local guidelines for SARS-CoV-2 prevention, which may include wearing a mask and keeping physical distance from others.

If you are hospitalized, we will pause your participation. We will ask for your medical records and results from tests you might have received at the hospital. It is unlikely that you will be hospitalized, but to prepare for this, we will ask you to sign and date a medical release of information so we can get records from your doctor or view your hospital records. When you are released from the hospital, please let us know. We will ask you to resume your study participation so that we can continue to monitor your health and safety for the rest of the study.

2. **The CoVPN will test your samples to see how your body, including your immune system, responds to the potential infection.**

We will send your samples to labs approved by the CoVPN. Your samples will not be labeled with your name or other identifying information.

Researchers may also do genetic testing related to this study on your samples. Your genes are passed to you from your birth parents. Differences in people's genes can help explain why some people get a disease while others do not. The genetic testing will only involve some of your genes, not all of your genes (your genome). The researchers will study only the genes related to the immune system and coronavirus, and genes that may affect how people get coronavirus.

If you get SARS-CoV-2, the researchers may look at all of the virus' genes that are in your samples. The researchers will use this information to learn more about SARS-CoV-2 and how the virus is impacted by the study vaccine.

The tests the researchers do on your samples are for research purposes, not to check your health. However, the nasal swab testing you do as part of this study may tell us if you are SARS-CoV-2 positive. Nasal swab test results from the study will be provided to the sites and be made available to you. You may be asked to get a confirmatory test. For all other lab tests, we will not provide the results to you or the site.

When your samples are no longer needed for this study, the CoVPN will continue to store them.

3. **When samples are no longer needed for this study, the CoVPN wants to use them in other studies and share them with other researchers.**

   The CoVPN calls these samples “extra samples”. The CoVPN will only allow your extra samples to be used in other studies if you agree to this. You will mark your decision at the end of this form.

   Do I have to agree? No. You are free to say yes or no, or to change your mind after you sign this form. At your request, the CoVPN will destroy all extra samples that it has. Your decision will not affect your being in this study.

   Where are the samples stored? Extra samples are stored in a secure central place called a repository. Your samples will be stored in the CoVPN repository in the United States.

   How long will the samples be stored? There is no limit on how long your extra samples will be stored. *[Site: Revise the previous sentence to insert limits if your regulatory authority imposes them.]*

   Will I be paid for the use of my samples? No. Researchers may make scientific discoveries or products using your samples. If this happens, there is no plan to share any money with you.

   Will I benefit from allowing my samples to be used in other studies? Probably not. Results from these other studies are not given to you, this clinic, or your doctor. They may help other people in the future.

   Will the CoVPN sell my samples and information? No, but the CoVPN may share your samples with other researchers. Once the CoVPN shares your samples and information, it may not be able to get them back.

   What information is shared with researchers? The samples and information will be labeled with a code number which will not be removed. The key to the code will stay at this clinic. However, some information that the CoVPN shares may be personal, such as your race, ethnicity, sex, and health information from the study.

   What kind of studies might be done with my extra samples and information? The studies will be related to vaccines, the immune system, coronavirus, and other diseases.

   Researchers may also do genetic testing on your samples.

   If you agree, researchers may compare all of your genes (your genome) to the genomes of many other people. Researchers look for common patterns of genes to help them understand diseases. The researchers may put the information into a protected database so that other researchers can access it. Your name and other personal information will not be included.

Usually, no one could connect your genome to you as a person. There are rules against this. It's also really difficult to do. But there is a risk that someone could combine information from your genome and other public information about you and identify you. If others found out, it could lead to discrimination or other problems. The risk of this happening is very small.

Who will have access to my information in studies using my extra samples?

People who may see your information are:

- Researchers who use your extra samples and information for other research
- Government agencies that fund or monitor the research using your extra samples and information
- The researcher's Institutional Review Board or Ethics Committee
- Any regulatory agency that reviews research studies
- The people who work with the researcher

All of these people will do their best to protect your information. If they publish their research, they will not use your name or identify you personally.

4. **We will compensate you for your participation.**

Site: Insert your compensation plan here, e.g. money for number of swabs returned. Remember to include any costs to participants.

5. **We will do our best to protect your private information.**

Site: Check HIPAA authorization for conflicts with this section.

Your study records and samples will be kept in a secure location. We will label all of your samples and most of your records with a code number, not your name or other personal information. We will not share your name with anyone who does not need to know it.

Your records may also be reviewed by groups who watch over this study. These groups include:

- Study monitors
- The CoVPN, people who work for it, and companies that help it with this study
- Some government agencies:

- The US National Institutes of Health
- The US Office for Human Research Protections
- Any regulatory agency that reviews research studies

- Some committees that make sure we protect your rights and keep you safe:
  - The Independent Data Monitoring Committee
  - Advarra Institutional Review Board (IRB)
  - *Sites may but are not required to include:* [Insert name of local IRB/EC]
  - *Sites include:* [Insert name of local IBC]

All reviewers will take steps to keep your records private.

Sites: Include the following boxed text. You can remove the box around the text.

We have a Certificate of Confidentiality from the US government to help protect your privacy. With the certificate, we do not have to release information about you to someone who is not connected to the study, such as the courts or police. Sometimes we can't use the certificate. Since the US National Institutes of Health funds this research, we cannot withhold information from it.

The CoVPN may share information from this study with other researchers. Researchers may publish the results of this study.

Sites: The text below may not be deleted or changed per FDA requirement. You can remove the box.

A description of this clinical trial will be available on http://www.ClinicalTrials.gov, as required by U.S. Law. This website will not include information that can identify you. At most, the website will include a summary of the results. You can search this website at any time.

6. **We may take you out of the study at any time.**

We may take you out of the study if:

- you do not follow instructions,
- we think that staying in the study might harm you,
- the study is stopped for any reason.

Risks

7. **There are some risks to being in this study.**

   This section describes the risks we know about. There may also be unknown risks, even serious ones. We will tell you if we learn anything new that may affect your willingness to stay in the study.

   Risks of blood draws:

   In this study, we will either take your blood or ask you to self-collect your blood with a small device. Taking blood can cause bruising, pain, fainting, soreness, redness, and itching.

   Risks of swabbing your nose:

   The feeling of having a small, soft-tipped swab inserted into your nostril and twirled around may be a little uncomfortable, but it should not be painful. There is a small chance there could be some bleeding, but this is unlikely.

   Risks to your personal information:

   We will take several steps to protect your personal information. Although the risk is very low, it is possible that your personal information could be given to someone who should not have it. If that happened, you could have stress or anxiety.

   Risks of COVID-19:

   While you are in this study, you should still follow federal, state, and local guidelines for SARS-CoV-2 prevention, which may include wearing a mask and keeping physical distance from others. If you get sick, you should follow COVID-19 precautions and isolate or self-quarantine.

Benefits

8. **This study may not benefit you.**

   This study may help researchers find out if people who got the vaccine and later got infected with SARS-CoV-2 are less likely to give it to people who they have been in close contact with. If the vaccine later becomes approved and sold, there are no plans to share any money with you.

   You do not have to join this research study if you don't want to.

Leaving the study

9. **Tell us if you decide to leave the study.**

   You are free to leave the study at any time and for any reason. If you want to leave, you will need to tell us. Your legal rights will not be affected.

   Previously collected information about you will remain in the study records and will be included in the analysis of results. Your information can not be removed from the study records.

   We may ask you to give a final blood sample or nasal swab.

   We believe these steps are important to protecting your health, but it is up to you whether to complete them.

Injuries

Sites: Approval from CoVPN Regulatory Affairs (at CoVPN.core.reg@fredhutch.org) is needed for any change (other than those that the instructions specifically request or those previously approved by CoVPN Regulatory Affairs) to the boxed text. You can remove the box around the text.

10. **If you have a life-threatening medical emergency, call 911 right away or seek help immediately.**

We do not expect you to get sick or injured as a result of being in this study. If you get COVID-19 while you are in the study, please let us know. *(Sites: adjust the following 2 sentences if applicable to the care available at your site)* We will tell you about the care that we can give here. For the care that we cannot provide, we will explain how we will help you get care elsewhere. You or your health insurance will have to pay for the care you get elsewhere.<br><br>Some injuries are not physical. For example, you might be harmed emotionally by being in this study. Or you might lose wages because you cannot go to work. However, there are no funds to pay for these kinds of injuries, even if they are study related.<br><br>You always have the right to use the court system if you are not satisfied.

Questions

11. **Whom to contact about this study**

    During the study, if you experience any medical problems, suffer a research-related injury, or have questions, concerns, or complaints about the study, please

contact the study doctor at the telephone number listed on the first page of this consent document.

An institutional review board (IRB) is an independent committee established to help protect the rights of research subjects.

If you have any questions about your rights as a research subject, and/or concerns or complaints regarding this research study, contact:
by mail:

Study Subject Adviser
Advarra IRB
6100 Merriweather Dr., Suite 600
Columbia, MD 21044

or call **toll free**: 877-992-4724
or by **email**: adviser@advarra.com

Please reference the following number when contacting the Study Subject Adviser: Pro00049375

Sites may include, but are not required to: You may also contact the [name of local IRB/EC] at [insert contact information].

Your permissions and signature

12. **In Section 3, we told you above about possible other uses of your extra samples** and information outside this study. Please choose only one of the options below and write your initials in the box next to it. Whatever you choose, the CoVPN keeps track of your decision about how your samples and information can be used. You can change your mind after signing this form.

☐ I allow my extra samples and information to be used for other studies related to vaccines, the immune system, coronavirus, and other diseases. This may include genetic testing.

OR

☐ I agree to the option above *and* also to allow my extra samples and information to be used in studies that look at my whole genome.

OR

☐ I do not allow my extra samples to be used in any other studies. This includes not allowing genetic testing, or studies that look at my whole genome.

13. **If you agree to join this study, you will need to sign below. Before you sign, make sure of the following:**

- You have read this consent form.
- You feel that you understand what the study is about and what will happen to you if you join. You understand what the possible risks and benefits are.
- You agree to join this study.

You will not be giving up any of your rights by signing this consent form.

Participant's Name (Print)	Participant's Signature	Date
Clinic Staff Conducting Consent Discussion (Print)	Clinic Staff Signature	Date

Appendix E Procedures for Main Cohort, Immediate Vaccination Group

Visit:	01[1]	02[2]	03	04	05	06
Day:		D1	D29	D57	D113	D141
Week:		W0	W4	W8	W16	W20
Month:		M0	M1	M2	M4	M5
Procedure	Scr.	VAC1	VAC2			
**Study procedures**						
Screening consent (if used)	✓	–	–	–	–	–
Assessment of understanding	✓	–	–	–	–	–
Protocol consent (eConsent)	✓	–	–	–	–	–
Medical history questionnaire[3]	✓	✓	–	–	–	–
SARS-CoV-2 infection risk assessment[3]	✓	Weekly				
COVID-19 symptom surveillance[3]	–	Weekly starts at week 2, not during week 5				
Confirm eligibility	✓	–	–	–	–	–
Obtain demographics[3]	✓	–	–	–	–	–
Randomize	–	✓	–	–	–	–
SARS-CoV-2 testing questionnaire[3]	–	approximately 2x per week				
End of study questionnaire[3]	–	–	–	–	✓	–
**Research Samples[4]**						
Serum for Immunogenicity Assays and Storage	–	✓	–	✓	–	–
Serum for SARS-CoV-2 clinical serology[5]	–	✓[6]	–	✓[6]	✓	–
Nasal swab	–	Daily[7]				–
**Vaccination procedures**						
COVID-19 symptom check[8]	–	✓	✓	–	–	–
Vaccination	–	✓	✓	–	–	–

Greyed out visit column does not apply to this group.

1 Screening may occur over the course of several contacts/visits up to and including day 1, prior to vaccination.

2 Blood draws required at vaccination visits must be performed prior to vaccination.

3 Results from screening at school, if applicable, or at other locations will be captured via eDiary, please see Section 9.16.

4 From screening to the final study visit, the total blood volume to be collected from each participant will be approximately 40 mL (maximum) with a maximum of approximately 17 mL collected in a single visit.

5 For blood self-collected by a participant the maximum volume will be less than 1 mL in a single collection.

6 SARS-CoV-2 clinical serology testing may be performed on leftover blood from samples collected for immunogenicity assays. No separate blood sample is needed. If a Main cohort participant is also in the Prospective Close Contact (PCC) cohort related to

an index case or in the Case-ascertained Close Contact (CACC) cohort, one additional blood collection (self-collection) will be performed (see table below).

7 Daily swabs will be collected from vaccination through D113. Swabs will be returned via drop-off location(s) specified by the clinic or through the mail.

8 COVID-19 Symptom Check to be performed within 24 hours prior to vaccination.

The following **additional** procedures will occur for Main cohort, immediate vaccination participants, who fall under any of the following categories:

- tested positive for SARS-CoV-2 from enrollment through Month 4 (see Section 9.7)
- enrolled also in the PCC cohort and are related to an index case (see Section 9.8)
- enrolled also in the CACC cohort (see Section 9.9)

Day relative to notification of infection:	D1-14
**Study procedures**	
Symptom tracking via eDiary	daily
**Research Samples**	
Serum for SARS-CoV-2 clinical serology[1]	✓[2]

[1] For blood self-collected by a participant the maximum volume will be less than 1 mL in a single collection.

[2] Sample for serology will be collected as soon as possible following report of positive SARS-CoV-2 test.

In addition, we may ask participants to sign a release to allow us to review their COVID-19 related medical records.

Appendix F Procedures for Main Cohort, Standard of Care and Vaccine Declined Groups

Visit:	01[1]	02	03	04	05	06
Day:		D1	D29	D57	D113	D141
Week:		W0	W4	W8	W16	W20
Month:		M0	M1	M2	M4	M5
Procedure	Scr.				VAC1	VAC2
**Study procedures**						
Screening consent (if used)	✓	–		–	–	–
Assessment of understanding	✓	–		–	–	–
Protocol consent (eConsent)	✓	–		–	–	–
Medical history questionnaire[2]	✓	✓		–	–	–
SARS-CoV-2 infection risk assessment[2]	✓	Weekly				–
COVID-19 symptom surveillance[2]	–	Weekly				–
Confirm eligibility	✓	–		–	–	–
Obtain demographics[2]	✓	–		–	–	–
Randomize, Standard of Care Group only	–	✓		–	–	–
SARS-CoV-2 testing questionnaire[2]	–	approximately 2x per week				–
End of study questionnaire[2]	–	–		–	✓[7]	✓
**Research Samples**						
Serum for SARS-CoV-2 clinical serology[3]	–	✓		✓	✓	–
Nasal swab	–	Daily[4]				–
**Vaccination procedures**[6]						
COVID-19 symptom check[5]	–	–		–	✓	✓
Vaccination	–	–		–	✓	✓

Greyed out visit does not apply to this group.

1 Screening may occur over the course of several contacts/visits up to and including day 1, prior to randomization.
2 Results from screening at school, if applicable, or at other locations will be captured via eDiary, please see Section 9.16.
3 From screening to the final study visit, the total blood volume to be collected from each participant will be approximately 15 mL (maximum) with a maximum of approximately 5 mL collected in a single visit. For blood self-collected by a participant the maximum volume will be less than 1 mL in a single collection. If a Main cohort participant is also in the Prospective Close Contact (PCC) cohort related to an index case or in the Case-ascertained Close Contact (CACC) cohort, one additional blood collection (self-collection) will be performed (see table below).
4 Daily swabs will be collected from D1 through D113. Swabs will be returned via drop-off location(s) specified by the clinic or through the mail.

5 COVID-19 Symptom Check to be performed within 24 hours prior to vaccination.
6 Vaccination procedures only apply to participants who have not received COVID-19 vaccine outside the study and for participants in the Vaccine Declined Group who request to be vaccinated.
7 Only applies to participants who are not receiving vaccination on study, ie, who have already received COVID-19 vaccine outside the study or who have declined vaccination (Visit 5 will be the last study visit for these participants).

The following **additional** procedures will occur for Main cohort, standard of care and vaccine declined participants, who fall under any of the following categories:

- tested positive for SARS-CoV-2 from enrollment through Month 4 (see Section 9.7)
- enrolled also in the PCC cohort and are related to an index case (see Section 9.8)
- enrolled also in the CACC cohort (see Section 9.9)

Day relative to notification of infection:	D1-14
**Study procedures**	
Symptom tracking via eDiary	daily
**Research Samples**	
Serum for SARS-CoV-2 clinical serology[1]	✓[2]

[1]For blood self-collected by a participant the maximum volume will be less than 1 mL in a single collection.
[2] Sample for serology will be collected as soon as possible following report of positive SARS-CoV-2 test

In addition, we may ask participants to sign a release to allow us to review their COVID-19 related medical records.

Appendix G Procedures for Prospective Close Contact (PCC) cohort

Visit:	**100**	**101**
Day:	D1	D113
Month:	M0	M4
	Enrollment	Follow-up
**Study procedures**		
Protocol consent (eConsent)	✓	–
Obtain demographics[1]	✓	–
SARS-CoV-2 testing questionnaire[1]	approximately 2x per week	
End of study questionnaire[1]	–	✓

1 Results from screening at school, if applicable, or at other locations will be captured via eDiary, please see Section 9.16.

If a PCC participant (or their Main study contact) tests positive for SARS-CoV-2, the following **additional** procedures will occur:

Day relative to notification of infection:	D1	D29
**Study procedures**		
Report of exposure[1]	✓	
Symptom tracking[1]	Daily for 14 days	–
**Research Samples**		
Serum for SARS-CoV-2 clinical serology[2]	✓	✓
Nasal swab[3]	Daily for 14 days	–

[1] Will be captured via eDiary , please see Section 9.16.
[2]For blood self-collected by a participant the maximum volume will be less than 1 mL in a single collection. Sample for serology will be collected as soon as possible following report of positive SARS-CoV-2 test.
[3]Swabs will be returned via drop-off location(s) specified by the clinic or through the mail. Nasal swabs will be collected as soon as possible following report of positive SARS-CoV-2 test.

In addition, we may ask participants to sign a release to allow us to review their COVID-19 related medical records.

Appendix H Procedures for Case-Ascertained Close Contact Cohort

Visit:	**200**	**201**	**202**
Day:	D1	D1-D3	D29
Month:	M0	M0	M1
	Enrollment	Initial specimen collection	Follow-up
**Study procedures**			
Protocol consent (eConsent)	✓	–	–
Collection of demographic information[1]	✓	–	–
Report of exposure[1]	✓	–	–
Symptom tracking[1]		Daily for 14 days	–
SARS-CoV-2 testing questionnaire[1]		approximately 2x per week	
End of study questionnaire[1]	–	–	✓
**Research Samples**			
Serum for SARS-CoV-2 clinical serology[2]		✓	✓
Nasal swab[3]		Daily for 14 days	–

[1]Results from screening at school, if applicable, or at other locations will be captured via eDiary, please see Section 9.16.
[2] For blood self-collected by a participant the maximum volume will be less than 1 mL in a single collection. Sample for serology will be collected as soon as possible following report of positive SARS-CoV-2 test (ie, as early as Day 1).
[3]Swabs will be returned via drop-off location(s) specified by the clinic or through the mail. Nasal swabs will be collected as soon as possible following report of positive SARS-CoV-2 test (ie, as early as Day 1).

In addition, we may ask participants to sign a release to allow us to review their COVID-19 related medical records.

Appendix I Visit windows

Main Cohort, Immediate Vaccination, Visit Windows

Visit Number	Visit Type	Lower Allowable Window	Lower Target Day	Target Day[1]	Upper Target Day	Upper Allowable Window
01.0	Screening	-56	-		-	0
02.0	**Enrollment/Randomization**[2]	-	-	1	-	-
03.0	**Vaccination 2**	-	-3	29[3]	+7	+14
04.0	Month 2 Follow-up	-	-7	57	+7	+14
05.0	Month 4 Follow-up	-13	-7	113	+14	+56

1.Target dates are relative to Visit 2.0 (Enrollment/Randomization), except for cases described in footnote 3.

2. Completion of post enrollment activities may occur up to approximately 7 days post enrollment (see Section 9.3) .

3. In the event that Vaccination 1 occurs on a different day than Visit 2.0 (Enrollment/Randomization), Visit 3.0 target dates are relative to date of Vaccination 1.

Main Cohort, Standard of Care and Vaccine Declined, Visit Windows

Visit Number	Visit Type	Lower Allowable Window	Lower Target Day	Target Day[1]	Upper Target Day	Upper Allowable Window
01.0	Screening	-56	-		-	0
02.0	**Enrollment/Randomization**[2]	-	-	1	-	-
04.0	Month 2 Follow-up	-	-7	57	+7	+14
05.0	**Vaccination 1**[3]	-13	-7	113	+14	+28
06.0	**Vaccination 2**[1, 3]	-	-3	141	+7	+14

1. Target dates are relative to Visit 2.0 (Enrollment/Randomization), except visit 06.0, which is relative to visit 05.0.

2. Completion of post enrollment activities may occur up to approximately 7 days post enrollment (see Section 9.5) .

3. Vaccination visits only apply to participants who have not received COVID-19 vaccine outside the study and for participants in the Vaccine Declined Group who request to be vaccinated.

Case-Ascertained Close Contact (CACC) Cohort and applicable Prospective Close Contact (PCC) participants[1], Visit Windows

Visit Number	Visit Type	Lower Allowable Window	Lower Target Day	Target Day[2]	Upper Target Day	Upper Allowable Window
200.0	**Enrollment**	-	-	1	-	-
201.0	Initial blood specimen collection		-2	3	+1	+4
202.0	Day 29 Follow-up	-	-7	29	+14	+28

1. Applicable to PCC participants when they (or their Main study contact) tests positive for SARS-CoV-2.

2. Target dates are relative to Visit 200.0 (Enrollment).

Appendix J Protocol Signature Page

A randomized controlled study to assess SARS CoV-2 infection, viral shedding, and subsequent potential transmission in individuals immunized with Moderna COVID-19 Vaccine

I will conduct the study in accordance with the provisions of this protocol and all applicable protocol-related documents. I agree to conduct this study in compliance with United States (U.S.) Health and Human Service regulations (45 CFR 46); applicable U.S. Food and Drug Administration regulations; standards of the International Council for Harmonisation of Technical Requirements for Pharmaceuticals for Human Use (ICH) Guideline for Good Clinical Practice (E6) (R2); Institutional Review Board/Ethics Committee determinations; all applicable in-country, state, and local laws and regulations; and other applicable requirements (eg, U.S. National Institutes of Health, Division of AIDS) and institutional policies.

Investigator of Record Name (print) | Investigator of Record Signature | Date

Protocol Number: CoVPN 3006

Protocol Version: Version 4.0

Protocol Date: May 20, 2021

COVID-19
Prevention Network

FINAL

June 15, 2021

Letter of Amendment 1

Protocol

Version 4.0

CoVPN 3006

A randomized controlled study to assess SARS CoV-2 infection, viral shedding, and subsequent potential transmission in individuals immunized with Moderna COVID-19 Vaccine

DAIDS-ES ID 38822

NON-IND STUDY

COVID-19 Prevention network (CoVPN) Clinical Research Site (CRS) filing instructions

The following information impacts the CoVPN 3006 study and must be forwarded to your Institutional Review Board (IRB)/Ethics Committee (EC) and any other applicable Regulatory Entity (RE) as soon as possible for their information and review. Their approval is required before implementation.

Upon receiving final IRB/EC and any other applicable RE approval(s), CRSs are required to submit regulatory documents to PPD. CRSs will receive a notification once PPD verifies that all the required regulatory documents have been received and are complete. CRSs are **not** required to wait for PPD to grant amendment registration in order to implement.

A copy of the notifications, along with this letter of amendment (LoA) and any IRB/EC and RE correspondence, should be retained in the CRS's regulatory files.

The following information does not affect the sample informed consent. The CRS's IRB/EC is responsible for determining the process of informing study participants of the contents of this LoA.

List of changes

Item 1 Added in Section 7.4, *Exclusion criteria for Main cohort, Vaccine Declined Group*: exclusion criterion for history of SARS-CoV-2 infection 2

The changes described herein will be incorporated in the next version of Protocol CoVPN 3006 if it undergoes full protocol amendment at a later time.

Item 1 Added in Section 7.4, *Exclusion criteria for Main cohort, Vaccine Declined Group*: exclusion criterion for history of SARS-CoV-2 infection

In order to harmonize the SARS-CoV-2 infection risk among all groups in the Main Cohort, the exclusion criterion regarding SARS-CoV-2 infection history listed for the Immediate Vaccination Group and the Standard of Care Group has been added as a new criterion for the Vaccine Decline Group.

Revised, new text is shown in underline:

7.4 Exclusion criteria for Main cohort, Vaccine Declined Group

1. **Prior administration of a coronavirus** (SARS-CoV-2, SARS-CoV, MERS-CoV) **vaccine** or current/planned simultaneous participation in another interventional study to prevent or treat COVID-19 (participation in studies of other investigational research agents allowed).

2. **Any medical, psychiatric, occupational, or other condition** that, in the judgment of the investigator, would interfere with, or serve as a contraindication to, protocol adherence, or a volunteer's ability to give informed consent.

3. **Self-reported known history of SARS-CoV-2 infection.**

Protocol modification history

Protocol modifications are made to CoVPN protocols via clarification memos, letters of amendment, or full protocol amendments.

The version history of, and modifications to, Protocol CoVPN 3006 are described below.

Date: June 15, 2021

Protocol version: Version 4.0
Protocol modification: Letter of Amendment 1

Item 1 Added in Section 7.4, *Exclusion criteria for Main cohort, Vaccine Declined Group*: exclusion criterion for history of SARS-CoV-2 infection

Date: May 20, 2021

Protocol version: Version 4.0

Protocol modification: Full Protocol Amendment 3

Item 1 Revised in Title page, Section 1, Overview, Section 2, Background, Section 6, Statistical considerations, Section 7.1, Inclusion criteria for main cohort, Appendices A, C, and D, Sample informed consent forms, and Appendices E-H, Procedures: definition of Main cohort participant expanded to include non-student adults aged 18 through 29

Item 2 Revised throughout the protocol: delayed vaccination group renamed standard of care group

Item 3 Added in Section 1, Overview, Section 2, Background, Section 6, Statistical considerations, Section 7.1 and 7.2, Inclusion and Exclusion criteria for Main Cohort, Immediate Vaccination and Standard of Care groups, Section 7.3, Inclusion criteria for Main cohort, Vaccine Declined group, Section 7.4, Exclusion criteria for Main cohort, Vaccine Declined Group, Section 9, Clinical procedures, Appendix B, Sample informed consent form for Main cohort, Vaccine Declined Group, Appendices A, C and D, Sample informed consent forms, and Appendix I, Visit windows: new observational group, “Vaccine Declined” group, of up to 6000 participants

Item 4 Added in Section 7.2, Exclusion criteria for Main Cohort, Immediate Vaccination Group and Standard of Care Group: exclusion for volunteers who prefer to be vaccinated immediately

Item 5 Revised in Section 1, Overview and Appendices A, C, and D, Sample informed consent forms: number of potential participants in close contact cohorts increased

Item 6 Updated in Section 1, Overview: increased estimated total study duration from 7 months to 9 months

Item 7 Clarified in Section 2.10, Moderna COVID-19 Vaccine, and Section 8.3, Acquisition of Moderna COVID-19 Vaccine: participants may also access Moderna COVID-19 EUA vaccine via other sources

Item 8 Revised in Section 3, Objectives and endpoints and Section 6, Statistical considerations: inclusion of all SARS-CoV-2 infection events in analysis for primary endpoints and secondary endpoints 1-5

Item 9 Revised in Section 3, Objectives and endpoints, exploratory objective 1: expanded to main study cohort

Item 10 Revised in Section 7.1, Inclusion criteria for Main Cohort, Immediate Vaccination Group and Standard of Care Group: timing of completion of assessment of understanding

Item 11 Revised in Sections 7.1, 7.3, 7.5, and 7.6, Inclusion criteria for all groups: access to device and internet is needed for completion of study procedures

Item 12 Added in Section 8, Study product: definitions of on-study and outside of study vaccination

Item 13 Clarified in Section 8, Study product: study allocated vaccine can only be administered to study participants

Item 14 Added in Section 8.3, Acquisition of Moderna COVID-19 Vaccine: participants can access Moderna COVID-19 EUA vaccine via other sources for on-study vaccinations if the administration fee is either paid for by the study or is not billed to any party

Item 15 Deleted from Section 9.3, Enrollment and vaccination visits for Main Cohort, Immediate Vaccination Group and Section 9.6, Month 4 (visit 5) and Month 5 (visit 6) Visits for Main Cohort, Standard of Care Group and Vaccine Declined Group: parenthetical reference to temperature in COVID-19 symptom check

Item 16 Added in Section 9, Clinical Procedures, and Appendix F, Procedures for Main Cohort, Standard of Care and Vaccine Declined Groups: end of study questionnaires as procedures

Item 17 Added in Section 9.1.2, Protocol-specific consent forms: consent forms for close contact cohorts may be managed centrally by the CoVPN

Item 18 Clarified in Section 9.15, COVID-19 symptom surveillance for Main Cohort: symptom overlap between COVID-19 and vaccination is only applicable to Immediate Vaccination Group

Item 19 Corrected in Appendix A, Sample informed consent form for the Main cohort, Immediate Vaccination and Standard of Care Groups, Appendix C, Sample informed consent form for Prospective Close Contact (PCC) cohort and Appendix D, Sample informed consent form for Case-Ascertained Close Contact (CACC) cohort: frequency of eDiary entries.

Item 20 Added in Appendix A, Sample informed consent form for the Main cohort, Immediate Vaccination and Standard of Care Groups: we may ask Main cohort participants to provide contact information for potential close contact cohort volunteers

Item 21 Clarified in Appendix A, Sample informed consent form for the Main cohort, Immediate Vaccination and Standard of Care Groups: Month 4 will be the last study visit for Standard of Care participants who receive COVID-19 vaccine outside the study prior to Month 4

Item 22 Added in Appendix A, Sample informed consent form for the Main cohort, Immediate Vaccination and Standard of Care Groups: the risk of delaying COVID-19 vaccination

Item 23 Added in Appendix C, Sample informed consent form for Prospective Close Contact (PCC) cohort and Appendix D, Sample informed consent form for Case-Ascertained Close Contact (CACC) cohort: new section "Risks of COVID-19"

Item 24 Deleted from Appendices A, C and D, Sample informed consent forms: sentence that states we will not share your name or identifying information with the CoVPN

Item 25 Clarified in Appendix A, Sample informed consent form for the Main cohort, Immediate Vaccination and Standard of Care Groups: benefits of COVID-19 vaccination

Item 26 Added in Appendices A, C, and D, Sample informed consent forms: participants will be prompted to scan the barcode on nasal swabs

Item 27 Updated in Appendices A, C, and D, Sample informed consent forms: recommendation to follow federal, state, and local guidelines for SARS-CoV-2 prevention

Item 28 Updated in Appendices A, C, and D, Sample informed consent forms: contact information for Advarra IRB

Item 29 Updated in Appendix A, Sample informed consent form for the Main cohort, Immediate Vaccination and Standard of Care Groups: Moderna COVID-19 Vaccine, risks and number of vaccinated people

Item 30 Updated Title page and Section 13, Version history: contents of this amendment

Item 31 Throughout the protocol: minor editorial changes, clarifications, and corrections

Date: April 12, 2021

Protocol version: 3.0
Protocol modification: Full protocol amendment 2

Item 1 Added in Section 1, *Overview* and Section 6, *Statistical considerations*: Clinical Research Sites (CRSs) that can participate in the study expanded to include non-university affiliated CoVPN Clinical Research Sites (CRSs)

Item 2 Revised in Section 1, *Overview*, Section 2, *Background*, Section 6, *Statistical considerations*, Section 7.1, *Inclusion criteria for main cohort*, Section 9, *Clinical procedures*, and Appendix A, *Sample informed consent form for the Main cohort*: definition of participant expanded to include students enrolled in any post-secondary educational institution

Item 3 Revised in Section 1, *Overview*, Section 2, *Background*, Section 6, *Statistical considerations,* Section 7.3*, Inclusion criteria for Prospective Close Contact (PCC) cohort,* Section 9*, Clinical procedures,* and Appendices A, B, D, E, F: University SARS-CoV-2 testing is not required

Item 4 Revised in Section 9.3*, Enrollment and vaccination visits for Main Cohort, Immediate Vaccination Group*, Section 9.5, *Enrollment (visit 2) and visit 4 (Month 2) for Main Cohort, Delayed Vaccination Group* and Appendix H, *Visit windows*: maximum recommended time between randomization and completion of Visit 2 activities increased from 4 days to 7 days

Item 5 Clarified in Section 2, *Background*, Section 6, *Statistical considerations*, and Section 9.7, *Procedures for Main Cohort participant diagnosed with a SARS-CoV-2 infection during the study*: any SARS-CoV-2 test result indicative of acute infection (on or off study) obtained from a Main cohort participant will trigger "index-case" activities

Item 6 Clarified in Section 2, *Background*, Section 6.4.3, *Vaccine efficacy (VE) analyses against SARS-CoV-2 infection*, Section 7.1, *Inclusion criteria for main cohort*, Section 9, *Clinical procedures*, Appendix A, *Sample informed consent form for Main cohort*, and Appendix B, *Sample informed consent form for Prospective Close Contact (PCC) cohort*: participants can choose to obtain an EUA or licensed COVID-19 vaccine outside the study

Item 7 Added to Section 3, *Objectives and endpoints*: 2 endpoints for the duration of viral shedding specified in corresponding secondary objective 4

Item 8 Clarified in Section 6, *Statistical considerations, 7th paragraph:* primary analysis occurs when participants complete 4 months of follow-up

Item 9 Clarified in Table 6-1, *Study analysis sets and definitions*: Day 29 is visit 3 for immediate vaccination arm and will be define in Statistical analysis plan (SAP) for delayed vaccination arm

Item 10 Revised in Section 6.3, *Randomization*: randomization evaluated within levels defined by study site and type of residence

Item 11 Clarified in Section 6.4.3.1, *Primary VE Analysis*: rationale for choice of analysis

Item 12 Revised in Section 6.4.5.2, *Analyses of the vaccine effect on secondary transmission*: details of the derivation

Item 13 Revised in Section 7.2, Exclusion criterion #1 for Main Cohort: removed febrile assessment

Item 14 Clarified in Section 2.10, *Moderna COVID-19 Vaccine*, and Section 8.3, *Acquisition of Moderna COVID-19 Vaccine*: Moderna COVID-19 Vaccine provided under EUA and allocated for this trial

Item 15 Corrected in Section 9, *Clinical procedures*: CRS staff will obtain informed consent of participants according to the COVPN 3006 SSP

Item 16 Corrected in Appendix A, *Sample informed consent form for Main cohort:* blood will be collected 3 times for delayed vaccination arm

Item 17 Added to Appendix B, *Sample informed consent form for Prospective Close Contact (PCC)* cohort: COVID-19 care is not paid for by the study

Item 18 Added to Appendices A, B, C, *Sample informed consent forms*: information regarding employee/family member participation

Item 19 Added to Appendices A, B, C, *Sample informed consent forms*: terms of use for data collection via web-based app

Item 20 Deleted in Appendix B, *Sample informed consent form for Prospective Close Contact (PCC)* cohort, and Appendix C, Sample informed consent form for Case-Ascertained Close Contact (CACC) cohort, item 8: text referring to vaccine receipt

Item 21 Added to Appendix H, *Visit windows for CACC:* upper allowable window for blood specimen collection

Item 22 Added changes per Protocol v 2.0, Letter of Amendment 1

Item 23 Updated Title page and Section 13, *Version history*: contents of this amendment

Item 24 Updated Section 1.1, *Protocol team*: deletion of Site Principal Investigator representative

Item 25 Throughout the protocol: minor editorial changes, clarifications, and corrections

Date: March 10, 2021

Protocol version: Version 2.0

Protocol modification: Letter of Amendment 1

Item 1 Added in Section 10.1, *CRS laboratory procedures*, Appendices A, B, and C, *Sample informed consent forms*: return of SARS-CoV-2 nasal swab testing results to participants

Item 2 Added to Section 1.1, *Protocol team*: Jasmine Marcelin, co-chair and Jeffrey L Carson, Site Principal Investigator representative

Date: February 26, 2021

Protocol version: 2.0

Protocol modification: Full protocol amendment 1

Item 1 Revised in Section 3, *Objective and Endpoints,* Section 6, *Statistical considerations,* Section 9, *Clinical procedures*, Appendix A, *Sample informed consent form for the main cohort*, and Appendices D and E, *Procedures for the main cohort*: Month 3 visit removed for both Main cohort groups, M1 visit removed for delayed vaccination group of Main cohort, and Month 1 blood collection removed for immediate vaccination group of Main cohort

Item 2 Added in Section 1, *Overview*: 2 new endpoint laboratories

Item 3 Updated in Section 1.1, *Protocol team*: team members

Item 4 Deleted Section 6.1, *Accrual restrictions*: no accrual restrictions for this study

Item 5 Revised in Section 6.4, *Statistical analyses*: minor clarifying text revisions

Item 6 Corrected in Section 6.4.4.7, *VL analyses,* 3rd paragraph: reference to secondary endpoint number

Item 7 Deleted in Section 9.3, *Enrollment and vaccination visits for Main Cohort, Immediate Vaccination Group* and Section 9.5, *Enrollment (visit 2) and visit 4 (Month 2) for Main Cohort, Delayed Vaccination Group:* sentence regarding clinical lab results

Item 8 Added in Section 9.15, *Use of electronic Diary (eDiary) for Clinical Outcomes Assessments,* and Appendices F through G, *Procedures*: end of study questionnaire regarding COVID vaccines received outside the study

Item 9 Added in Appendices A, B, and C, *Sample informed consent forms*, section “Being in the study”: participants will take an end of study questionnaire

Item 10 Added in Appendix B, *Sample informed consent form for Prospective Close Contact (PCC) cohort*, and Appendix C, *Sample informed consent form for Case-Ascertained Close Contact (CACC) cohort*, section “Being in the study”: applicable participants will receive SARS-CoV-2 screening at the University about two times per week.

Item 11 Deleted in Appendices A, B, and C, *Sample informed consents,* second sentence*:* reference to alternate study name of "vPROTECT"

Item 12 Added in Appendix A, *Sample informed consent form for the main cohort:* additional information for participant regarding text or email reminders for nasal swabs

Item 13 Deleted in Appendices A, B and C, *Sample informed consents:* reference to "electronically" for signature

Item 14 Deleted in Appendices A, B and C, *Sample informed consents*, signature table: column for denoting time of signature

Item 15 Deleted in Appendix A, *Sample informed consent form for the main cohort*, section 8: redundant information

Item 16 Corrected in in Appendix A, *Sample informed consent form for the main cohort*, section10: VAERS reporting requirements

Item 17 Revised in Appendices F and G: table text and associated footnotes

Item 18 Revised Appendix H, *Visit windows,* Case-Ascertained Close Contact (CACC) cohort, to include applicable Prospective Close Contact (PCC) participants who test positive for SARS-CoV-2 or whose Main Cohort study contact tests positive for SARS-CoV-2

Item 19 Revised throughout protocol: minor grammatical, editorial, and formatting corrections.

Item 20 Updated Title page, Table of Contents, Section 13, *Version history*, and Appendix I*, Protocol signature page*: dates, version number, and contents of this amendment

Date: February 17, 2021

Protocol version:1.0
Protocol modification: Not applicable

Original protocol

Protocol Signature Page

A randomized controlled study to assess SARS CoV-2 infection, viral shedding, and subsequent potential transmission in individuals immunized with Moderna COVID-19 Vaccine

I will conduct the study in accordance with the provisions of this protocol and all applicable protocol-related documents. I agree to conduct this study in compliance with United States (US) Health and Human Service regulations (45 CFR 46); applicable U.S. Food and Drug Administration regulations; standards of the International Conference on Harmonization Guideline for Good Clinical Practice (E6); Institutional Review Board/Ethics Committee determinations; all applicable in-country, state, and local laws and regulations; and other applicable requirements (e.g., US National Institutes of Health, Division of AIDS) and institutional policies

---

Investigator of Record Name (print)	Investigator of Record Signature	Date

DAIDS Protocol Number: CoVPN 3006

DAIDS Protocol Version: Version 4.0

Protocol Date: May 20, 2021

FINAL

July 07, 2021

Letter of Amendment 2

Protocol

Version 4.0

CoVPN 3006

A randomized controlled study to assess SARS CoV-2 infection, viral shedding, and subsequent potential transmission in individuals immunized with Moderna COVID-19 Vaccine

DAIDS-ES ID 38822

NON-IND STUDY

COVID-19 Prevention network (CoVPN) Clinical Research Site (CRS) filing instructions

The following information impacts the CoVPN 3006 study and must be forwarded to your Institutional Review Board (IRB)/Ethics Committee (EC) and any other applicable Regulatory Entity (RE) as soon as possible for their information and review. Their approval is required before implementation.

Upon receiving final IRB/EC and any other applicable RE approval(s), CRSs are required to submit regulatory documents to PPD per the Study Specific Procedure (SSP) on Protocol Registration and Activation. CRSs will receive a notification once PPD verifies that all the required regulatory documents have been received and are complete. CRSs are **not** required to wait for PPD to grant amendment registration in order to implement.

A copy of the notifications, along with this letter of amendment (LoA) and any IRB/EC and RE correspondence, should be retained in the CRS's regulatory files.

The following information does affect the sample informed consents.

List of changes

Item 1 Added in Appendix A, *Sample informed consent form for Main cohort, Immediate Vaccination and Standard of Care Groups* and the Addendum to Appendix B, *Sample informed consent form for Vaccine Declined Group participants who decide to take up Moderna COVID-19 Vaccine on study*: myocarditis and pericarditis as risks of the Moderna COVID-19 Vaccine................ 2

The changes described herein will be incorporated in the next version of Protocol CoVPN 3006 if it undergoes full protocol amendment at a later time.

Item 1 Added in Appendix A, *Sample informed consent form for Main cohort, Immediate Vaccination and Standard of Care Groups* and the Addendum to Appendix B, *Sample informed consent form for Vaccine Declined Group participants who decide to take up Moderna COVID-19 Vaccine on study*: myocarditis and pericarditis as risks of the Moderna COVID-19 Vaccine

Post emergency-use authorization (EUA) surveillance of adverse events following Moderna COVID-19 vaccination has identified myocarditis and pericarditis as new potential risks. The "Fact Sheet For Recipients And Caregivers Emergency Use Authorization (EUA) of The Moderna Covid-19 Vaccine To Prevent Coronavirus Disease 2019 (COVID-19) In Individuals 18 Years Of Age And Older" (https://www.modernatx.com/covid19vaccine-eua/eua-fact-sheet-recipients.pdf) has been updated accordingly, and the new risk information has been added to the protocol sample informed consents as indicated below.

Revised in both Sample informed consent forms under section 1, *This study's vaccine has an Emergency Use Authorization (EUA) for individuals 18 and older*. New text is shown in underline:

Risks of the Moderna COVID-19 Vaccine:

To date, more than 100 million people have already received the Moderna COVID-19 Vaccine. In clinical trials, most people who got the vaccine had some reaction after their injections, especially after the second injection. The reaction to the vaccine didn't affect their daily lives and it went away after two or three days. Most of these people said they had pain in the arm where they got the injection. These people also felt tired, had headaches, muscle and joint pain, and chills. A much smaller number of these people said they had redness or swelling where the needle went in their arm. Some people had swelling of the lymph nodes in the same arm of the injection.

The risks of the study vaccine seen so far are the same as what have been seen with most vaccines. Generally, vaccines can cause fever, chills, rash, aches and pains, nausea, headache, dizziness, and feeling tired. Vaccines can also cause pain, redness, swelling, or itching where you got the injection. Most people can still do their planned activities after getting a vaccine. Rarely, people have side effects that limit their normal activities or make them go to the doctor.

Rarely, a vaccine can cause you to have an allergic reaction. You might have a rash, hives, swelling of your face and throat, a fast heartbeat, dizziness and weakness, or trouble breathing. Allergic reactions can be life-threatening. Tell us if you have ever had a bad reaction to any injection or vaccine.

Myocarditis (inflammation of the heart muscle) and pericarditis (inflammation of the lining around the heart) have occurred in some people who got mRNA COVID-19 vaccines like the Moderna COVID-19 vaccine. They have been more common in young adults than in older people. In most of these people, symptoms began within a few days after the second dose of the vaccine. Almost all people have responded well to medications and rest, and their symptoms improved quickly. The chance of having this inflammation occur is very low.

You should seek medical attention right away if you have any of the following symptoms after getting the Moderna COVID-19 vaccine:

- **Chest pain**
- **Shortness of breath**
- **Having a fast-beating, fluttering, or pounding heart.**

There may be other risks that we don't yet know about, even serious ones. We will tell you if we learn about any new risks.

Protocol modification history

Protocol modifications are made to CoVPN protocols via clarification memos, letters of amendment, or full protocol amendments.

The version history of, and modifications to, Protocol CoVPN 3006 are described below.

Date: July 07, 2021

Protocol version: Version 4.0
Protocol modification: Letter of Amendment 2

Item 1 Added in Appendix A, *Sample informed consent form for Main cohort, Immediate Vaccination and Standard of Care Groups* and the Addendum to Appendix B, *Sample informed consent form for Vaccine Declined Group participants who decide to take up Moderna COVID-19 Vaccine on stud*y: myocarditis and pericarditis as risks of the Moderna COVID-19 Vaccine

Date: June 15, 2021

Protocol version: Version 4.0
Protocol modification: Letter of Amendment 1

Item 1 Added in Section 7.4, *Exclusion criteria for Main cohort, Vaccine Declined Group*: exclusion criterion for history of SARS-CoV-2 infection

Date: May 20, 2021

Protocol version: Version 4.0
Protocol modification: Full Protocol Amendment 3

Item 1 Revised in Title page, Section 1, Overview, Section 2, Background, Section 6, Statistical considerations, Section 7.1, Inclusion criteria for main cohort, Appendices A, C, and D, Sample informed consent forms, and Appendices E-H, Procedures: definition of Main cohort participant expanded to include non-student adults aged 18 through 29

Item 2 Revised throughout the protocol: delayed vaccination group renamed standard of care group

Item 3 Added in Section 1, Overview, Section 2, Background, Section 6, Statistical considerations, Section 7.1 and 7.2, Inclusion and Exclusion criteria for Main Cohort, Immediate Vaccination and Standard of Care groups, Section 7.3, Inclusion criteria for Main cohort, Vaccine Declined group, Section 7.4, Exclusion criteria for Main cohort, Vaccine Declined Group, Section 9, Clinical procedures, Appendix B, Sample informed consent form for Main cohort, Vaccine Declined Group, Appendices A, C and D, Sample informed consent forms, and Appendix I, Visit windows: new observational group, “Vaccine Declined” group, of up to 6000 participants

Item 4 Added in Section 7.2, Exclusion criteria for Main Cohort, Immediate Vaccination Group and Standard of Care Group: exclusion for volunteers who prefer to be vaccinated immediately

Item 5 Revised in Section 1, Overview and Appendices A, C, and D, Sample informed consent forms: number of potential participants in close contact cohorts increased

Item 6 Updated in Section 1, Overview: increased estimated total study duration from 7 months to 9 months

Item 7 Clarified in Section 2.10, Moderna COVID-19 Vaccine, and Section 8.3, Acquisition of Moderna COVID-19 Vaccine: participants may also access Moderna COVID-19 EUA vaccine via other sources

Item 8 Revised in Section 3, Objectives and endpoints and Section 6, Statistical considerations: inclusion of all SARS-CoV-2 infection events in analysis for primary endpoints and secondary endpoints 1-5

Item 9 Revised in Section 3, Objectives and endpoints, exploratory objective 1: expanded to main study cohort

Item 10 Revised in Section 7.1, Inclusion criteria for Main Cohort, Immediate Vaccination Group and Standard of Care Group: timing of completion of assessment of understanding

Item 11 Revised in Sections 7.1, 7.3, 7.5, and 7.6, Inclusion criteria for all groups: access to device and internet is needed for completion of study procedures

Item 12 Added in Section 8, Study product: definitions of on-study and outside of study vaccination

Item 13 Clarified in Section 8, Study product: study allocated vaccine can only be administered to study participants

Item 14 Added in Section 8.3, Acquisition of Moderna COVID-19 Vaccine: participants can access Moderna COVID-19 EUA vaccine via other sources for on-study vaccinations if the administration fee is either paid for by the study or is not billed to any party

Item 15 Deleted from Section 9.3, Enrollment and vaccination visits for Main Cohort, Immediate Vaccination Group and Section 9.6, Month 4 (visit 5) and Month 5 (visit 6) Visits for Main Cohort, Standard of Care Group and Vaccine Declined Group: parenthetical reference to temperature in COVID-19 symptom check

Item 16 Added in Section 9, Clinical Procedures, and Appendix F, Procedures for Main Cohort, Standard of Care and Vaccine Declined Groups: end of study questionnaires as procedures

Item 17 Added in Section 9.1.2, Protocol-specific consent forms: consent forms for close contact cohorts may be managed centrally by the CoVPN

Item 18 Clarified in Section 9.15, COVID-19 symptom surveillance for Main Cohort: symptom overlap between COVID-19 and vaccination is only applicable to Immediate Vaccination Group

Item 19 Corrected in Appendix A, Sample informed consent form for the Main cohort, Immediate Vaccination and Standard of Care Groups, Appendix C, Sample informed consent form for Prospective Close Contact (PCC) cohort and Appendix D, Sample informed consent form for Case-Ascertained Close Contact (CACC) cohort: frequency of eDiary entries.

Item 20 Added in Appendix A, Sample informed consent form for the Main cohort, Immediate Vaccination and Standard of Care Groups: we may ask Main cohort participants to provide contact information for potential close contact cohort volunteers

Item 21 Clarified in Appendix A, Sample informed consent form for the Main cohort, Immediate Vaccination and Standard of Care Groups: Month 4 will be the last study visit for Standard of Care participants who receive COVID-19 vaccine outside the study prior to Month 4

Item 22 Added in Appendix A, Sample informed consent form for the Main cohort, Immediate Vaccination and Standard of Care Groups: the risk of delaying COVID-19 vaccination

Item 23 Added in Appendix C, Sample informed consent form for Prospective Close Contact (PCC) cohort and Appendix D, Sample informed consent form for Case-Ascertained Close Contact (CACC) cohort: new section "Risks of COVID-19"

Item 24 Deleted from Appendices A, C and D, Sample informed consent forms: sentence that states we will not share your name or identifying information with the CoVPN

Item 25 Clarified in Appendix A, Sample informed consent form for the Main cohort, Immediate Vaccination and Standard of Care Groups: benefits of COVID-19 vaccination

Item 26 Added in Appendices A, C, and D, Sample informed consent forms: participants will be prompted to scan the barcode on nasal swabs

Item 27 Updated in Appendices A, C, and D, Sample informed consent forms: recommendation to follow federal, state, and local guidelines for SARS-CoV-2 prevention

Item 28 Updated in Appendices A, C, and D, Sample informed consent forms: contact information for Advarra IRB

Item 29 Updated in Appendix A, Sample informed consent form for the Main cohort, Immediate Vaccination and Standard of Care Groups: Moderna COVID-19 Vaccine, risks and number of vaccinated people

Item 30 Updated Title page and Section 13, Version history: contents of this amendment

Item 31 Throughout the protocol: minor editorial changes, clarifications, and corrections

Date: April 12, 2021

Protocol version: 3.0
Protocol modification: Full protocol amendment 2

Item 1 Added in Section 1, *Overview* and Section 6, *Statistical considerations*: Clinical Research Sites (CRSs) that can participate in the study expanded to include non-university affiliated CoVPN Clinical Research Sites (CRSs)

Item 2 Revised in Section 1, *Overview*, Section 2, *Background*, Section 6, *Statistical considerations*, Section 7.1, *Inclusion criteria for main cohort*, Section 9, *Clinical procedures*, and Appendix A, *Sample informed consent form for the Main cohort*: definition of participant expanded to include students enrolled in any post-secondary educational institution

Item 3 Revised in Section 1, *Overview*, Section 2, *Background*, Section 6, *Statistical considerations,* Section 7.3*, Inclusion criteria for Prospective Close Contact (PCC) cohort,* Section 9*, Clinical procedures,* and Appendices A, B, D, E, F: University SARS-CoV-2 testing is not required

Item 4 Revised in Section 9.3*, Enrollment and vaccination visits for Main Cohort, Immediate Vaccination Group*, Section 9.5, *Enrollment (visit 2) and visit 4 (Month 2) for Main Cohort, Delayed Vaccination Group* and Appendix H, *Visit windows*: maximum recommended time between randomization and completion of Visit 2 activities increased from 4 days to 7 days

Item 5 Clarified in Section 2, *Background*, Section 6, *Statistical considerations*, and Section 9.7, *Procedures for Main Cohort participant diagnosed with a SARS-CoV-2 infection during the study*: any SARS-CoV-2 test result indicative of acute infection (on or off study) obtained from a Main cohort participant will trigger "index-case" activities

Item 6 Clarified in Section 2, *Background*, Section 6.4.3, *Vaccine efficacy (VE) analyses against SARS-CoV-2 infection*, Section 7.1, *Inclusion criteria for main cohort*, Section 9, *Clinical procedures*, Appendix A, *Sample informed consent form for Main cohort*, and Appendix B, *Sample informed consent form for Prospective Close Contact (PCC) cohort*: participants can choose to obtain an EUA or licensed COVID-19 vaccine outside the study

Item 7 Added to Section 3, *Objectives and endpoints*: 2 endpoints for the duration of viral shedding specified in corresponding secondary objective 4

Item 8 Clarified in Section 6, *Statistical considerations, 7th paragraph:* primary analysis occurs when participants complete 4 months of follow-up

Item 9 Clarified in Table 6-1, *Study analysis sets and definitions*: Day 29 is visit 3 for immediate vaccination arm and will be define in Statistical analysis plan (SAP) for delayed vaccination arm

Item 10 Revised in Section 6.3, *Randomization*: randomization evaluated within levels defined by study site and type of residence

Item 11 Clarified in Section 6.4.3.1, *Primary VE Analysis*: rationale for choice of analysis

Item 12 Revised in Section 6.4.5.2, *Analyses of the vaccine effect on secondary transmission*: details of the derivation

Item 13 Revised in Section 7.2, Exclusion criterion #1 for Main Cohort: removed febrile assessment

Item 14 Clarified in Section 2.10, *Moderna COVID-19 Vaccine*, and Section 8.3, *Acquisition of Moderna COVID-19 Vaccine*: Moderna COVID-19 Vaccine provided under EUA and allocated for this trial

Item 15 Corrected in Section 9, *Clinical procedures*: CRS staff will obtain informed consent of participants according to the COVPN 3006 SSP

Item 16 Corrected in Appendix A, *Sample informed consent form for Main cohort:* blood will be collected 3 times for delayed vaccination arm

Item 17 Added to Appendix B, *Sample informed consent form for Prospective Close Contact (PCC)* cohort: COVID-19 care is not paid for by the study

Item 18 Added to Appendices A, B, C, *Sample informed consent forms*: information regarding employee/family member participation

Item 19 Added to Appendices A, B, C, *Sample informed consent forms*: terms of use for data collection via web-based app

Item 20 Deleted in Appendix B, *Sample informed consent form for Prospective Close Contact (PCC)* cohort, and Appendix C, Sample informed consent form for Case-Ascertained Close Contact (CACC) cohort, item 8: text referring to vaccine receipt

Item 21 Added to Appendix H, *Visit windows for CACC:* upper allowable window for blood specimen collection

Item 22 Added changes per Protocol v 2.0, Letter of Amendment 1

Item 23 Updated Title page and Section 13, *Version history*: contents of this amendment

Item 24 Updated Section 1.1, *Protocol team*: deletion of Site Principal Investigator representative

Item 25 Throughout the protocol: minor editorial changes, clarifications, and corrections

Date: March 10, 2021

Protocol version: Version 2.0
Protocol modification: Letter of Amendment 1

Item 1 Added in Section 10.1, *CRS laboratory procedures*, Appendices A, B, and C, *Sample informed consent forms*: return of SARS-CoV-2 nasal swab testing results to participants

Item 2 Added to Section 1.1, *Protocol team*: Jasmine Marcelin, co-chair and Jeffrey L Carson, Site Principal Investigator representative

Date: February 26, 2021

Protocol version: 2.0
Protocol modification: Full protocol amendment 1

Item 1 Revised in Section 3, *Objective and Endpoints,* Section 6, *Statistical considerations,* Section 9, *Clinical procedures*, Appendix A, *Sample informed consent form for the main cohort*, and Appendices D and E, *Procedures for the main cohort*: Month 3 visit removed for both Main cohort groups, M1 visit removed for delayed vaccination group of Main cohort, and Month 1 blood collection removed for immediate vaccination group of Main cohort

Item 2 Added in Section 1, *Overview*: 2 new endpoint laboratories

Item 3 Updated in Section 1.1, *Protocol team*: team members

Item 4 Deleted Section 6.1, *Accrual restrictions*: no accrual restrictions for this study

Item 5 Revised in Section 6.4, *Statistical analyses*: minor clarifying text revisions

Item 6 Corrected in Section 6.4.4.7, *VL analyses,* 3rd paragraph: reference to secondary endpoint number

Item 7 Deleted in Section 9.3, *Enrollment and vaccination visits for Main Cohort, Immediate Vaccination Group* and Section 9.5, *Enrollment (visit 2) and visit 4 (Month 2) for Main Cohort, Delayed Vaccination Group:* sentence regarding clinical lab results

Item 8 Added in Section 9.15, *Use of electronic Diary (eDiary) for Clinical Outcomes Assessments,* and Appendices F through G, *Procedures*: end of study questionnaire regarding COVID vaccines received outside the study

Item 9 Added in Appendices A, B, and C, *Sample informed consent forms*, section "Being in the study": participants will take an end of study questionnaire

Item 10 Added in Appendix B, *Sample informed consent form for Prospective Close Contact (PCC) cohort*, and Appendix C, *Sample informed consent form for Case-Ascertained Close Contact (CACC) cohort*, section "Being in the study": applicable participants will receive SARS-CoV-2 screening at the University about two times per week.

Item 11 Deleted in Appendices A, B, and C, *Sample informed consents,* second sentence*:* reference to alternate study name of "vPROTECT"

Item 12 Added in Appendix A, *Sample informed consent form for the main cohort:* additional information for participant regarding text or email reminders for nasal swabs

Item 13 Deleted in Appendices A, B and C, *Sample informed consents:* reference to "electronically" for signature

Item 14 Deleted in Appendices A, B and C, *Sample informed consents*, signature table: column for denoting time of signature

Item 15 Deleted in Appendix A, *Sample informed consent form for the main cohort*, section 8: redundant information

Item 16 Corrected in in Appendix A, *Sample informed consent form for the main cohort*, section10: VAERS reporting requirements

Item 17 Revised in Appendices F and G: table text and associated footnotes

Item 18 Revised Appendix H, *Visit windows,* Case-Ascertained Close Contact (CACC) cohort, to include applicable Prospective Close Contact (PCC) participants who test positive for SARS-CoV-2 or whose Main Cohort study contact tests positive for SARS-CoV-2

Item 19 Revised throughout protocol: minor grammatical, editorial, and formatting corrections.

Item 20 Updated Title page, Table of Contents, Section 13, *Version history*, and Appendix I*, Protocol signature page*: dates, version number, and contents of this amendment

Date: February 17, 2021

Protocol version:1.0
Protocol modification: Not applicable

Original protocol

Protocol Signature Page

A randomized controlled study to assess SARS CoV-2 infection, viral shedding, and subsequent potential transmission in individuals immunized with Moderna COVID-19 Vaccine

I will conduct the study in accordance with the provisions of this protocol and all applicable protocol-related documents. I agree to conduct this study in compliance with United States (US) Health and Human Service regulations (45 CFR 46); applicable U.S. Food and Drug Administration regulations; standards of the International Conference on Harmonization Guideline for Good Clinical Practice (E6); Institutional Review Board/Ethics Committee determinations; all applicable in-country, state, and local laws and regulations; and other applicable requirements (e.g., US National Institutes of Health, Division of AIDS) and institutional policies

Investigator of Record Name (print) Investigator of Record Signature Date

DAIDS Protocol Number: CoVPN 3006

DAIDS Protocol Version: Version 4.0

Protocol Date: May 20, 2021
