# Supplementary material for: Efficacy of Messenger RNA–1273 Against Severe Acute Respiratory Syndrome Coronavirus 2 Acquisition in Young Adults From March to December 2021
Source: Open Forum Infect Dis. 2023 Nov 2;10(11):ofad511. doi: 10.1093/ofid/ofad511 (PMC10655942; doi:10.1093/ofid/ofad511)
Supplement: ofad511_Supplementary_Data [file ofad511_supplementary_data.zip › Manuscript Supplement Revisions.docx]

**3006 Supplemental Materials**

*Protocols*

See additional PDF.

*Statistical Methods*

**Baseline covariate adjustment**

Baseline covariates were selected for inclusion in the primary analysis of VE based on univariate screening for their association with outside vaccination or SARS-CoV-2 infection absent vaccination. Specifically, univariate Cox proportional hazards models on the calendar time scale were fit with outside vaccination as the outcome; participants without outside vaccination were censored at the last study contact, using baseline SARS-CoV-2 negative participants from the SoC group, where outside vaccination censoring was used. The models stratified on study site. Baseline variables with nominally significant associations (Wald p < 0.10) were identified. Similarly, univariate Cox proportional hazards models were fit with PCR infection as the outcome and outside vaccination censoring, using baseline SARS-CoV-2 negative participants from the SoC group. Baseline variables with nominally significant associations (Wald p < 0.10) were identified. After excluding highly correlated variables (Spearman correlation > 0.9), a Cox regression with lasso penalty was fit to derive a ‘SARS-CoV-2 risk score’, with PCR infection as the outcome and outside vaccination censoring, using baseline SARS-CoV-2 negative participants from the SoC group, and including variables nominally associated with infection and not screened out.

The following baseline variables were considered: Age, sex, race/ethnicity, place of residence, attributes from the Baseline SARS-CoV-2 Infection Risk Assessment. The variables identified as nominally associated with outside vaccination were sex at birth, residence (apartment building or condo, dormitory/campus housing/fraternity/sorority, stand-alone house, other), sports team participation (yes/no), mask wearing (never, rarely, occasionally, often, most of the time, all of the time). The variables that comprised the SARS-CoV-2 risk score were sex at birth and race (under-represented minority, yes/no); thus, the risk score was omitted from further analysis and the ‘race’ variable was used instead.

*Set of qualifying COVID-19 Symptoms*

At least one of the following: hospitalization, fever, chills, cough, shortness of breath, difficulty breathing, tiredness/fatigue, muscle aches, joint aches, body aches, headache, change in sense of taste, change in sense of smell, sore throat, nasal congestion, runny nose, nausea, vomiting, diarrhea, oral ulcers (mouth sores) and clinical or radiographical evidence of pneumonia.

**SUPPLEMENTAL TABLES**

**Table S1. Median SARS-CoV-2 free follow-up (days) by analysis set, group and censoring approach**

|  | **Analysis Set** |  | **Group** |  | **Censoring** |  |  |
| --- | --- | --- | --- | --- | --- | --- | --- |
|  |  |  |  |  | *Outside Vacc.* | *ITT* |  |
|  | *Baseline negative* |  | *Immediate* |  | 105 | 105 |  |
|  | *Baseline negative* |  | *SoC* |  | 44 | 102 |  |
|  | *Baseline negative* |  | *Vaccine Declined* |  | 58 | 65 |  |
|  | *FAS­P* |  | *Immediate* |  | 104 | 104 |  |
|  | *FAS­P* |  | *SoC* |  | 43 | 102 |  |
|  | *FAS­P* |  | *Vaccine Declined* |  | 60 | 65 |  |

**Table S2. Median and Mean Weekly Study Nasal Swab Adherence (%) by Study Week and Group**

|  | **Immediate** | | | **SoC** | | | **Vaccine Declined** | | |
| --- | --- | --- | --- | --- | --- | --- | --- | --- | --- |
| **Study week** | **N*** | **Mean** | **Median** | **N*** | **Mean** | **Median** | **N*** | **Mean** | **Median** |
|  | | | | | | | | | |
| Week 1 | 723 | 85% | 100% | 707 | 78% | 100% | 468 | 81% | 86% |
| Week 2 | 719 | 77% | 86% | 612 | 69% | 86% | 466 | 72% | 86% |
| Week 3 | 719 | 72% | 86% | 544 | 63% | 71% | 458 | 66% | 86% |
| Week 4 | 718 | 68% | 86% | 521 | 61% | 71% | 451 | 63% | 71% |
| Week 5 | 716 | 65% | 71% | 514 | 56% | 71% | 442 | 57% | 71% |
| Week 6 | 714 | 62% | 71% | 498 | 51% | 57% | 437 | 53% | 71% |
| Week 7 | 713 | 57% | 71% | 477 | 49% | 57% | 428 | 48% | 57% |
| Week 8 | 713 | 55% | 57% | 462 | 46% | 43% | 424 | 46% | 43% |
| Week 9 | 713 | 54% | 57% | 447 | 45% | 43% | 405 | 44% | 43% |
| Week 10 | 713 | 53% | 57% | 438 | 42% | 43% | 390 | 43% | 29% |
| Week 11 | 712 | 51% | 57% | 434 | 40% | 29% | 369 | 41% | 29% |
| Week 12 | 712 | 48% | 57% | 426 | 37% | 14% | 356 | 38% | 29% |
| Week 13 | 711 | 46% | 43% | 422 | 35% | 0% | 314 | 39% | 14% |
| Week 14 | 706 | 44% | 43% | 415 | 31% | 0% | 292 | 35% | 14% |
| Week 15 | 695 | 43% | 43% | 413 | 28% | 0% | 249 | 34% | 0% |
| Week 16 | 650 | 39% | 14% | 406 | 26% | 0% | 209 | 30% | 0% |
|  | | | | | | | | | |
| **Weeks 1-16** | **725** | **59%** | **64%** | **728** | **64%** | **74%** | **470** | **58%** | **61%** |

*N=all participants before the minimum of the first outside vaccination date, the termination date, and the Dec. 31, 2021, swabbing cutoff date. Full analysis set. Expected swabbing starting from first swab date (or enrollment if no swab).

**Table S3. Incidence of SARS-CoV-2 Infection and Adjusted Vaccine Efficacy by Group and Vaccine Receipt with ITT Censoring**

| **Group** | **Exposure time-period** | **# of Participants contributing person-time** | **Number of Incident Infections** | **Person­ Years (PY)** | **Incidence Rate Per 100 PY (95% CI)** | **Adjusted Estimates of Vaccine Efficacy against SARS-CoV-2 infection* (95% CI)** |
| --- | --- | --- | --- | --- | --- | --- |
| Immediate | Before vaccination | 523 | 0 | 5.9 | 0.0 (0.0, -63.2) | N/A |
|  | After 1 dose | 573 | 13 | 43.8 | 29.7 (15.8­, 50.7) | ­27.0% (­173.0%, 40.9%) |
|  | After 2 doses | 489 | 11 | 89.9 | 12.2 (6.1, 21.9) | 37.9% (­40.1%, 72.5%) |
| SoC | Unvaccinated | 549 | 29 | 125.0 | 23.2 (15.5, 33.3) | N/A |
|  |  |  |  |  |  |  |
| Vaccine Declined | Unvaccinated | 311 | 45 | 54.4 | 82.7 (60.3, 110.7) | N/A |

*Baseline SARS-CoV-2 negative subset of full analysis set with PCR data. ITT censoring.

*Cox proportional hazard models of incident SARS­CoV­2 infection event on the calendar time scale, stratified by site and adjusted for sex, residence, team sport participation at baseline, mask wearing at baseline and SARS­CoV­2 exposure risk score.

95% confidence intervals calculated by Wald method.

**Table S4. Incidence of SARS­CoV-2 Infection and Adjusted Vaccine Efficacy by Group and Vaccine Receipt for FAS-P Participants**

| **Group** | **Exposure**  **time-period** | **Censoring** | **# of**  **Participants contributing to person-time** | **# of Incident**  **Infections** | **Person­ Years (PY)** | **Incidence Rate Per 100 PY**  **(95% CI)** | **Adjusted estimates of VE against acquisition of SARS­CoV­2 infection.** |
| --- | --- | --- | --- | --- | --- | --- | --- |
| Immediate | Before vaccination | Outside vacc. | 607 | 1 | 5.7 | 17.6 (0.4, 98.1) | N/A |
|  |  | ITT | 609 | 1 | 7.2 | 13.8 (0.4, 77.1) | N/A |
|  | After 1 dose | Outside vacc. | 657 | 14 | 50.5 | 27.7 (15.1, 46.5) | ­13.4% (­142.3%, 46.9%) |
|  |  | ITT | 657 | 14 | 50.5 | 27.7 (15.1, 46.5) | ­31.9% (­172.2%, 36.1%) |
|  | After 2 doses | Outside vacc. | 563 | 12 | 102.6 | 11.7 (6.1, 20.4) | 41.2% (­37.7%, 74.9%) |
|  |  | ITT | 563 | 12 | 102.6 | 11.7 (6.1, 20.4) | 25.7% (­63.0%, 66.1%) |
| SoC | Unvaccinated | Outside vacc. | 622 | 25 | 88.3 | 28.3 (18.3, 41.8) | N/A |
|  |  | ITT | 642 | 30 | 143.5 | 20.9 (14.1, 29.8) | N/A |
| Vaccine Declined | Unvaccinated | Outside vacc. | 452 | 56 | 73.7 | 76.0 (57.4, 98.7) | N/A |
|  |  | ITT | 452 | 56 | 79.4 | 70.5 (53.2, 91.5) | N/A |

FAS­P = Full analysis set with PCR data. Outside vaccination censoring.

proportional hazard models of incident SARS­CoV­2 infection event on the calendar time scale, stratified by site and adjusted for sex, residence, team sport participation at baseline, mask wearing at baseline and SARS­CoV­2 exposure risk score.

95% confidence intervals calculated by Wald method.

**Table S5. Variant for incident SARS­-COV-2 infections by Group and Vaccine Receipt**

Immediate SoC Vaccine Declined Total

| **Variant(s)** | **Single**  **dose** | **Two**  **doses** |  | **Unvaccinated** |  | **Unvaccinated** |  | | |  |
| --- | --- | --- | --- | --- | --- | --- | --- | --- | --- | --- |
| *Alpha* | 3 | 0 |  | 2 |  | 0 |  | 5 |  | |
| *Alpha+Delta* | 0 | 0 |  | 0 |  | 1 |  | 1 |  | |
| *Alpha+Wuhan* | 1 | 0 |  | 0 |  | 0 |  | 1 |  | |
| *Delta* | 6 | 3 |  | 10 |  | 21 |  | 40 |  | |
| *Gamma* | 0 | 0 |  | 1 |  | 0 |  | 1 |  | |
| *Mu* | 0 | 1 |  | 0 |  | 0 |  | 1 |  | |
| *Unavailable* | 3 | 7 |  | 11 |  | 23 |  | 44 |  | |
| *Total* | 13 | 11 |  | 24 |  | 45 |  | 93 |  | |

Baseline SARS­CoV­2 negative participants. Outside vaccination censoring.

Includes successful and borderline variant calls (either non-suspicious or suspicious).

“Suspicious” indicates that a sample was deemed suspicious for contamination based on phylogenetic tree analysis.

**Table S6. Incidence of COVID-19 Disease by Group and Vaccine Receipt**

| **Group** | **Exposure time-period** | **Censoring** | **Number of**  **Participants contributing person-time** | **Number of Incident**  **Infections** | **Person­ Years (PY)** | **Incidence Rate Per 100 PY**  **(95% CI)** |
| --- | --- | --- | --- | --- | --- | --- |
| Immediate | Before vaccination | Outside vacc. | 521 | 0 | 4.8 | 0.0 (0.0, 77.3) |
|  |  | ITT | 523 | 0 | 5.9 | 0.0 (0.0, 62.9) |
|  | After 1 dose | Outside vacc. | 573 | 4 | 43.8 | 9.1 (2.5, 23.4) |
|  |  | ITT | 573 | 4 | 43.8 | 9.1 (2.5, 23.4) |
|  | After 2 doses | Outside vacc. | 489 | 4 | 89.9 | 4.5 (1.2, 11.4) |
|  |  | ITT | 489 | 4 | 89.9 | 4.5 (1.2, 11.4) |
| SoC | Unvaccinated | Outside vacc. | 531 | 14 | 75.5 | 18.5 (10.1, 31.1) |
|  |  | ITT | 549 | 15 | 124.9 | 12.0 (6.7, 19.8) |
| Vaccine Declined | Unvaccinated | Outside vacc. | 311 | 27 | 50.2 | 53.8 (35.5, 78.3) |
|  |  | ITT | 311 | 27 | 54.4 | 49.7 (32.7, 72.3) |

*Baseline SARS-CoV-2 negative subset of full analysis set. Outside vaccination censoring.

**Table S7. Adjusted Estimates of Vaccine Efficacy against COVID-19 Disease**

|  |  | **1 study dose** | **2 study doses** |
| --- | --- | --- | --- |
| **Set** | **Censoring** | **VE (95% CI)** | **VE (95% CI)** |
| SARS-CoV-2  negative | Outside vaccination | 54.4% (-60.8%, 87.1%) | 71.0% (-9.5%, 92.3%) |
|  | ITT | 39.9% (-100.2%, 82.0%) | 46.0% (-81.9%, 84.0%) |
| FAS-P | Outside vaccination | 54.3% (-61.9%, 87.1%) | 70.1% (-15.0%, 92.2%) |
|  | ITT | 39.8% (-101.0%, 82.0%) | 44.0% (-89.8%, 83.5%) |

Baseline SARS-CoV-2 and FAS-P participants. FAS-P, full analysis set with PCR data. Outside vaccination censoring.

Cox proportional hazard models of incident COVID-19 disease event on the calendar time scale, stratified by site and adjusted for sex, residence, team sport participation at baseline, mask wearing at baseline and SARS-CoV-2 exposure risk score. 95% confidence intervals calculated by Wald method. Data cutoff date: Jun 02, 2022.

**Table S8. Baseline Lifestyle Circumstances related to COVID-19**

|  | **Total** | **Immediate** | **SoC** | **Vaccine Declined** |
| --- | --- | --- | --- | --- |
| **Total baseline SARS-CoV-2 negative** | 1460 | 600 | 549 | 311 |
| **Number of roommates (sleep in same room)** |  |  |  |  |
| 0 | 904 (61.9%) | 392 (65.3%) | 362 (65.9%) | 150 (48.2%) |
| 1 | 451 (30.9%) | 170 (28.3%) | 157 (28.6%) | 124 (39.9%) |
| >1 | 100 (6.8%) | 37 (6.2%) | 29 (5.3%) | 34 (10.9%) |
| **In-person work, volunteer, or on-campus study time** |  |  |  |  |
| 0 days/week | 497 (34.0%) | 217 (36.2%) | 192 (35.0%) | 88 (28.3%) |
| 1 day/week | 143 (9.8%) | 75 (12.5%) | 56 (10.2%) | 12 (3.9%) |
| 2-4 days/week | 483 (33.1%) | 207 (34.5%) | 187 (34.1%) | 89 (28.6%) |
| 5 or more days/week | 332 (22.7%) | 100 (16.7%) | 113 (20.6%) | 119 (38.3%) |
| **Member of a fraternity or sorority** |  |  |  |  |
| Yes | 136 (9.3%) | 59 (9.8%) | 68 (12.4%) | 9 (2.9%) |
| No | 1319 (90.3%) | 540 (90.0%) | 480 (87.4%) | 299 (96.1%) |
| **Current participation in any in-person team sport** |  |  |  |  |
| Yes | 104 (7.1%) | 40 (6.7%) | 44 (8.0%) | 20 (6.4%) |
| No | 1351 (92.5%) | 559 (93.2%) | 504 (91.8%) | 288 (92.6%) |
| **In last 2 weeks, sat inside a bar, restaurant, cafe, cafeteria** |  |  |  |  |
| Yes | 1012 (69.3%) | 400 (66.7%) | 367 (66.8%) | 245 (78.8%) |
| No | 443 (30.3%) | 199 (33.2%) | 181 (33.0%) | 63 (20.3%) |
| **In last 2 weeks, usual alcohol consumption** |  |  |  |  |
| Everyday | 8 (0.5%) | 1 (0.2%) | 2 (0.4%) | 5 (1.6%) |
| 5-6 times per week | 14 (1.0%) | 5 (0.8%) | 4 (0.7%) | 5 (1.6%) |
| 3-4 times per week | 82 (5.6%) | 34 (5.7%) | 29 (5.3%) | 19 (6.1%) |
| Twice per week | 190 (13.0%) | 84 (14.0%) | 75 (13.7%) | 31 (10.0%) |
| Once per week | 196 (13.4%) | 91 (15.2%) | 69 (12.6%) | 36 (11.6%) |
| Once every other week | 258 (17.7%) | 94 (15.7%) | 97 (17.7%) | 67 (21.5%) |
| None | 707 (48.4%) | 290 (48.3%) | 272 (49.5%) | 145 (46.6%) |
| **In last 2 weeks, usual number of drinks when consuming alcohol** |  |  |  |  |
| 7 drinks or more | 15 (1.0%) | 3 (0.5%) | 9 (1.6%) | 3 (1.0%) |
| 4-6 drinks | 136 (9.3%) | 54 (9.0%) | 51 (9.3%) | 31 (10.0%) |
| 1-3 drinks | 589 (40.3%) | 250 (41.7%) | 214 (39.0%) | 125 (40.2%) |
| Did not drink alcohol | 715 (49.0%) | 292 (48.7%) | 274 (49.9%) | 149 (47.9%) |
| **In last 2 weeks, potential exposure to SARS-CoV-2** |  |  |  |  |
| Yes, someone with a recent positive test | 23 (1.6%) | 4 (0.7%) | 9 (1.6%) | 10 (3.2%) |
| Yes, someone with possible symptoms, but no diagnosis or test | 7 (0.5%) | 0 (0.0%) | 4 (0.7%) | 3 (1.0%) |
| Yes, third party notification system | 5 (0.3%) | 0 (0.0%) | 1 (0.2%) | 4 (1.3%) |
| No, not to my knowledge | 1421 (97.3%) | 596 (99.3%) | 534 (97.3%) | 291 (93.6%) |
| **In last 2 weeks when leaving home, worn a mask inside when around other people** |  |  |  |  |
| Never | 100 (6.8%) | 20 (3.3%) | 23 (4.2%) | 57 (18.3%) |
| Rarely | 111 (7.6%) | 25 (4.2%) | 29 (5.3%) | 57 (18.3%) |
| Occasionally | 161 (11.0%) | 48 (8.0%) | 54 (9.8%) | 59 (19.0%) |
| Often | 149 (10.2%) | 58 (9.7%) | 53 (9.7%) | 38 (12.2%) |
| Most of the time | 364 (24.9%) | 162 (27.0%) | 153 (27.9%) | 49 (15.8%) |
| All of the time | 570 (39.0%) | 286 (47.7%) | 236 (43.0%) | 48 (15.4%) |
| **In last 2 weeks when leaving home, maintained physical distancing from others** |  |  |  |  |
| Never | 46 (3.2%) | 9 (1.5%) | 9 (1.6%) | 28 (9.0%) |
| Rarely | 86 (5.9%) | 24 (4.0%) | 20 (3.6%) | 42 (13.5%) |
| Occasionally | 185 (12.7%) | 53 (8.8%) | 55 (10.0%) | 77 (24.8%) |
| Often | 254 (17.4%) | 110 (18.3%) | 96 (17.5%) | 48 (15.4%) |
| Most of the time | 555 (38.0%) | 243 (40.5%) | 239 (43.5%) | 73 (23.5%) |
| All of the time | 329 (22.5%) | 160 (26.7%) | 129 (23.5%) | 40 (12.9%) |
| **In last 2 weeks when leaving home, met with others in a group of 10 or more** |  |  |  |  |
| Never | 584 (40.0%) | 273 (45.5%) | 240 (43.7%) | 71 (22.8%) |
| Rarely | 501 (34.3%) | 188 (31.3%) | 205 (37.3%) | 108 (34.7%) |
| Occasionally | 246 (16.8%) | 103 (17.2%) | 74 (13.5%) | 69 (22.2%) |
| Often | 84 (5.8%) | 23 (3.8%) | 23 (4.2%) | 38 (12.2%) |
| Most of the time | 17 (1.2%) | 3 (0.5%) | 5 (0.9%) | 9 (2.9%) |
| All of the time | 23 (1.6%) | 9 (1.5%) | 1 (0.2%) | 13 (4.2%) |
| **In last 2 weeks when leaving home, encountered people not wearing masks** |  |  |  |  |
| Never | 83 (5.7%) | 44 (7.3%) | 32 (5.8%) | 7 (2.3%) |
| Rarely | 290 (19.9%) | 149 (24.8%) | 123 (22.4%) | 18 (5.8%) |
| Occasionally | 470 (32.2%) | 207 (34.5%) | 205 (37.3%) | 58 (18.6%) |
| Often | 297 (20.3%) | 110 (18.3%) | 111 (20.2%) | 76 (24.4%) |
| Most of the time | 166 (11.4%) | 51 (8.5%) | 41 (7.5%) | 74 (23.8%) |
| All of the time | 149 (10.2%) | 38 (6.3%) | 36 (6.6%) | 75 (24.1%) |

Total baseline SARS-CoV-2 negative. Due to missing data, percentages may not sum to 100%.
